# The 2021 report of the *Lancet* Countdown on health and climate change: code red for a healthy future

**DOI:** 10.1016/S0140-6736(21)01787-6

**Published:** 2021-10-20

**Authors:** Marina Romanello, Alice McGushin, Claudia Di Napoli, Paul Drummond, Nick Hughes, Louis Jamart, Harry Kennard, Pete Lampard, Baltazar Solano Rodriguez, Nigel Arnell, Sonja Ayeb-Karlsson, Kristine Belesova, Wenjia Cai, Diarmid Campbell-Lendrum, Stuart Capstick, Jonathan Chambers, Lingzhi Chu, Luisa Ciampi, Carole Dalin, Niheer Dasandi, Shouro Dasgupta, Michael Davies, Paula Dominguez-Salas, Robert Dubrow, Kristie L Ebi, Matthew Eckelman, Paul Ekins, Luis E Escobar, Lucien Georgeson, Delia Grace, Hilary Graham, Samuel H Gunther, Stella Hartinger, Kehan He, Clare Heaviside, Jeremy Hess, Shih-Che Hsu, Slava Jankin, Marcia P Jimenez, Ilan Kelman, Gregor Kiesewetter, Patrick L Kinney, Tord Kjellstrom, Dominic Kniveton, Jason K W Lee, Bruno Lemke, Yang Liu, Zhao Liu, Melissa Lott, Rachel Lowe, Jaime Martinez-Urtaza, Mark Maslin, Lucy McAllister, Celia McMichael, Zhifu Mi, James Milner, Kelton Minor, Nahid Mohajeri, Maziar Moradi-Lakeh, Karyn Morrissey, Simon Munzert, Kris A Murray, Tara Neville, Maria Nilsson, Nick Obradovich, Maquins Odhiambo Sewe, Tadj Oreszczyn, Matthias Otto, Fereidoon Owfi, Olivia Pearman, David Pencheon, Mahnaz Rabbaniha, Elizabeth Robinson, Joacim Rocklöv, Renee N Salas, Jan C Semenza, Jodi Sherman, Liuhua Shi, Marco Springmann, Meisam Tabatabaei, Jonathon Taylor, Joaquin Trinanes, Joy Shumake-Guillemot, Bryan Vu, Fabian Wagner, Paul Wilkinson, Matthew Winning, Marisol Yglesias, Shihui Zhang, Peng Gong, Hugh Montgomery, Anthony Costello, Ian Hamilton

**Affiliations:** Institute for Global Health, University College London, London, UK; Institute for Global Health, University College London, London, UK; School of Agriculture, Policy and Development, University of Reading, Reading, UK; Institute for Sustainable Resources, University College London, London, UK; Institute for Sustainable Resources, University College London, London, UK; Institute for Global Health, University College London, London, UK; UCL Energy Institute, University College London, London, UK; Department of Health Sciences, University of York, York, UK; UCL Energy Institute, University College London, London, UK; Department of Meteorology, University of Reading, Reading, UK; Institute for Environment and Human Security, United Nations University, Bonn, Germany; Centre on Climate Change and Planetary Health, London School of Hygiene & Tropical Medicine, London, UK; Department of Earth System Science, Tsinghua University, Beijing, China; Department of Environment, Climate Change and Health, World Health Organization, Geneva, Switzerland; Centre for Climate Change and Social Transformations, School of Psychology, Cardiff University, Cardiff, UK; Institute for Environmental Sciences, World Health Organization, Geneva, Switzerland; Yale Center on Climate Change and Health, Yale University, New Haven, CT, USA; The Walker Institute, University of Reading, Reading, UK; Institute for Sustainable Resources, University College London, London, UK; School of Government, University of Birmingham, Birmingham, UK; Economic analysis of Climate Impacts and Policy, Centro Euro-Mediterraneo sui Cambiamenti Climatici, Venice, Italy; Institute for Environmental Design and Engineering, University College London, London, UK; Natural Resources Institute, University of Greenwich, London, UK; Yale Center on Climate Change and Health, Yale University, New Haven, CT, USA; Department of Global Health, University of Washington, Seattle, WA, USA; Department of Civil and Environmental Engineering, Northeastern University, Boston, MA, USA; Institute for Sustainable Resources, University College London, London, UK; Department of Fish and Wildlife Conservation, Virginia Polytechnic Institute and State University, Blacksburg, VA, USA; Department of Geography, University College London, London, UK; Animal and Human Health Program, International Livestock Research Institute, Nairobi, Kenya; Department of Health Sciences, University of York, York, UK; Human Potential Translational Research Programme, Yong Loo Lin School of Medicine, National University Singapore, Singapore; School of Public Health and Administration, Universidad Peruana Cayetano Heredia, Lima, Peru; The Bartlett School of Sustainable Construction, University College London, London, UK; Institute for Environmental Design and Engineering, University College London, London, UK; Centre for Health and the Global Environment, University of Washington, Seattle, WA, USA; UCL Energy Institute, University College London, London, UK; Data Science Lab, Hertie School, Berlin, Germany; Department of Epidemiology, Harvard T H Chan School of Public Health, Boston, MA, USA; Institute for Global Health, University College London, London, UK; Air Quality and Greenhouse Gases Programme, International Institute for Applied Systems Analysis, Laxenburg, Austria; Department of Environmental Health, School of Public Health, Boston University, Boston, MA, USA; Health and Environment International Trust, Nelson, New Zealand; School of Global Studies, University of Sussex, Falmer, UK; Human Potential Translational Research Programme, Yong Loo Lin School of Medicine, National University Singapore, Singapore; School of Health, Nelson Marlborough Institute of Technology, Nelson, New Zealand; Gangarosa Department of Environmental Health, Rollins School of Public Health, Emory University, Atlanta, GA, USA; Department of Earth System Science, Tsinghua University, Beijing, China; Center on Global Energy Policy, Columbia University, New York, NY, USA; Centre for Mathematical Modelling of Infectious Diseases, London School of Hygiene & Tropical Medicine, London, UK; Department of Genetics and Microbiology, Universitat Autònoma de Barcelona, Barcelona, Spain; Department of Geography, University College London, London, UK; Center for Energy Markets, Technical University of Munich, Munich, Germany; School of Geography, Earth and Atmospheric Sciences, University of Melbourne, Melbourne, VIC, Australia; The Bartlett School of Sustainable Construction, University College London, London, UK; Department of Public Health, Environments, and Society, London School of Hygiene & Tropical Medicine, London, UK; Copenhagen Center for Social Data Science, University of Copenhagen, Copenhagen, Denmark; Institute for Environmental Design and Engineering, University College London, London, UK; Preventive Medicine and Public Health Research Center, Psychosocial Health Research Institute, Iran University of Medical Sciences, Tehran, Iran; Department of Technology, Management and Economics, Technical University of Denmark, Copenhagen, Denmark; Data Science Lab, Hertie School, Berlin, Germany; MRC Centre for Global Infectious Disease Analysis, School of Public Health, Imperial College London, UK; Department of Environment, Climate Change and Health, World Health Organization, Geneva, Switzerland; Department of Epidemiology and Global Health, Umeå University, Umeå, Sweden; Umeå University, Umeå, Sweden; Centre for Humans and Machines, Max Planck Institute for Human Development, Berlin, Germany; Section of Sustainable Health, Department of Public Health and Clinical Medicine, Umeå University, Umeå, Sweden; UCL Energy Institute, University College London, London, UK; Department of Arts, Media & Digital Technologies, Nelson Marlborough Institute of Technology, Nelson, New Zealand; Iranian Fisheries Science Research Institute, Agricultural Research, Education, and Extension Organisation, Tehran, Iran; Cooperative Institute of Research in Environmental Sciences, University of Colorado, Boulder, CO, USA; College of Medicine and Health, Exeter University, Exeter, UK; Iranian Fisheries Science Research Institute, Agricultural Research, Education, and Extension Organisation, Tehran, Iran; School of Agriculture, Policy and Development, University of Reading, Reading, UK; Section of Sustainable Health, Department of Public Health and Clinical Medicine, Umeå University, Umeå, Sweden; Harvard Medical School, Harvard University, Boston, MA, USA; Lateral Public Health Consulting, Stockholm, Sweden; Department of Anesthesiology, Yale University, New Haven, CT, USA; Gangarosa Department of Environmental Health, Rollins School of Public Health, Emory University, Atlanta, GA, USA; Oxford Martin School, University of Oxford, Oxford, UK; Higher Institution Centre of Excellence, Institute of Tropical Aquaculture and Fisheries, Universiti Malaysia Terengganu, Kuala Terengganu, Malaysia; Department of Civil Engineering, Tampere University, Tampere, Finland; Department of Electronics and Computer Science, Universidade de Santiago de Compostela, Santiago, Spain; WHOWMO Joint Climate and Health Office, Geneva, Switzerland; Gangarosa Department of Environmental Health, Rollins School of Public Health, Emory University, Atlanta, GA, USA; Air Quality and Greenhouse Gases Programme, International Institute for Applied Systems Analysis, Laxenburg, Austria; Department of Public Health, Environments, and Society, London School of Hygiene & Tropical Medicine, London, UK; Institute for Sustainable Resources, University College London, London, UK; School of Public Health and Administration, Universidad Peruana Cayetano Heredia, Lima, Peru; Department of Earth System Science, Tsinghua University, Beijing, China; Department of Geography, University of Hong Kong, Hong Kong Special Administrative Region, China; Centre for Human Health and Performance, University College London, London, UK; Institute for Global Health, University College London, London, UK; UCL Energy Institute, University College London, London, UK

## Abstract

The *Lancet* Countdown is an international collaboration that independently monitors the health consequences of a changing climate. Publishing updated, new, and improved indicators each year, the *Lancet* Countdown represents the consensus of leading researchers from 43 academic institutions and UN agencies. The 44 indicators of this report expose an unabated rise in the health impacts of climate change and the current health consequences of the delayed and inconsistent response of countries around the globe—providing a clear imperative for accelerated action that puts the health of people and planet above all else.

The 2021 report coincides with the UN Framework Convention on Climate Change 26th Conference of the Parties (COP26), at which countries are facing pressure to realise the ambition of the Paris Agreement to keep the global average temperature rise to 1·5°C and to mobilise the financial resources required for all countries to have an effective climate response. These negotiations unfold in the context of the COVID-19 pandemic—a global health crisis that has claimed millions of lives, affected livelihoods and communities around the globe, and exposed deep fissures and inequities in the world’s capacity to cope with, and respond to, health emergencies. Yet, in its response to both crises, the world is faced with an unprecedented opportunity to ensure a healthy future for all.

**Deepening inequities in a warming world:**

Record temperatures in 2020 resulted in a new high of 3·1 billion more person-days of heatwave exposure among people older than 65 years and 626 million more person-days affecting children younger than 1 year, compared with the annual average for the 1986–2005 baseline (indicator 1.1.2). Looking to 2021, people older than 65 years or younger than 1 year, along with people facing social disadvantages, were the most affected by the record-breaking temperatures of over 40°C in the Pacific Northwest areas of the USA and Canada in June, 2021—an event that would have been almost impossible without human-caused climate change. Although the exact number will not be known for several months, hundreds of people have died prematurely from the heat. Furthermore, populations in countries with low and medium levels of UN-defined human development index (HDI) have had the biggest increase in heat vulnerability during the past 30 years, with risks to their health further exacerbated by the low availability of cooling mechanisms and urban green space (indicators 1.1.1, 2.3.2, and 2.3.3). Agricultural workers in countries with low and medium HDI were among the worst affected by exposure to extreme temperatures, bearing almost half of the 295 billion potential work hours lost due to heat in 2020 (indicator 1.1.4). These lost work hours could have devastating economic consequences to these already vulnerable workers—data in this year’s report shows that the average potential earnings lost in countries in the low HDI group were equivalent to 4–8% of the national gross domestic product (indicator 4.1.3).

Through these effects, rising average temperatures, and altered rainfall patterns, climate change is beginning to reverse years of progress in tackling the food and water insecurity that still affects the most underserved populations around the world, denying them an essential aspect of good health. During any given month in 2020, up to 19% of the global land surface was affected by extreme drought; a value that had not exceeded 13% between 1950 and 1999 (indicator 1.2.2). In parallel with drought, warm temperatures are affecting the yield potential of the world’s major staple crops—a 6·0% reduction for maize; 3·0% for winter wheat; 5·4% for soybean; and 1·8% for rice in 2020, relative to 1981–2010 (indicator 1.4.1)—exposing the rising risk of food insecurity.

Adding to these health hazards, the changing environmental conditions are also increasing the suitability for the transmission of many water-borne, air-borne, food-borne, and vector-borne pathogens. Although socioeconomic development, public health interventions, and advances in medicine have reduced the global burden of infectious disease transmission, climate change could undermine eradication efforts.

The number of months with environmentally suitable conditions for the transmission of malaria (*Plasmodium falciparum*) rose by 39% from 1950–59 to 2010–19 in densely populated highland areas in the low HDI group, threatening highly disadvantaged populations who were comparatively safer from this disease than those in the lowland areas (indicator 1.3.1). The epidemic potential for dengue virus, Zika virus, and chikungunya virus, which currently primarily affect populations in central America, South America, the Caribbean, Africa, and south Asia, increased globally, with a basic reproductive rate increase of 13% for transmission by *Aedes aegypti* and 7% for transmission by *Aedes albopictus* compared with the 1950s. The biggest relative increase in basic reproductive rate of these arboviruses was seen in countries in the very high HDI group (indicator 1.3.1); however, people in the low HDI group are confronted with the highest vulnerability to these arboviruses (indicator 1.3.2).

Similar findings are observed in the environmental suitability for *Vibrio cholerae*, a pathogen estimated to cause almost 100 000 deaths annually, particularly among populations with poor access to safe water and sanitation. Between 2003 and 2019, the coastal areas suitable for *V cholerae* transmission increased substantially across all HDI country groups—although, with 98% of their coastline suitable to the transmission of *V cholerae* in 2020, it is people in the low HDI country group that have the highest environmental suitability for this disease (indicator 1.3.1).

The concurrent and interconnecting risks posed by extreme weather events, infectious disease transmission, and food, water, and financial insecurity are over-burdening the most vulnerable populations. Through multiple simultaneous and interacting health risks, climate change is threatening to reverse years of progress in public health and sustainable development.

Even with overwhelming evidence on the health impacts of climate change, countries are not delivering an adaptation response proportionate to the rising risks their populations face. In 2020, 104 (63%) of 166 countries did not have a high level of implementation of national health emergency frameworks, leaving them unprepared to respond to pandemics and climate-related health emergencies (indicator 2.3.1). Importantly, only 18 (55%) of 33 countries with a low HDI had reported at least a medium level of implementation of national health emergency frameworks, compared with 47 (89%) of 53 countries with a very high HDI. In addition, only 47 (52%) of 91 countries reported having a national adaptation plan for health, with insufficient human and financial resources identified as the main barrier for their implementation (indicator 2.1.1). With a world facing an unavoidable temperature rise, even with the most ambitious climate change mitigation, accelerated adaptation is essential to reduce the vulnerabilities of populations to climate change and protect the health of people around the world.

**An inequitable response fails everyone:**

10 months into 2021, global and equitable access to the COVID-19 vaccine had not been delivered—more than 60% of people in high-income countries have received at least one dose of a COVID-19 vaccine compared with just 3·5% of people in low-income countries. Data in this report exposes similar inequities in the global climate change mitigation response.

To meet the Paris Agreement goals and prevent catastrophic levels of global warming, global greenhouse gas emissions must reduce by half within a decade. However, at the current pace of reduction, it would take more than 150 years for the energy system to fully decarbonise (indicator 3.1), and the unequal response between countries is resulting in an uneven realisation of the health benefits of a low-carbon transition.

The use of public funds to subsidise fossil fuels is partly responsible for the slow decarbonisation rate. Of the 84 countries reviewed, 65 were still providing an overall subsidy to fossil fuels in 2018 and, in many cases, subsidies were equivalent to substantial proportions of the national health budget and could have been redirected to deliver net benefits to health and wellbeing. Furthermore, all the 19 countries whose carbon pricing policies outweighed the effect of any fossil fuels subsidies came from the very high HDI group (indicator 4.2.4).

Although countries in the very high HDI group have collectively made the most progress in the decarbonisation of the energy system, they are still the main contributors to CO_2_ emissions through the local production of goods and services, accounting for 45% of the global total (indicator 4.2.5). With a slower pace of decarbonisation and poorer air quality regulations than countries in the very high HDI group, the medium and high HDI country groups produce the most fine particle matter (PM_2·5_) emissions and have the highest rates of air pollution-related deaths, which are about 50% higher than the total deaths in the very high HDI group (indicator 3.3). The low HDI group, with comparatively lower amounts of industrial activity than in the other groups, has a local production that contributes to only 0·7% of global CO_2_ emissions, and has the lowest mortality rate from ambient air pollution. However, with only 12% of its inhabitants relying on clean fuels and technologies for cooking, the health of these populations is still at risk from dangerously high concentrations of household air pollution (indicator 3.2). Even in the most affluent countries, people in the most deprived areas over-whelmingly bear the burden of health effects from exposure to air pollution. These findings expose the health costs of the delayed and unequal mitigation response and underscore the millions of deaths to be prevented annually through a low-carbon transition that prioritises the health of all populations.

However, the world is not on track to realising the health gains of the transition to a low-carbon economy. Current global decarbonisation commitments are insufficient to meet Paris Agreement ambitions and would lead to a roughly 2·4°C average global temperature increase by the end of the century. The current direction of post-COVID-19 spending is threatening to make this situation worse, with just 18% of all the funds committed for economic recovery from the COVID-19 pandemic by the end of 2020 expected to lead to a reduction of greenhouse gas emissions. Indeed, the economic recovery from the pandemic is already predicted to lead to an unprecedented 5% increase in greenhouse gas emissions in 2021, which will bring global anthropogenic emissions back to their peak amounts.

In addition, the current economic recession is threatening to undermine the target of mobilising US$100 billion per year from 2020 onwards to promote low-carbon shifts and adaptation responses in the most underserved countries, even though this quantity is minute compared with the trillions allocated to COVID-19 recovery. The high amounts of borrowing that countries have had to resort to during the pandemic could erase their ability to deliver a green recovery and maximise the health gains to their population of a low-carbon transition.

**An unprecedented opportunity to ensure a healthy future for all:**

The overshoot in emissions resulting from a carbon-intensive COVID-19 recovery would irreversibly prevent the world from meeting climate commitments and the Sustainable Development Goals and lock humanity into an increasingly extreme and unpredictable environment. Data in this report expose the health impacts and health inequities of the current world at 1·2°C of warming above pre-industrial levels and supports that, on the current trajectory, climate change will become the defining narrative of human health.

However, by directing the trillions of dollars that will be committed to COVID-19 recovery towards the WHO’s prescriptions for a healthy, green recovery, the world could meet the Paris Agreement goals, protect the natural systems that support wellbeing, and minimise inequities through reduced health effects and maximised co-benefits of a universal low-carbon transition. Promoting equitable climate change mitigation and universal access to clean energies could prevent millions of deaths annually from reduced exposure to air pollution, healthier diets, and more active lifestyles, and contribute to reducing health inequities globally. This pivotal moment of economic stimulus represents a historical opportunity to secure the health of present and future generations.

There is a glimpse of positive change through several promising trends in this year’s data: electricity generation from renewable wind and solar energy increased by an annual average of 17% between 2013 and 2018 (indicator 3.1); investment in new coal capacity decreased by 10% in 2020 (indicator 4.2.1); and the global number of electric vehicles reached 7·2 million in 2019 (indicator 3.4). Additionally, the global pandemic has driven increased engagement in health and climate change across multiple domains in society, with 91 heads of state making the connection in the 2020 UN General Debate and newly widespread engagement among countries in the very high HDI group (indicator 5.4). Whether COVID-19 recovery supports, or reverses these trends, is yet to be seen.

Neither COVID-19 nor climate change respect national borders. Without widespread, accessible vaccination across all countries and societies, SARS-CoV-2 and its new variants will continue to put the health of everybody at risk. Likewise, tackling climate change requires all countries to deliver an urgent and coordinated response, with COVID-19 recovery funds allocated to support and ensure a just transition to a low-carbon future and climate change adaptation across the globe. Leaders of the world have an unprecedented opportunity to deliver a future of improved health, reduced inequity, and economic and environmental sustainability. However, this will only be possible if the world acts together to ensure that no person is left behind.

## Introduction

The COVID-19 pandemic has changed societies in previously unimaginable ways, with deepening and widespread concerns about global health security, inequities, and anthropogenic influences on the environment. As of May 11, 2021, the pandemic has resulted in almost 191 million cases and 4·1 million deaths,^1,2^ and its multidimensional impacts on health and wellbeing, together with its disruption to work, social, and leisure activities, still continue. The overwhelming demand for health care caused 94 of the 105 countries examined to have disruptions to the delivery of essential health services, undermining health and wellbeing.^3^ COVID-19 led to a worldwide economic recession; an estimated 90 million people were pushed below the extreme poverty threshold in 2020,^4,5^ and pandemic-induced borrowing by the World Trade Organization’s so-called developing countries amounted to US$130 billion by July, 2020.^6^

While the world’s attention has been diverted towards the ongoing acute health crisis, the health effects of human-induced climate change continue to increase. Climate change contributed to the unusually high temperatures seen during 2020 in the UK and Siberia; the record-breaking heatwave that affected populations across the Pacific Northwest areas of the USA and Canada in June, 2021, which caused more than 1000 deaths (a number expected to increase); accelerated glacier retreat that is putting the Huaraz (Peru) under imminent flooding risk; and Australia’s devastating 2019–20 bushfire season.^7–11^ During a 6 month period in 2020, 84 disasters from floods, droughts, and storms affected 51·6 million people in countries already struggling with COVID-19,^12^ with the escalating impacts of disasters reducing their ability to respond to health emergencies. Additionally, climate impacts might undermine the capacity of countries to repay their debts, hindering their progress towards the Sustainable Development Goals (SDGs).^13,14^ As with COVID-19, the health impacts of climate change are inequitable, with disproportionate effects on the most susceptible populations in every society, including people with low incomes, members of minority groups, women, children, older adults, people with chronic diseases and disabilities, and outdoor workers.^15^ Relationships between climate change and COVID-19 provide ongoing evidence of the interconnectedness of the world and the health consequences of inequities. This report depicts the synergies and interactions between these two crises. The world is now 1·2°C warmer than in the pre-industrial period (1850–1900), the past 7 years have been the hottest 7 years on record, and 2020 tied with 2016 as the hottest year yet.^16–18^ Atmospheric CO_2_ concentrations have reached a concerning milestone and are now 50% higher than in the pre-industrial era.^19^ Changes, such as reduced soil moisture, could limit the Earth’s carbon reuptake, resulting in increased CO_2_ concentrations in the atmosphere.^20^ Furthermore, some critical tipping points are close or might have been surpassed, which could destabilise the Earth’s climate system.^21,22^ Although the large reductions in transport use and industrial manufacturing during the pandemic resulted in energy-related emissions for 2020 falling by 5·8% (the largest annual percentage decline since World War 2), this reduction was short-lived and emissions have risen in 2021.^23–25^ Without an adequate response, the health effects of climate change will worsen throughout the coming decades.

The world now turns with hope to the 2021 UN Framework Convention on Climate Change (UNFCCC) conference in Glasgow (UN Climate Change 26th Conference of Parties; COP26), originally scheduled for 2020. Over the past year, the world has seen more ambitious climate targets from governments and businesses than before and 73% of current global emissions are now covered by emissions targets of net zero announced in May, 2021. Nevertheless, these announcements are non-binding, and, even with their full implementation, the world would be on track for a warming of roughly 2·4°C (1·9–3·0°C) since pre-industrial times by 2100.^26^

These climate announcements are being made against the backdrop of huge investments in economic recovery from COVID-19. Depending on their consistency with climate targets, these investments could take the world in one of two directions—either directing the world towards the goals of the Paris Agreement or locking it into increased emissions and climate change that will damage the health of current and future generations. As humanity faces a crucial turning point, the indicators in this report provide the health evidence to inform a global response to the impacts of climate change and to identify the considerable health, environmental, and economic benefits that would result if a so-called green recovery from COVID-19 was prioritised.

### Sixth annual report tracking progress on health and climate change

The *Lancet* Countdown is an independent, international, and multidisciplinary collaboration that monitors the health impacts of climate change, and the progress, or absence of, in the world’s response. The *Lancet* Countdown draws on the expertise of climate scientists, economists, energy and transport experts, social and political scientists, public health experts and health professionals, and others, spanning 43 academic and UN institutions. Together, these contributors report on 44 indicators that are organised in five domains: climate change impacts, exposures, and vulnerabilities; adaptation, planning, and resilience for health; mitigation actions and health co-benefits; economics and finance; and public and political engagement.

The *Lancet* Countdown’s indicator domains were selected through an open, global consultation process that identified scientifically documented links between health and climate change, with indicators developed according to well-established methods and the availability of reliable and regularly updated data with adequate geographical and temporal scales.^27^ Each year, the indicators have been improved through an open, iterative, and adaptive approach, and new indicators have been introduced to provide an increasingly complete picture of the health dimensions of climate change. For the 2020 and 2021 reports, all new indicators underwent an independent assessment process led by world experts before the formal peer review, adding rigour and transparency to the collaboration’s research. Existing indicators are undergoing a similar, independent quality improvement process, aimed at ensuring they continue to use the best available data and methods.

Three new indicators are added to the 2021 report: incorporating considerations of mental wellbeing by tracking the effect of heat on expressed online sentiment; capturing the influence of heat on safe physical activity; and tracking consumption-based greenhouse gas and fine particle matter (PM_2·5_) emissions. Most of the pre-existing indicators underwent major improvements, with strengthened methods, datasets, and metrics and expanded geographical and temporal coverage. All indicators, including their methods, data sources, caveats, and plans for future improvements, are described in detail in appendix 5 (an essential manual for this report). The indicators for the 2021 report are listed in panel 1.

Each indicator, wherever possible and appropriate, is disaggregated into very high, high, medium, and low human development index (HDI) country groups, as defined by the UNDP, in the latest year that data were available (2019).^28^ This composite HDI captures three dimensions: a long and healthy life (with life expectancy as a proxy), education (captured by the mean of years of schooling), and standard of living (measured by per-capita gross national income).^28^ In line with the priorities of *The Lancet*’s Diversity Board, gender disparities are also considered wherever relevant. However, a scarcity of gender-disaggregated data means that few indicators can capture these differences quantitatively and often do so using sex disaggregation as a proxy for gender (see panel 2).

The COVID-19 pandemic will alter the trends of many of the indicators reported; some of these trends can be identified in this report and others will become apparent in the coming years. COVID-19 has also altered population demographics, mortality rates, and the structure and size of the labour force. These changes are not reflected in the current indicators, presenting methodological challenges in the assessment of the health impacts of climate change. How the COVID-19 pandemic affects the methods and assumptions of the *Lancet* Countdown’s indicators will become clearer in future reports as more data will be available.

The global reach of the *Lancet* Countdown is expanding. Two regional offices, one in South America (Universidad Peruana Cayetano Heredia, Lima, Peru) and one in Asia (Tsinghua University, Beijing, China), were established in 2020 and an office in Europe was established in 2021 (Barcelona Supercomputing Centre, Barcelona, Spain). These regional collaborators contributed indicators to the 2021 report and are working on nationally-relevant and regionally-relevant health and climate change research, accompanied by local communications and policy engagement. A third regional office, based at the University of the West Indies (Kingston, Jamaica), was established in September, 2021, and aims to build on the network and evidence base of health and climate change in small island developing states (SIDS). The *Lancet* Countdown is also working in collaboration with the European Environment Agency, incorporating policy-relevant data from its indicators into the European Climate and Health Observatory.

National and regional reports were published for Australia (in partnership with the *Medical Journal of Australia*), China, and SIDS.^49–51^ For the third year, the data underpinning each of the *Lancet* Countdown’s indicators have been shared through an online data visualisation platform, where they can be explored at finer spatial and temporal scales.

The work of this collaboration is driven by the ongoing support from *The Lancet* and the Wellcome Trust, the *Lancet* Countdown’s scientific advisory group and higher-level advisory board, and, importantly, the *Lancet* Countdown’s authors and collaborators. The collaboration welcomes offers of support from new experts and new institutions willing to build on this analysis as the *Lancet* Countdown monitors the world’s response to the health effects of climate change during this decade.

## Section 1: climate change impacts, exposures, and vulnerability

Climate change threatens human health and wellbeing through effects on weather, ecosystems, and human systems. These effects increase exposure to extreme events, change the environmental suitability for infectious disease transmission, alter population movements, and undermine people’s livelihoods and mental health.^52–56^ The resulting strains on health and social systems disproportionately affect the most disadvantaged in society, with climate change amplifying inequities.^52,53^

Section 1 of the 2021 report monitors the health impacts of climate change, with indicators tracking climate hazards, human exposure and vulnerabilities to climate hazards, and the resulting health outcomes of these. The first group of indicators addresses the direct implications of rising temperatures for health, exploring changes in the exposure and vulnerabilities of populations around the world to extreme heat and its impacts on health and wellbeing (indicators 1.1.1–1.1.6, see panel 1). Each of these indicators takes gridded heat data and overlays them with relevant exposure and vulnerability data to reflect health outcomes. Two new indicators have been introduced since the 2020 report.^53^ One of these indicators shows the effect of heat on time available for safe outdoor exercise (indicator 1.1.3) and the other indicator approaches the challenge of assessing the influence of extreme heat on sentiment with Twitter data to capture people’s online expressions (indicator 1.1.5).^57^

The second group of indicators in this section sheds light on climate-sensitive extreme events, tracking exposure to wildfire and wildfire risk (indicator 1.2.1), the incidence of droughts (indicator 1.2.2), and the lethality of extreme weather events (indicator 1.2.3). Assessing the influence of environmental changes on ecological niches for human pathogens, the section also models the changing suitability for the transmission of climate-sensitive infectious diseases, expanding the analysis from previous years to include three diseases of global public health relevance (Zika, chikungunya, and *Vibrio cholerae*) and improving models from the 2020 report to reflect the reproduction number for arbovirus transmission. With health outcomes of vector-borne disease transmission being strongly influenced by socioeconomic factors and health-care access, indicator 1.3.2 incorporates considerations of implemented adaptation measures to assess the changing vulnerability of populations to arboviruses. Vector-borne disease transmission is followed by indicators of environmental pressure on terrestrial and marine food productivity. In this year’s report, the anlaysis has been extended to assess the association between heat stress and severe food insecurity (indicators 1.4.1 and 1.4.2). The final indicator in this section focuses on exposure to rising sea levels and its implications for human mobility (indicator 1.5).

### Indicator 1.1: health and heat

#### Indicator 1.1.1 vulnerability to the extremes of heat—headline finding: although vulnerability to heat in the low and medium HDI country groups is 27–38% lower than in the very high HDI group, it is increasing in all groups and, since 1990, it has increased by 19% in the low HDI group and by 20% in the medium HDI group

Exposure to extreme heat poses an acute health hazard, with individuals older than 65 years,^58–60^ populations in urban environments,^59,60^ and people with health conditions^58,59^ being particularly at risk. Heat disproportionately affects people who are marginalised or under-resourced that have little access to cooling mechanisms and health care, amplifying health and social inequities.^61–64^

This indicator tracks vulnerability to extreme heat through an index that combines the proportion of the population older than 65 years, the prevalence of relevant chronic diseases (respiratory disease, cardiovascular disease, and diabetes) in that group, and the proportion of the total population living in urban areas.

With aging populations, high prevalence of chronic diseases, and increasing urbanisation, the countries with a very high HDI had the highest vulnerability to extremes of heat. However, vulnerability to heat is rising across all HDI groups, with countries of low and medium HDI having the largest increases in vulnerability to heat since 1990 (19% for the low HDI group and 20% for the medium HDI group). The worsening trends in extreme temperature, as exposed in other indicators from this section, highlight a need to identify populations who are vulnerable to the health impacts of heat at the national and local levels. Additional work will be done to capture other heat vulnerabilities for this indicator.

#### Indicator 1.1.2 exposure of vulnerable populations to heatwaves—headline finding: children younger than 1 year were affected by 626 million more person-days of heatwave exposure and adults older than 65 years were affected by 3·1 billion more person-days of heatwave exposure in 2020 than in the 1986–2005 average

Young children and older people are especially susceptible to the health risks of high temperatures and heatwaves.^65^ This indicator reports the total number of days adults older than 65 years and (for the first time) children younger than 1 year were exposed to life-threatening heatwave events. In an improvement from previous years’ reports, the definition of a heatwave now aligns with the World Meteorological Organization (WMO) and other scientific literature.^66–68^ Additional details are given in appendix 5 (pp 6–8).

**Figure 1:**
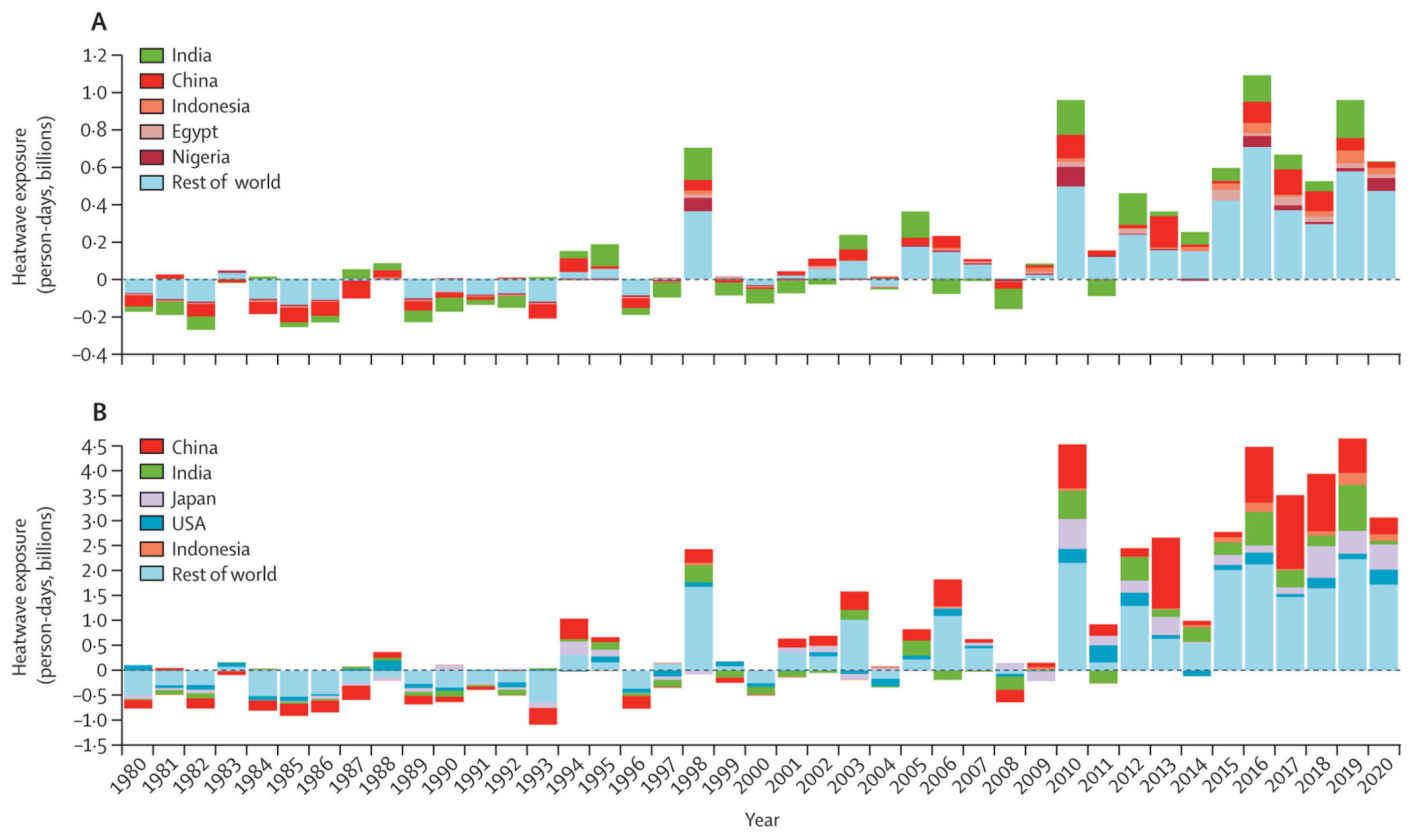
Change in person-days of heatwave exposure relative to the 1986–2005 baseline (A) People younger than 1 year. (B) People older than 65 years. The dotted line at 0 represents the baseline.

Results show a steady increase in the person-days of exposure for adults older than 65 years, with an annual average of 2·9 billion additional person-days of heatwave exposure in the past 10 years and 3·1 billion more (or an average of 4·1 days per person >65 years) in 2020, with respect to the 1986–2005 baseline average (figure 1). For children younger than 1 year, there were an estimated 626 million additional person-days of exposure (4·6 days per person <1 year) affecting this vulnerable group in 2020 compared with baseline years.

#### Indicator 1.1.3 heat and physical activity—headline finding: the past four decades saw an increase in the number of hours in which temperatures were too high for safe outdoor exercise, with people in the low HDI country group having an average loss of 3·7 h of safe exercise per day in 2020

Physical exercise provides mental health benefits and reduces the risk of cardiovascular disease, diabetes, cancer, cognitive decline, and all-cause mortality.^69–73^ However, high temperatures can reduce the frequency of physical activity, duration of physical activity, and the desire to engage in exercise,^74–76^ and even low amounts of physical activity in high temperatures can pose a risk to health.^77^ This indicator estimates the loss of potential hours of safe physical activity per person due to ambient temperature, humidity, and radiant heat, by tracking the hours per day that the wet bulb globe temperature exceeds 28°C, a threshold above which the national sports medicine authorities of the USA, Australia, and Japan recommend outdoor physical activities are done with discretion.^78,79^

**Figure 2:**
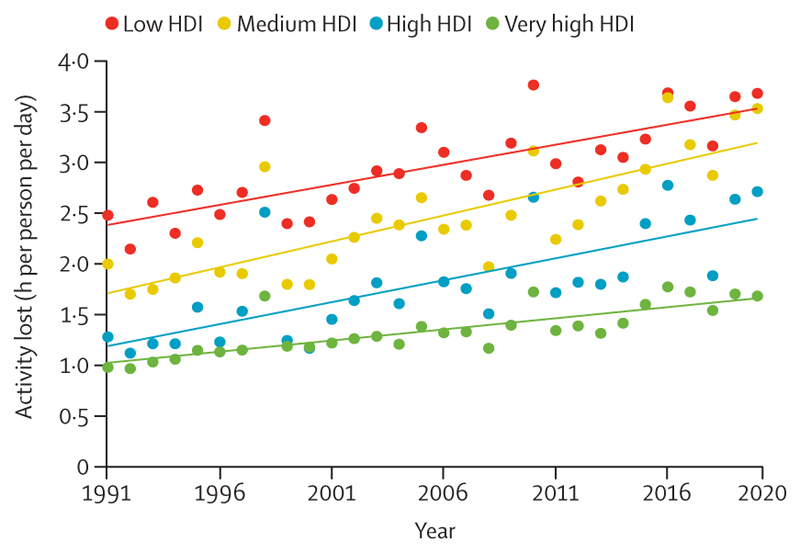
Average hours of safe physical activity lost per person due to high wet bulb globe temperatureby 2019 HDI country group (1980–2020) HDI=human development index.

Due to rising temperatures, the loss in the number of hours available for safe physical activity per day increased in all four country HDI groups (figure 2). The greatest loss of available time for safe physical activity occurred in the low HDI group, with an average increase from 2·5 h/person per day in 1991 to 3·7 h/person per day in 2020.

#### Indicator 1.1.4 change in labour capacity—headline finding: 295 billion h of potential work were lost due to extreme heat exposure in 2020, with 79% of all losses in countries with a low HDI occurring in the agricultural sector

In addition to direct impacts on health, high temperatures can also affect people’s ability to work.^80^ This indicator estimates the potential work hours lost as a result of heat exposure, by linking wet bulb globe temperature with the power (metabolic rate) typically expended by a worker. Data are broken down by labour sector, into construction, manufacturing, agriculture, and all other labour sectors (including the service sector).

**Figure 3:**
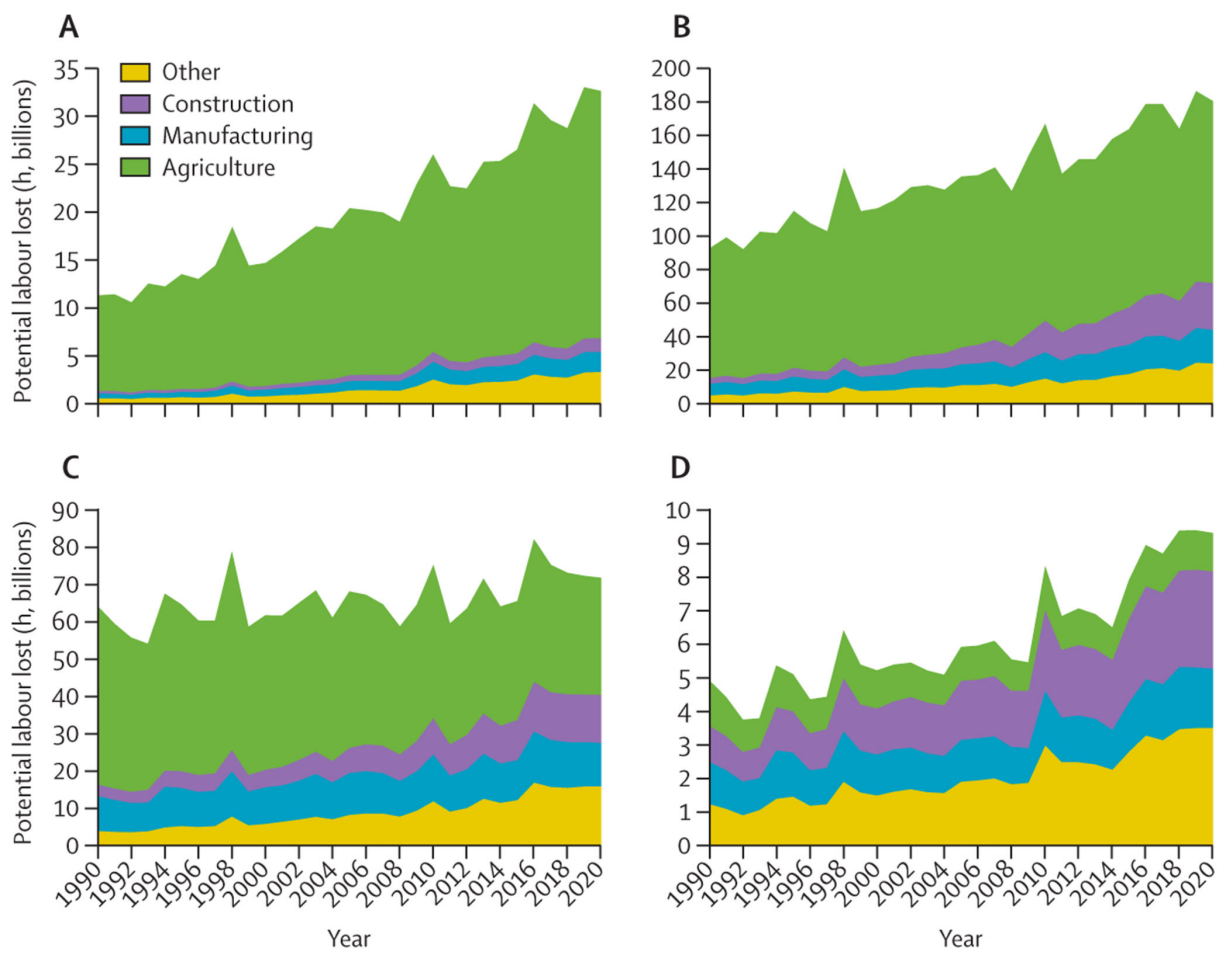
Potential labour lost due to heat-related factors in each sector (1990–2000) Low HDI (A), medium HDI (B), high HDI (C), and very high HDI (D) groups (2019 HDI country group). HDI=human development index.

In a rising trend since at least 1990, 295 billion h of potential work were lost across the globe in 2020 due to heat exposure—ie, the equivalent to 88 work h per employed person (figure 3). The three most populous countries in the medium HDI group (Pakistan, Bangladesh, and India) had the greatest losses among this group (2·5–3 times the world average and the equivalent to 216–261 h lost per employed person in 2020). 36 (77%) of 47 of the countries within the lowest quartile in terms of number of hours of potential labour lost per person belong to the very high HDI group. With lockdowns around the world, COVID-19 led to the loss of millions of hours of effective labour, particularly within service, construction, and manufacturing sectors.^81^ The changes in labour structure induced by COVID-19 are not accounted for by this indicator.

Almost half of the total potential work hours lost globally occurred in the agricultural sector of low and medium HDI countries. Occupational heat exposure disproportionately affects labourers in the agricultural sector of low HDI countries, with 25·8 billion h (79%) of 32·6 billion h of these countries’ losses occurring in this sector, compared with only 1·1 billion h (12%) of 9·3 billion h in very high HDI countries. The impact of heat exposure on working hours could therefore affect food production. Although heat affects labour capacity across all genders, differences in occupation might drive gender disparity. Men make up 80% of the total employment in the construction sector, and women in rural areas, and particularly indigenous women in rural areas, who are dependent on local natural resources for their livelihood would be particularly affected by the impacts of climate change on labour capacity.^82–84^

#### Indicator 1.1.5 heat and sentiment—headline finding: exposure to heatwave events worsens expressed sentiment, with a 155% increase in negative expressions on Twitter during heatwaves in 2020 from the 2015–19 average

Increases in heat extremes that are related to climate change pose diverse risks to mental health globally, ranging from altered affective states to increased mental health-related hospital admissions and suicidality.^54–56,85–88^ However, because the definition, acknowledgment, stigmatisation, and treatment of mental health varies across different regions and cultures,^57^ assessing the mental health effects of climate change is a challenge that the *Lancet* Countdown will work to address in upcoming years.

This indicator, which is new to the 2021 report, tracks the effect of heatwaves on the general sentiment of expressions from Twitter users around the world with previously published methods for estimating climate impacts.^89–91^ This indicator classifies the sentiment expressed in more than 6 billion geolocated tweets collected between 2015 and 2020, using the linguistic inquiry word count sentiment classification tool.^92^ A multivariate ordinary least squares fixed effects model is then used to estimate the annual effect of heatwaves on expressed sentiment. Using this method, this indicator compares sentiment expression during heatwave days (as defined in indicator 1.1.2) with non-heatwave days in 40 000 unique geographical localities for nearly 1 million individuals per day. Potential temporal and geographical confounders were adjusted for by considering the month, calendar date, and location of each tweet in the analysis. Additional detail is provided in appendix 5 (pp 16–19). This indicator offers a glimpse into the influence of heat extremes on the sentiment of people around the world. However, since Twitter access and social media use are not evenly distributed, countries with a higher income are disproportionately represented.

**Figure 4:**
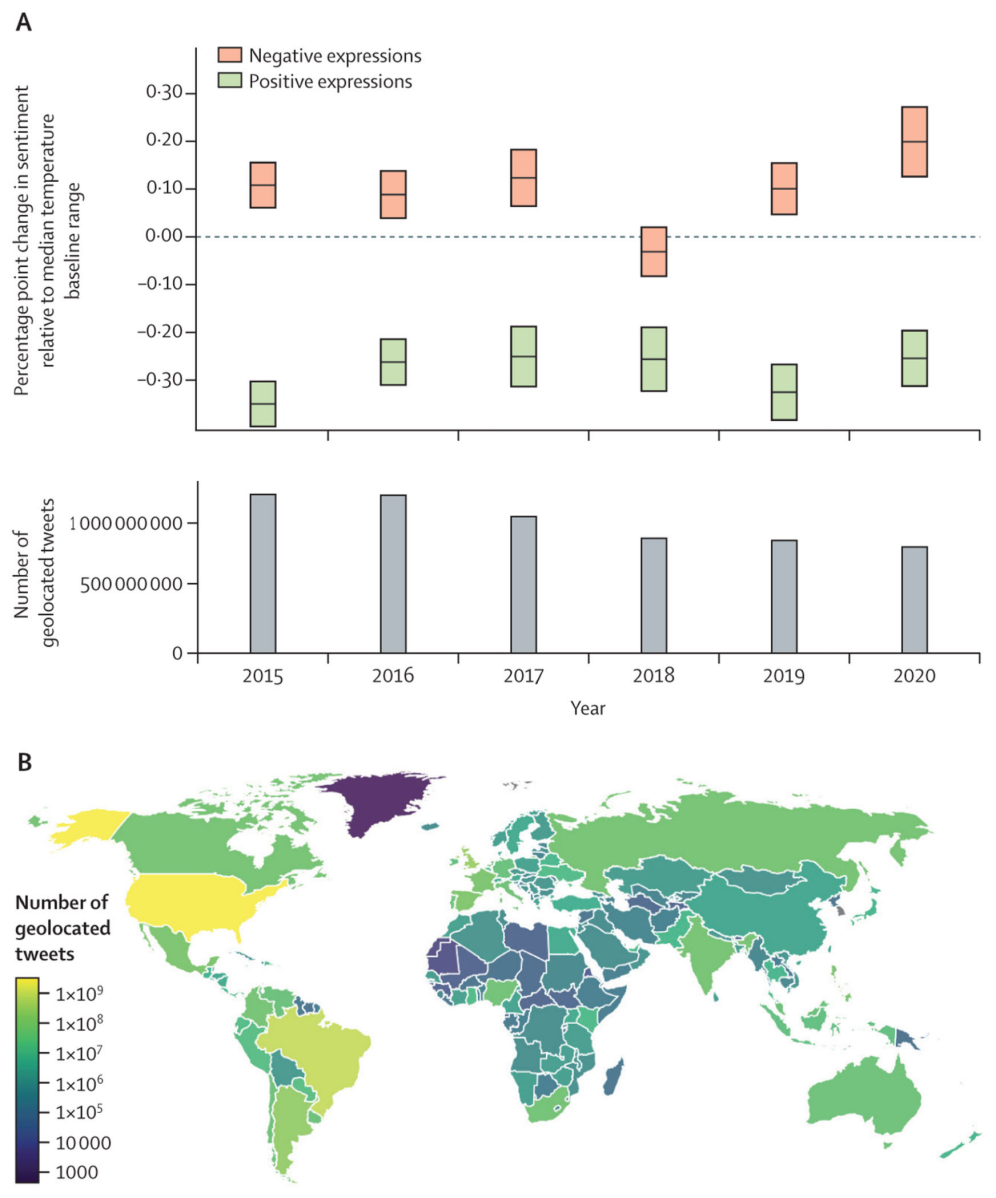
Heatwaves and sentiment on Twitter (A) Annual effect of heatwave exposure on the sentiment of Twitter users expressions from 2015–20. Boxes depict 95% CIs of the estimated average change in general sentiment expressions during days with heatwaves, relative to the median daily maximum temperature baseline range for each location and year. Sentiment was extracted from Twitter posts using a dictionary-based approach across multiple languages, see appendix 5 (p 16). Grey bars depict the geolocated Tweet count by year of observation. (A) Country-level count of total geolocated tweets for 2015–20.

Local heatwave exposure was found to significantly reduce positive expressions and increase negative expressions (figure 4). In 2020, the percentage point change in negative sentiment during a heatwave day was 0·20 (95% CI 0·31–0·08); 155% higher than the 2015–19 average increase. Compared with the 2015–19 baseline average, the magnitude of this increase was substantial, equivalent to three-quarters of the total rise in negative sentiment observed during a benchmark flooding event (appendix 5 p 19). The reduction in positive sentiment observed during heatwaves in 2020 was 11·9% less than that observed during heatwaves in 2015–19.

#### Indicator 1.1.6 heat-related mortality—headline finding: heat-related deaths in people older than 65 years reached a record high of an estimated 345000 deaths in 2019; between 2018 and 2019, all WHO regions, except for Europe, saw an increase in heat-related deaths in this vulnerable age group

Exposure to extreme heat increases the risk of death from cardiovascular, cerebrovascular, and respiratory conditions and all-cause mortality.^93^ As in the 2020 report, this indicator uses the exposure-response function and minimum mortality temperature defined by Honda and colleagues^94^ to estimate deaths attributable to extremes of heat, with work ongoing to increase the accuracy of local estimates.^95^ Using life expectancy data from the 2019 Global Burden of Diseases, Injuries, and Risk Factors Study,^96^ years of life lost (YLL) were also calculated to better reflect health burdens.

**Figure 5:**
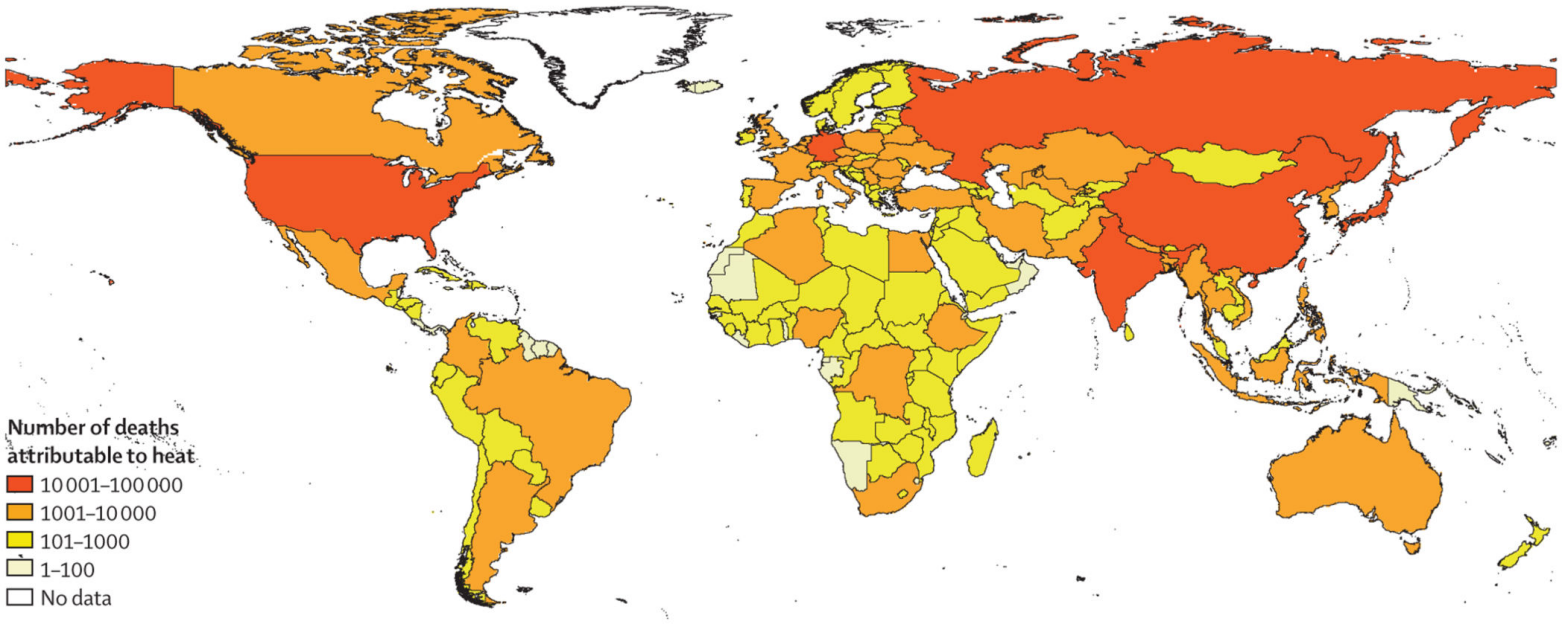
Heat-related deaths of people older than 65 years in each country in 2019

Heat-related mortality for people older than 65 years increased throughout the study, reaching a record high of almost 345 000 deaths in 2019 (figure 5)—80·6% higher than in the 2000–05 average. Between 2018 and 2019, India and Brazil had the biggest absolute increase in heat-related mortality. Although heat-related mortality decreased between 2018 and 2019 in the WHO European region (due to fewer attributable deaths in countries such as Germany, Russia, and the UK), this region is still the most affected, with almost 108 000 deaths attributable to heat exposure in 2019.

### Indicator 1.2: health and extreme weather events

#### Indicator 1.2.1: wildfires—headline finding: nearly 60% of countries had an increase in the number of days people were exposed to very high or extremely high fire danger in 2017–20 compared with 2001–04, and 72% of countries had increased human exposure to wildfires across the same period

Hotter and drier conditions caused by climate change increase the risk of wildfires and the extent of their damage.^97^ As in previous years, this indicator tracks wildfire exposure by combining satellite-observed active fire spots^98,99^ and human exposure to high and extremely high wildfire danger (considering a fire weather index score of worse than 5 and population data).^100^ The fire weather index, provided by the Copernicus Emergency Management Service for the European Forest Fire Information System,^101^ combines air temperature, relative humidity, wind speed, and drought effects to capture the chances of a fire starting, its rate of spread, its intensity, and its difficulty of suppression. A full description of the methods used can be found in appendix 5 (pp 23–24). This indicator does not yet quantify exposure to wildfire smoke, which can affect much larger populations and have larger health consequences than direct exposure to the fire; it is estimated that smoke from the 2019–20 Australian fires affected 80% of Australia’s population and resulted in hundreds of deaths and thousands of people admitted to hospital.^102^

**Figure 6:**
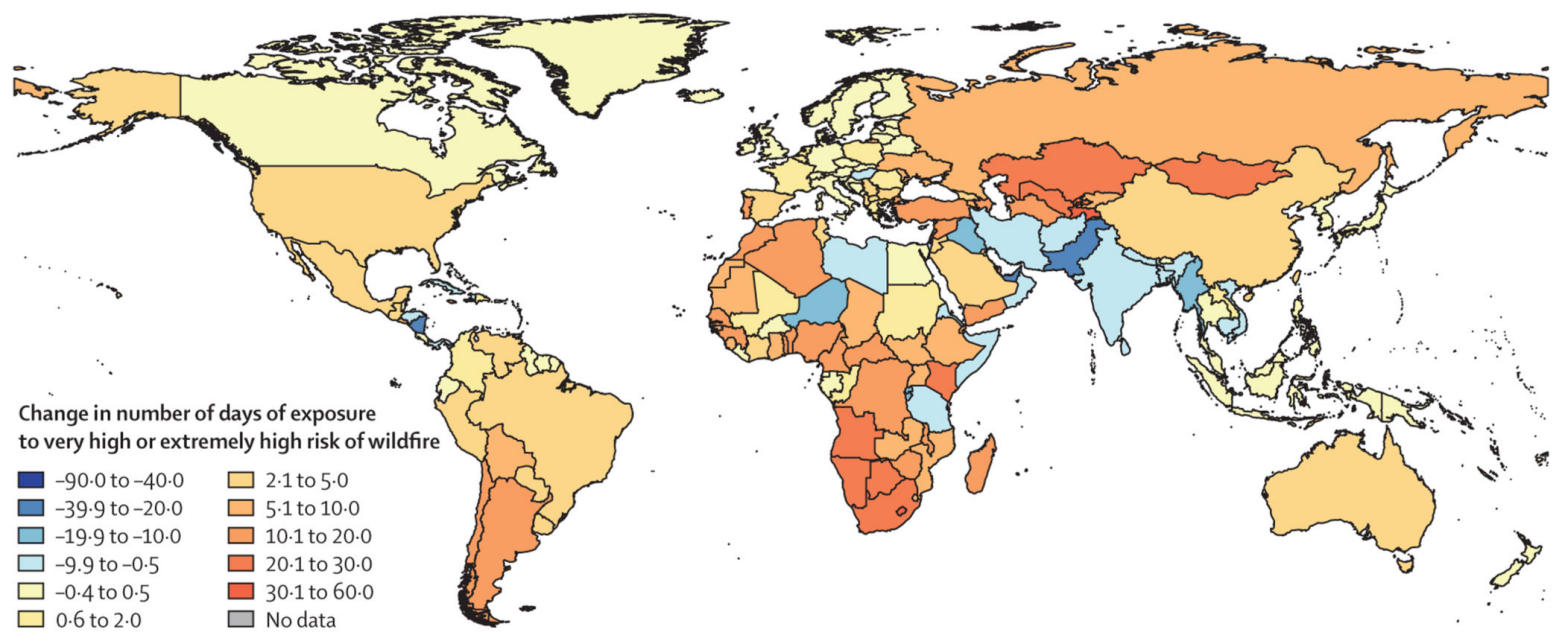
Annual population-weighted mean change in the number of days with very high and extremely high risk of wildfire from 2001–04 to 2017–20 for each country or territory Large urban areas with a population density ≥400 persons/km^2^ are excluded in the calculations of population-weighted mean values. Very high and extremely high risk is defined by the fire weather index.^101^

Globally, in 2017–20, there was an average of 215 531 more person-days of wildfire exposure than in 2001–04. Overall, 134 (72·4%) of 185 countries had an increase in wildfire exposure in 2017–20 compared with 2001–04. But this increase was unequal—27 (83%) of 32 low HDI countries had an increase in wildfire exposure compared with 40 (62·5%) of 64 very high HDI countries. The largest increases in wildfire exposure were observed in the Democratic Republic of the Congo, India, and China. During the same time period, the climatological danger of wildfire increased in 110 countries, with the largest growth occurring in Lebanon, The Gambia, and Lesotho (figure 6).

#### Indicator 1.2.2: drought—headline finding: in 2020, up to 19% of the global land surface was affected by extreme drought in any given month

Climate change is increasing the frequency, intensity, and duration of drought events. These changes pose threats to water security, sanitation, and food productivity and increase the risk of wildfires and exposure of the environment to pollutants.^52,103^

This indicator tracks the land area affected by extreme drought events using the standardised precipitation-evapotranspiration index (extreme drought ≤1·6 and exceptional drought ≤2, in alignment with the Federal Office of Meteorology and Climatology MeteoSwiss^104^), capturing the changes in precipitation and the effect of temperature on evaporation and moisture loss. More details about this indicator are provided in appendix 5 (pp 25–27).

**Figure 7:**
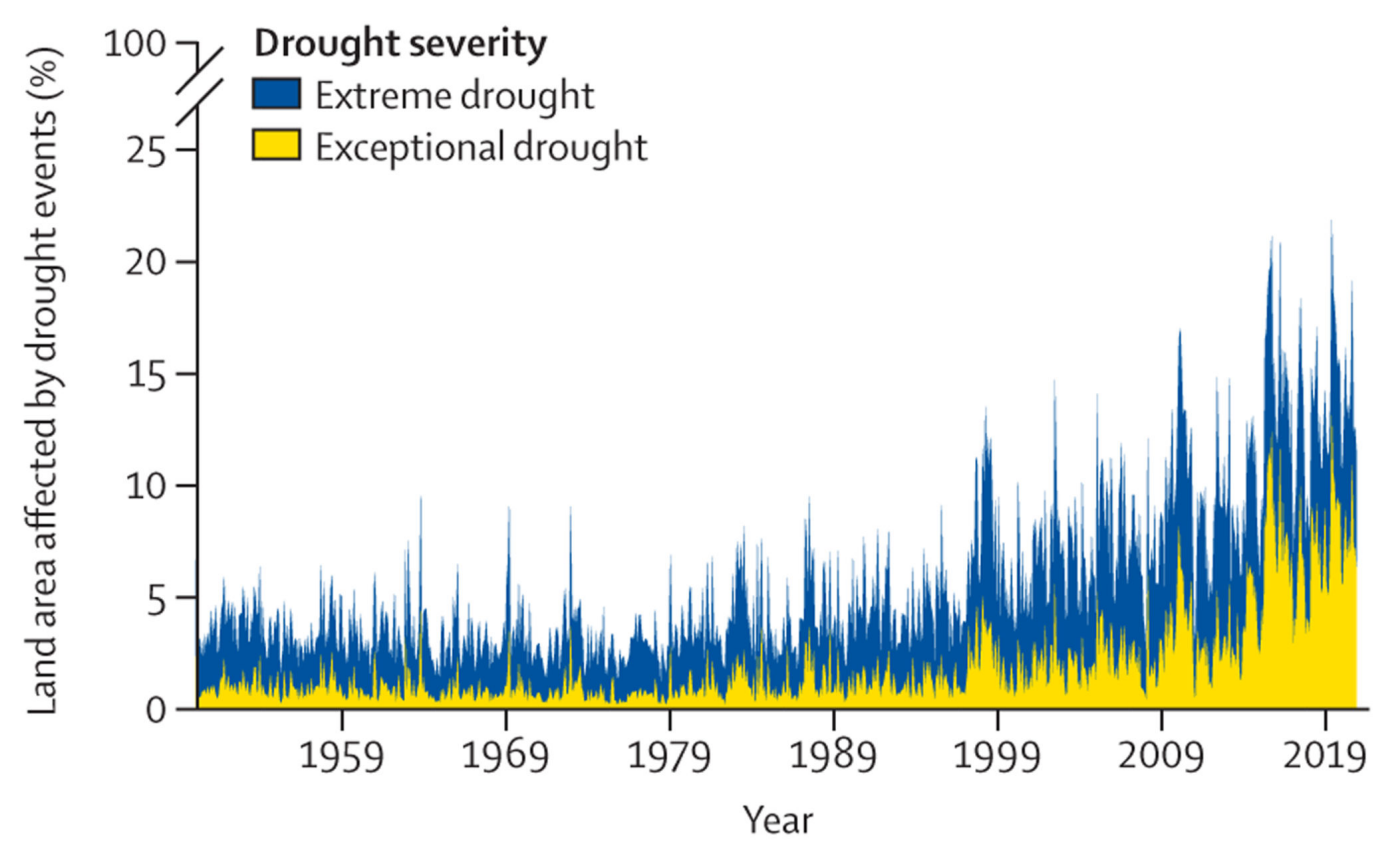
Global land area affected by drought events per month Extreme drought is defined by a SPEI of ≤1·6 and exceptional drought is defined by a SPEI of ≤2. SPEI=standardised precipitation-evapotranspiration index.

The global land surface area affected by extreme drought conditions has consistently increased since 1990. The proportion of the world’s land surface with extreme drought in any given month reached a maximum of 22% in 2010–19; a value that had only reached 13% in 1950–99 (figure 7). Furthermore, the 5 years with the most area affected by extreme drought have all occurred since 2015, and the Horn of Africa, a region impacted by recurrent extreme droughts and food insecurity,^105^ was one of the most affected areas in 2020.

#### Indicator 1.2.3: lethality of extreme weather events—headline finding: the past 30 years have seen statistically significant increases in the number of extreme weather events; however, only the low HDI group had a statistically significant increase in the number of people affected by these events

This indicator tracks the number of occurrences of weather-related disasters that are climate sensitive, and the number of people affected or killed per event. Data are taken from the Centre for Research on the Epidemiology of Disasters and have been presented as standard anomalies across the 1990–2020 period. All HDI country groups have had a consistent and statistically significant increase in the number of extreme weather events during the past 30 years, with the very high HDI group having the highest increase (appendix 5 pp 28–32). However, only the low HDI group has had a statistically significant increase of people affected per disaster event—a situation that might reflect a more rapid growth in the populations living in high-risk areas within low HDI countries or inequities between HDI groups in adaptive capacity and preparedness to respond to worsening climate change hazards.

### Indicator 1.3: climate-sensitive infectious diseases

#### Indicator 1.3.1 climate suitability for infectious disease transmission—headline finding: in 2011–21, the area of coastline suitable for Vibrio bacterial transmission has increased by 35% in the Baltics, 25% in the Atlantic Northeast, and 4% in the Pacific Northwest; the number of months suitable for malaria transmission increased by 39% between 1950–59 and 2010–19 in highland areas of the low HDI group

Climate change is affecting the distribution of arthropodborne, food-borne, and water-borne diseases.^46,47^ Together with global mobility and urbanisation, climate change is a major driver of the increase in the number of dengue virus infections,^106^ which have doubled every decade since 1990.^96^ Other important emerging or re-emerging arboviruses, transmitted by mosquitoes, are likely to have a similar response to climate change.^107^ This indicator tracks the environmental suitability for the transmission of arboviruses (dengue, chikungunya, and Zika) with an improved model to assess the influence of temperature and rainfall on vectorial capacity and vector abundance, and overlays it with human population density data to estimate the reproductive number (R_0_; the expected number of secondary infections resulting from one infection). The R_0_ for all arboviral diseases tracked has increased with respect to the 1950–54 average, and, in 2020, was 13% higher for transmission by *A aegypti* and 7% higher for transmission by *A albopictus* than in baseline years (1950–54). The largest increases in epidemic potential for dengue, Zika, and chikungunya were in countries with very high HDI, mainly from the ongoing geographical expansion of *Aedes* mosquitoes.

The influence of the changing climate on the length of the transmission season for *Plasmodium falciparum* malaria was also tracked with a threshold-based model that incorporates precipitation accumulation, average temperature, and relative humidity.^12^ There were substantial differences in the number of months suitable for transmission of malaria in highland areas (ie, areas ≥1500 m above sea level) in 2010–19 compared with in 1950–59, with a 39% increase in the low HDI country group and a 15% increase in the medium country HDI group. The difference between high and medium HDI areas is even more marked at a subnational level than at a national level, which suggests that climate change might make malaria eradication efforts increasingly difficult in already disadvantaged areas.

**Figure 8:**
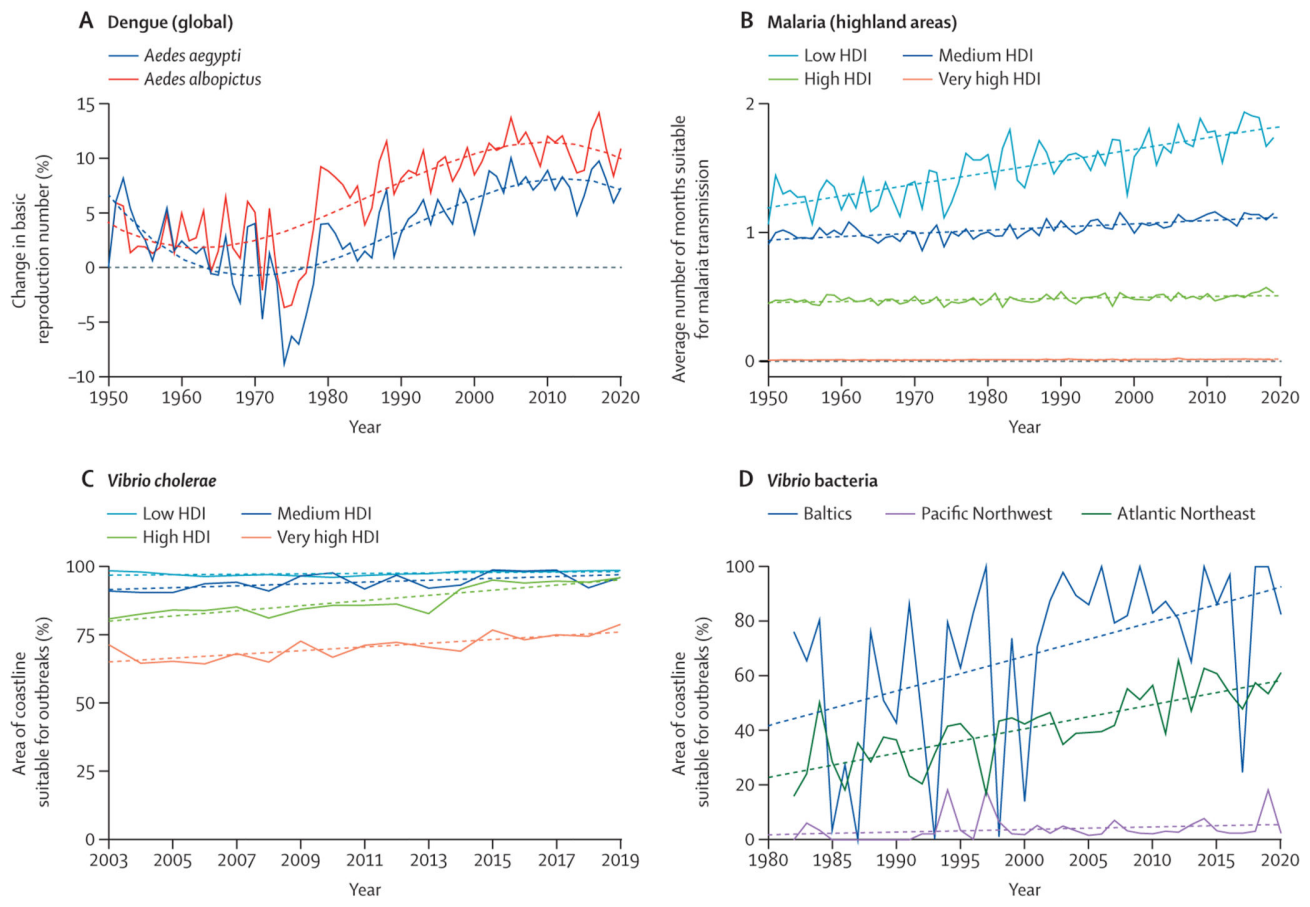
Change in climate suitability for infectious diseases Solid lines represent the annual change. Dashed lines represent the trend since 1950 (for dengue and malaria), 1982 (for *Vibrio* bacteria), and 2003 (for *Vibrio cholerae*). HDI=human development index.

This indicator also monitors the environmental suitability for the transmission of *Vibrio* bacteria in coastal waters. *Vibrio* pathogens can cause gastroenteritis, life-threatening cholera, severe wound infections, and sepsis.^14^ Driven by changes in sea surface temperature and sea surface salinity, the area of coastline showing suitable conditions for the transmission of non-*cholerae Vibrio* species at any one point during the year increased by 56% (from 7·0% to 10·9% of the coastline) in latitudes of the northern hemisphere (40–70° north) in 2020 compared with the 1982–89 baseline. From 1982–89 to 2011–20, the area of coastline suitable for non-*cholerae Vibrio* species at any point during the year has risen from 47·5% to 82·4% in the Baltics, 29·9% to 54·9% in the Atlantic Northeast, and 1·2% to 5·1% in the Pacific Northwest (figure 8). Between 2003 and 2019, there was an increase in the proportion of coastline with suitable conditions for *V cholerae* across all HDI country groups, with the low HDI country group having the highest suitability for *V cholerae* on average (at 98·6% of countries’ coastlines in 2019). However, the high HDI country group had the greatest increase in suitable coastline area during this period, at a rate of almost an additional 1% of their coastline area becoming suitable each year (coefficient of determination=0·78; df=15; p<0·01).

#### Indicator 1.3.2: vulnerability to mosquito-borne diseases—headline finding: although vulnerability to arboviruses transmitted by A albopictus and A aegypti has decreased across all countries since 2000, people in countries in the low HDI group are still the most vulnerability on average

As shown by indicator 1.3.1, climate change is making environmental conditions increasingly favourable for the transmission of some arboviruses. Although interventions to reduce the vulnerability of people to infection can partly counteract the increase in risk of transmission, environmental pressures make these interventions increasingly challenging. This indicator combines the environmental suitability for the transmission of dengue (as described in indicator 1.3.1) with indicators of social vulnerability to this disease—ie, access to sanitation and water services, income level, and health-care quality.^108,109^

Due to improvements in sanitation, income, and health-care quality, vulnerability to mosquito-borne diseases is decreasing, even despite increases in their environmental suitability. Although the vulnerability of countries in the low HDI group to disease transmission by *A aegypti* has decreased by 34% between 2000 and 2017, the same time period has had a 61% decrease in vulnerability to disease transmission by *A aegypti* in the very high HDI country group and a 73% decrease in the high HDI country group. The vulnerability index is inversely related to the level of HDI, with countries in the low HDI group having a vulnerability index of more than 360 times higher than countries in the very high HDI group in 2017 (appendix 5 pp 46–47).

### Indicator 1.4: food security and undernutrition

#### Indicator 1.4.1: terrestrial food security and undernutrition—headline finding: crop yield potential continues to follow a downward trend, with 6·0% reduction in the crop yield potential of maize, 3·0% for winter wheat, 5·4% for soybean, and 1·8% for rice, relative to the 1981–2010 average crop yield potential

Food insecurity is increasing and has affected 2 billion people in 2019.^110^ Climate change threatens to exacerbate this crisis, which will disproportionately affect people who are the most vulnerable and those already facing undernutrition. Due to socially defined gender roles and less empowerment than men, food insecurity disproportionately affects rural women, reinforcing their disadvantaged position through reduced educational attainment, income, and socioeconomic status.^111^

**Figure 9:**
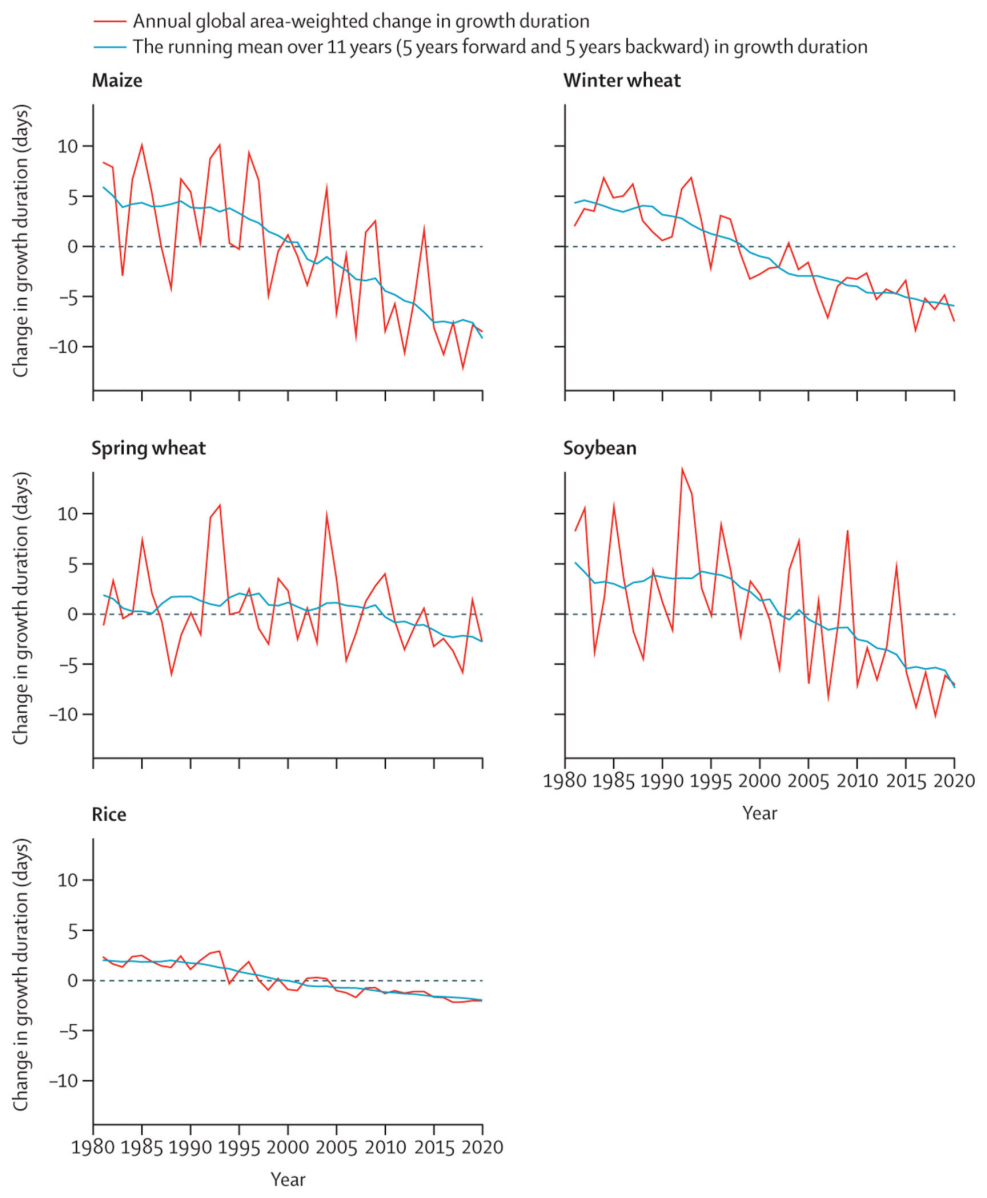
Change in crop growth duration relative to the 1981–2010 global average The red line represents the annual global area-weighted change in crop growth duration. The blue line represents the running mean of change in crop growth duration over 11 years (5 years before and 5 years after).

This indicator tracks the change in crop yield potential resulting from rising temperatures with the same methods as for the 2020 report,^53^ in which crop yield potential is the yield that could be obtained with no limitations on water or nutrients or extreme events. Rising temperatures shorten the time taken for crops to reach maturity (ie, reduced crop growth duration), thereby leading to reduced seed yield potential.^112^ Therefore, a reduction in crop growth duration can be considered an indicator of future crop yield reductions due to higher growing season temperatures (and therefore a shortened growing season), in the absence of adaptation. Crop yield potential continues to follow a consistent downward trend, adding additional pressure to already strained food systems around the world. Reductions in time to maturity are observed in all staple crops tracked, amounting to a 6·0% reduction for maize, 3·0% for winter wheat, 5·4% for soybean, and 1·8% for rice yield relative to the average crop yield potential in 1981–2010 (figure 9).

Data from the Food Insecurity Experience Scale of the UNs’ Food and Agriculture Organization (FAO)^113^ was used to assess self-reported experiences of severe food insecurity (defined as a situation in which an individual went at least one day without eating as a result of scarcity of resources in the past 12 months) in 83 countries. A fixed-effects, time-varying regression showed that every 1°C of temperature increase was associated with a global increase of 1·4% in the probability of severe food insecurity (95% CI 1·3–1·47; p<0·001) in 2014 and 1·64% (1·6–1·65; <0·001) in 2019.

#### Indicator 1.4.2: marine food security—headline finding: in 2018–20, nearly 70% of countries showed increases in average sea surface temperature in their territorial waters compared with in 2003–05, reflecting an increasing threat to their marine food productivity and marine food security

Per-capita fish consumption has increased steadily since the 1960s.^114^ About 3·3 billion people depend on marine food, with coastal populations in low and medium HDI countries, SIDS, and indigenous people in particular relying on it for their nutrition and livelihoods.^114,115^ Climate change is resulting in changes in marine fish capacity and capture through increases in sea water temperatures (and the associated reduced oxygenation), ocean acidification, and coral reef bleaching. As a result of these changes, coastal tropical countries are the most at risk from reduction in marine crop yield potential, and are also the most vulnerable to the associated socioeconomic impacts.^115–117^

This indicator expands its geographical scope for 2021, tracking sea surface temperature in territorial waters of 136 countries to reflect the changing threats of climate change on marine productivity and, therefore, on marine food security. The indicator is complemented by the reported changes in marine capture based-per-capita fish consumption, using data collected by the FAO (appendix 5 pp 51–71).

Average sea surface temperature increased in the territorial waters of 95 (70%) of 136 studied countries in 2018–20 compared with 2003–05, posing threats to marine food productivity. Marine capture-based fish consumption has also reduced since 1988, coupled with an increase in the consumption of farm-based fish products of lower nutritional quality and omega-3 content.^118^ These trends expose the threats that climate change poses to marine food security around the world.

### Indicator 1.5: migration, displacement, and rising sea levels

*Headline finding: there are currently 569·6 million people settled lower than 5 m above sea level who could face risks from the direct and indirect hazards posed by the rising sea levels*

Between 1902 and 2015, the global mean sea level increased by 0·12–0·21 m.^119^ If unabated, sea level rise is projected to reach up to 2 m above current levels within 80 years, or even higher in some locations if considering ice sheet collapse, waves, tidal contributions, and other factors.^120–123^ This indicator tracks size of the population settled in areas at risk of global mean sea level rise, based on coastal elevation and population distribution,^124,125^ and the national policies connecting climate change, human mobility, and health.

There are currently 146·6 million people living in coastal areas less than 1 m above current sea levels, 27·3% of whom reside in areas with low HDI levels. Furthermore, as sea levels continue to rise, the 569·6 million people settled in areas less than 5 m above current sea levels could face increased risks of flooding, more intense storms, soil and water salinification,^126^ and local emergence of infectious diseases;^127^ 26·6% of these people live in areas with low HDI levels. Where erosion occurs, dwellings and other infrastructure can be damaged.

Migration and mobility could be a response to increased sea levels, and also increase in response to other impacts of climate change. Increased migration and mobility would affect livelihoods, access to essential services, and psychosocial wellbeing.^128–130^ As of Dec 31, 2020, 45 policies connecting climate change and migration were identified in 37 countries (appendix 5 pp 72–78), all of which mentioned health or wellbeing, but this mention was typically related to climate change effects rather than to the potential health effects of forced migration. Although these policies often accepted that mobility could be domestic and international, immobility was rarely acknowledged. National policies that recognise and respond to the health risks and health benefits of different mobility patterns will partly shape the overall health outcomes.^131^

### Conclusion

In this sixth iteration of the *Lancet* Countdown indicators, section 1 of the 2021 report highlights a continuous increase in the impacts of climate change on all monitored aspects of human health, providing additional evidence that climate change is having quantifiable and increasingly negative impacts on human health.

Although its health impacts are felt across the world, climate change disproportionately affects disadvantaged populations, exacerbating their vulnerabilities. The stratification of indicators by HDI groups reveals the higher risks faced by low and medium HDI countries, particularly with regards to labour capacity and livelihoods, food security, and vector-borne disease transmission. Reporting the health impacts on disadvantaged groups and the necessary adaptation responses (described in section 2) represents a major challenge, made greater by the absence of disaggregated data.^15^ With respect to gender, these challenges are explored in panel 2. Moreover, although section 1 considers the impact of heat on online sentiment expression, the difficulties of capturing the mental health effects of climate change have not been addressed. The *Lancet* Countdown will continue to focus on closing this gap.

## Section 2: adaptation, planning, and resilience for health

The past year has affirmed the centrality of health and wellbeing to socioeconomic development, illustrating how health risks can compound and cascade across sectors and nations and highlighting the potential consequences of scarce investments into health systems that are climate resilient and environmentally sustainable.^132,133^ The COVID-19 pandemic has also exposed stark differences in the capacity of health systems and the resilience of populations to health emergencies,^134,135^ highlighting the urgent need for health authorities to increase national and international coordination and preparedness. This coordination should include integrated surveillance and monitoring of emerging health threats, developing and deploying early warning and response systems, and financially supporting low-resource nations and communities.^136^ To be effective, public health responses must address the needs of the most vulnerable, reducing inequities and therefore benefiting the whole society.

Building health systems that are climate resilient and environmentally sustainable would not only help reduce the health impacts of climate change explored in section 1, but also contribute to minimising the risk of future pandemics. This section reports eight indicators of adaptation, planning, and resilience, which are closely linked with the components of the WHO Operational Framework for Building Climate Resilient Health Systems: planning and assessment (indicators 2.1.1–2.1.3); information systems (indicator 2.2); delivery and implementation (indicators 2.3.1–2.3.3); and funding and spending (indicator 2.4). Each of these indicators provide insights into inequities. Data on health adaptation funding from global financing mechanisms, which are necessary to help countries with a low or medium HDI to adapt to the worsening health impacts of climate change, have been reintroduced into this year’s report (indicator 2.4).

An unaddressed challenge in section 2 is the scarcity of clear metrics to monitor adaptation progress. Although efforts were made to validate the indicators, self-reported data for adaptation plans, assessments, and services might be have reporting bias, particularly where COVID-19 resulted in the redeployment of public health resources and where surveys had a decline in participation.

### Indicator 2.1: adaptation planning and assessment

#### Indicator 2.1.1: national adaptation plans for health—headline finding: in 2021, 47 (52%) of 91 countries reported having national health and climate change strategies or plans in place

Health systems are under pressure to respond to the acute and long-term threats from climate change and other, simultaneous, public health risks. Comprehensive, implemented health adaptation plans can not only improve health resilience of populations to climate change but also contribute to a broader strengthening of health systems and catalyse effective collaboration with other health-determining sectors.

Data for indicators 2.1.1 and 2.1.2 are from the 2021 WHO Health and Climate Change Global Survey,^137^ which provides self-reported data on health sector response to climate change from 91 governments and is described in appendix 5 (pp 79–80). This indicator tracks the development of national health and climate change strategies and the barriers to implementation.

In the 2021 WHO Health and Climate Change Global Survey, 47 (52%) of 91 countries reported that they have a national health and climate change strategy or plan in place, which is comparable to the proportion reported in 2018 by the WHO survey. Implementation is still a challenge for countries from all HDI levels, with less than a quarter of countries who responded to the survey reaching high or very high levels of implementation. Insufficient financing was identified as a main barrier to reaching full implementation by 31 (69%) of all 45 responding countries, with 10 (25%) reporting that they have no current sources of funding available for the priorities set out in their strategies and plans. Other barriers to implementation were insufficient human resource capacity (expressed by 24 [53%] of 45 countries), COVID-19 related constraints (23 [51%]), and insufficient research, technologies, or tools (20 [44%]).

A desktop review of National Adaptation Plans (NAPs) submitted to the UNFCCC found that four of the 19 NAPs considered gender in health adaptation actions. However, although NAPs might mention the principles of gender equality, they often did not demonstrate they were integrating gender issues in a way that challenges gender norms, power, and structures. The recommendations in the WHO guidance, *Mainstreaming gender in health adaptation to climate change programmes*, provide countries with guidance for achieving gender mainstreaming, including through national health and climate change plans.^138,139^

#### Indicator 2.1.2: national assessments of climate change impacts, vulnerability, and adaptation for health—headline finding: 45 (49%) of 91 countries in 2021 reported having done a climate change and health vulnerability and adaptation assessment

Evidence-based policy development and planning require a comprehensive evaluation of the climate change-associated health risks faced by populations and health systems. This indicator monitors the number of countries that report having done a climate change, health vulnerability, and adaptation assessment. These assessments are crucial as they not only allow countries to establish and re-evaluate health risks but also consider the vulnerabilities to climate hazards that contribute to health outcomes.

Although 45 (49%) of 91 countries disclosed they had done a climate change and health vulnerability and adaptation assessment, only 8 (19%) of these countries reported that the findings strongly influenced the allocation of human and financial resources. In comparison, 17 (56%) of 43 countries reported that the findings strongly informed the development of health policies and programmes. Most countries specifically considered population groups vulnerable to the effects of climate change in their assessments, including children, women, older adults, workers, rural and urban populations, people living in poverty, and, to a lesser extent, indigenous groups, migrant populations, or displaced populations. However, the comprehensiveness of these assessments varied.

As explored in section 1, health vulnerabilities to climate change are unevenly distributed and can exacerbate existing health inequities. As health vulnerability and adaptation assessments inform national health and climate change plans and programmes, data gathered for these assessments must be disaggregated according to social determinants of health. This disaggregation will enable public health interventions to actively identify and support the populations most vulnerable to the effects of climate change and proactively reduce subnational health inequities relating to climate change.

#### Indicator 2.1.3: city-level climate change risk assessments —headline finding: in 2020, 546 (81%) of 670 cities reported having completed or being in the process of doing climate change risk assessments; heat-related illness was the most common climate-related health concern, identified by 169 (55%) of 308 cities

The COVID-19 pandemic revealed the persistent health inequities and vulnerabilities of cities and urban sub-populations to health emergencies.^140,141^ Home to more than half the world’s population (a proportion projected to increase to 70% by 2050), cities have a crucial role in leading the local health adaptation to climate change.^142^ With data from the Carbon Disclosure Project’s 2020 survey of global cities, this indicator shows the number of cities that report having completed a climate change risk or vulnerability assessment and the climate-related health impacts and vulnerabilities of these cities.

In 2020, 546 (81%) of 670 cities that responded to this questionnaire reported that they had completed, or were currently doing, climate change risk assessments. For those cities that responded in both 2019 and 2020, an additional 45 (9%) of 491 reported having completed a climate change risk assessment in 2020. However, 618 (94%) of 654 cites responding to this particular question belonged to countries with a high or very high HDI, meaning that cities and countries with low and medium levels of HDI were under-represented in these data. 308 (62%) of 495 cities responded positively to the question on whether their city faces risks to public health or health systems associated with climate change. The most prominent perceived health concern pertained to heat-related illness, with 169 (55%) of 308 responding cities reporting this concern. The populations identified as most vulnerable to climate change were so-called elderly adults (reported by 213 [69%] cities), so-called children and youth (180 [58%]), and people in low-income households (170 [55%]), and 94 cities (31%) identified women as vulnerable to climate-related health impacts.

#### Indicator 2.2: climate information services for health

*Headline finding: in 2020, national meteorological and hydrological services of 86 countries reported providing climate information to the health sector; only five of the 86 indicated that these climate services guide health sector policy and investment plans*

Health adaptation to climate change relies on accurate meteorological data and forecasts for the integrated surveillance and monitoring of emerging health threats, the development and deployment of early warning and response systems, and the implementation of adaptation interventions. This indicator monitors the extent to which national health and meteorological services provide climate information services to the health sector with data reported to the WMO.

In 2020, 86 national meteorological and hydrological services reported providing climate services to the health sector. Within the very high HDI group, 50% of countries that reported providing climate services to the health sector also reported that they were codesigning or providing tailored climate information services or products, compared with 36% of low HDI countries.

### Indicator 2.3: adaptation delivery and implementation

#### Indicator 2.3.1: detection, preparedness, and response to health emergencies—headline finding: 124 (75%) of 166 countries reported medium-to-high implementation of a national health emergency framework in 2020; an increase of 14% since 2019

The International Health Regulations (IHR) are legally binding instruments that define countries’ rights and obligations in handling public health events and mergencies that could cross national borders.^46^ Under the IHR, IHR state parties are required to provide self-evaluations of emergency response preparedness against 13 core capacities published in the State Party Annual Report (SPAR). Limitations of the IHR in ensuring an effective response to the COVID-19 pandemic have been identified and these limitations continue to be evaluated,^143^ as discussed in appendix 5 (pp 89–90). However, countries with higher SPAR scores had lower incidence of COVID-19 and mortality per 100 000 population within 30 days of the first COVID-19 diagnosis, stressing the relevance of the IHR.^144^

This indicator tracks the degree to which countries have implemented a national health emergency framework under IHR core capacity 8, which include emergency preparedness and response planning, emergency management structures, and mobilisation of resources. IHR core capacity 8 assesses whether countries are prepared to respond to all public health events, including climate-related emergencies. In 2020, 166 (85%) of 196 IHR state parties completed the section of the SPAR that related to core capacity 8, and 124 (75%) of 166 state parties reported medium-to-high degrees of implementation of a national health emergency framework (a 14% increase since 2019). However, only 62 (37%) of the 166 state parties reported high levels of implementation, indicated by a capacity score of 75% or greater. The level of implementation varied greatly by HDI group, with 89% of very high HDI countries reporting medium-to-high implementation compared with 55% of low HDI countries.

To prepare for future health crises, it is essential that global institutions improve emergency response preparedness using the lessons learned during the COVID-19 pandemic. The ongoing review of the IHR is an important step in this direction to ensure that the IHR is effective when faced with health emergencies associated with climate change.

#### Indicator 2.3.2: air conditioning: benefits and harms—headline finding: use of air conditioning, a widespread technology for indoor cooling in some regions of the world, averted an estimated 195 000 heat-related deaths among people aged 65 years or older in 2019; however, air conditioning also contributed to greenhouse gas emissions, air pollution, peak electricity demand, and urban heat islands

Indoor cooling is an effective strategy for preventing heat-related mortality.^145^ In this year’s report, this indicator combines the prevented fraction of deaths^146^ and heat-related death estimates from indicator 1.1.6 to track the number of heat-related deaths averted by air conditioning in people who are 65 and older. The methods for this indicator are described in appendix 5 (pp 92–102).

Applying country-specific and region-specific prevented fractions to the data from indicator 1.1.6 revealed that, in the absence of air conditioning, an estimated 195 400 more heat-related deaths would have occurred globally among people aged 65 years and older in 2019, in addition to the 345 000 heat-related deaths that are estimated to have occurred. In this age group, air conditioning averted an estimated 69 500 deaths in China (where 72 000 deaths attributable to heat exposure are estimated to have occurred in 2019 and 65% of households had air conditioning), 47 800 in the USA (where 20 500 deaths are estimated to have occurred and 92% of households had air conditioning), 30 400 in Japan (where 12 400 deaths are estimated to have occurred and 93% of households had air conditioning), but only 2400 in India (where 46 600 deaths are estimated to have occurred and 6% of households had air conditioning). These figures show the power of indoor cooling to prevent death and the inequities in access to indoor cooling across countries.

**Figure 10:**
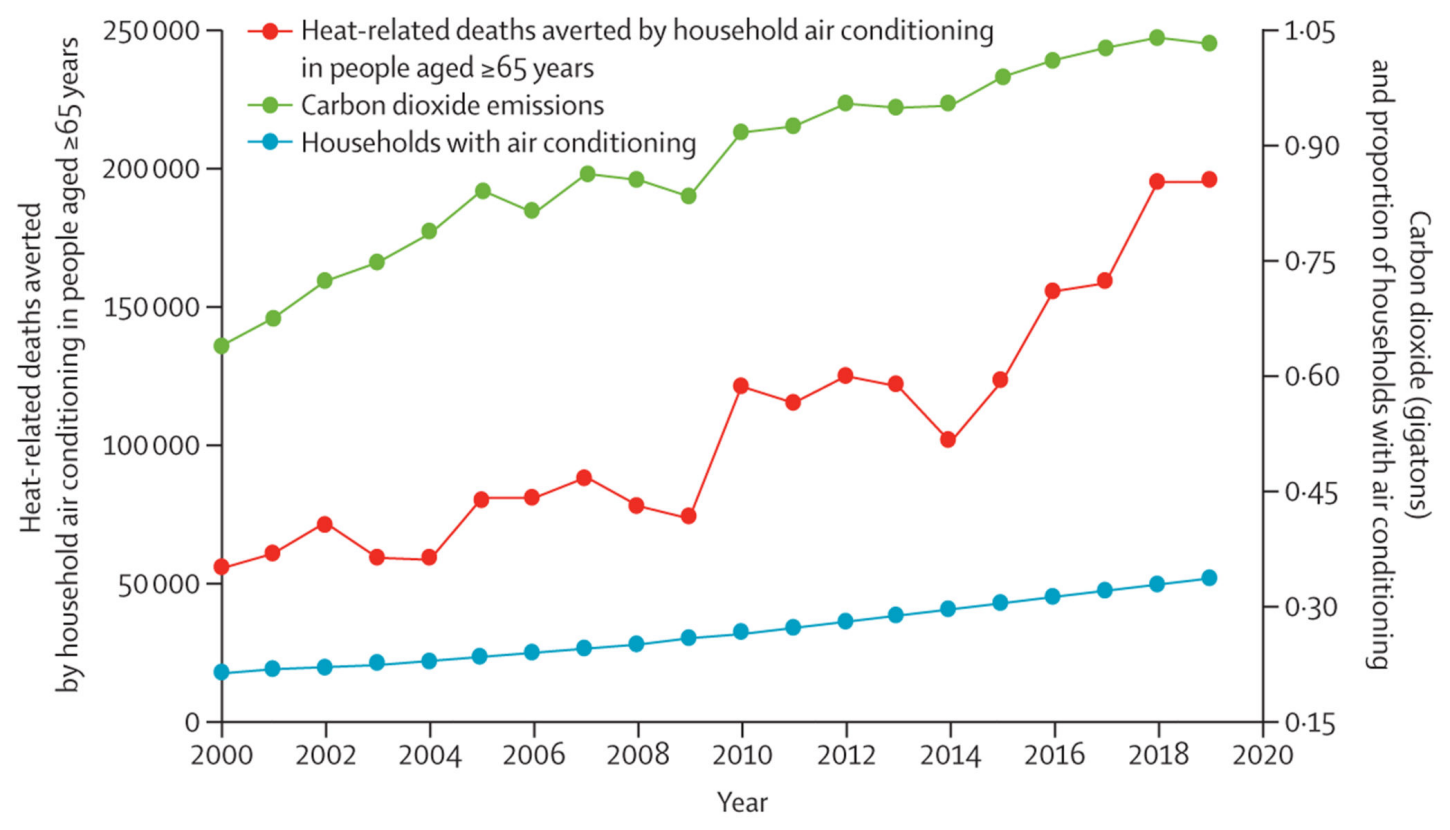
Global heat-related deaths of people aged 65 years and older and household air conditioning

Current air conditioning technology is unsustainable and leads to adverse health outcomes from increased air pollution, urban heat, and greenhouse gas emissions (see panel 3).^159^ In 2019, an estimated 21 000 deaths were attributable to exposure to PM_2·5_ from fossil-fuel powered electricity used for air conditioning, estimated with the same approach as in indicator 3.3. Between 2000 and 2019, the global proportion of households with air conditioning rose 57% and CO_2_ emissions from air conditioning use rose 61% (figure 10).

Sustainable indoor cooling approaches are urgently needed, including strong, enforced codes that mandate energy-efficient buildings,^159^ a return to traditional tropical and subtropical building designs,^159^ use of fans in climate zones where they provide effective cooling,^160^ stringent minimum energy performance standards for air conditioners,^159^ cool roofs (see panel 3), and increased urban green space (indicator 2.3.3).

#### Indicator 2.3.3: urban green space—headline finding: globally in 2020, 27% of urban centres were classified as being moderately green or above (an increase from 14% in 2010); the percentage of cities under this classification varied from 17% of urban centres in the low HDI country groups to 39% of urban centres in the very high HDI country group

There is growing evidence that access to urban green spaces provides benefits to human physical and mental health. These benefits include reducing exposure to air pollution, relieving stress, and increasing social interaction and physical activity, with overall improved general health outcomes and lower mortality risk.^161,162^ Green space also helps climate change mitigation and adaptation by sequestering carbon and delivering local cooling benefits. However, urban green spaces should be carefully designed and managed to conserve biodiversity, ensure they do not provide habitats and breeding sites for vectors of human diseases, or contribute to social inequities.^163–169^

This indicator provides an estimate of the magnitude of green vegetation in urban centres using the satellite-based normalised difference vegetation index (NDVI), with higher values indicating higher greenness levels. In the 2021 report, the sample size was increased to include 1029 urban centres across 170 countries. This sample encompasses all urban centres with more than 500 000 inhabitants and the most populated urban centre in countries that had no urban centres above this threshold. Full details are in appendix 5 (pp 103–107).

**Figure 11:**
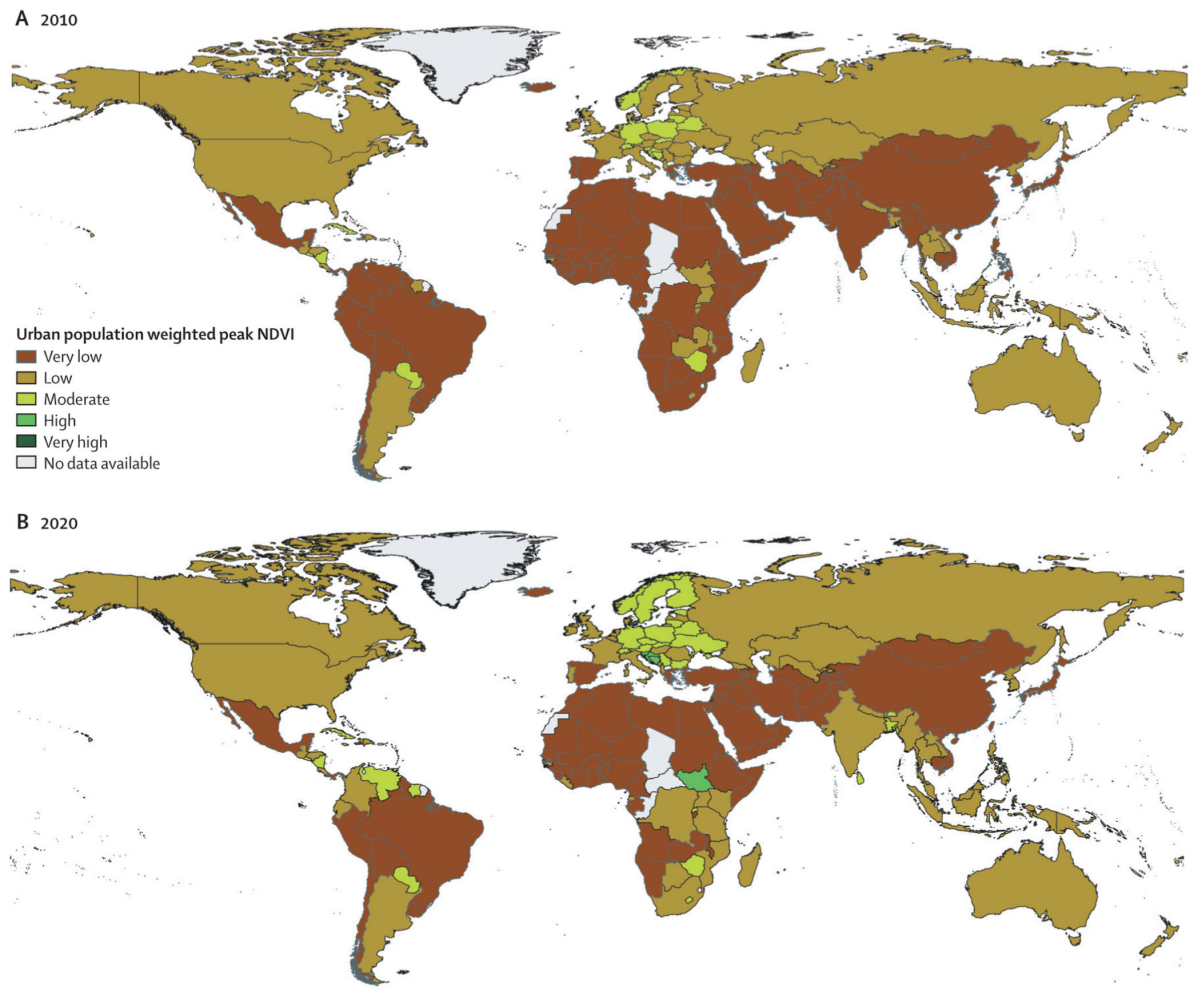
Average urban population-weighted peak NDVI in each country or territory Average urban population-weighted peak NDVI for 2010 (A) and 2020 (B). Urban centres with >500000 inhabitants were included in the data. For countries without an urban centre of >500000 inhabitants, the most populated urban centre was used in the analysis. NDVI=normalised difference vegetation index.

Averaged across all urban centres sampled, population-weighted peak NDVI increased from 0·26 to 0·32 (23%) between 2010 and 2020, with 27% of urban centres being classified as moderately green or above (ie, NDVI ≥0·40) in 2020 (figure 11). The level of greenness varies greatly by HDI level. In the very high HDI country group, 39% of urban centres had at least moderate levels of greenness (mean NDVI 0·34) in 2020, compared with 17% (mean NDVI 0·27) in the low HDI country group, 36% (mean NDVI 0·33) in the medium HDI country group, and 15% (mean NDVI 0·30) in the high HDI country group. This discrepancy highlights the inequities in the availability of green spaces between urban centres.

With its potential to simultaneously improve health outcomes, reduce health inequities, and facilitate climate mitigation and adaptation, urban green space design should involve interdisciplinary experts to ensure the health and environmental benefits are maximised.^170^ With health at the centre of planning in areas such as housing, transport, energy, and water and sanitation, urban centres can be places that are safe, comfortable, and enjoyed by everyone.^171^

### Indicator 2.4: health adaptation-related global funding and financial transactions

*Headline finding: globally, adaptation funding that is directed at health systems represents a small proportion of total climate change adaptation funding (0·3%), and only 5·6% of all transactions with adaptation potential were relevant to health in 2019–20*

This indicator monitors two elements of spending that could provide adaptation for health. The first element is the global funding approved for health-related adaptation projects through multilateral funds. The second element is global financial transactions with the potential to deliver adaptation in the health and care sector and other sectors that are relevant to the determinants of health (eg, waste and water management, built environment, or agricultural sectors). The first element draws on data from the Climate Funds Update Data Dashboard, whereas the second uses the Adaptation and Resilience to Climate Change (A&RCC) dataset produced by kMatrix. These complementary elements provide an evaluation of proactive adaptation funding that is potentially related to health and the global size of all economic transactions that can offer climate change adaptation potential for health.

Between 2018 and 2020, US$5·1 billion of multilateral climate change adaptation funding was approved globally. Only $711 million (13·9%) of this adaption funding was related to health. Adaptation funding that was related to health consisted of $14·0 million (0·3%) of approved funding directed specifically at health systems and $697 million (13·6%) of funding with potential secondary benefits for health.

**Figure 12:**
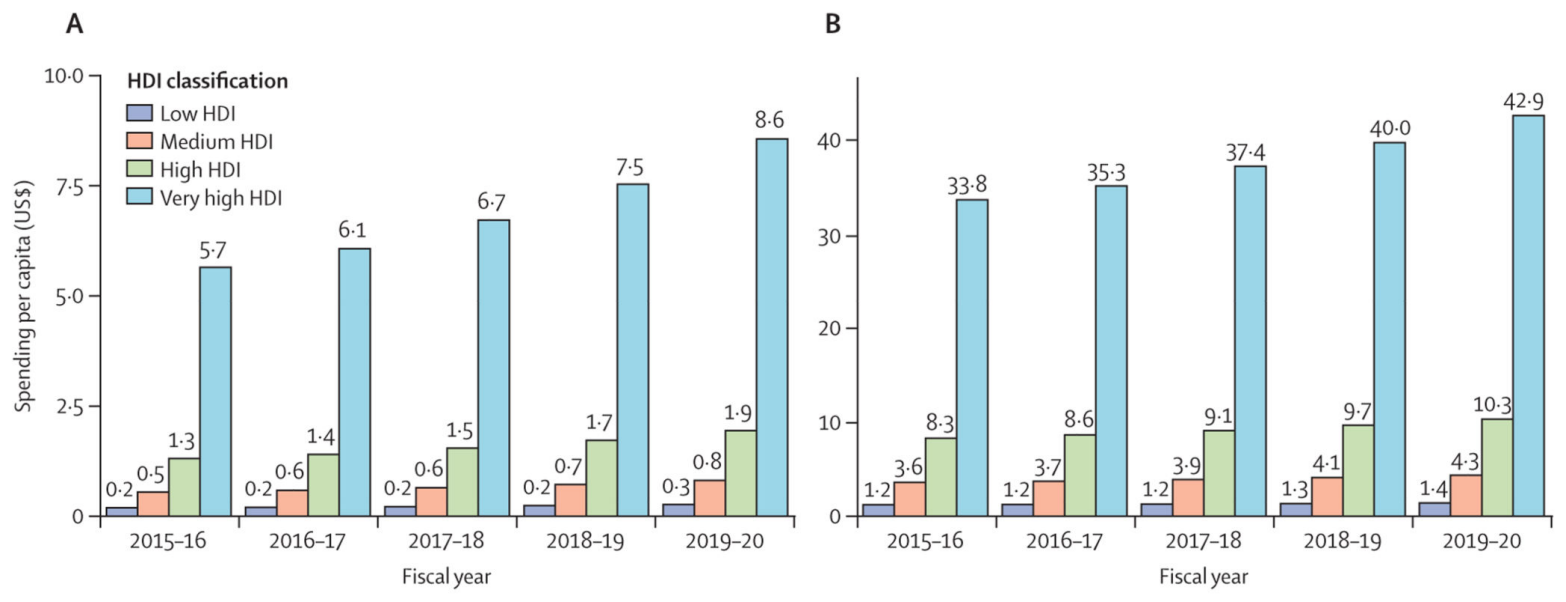
Spending per capita for potential adaptation to climate change for health Data for the health and health-care sector (A) and health-relevant sectors (B; see appendix 5 [p 109] for definition) in each 2019 HDI group. HDI=human development index.

The value of all financial transactions with the potential to deliver adaptation for health (ie, adaptation-relevant transactions within the dataset-defined health and health-care sectors) increased by 14·0% from 2018–19 to 2019–20, reaching 5·6% of total adaptation spending. Spending in other sectors that could be relevant to health (eg, in the waste and water management, built environment, or agricultural sectors) is estimated to have increased by 7·6% from 2018–19 to 2019–20, representing 28·6% of total transactions. Grouped by HDI, $234 million (1%) of spending was in low HDI countries, $1·8 billion (8%) was in medium HDI countries, $5·7 billion (27%) in high HDI countries, and $13·3 billion (64%) in very high HDI countries (figure 12). For spending in health-relevant economic sectors, a similar narrative emerges, in which $1·2 billion (1%) of spending occurred in low HDI countries compared to $66·7 billion (62%) in countries with a very high HDI. As the data covers financial years, the data (up to March 31, 2020) in this indicator are unlikely to reflect the anticipated economic impact of the COVID-19 pandemic on adaptation spending.

These findings highlight a growing global market for health-relevant adaptation transactions, but this growth has yet to translate into sufficient targeted health adaptation funding. As world economies recover from COVID-19, sufficient resources should be redirected towards health adaptation to build resilience to the increasing health threats of climate change.

### Conclusion

The indicators in this section show a complex landscape of adaptation, planning, and resilience for health in the past 12 months, in which the small global improvements to adaptation planning and assessment (indicators 2.1.1, 2.1.2, and 2.1.3) and intersectoral collaboration (indicator 2.2) are overshadowed by slow progress in implementation (indicators 2.3.2 and 2.3.3) and insufficient investment (indicator 2.4). A key theme across all the indicators is inequity and, although these indicators mostly track inequities between countries, within-country inequity is a substantial challenge in moving towards resilience and sustainability.

Although the world economy and health systems are recovering from a substantial acute global health crisis, climate change poses a much greater health threat in the coming decades. It is crucial that organisations and institutions capitalise on the insights generated from the pandemic to improve adaptability and resilience. Research is needed to identify current and future vulnerabilities, project risks from climate change at scales relevant for decision making in different climate and development scenarios, and identify and evaluate adaptation options to prepare for and protect health in a changing climate. Adaptation plans should be reviewed and updated to consider medium-term and long-term risks of climate change for health and to build resilience. Greater collaboration and coordination are necessary across public and private sectors and global institutions, along with increasing investments in adaptation.

## Section 3: mitigation actions and health co-benefits

Continuing an unbroken upward trend, global atmospheric CO_2_ concentrations passed 415 ppm in January, 2021, and, for the first time, the CO_2_ concentrations for much of 2020 are expected to be 50% higher than the 1750–1800 average.^19^ Total emissions of all greenhouse gases in 2019 were 59·1 GtCO_2_e (SD 5·9), which includes greenhouse gases generated by land-use changes. To limit warming to 1·5°C, annual global emissions must be reduced to 25 GtCO_2_e by 2030.^172^

COVID-19 and the associated lockdowns across the globe have had profound impacts on the global economy, most prominently in the surface and air transportation and industrial sectors.^173^ Emissions from very high HDI countries, which account for 48% of the global total, were around 10% lower than 2019 levels.^173^ However, without targeted intervention, emissions will rebound as the world recovers from the pandemic. Indeed, the 5·8% drop in energy-related CO_2_ emissions seen in 2020 is forecast to be matched with an unprecedented 4·8% rise in 2021.^23^

The necessity of steering the economic recovery to a lower-emissions pathway has been well publicised, but it has yet to be well-integrated into recovery plans (see panel 4).^181^ Nevertheless, the COVID-19 recovery presents the challenge and simultaneous opportunity to encourage action that yields benefits to health.

Tracking this global challenge, section 3 covers the relationships between climate change mitigation actions and health. This section provides an overview of the global energy system (indicator 3.1) alongside the associated global exposure to ambient PM_2·5_ air pollution and its health impacts (indicator 3.3). Energy use in the home is also reported, with new detail on fuels used and estimates of indoor air pollution concentrations (indicator 3.2). Individual sectors are then examined—namely, transport (indicator 3.4), food and agriculture (indicators 3.5.1 and 3.5.2), and the global health-care sector (indicator 3.6). Where possible, the ways in which relationships between health and climate change mitigation influence, and are influenced by, societal inequities are explored.

### Indicator 3.1: energy system and health

*Headline finding: from 2014 to 2018, despite strong growth in renewable energy in countries with a very high HDI, the carbon intensity of the global energy system has seen an annual average decline of just 0·6%, which is a rate incompatible with meeting the ambitions of the Paris Agreement*

Fossil fuel combustion within the energy system is the largest single source of greenhouse gas emissions, with a global share of 65%.^172^ The rapid shift from coal to renewable energy use is crucial, not only to mitigate these emissions but also to prevent deaths due to ambient air pollution (indicator 3.3) and eliminate other harmful pollutants related to coal mining and combustion.^182^ With data from the International Energy Agency (IEA), this indicator tracks three components—namely, the carbon intensity of the global energy system, coal phase-out, and zero-emission electricity. Full details are described in appendix 5 (pp 111–117).

The carbon intensity of the global energy system fell slightly for the fifth year in a row to 56·0 tCO_2_e/TJ (excluding land use emissions) in 2018. However, progress remains very slow, with an annual rate of decline of just 0·6% from 2014 to 2018. At this rate, it would take more than 150 years to fully decarbonise the energy system (far from the 2040 deadline required to keep temperature rise to 1·5°C).^183^ Progress has been made in the very high HDI country group since 1970 and carbon intensity in the high HDI country group could be at a possible peak. However, driven by the need to develop, the low and medium HDI country groups have had a sustained growth in emissions per unit of energy since 1970.

China continues to dominate global coal consumption, representing 18·1% of the world’s population and accounting for 53% of global coal use in 2019. While global coal use for all activities fell 1·2% in 2019, including a fall of 13·4% in the USA and 21% in Europe, China’s usage grew by 1·1%.

Between 2013 and 2018, electricity generation from renewable wind and solar energy increased by an annual average of 17%, with its global share of electricity generation reaching 7·2% in 2018. While total energy demand for coal, gas, oil, and nuclear fell in 2020, the production of electricity from renewable sources grew by a small amount (0·9%).^184^

Global coal demand is expected to rise by 4·5% in 2021, 80% of which is due to rapid increases in coal-fired electricity generation, and demand for renewable energy is set to rise by more than 8%.^23^ A redirection of efforts towards the decarbonisation of the energy system (see panel 4) could put the world on track to meet the 1·5°C temperature goal and prevent deaths associated with climate change and air pollution.

### Indicator 3.2: clean household energy

*Headline finding: in 2019, only 5% of rural households in countries in the low HDI country group relied primarily on clean fuels and technologies for cooking (up from just 2% in 2000), putting them at risk of morbidity and mortality due to exposure to household air pollution*

Around 10% of the world’s population, three-quarters of whom live in sub-Saharan Africa, do not have access to electricity for any service provision and 2·6 billion people do not have access to clean fuel for cooking.^184,185^ COVID-19 poses additional impediments to achieving SDG 7 (access to clean energy), with 2020 seeing a 2% rise in people without access to electricity in sub-Saharan Africa,^186^ driving low-income communities in places such as Nairobi to increase their usage of wood and kerosene.^187^ Energy poverty remains a concern even in high and very high HDI countries and around 7% of people in the EU struggle to afford sufficient heat for their homes,^188^ putting them at risk of cold-related adverse health outcomes.^189^ Energy poverty related to excess heat is also an important issue around the world (as highlighted in panel 3).^190^

This indicator tracks energy usage in the home using data from both the IEA and WHO.^185,191–193^ The WHO household energy database compiled data from national surveys, presented here from 2000 to 2017 and projected for 2019, which provides information on fuels and technologies used for cooking, heating, and lighting. With these data, this indicator also estimates household air pollution concentration for 29 countries. A full description of the methods, data, and caveats is given in appendix 5 (pp 118–121).

In the low HDI country group, domestic energy use is dominated by biofuels. Primary reliance on clean fuels and technologies for cooking in households in the low HDI country group was estimated to have been at only 12% in 2019. This proportion is even lower in rural households in the low HDI country group, with only 5% relying on clean fuels and technologies—a marginal increase from 2% in 2000. In homes in the medium and high HDI country groups, the share of solid biofuel use has fallen more rapidly than in the low HDI country group, and clean cooking fuel and technology use has risen substantially; although, in rural areas, use of solid biofuel remains at 54% for the high HDI group and 39% for the medium HDI group.

These patterns of energy use, ventilation practices, and the infiltration of air have implications on household air pollution concentrations. In rural households in several low and medium HDI countries, the average PM_2·5_ concentration in the main indoor cooking area is estimated to be more than 500 μg/m^3^. In Ethiopia, the average PM_2·5_ concentration in indoor cooking areas is more than 1200 μg/m^3^, 120 times the WHO threshold of 10 μg m^3^.^194^ Exposure to these harmful air pollutants in the home results in an estimated 2·31 million deaths per year globally.^195^

Although gender-differentiated effects might change across different geographies and cultures,^196^ exposure to household air pollution is estimated to be around 40% higher for women than for men.^197^ In many places, women are also at higher risk of musculoskeletal injuries and violence due to travel through unsafe areas that result from their domestic role in collecting and using fuels for cooking and heating, which poses additional risks to their physical and mental wellbeing.^198–201^ Thus, progress towards meeting SDG 7 would improve health and reduce gender inequities.

### Indicator 3.3: mortality from ambient air pollution by sector

*Headline finding: 3·3 million deaths were attributable to ambient PM*_*2·5*_ *pollution from human sources in 2019, a third of which were directly related to fossil fuel combustion; the medium and high HDI country groups had the highest mortality rates*

Awareness of the health impacts of air pollution has increased during the past years. Legislation shifts include the proposed revision of the EU ambient air quality directives^202^ and a landmark ruling on the death of nine-year-old Ella Adoo-Kissi-Debrah in 2020 in the UK, which is thought to be the first time that air pollution has been listed as a cause of death in a death certificate.^203^ This indicator estimates ambient PM_2·5_ exposure and the resulting attributable deaths from different economic sectors. For the 2021 report, the methods have been updated to use the integrated exposure-response functions (meta-regression-Bayesian regularised trimmed) used by the Global Burden of Disease Study 2019.^204^

In total, 4·0 million deaths were estimated to be attributable to exposure to ambient PM_2·5_ in 2019, 3·3 million of which were from anthropogenic sources and 1·1 million were directly related to fossil fuel combustion. Deaths due to coal combustion have decreased from 620 000 in 2015 to 507 000 in 2019, largely due to strict air pollution control measures in China, including the reduction of coal for residential heating.

**Figure 13:**
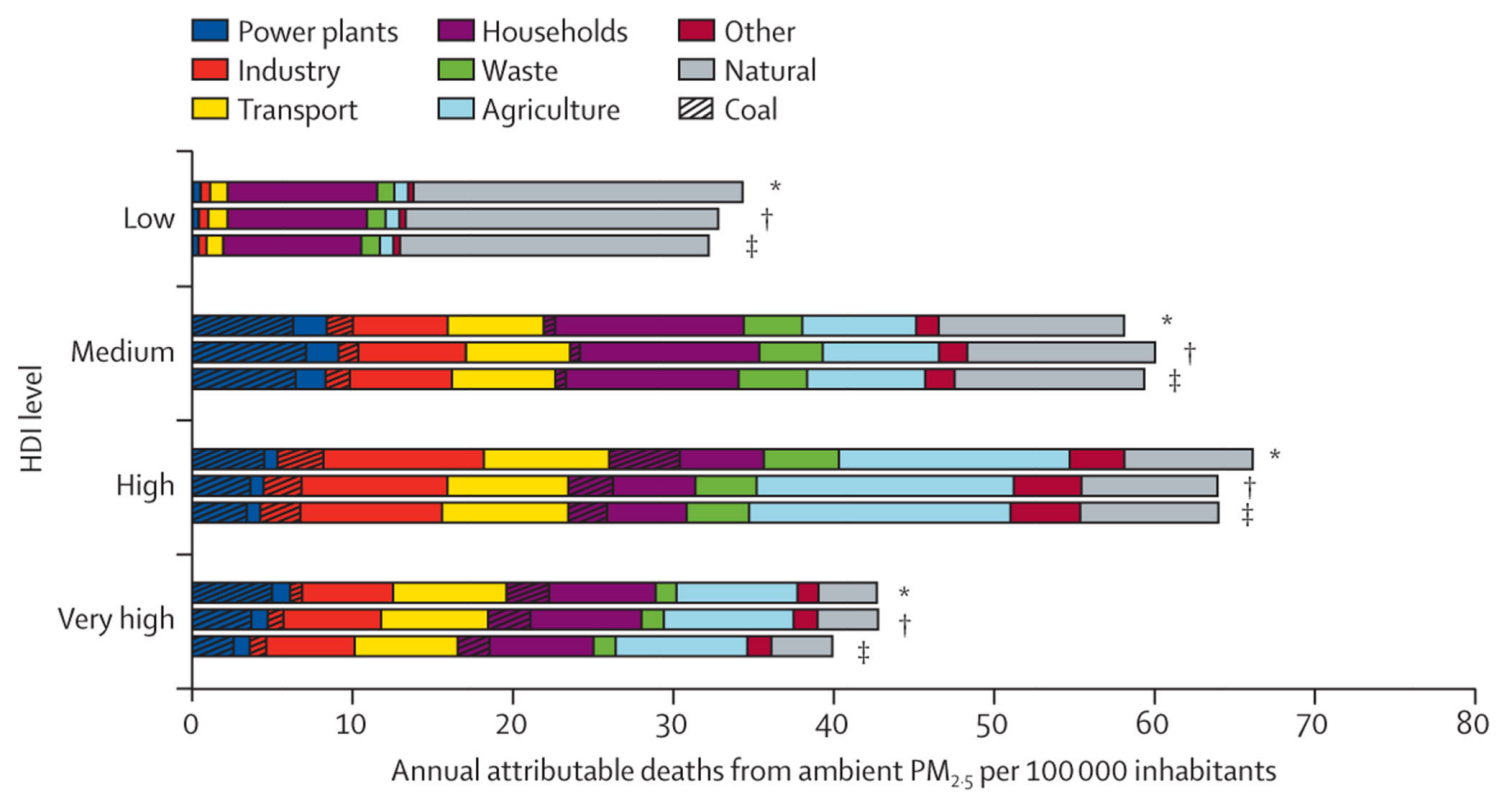
Deaths attributable to exposure to PM_2·5_ in 2015, 2018, and 2019 by key sources of pollution and 2019 HDI groups HDI=human development index. PM_2·5_=fine particulate matter. *2015. †2018. ‡2019.

Ambient concentrations of PM_2·5_ differ strongly across world regions and between urban and rural areas. As a result of higher industrial activity than in other HDI groups, poorer emissions controls, and the continuing use of solid fuels in the domestic sector, countries in medium and high HDI groups have the highest rates of air pollution-related mortality (60 deaths per 100 000 inhabitants in the medium HDI country group and 65 deaths per 100 000 inhabitants in the high HDI country group; figure 13). Deaths are lower in the low HDI country group (34 deaths per 100 000 inhabitants) and the very high HDI country group (40 deaths per 100 000 inhabitants). These results are due to lower industrial activity and younger populations in countries with a low HDI and cleaner electricity generation, industrial production, and end-of-pipe emission controls in countries with a very high HDI.

### Indicator 3.4: sustainable and healthy road transport

*Headline finding: electricity use in transport rose by 15% from 2017 to 2018 and the global electric vehicle fleet topped 7·2 million cars in 2019; however, emissions from road transport also continued to increase*

With road transport accounting for nearly 18% of global CO_2_ emissions in 2019, the shift to electric vehicles is an important mitigation measure.^205^ Beyond this shift, the promotion of walking and cycling (ie, active travel) could cut emissions and provide enormous health dividends through the increase of physical activity.^206^ The mode share of cycling varies greatly between and within countries with different levels of HDI. For example, cycling makes up 0·3% of all trips in São Paulo; 0·6% of all trips in Cape Town; 1·1–1·9% of all trips in US and Australian cities; 4·8% of trips in Delhi; and 14·1–28·7% of all trips in cities in Germany, Japan, and the Netherlands, with a higher mode share being associated with more equal gender representation in cycling.^207^ Unless active travel infrastructure is rolled out with consideration of sociocultural inequities, the benefits might not be equally manifested across all populations.^208–212^

This indicator uses data from the IEA to monitor fuels used for transport and electric vehicles, with full details provided in appendix 5 (pp 124–125).^213–215^ The global number of electric vehicles rose from 5·1 million in 2018 to 7·2 million in 2019. However, electric vehicles still only represent 1% of global car stock, and road transport emissions increased in 2019 as demand for larger vehicles grew in the USA, Europe, and Asia in tandem with increasing demand for transport in low and medium HDI countries. Overall, total direct use of fossil fuels for road transport increased by 0·7% between 2017 and 2018 and the use of electricity in transport rose by 15%—although electricity remains just 0·27% of total road transport energy use.

Between January and March, 2019, the COVID-19 pandemic led to a nearly 50% decrease in global road transport demand.^216,217^ Although the use of fossil fuels for road travel has mostly rebounded, many public transport networks now face critical decreases in use.^218^ City governments around the world had implemented measures to promote active travel during their lockdowns, many of which are intended to be permanent.^216,217^ As cities emerge from the COVID-19 crisis, implementing policies to reinforce positive shifts in travel mode would promote physical activity, reduce urban air pollution, and mitigate climate change.^219^

### Indicator 3.5: food, agriculture, and health

#### Indicator 3.5.1: emissions from agricultural production and consumption—headline finding: mostly caused by high quantities of red meat consumption, per-capita emissions from food consumption are considerably greater in the very high HDI country group than in other HDI country groups and are 41% higher than in the low HDI group in 2018

Food systems, including agricultural production, cause 21–37% of all greenhouse gas emissions and also hold high carbon sequestration potential.^45^ These emissions make food systems key to limiting global warming to 1·5°C. This indicator tracks emissions from agricultural production and consumption of food products, combining modelling and FAO data.

Despite moderate improvements in efficiency, total agricultural production emissions continued to grow, reaching 5·6 GtCO_2_e in 2018 (1·5% higher than in 2017). Of this total, cattle products (mainly meat and milk) contributed 52% of global agricultural production emissions.

Data reveal stark differences in agricultural emissions based on per-capita consumption across countries in different HDI groups. Per-capita emissions in the very high HDI country group are 39% higher than in the high HDI group and 41% higher than in the low HDI group. These differences in emissions are despite high emission-intensity beef farming in the low HDI group (around three times higher than in the very high HDI group), which is mitigated by a much lower per-capita consumption of beef. 68% of the total consumption-based agricultural emissions in the very high HDI country group are attributable to cattle products, mainly beef production, which is slightly down from 71% of the total consumption-based agricultural emissions in 2000.

Progress towards zero hunger (SDG 2) will probably be associated with increases in consumption-based agricultural emissions in low and medium HDI countries. To meet emission reduction goals, consumption of red meat should be safely reduced in relevant population groups, especially in very high HDI countries.^220^ This reduction would also deliver substantial health co-benefits, as indicator 3.5.2 shows. Additional scope to reduce emissions from the food production system comes from waste reduction, deforestation curtailment, and yield improvement.^221^

#### Indicator 3.5.2: diet and health co-benefits—headline finding: between 2017 and 2018, estimated deaths due to excess red meat consumption rose by 1·8% to 842 000

With current production efficiency interventions failing to curb or reduce agricultural greenhouse gas emissions, dietary shifts (eg, greatly reducing red meat and increasing plant-based foods consumption) are necessary, particularly in the very high and high HDI countries.^206^ For the low and medium HDI countries, sustainable farming and agricultural practices will help keep agricultural emissions low while efforts are made to meet the nutritional requirements of populations.^222^ To monitor this dietary transition, this indicator models deaths attributable to dietary risk factors with updated data on food consumption and mortality rates by sex, age, and country.^223,224^

In 2018, 9·6 million deaths were attributable to imbalanced diets (both dietary composition and caloric intake). Although dietary risks and baseline mortality rates declined in 2018, there was an overall increase in diet-related mortality compared with 2017 (see appendix 5 pp 132–140). Diets in the high and very high HDI country groups contain four to seven times more red meat than diets in the low and medium HDI groups. Together with greater non-communicable disease-related mortality rates, the difference in diets translates to a rate of red meat-related mortality almost nine times greater in the very high HDI country group (19 deaths per 100 000 people) than in the low HDI group (2 deaths per 100 000 people).

**Figure 14:**
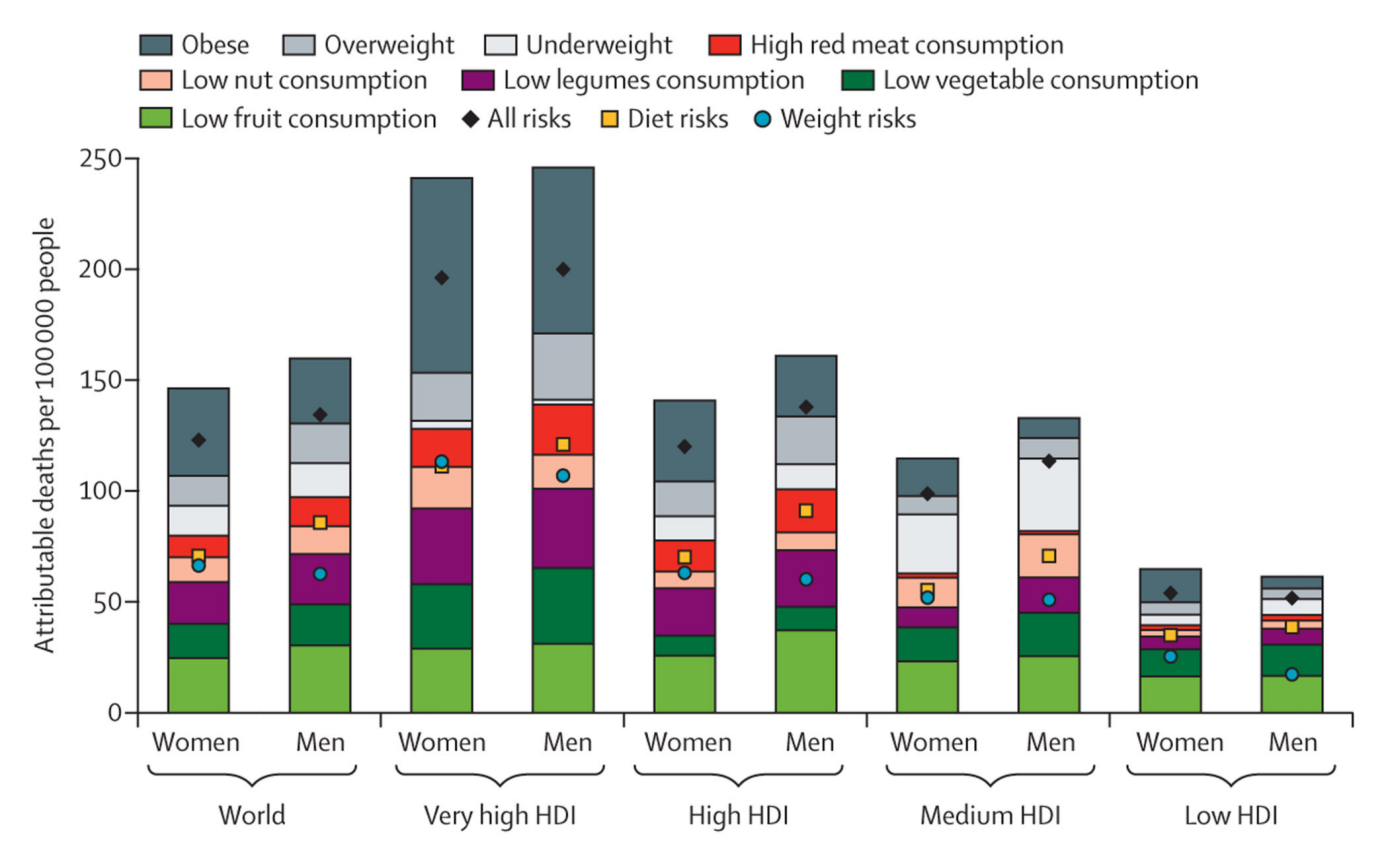
Deaths attributable to imbalanced diets and weight in 2018 by risk factor in each 2019 HDI group Each component in the stacked bar represents its individual contribution to attributable deaths. Since these contributions cannot be summed directly, the overall contribution by diet and weight components are represented by the dots as given in the key. HDI=human development index.

Diets, and the associated health impacts, differ across sexes. In general, the diets of men tend to be less healthy than the diets of women, containing 6% fewer fruits, 1% fewer vegetables, 10% fewer legumes, and 4% more red meat.^225–228^ The differences in diet resulted in an estimated 455 000 (10%) more men dying from preventable, diet-related diseases than women—a pattern reflected across each of the HDI country groupings (figure 14).

### Indicator 3.6: health-care sector emissions

*Headline finding: in 2018, emissions from the health-care sector increased slightly to 4·9% of global greenhouse gas emissions; health-care emissions are positively associated with HDI levels, mostly through health spending, but there is little association after 400 kg CO*_*2*_ *per capita*

The health-care sector is central to improving human development. In providing services, health-care systems mobilise a vast array of products and use energy in various forms, all of which result in emissions of greenhouse gases and other pollutants that can be calculated throughout global supply chains. With this contribution to greenhouse gas emissions and their important role in improving patient care in the face of climate change,^229^ health-care institutions are beginning to seriously commit to reducing emissions.^230^

In this indicator, both direct and indirect emissions from the global health-care sector are modelled with environmentally extended multiregion input–output models combined with annual WHO data on national health-care expenditure. A full description of these methods is in appendix 5 (pp 141–142).

In 2018, the global health-care sector contributed approximately 4·9% of global greenhouse gas emissions; a rise of 5·2% in health sector emissions since 2017. Expansion of health-care services in China contributed more than half of this global increase. Although China’s national health-care emissions are now 35% greater than those of the USA’s, on a per-capita basis, China ranks 21st among all major economies assessed.

Per-capita comparisons do not account for differences in health-care access and quality, specifically those measured through health outcomes, such as life expectancy (which is one of the components of the HDI). Plotting per-capita health-care emissions against HDI reveals that emissions are positively associated with HDI level, an association strongest for lower emissions. For example, a wide range of HDI levels are associated with per-capita health-care emissions of 500–600 kgCO_2_e, reflecting both differences in health system efficacy and other development indicators, but also in emissions intensities. Additional emissions above 500–600 kgCO2e are not associated with improved HDI.

### Conclusion

Before the pandemic, the rapid rate of growth in renewable electricity generation was insufficient to counteract the slow decline in coal use. The result of this was that the carbon intensity of the global energy system remained virtually unchanged. At the same time, there has been very little progress in increasing the use of clean household energy. These delays are costing millions of lives each year from household and ambient air pollution. Food-related agricultural emissions continue to rise and so do deaths attributable to dietary risk factors.

Across this section, many inequities can be highlighted. Low HDI countries have the highest use of dirty fuels in the home, putting people in low HDI countries at greater risk of morbidity and mortality from exposure to household air pollution. As a result of higher industrial activity and inadequate emissions controls, countries of medium and high levels of HDI have the highest carbon intensity of energy and the greatest amount of deaths due to ambient air pollution. People in very high HDI countries have the most carbon-intensive diets, and, with high amounts of red meat consumption, they also have the most to gain from a shift towards a more plant-based diet.

Although the effects of the COVID-19 pandemic are not yet fully known, there was a temporary, but substantial, drop in emissions due to lockdowns and the associated reductions in economic activities and international travel. However, emissions are already rebounding. The challenge moving forward will be to adopt measures that provide near-term economic relief while building towards long-term emission reductions and protecting future health—a challenge also explored in section 4.

## Section 4: economics and finance

Avoiding the worst of the climate change impacts described in section 1 will require both sustained adaptation efforts (section 2) and a rapid transformation of the world’s economies to cut greenhouse gas emissions (section 3). Section 4 examines the economic and financial implications of this transition.

First, this section explores the economic impact of climate change and its mitigation (indicators 4.1.1 to 4.1.4). These indicators use a range of methods to estimate some of the costs that climate change might already be imposing on society through its impacts on human health. Indicators 4.2.1 to 4.2.5 investigate the economics of the transition to zero-carbon economies, which are fundamental to the improvement of human health and wellbeing. These indicators consider whether investments and jobs are beginning to move away from fossil fuels and if the appropriate economic signals are encouraging this transition. A new indicator for this year’s report (indicator 4.2.5) explores the effect of global trade on greenhouse gas and PM_2·5_ emissions, highlighting that harms might occur in countries different from the demands that drive those emissions.

Achieving the required investments in the low-carbon transition requires clear and committed action from governments and private sector actors, and could result in health and economic benefits. Aiming for a green global recovery from COVID-19, rather than business-as-usual economic growth, will ensure economic recovery through

### Indicator 4.1: the economic impact of climate change and its mitigation

#### Indicator 4.1.1: economic losses due to climate-related extreme events—headline finding: when normalised by gross domestic product, economic losses from climate-related extreme events in 2020 were three times greater in the medium HDI country group than in the very high HDI country group

The loss of physical infrastructure and the resulting economic losses due to climate-related extreme events can exacerbate the health impacts described in section 1. This indicator tracks the total annual economic losses (insured and uninsured) that result from climate-related extreme events with data provided by Swiss Re.^240^ The methods are described in appendix 5 (pp 143–145).

In 2020, there were 242 recorded climate-related extreme events and absolute economic losses from these events totalled US$178 billion. Although two-thirds of these losses occurred in very high HDI economies, when normalised by gross domestic product (GDP), losses in the medium HDI country group were around three times greater than in the very high HDI country group. Although $76 100 million (66%) of $115 300 million of the losses in the very high HDI country group were insured, almost $34 200 million (93%) of $36 900 million of losses were uninsured in the high HDI group. The uninsured measurable losses rise to $24 200 million (97%) of $25 000 million in the medium HDI group and $576 million (100%) in the low HDI country group, which creates a larger economic burden and reinforces inequities for disadvantaged countries, as uninsured losses are either not replaced or are replaced through out-of-pocket expenses.

#### Indicator 4.1.2: costs of heat-related mortality—headline finding: the monetised value of global heat-related mortality increased by 6·7%, from 0·27% of gross world product in 2018 to 0·28% in 2019; Europe continued to be the worst affected region, facing costs equivalent to the combined average incomes of 6·1 million of its citizens

The increase in morbidity and mortality due to extremes of heat represents a high cost to all of society. This indicator uses data on years of life lost due to extremes of heat from indicator 1.1.6 to provide a measure of the costs of global deaths attributable to heat.^94^ Improved in the 2021 report, the indicator combines a value of statistical life-year with YLL to estimate the monetised loss caused by deaths attributable to heat. The valuation of life across varying HDI levels shows a methodological and ethical challenge, which this indicator addresses by presenting the cost of deaths attributable to heat as the proportion of GDP and the equivalent annual average income.

**Figure 15:**
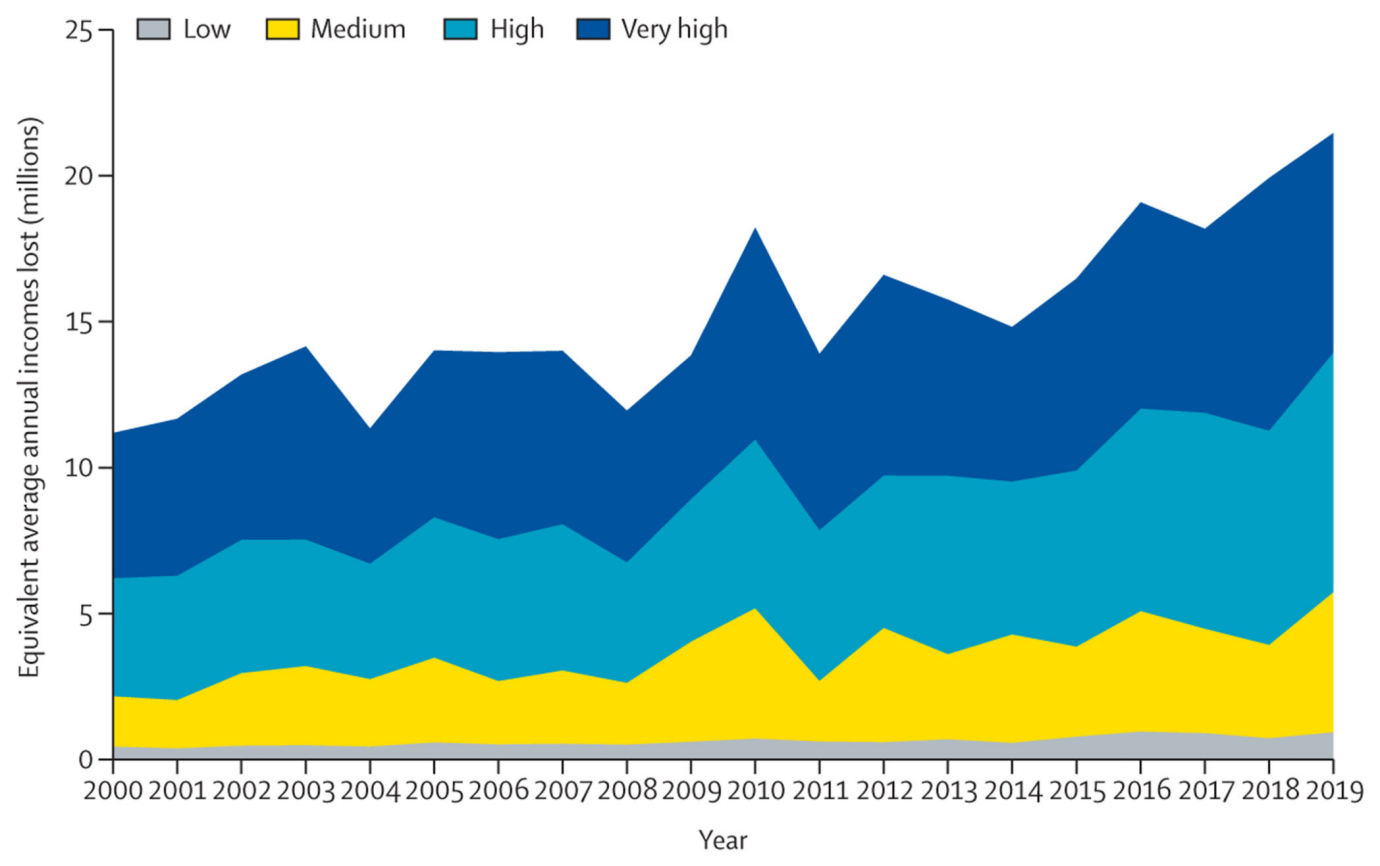
Monetised cost of heat-related deaths by 2019 HDI group Monetised costs are expressed as the equivalent number of annual incomes of the average person lost. HDI=human development index.

The monetised value of global heat-related mortality in people 65 years or older increased by 6·7%, from 0·27% of gross world product in 2018 to 0·28% in 2019 (figure 15). Reflecting the distribution of impacts found in indicator 1.1.6, the costs of heat-related mortality were found to be equivalent to the average combined incomes of 0·94 million of their citizens for the low HDI country group, 4·80 million of their citizens in the medium HDI country group, 8·20 million of their citizens in the high HDI country group, and 7·52 million of their citizens in the very high HDI country group. As in indicator 1.1.6, WHO’s European region was the worst affected in 2019, with costs equal to the average income of 6·1 million of its citizens and 0·66% of regional GDP. However, the costs were lower in 2019 than in 2018 due to fewer estimated heat-related deaths in the European region (indicator 1.1.6). However, costs increased in other regions between 2018 and 2019, especially WHO’s South-East Asia region.

#### Indicator 4.1.3: loss of earnings from heat-related labour capacity reduction—headline finding: working in conditions of extreme heat is a health risk; such conditions could reduce the capacity for paid labour, with an impact on workers’ earnings equivalent to 4–8% of GDP in the low HDI country group in 2020

As reflected in indicator 1.1.4, increased temperatures, driven by climate change, are affecting people’s ability to work. This indicator considers the loss of earnings that could result from such reduced capacity. Losses of earnings could compound the health impacts of extreme heat through effects on the socioeconomic determinants of good health.^241^ Indicator 4.1.3 combines the outputs of indicator 1.1.4 with data on average earnings by country and sector held in the International Labour Organization databases.^242^ The methods and additional analyses are described in appendix 5 (pp 148–154). In this year’s report, the number of countries included in this indicator has been increased from 25 to 183.

**Figure 16:**
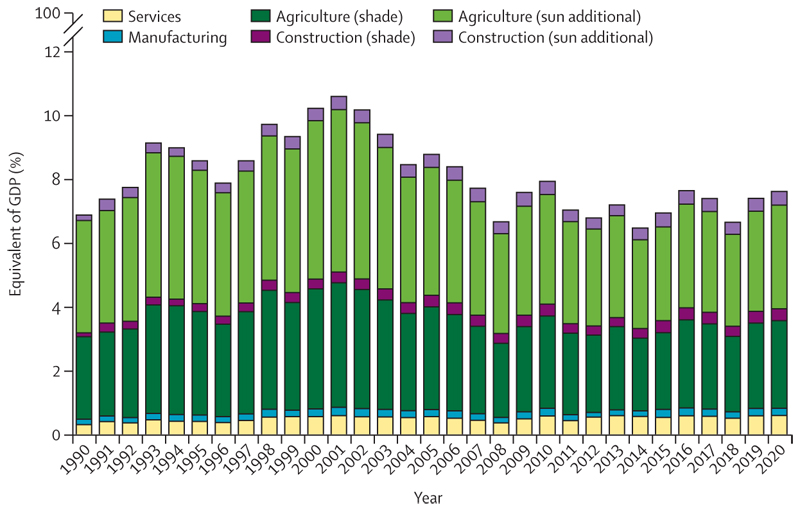
Average potential loss of earnings in the low HDI group as a result of potential labour loss due to heat exposure Losses are presented as a share of GDP by sector of employment. The agriculture and construction (sun additional) blocks represent the losses that would have been incurred in addition to those from agriculture and construction (shade) if all of the activities in these sectors had been carried out in direct sunlight. GDP=gross domestic product.

Indicators 1.1.6 and 4.1.2 found Europe to be the region most affected by heat-related mortality in people aged 65 years older. In contrast, this indicator focuses on working-age populations and, in alignment with the outputs of indicator 1.1.4, it finds that greater losses of earnings due to reduced labour capacity occur in low and medium HDI countries. Countries with lower HDI levels tend to have greater proportional losses of earnings, emphasising the impact of climate change on deepening inequities. In the low HDI country group, potential income losses in 2020 were equivalent to 3·9–7·6% of GDP, depending on the degree of shade or sun exposure during agricultural and construction work (figure 16). Potential income loss in 2020 was 2·2–4·1% of GDP in the medium HDI group, 0·9–1·5% in the high HDI group, and 0·3–0·5% in the very high HDI country group. These potential losses will mainly affect men who work in sectors such as construction, where they represent more than 90% of the global workforce, and in manufacturing and agriculture, where they represent more than 60% of the global workforce.^83^ However, these data do not account for informal or unpaid domestic and agricultural work—a group in which women are often over-represented.^243–245^ The indirect economic impacts from reduced labour capacity extend well beyond the loss of earnings. For example, modelling both direct and indirect impacts, the heat-related economic cost of labour loss in 2020 was estimated to be 1·36% of China’s GDP and 6·75% of the GDP in Hainan.^50^

#### Indicator 4.1.4: costs of the health impacts of air pollution—headline finding: equivalent to the average annual income of 78·1 million and 99·1 million people, the greatest economic costs of mortality due to air pollution fall on countries in the medium and high HDI country groups; costs relative to GDP decreased between 2015 and 2019 globally, with the exception of costs in southeast Asia

As described in indicator 3.3, global mortality due to ambient PM_2·5_ pollution has increased. This indicator captures the cost of this mortality by placing an economic value on the YLLs that result from exposure to anthropogenic ambient PM_2·5_. This indicator has been expanded for the 2021 report, from a European-only focus to global coverage, and has a revised definition of YLLs. The methods, data, and further analysis are described in appendix 5 (pp 155–157).

**Figure 17:**
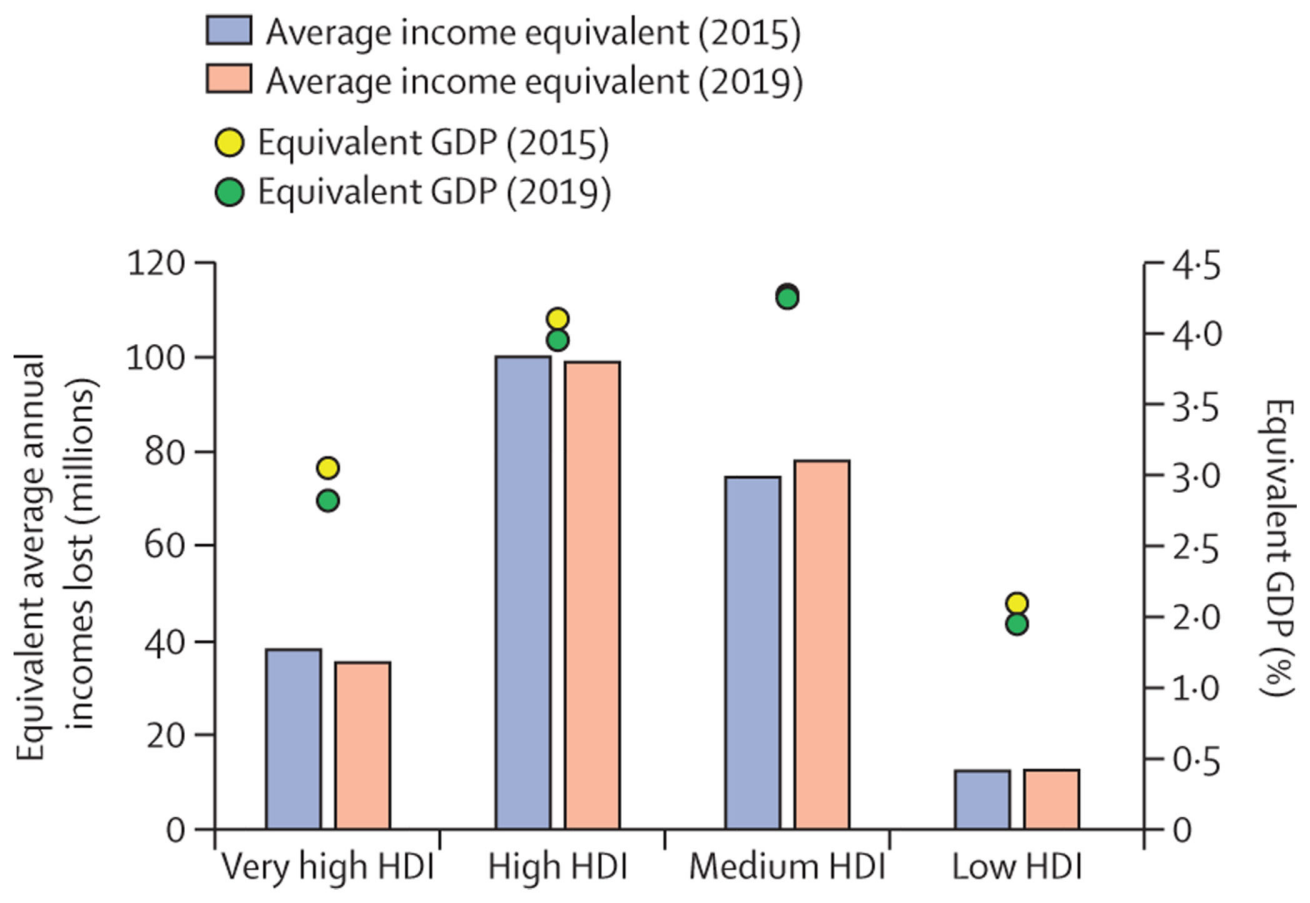
Cost of year of life lost in 2015 and 2019 The equivalent number of annual incomes of the average person lost and total GDP in each 2019 HDI group. GDP=gross domestic product. HDI=human development index.

Figure 17 presents the economic value of YLLs in 2015 and 2019 by country HDI group, relative to both total GDP and the annual income of the average person in these categories. The medium and high HDI country groups have the greatest relative costs from YLLs attributable to ambient PM_2·5_ exposure, equivalent to the annual average income of 78·1 million people in the medium HDI country group and 99·1 million people in the high HDI country group. Costs relative to average income increased between 2015 and 2019 in the low and medium HDI country groups. However, with the growth of GDP outpacing the growth of the population, costs relative to total GDP have decreased in all HDI groups.

### Indicator 4.2: the economics of the transition to zero-carbon economies

#### Indicator 4.2.1: coal and clean energy investment—headline finding: global investment in energy supply and energy efficiency reduced by 13% between 2019 and 2020; investment in renewable energy and energy efficiency increased by 3% and investment in new coal capacity reduced by 13%

Coal combustion has been responsible for more than 30% of the global average temperature increase above pre-industrial levels and for 491 000 deaths from PM_2·5_ exposure in 2019 (indicator 3.3).^246^ Therefore, coal phase-out is essential for both mitigating climate change and for reducing premature mortality due to air pollution. At the same time, it is necessary to invest in renewables, energy efficiency, and the electricity grid to reduce the carbon intensity of energy supply, as described in indicator 3.1. Taking data from the IEA, this indicator tracks global investment in energy supply and energy efficiency, and highlights ongoing capital spending in new coal-fired power generation globally and for key countries and regions. These data, presented as an index, represent ongoing capital spending.

**Figure 18:**
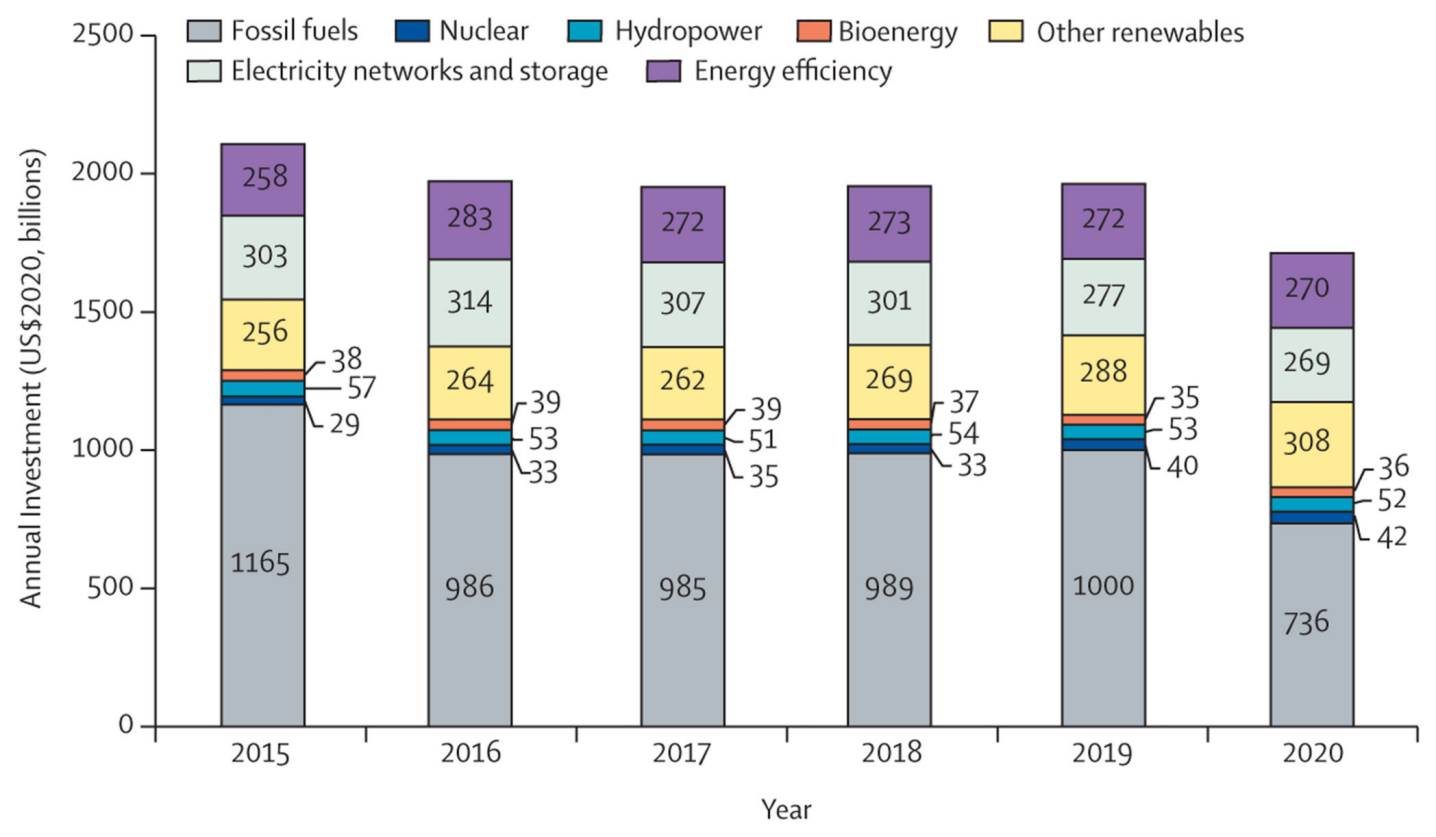
Annual investment in energy supply and energy efficiency globally

Between 2019 and 2020, investment in global energy supply and energy efficiency reduced from nearly US$2 trillion to about $1·7 trillion (figure 18), almost entirely due to declining investment in fossil fuels after reduced demand as a result of the COVID-19 pandemic (investment in coal power capacity declined by 13%). Investment in renewables and energy efficiency increased by 3% between 2019 and 2020, with their share of total investment in global energy supply increasing from 33% to 39%. However, to maintain a maximum of 1·5°C of warming this century, annual investments in clean energy must at least triple during the 2020s.^247^

#### Indicator 4.2.2: employment in low-carbon and high-carbon industries—headline finding: direct employment in fossil fuel extraction declined by 14% from 11·6 million employees in 2019 to 9·9 million in 2020

Evidence supports that employees in some fossil fuel extraction industries, particularly coal mining, and their local communities, have a greater incidence of cardio-vascular and cerebrovascular disease, respiratory disease, and cancers than the general population.^248^ Investments in renewable energies and energy efficiency are estimated to create almost three times more jobs per unit of spending than investments in fossil fuel industries.^249^ Along with strong labour and environmental standards, investment and employment in renewables present an opportunity to improve health and livelihoods. This indicator tracks global direct employment in fossil fuel extraction industries and direct and indirect (supply chain) employment in renewable energy. A full description is available in appendix 5 (pp 160–161).

Around 11·5 million people globally were employed directly or indirectly by the renewable energy industry in 2019, representing an increase of 4·2% from 2018. Currently, data for 2020 are unavailable; however, due to the pandemic, the extent to which such data will be indicative of a long-term trend is unclear. Fossil fuel extraction industries employed more people globally than all renewable energy industries combined in 2019, although the number of jobs in 2020 was slightly lower than in 2019, at 9·9 million compared with 11·6 million.

Although there are still more men than women in the energy sector, the field of renewable energy employs a considerably higher share of women (32%) than the oil and gas industry (22%).^250^ With adequate policies in place, the transition to a low-carbon economy therefore represents an opportunity to reduce gender inequities and empower women.

With trillions of dollars earmarked for COVID-19 recovery, investments in the renewable fuel industry could offer a triple gain in terms of better health through safer jobs and improved livelihoods, climate change mitigation, and increased employment opportunities.

#### Indicator 4.2.3: funds divested from fossil fuels—headline finding: the global value of funds committing to fossil fuel divestment between 2008 and 2020 was US$14·52 trillion, with health institutions accounting for $42 billion

By reducing financial interests in the fossil fuel industry, divestment both reduces the social licence of fossil fuel companies and hedges against investors’ risk of losses due to so-called stranded assets in an increasingly decarbonising world (panel 5).^251,252^ Investors can also effect change through shareholder action, exemplified recently by activist hedge fund Engine No 1 taking seats on ExxonMobil’s board.^253^ Concerned with the immediate and long-term damages of continued fossil fuel use, health institutions have the imperative to lead the way in divesting. This indicator tracks the total global value of funds divested from fossil fuels and the value of funds divested by health institutions using data provided by 350.org.^254^

From 2008 until the end of 2020, 1398 organisations, with assets worth at least $14·52 trillion, have committed to divestment. Of these organisations, only 25 are health institutions, with assets totalling $42 billion. The value of new funds committed to divesting in 2020 was $2·5 trillion, with health institutions accounting for $61 million of the value.

#### Indicator 4.2.4: net value of fossil fuel subsidies and carbon prices—headline finding: 65 (77%) of the 84 countries reviewed had a net-negative carbon price in 2018; the resulting net loss of revenue was, in many cases, equivalent to substantial proportions of the national health budget

Placing a carbon price on fossil fuel use helps to accurately reflect its negative externalities, including its impact on health, and to encourage the transition away from fossil fuels. However, not all countries set carbon prices, and, where they are imposed, they can be undermined by subsidies provided for fossil fuels.

This indicator compares carbon prices and fossil fuel subsidies to calculate net-economy-wide average carbon prices and revenues. The indicator includes 84 countries, which are responsible for around 92% of global CO_2_ emissions, and is based on data from the IEA,^255^ Organisation for Economic Co-operation and Development,^256^ the World Bank,^257^ and WHO, with methods and additional analysis in appendix 5 (pp 164–167).^258^

**Figure 19:**
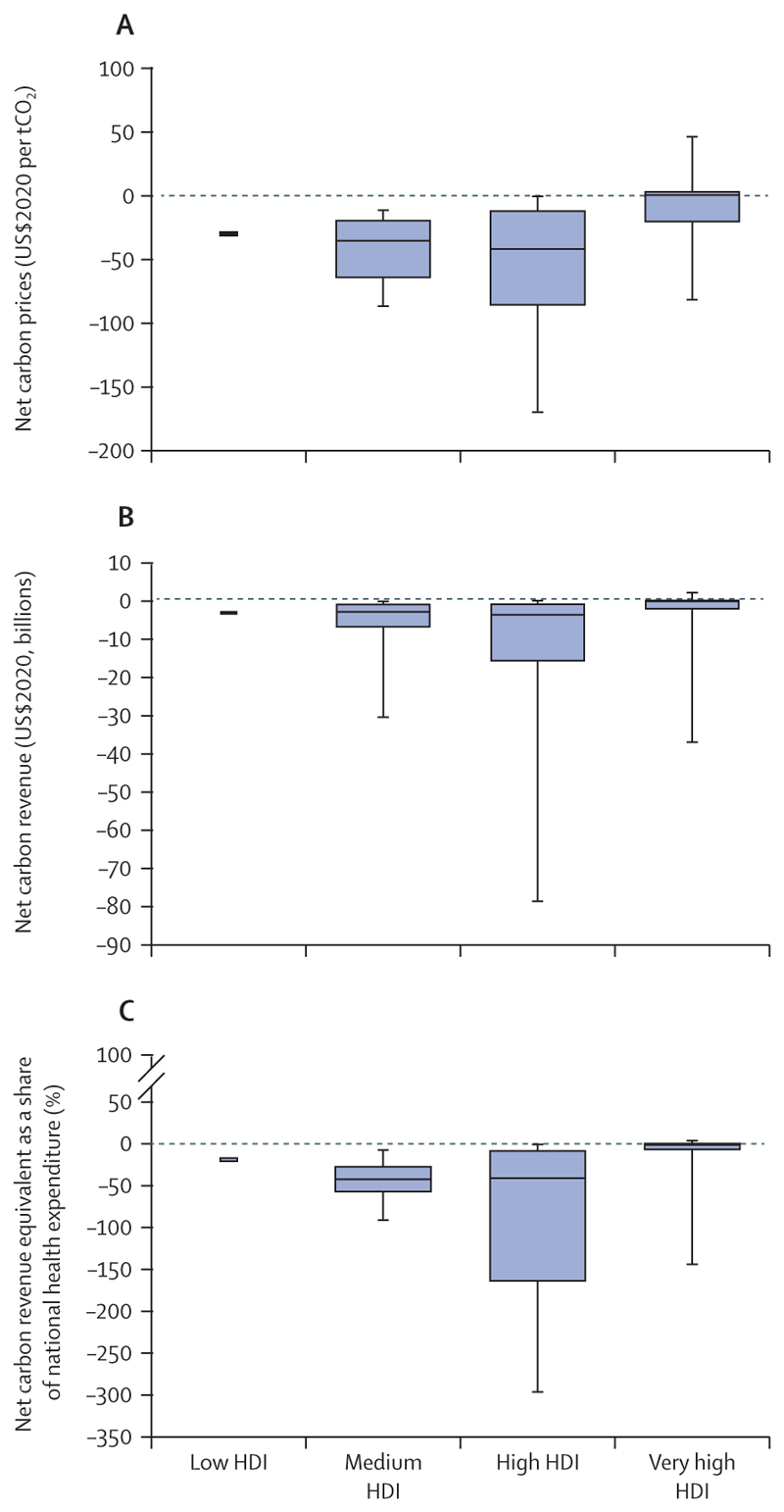
Net carbon prices, net carbon revenues, and net carbon revenue as a share of current national health expenditure across HDI groups (A) Net carbon prices. (B) Net carbon revenues. (C) Net carbon revenue as a share of current national health expenditure. Data from 84 countries in 2018, arranged by 2019 HDI group (low [n=1], medium [n=7], high [n=23], and very high [n=53]). Boxes represent IQR, horizontal lines inside the boxes represent the medians, and the brackets represent the range. HDI=human development index. tCO_2_=tonnes of carbon dioxide.

In 2018, 65 (77%) of the 84 countries analysed had net-negative carbon prices, reflecting an overall subsidising of fossil fuels. The median value of the subsidy in these countries was US$1 billion, with some countries providing net subsidies to fossil fuels in the tens of billions of US dollars each year. 42 countries had a carbon pricing mechanism in place, but only 19 succeeded in discouraging fossil fuels with net-positive carbon prices (all of which were countries with a very high HDI). Nonetheless, most very high HDI countries still had net-negative carbon prices (figure 19). These net subsidies are equivalent to substantial proportions of national health spending in many countries.

With low-income populations vulnerable to energy costs, removing subsidies can be a challenge, but redirecting spending from fossil subsidies to healthcare and health-related services is likely to deliver net benefits to their wellbeing.^259^ Furthermore, international financing mechanisms to support low-income countries in their transition to sustainable energy sources are essential to safeguard all dimensions of human health.^260^

#### Indicator 4.2.5: production-based and consumption-based attribution of CO_*2*_ *and PM*_*2·5*_ *emissions—headline finding: in 2019, 18% of CO*_*2*_ *and 17% of PM*_*2·5*_ *global emissions were embodied in trades between countries of different HDI levels*

The production of goods and services often drives greenhouse gas and PM_2·5_ emissions, thus contributing to impacts on health and wellbeing. Emissions from local production (ie, production-based emissions) occur within the geographical territories through the local production of goods and services. An alternative way of accounting for the burden of pollution is to assign emissions to the country that is the final consumer of the products that are made (ie, consumption-based emissions). A comparison of production-based and consumption-based emissions gives a better understanding of how emissions are embodied in global trade, which is essential to enable better international policy formulation that protects human health in all geographies.

This indicator captures the pollution burden from a country’s local production and from a nation’s domestic final consumption, including the burden embedded in its imports. The indicator uses an environmentally extended multiregional input–output (EE-MRIO) model and the EXIOBASE database to estimate CO_2_ emissions,^261,262^ and the greenhouse gas–air pollution interactions and synergies (GAINS) model to produce a PM_2·5_ emission inventory.^263^ More details on the methods and additional analysis can be found in appendix 5 (pp 168–174).

**Figure 20:**
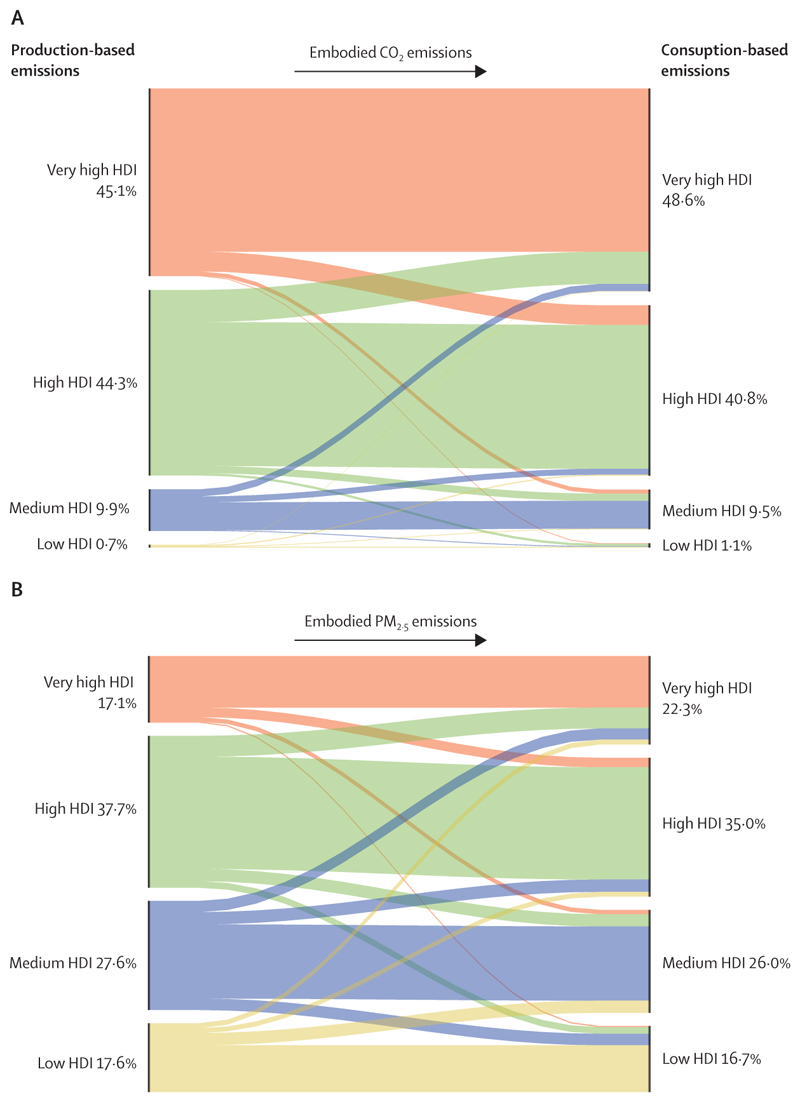
The flows of embodied CO2 and PM_2·5_ emissions among HDI country groups in 2019 HDI=human development index.

In 2019, 18% of the 35·6 Gt world total of CO_2_ and 17% of 37·4 Mt world total of PM_2·5_ global emissions were embodied in trades among countries of different HDI levels (figure 20). The largest contributors to global consumption-based CO_2_ and PM_2·5_ emissions were China (28 % and 18%), the USA (17% and 5%), the EU (10% and 6%), and India (7% and 16%). The USA did the most outsourcing of emissions, with 1·2 Gt (21%) of their 5·9 Gt total CO_2_ and 0·8 Mt (49%) of their 1·7 Mt total PM_2·5_ emissions resulting from its consumption of goods that were produced in other countries. In China, 1·8 Gt (16%) of the 10·8 Gt total CO_2_ and 0·8 Mt (13%) of the 6·8 Mt total PM_2·5_ emissions that occurred within its borders resulted from the local production of goods that were ultimately exported to consumers in other countries.

The very high HDI country group contributed the most production-based (45%) and consumption-based (49%) CO_2_ emissions in 2019. However, the high HDI country group was the biggest contributor to both production-based (38%) and consumption-based (35%) PM_2·5_ emissions. The very high HDI country group was the lowest emitter of PM_2·5_, partly as a result of stricter local air pollution regulations. Importantly, the very high HDI country group was the only group with higher consumption-based emissions than production-based emissions—ie, a high net outsourcing in terms of their consumption-related emissions.

### Conclusion

The impacts of climate change on health are already having substantial economic consequences in different ways across countries of all HDI levels. The economic losses of climate-related extreme events are three times higher in medium HDI countries than they are in very high HDI countries. However, the monetised value of global heat-related deaths is highest in Europe and the greatest costs of premature mortality due to air pollution fall in countries with medium and high HDI levels. WHO’s South-East Asia was the only region with increasing air pollution mortality costs between 2015 and 2019, relative to GDP. Extreme heat can create economic impacts by reducing labour capacity. In this case, people employed in low-wage, outdoor work in low HDI countries are likely to be most affected.

Because of the potentially large and unequally distributed impacts of climate change on human health, incomes, and wellbeing, substantial and sustained investment in a low carbon transition is required. Overall, global investments in coal power continue to decline, although with worrying countertrends in particular countries. Investments in renewables and energy efficiency continue to grow, as do divestments from fossil fuel assets; however, a considerable increase in the pace of change is required.

Both governments and the private sector have crucial roles in bringing about the required transition. Governments across all HDI groups should address fossil fuels subsidies in countries. Although withdrawing energy subsidies is challenging when it affects people on low incomes, other forms of government spending, including on health services, can provide better and more targeted support to decrease inequities and maximise wellbeing. The global trade system means that almost a fifth of CO_2_ and PM_2·5_ emissions occur in the production of goods that are subsequently traded between countries of different HDI levels. This proportion underlines the importance of inclusive global agreements that facilitate cooperation on policies for the reduction of both production and consumption emissions.

As governments begin to invest in recovery from COVID-19, there is a crucial window of opportunity to reduce fossil fuel subsidies, invest more in clean energy, and support a green recovery. Policies and regulations should be developed that greater scrutinise fossil fuel companies and ensure their alignment with a world below 2°C.

## Section 5: public and political engagement

As sections 1–4 make clear, climate change is damaging people’s health and increasing inequities, with the human costs amplified by COVID-19.^28,264,265^ The people least responsible for climate change are most exposed to its impacts, which are “hitting harder and sooner” than climate assessments indicated even a decade ago.^266^ Action at the speed and scale that is needed to meet the ambitions of the Paris Agreement requires public and political engagement, particularly in industrialised countries (where most emissions originate).^267^ This section tracks engagement in health and climate change by media, individuals, scientists, governments, and the corporate sector.

The mainstream media is a major platform for public engagement. Mainstream media is the most widely-used source of information,^268^ shaping public perceptions,^269–271^ and influencing the social media agenda.^272^ Indicator 5.1 tracks coverage of health and climate change in 67 newspapers from 37 countries, including the *People’s Daily* (in its Chinese-language edition, *Renmin Ribao*), which is China’s longest running national newspaper and the official outlet of the Chinese Government.^273,274^ The indicator also includes a content analysis of coverage in India and the USA, focusing on so-called prestige newspapers with influence on the countries’ political and economic elites.^275–277^

Individual engagement (indicator 5.2) is tracked through individuals’ searches on Wikipedia—the online information source with a wider reach and coverage than traditional encyclopaedias.^278–280^ The third indicator (Indicator 5.3) tracks engagement in peer-reviewed journals—the primary source of scientific evidence for the media, government, and the public.^281^

Government engagement (indicator 5.4) is tracked by statements made by national leaders at the UN General Assembly (the policy making body of the UN). The annual meeting opens with the General Debate, in which heads of government, or their high-ranking representatives, address the global community on issues they consider important.^282,283^ Indicator 5.4 also considers engagement with health in the enhanced nationally determined contributions (NDCs), submitted in compliance with the 2015 Paris Agreement.^284–286^ Panel 6 compares health engagement in the initial and enhanced set of NDCs held on the UNFCCC NDC registry on April 1, 2021.

Action by the corporate sector will be decisive in moving societies away from dependence on fossil fuels.^292–294^ Indicator 5.5 tracks engagement in health and climate change by companies within the UN Global Compact—the world’s biggest corporate sustainability initiative.^295^ Companies commit to shared principles of sustainable behaviour and submit annual reports on progress.

With increasing acknowledgment of the need to recognise and investigate gender inequities in the representation, communication, and governance of climate change,^296–299^ engagement with gender is incorporated where appropriate. Engagement with health, climate change, COVID-19, and analyses by WHO region and HDI country group are also included. Details of data sources and methods for all indicators are provided in appendix 5 (pp 175–267), along with additional analyses.

### Indicator 5.1: media coverage of health and climate change

*Headline finding: in 2020, the upward trend in coverage of health and climate change continued but did not match the increase seen in 2019; in 2020, most of the coverage of health and climate change referred to COVID-19*

Newspapers provide an important forum for public engagement. Newspapers shape public understanding of climate change, both through their influence on their readers and the wider political agenda.^270,300^ This indicator tracks coverage of health and climate change from 2007, which is the year before the WHO World Health Assembly made a multilateral commitment to protect people’s health from climate change.^301^ The indicator includes 66 newspapers spanning 36 countries and four languages, together with an additional analysis of China’s *People’s Daily*. The indicator also examines the content of 2020 coverage in newspapers in India and the USA. Methods and additional analysis are provided in appendix 5 (pp 175–198)

Across the 36 countries, the upward trend in newspaper coverage of health and climate change continued, reaching 11 371 articles in 2020. However, the rate of increase was lower than that of 2019, with a 6% increase from 2019 to 2020 compared with a 96% increase from 2018 to 2019. As in 2019, coverage was greatest in the WHO America and Europe regions and lowest in the African region.

Engagement with gender and COVID-19 was examined in English language newspapers across 23 countries. Although the proportion of articles on climate change and health referring to gender increased from 97 (2%) of 6044 articles in 2007 to 573 (6%) of 10 092 in 2020, gender remains marginal to the representation of health and climate change in the mainstream press. In 2020, more than 60% (6238) of the 11 371 articles referring to health and climate change also referred to COVID-19, and it was more than 80% in April and May, 2020.

In China’s *People’s Daily*, the sparse coverage of health and climate change, noted in previous *Lancet* Countdown reports, was again evident in 2020. Of the 1106 articles discussing climate change, 2% were related to human health. Across the 2008–20 period, no articles related to health and climate change engaged with gender issues. In 2020, no articles discussed the relationships between climate change and COVID-19 or how they influenced health together.

Analysis of the content of coverage of health and climate change focused on India (medium HDI) and the USA (very high HDI). The selected newspapers, the *Times of India, Hindustan Times, New York Times*, and *Washington Post*, form part of the so-called prestige press, seen to exercise influence on political and economic elites and the wider policy agenda.^277,302^

One set of themes in all prestige newspapers was the health impacts of climate-related hazards, including heatwaves and wildfires. For example, the *New York Times* (June 18, 2020), noted that “people with health issues, older people and young children are especially susceptible to the effects of extreme heat. It’s a threat that grows as climate change continues.”^303^ Another set of themes was related to the spread of infectious disease, including COVID-19. For example, the *Hindustan Times* (March 15, 2020) reported the impact of climate change on the capacity to control infectious diseases, and that “a rise in zoonotic diseases—Nipah, Ebola, Zika, Coronavirus to name a few in recent decades—is driven by biodiversity loss and climate change”.^304^ As this last comment indicates, climate change and environmental change are often linked together; scientific reports (including the *Lancet* Countdown) are cited as evidence that “we are close to running out of time—approaching a point of no return for human health, which depends on planetary health” (*New York Times*; April 28, 2020).^305^

### Indicator 5.2: individual engagement in health and climate change

*Headline finding: individual information seeking about health and climate change decreased overall by 15% from 2019 to 2020; spikes in engagement in mid-2020 were almost exclusively due to interest in pandemic-related content*

Individual engagement in climate change and health is tracked through the digital footprint of users of the online encyclopaedia, Wikipedia. Wikipedia has outpaced traditional encyclopaedias in terms of reach, coverage, and comprehensiveness and is one of the most-visited websites worldwide.^278,306,307^ This analysis is based on the English-language Wikipedia, which represents around 50% of global traffic to all Wikipedia language editions.^308,309^

This indicator focuses on so-called clickstream activity, in which an individual clicks between an article on health and climate change (or vice versa). Because clickstream activity captures only pairs of sequential visits, for the 2021 report, the set of articles was extended to include a wider range of health and climate change articles than in the 2020 report. In 2020, as in previous years, individuals seldom moved between health and climate change; instead, click activity was predominantly within the set of articles on health or climate change.

**Figure 21:**
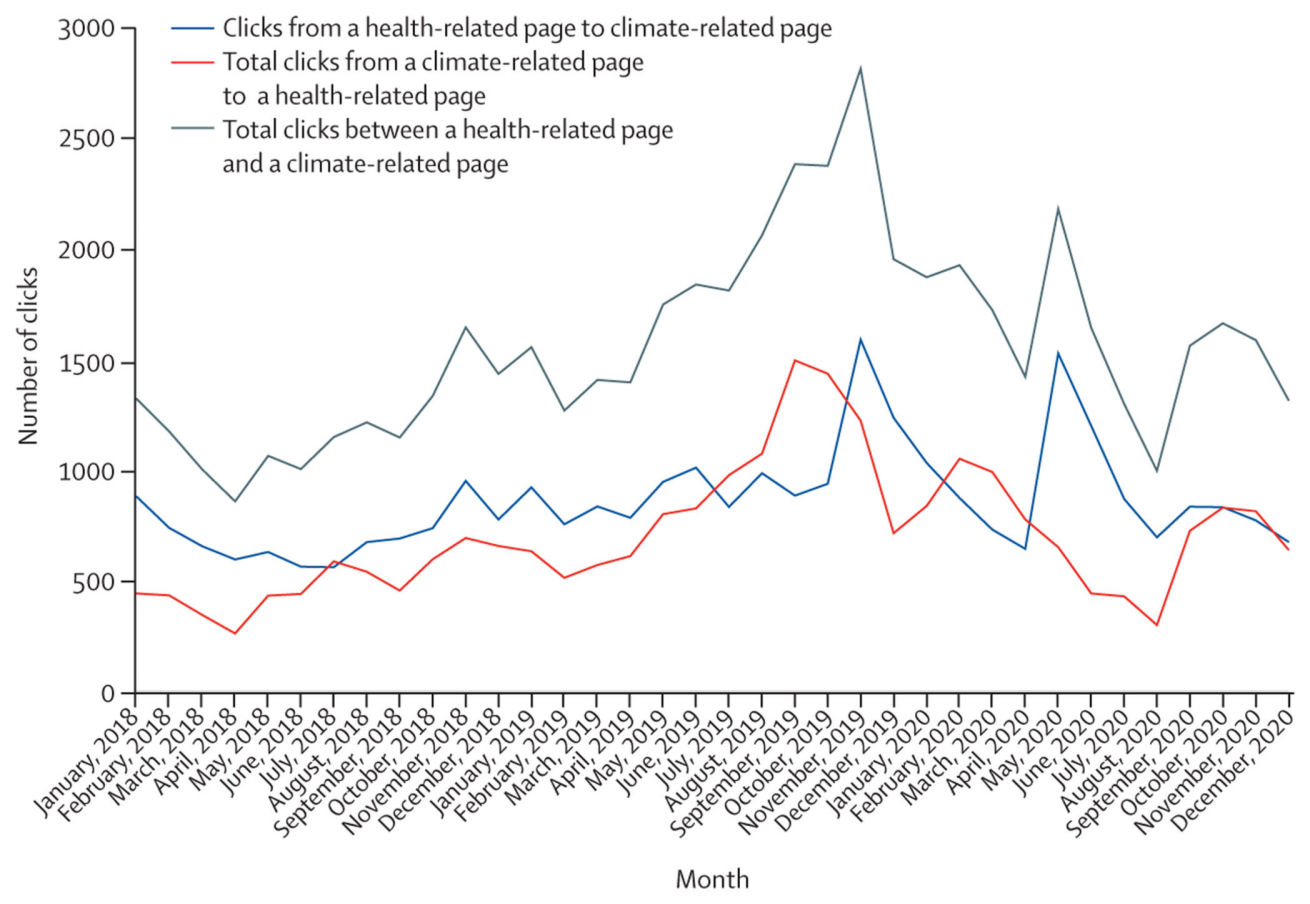
Aggregate monthly clicks between a health-related article and a climate-related article in Wikipedia, 2018—20

Figure 21 tracks click activity from 2018 to 2020, looking separately at the volume generated by clicks on a climate-related link in a health-related page, health-related link in a climate-related page, and the sum of both. Overall, the numbers of clicks are very low, confirming that engagement in either climate change or health rarely triggers engagement in the other topic. Furthermore, total clickstream activity between health-related and climate-related pages fell in 2020 by 15%, reversing the upward trend evident in 2019. When clicks to an article relating to COVID-19 are excluded, the downward trend in 2020 becomes even more pronounced. The spike in co-clicks in mid-2020 was almost exclusively due to interest in pandemic-related content, which then sparked interest in climate change, whereas the rise during September and October, 2020, was generated by an initial interest in climate change.

### Indicator 5.3: coverage of health and climate change in scientific journals

*Headline finding: original research on health and climate change increased 11-fold between 2007 and 2020, driven primarily by scientists in countries in the very high HDI group; the number of articles on health and climate change that addressed gender remained low; in 2020, 7% of health and climate change articles referred to COVID-19*

Scientific evidence is a key resource for media outlets, individuals, and governments, and has a crucial role shaping public and political engagement in health and climate change.^280,310^ This indicator is based on searches in OVID MEDLINE and OVID Embase and uses references to health and climate change in article titles and abstracts. Methods and additional analyses are provided in appendix 5 (pp 221–234).

The upward trend in scientific engagement in health and climate change noted in previous *Lancet* Countdown reports has been maintained,^53,311^ with the number of articles on health and climate change increasing by 28% between 2019 and 2020 to reach its highest recorded level of 858 articles. This trend is driven by the rapid increase in original research (ie, primary studies and systematic reviews), which increased by 32% between 2019 and 2020. Research-related articles (eg, evidence reviews, editorials, and letters) also increased, but at a lower rate.

**Figure 22:**
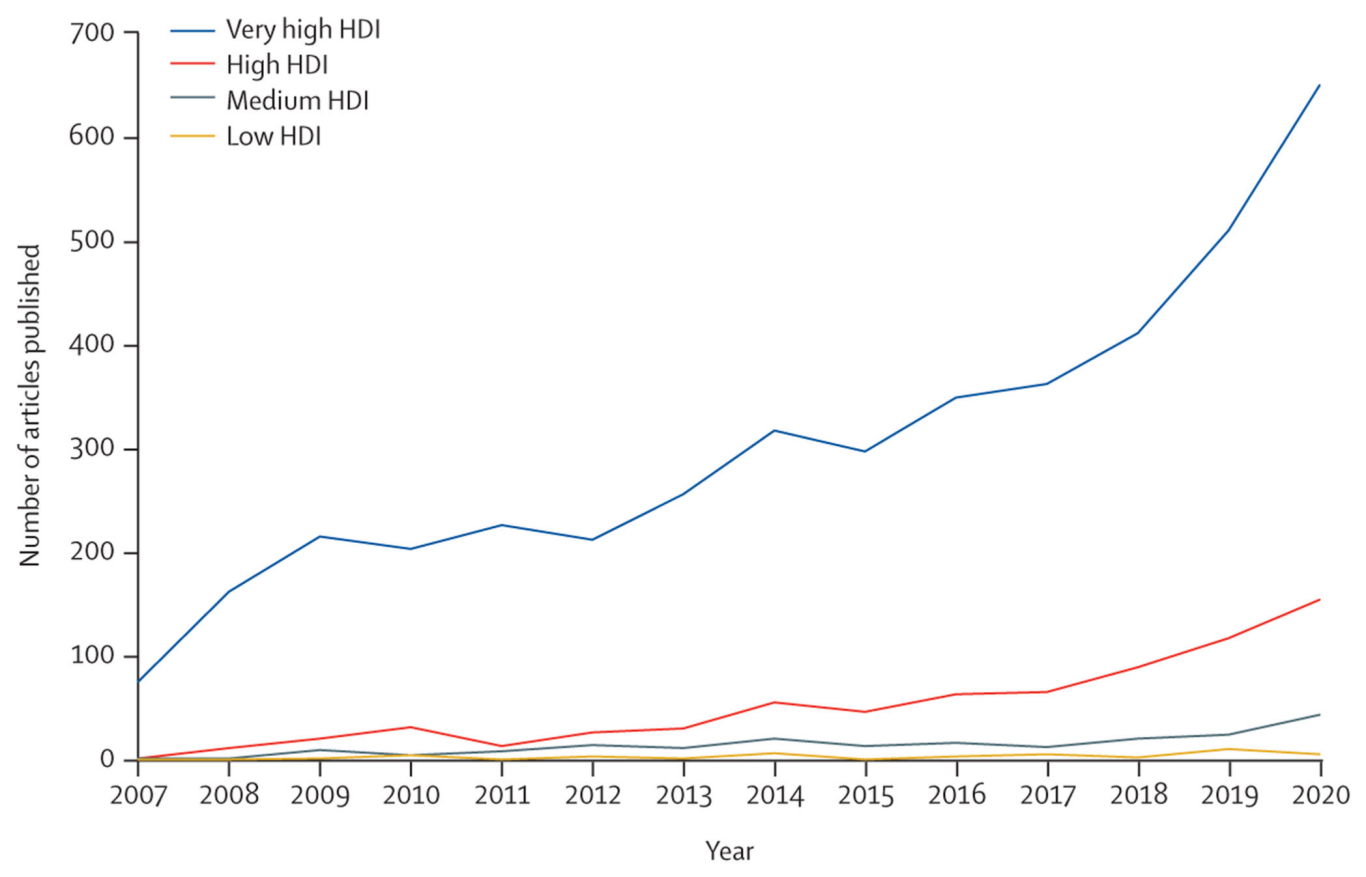
Scientific journal articles relating to health and climate change by 2019 HDI group of the main country of affiliation of the first author, 2007–20

Increasing scientific engagement in health and climate change is driven by very high HDI countries (figure 22); 76% of the total output in 2020 was led by researchers in this group. In contrast, scientists in low HDI countries were lead authors of just 1% of journal articles.

In 2007, less than 2% of health and climate change articles engaged with gender in some way, and in 2020, the proportion was 6%. In 2020, only 7% of the articles on heath and climate change addressed COVID-19, suggesting this rise in scientific research in health and climate change is independent of the concurrent global health crisis. Articles engaging with gender and with COVID-19 were predominantly led by scientists in the very high HDI countries.

### Indicator 5.4: government engagement in health and climate change

*Headline finding: in 2020, 47% of government leaders engaged with the health dimensions of climate change in their statements at the UN General Debate, which is more than double the proportion in 2019; this increase was linked to engagement with the COVID-19 pandemic*

Government leadership, backed by strong near-term policies, is required if the increase in global temperature is to be halted.^172^ This indicator examines government engagement with health and climate change in the UN General Debate. Engagement with health in commitments to emissions reduction made by governments under the 2015 Paris Agreement is also considered in panel 6.

The UN General Debate opens each new session of the UN General Assembly. It provides all UN member states with an opportunity to address the global community on priorities for action. Among many global challenges, including economic recession and social conflict, the indicator captures whether government leaders draw attention to health and climate change. Analysis in this indicator is based on the application of a keyword search in the UN General Debate corpus with natural language processing.^312,313^ 8288 statements made between 1970 and 2020 were analysed.

**Figure 23:**
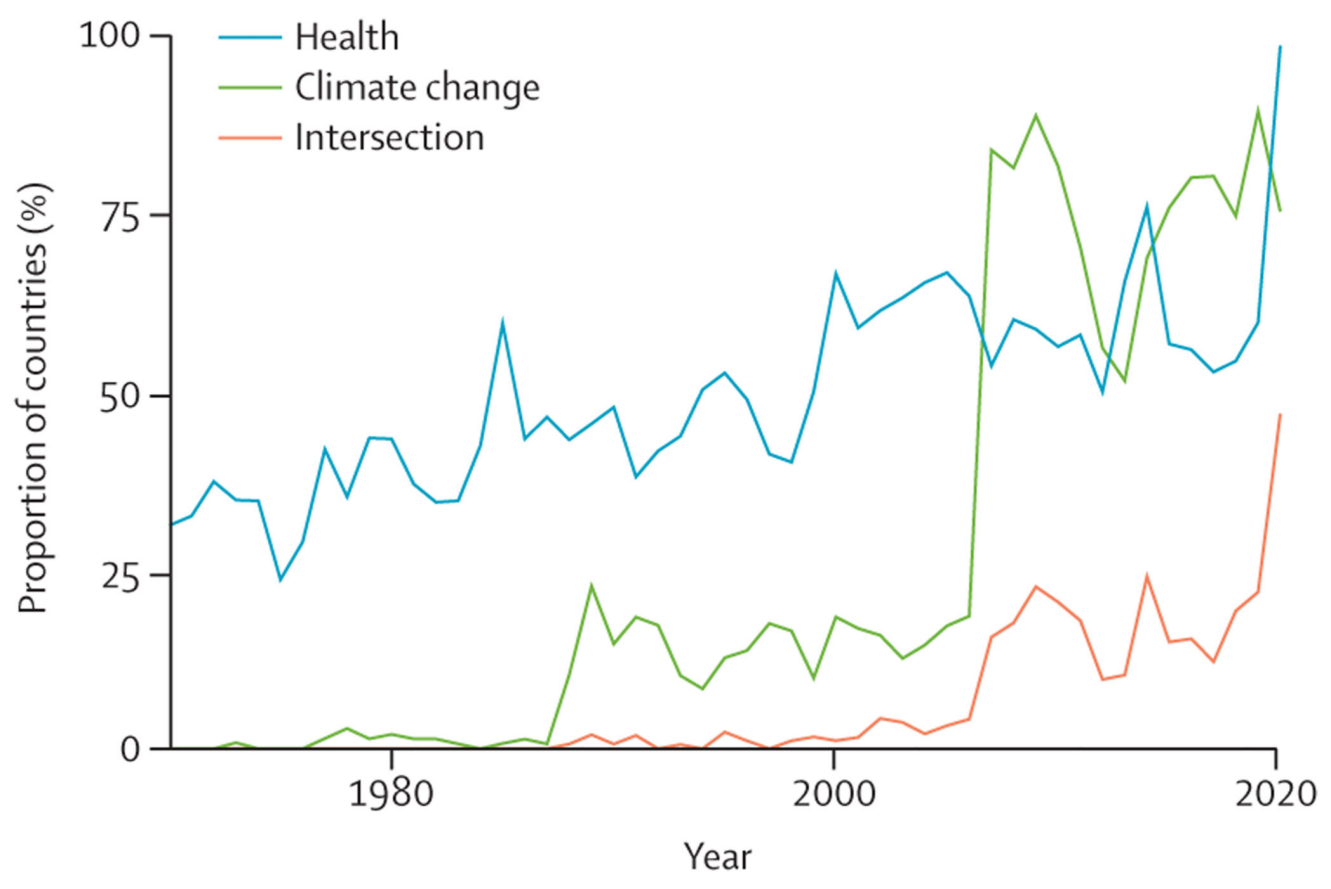
Proportion of countries referring to climate change, health, and the intersection between climate change and health in their UN General Debate statements, 1970–2020

Figure 23 shows the proportion of countries referring to health and climate change in their UN General Debate statements between 1970 and 2020. In 2020, the proportion of countries engaging with the health dimensions of climate change was the highest on record, increasing from 43 (22%) of 195 in 2019 to 91 (47%) of 193 countries in 2020. Additionally, and for the first time in the UN General Debate, every member state referred to health in their 2020 address—a reflection of the ongoing global pandemic.

The increased engagement in health and climate change is linked to the discussion of the COVID-19 pandemic, represented by government leaders as both a threat and an opportunity. The pandemic highlights “the vulnerabilities of our societies [to]…the next global disaster…[like] climate change” (Austria).^314^ This engagement also presents an opportunity to tackle the climate crisis, with Fiji stating “our recovery from this pandemic must mark a transition to a decarbonized, climate-resilient economic system”.^315^

Engagement in health and climate change continues to be led by countries in the low HDI group and, in particular, by the SIDS.^285,286^ For the SIDS, COVID-19 has amplified the risks of climate change, and the Government of St Lucia has stated that “our unique circumstances and consequent vulnerabilities have left us exposed to the ravages of the twin crises of the pandemic and climate change”.^314^ In 2020, 30 (81%) of the 37 SIDS discussed health and climate change in the 2020 UN General Debate. However, there was a greater engagement among very high HDI countries in 2020 than in previous years. A key issue is whether this pandemic-related increase in engagement among very high HDI countries will be maintained in future years.

### Indicator 5.5: corporate sector engagement in health and climate change

*Headline finding: in 2020, engagement in health and climate change increased to its highest level among companies in the UN Global Compact; 38% of companies referred to the health dimensions of climate change in their 2020 progress reports*

The indicator tracks engagement in health and climate change among companies signed up to the UN Global Compact, which was established to promote corporate social and environmental responsibility. However, the effectiveness of the UN Global Compact has been critiqued with the suggestion that membership could be a form of so-called greenwashing and bluewashing for some companies.^316^ The Compact represents more than 12 000 companies from 160 countries, with each submitting an annual communication on progress (Gobal Compact Communication of Progress) against a set of social and environmental principles.

This indicator is based on the application of a keyword search in the text corpus of 17 984 GCCOP reports submitted in English between 2011 and 2020. In the 2019 and 2020 *Lancet* Countdown reports, the focus was on the health-care sector. This report considers corporate engagement across all sectors.

**Figure 24:**
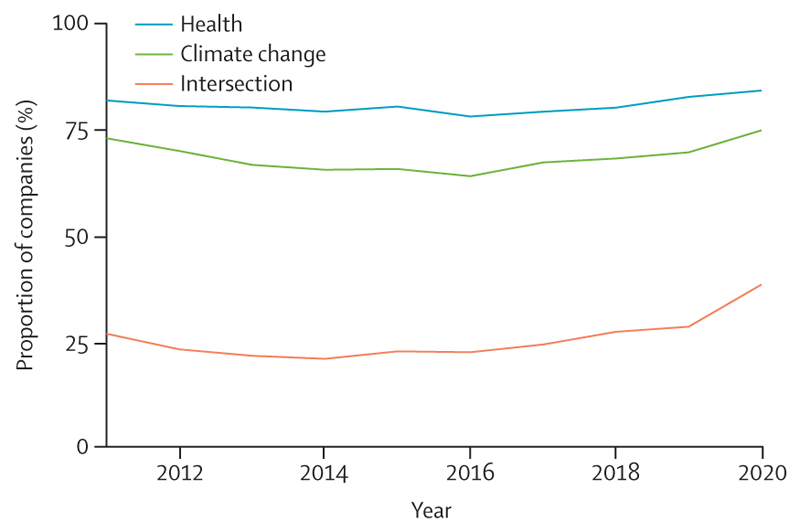
Proportion of companies referring to climate change, health, and the intersection of climate change and health in their UN Global Compact *Communication on Progress* reports, 2011–20

Figure 24 shows engagement in health and climate change in annual GCCOP reports published from 2011 to 2020. The large majority of reports refer to health (1742 [84%] of 2029 reports in 2020) and climate change (1547 [75%] reports in 2020) as separate topics. Only a minority of reports referred to the health dimensions of climate change (791 [38%] in 2020). However, this minority represents a large increase from 2014, the low point of engagement, when only 21% of corporations referred to the intersection between climate change and health. Three sectors stand out for their high levels of engagement in health and climate change—namely, food and drug retailers, oil and gas producers, and alternative energy. In 2020, more than 70% of corporations in these sectors made reference to health and climate change. However, in the health-care sector, this proportion was only 37%.

Additional analyses examined references to gender in the GCCOP reports engaging with health and climate change. Only a minority of reports that engaged with health and climate change referred to gender. However, this proportion increased from 5% in 2014 to 19% in 2019. In 2020, gender engagement fell to 13% (appendix 5 pp 252–267).

### Conclusion

Public and political engagement is essential if the ambitions of the Paris Agreement are to be reached.^172^Section 5 has focused on five areas of engagement—namely, the media, the public, the scientific community, national governments, and the corporate sector. Three conclusions can be drawn.

First, health and climate change are increasingly addressed together. The trend is particularly pronounced for indicators relating to the media, science, government, and the corporate sector. In all these areas, engagement with health and climate change reached its highest recorded level in 2020. Gender is rarely integrated into engagement within the health and climate change nexus, although there is increased recognition in countries’ enhanced NDCs.

Second, the COVID-19 pandemic appears to be a major driver of engagement in 2020. For example, more than half of newspaper coverage of health and climate change was linked to COVID-19, and individual engagement in health and climate change was largely sustained by searches for articles related to COVID-19. Government engagement in the health dimensions of climate change was similarly underpinned by engagement in the pandemic. It is not known whether the heightened engagement in health and climate change will be maintained if, and when, pandemic-related crises are contained.

Third, social inequities are deeply etched into public and political engagement. In the media and science, coverage of health and climate change engagement is greatest in the countries with a very high HDI (ie, the countries that are exerting the greatest pressure on the planet but that are also the most protected from the health impacts of climate change). Countries with medium and low HDIs have much smaller carbon and environmental footprints than countries with very high HDI; however, they are shouldering the immediate burden of climate change and are far less represented in the scientific literature. As in previous years, the SIDS are leading global engagement with the health impacts of climate change at the UN General Debate. It is not known what is required for the leadership of SIDS to be matched by the countries and communities contributing most to climate change.

## Conclusion: the 2021 report of the *Lancet* Countdown

The 2021 report of the *Lancet* Countdown finds a world overwhelmed by an ongoing global health crisis, which has made little progress to protect its population from the simultaneously aggravated health impacts of climate change. The inequities of these impacts and the response, including those of gender, are brought into sharp focus within each of the indicators presented. This exposes the urgent need for the collection of standardised data to capture inequities and vulnerabilities (panel 2).

Climate-sensitive infectious diseases are of increasing global concern and the environmental suitability for the transmission of all infectious diseases is increasing (indicator 1.3.1). For non-*cholerae Vibrio* bacteria, the environmental suitability for transmission in northern latitudes has increased by 56% since the 1980s. The number of months suitable for malaria transmission has increased by 39% in highland areas of the low HDI country group and, during the past 5 years, the environmental suitability for the transmission of emerging arboviruses (eg, dengue, chikungunya, and Zika) was between 7% and 13% higher than it was in the 1950s.

The high temperatures in 2020, a year that tied with 2016 as the hottest year on record, resulted in extreme heat-related health impacts, affecting the emotional and physical wellbeing of populations around the world (indicators 1.1.1–1.1.6). These higher temperatures and altered weather patterns are also leading to more frequent extreme weather events and increased wildfire exposure (indicators 1.2.1, 1.2.2, and 1.2.3) and are putting years of progress on food and water security at risk in many parts of the world. The 5 years with the greatest area of the world’s surface affected by droughts have all occurred between 2015 and 2020 (indicator 1.2.2), the yield potential of all major staple crops continues to fall as a result of the rising temperatures (indicator 1.4.1), and 79% of all potential work hours lost to extreme heat in low HDI countries occurred in the agricultural sector in 2020 (indicator 1.1.4).

However, measures to curb emissions have been grossly inadequate. Emissions are declining too slowly or heading in the wrong direction in the highest emitting sectors (indicators 3.1, 3.4, and 3.5.1). This delay in progress is contributing to millions of deaths each year due to exposure to indoor and ambient PM_2·5_ pollution and due to high-carbon, unhealthy diets (indicators 3.2, 3.3, and 3.5.2). Importantly, these effects manifest differently between HDI country groups and genders, underscoring profound inequities.

Despite years of scientific reporting on the impacts of climate change, efforts to build resilience have been slow and unequal, with countries with low levels of HDI being the least prepared to respond to the changing health profile of climate change and funding remaining a consistent challenge (indicators 2.1.1, 2.3.1, and 2.4). At the same time, 65 of 84 countries reviewed continue to provide subsidies for fossil fuels that outweigh any revenue received from carbon pricing instruments. The resulting net carbon subsidies are, in many cases, equivalent to substantial proportions of countries’ national health budgets (indicator 4.2.4).

Governments with the fiscal capacity have responded to the COVID-19 pandemic with massive spending packages, to cushion the impacts of the crisis and start to bring about economic recovery. But as the world approaches COP26, the response to climate change, and commensurate investment, remains inadequate. The opportunity for the green recovery is in danger of being missed. A fossil-fuel driven recovery, although potentially meeting narrow and near-term economic targets, could push the world irrevocably off course for the ambitions of the Paris Agreement, with enormous costs to human health.

With government leaders more engaged with the health dimensions of climate change than ever before (indicator 5.4), countries across the globe should pursue low-carbon economic recovery pathways, implementing policies that reduce inequities and improve human health. The *Lancet* Countdown indicators show the evidence to support the urgency and opportunity of this transition, and that no people are safe until everyone is safe.

## Supplementary Material

Chinese translation of the Executive Summary

French translation of the Executive Summary

German translation of the Executive Summary

Spanish translation of the Executive Summary

Supplementary appendix 5

## Panel 1 Working group indicator

### Climate change impacts, exposures, and vulnerabilities

1.1 : health and heat
  1.1.1.: vulnerability to extremes of heat
  1.1.2 : exposure of vulnerable populations to heatwaves
  1.1.3 : heat and physical activity
  1.1.4 : change in labour capacity
  1.1.5 : heat and sentiment
  1.1.6 : heat-related mortality
1.2 : health and extreme weather events
  1.2.1 : wildfires
  1.2.2 : drought
  1.2.3 : lethality of extreme weather events
1.3 : climate-sensitive infectious diseases
  1.3.1 : climate suitability for infectious disease transmission
  1.3.2 : vulnerability to mosquito-borne diseases
1.4 : food security and undernutrition
  1.4.1 : terrestrial food security and undernutrition
  1.4.2 : marine food security and undernutrition
1.5 : migration, displacement, and rising sea levels

### Adaptation, planning, and resilience for health

2.1 : adaptation planning and assessment
  2.1.1 : national adaptation plans for health
  2.1.2 : national assessments of climate change impacts, vulnerability, and adaptation for health
  2.1.3 : city-level climate change risk assessments
2.2 : climate information services for health
2.3 : adaptation delivery and implementation
  2.3.1 : detection, preparedness, and response to health emergencies
  2.3.2 : air conditioning: benefits and harms
  2.3.3 : urban green space
2.4 : health adaptation-related global funding and financial transactions

### Mitigation actions and health co-benefits

3.1 : energy system and health
3.2 : clean household energy
3.3 : premature mortality from ambient air pollution by sector
3.4 : sustainable and healthy transport
3.5 : food, agriculture, and health
  3.5.1 : emissions from agricultural production and consumption
  3.5.2 : diet and health co-benefits
3.6 : mitigation in the healthcare sector

### Economics and finance

4.1 : the economic impact of climate change and its mitigation
  4.1.1: economic losses due to climate-related extreme events
  4.1.2 : costs of heat-related mortality
  4.1.3 : loss of earnings from heat-related labour capacity reduction
  4.1.4 : costs of the health impacts of air pollution
4.2 : the economics of the transition to zero-carbon economies
  4.2.1 : coal and clean energy investment
  4.2.2 : employment in low-carbon and high-carbon industries
  4.2.3 : funds divested from fossil fuels
  4.2.4 : net value of fossil fuel subsidies and carbon prices
  4.2.5 : production-based and consumption-based attribution of CO_2_ and PM_2·5_ emissions

### Public and political engagement

5.1 : media coverage of health and climate change
5.2 : individual engagement in health and climate change
5.3 : coverage of health and climate change in scientific journals
5.4 : government engagement in health and climate change
5.5 : corporate sector engagement in health and climate change

## Box

***Panel 2:* Gender, health, and climate change**

The health impacts of climate change are both underpinned and amplified by gender norms and gender inequities, with numerous examples cited throughout this report.^29^ Gender also influences who sets the agenda and drives responses to climate change. Evidence shows that greater representation of women in parliament is associated with stronger climate change policies.^30–32^ However, only 41 (21%) of 196 heads of delegation to the UN Framework Convention on Climate Change (UNFCCC) Conference of Parties in 2019 were women, and women headed just 29% of national delegations to the UNFCCC intersessional in June 2019. Additionally, of the 1000 scholars listed by Reuters as the most influential on climate change, only 122 were women.^33^

There is an urgent need for gender-sensitive responses to the health dimensions of climate change. These responses are underpinned by the collection and reporting of data that is sufficiently disaggregated, granular, and intersectional to reveal local inequities—eg, data disaggregated not only by gender but also by geography, age, ethnicity, class, and other markers of marginalisation and vulnerability.^34–38^ However, in many cases, a scarcity of standardised, gender-disaggregated data hampers these efforts.^39–42^ It is the very social structures that shape how gender is perceived and prioritised that undermine progress—eg, cultural norms often translate into weak political and financial support and limit the capacity of researchers to engage with gender inequities.^40,43^ Only 6% of all scientific articles covering climate change and health in 2020 considered gender (indicator 5.3), and, despite a workstream established for this purpose, only 6 of the 44 indicators in the 2021 report of the *Lancet* Countdown provide data by sex or gender.

Starting to reverse this, the UN Entity for Gender Equality and the Empowerment of Women (UN Women) is leading global efforts to increase the availability of information on gender through its flagship programme, Making Every Woman and Girl Count. Through this programme, UN Women supports countries with the development of priority indicators to expose and record gender inequities (both through indicator selection and data collection).^44^ A model questionnaire has been developed for this purpose, and several countries, including Bangladesh, Mongolia, and several Pacific island countries, have either begun (or are currently preparing for) their rollout. With the purpose of helping countries understand the connections between the environment and gender equality, the programme also supports data reprocessing and the integration of geospatial information with demographic and health surveys. The importance of this work is already materialising. Preliminary analysis shows the accentuation of gender inequities as a result of weather events, including drought episodes driving spikes in child marriage for girls in almost all Asian countries analysed.

Gender, as a social construction, affects everyone in society.^28,45–48^ A gender-sensitive response to climate change would generate benefits for the whole of society. Ensuring gender is represented in national statistical strategies and regular data collection processes will expose the true dimensions of the challenge. Ensuring this representation, along with more diverse leadership, will inform and drive a commensurate response.

## Box

***Panel 3:* The urban heat island and the impact of cool roofs**

As a result of human activity and the urban fabric, cities tend to be hotter than surrounding rural or suburban areas; an effect known as the urban heat island (UHI) effect.

With increasing temperatures and urbanisation, the demand for cooling mechanisms is on the rise. Although offering protection from life-threatening extreme heat exposure, the use of air conditioning contributes to climate change through its energy consumption and its leakage of hydrofluorocarbons that act as powerful greenhouse gases; contributes to the intensification of the UHI effect through its waste heat emissions; and contributes to increasing peak electricity demand and urban air pollution (see indicator 2.3.2).^147–149^ Furthermore, the high costs of air conditioning are amplifying the energy poverty gap.^149,150^ The development of sustainable and affordable cooling alternatives is, therefore, crucial to protect the health of urban populations and keep the world on track to meet the Paris Agreement goals.

This case study explores the use of so-called cool (ie, reflective) roofs as sustainable cooling mechanisms, ranging from specially designed roofing materials to affordable alternatives, such as light-coloured paint. Focusing on Birmingham, UK, and the West Midlands region of the UK, urban air temperatures were simulated at 1 × 1 km horizontal resolution by combining detailed land use data with a building energy parameterisation scheme in a regional climate model.^151^ To estimate the effect of the UHI, temperatures were compared with those from a simulated counterfactual scenario, with urban surfaces replaced by rural surfaces.

The UHI intensity was on average around 3°C during summer and up to 9°C during heatwaves in this region. The overheating resulting from the UHI was estimated to contribute to approximately 40% of heat-related deaths during summer seasons and up to 50% during heatwaves.^152–154^ Spatial analysis revealed that the most underserved population groups were particularly exposed to urban heat.^154^

Simulations introducing reflective surfaces found that cool roofs could reduce maximum daytime air temperatures by 0·5°C on average and up to 3°C during heatwaves. This cooling has the potential to reduce heat-related mortality due to the UHI effect by 18% during a summer season and 23% during a heatwave.^155^ Considering this assessment was done in a country with a cool climate, the impact of cool roofs might be even greater if applied in warmer parts of the world. Although the UHI effect can reduce cold-related mortality by roughly 15% in the winter, cool roofs were shown to have negligible effects in winter months, suggesting they would not contribute to increased mortality in the winter.^156,157^

Because roofs can affect other factors, such as precipitation, their use should be assessed on a case-by-case basis.^158^ However, with a net annual benefit on temperature-related mortality, the adoption of cool roofs in the face of a warming world could provide a low-carbon cooling alternative, with health benefits to the whole urban population.

## Box

***Panel 4:* Recovering from COVID-19: stimulus measures for a sustainable economy**

The COVID-19 pandemic, and measures to tackle it, triggered a global recession of a depth only exceeded in the past 150 years by two world wars and the Great Depression of the 1930s.^174^ Governments with the fiscal capacity have responded with massive spending packages. By the end of 2020, the world’s 50 largest economies had committed US$14·6 trillion in fiscal measures (many times higher than the value of global stimulus measures after the 2008–09 financial crisis). Although $12·7 trillion (87%) of the $14·6 trillion was designed to prevent an even deeper health and economic crisis, rather than encourage recovery,^175^ promoting recovery will become the main objective as time goes by and additional measures are announced.

How these measures are designed and targeted will determine whether this spending entrenches existing technical, economic, and social structures and systems, or promotes those that are more sustainable, healthy, and equitable. Evidence from stimulus measures introduced after the 2008–09 financial crisis shows that so-called green stimulus measures often have advantages over so-called brown or colourless measures.^176^

So far, the signs of the current recovery commitments are not encouraging. Of the $1·9 trillion directed towards recovery by the end of 2020, just 18% is expected to reduce greenhouse gas emissions (or 2·5% of the value of all fiscal measures), and the overall impact of this investment on air pollution and natural capital—eg, through the expansion of road transport and defence services in particular—is likely to be negative. Just a few nations have positive measures, particularly countries in Europe,^175^ although measures announced so far in 2021 indicate some movement towards greater consideration of sustainability in other countries.^177,178^ However, despite the global CO_2_ emissions dropping by a record 6% in 2020, they have rebounded quickly, and global CO_2_ emissions in December, 2020 were about 2% higher than in December, 2019.^179^ Therefore, the trillions of dollars for stimulus measures that are yet to be announced must be oriented toward a green and healthy recovery.

In May, 2020, WHO published six prescriptions for a healthy and green recovery: protect and preserve the source of human health (ie, nature); invest in essential services, from water and sanitation to clean energy and health-care facilities; ensure a quick, healthy energy transition; promote healthy, sustainable food systems; build healthy, liveable cities; and stop the use of taxes to fund pollution (particularly through fossil fuel subsidies).^180^ If governments are serious about their commitments under the Paris Agreement and Sustainable Development Goals, they should take note of these priorities, plan ahead, and learn from their own previous experiences and from those generated elsewhere to implement well-designed and context-appropriate policy. Where necessary, multilateral institutions, processes, and instruments should be galvanised in support of a global recovery that is both sustainable and equitable.^175^

## Box

***Panel 5*: Compatibility of fossil fuel company strategies with the trajectories of well below 2°C**

Globally, CO_2_ from the combustion of fossil fuels represents 65% of total greenhouse gas emissions.^172^ In the 2015 Paris Agreement, countries agreed to reduce their emissions to keep global warming to well below 2°C. The carbon budget for a 66% probability of limiting global warming to 1·5°C by the end of the century has been estimated at 420 GtCO_2_.^14^ However, the potential CO_2_ emissions from reserves held by the 200 largest public fossil fuel companies are at least 1541 GtCO_2_, ^231^ and the carbon contained in global resources of fossil fuels is estimated at about 11000 GtCO_2,_^232^well beyond the maximum that can be used if the world is to meet the Paris Agreement goals. Roughly 30% of oil reserves, 50% of gas reserves, and more than 80% of coal reserves worldwide should remain unused to keep global warming below 2°C,^232^ representing stranded assets and useable carbon.^178,233^ Future energy system scenarios with strict carbon constraints, low fossil fuel demand, high capital costs projects, and carbonin-tensive reserves increase the risk of stranding assets,^234^ with considerable financial consequences for their owners and industry stakeholders.^235^

Although the fossil fuel industry has begun to acknowledge that the energy system is transitioning away from unabated oil, gas, and coal, countries’ fossil fuel production plans until 2030 could exceed emissions consistent with limiting warming to 2°C by 50% and by 120% in relation to 1·5°C.^236^ Companies have diverging business strategies,^237^ with strategies falling short of what is required to mitigate transition risks. Although an increasing number of oil and gas companies are announcing net-zero commitments, for these to be consistent with climate ambitions they should be framed on their total emissions rather than on their emission intensities, consider scope 1, 2, and 3 emissions of the greenhouse gas protocol,^238^ and account for activities on the basis of a company’s full equity share.^82,239^ Those companies who better understand systemic risks, stress-test potential scenarios, and develop business strategies with interim targets and investments that align adequately with the targets of well below 2°C (and preferably 1·5°C) are likely to become more resilient during the coming years as climate-risk scrutiny from investors and financial regulators increases. the generation of new jobs in low-carbon industries and accelerate progress towards the Paris Agreement goals and the SDGs, yielding health gains both by preventing the health impacts of climate change, and by maximising the health co-benefits directly associated with climate actions, including those of cleaner air, healthier diets, more active lifestyles, and increased exposure to green space.^176^ International economic cooperation will be essential to ensure that global emission targets are met and to prevent the widening of inequity gaps.^13^ Therefore, this section also reflects on the extent to which COVID-19 recovery spending has prioritised green investment (panel 4) and discusses the alignment of fossil fuel companies’ strategies with the requirements of the transition (panel 5).

## Box

***Panel 6*: The place of health in the enhanced nationally determined contributions**

The 2015 Paris Agreement is the only global framework for reducing greenhouse gas emissions to protect people’s health.^287^ Countries have committed to a reduction of emissions via nationally determined contributions (NDCs), which are to be enhanced every 5 years. In 2015–16, 185 countries, including an EU submission for 27 countries, submitted initial NDCs. By July, 2021, 87 countries, including an EU submission for 26 countries, had submitted enhanced or new NDCs.^288^

Compared with their initial NDCs, the proportion of countries referring to health increased, from 49 (56%) of 87 in 2015–16 to 79 (91%) of 87 in 2021. However, health engagement remained low in NDCs. Overall, in both initial and enhanced NDCs, less than 3% of the text related to health; in the enhanced NDCs, text relating to health was an average of 240 of 10466 words. Of the references to health, 249 (30%) of 832 references noted health impacts, challenges, or risks of climate change; for example, “the Kenyan economy is dependent on climate-sensitive sectors, such as rain-fed agriculture, water, energy, tourism, wildlife, and health, whose vulnerability is increased by climate change” (Kenya, updated submission).^289^ An additional 210 (25%) of 832 references related to health sector adaptation; for example, climate change “threatens the ability of health institutions and organizations to maintain and improve health services into the future” (Marshall Islands, updated submission).^290^

The enhanced NDCs had an increased engagement with gender, health, and climate change, with 9 (10%) of 87 NDCs making a meaningful connection compared with just 2 (2%) in their initial contributions. The majority of the references to gender, health, and climate change are references to the specific impact of climate change on women; for example, “further strain on the workload of women and climate change-related stress during pregnancy could contribute to low birth weight, leading to increases in risks of undernutrition and non-communicable diseases” (Cambodia, updated submission).^291^

In summary, although health engagement remains low, there is greater recognition that climate change takes a disproportionate toll on women in the latest NDCs compared with those published in 2015–16.
