# Supplementary material for: The 2021 report of the Lancet Countdown on health and climate change: code red for a healthy future
Source: Lancet. Author manuscript; Available in PMC 2024 Nov 15. (PMC7616807; doi:10.1016/S0140-6736(21)01787-6)
Supplement: Chinese translation of the Executive Summary [file EMS200003-supplement-Chinese_translation_of_the_Executive_Summary.pdf]

THE LANCET

Supplementary appendix

This translation in Chinese was submitted by the authors and we reproduce it as supplied. It has not been peer reviewed. *The Lancet*'s editorial processes have only been applied to the original in English, which should serve as reference for this manuscript.

此简体中文译文由作者提交，我方按照提供的版本刊登。此译文并未经过同行审阅。医学期刊《柳叶刀》的编辑流程仅适用于英文原稿，英文原稿应作为此手稿的参考。

Supplement to: Romanello M, McGushinA, Di Napoli C, et al. The 2021 report of the *Lancet* Countdown on health and climate change: code red for a healthy future. *Lancet* 2021; published online Oct 20. http://dx.doi.org/10.1016/S0140-6736(21)01787-6.

2021 年 Lancet Countdown 健康与气候变化报告: 为健康未来发出红色预警

Marina Romanello, Alice McGushin, Claudia Di Napoli, Paul Drummond, Nick Hughes, Louis Jamart, Harry Kennard, Pete Lampard, Baltazar Solano Rodriguez, Nigel Arnell, Sonja Ayeb-Karlsson, Kristine Belesova, Wenjia Cai, Diarmid Campbell-Lendrum, Stuart Capstick, Jonathan Chambers, Lingzhi Chu, Luisa Ciampi, Carole Dalin, Niheer Dasandi, Shouro Dasgupta, Michael Davies, Paula Dominguez-Salas, Robert Dubrow, Kristie L Ebi, Matthew Eckelman, Paul Ekins, Luis E Escobar, Lucien Georgeson, Delia Grace, Hilary Graham, Samuel H Gunther, Stella Hartinger, Kehan He, Clare Heaviside, Jeremy Hess, Shih-Che Hsu, Slava Jankin, Marcia P Jimenez, Ilan Kelman, Gregor Kiesewetter, Patrick L Kinney, Tord Kjellstrom, Dominic Kniveton, Jason K W Lee, Bruno Lemke, Yang Liu, Zhao Liu, Melissa Lott, Rachel Lowe, Jaime Martinez-Urtaza, Mark Maslin, Lucy McAllister, Celia McMichael, Zhifu Mi, James Milner, Kelton Minor, Nahid Mohajeri, Maziar Moradi-Lakeh, Karyn Morrissey, Simon Munzert, Kris A Murray, Tara Neville, Maria Nilsson, Nick Obradovich, Maquins Odhiambo Sewe, Tadj Oreszczyn, Matthias Otto, Fereidoon Owfi, Olivia Pearman, David Pencheon, Mahnaz Rabbaniha, Elizabeth Robinson, Joacim Rocklöv, Renee N Salas, Jan C Semenza, Jodi Sherman, Liuhua Shi, Marco Springmann, Meisam Tabatabaei, Jonathon Taylor, Joaquin Trinanes, Joy Shumake-Guillemot, Bryan Vu, Fabian Wagner, Paul Wilkinson, Matthew Winning, Marisol Yglesias, Shihui Zhang, Peng Gong, Hugh Montgomery, Anthony Costello, Ian Hamilton

执行摘要

Lancet Countdown 是一项国际合作项目，旨在独立监测变化迅速的气候所带来的健康后果。*Lancet* Countdown 每年发布更新、改进的新指标，它是来自 38 所学术机构和联合国机构的主要研究人员所得的共识。这份报告的 44 个指标揭示了气候变化对人类健康构成的影响以及由于全球各国行动迟缓和不一致对健康的影响有增无减--均提出了加速行动的明确要求，将人类和地球的健康置于首位。

2021 年，这份报告恰逢《联合国气候变化框架公约》第 26 届联合国气候变化大会（COP26）召开之际，各国都面临着巨大压力，因为他们必须实现《巴黎协定》中将全球平均气温上升控制在 1.5℃ 的目标，并调动所有国家所需的财政资源，以采取有效的气候对策。这些谈判是在新冠病毒的背景下展开的--这场全球健康危机已经夺走了数百万人的生命，影响了全球各地的生计和社区，并揭露了各国在应对和回应突发卫生事件的能力方面存在巨大差异和不平等现象。然而，在应对这两场危机时，各国面前都有一个确保全人类享有健康未来的难得机会。

全球变暖导致不平等现象日益加深

相比于 1986-2005 年基线年均值，2020 年创纪录的气温导致 65 岁以上的人暴露热浪的天数增加了31 亿人・日， 1 岁以下儿童的暴露天数增加了 6.26 亿人・日（指标 1.1.2）。 2021年，65 岁以上或 1 岁以下的人群，以及社会弱势群体，受 2021 年 6 月美国和加拿大西北太平洋地区超过 40℃ 的破纪录气温的影响最大--如果没有人类造成的气候变化，这一事件几乎不会发生。虽然确切的数字在几个月后才会揭晓，但有数百人过早死于热浪。此外，在过去 30 年里，人类发展指数（HDI）处于中低水平国家的人口在热脆弱性方面增幅最大，冷却机制和城市绿地供应不足进一步加剧了热浪对他们构成的健康风险（指标 1.1.1、2.3.2 和 2.3.3）。

人类发展指数处于中低水平国家的农民是受极端温度影响最严重的人群之一，在 2020 年因高温而损失的 2950 亿个潜在工作时间中，他们几乎占了一半（指标 1.1.4）。这些工作时间的损失可能会对这些已经处于弱势的工人产生破坏性的经济后果--这份报告中的数据显示，低HDI国家的收入损失约占到这些国家国内生产总值的4-8%。

通过这些影响、平均气温上升和降雨模式的改变，气候变化正开始扭转多年来在解决粮食和水安全方面取得的进展，粮食和水的问题仍然影响着世界各地医疗条件薄弱的人群，使他们无法获得良好健康。在 2020 年的任何特定月份，全球高达 19% 的陆地表面受到极端干旱的影响，这一数值在 1950 年至 1999 年期间从未超过 13%（指标 1.2.2）。在干旱的同时，气温升高正在影响世界主要主食作物的产量潜力--相对于 1981-2010 年，2020 年的玉米产量减少了 6.0%；冬麦产量减少了 3.0%；大豆产量减少了 5.4%；水稻产量减少了 1.8%（指标 1.4.1）--暴露出粮食不安全的风险有所上升。

除了这些健康危害，变化迅速的环境条件也增加了许多水传播、空气传播、食物传播和病媒传播病原体的适宜性。尽管社会经济发展、公共卫生干预和医学进步已经减轻了全球传染病传播负担，但气候变化可能会破坏消除传染病的努力。

从 1950-1959 年到 2010-2019 年，低HDI国家中人口稠密的高原地区，具有适合传播疟疾（恶性疟原虫）的环境条件的月份数增加了 39%，这一趋势威胁到高度弱势人群——相比于低地地区的人口，他们相对更为安全，不受这种疾病影响（指标 1.3.1）。登革热病毒、兹卡病毒和基孔肯雅病毒的流行潜力在全球范围内增加，这些病毒目前主要影响中美洲、南美洲、加勒比地区、

非洲和南亚人口。相比于 20 世纪 50 年代，*埃及伊蚊*传播的基本繁殖率增加了 13%，*冈比亚疟蚊*传播的基本繁殖率增加了 7%。这些虫媒病毒基本繁殖率最大相对增长出现在人类发展指数处于极高水平的国家（指标 1.3.1）；然而，人类发展指数处于低水平的国家的人民最容易受到这些虫媒病毒的影响（指标 1.3.2）。

*霍乱弧菌*的环境适宜性方面也观察到类似的结果。据估计，霍乱弧菌每年造成近 10 万人死亡，尤其是在难以获得安全饮用水和卫生设施的群体中。2003 年至 2019 年，在所有人类发展指数国家组别中，适合霍乱弧菌传播的沿海地区大幅增加--尽管 2020 年 98% 的海岸线适合霍乱弧菌传播，但居于人类发展指数处于低水平的国家组别中的人民对这种疾病的环境适宜性最高（指标 1.3.1）。

极端天气事件、传染病传播以及粮食、水和金融不安全所带来的同时存在且相互关联的风险，使最弱势群体不堪重负。通过多种同时存在且相互影响的健康风险，气候变化有可能扭转多年来在公共卫生和可持续发展方面取得的进展。

即使有非常明确的证据表明气候变化会对健康构成影响，各国也没有采取适应性对策来应对其人口所面临的不断增加的风险。2020 年，在 166 个国家中，104 个国家（63%）的国家卫生应急框架实施程度不高，使得它们无法做好准备应对疫情和气候相关的突发卫生事件（指标 2.3.1）。值得注意的是，在 33 个人类发展指数处于低水平的国家中，只有18 个国家（55%）报告说至少在中等水平上实施了国家卫生应急框架，而在 53 个人类发展指数处于高水平的国家中，有 47 个国家（89%）报告说实施了国家卫生应急框架。此外，在 *91 个国家中，只有 47 个国家（52%）*报告制定了国家卫生适应计划，人力和财政资源不足被认为是落实该计划的主要障碍（指标 2.1.1）。随着全球升温无法避免，即使有最具雄心的气候变化缓解措施，加速适应对于减少气候变化对脆弱人群的影响和保护世界各地人民的健康至关重要。

不公平的对策让所有人失望

截至 2021 年 10 月，全球范围内公平地获得 COVID-19 疫苗接种的目标尚未实现--高收入国有超过 60% 的人至少接受了一剂新冠疫苗，低收入国却只有 3.5% 的人接种了一剂疫苗。这份报告中的数据揭露了全球气候变化缓解对策中类似的不平等现象。

为了实现《巴黎协定》的目标并防止灾难性的全球变暖，全球温室气体排放量必须在十年内减半。然而，按照目前的减排速度，能源系统需要耗时超过 150 年才能完全脱碳（指标 3.1），而各国家之间的不平等对策使得在实现低碳转型的健康利益时失去平衡。

使用公共资金补贴化石燃料是造成脱碳速度缓慢的部分原因。在 84 个接受审查的国家中，有 65 个国家在 2018 年仍在为化石燃料提供全额补贴，在许多情况下，补贴相当于国家卫生预算的很大一部分，本可转用于为健康和福祉提供净效益。此外，碳定价政策超过任何化石燃料补贴影响的所有 19 个国家的人类发展指数（HDI）均处于高水平（指标 4.2.4）。

尽管人类发展指数处于高水平的国家在能源系统脱碳方面集体取得了更多进展，但它们仍是全球二氧化碳排放的主要贡献者，其通过当地生产商品和提供服务产生的二氧化碳占全球排放总量的 45%（指标 4.2.5）。比起人类发展处于极高水平的国家，由于脱碳步伐相对缓慢，空气质量法规较不完善，人类发展指数处于中高水平的国家排放了最多微细颗粒物（PM2·5）——空气污染相关的死亡率最高，总死亡人数比发展指数处于极高水平的国家高出约 50%（指标 3.3）。人类发展指数处于低水平的国家比起其他国家拥有较低的工业活动量，本地生产仅占全球二氧化碳排放量的 0-7%，环境空气污染相关的死亡率最低。然而，在这些国家中，由于只有 12% 的居民依靠清洁燃料和技术烹饪，这些人群的健康状况仍然受到威胁，因为他们可能吸入危害健康的高浓度家庭空气污染物（指标 3.2）。即使在最富裕的国家，最贫困地区的民众也绝大部分承担着因暴露于空气污染物而产生的健康影响。这些发现揭露了迟缓和不平等的减缓行动所带来的健康成本，并强调了通过将所有人群健康放在首位的低碳转型，每年可避免数百万人死亡。

然而，世界尚未步入正轨，没能实现有利于人群健康的低碳经济转型。目前，全球脱碳承诺不足以实现《巴黎协定》的目标，并将导致到本世纪末全球平均气温上升约 2.4°C。后疫情时代支出的当前方向有可能使这种情况变得更糟，到2020年底，在用于新冠疫情经济复苏的资金中，仅有18%有助于减少温室气体排放。根据预测，新冠疫情经济复苏将导致 2021 年的温室气体排放空前地增加5% ，这将使全球的人为排放量回到其峰值。

此外，现阶段经济衰退有可能破坏从 2020 年起每年调动 1000 亿美元来促进服务最欠发达国家的低碳转型和适应性对策的目标，尽管这一数目与分配给新冠疫情复苏的数万亿资金相比是微不足道的。各国在新冠疫情期间被迫求助于大量借款，这可能会削弱他们实现绿色复苏并使其人口在低碳转型中获得最大健康收益的能力。

一个确保全人类享有健康未来的难得机会

由碳密集型的新冠疫情复苏导致的排放过高将不可逆转地阻止世界实现气候承诺和可持续发展目标，并将人类置于一个日益极端和不可预知的环境之中。这份报告中的数据揭示了当前世界比前工业时代升温 1.2 摄氏度的健康影响和健康不平等现象，基于目前事态发展趋势，支持气候变化将成为人类健康的决定性因素的观点。

然而，通过将数万亿美元投入到新冠疫情复苏计划，走向世卫组织的健康、绿色复苏道路，世界可以达成《巴黎协定》的目标，保护有利于健康的自然系统，并通过减少健康影响和最大化低碳转型的协同效益，将不平等现象最小化。促进公平的减缓气候变化和普及清洁能源，每年可以防止数百万人死亡，因为人们减少暴露在空气污染中，拥有更健康的饮食和更积极的生活方式，从而有助减少全球健康不平等现象。这个刺激经济的关键时刻是一次确保今代和子孙后代健康得到保障的历史性机会。

我们可以从本年度的数据看到几个积极变化：2013年至 2018年，可再生风能和太阳能的发电量年均增长了17%（指标 3.1）；2020 年新增煤炭产能的投资下降了10%（指标 4.2.1）；2019 年全球电动车数量达到720万辆（指标 3.4）。此外，全球疫情推动了社会多个领域积极参与到健康和气候变化课题中，91 位国家元首在2020 年的联合国一般性辩论中建立了联系，人类发展指数处于极高水平的国家组别也广泛参与其中（指标 5.4）。新冠疫情复苏会是支持还是扭转这些趋势，还是个未知数。

新冠疫情和气候变化这两者并无国界之分。要是所有国家和社会各界没有推进广泛的接种疫苗计划，严重急性呼吸综合征 2 型冠状病毒（SARS-CoV-2）及其新变种病毒将继续危害我们所有人的健康。同样，应对气候变化也需要各个国家采取紧急的、协调性对策，将新冠疫情复苏资金用于支持并确保全球向低碳未来和气候变化适应的公正转型。世界各国领导人拥有一次千载难逢的机会去实现一个改善健康、减少不平等以及经济和环境可持续性的未来。然而，只有当世界携手合作并确保不让每一个人掉队，这一目标才得以实现。
