# Supplementary material for: The 2021 report of the Lancet Countdown on health and climate change: code red for a healthy future
Source: Lancet. Author manuscript; Available in PMC 2024 Nov 15. (PMC7616807; doi:10.1016/S0140-6736(21)01787-6)
Supplement: French translation of the Executive Summary [file EMS200003-supplement-French_translation_of_the_Executive_Summary.pdf]

THE LANCET

Supplementary appendix

This translation in French was submitted by the authors and we reproduce it as supplied. It has not been peer reviewed. *The Lancet*'s editorial processes have only been applied to the original in English, which should serve as reference for this manuscript.

Cette traduction en français a été proposée par les auteurs et nous l'avons reproduite telle quelle. Elle n'a pas été examinée par des pairs. Les processus éditoriaux du *Lancet* n'ont été appliqués qu'à l'original en anglais et c'est cette version qui doit servir de référence pour ce manuscrit.

Supplement to: Romanello M, McGushinA, Di Napoli C, et al. The 2021 report of the *Lancet* Countdown on health and climate change: code red for a healthy future. *Lancet* 2021; published online Oct 20. http://dx.doi.org/10.1016/S0140-6736(21)01787-6.

Rapport 2021 du Lancet Countdown sur la santé et le changement climatique : code rouge pour un avenir sain

Marina Romanello, Alice McGushin, Claudia Di Napoli, Paul Drummond, Nick Hughes, Louis Jamart, Harry Kennard, Pete Lampard, Baltazar Solano Rodriguez, Nigel Arnell, Sonja Ayeb-Karlsson, Kristine Belesova, Wenjia Cai, Diarmid Campbell-Lendrum, Stuart Capstick, Jonathan Chambers, Lingzhi Chu, Luisa Ciampi, Carole Dalin, Niheer Dasandi, Shouro Dasgupta, Michael Davies, Paula Dominguez-Salas, Robert Dubrow, Kristie L Ebi, Matthew Eckelman, Paul Ekins, Luis E Escobar, Lucien Georgeson, Delia Grace, Hilary Graham, Samuel H Gunther, Stella Hartinger, Kehan He, Clare Heaviside, Jeremy Hess, Shih-Che Hsu, Slava Jankin, Marcia P Jimenez, Ilan Kelman, Gregor Kiesewetter, Patrick L Kinney, Tord Kjellstrom, Dominic Kniveton, Jason K W Lee, Bruno Lemke, Yang Liu, Zhao Liu, Melissa Lott, Rachel Lowe, Jaime Martinez-Urtaza, Mark Maslin, Lucy McAllister, Celia McMichael, Zhifu Mi, James Milner, Kelton Minor, Nahid Mohajeri, Maziar Moradi-Lakeh, Karyn Morrissey, Simon Munzert, Kris A Murray, Tara Neville, Maria Nilsson, Nick Obradovich, Maquins Odhiambo Sewe, Tadj Oreszczyn, Matthias Otto, Fereidoon Owfi, Olivia Pearman, David Pencheon, Mahnaz Rabbaniha, Elizabeth Robinson, Joacim Rocklöv, Renee N Salas, Jan C Semenza, Jodi Sherman, Liuhua Shi, Marco Springmann, Meisam Tabatabaei, Jonathon Taylor, Joaquin Trinanes, Joy Shumake-Guillemot, Bryan Vu, Fabian Wagner, Paul Wilkinson, Matthew Winning, Marisol Yglesias, Shihui Zhang, Peng Gong, Hugh Montgomery, Anthony Costello, Ian Hamilton

Résumé

Le *Lancet* Countdown est une collaboration internationale qui surveille de manière indépendante les conséquences sanitaires du changement climatique. Publiant chaque année des indicateurs mis à jour, nouveaux et améliorés, le *Lancet* Countdown représente le consensus de chercheurs de premier plan de 38 institutions universitaires et agences des Nations Unies. Les 44 indicateurs de ce rapport exposent une augmentation incessante des impacts sanitaries du changement climatique et les conséquences actuelles sur la santé de la réponse retardée et incohérente des pays du monde entier, fournissant un impératif clair pour une action accélérée qui place la santé des personnes et de la planète au premier plan.

Le rapport 2021 coïncide avec la 26ème Conférence des Parties à la Convention-cadre des Nations Unies sur les changements climatiques (COP26), pendant laquelle les chefs d'État seront confrontés à des pressions politiques pour réaliser l'ambition de l'Accord de Paris de maintenir la hausse de la température moyenne mondiale en-desous 1,5°C et de mobiliser les ressources financières nécessaires pour que tous les pays aient une réponse climatique efficace. Ces négociations se déroulent dans le contexte de la pandémie du COVID-19, crise sanitaire mondiale qui a coûté la vie à des millions de personnes, affecté les moyens de subsistance dans les communautés du monde entier, et révélé de profondes fissures et inégalités dans la capacité du monde à faire face et à y répondre aux urgences sanitaires . Pourtant, dans sa réponse aux deux crises, le monde est confronté à une occasion sans précédent d'assurer un avenir sain pour tous.

Aggravation des inégalités dans un monde qui se réchauffe

Les températures record en 2020 ont entraîné un nouveau sommet de 3,1 milliards de nombre de jours supplémentaires d'exposition des personnes âgées de plus de 65 ans aux épisodes caniculaires, âet de 626 millions de jours-

de plus touchant les enfants de moins de 1 an, par rapport à la moyenne annuelle entre 1986 à 2005 (indicateur 1.1.2). En 2021, les personnes âgées de plus de 65 ans ou de moins de 1 an, ainsi que les personnes confrontées à des désavantages sociaux, ont été les plus touchées par les températures record de plus de 40 °C dans la région nord-ouest du Pacifique des États-Unis et du Canada en juin 2021 – un événement qui aurait été presque impossible sans le changement climatique causé par l'homme. Bien que le nombre exact ne soit pas connu avant plusieurs mois, des centaines de personnes sont mortes prématurément de la chaleur. En outre, les populations des pays ayant des niveaux faibles et moyens d'indice de développement humain (IDH) défini par l'ONU ont connu la plus forte augmentation de la vulnérabilité à la chaleur au cours des 30 dernières années, les risques pour leur santé accentués par la faible disponibilité de mécanismes de refroidissement et d'espaces verts urbains (indicateurs 1.1.1, 2.3.2 et 2.3.3).

Les travailleurs agricoles des pays à IDH faible et moyen ont été parmi les plus touchés par l'exposition à des températures extrêmes, supportant près de la moitié des 295 milliards d'heures de travail potentielles perdues en raison de la chaleur en 2020 (indicateur 1.1.4). Ces heures de travail perdues pourraient avoir des conséquences économiques dévastatrices pour ces travailleurs déjà vulnérables – les données du rapport de cette année montrent que les gains potentiels

moyens perdus dans les pays du groupe à faible IDH équivalaient à entre 4 à 8 % de la production intérieure brute nationale pour les pays de ce groupe (indicateur 4.1.3).

À travers ces effets, la hausse des températures moyennes, et la modification des régimes pluviométriques, le changement climatique commence à inverser des années de progrès dans la lutte contre l'insécurité alimentaire et hydrique qui affecte encore les populations les plus mal desservies dans le monde, les privant d'un aspect essentiel lié à une bonne santé. Au cours d'un mois donné en 2020, jusqu'à 19 % de la surface terrestre mondiale a été affectée par une sécheresse extrême, une valeur qui n'avait pas dépassé 13 % entre 1950 et 1999 (indicateur 1.2.2). En même temps que la sécheresse, les températures chaudes affectent le potentiel de rendement des principales cultures de base du monde – une réduction de 6,0 % pour le maïs ; 3,0 % pour le blé d'hiver ; 5,4 % pour le soja ; et 1,8 % pour le riz en 2020, par rapport à 1981-2010 (indicateur 1.4.1), ce qui expose au risque croissant d'insécurité alimentaire.

En plus de ces risques pour la santé, les conditions environnementales changeantes augmentent également l'aptitude à la transmission de nombreux agents pathogènes d'origine hydrique, atmosphérique, alimentaire et vectorielle. Bien que le développement socio-économique, les interventions de santé publique et les progrès de la médecine aient réduit le fardeau mondial de la transmission des maladies infectieuses, le changement climatique pourrait saper les efforts d'éradication.

Le nombre de mois où les conditions de transmission du paludisme *(Plasmodium falciparum)* sont appropriées sur le plan environnemental a augmenté de 39 % entre 1950-59 et 2010-2019 dans les zones de hautes terres densément peuplées du groupe à faible IDH, menaçant les populations très défavorisées qui étaient comparativement plus à l'abri de cette maladie que celles des zones de plaine (indicateur 1.3.1). Le potentiel épidémique du virus de la dengue, du virus Zika et du virus chikungunya, qui touchent actuellement principalement les populations d'Amérique centrale, d'Amérique du Sud, des Caraïbes, d'Afrique et d'Asie du Sud, a augmenté à l'échelle mondiale, avec une augmentation du taux de reproduction de base de 13 % pour la transmission par *Anopheles aegypti* et de 7 % pour la transmission par *Anopheles albopictus* par rapport aux années 1950. La plus forte augmentation relative du taux de reproduction de base de ces arbovirus a été observée dans les pays du groupe à IDH très élevé (indicateur 1.3.1); cependant, les personnes du groupe à faible IDH sont les plus vulnérables à ces arbovirus (indicateur 1.3.2).

Des résultats similaires sont observés dans l'adéquation environnementale du *Vibrio cholerae*, un agent pathogène qui causerait près de 100 000 décès par an, en particulier parmi les populations ayant un accès limité à l'eau potable et à l'assainissement. Entre 2003 et 2019, les zones côtières propices à la transmission du *choléra V* ont considérablement augmenté dans tous les groupes de pays de l'IDH – bien que, avec 98 % de leur littoral propice à la transmission du *V. cholerae* en 2020, ce sont les personnes du groupe de pays à faible IDH qui ont la plus grande aptitude environnementale à cette maladie (indicateur 1.3.1).

Les risques concomitants et interconnectés posés par les phénomènes météorologiques extrêmes, la transmission de maladies infectieuses et l'insécurité alimentaire, hydrique et financière surchargent les populations les plus vulnérables. En raison de multiples risques sanitaires simultanés et en interaction, le changement climatique menace d'inverser des années de progrès en matière de santé publique et de développement durable.

Même avec des preuves accablantes sur les impacts du changement climatique sur la santé, les pays ne fournissent pas une réponse d'adaptation proportionnelle aux risques croissants auxquels leurs populations sont confrontées. En 2020, 104 (63 %) des 166 pays ayant répondu à une enquête de l'organisation mondiale de santé n'avaient pas un niveau élevé de mise en œuvre des cadres nationaux d'urgence sanitaire, ce qui les laissait mal préparés à répondre aux pandémies et aux urgences sanitaires liées au climat (indicateur 2.3.1). Il est important de souligner que seuls 18 (55 %) des 33 pays ayant un IDH faible avaient signalé un niveau moyen de mise en œuvre des cadres nationaux d'urgence sanitaire, contre 47 (89 %) des 53 pays ayant un IDH très élevé. En outre, seuls 47 (52 %) des 91 pays ont déclaré disposer d'un plan national d'adaptation en matière de santé, l'insuffisance des ressources humaines et financières étant identifiée comme le principal obstacle à leur mise en œuvre (indicateur 2.1.1). Avec un monde confronté à une augmentation inévitable des températures, même avec l'atténuation du changement climatique la plus ambitieuse, une adaptation accélérée est essentielle pour réduire les vulnérabilités des populations au changement climatique et protéger la santé des populations du monde entier.

Une réponse inéquitable met tout le monde en échec

Dix mois après le début de 2021, l'accès mondial et équitable au vaccin contre le COVID-19 n'avait pas été assuré - plus de 60 % des personnes dans les pays à revenu élevé ont reçu au moins une dose d'un vaccin contre le COVID-19, contre seulement 3,5 % des personnes dans les pays à faible revenu. Les données de ce rapport exposent des inégalités similaires dans la réponse mondiale à l'atténuation du changement climatique.

Pour atteindre les objectifs de l'Accord de Paris et prévenir des niveaux catastrophiques de réchauffement climatique, les émissions mondiales de gaz à effet de serre doivent être diminuées de moitié d'ici une décennie. Cependant, au rythme actuel de réduction, il faudrait plus de 150 ans pour que le système énergétique se décarbonise complètement (indicateur 3.1), et la réponse inégale entre les pays se traduit par une réalisation inégale des avantages pour la santé d'une transition à faible intensité de carbone.

L'utilisation de fonds publics pour subventionner les combustibles fossiles est en partie responsable de la lenteur du taux de décarbonisation. Sur les 84 pays examinés, 65 fournissaient encore une subvention globale aux combustibles fossiles en 2018 et, dans de nombreux cas, les subventions équivalaient à des proportions substantielles du budget national de la santé et auraient pu être réorientées pour apporter des avantages nets à la santé et au bien-être. En outre, les 19 pays dont les politiques de tarification du carbone l'emportaient sur l'effet de toute subvention aux combustibles fossiles provenaient du groupe des IDH très élevés (indicateur 4.2.4).

Bien que les pays du groupe à IDH très élevé aient collectivement fait le plus de progrès dans la décarbonisation du système énergétique, ils restent les principaux contributeurs aux émissions de CO_2 par la production locale de biens et de services, représentant 45 % du total mondial (indicateur 4.2.5). Avec un rythme de décarbonisation plus lent et des réglementations plus médiocres en matière de qualité de l'air que les pays du groupe des IDH très élevés, les groupes de pays à IDH moyen et élevé produisent le plus d'émissions de particules fines ($PM_{2·5}$) et ont les taux les plus élevés de décès liés à la pollution atmosphérique, qui sont environ 50 % plus élevés que les décès totaux dans le groupe IDH très élevé (indicateur 3.3). Le groupe à faible IDH, avec des quantités d'activité industrielle comparativement plus faibles que dans les autres groupes, a une production locale qui ne contribue qu'à 0,7 % des émissions mondiales de CO_2 et il a le taux de mortalité le plus faible de la pollution de l'air ambiant. Cependant, avec seulement 12 % de ses habitants dépendant de combustibles et de technologies propres pour cuisiner, la santé de ces populations est toujours menacée par des concentrations dangereusement élevées de pollution de l'air domestique (indicateur 3.2). Même dans les pays les plus riches, les habitants des zones les plus défavorisées supportent massivement le fardeau des effets sur la santé de l'exposition à la pollution atmosphérique. Ces résultats exposent les coûts sanitaires de la réponse d'atténuation retardée et inégale et soulignent les millions de décès à prévenir chaque année grâce à une transition à faible émission de carbone qui donne la priorité à la santé de toutes les populations.

Cependant, le monde n'est pas sur la bonne voie pour réaliser les gains en matière de santé de la transition vers une économie à faible émission de carbone. Les engagements mondiaux actuels en matière de décarbonisation sont insuffisants pour répondre à l'ambition de l'Accord de Paris et entraîneraient une augmentation de la température moyenne mondiale d'environ 2,4 °C d'ici la fin du siècle. L'orientation actuelle des dépenses post-COVID-19 menace d'aggraver cette situation, avec seulement 18 % de tous les fonds engagés pour la reprise économique après la pandémie du COVID-19 d'ici la fin de 2020 qui devrait conduire à une réduction des émissions de gaz à effet de serre. En effet, la reprise économique après la pandémie devrait déjà conduire à une augmentation sans précédent de 5 % des émissions de gaz à effet de serre en 2021, ce qui ramènera les émissions anthropiques mondiales à leurs niveaux record.

En outre, la récession économique actuelle menace de saper l'objectif de mobiliser 100 milliards de dollars par an à partir de 2020 pour promouvoir des changements à faible émission de carbone et des réponses d'adaptation dans les pays les plus mal desservis, même si cette quantité est infime par rapport aux billions alloués à la reprise post-COVID-19. Les montants élevés d'emprunt auxquels les pays ont dû recourir pendant la pandémie pourraient effacer leur capacité à assurer une reprise verte et maximiser les gains en matière de santé pour leur population d'une transition à faible émission de carbone.

Une occasion sans précédent d'assurer un avenir sain pour tous

Le dépassement des émissions résultant d'une reprise à forte intensité de carbone suite au COVID-19 empêcherait irréversiblement le monde de respecter les engagements climatiques et les objectifs de développement durable, et enfermerait l'humanité dans un environnement de plus en plus extrême et imprévisible. Les données de ce rapport exposent les impacts sur la santé et les inégalités en matière de santé du monde actuel à 1,2 °C de réchauffement par rapport à l'époque préindustrielle, et soutiennent que, sur la trajectoire actuelle, le changement climatique deviendra le récit déterminant de la santé humaine.

Cependant, en orientant les milliers de milliards de dollars qui seront engagés pour la reprise du COVID-19 vers les prescriptions de l'OMS pour une reprise saine et verte, le monde pourrait atteindre les objectifs de l'Accord de Paris, protéger les systèmes naturels qui soutiennent le bien-être et minimiser les inégalités grâce à la réduction des effets sur la santé et à la maximisation des co-avantages d'une transition universelle à faible

émission de carbone. La promotion d'une atténuation équitable du changement climatique et d'un accès universel aux énergies propres pourrait prévenir des millions de décès chaque année dus à une exposition réduite à la pollution atmosphérique, à une alimentation plus saine et à des modes de vie plus actifs, et contribuer à réduire les inégalités en matière de santé à l'échelle mondiale. Ce moment charnière de la relance économique représente une occasion historique d'assurer la santé des générations présentes et futures.

Il y a un aperçu de changement positif à travers plusieurs tendances prometteuses dans les données de cette année : la production d'électricité à partir d'énergie éolienne et solaire renouvelable a augmenté de 17 % entre 2013 et 2018 en moyenne annuelle (indicateur 3.1) ; l'investissement dans de nouvelles capacités charbonnières a diminué de 10 % en 2020 (indicateur 4.2.1) ; et le nombre mondial de véhicules électriques a atteint 7,2 millions en 2019 (indicateur 3.4). En outre, la pandémie mondiale a entraîné un engagement accru dans le domaine de la santé et du changement climatique dans de multiples domaines de la société, 91 chefs d'État se rapprochant lors du débat général des Nations Unies de 2020 et un engagement nouvellement généralisé parmi les pays du groupe des IDH très élevés (indicateur 5.4). Il reste à voir si la reprise liée au COVID-19 soutient ou inverse ces tendances.

Ni le COVID-19 ni le changement climatique ne respectent les frontières nationales. Sans une vaccination généralisée et accessible dans tous les pays et toutes les sociétés, le SRAS-CoV-2 et ses nouvelles variantes continueront de mettre en danger la santé de tous. De même, la lutte contre le changement climatique exige que tous les pays apportent une réponse urgente et coordonnée, avec des fonds de relance COVID-19 alloués pour soutenir et assurer une transition juste vers un avenir à faible émission de carbone et l'adaptation au changement climatique à travers le monde. Les dirigeants du monde ont une occasion sans précédent d'offrir un avenir d'amélioration de la santé, de réduction des inégalités et de durabilité économique et environnementale. Cependant, cela ne sera possible que si le monde agit ensemble pour s'assurer que personne n'est laissé pour compte.
