# Supplementary material for: The 2021 report of the Lancet Countdown on health and climate change: code red for a healthy future
Source: Lancet. Author manuscript; Available in PMC 2024 Nov 15. (PMC7616807; doi:10.1016/S0140-6736(21)01787-6)
Supplement: German translation of the Executive Summary [file EMS200003-supplement-German_translation_of_the_Executive_Summary.pdf]

THE LANCET

Supplementary appendix

This translation in German was submitted by the authors and we reproduce it as supplied. It has not been peer reviewed. *The Lancet*'s editorial processes have only been applied to the original in English, which should serve as reference for this manuscript.

Diese Übersetzung in deutscher Sprache wurde von den Autoren eingereicht und wir reproduzieren sie wie vorgelegt. Die Übersetzung wurde nicht von Experten begutachtet. Die redaktionellen Prozesse von The Lancet wurden nur auf das Original in englischer Sprache angewendet, das als Referenz für dieses Manuskript dienen soll.

Supplement to: Romanello M, McGushinA, Di Napoli C, et al. The 2021 report of the *Lancet* Countdown on health and climate change: code red for a healthy future. *Lancet* 2021; published online Oct 20. http://dx.doi.org/10.1016/S0140-6736(21)01787-6.

Der *Lancet* Countdown Jahresbericht 2021 zu Gesundheit und Klimawandel: Alarmstufe Rot für eine gesunde Zukunft

Marina Romanello, Alice McGushin, Claudia Di Napoli, Paul Drummond, Nick Hughes, Louis Jamart, Harry Kennard, Pete Lampard, Baltazar Solano Rodriguez, Nigel Arnell, Sonja Ayeb-Karlsson, Kristine Belesova, Wenjia Cai, Diarmid Campbell-Lendrum, Stuart Capstick, Jonathan Chambers, Lingzhi Chu, Luisa Ciampi, Carole Dalin, Niheer Dasandi, Shouro Dasgupta, Michael Davies, Paula Dominguez-Salas, Robert Dubrow, Kristie L Ebi, Matthew Eckelman, Paul Ekins, Luis E Escobar, Lucien Georgeson, Delia Grace, Hilary Graham, Samuel H Gunther, Stella Hartinger, Kehan He, Clare Heaviside, Jeremy Hess, Shih-Che Hsu, Slava Jankin, Marcia P Jimenez, Ilan Kelman, Gregor Kiesewetter, Patrick L Kinney, Tord Kjellstrom, Dominic Kniveton, Jason K W Lee, Bruno Lemke, Yang Liu, Zhao Liu, Melissa Lott, Rachel Lowe, Jaime Martinez-Urtaza, Mark Maslin, Lucy McAllister, Celia McMichael, Zhifu Mi, James Milner, Kelton Minor, Nahid Mohajeri, Maziar Moradi-Lakeh, Karyn Morrissey, Simon Munzert, Kris A Murray, Tara Neville, Maria Nilsson, Nick Obradovich, Maquins Odhiambo Sewe, Tadj Oreszczyn, Matthias Otto, Fereidoon Owfi, Olivia Pearman, David Pencheon, Mahnaz Rabbaniha, Elizabeth Robinson, Joacim Rocklöv, Renee N Salas, Jan C Semenza, Jodi Sherman, Liuhua Shi, Marco Springmann, Meisam Tabatabaei, Jonathon Taylor, Joaquin Trinanes, Joy Shumake-Guillemot, Bryan Vu, Fabian Wagner, Paul Wilkinson, Matthew Winning, Marisol Yglesias, Shihui Zhang, Peng Gong, Hugh Montgomery, Anthony Costello, Ian Hamilton

Kurzfassung

Der *Lancet* Countdown ist ein internationales Kollaborationsprojekt, das die gesundheitlichen Folgen des Klimawandels unabhängig analysiert. Jedes Jahr veröffentlicht der *Lancet* Countdown aktualisierte, neue und verbesserte Indikatoren und stellt den Konsens führender Forscher von 43 akademischen Einrichtungen und UN-Agenturen vor. Die 44 im Bericht untersuchten Indikatoren zeigen, dass die gesundheitlichen Auswirkungen des Klimawandels stets zunehmen und verdeutlichen die Konsequenzen, die durch Verzögerungen und Uneinheitlichkeit von internationalen Maßnahmen entstehen. Sie unterstreichen, dass es notwendig ist, schneller zu reagieren und dabei die Gesundheit von Menschen und die Gesundheit des Planeten an erste Stelle zu stellen.

Der Jahresbericht 2021 fällt mit der UN-Klimakonferenz COP26 zusammen, welches die Zusammenkunft jener Staaten ist, die der Klimarahmenkonvention (UN Framework Convention on Climate Change) beigetreten sind. Die teilnehmenden Länder stehen dabei in der Verantwortung, das Ziel des Abkommens von Paris umzusetzen und den weltweiten durchschnittlichen Temperaturanstieg auf 1,5 °C zu begrenzen. Des Weiteren müssen die erforderlichen finanziellen Mittel bereitgestellt werden, um allen Ländern eine effektive Umsetzung von Maßnahmen gegen den Klimawandel zu ermöglichen. Die Verhandlungen finden im Kontext der COVID-19-Pandemie statt. Diese globale Gesundheitskrise hat Millionen von Leben gefordert, Existenzgrundlagen zerstört und Gemeinschaften in aller Welt betroffen. Sie hat zudem gezeigt, dass es international eine große Ungleichheit und Spaltung gibt in der Fähigkeit auf Gesundheitskrisen angemessen zu reagieren und diese zu bewältigen. Dennoch bieten uns diese Krisen eine noch nie dagewesene Gelegenheit, um für eine gesunde Zukunft für alle zu sorgen.

Intensivierte Ungleichheiten in einer sich erwärmenden Welt

Im Vergleich zum Jahresdurchschnitt zwischen 1986 und 2005 (Indikator 1.1.2) waren durch die Rekordtemperaturen im Jahr 2020 3,1 Milliarden zusätzliche Personentage mit Belastung durch Hitzewellen unter Menschen über 65 Jahren zu verzeichnen – ein neuer Höchststand. Bei Kindern unter einem Jahr waren es 626 Millionen zusätzliche Personentage. Im Jahr 2021 waren die genannten Personengruppen neben sozial benachteiligten Menschen am stärksten von den Rekordtemperaturen von über 40 °C betroffen, die im pazifischen Nordwesten der USA und in Kanada im Juni auftraten. Dieses Ereignis wäre ohne den durch Menschen verursachten Klimawandel praktisch unmöglich gewesen. Obwohl uns die exakten Zahlen erst in einigen Monaten vorliegen werden, wissen wir schon jetzt, dass die Hitze hunderte vorzeitige Todesfälle verursacht hat. Bevölkerungen in Ländern mit einem niedrigen bis mittleren UN-Entwicklungsindex HDI (Human Development Index) erlebten im Verlauf der letzten 30 Jahre die stärkste Zunahme an Vulnerabilität gegenüber Hitzeereignissen. Die gesundheitlichen Risiken werden dabei durch das Fehlen von ausreichenden Kühlmechanismen und urbanen Grünflächen weiter erhöht (Indikatoren 1.1.1, 2.3.2 und 2.3.3).

In der Landwirtschaft tätige Menschen in Ländern mit einem niedrigen bis mittleren HDI zählten zu den am meisten von extremen Temperaturen Betroffenen. Fast die Hälfte der 295 Milliarden potenziellen Arbeitsstunden, die 2020 aufgrund von Hitze verloren gingen, fielen auf diese Gruppe zurück (Indikator 1.1.4). Diese verlorenen Arbeitsstunden können katastrophale ökonomische Konsequenzen haben für diese bereits vulnerable Gruppe von Arbeiterinnen und Arbeitern. Die Daten des diesjährigen Berichts zeigen, dass die

durchschnittlichen potenziellen Einkommensverluste in Ländern mit einem niedrigen HDI 4 bis 8 % des jeweiligen nationalen Bruttoinlandsprodukts betrugen (Indikator 4.1.3).

Durch steigende Durchschnittstemperaturen und geänderte Niederschlagsmuster führt der Klimawandel dazu, dass Jahre des Fortschritts im Kampf gegen Nahrungsmittel- und Wasserversorgungsunsicherheit zunichte gemacht werden. Diese Unsicherheit betrifft Bevölkerungen, die weltweit am unterversorgtesten sind und versagt ihnen einen entscheidenden Aspekt für eine gute Gesundheit. In jedem beliebigen Monat des Jahres 2020 waren bis zu 19 % der globalen Landoberfläche von extremer Dürre betroffen. Zwischen 1950 und 1999 wurde dagegen nie ein Wert von 13 % überschritten. Neben Dürren beeinträchtigen zudem die warmen Temperaturen das Ertragspotenzial der bedeutendsten Grundnahrungserzeugnisse der Welt. Im Jahr 2020 waren die Erträge im Vergleich zu 1981 bis 2010 (Indikator 1.4.1) um folgende Anteile geringer: 6,0 % für Mais, 3,0 % für Winterweizen, 5,4 % für Sojabohnen und 1,8 % für Reis. Dies verdeutlicht das steigende Risiko für Nahrungsmittelunsicherheit.

Zusätzlich zu diesen Gesundheitsgefahren kommen weitere hinzu. Beispielsweise erhöhen sich ändernde Umweltbedingungen das Potenzial für die Übertragung zahlreicher Krankheitserreger durch Wasser, Luft, Nahrungsmittel und Vektoren. Obwohl die globale Belastung durch Infektionskrankheiten reduziert werden konnte dank sozioökonomischen Entwicklungen, Interventionen im Bereich der öffentlichen Gesundheit und medizinischen Fortschritten, könnte der Klimawandel die Anstrengungen zur Ausrottung dieser Krankheiten untergraben.

Die Anzahl der Monate mit für die Übertragung von Malaria (*Plasmodium falciparum*) geeigneten Umweltbedingungen stieg in dicht bevölkerten Hochlandregionen mit einem niedrigen HDI vom Zeitraum zwischen 1950 und 1959 bis zum Zeitraum 2010 bis 2019 um 39 %. Somit sind hochgradig benachteiligte Bevölkerungen nun bedroht, die im Vergleich zu Bevölkerungen in tief gelegenen Gebieten relativ sicher vor dieser Krankheit waren (Indikator 1.3.1). Das Epidemiepotenzial von Viren wie dem Dengue-Virus, dem Zika-Virus und dem Chikungunya-Virus, die aktuell hauptsächlich Bevölkerungen in Mittelamerika, Südamerika, der Karibik, Afrika und Südasien betreffen, ist weltweit angestiegen. Die Basisreproduktionsrate für die Übertragung durch *Anopheles aegypti* stieg um 13 % und für die Übertragung durch *Anopheles albopictus* um 7 % im Vergleich zu den 1950ern. Der höchste relative Anstieg der Basisreproduktionsrate wurde in Ländern mit einem sehr hohen HDI beobachtet (Indikator 1.3.1), allerdings ist die Vulnerabilität gegenüber Arboviren für Menschen aus Ländern mit einem niedrigen HDI am höchsten (Indikator 1.3.2).

Ähnliches lässt sich auch im Hinblick auf geeignete Umweltbedingungen für *Vibrio cholerae* beobachten. Dieser Krankheitserreger ist Schätzungen zufolge jährlich für fast 100.000 Todesfälle verantwortlich, besonders unter Bevölkerungen mit unzureichendem Zugang zu sicherem Wasser und sanitären Anlagen. Zwischen 2003 und 2019 stieg die Fläche von Küstengebieten, die für die Übertragung von *Vibrio cholerae* geeignete Bedingungen aufweisen, beträchtlich an. Allerdings sind Menschen in Ländern mit einem niedrigen HDI am meisten betroffen – 98 % der Küsten dieser Länder weisen geeignete Bedingungen für die Übertragung von *Vibrio cholerae* auf (Indikator 1.3.1).

Die gleichzeitig bestehenden und zusammenhängenden Risiken, die durch Extremwetterereignisse, Infektionskrankheiten, Nahrungsmittel-, Wasser- und finanzielle Unsicherheiten entstehen, überlasten die am meisten anfälligen Bevölkerungen. Durch die Vielfalt bestehender und interagierender Gesundheitsrisiken droht der Klimawandel, Jahre des Fortschritts in den Bereichen öffentliche Gesundheit und nachhaltige Entwicklung zunichtezumachen.

Selbst angesichts der überwältigenden Evidenz im Hinblick auf die gesundheitlichen Auswirkungen des Klimawandels setzen Länder keine Anpassungen um, die im Verhältnis zu den steigenden Risiken für ihre jeweiligen Bevölkerungen angemessen sind. Im Jahr 2020 wiesen 104 von 166 Ländern (63 %) keinen hohen Umsetzungsgrad im Hinblick auf nationale Notfallrahmenpläne für das Gesundheitswesen auf. Diese Länder sind daher nicht vorbereitet, um auf Pandemien und klimabedingte Gesundheitskrisen zu reagieren (Indikator 2.3.1). Bedeutender Weise berichteten nur 18 von 33 Ländern mit einem niedrigen HDI (55 %) zumindest einen mittleren Umsetzungsgrad im Hinblick auf nationale Notfallrahmenpläne für das Gesundheitswesen. Für Länder mit einem sehr hohen HDI trifft dies auf 47 von 53 zu (89 %). Außerdem berichteten nur 47 von 91 Ländern (52 %), dass ein nationaler Anpassungsplan für das Gesundheitswesen besteht. Nicht ausreichende menschliche und finanzielle Ressourcen wurden als die wichtigsten Hindernisse für eine Implementierung identifiziert (Indikator 2.1.1). Da sich ein globaler Temperaturanstieg selbst mit den ehrgeizigsten Strategien zur Eindämmung des Klimawandels nicht vermeiden lässt, ist eine beschleunigte Anpassung entscheidend, um die Vulnerabilitäten von Bevölkerungen im Hinblick auf den Klimawandel zu reduzieren und die Gesundheit von Menschen weltweit zu schützen.

Die Ungleichheit in globalen Reaktionen schadet allen

Im Oktober 2021 konnte noch kein globaler, gerechter Zugang zu COVID-19-Impfstoffen erreicht werden. Mehr als 60 % der Bevölkerung in Ländern mit hohem Einkommen haben zumindest eine Dosis eines COVID-19-Impfstoffs erhalten. In Ländern mit niedrigem Einkommen beträgt dieser Wert gerade einmal 3,5 %. Die Daten dieses Berichts zeigen vergleichbare Ungleichheiten in der globalen Reaktion zur Eindämmung des Klimawandels.

Um die Ziele des Abkommens von Paris zu erreichen und ein katastrophales Ausmaß der Erderwärmung zu verhindern, müssen Treibhausgasemissionen innerhalb des nächsten Jahrzehnts um die Hälfte reduziert werden. Allerdings würde es bei der aktuellen Geschwindigkeit der Reduktion mehr als 150 Jahre dauern, bis die Dekarbonisierung des Energiesystems vollständig abgeschlossen ist (Indi kator 3.1). Die Ungleichheit der Reaktionen von Ländern führt zu einem ungleichmäßigem Erhalt von gesundheitlichen Vorteile durch die Umstellung auf emissionsarme Technologien.

Der Einsatz öffentlicher Mittel zur Subventionierung fossiler Brennstoffe ist teilweise für die langsame CO_2-Reduktion verantwortlich. 65 der 84 untersuchten Länder subventionierten 2018 weiterhin fossile Brennstoffe. In vielen Fällen entsprachen diese Subventionen einem beträchtlichen Anteil des Budgets für nationale Gesundheit. Durch die Umverteilung dieser Mittel hätte ein Nettonutzen für die Gesundheit und das Gesamtwohl erreicht werden können. Des Weiteren gehören alle 19 Länder, deren CO_2-Preisgestaltungspolitik die Effekte jeglicher Subventionen für fossile Brennstoffe übertrifft, zur Gruppe mit einem sehr hohen HDI (Indikator 4.2.4).

Auch wenn die Länder in der Gruppe mit einem sehr hohen HDI gemeinsam die größten Fortschritte im Hinblick auf die CO_2-Reduktion im Energiesystem erzielt haben, so sind diese Länder jedoch durch die lokale Produktion von Gütern und Diensten mit 45 % des globalen Gesamtwerts weiterhin Hauptverursacher von CO_2-Emissionen (Indikator 4.2.5). Die Gruppe von Ländern mit einem mittleren bis hohen HDI weist im Vergleich zu Ländern mit einem sehr hohen HDI eine langsamere CO_2-Reduktion auf, hat schlechtere Luftqualitätsvorschriften und produziert die höchsten Emissionen an Feinstaub (PM 2,5). In diesen Ländern sind die Raten von Todesfällen aufgrund von Luftverschmutzung am höchsten. Sie liegen um ca. 50 % über dem Wert der Gruppe mit einem sehr hohen HDI (Indikator 3.3). Ländern mit einem niedrigen HDI weisen im Vergleich zu anderen Gruppen weniger industrielle Aktivitäten auf, sodass die lokale Produktion nur 0,7 % der globalen CO_2-Emissionen ausmacht. Die Mortalitätsrate aufgrund von Verschmutzung der Umgebungsluft ist in dieser Gruppe am niedrigsten. Allerdings stehen hier nur für 12 % der Einwohner saubere Brennstoffe und Technologien zum Kochen zur Verfügung, sodass das Risiko gefährlich hoher Konzentrationen von verschmutzter Innenraumluft besteht (Indikator 3.2). Selbst in den wohlhabendsten Ländern tragen Menschen in benachteiligten Gebieten einen Großteil der Belastung durch die gesundheitlichen Auswirkungen von Luftverschmutzung. Diese Erkenntnisse verdeutlichen die gesundheitlichen Kosten der Verzögerung und Ungleichheit in Minderungsmaßnahmen. Sie weisen außerdem auf die Millionen von Todesfällen hin, die sich jährlich durch den Übergang zu einer emissionsärmeren Zukunft, in der die Gesundheit aller Menschen priorisiert, verhindern lassen könnten.

Die Menschheit ist nicht auf dem richtigen Kurs, um die gesundheitlichen Vorteile des Übergangs zu einer emissionsarmen Wirtschaft zu realisieren. Die aktuellen globalen Verpflichtungen zur CO_2-Reduktion reichen nicht aus, um das Ziel des Abkommens von Paris zu erreichen. Der durchschnittliche globale Temperaturanstieg bis zum Ende dieses Jahrhunderts wird unter gegebenen Bedingungen ca. 2,4 °C betragen. Die Art und Weise, in der Ausgaben nach der COVID-19-Pandemie eingesetzt werden, droht, diese Situation weiter zu verschlimmern. Nur 18 % aller bis zum Ende des Jahres 2020 aufgebrachten Mittel werden Erwartungen zufolge zu einer Reduktion von Treibhausgasemissionen führen. Prognosen zufolge wird die wirtschaftliche Erholung nach der Pandemie im Jahr 2021 sogar zu einem noch nie dagewesenen Anstieg der Treibhausgasemissionen von 5 % führen. Damit erreichen die globalen vom Menschen produzierten Emissionen wieder die vergangenen Höchststände.

Außerdem ist die Umsetzung des Ziels, ab 2020 100 Milliarden US-Dollar pro Jahr für die Förderung von CO_2-Reduktion und Anpassungsmaßnahmen in den unterversorgtesten Ländern aufzubringen, aufgrund der aktuellen wirtschaftlichen Rezession in Gefahr. Allerdings ist diese Summe im Vergleich zu den Billionen, die für die wirtschaftliche Erholung von der Pandemie eingeplant sind, sehr klein. Die hohen Schulden, die Länder im Verlauf der Pandemie aufnehmen mussten, könnten deren Möglichkeit, eine grüne Erholung umzusetzen und die gesundheitlichen Vorteile der CO_2-Reduktion zu maximieren, eliminieren.

Eine nie dagewesene Chance, um eine gesunde Zukunft für alle Menschen sicherzustellen

Die Überschreitung der Emissionsziele aufgrund einer CO_2-intensiven Erholung nach der Pandemie würde die Welt unwiderruflich daran hindern, Klimaverpflichtungen einzuhalten und die internationalen Ziele für nachhaltige Entwicklung (Sustainable Developement Goals) zu erreichen. Für die Menschheit würde dies bedeuten von nun an mit einem zunehmend extremen und unvorhersehbarem Lebensraum zurechtkommen zu müssen. Die Daten dieses Berichts offenbaren die gesundheitlichen Auswirkungen und Ungleichheiten in der gegenwärtigen Welt bei einem Temperaturanstieg von 1,2 °C gegenüber der vorindustriellen Zeit. Angesicht der aktuellen Entwicklung ist davon auszugehen, dass der Klimawandel das entscheidende Kriterium für die menschliche Gesundheit werden wird.

Wenn die Billionensummen, die für die wirtschaftliche Erholung nach COVID-19 aufgebracht werden, gemäß den Vorgaben der Weltgesundheitsorganisation für eine gesunde und grüne Erholung eingesetzt werden, dann können die Ziele des Abkommens von Paris erreicht werden. Natürliche Systeme, die dem Gesamtwohl förderlich sind, könnten geschützt werden, Ungleichheiten könnten durch reduzierte gesundheitliche

Auswirkungen minimiert werden und die zusätzlichen Vorteile einer Transition zu einer emissionsarmen Gesellschaft könnten maximiert werden. Die Förderung einer gerechten Eindämmung des Klimawandels und universeller Zugang zu sauberer Energieversorgung könnten jährlich Millionen Todesfälle verhindern. Reduzierte Luftverschmutzung, gesunde Ernährung und aktivere Lebensweisen könnten helfen gesundheitsbezogene Ungleichheit überall auf der Welt zu reduzieren. Aktuell ist ein entscheidender Moment im Hinblick auf Konjunkturprogramme, welche eine historisch einmalige Gelegenheit darstellen, um die Gesundheit heutiger und zukünftiger Generationen zu schützen.

Einige vielversprechende Trends in den diesjährigen Daten deuten aber auf positive Veränderungen hin: So stieg der jährliche Durchschnitt von durch erneuerbare Wind- und Solartechnologien gewonnenem Strom zwischen 2013 und 2018 um 17 % an (Indikator 3.1); Investitionen in Kohlekapazitäten fielen im Jahr 2020 um 10 % ab (Indikator 4.2.1); und die Anzahl von Elektroautos in aller Welt betrug 2019 7,2 Millionen (Indikator 3.4). Des Weiteren hat die globale Pandemie das Interesse an den Themen Gesundheit und Klimawandel in den verschiedensten Teilen der Gesellschaft gesteigert. 91 Staats- und Regierungschefs erwähnten das Thema in der UN-Generaldebatte 2020 und in Ländern mit einem sehr hohen HDI ist ein neues und breites gesellschaftliches Engagement zu beobachten (Indikator 5.4). Ob die Erholung von der COVID-19-Pandemie diese Trends unterstützen oder umkehren wird, wird sich noch zeigen.

Weder COVID-19 noch der Klimawandel lassen sich von nationalen Grenzen aufhalten. Ohne breite, zugängliche Impfungen in allen Ländern und Gesellschaften werden SARS-CoV-2 und neue Varianten des Viruses die Gesundheit aller Menschen weiterhin gefährden. Genauso erfordert auch die Bekämpfung des Klimawandels, dass alle Länder umgehend und koordinierte Maßnahmen umsetzen. Hier müssen die Mittel zur wirtschaftlichen Erholung nach der Pandemie für die Unterstützung und Gewährleistung eines gerechten Übergangs zu einer emissionsarmen Zukunft, sowie einer weltweiten Klimawandelanpassung eingesetzt werden. Staats- und Regierungschefs bietet sich eine noch nie dagewesene Gelegenheit, um eine Zukunft mit einer besseren Gesundheit, weniger Ungleichheit und ökonomischer und ökologischer Nachhaltigkeit zu kreieren. Dies lässt sich nur erreichen, wenn die Welt zusammenarbeitet und sicherstellt, dass niemand zurückbleibt.
