# Supplementary material for: The 2021 report of the Lancet Countdown on health and climate change: code red for a healthy future
Source: Lancet. Author manuscript; Available in PMC 2024 Nov 15. (PMC7616807; doi:10.1016/S0140-6736(21)01787-6)
Supplement: Spanish translation of the Executive Summary [file EMS200003-supplement-Spanish_translation_of_the_Executive_Summary.pdf]

THE LANCET

Supplementary appendix

This translation in Spanish was submitted by the authors and we reproduce it as supplied. It has not been peer reviewed. *The Lancet*'s editorial processes have only been applied to the original in English, which should serve as reference for this manuscript.

Los autores nos proporcionaron esta traducción al español y la reproducimos tal como nos fue entregada. No la hemos revisado. Los procesos editoriales de *The Lancet* se han aplicado únicamente al original en inglés, que debe servir de referencia para este manuscrito.

Supplement to: Romanello M, McGushinA, Di Napoli C, et al. The 2021 report of the *Lancet* Countdown on health and climate change: code red for a healthy future. *Lancet* 2021; published online Oct 20. http://dx.doi.org/10.1016/S0140-6736(21)01787-6.

El informe de 2021 de *Lancet* Countdown sobre salud y cambio climático: código rojo para un futuro saludable

Marina Romanello, Alice McGushin, Claudia Di Napoli, Paul Drummond, Nick Hughes, Louis Jamart, Harry Kennard, Pete Lampard, Baltazar Solano Rodriguez, Nigel Arnell, Sonja Ayeb-Karlsson, Kristine Belesova, Wenjia Cai, Diarmid Campbell-Lendrum, Stuart Capstick, Jonathan Chambers, Lingzhi Chu, Luisa Ciampi, Carole Dalin, Niheer Dasandi, Shouro Dasgupta, Michael Davies, Paula Dominguez-Salas, Robert Dubrow, Kristie L Ebi, Matthew Eckelman, Paul Ekins, Luis E Escobar, Lucien Georgeson, Delia Grace, Hilary Graham, Samuel H Gunther, Stella Hartinger, Kehan He, Clare Heaviside, Jeremy Hess, Shih-Che Hsu, Slava Jankin, Marcia P Jimenez, Ilan Kelman, Gregor Kiesewetter, Patrick L Kinney, Tord Kjellstrom, Dominic Kniveton, Jason K W Lee, Bruno Lemke, Yang Liu, Zhao Liu, Melissa Lott, Rachel Lowe, Jaime Martinez-Urtaza, Mark Maslin, Lucy McAllister, Celia McMichael, Zhifu Mi, James Milner, Kelton Minor, Nahid Mohajeri, Maziar Moradi-Lakeh, Karyn Morrissey, Simon Munzert, Kris A Murray, Tara Neville, Maria Nilsson, Nick Obradovich, Maquins Odhiambo Sewe, Tadj Oreszczyn, Matthias Otto, Fereidoon Owfi, Olivia Pearman, David Pencheon, Mahnaz Rabbaniha, Elizabeth Robinson, Joacim Rocklöv, Renee N Salas, Jan C Semenza, Jodi Sherman, Liuhua Shi, Marco Springmann, Meisam Tabatabaei, Jonathon Taylor, Joaquin Trinanes, Joy Shumake-Guillemot, Bryan Vu, Fabian Wagner, Paul Wilkinson, Matthew Winning, Marisol Yglesias, Shihui Zhang, Peng Gong, Hugh Montgomery, Anthony Costello, Ian Hamilton

Resumen ejecutivo

Lancet Countdown es una colaboración internacional que monitorea de manera independiente las consecuencias sanitarias de un clima cambiante. Mediante la publicación de indicadores nuevos, actualizados y mejorados cada año, *Lancet* Countdown representa el consenso de investigadores líderes de 38 instituciones académicas y agencias de la ONU. Los 44 indicadores expuestos en este informe exponen un incesante aumento en los efectos del cambio climático sobre la salud humana y las consecuencias de la respuesta inconsistente y tardía de los países de todo el mundo sobre la salud – arrojando una clara llamada de alerta para acelerar acciones que pongan a la salud de las personas y del planeta como principal prioridad.

El informe del 2021 coincide con la 26ª Conferencia de las Partes de la Convención Marco de las Naciones Unidas sobre el Cambio Climático (COP26), en la que los países se enfrentan a la presión de hacer realidad el ambicioso Acuerdo de París: mantener el aumento de la temperatura promedio mundial en 1·5 °C, y movilizar los recursos económicos necesarios para que todos los países tengan una respuesta climática eficaz. Dichas negociaciones se desarrollan en el contexto de la pandemia de la COVID-19, una crisis sanitaria mundial que ha cobrado millones de vidas, ha afectado los medios de subsistencia y a las comunidades de todo el mundo, y ha dejado al descubierto profundas brechas y desigualdades en la capacidad mundial para hacer frente y responder a las emergencias sanitarias. Sin embargo, en su respuesta a ambas crisis simultáneas, el mundo tiene una oportunidad sin precedentes de garantizar un futuro saludable para todos.

Aumento de las desigualdades en un mundo que se calienta

Las temperaturas récord de 2020 dieron lugar a un nuevo máximo de 31.000 millones más de días-persona de exposición a olas de calor entre las personas mayores de 65 años y 626 millones más de días-persona que afectan a los niños menores de 1 año, en comparación con el promedio anual registrado entre 1986–2005 (indicador 1.1.2). En el 2021, las personas mayores de 65 años o menores de 1 año, junto con las personas en situación de desventaja social, fueron las más afectadas por las temperaturas récord de más de 40 °C reportadas en el mes de junio, en las zonas del noroeste del Pacífico de EE.UU. y Canadá, un acontecimiento que habría sido casi imposible sin el aporte antropogénico al cambio climático. Aunque la cantidad exacta de afectados no se conocerá aún por varios meses, cientos de personas murieron de forma prematura debido al calor. Además, las poblaciones de países con el índice de desarrollo humano (IDH) definido por la ONU como bajos y medios, han sufrido el mayor aumento de vulnerabilidad debido al calor durante los últimos 30 años. El riesgo para su salud se ha visto agravado por la escasa disponibilidad de mecanismos de refrigeración y espacios verdes urbanos (indicadores 1.1.1, 2.3.2 y 2.3.3).

Los trabajadores agrícolas de países con un IDH bajo y medio se encontraron entre los más afectados, por tener una mayor la exposición a temperaturas extremas. Este grupo perdió casi la mitad de las 295.000 millones de horas potenciales de trabajo totales perdidas debido al calor en 2020 (indicador 1.1.4). Estas horas perdidas pudieron tener consecuencias económicas devastadoras para estos trabajadores que se encuentran estado de vulnerabilidad continuo. Los datos del informe de este año muestran que la pérdida promedio de ingresos potenciales en los países del grupo de IDH bajo

equivalieron a 4–8 % del producto interno bruto nacional de estos países (indicador 4.1.3).

Mediante estos efectos, el aumento de las temperaturas promedio y la alteración de los regímenes pluviales, el cambio climático está empezando a revertir los años de avance en la lucha contra la inseguridad alimentaria e hídrica que todavía afecta a las poblaciones más desatendidas de todo el mundo, negándoles un aspecto esencial de la buena salud. Durante cualquier dado mes de 2020, sequías extremas afectaron hasta 19% de la superficie terrestre mundial, un valor que no había superado 13 % entre 1950 y 1999 (indicador 1.2.2). En paralelo a las sequías, el aumento de la temperatura está afectando al potencial de rendimiento de los principales cultivos básicos del mundo. En el 2020, en relación con la línea de base del 1981–2010, se observó una reducción del potencial productivo del 6.0% en el caso del maíz, de 3.0% en el caso del trigo de invierno, 5.4 % en el caso de la soja y de 1.8 % en el caso del arroz (indicador 1.4.1). Esto se traduce a un riesgo creciente de inseguridad alimentaria global.

Además de estos peligros para la salud, las condiciones ambientales cambiantes también están aumentando la idoneidad para la propagación de muchos patógenos transmitidos por el agua, el aire, los alimentos y los vectores. Aunque el desarrollo socioeconómico, las intervenciones de salud pública y los avances en la medicina han disminuido la carga global de la transmisión de enfermedades infecciosas, el cambio climático podría diezmar los esfuerzos realizados en materia de control y erradicación.

La cantidad de meses con condiciones adecuadas desde un punto de vista ambiental para la transmisión de la malaria (*Plasmodium falciparum*) aumentó en un 39% de 1950–59 a 2010–19 en zonas densamente pobladas de mayor altitud y con un IDH bajo. Este aumento en la aptitud ambiental supone una amenaza para las a las poblaciones altamente desfavorecidas de estas zonas, que estaban comparativamente más a salvo de la malaria que aquellas en tierras más bajas (indicador 1.3.1). El potencial epidémico del virus del dengue, el de Zika y el de chikungunya, que en la actualidad afectan principalmente a las poblaciones de América Central, América del Sur, el Caribe, África y el sur de Asia, aumentó a nivel mundial, con un incremento de la tasa reproductiva básica de 13 % para la transmisión por *Anopheles aegypti* y de 7 % para la transmisión por *Anopheles albopictus* en comparación con la década de 1950. El mayor aumento relativo de la tasa de reproducción básica de estos arbovirus se observó en los países del grupo de IDH muy alto (indicador 1.3.1); sin embargo, las personas del grupo de IDH bajo aún son las más vulnerables a estos arbovirus (indicador 1.3.2).

Se observaron resultados similares en relación con la aptitud ambiental para la transmisión de *Vibrio cholerae,* un patógeno el cual se estima que causa casi 100.000 muertes al año, principalmente entre poblaciones con escaso acceso al agua potable y al saneamiento. Entre 2003 y 2019, las zonas costeras aptas para la transmisión del *V cholerae* aumentaron significativamente en todos los grupos de los países del IDH; no obstante, los habitantes del grupo de países con IDH bajo enfrentan la mayor aptitud ambiental para esta enfermedad (indicador 1.3.1), y el 98 % de sus costas presentaron condiciones aptas para la transmisión del *V cholerae* en 2020.

Los riesgos simultáneos e interconectados que suponen los fenómenos meteorológicos extremos, la transmisión de enfermedades infecciosas y la inseguridad alimentaria, hídrica y financiera están sobrecargando a las poblaciones más vulnerables. Mediante múltiples riesgos simultáneos e interactivos, el cambio climático amenaza con revertir años de progreso en materia de salud pública y desarrollo sostenible.

Incluso ante las pruebas contundentes sobre las consecuencias sobre la salud humana del cambio climático, los países no están dando una respuesta de adaptación adecuada y proporcional a los crecientes riesgos a los que se enfrentan sus poblaciones. En el 2020, 104 (63 %) de 166 países no contaban un nivel alto de implementación de sus marcos nacionales de emergencias sanitarias, lo que los deja sin la preparación necesaria para responder a pandemias y emergencias sanitarias relacionadas con el clima (indicador 2.3.1). Es importante destacar que sólo 18 (55 %) de 33 países con un IDH bajo habían indicado que tenían al menos un nivel medio de implementación del marco nacional de emergencia sanitaria, en comparación con 47 (89 %) de 53 países con un IDH muy alto. Además, solo 47 (52 %) de 91 países informaron que contaban con un plan nacional de adaptación para la salud, y el principal obstáculo identificado para su implementación fue la falta de recursos económicos y humanos (indicador 2.1.1). Con un planeta que se enfrenta a un aumento inevitable de la temperatura, incluso si se implementara la más ambiciosa mitigación del cambio climático, la adaptación acelerada sería esencial para reducir la vulnerabilidad de las poblaciones en relación con el cambio climático y proteger la salud de las personas en todo el mundo.

Una respuesta desigual perjudica a todos

A diez meses de 2021, no se había producido el acceso global y equitativo a la vacuna contra la COVID-19: más del 60% de las personas de los países de ingresos altos han recibido al menos una dosis de la vacuna, en comparación con solo el 3,5% de las personas de los países de ingresos bajos. Los datos de este informe exponen desigualdades similares en lo que se refiere a la respuesta mundial para la mitigación del cambio climático.

Con el fin de cumplir con los objetivos del Acuerdo de París y evitar niveles catastróficos de calentamiento global, se deben reducir a la mitad las emisiones mundiales de gases de efecto invernadero en una década. No obstante, al ritmo actual, el sistema energético tardaría más de 150 años en descarbonizarse por completo (indicador 3.1), y la respuesta desigual entre países está dando lugar a una manifestación dispar de los beneficios

para la salud relacionados con la reducción de las emisiones.
El uso de fondos públicos para subsidiar los combustibles fósiles es responsable, en parte, del ritmo lento de descarbonización. En 2018, de los 84 países examinados, 65 países seguían proporcionando un subsidio global a los combustibles fósiles y, en muchos casos, los subsidios equivalían a proporciones sustanciales del presupuesto sanitario nacional y podrían haberse reorientado con el fin de obtener beneficios netos para la salud y el bienestar. Asimismo, los 19 países cuyas políticas de precios del carbono superaron el efecto de cualquier subsidio a los combustibles fósiles pertenecían al grupo de IDH muy alto (indicador 4.2.4).
Aunque los países del grupo de IDH muy alto son los que más han avanzado colectivamente en la descarbonización del sistema energético, todavía siguen siendo los principales contribuyentes a las emisiones de CO_2 a través de la producción local de bienes y servicios, lo que representa el 45 % del total mundial (indicador 4.2.5). Con un ritmo de descarbonización más lento y una normativa sobre la calidad del aire más pobre que la de los países del grupo de IDH muy alto, los grupos de países de IDH medio y alto producen la mayor cantidad de emisiones de partículas finas ($PM_{2,5}$) y tienen los índices más altos de muertes relacionadas con la contaminación atmosférica, que son aproximadamente 50% más altos que el total de muertes del grupo de IDH muy alto (indicador 3.3). El grupo de IDH bajo, con cantidades comparativamente menores de actividad industrial que las de los otros grupos, tiene una producción local que contribuye solo al 0,7 % de las emisiones mundiales de CO_2, y tiene la tasa de mortalidad más baja por contaminación atmosférica. Sin embargo, como solo el 12% de sus habitantes utilizan combustibles y tecnologías limpias para cocinar, la salud de estas poblaciones sigue estando en riesgo debido a las concentraciones peligrosas de contaminación atmosférica dentro de los hogares (indicador 3.2). Incluso en los países más prósperos, los habitantes de las zonas más desfavorecidas soportan de forma abrumadora la carga de los efectos para la salud relacionados con la exposición a la contaminación atmosférica. Estos resultados exponen los costos sanitarios de la respuesta de mitigación tardía y desigual, y subrayan los millones de muertes que se evitarían anualmente mediante una transición con bajas emisiones de carbono que dé prioridad a la salud de todas las poblaciones.
El mundo no está en camino de conseguir los beneficios para la salud que una transición a una economía baja en carbono supondría. Los compromisos actuales de descarbonización a nivel mundial son insuficientes para cumplir con los objetivos del Acuerdo de París y conducirían a un aumento de alrededor de 2,4ºC de la temperatura promedio mundial a finales del siglo. La dirección actual del gasto post-COVID-19 amenaza con empeorar esta situación, ya que se espera que solo el 18 % de todos los fondos destinados a la recuperación económica de la pandemia a finales de 2020 se traduzca en una reducción de las emisiones de gases de efecto invernadero. De hecho, se prevé que la recuperación económica de la pandemia provoque un aumento sin precedentes del 5% de las emisiones de gases de efecto invernadero en 2021, lo que ocasionará que las emisiones antropogénicas mundiales vuelvan a alcanzar sus niveles máximos.
Además, la actual recesión económica amenaza con socavar el objetivo de movilizar 100.000 millones de dólares anuales a partir de 2020 para promover cambios en las emisiones de carbono y respuestas de adaptación en los países más desatendidos, aunque este monto es ínfimo comparado con los billones destinados a la recuperación de la COVID-19. Las grandes cantidades de préstamos a las que han tenido que recurrir los países durante la pandemia podrían acabar con su capacidad de lograr una recuperación sostenible, y de maximizar los beneficios para la salud de su población relacionados con una transición a un sistema de cero emisiones.

Una oportunidad sin precedentes de garantizar un futuro saludable para todos.

Una recuperación de la pandemia de COVID-19 basada en el carbono resultaría en un aumento acelerado de emisiones, que impediría de forma irreversible que el mundo cumpla los compromisos climáticos, así como los Objetivos de Desarrollo Sostenible – encerrando a la humanidad en un ambiente cada vez más extremo e impredecible. Los datos de este informe presentan los impactos en la salud y las desigualdades sanitarias del mundo actual con 1,2ºC de calentamiento por encima de la época preindustrial. Los datos sugieren que, en la trayectoria actual, el cambio climático se convertirá en la narrativa definitoria de la salud humana.
No obstante, si se orientaran los billones de dólares que se destinarán a la recuperación de la COVID-19 hacia las propuestas de la OMS para una recuperación saludable y sostenible, el mundo podría cumplir los objetivos del Acuerdo de París, proteger los sistemas naturales que sustentan el bienestar, y minimizar las desigualdades mediante la reducción de los efectos para la salud y la maximización de los cobeneficios de una transición global a un sistema de bajas emisiones. Promover la mitigación equitativa del cambio climático y el acceso universal a las energías limpias podría evitar millones de muertes anuales gracias a la reducción de la exposición a la contaminación atmosférica, a dietas más saludables y a estilos de vida más activos, y contribuiría a reducir las desigualdades sanitarias a nivel mundial. Este momento crucial de estímulo económico representa una oportunidad histórica de garantizar la salud de las generaciones presentes y futuras.
En los datos de este año, hay un destello de cambio positivo que se aprecia en varias tendencias prometedoras: la generación de electricidad a partir de energía renovable eólica y solar aumentó un promedio anual de 17 % entre 2013 y 2018 (indicador 3.1), la inversión en nueva capacidad de carbón disminuyó 10% en 2020 (indicador 4.2.1) y la cantidad mundial de

vehículos eléctricos alcanzó los 72 millones en 2019 (indicador 3.4). Además, la pandemia mundial ha impulsado un mayor compromiso con la salud y el cambio climático en múltiples ámbitos de la sociedad, por ejemplo, con la participación de 91 jefes de Estado en el Debate General de la ONU de 2020 y un compromiso recientemente extendido entre los países del grupo de IDH muy alto (indicador 5.4). Todavía está por verse si la recuperación de la COVID-19 favorece o contrarresta estas tendencias.

Ni la COVID-19 ni el cambio climático respetan las fronteras nacionales. Sin una vacunación universal y accesible en todos los países y sociedades, el SARS-CoV-2 y sus nuevas variantes seguirán poniendo en riesgo la salud de todos. Asimismo, la lucha contra el cambio climático requiere que todos los países den una respuesta urgente y coordinada, con la asignación de fondos de recuperación en relación con la COVID-19 para apoyar y garantizar una transición justa hacia un futuro con bajas emisiones de carbono y la adaptación al cambio climático en todo el planeta. Los líderes mundiales tienen una oportunidad sin precedentes de ofrecer un futuro con mejor salud, menos desigualdades, y sostenibilidad económica y ambiental. No obstante, solo será posible si el mundo actúa en conjunto para garantizar que nadie se quede atrás.
