# Supplementary material for: The 2021 report of the Lancet Countdown on health and climate change: code red for a healthy future
Source: Lancet. Author manuscript; Available in PMC 2024 Nov 15. (PMC7616807; doi:10.1016/S0140-6736(21)01787-6)
Supplement: Supplementary appendix 5 [file EMS200003-supplement-Supplementary_appendix_5.pdf]

THE LANCET

Supplementary appendix

This appendix formed part of the original submission and has been peer reviewed. We post it as supplied by the authors.

Supplement to: Romanello M, McGushinA, Di Napoli C, et al. The 2021 report of the *Lancet* Countdown on health and climate change: code red for a healthy future. *Lancet* 2021; published online Oct 20. http://dx.doi.org/10.1016/S0140-6736(21)01787-6.

The 2021 Report of the *Lancet* Countdown on Health and Climate Change

Appendix

Table of Contents

Section 1: Climate Change Impacts, Exposures, and Vulnerability 3
Indicator 1.1.1: Vulnerability to Extremes of Heat 5
Indicator 1.1.2: Exposure of Vulnerable Populations to Heatwaves 6
Indicator 1.1.3: Heat and Physical Activity 9
Indicator 1.1.4: Change in Labour Capacity 11
Indicator 1.1.5: Heat and Sentiment 16
Indicator 1.1.6: Heat-Related Mortality 20
Indicator 1.2.1: Wildfires 23
Indicator 1.2.2: Drought 25
Indicator 1.2.3: Lethality of Extreme Weather Events 28
Indicator 1.3.1: Climate Suitability for Infectious Disease Transmission 33
Indicator 1.3.2: Vulnerability to Mosquito-Borne Diseases 46
Indicator 1.4.1: Terrestrial Food Security and Undernutrition 48
Indicator 1.4.2: Marine Food Security and Undernutrition 51
Indicator 1.5: Migration, Displacement and Rising Sea Levels 72
Section 2: Adaptation, Planning, and Resilience for Health 79
Indicator 2.1.1: National Adaptation Plans for Health 79
Indicator 2.1.2: National Assessments of Climate Change Impacts, Vulnerability, and Adaptation for Health 81
Indicator 2.1.3: City-Level Climate Change Risk Assessments 83
Indicator 2.2: Climate Information Services for Health 87
Indicator 2.3.1: Detection, Preparedness and Response to Health Emergencies 90
Indicator 2.3.2: Air Conditioning Benefits and Harms 92
Indicator 2.3.3: Urban Green Space 103
Indicator 2.4: Global Funding and Financial Transactions Related to Health Adaptation 108
Section 3: Mitigation Actions and Health Co-Benefits 111
Indicator 3.1.1: Carbon Intensity of the Energy System 111
Indicator 3.1.2: Coal Phae-Out 114
Indicator 3.1.3: Zero-Carbon Emission Electricity 116
Indicator 3.2: Clean Household Energy 118
Indicator 3.3: Mortality from Ambient Air Pollution by Sector 122
Indicator 3.4: Sustainable and Healthy Transport 124
Indicator 3.5.1: Emissions from Agricultural Production and Consumption 126
Indicator: 3.5.2 Diet and Health Co-Benefits 132
Indicator 3.6: Mitigation in the Healthcare Sector 141
Section 4: Economics and Finance 143
Indicator 4.1.1: Economic Losses due to Climate-Related Extreme Events 143
Indicator 4.1.2: Monetized value of Heat-related Mortality 146
Indicator 4.1.3: Potential Loss of Earnings from Heat-Related Labour Capacity Reduction 148
Indicator 4.1.4: Costs of the Health Impacts of Air Pollution 155
Indicator 4.2.1: Clean energy investment 158
Indicator 4.2.2: Employment in Low-Carbon and High-Carbon Industries 160
Indicator 4.2.3: Funds Divested from Fossil Fuels 162
Indicator 4.2.4: Net Value of Fossil Fuel Subsidies and Carbon Prices 164
Indicator 4.2.5: Production and Consumption-based Attribution of CO_2 and $PM_{2.5}$ Emissions 168
Section 5: Public and Political Engagement 175
Indicator 5.1.1: Global Coverage of Health and Climate Change 175
Indicator 5.1.2: Coverage of Health and Climate Change in China's People's Daily 185
Indicator 5.1.3: Content of Coverage in US and Indian Newspapers 193
Indicator 5.2: Individual Engagement in Health and Climate Change 199
Indicator 5.3: Coverage of Health and Climate Change in Scientific Journals 221
Indicator 5.4: Government Engagement in Health and Climate Change 235
Indicator 5.5: Corporate Engagement in Health and Climate Change in the Healthcare Sector 252
References 268

Section 1: Climate Change Impacts, Exposures, and Vulnerability

1.1: Heat and Health

Health and Exposure to Warming

Methods

The methodology for this indicator has been updated from the 2020 report of the Lancet Countdown.[1] The indicator uses monthly temperature from European Centre for Medium-Range Weather Forecasts (ECMWF) ERA5 climate reanalysis dataset. From this, a baseline global mean temperature grid was first calculated as the average of summer temperatures (June, July, August for the northern hemisphere, December, January, February for the southern hemisphere) from 1986-2005, the same period used by the Intergovernmental Panel on Climate Change (IPCC AR5[2,3]). Then global summer temperature changes relative to the 1986-2005 average were calculated for every grid point for every year. The 'population-weighted' average was calculated by weighting each grid cell by the fraction of the total world population contained within that grid cell. New to 2021, population data from 2000 to present are from NASA GPWv4 at 0.25° x 0.25° spatial resolution, the same as ECMWF ERA5. Population data from 1980 to 2000 are from the ISIMIP Histsoc dataset at 0.5° x 0.5° spatial resolution. This is an improvement from previous reports where the population data had had a coarser spatial resolution throughout the considered time period.

Data

1. Climate data from the European Centre for Medium-Range Weather Forecasts (ECMWF) ERA5 reanalysis.[4]
2. Population data from the NASA Socioeconomic Data and Applications Center (SEDAC) Gridded Population of the World (GPWv4) and The Inter-Sectoral Impact Model Intercomparison Project (ISIMIP) Histsoc dataset.[5,6]

Future form of the indicator

Future development of this indicator will include the anticipated extension of the ECMWF ERA5 dataset back to 1950, which will allow a longer time series to be analysed.

Additional analysis

The change in summer temperature relative to the 1986–2005 average is presented (Figure 1). In 2020 the upward trend in both the global mean summer temperature anomaly relative (+0.34˚C) and population weighted summer temperature anomaly (+0.60˚C) continued.

Figure 2 maps the mean summer temperature anomaly for 2020 and highlights warmer-than-average temperatures across much of the globe, with regions such as south-western U.S.A. and north-western Siberia particularly above average.[7]

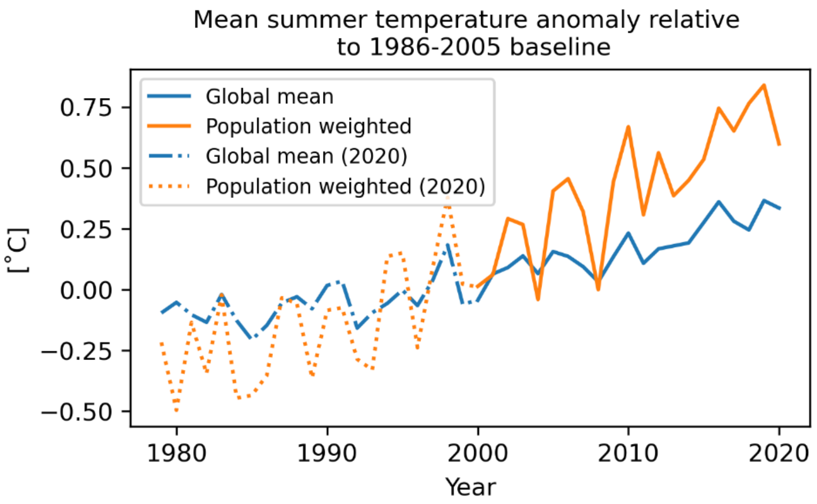

Figure 1. Global mean trends of summer temperature anomaly compared to the population weighted trend (relative to the 1986-2005 baseline).

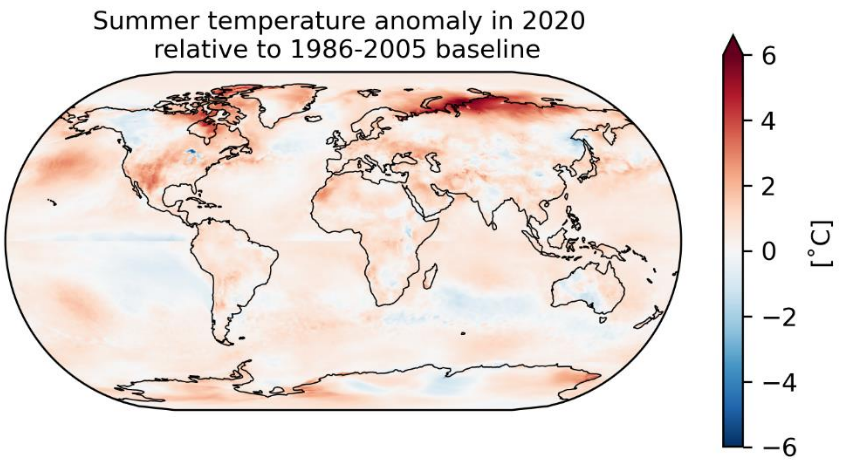

Figure 2. Map of summer temperature anomaly for 2020 relative to the 1986-2005 baseline.

Indicator 1.1.1: Vulnerability to Extremes of Heat

Methods

This indicator displays an index derived by taking mean of proportion of the population over 65 years (1)[8]; the prevalence of cardiovascular, diabetes and chronic respiratory diseases among population over 65 years from the GBD study 2019 estimates (2)[9] and the proportion of the population living in urban areas (3)[10] as a measure of exposure to urban heat island. The index ranges between 0 and 100 and is a measure of potential vulnerability of a country to heat exposure. Aggregated trends were displayed by WHO regional classifications and UNDP human development index (HDI) for the period 1990 to 2019.

Data

1. Global Burden of Disease Study 2019. Seattle, United States: Institute for Health Metrics and Evaluation (IHME), 2020. Available from http://ghdx.healthdata.org/gbd-results-tool.[11]
2. The United Nations Population Division's World Urbanization Prospects.[12]

Caveats

There is no consistent and universally accepted standard for distinguishing urban from rural areas, in part because of the wide variety of situations across countries. Most countries use an urban classification related to the size or characteristics of settlements.[12] This indicator does not include the existence of heat early warning systems, or prevalence of cooling devices. Neither does it include the prevalence of green areas in cities.

Future Form of Indicator

The inclusion of further indicators of population vulnerability as well as heat adaptation measures will be explored for future iterations of this indicator.

Additional analysis

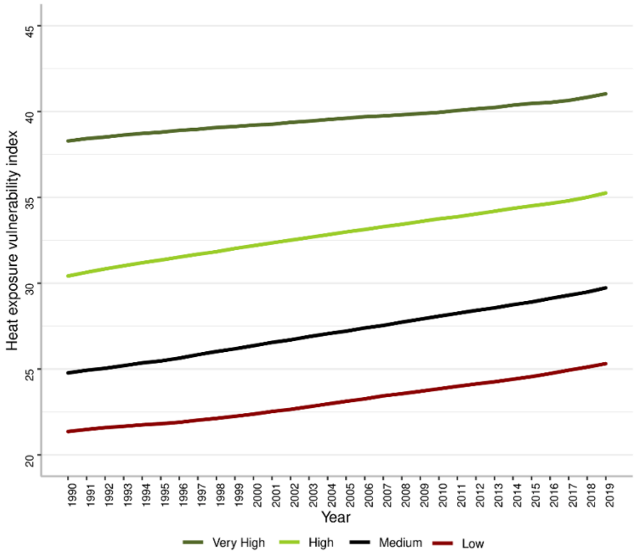

Figure 3. Change in the vulnerability to heat index grouped by 2019 country Human Development Index level for 1990-2019. This index includes the proportion of the population older than 65 years, the prevalence of relevant chronic diseases (respiratory disease, cardiovascular disease, and diabetes) in persons aged over 65 years, and the proportion of the total population living in urban areas

Indicator 1.1.2: Exposure of Vulnerable Populations to Heatwaves

Methods

The methodology and input data for this indicator have been improved and extended for the 2021 report.

A new heatwave definition has been adopted which defines a heatwave as a period of at least two days where both the daily minimum and maximum temperatures are above the 95th percentile of their respective climatologies. This reflects the definition from the World Meteorological Organization as well as from published scientific literature on the topic.[13,14] It also aims to capture the health effects of both direct heat extremes (i.e. caused by high maximum temperatures) and the problems associated with lack of recovery (i.e. caused by high minimum temperatures) over persisting hot periods.[15]

The gridded 95th percentile of daily minimum and maximum temperatures, taken from the European Centre for Medium-Range Weather Forecasts (ECMWF) ERA5 dataset, were calculated on a 0.25° x 0.25° global grid for 1986-2005. For each year from 1980 to 2020, the number of heatwave events and total days of heatwaves per year was calculated according to the definition above.

Inspection of the data has shown that increasing heatwave length can result in fewer discrete heatwave events as they merge into single long events – this is therefore better captured by the person-days metric. In continuity with previous reports, the exposure of vulnerable populations to heatwaves was therefore computed as person-days, i.e. as number of days of heatwave times the number of people affected. This captures the changes in duration as well as in frequency of heatwaves.

Population and demographic data from NASA GPWv4 were used for the period 2000-2020 as their resolution matches ECMWF ERA5's. For the period pre-2000, the ISIMIP Histsoc dataset was used after been upsampled to a 0.25° x 0.25° resolution via a 2D linear interpolation of population densities with land area data from NASA GPWv4. Both NASA GPWv4 and ISIMIP Histsoc datasets refer to population older than 65 years old.

New to 2021, the indicator aims to investigate exposure to heatwaves also in infants, i.e. children under one year of age. This population is also known to be vulnerable to heatwaves.[16]

The number of births minus the mortality rate of children under 1 was used as an approximation of the number of children under 1 year old. The United Nation World Population Prospects (UN WPP) data for birth rates were used. UN WPP provide Crude Birth Rate (CBR) and Infant Mortality Rate (IMR) values per country as averages for 5-year periods. To estimate the spatial distribution of births within a country, it was assumed that the spatial distribution of children under one year of age was the same as the spatial distribution of children under 5 as given by the NASA GPWv4 dataset. It was furthermore assumed that the IMR within a country was constant for all locations, as sub-national data was not able to be applied for this study. For each country, the total number of births was calculated for the mid-period year of the 5-year time periods as *Country population * CBR * (1 - IMR)*. Spatial weighting matrices were derived from the NASA GPWv4 demographic data for under-5s. This was used to estimate the total births number of infants for each grid cell for each country. Finally, the estimates for the years in between the mid-period years were calculated through linear interpolation.

Data

1. Climate data from the European Centre for Medium-Range Weather Forecasts (ECMWF) ERA5 reanalysis.[17]
2. Population data from the NASA Socioeconomic Data and Applications Center (SEDAC) Gridded Population of the World (GPWv4) and The Inter-Sectoral Impact Model Intercomparison Project (ISIMIP) Histsoc dataset.[5,6]
3. Demographic data from the United Nation World Population Prospects (UN WPP).[18]

Caveats

As two distinct sources were used for population data, there may be some inconsistencies between the pre and post 2000 values. Therefore, the indicator is presented as exposure to change rather than change in exposure, as this avoids calculating changes in population across the data discontinuity. Furthermore, the interpolation of

ISIMIP Histsoc dataset to a finer spatial resolution introduces some additional uncertainty and risk of data errors (i.e., pixels with incorrect values). For sake of clarity pre-2000 results are highlighted in the figures reported in the following section.

With respect to the spatial distribution of children under one year of age, the analysis assumes that the distribution of under-5s and under-1s is the same, and that the IMR rate is constant per country. It is known however the mean IMR can vary significantly between regions within one country, addressing this limitation would require much more detailed demographic data at the sub-national level, which is not currently available in a globally harmonised form.

Future form of the indicator

Future iterations of this indicator could take advantage of the extension of the ERA5 dataset back to 1950.

Additional analysis

Improvements for the 2021 indicator mean that numbers are not directly comparable with the results of the previous Lancet Countdown publications. All years have therefore been re-calculated for the current publication.

In 2017-2020 Lancet Countdown publications, only minimum temperatures were used, the 99th percentile of temperatures were taken as the minimum threshold, and a period of 3 or more days was required. The additional conditions on maximum temperatures introduced for the 2021 report tend to reduce the number of conditions that are considered to be heatwaves. On the other hand, the reduction in the percentile threshold and number of days threshold tend to increase this number. This can be seen when comparing the new results with those of the 2020 report (Figure 4, Figure 5). The new absolute indicator values are about 30% higher than when using the previous definition. However, the overall trends are very similar, especially in highlighting the large increase in exposures in the post-2010 period.

Mapping the change in number of heatwaves in 2020 (Figure 4) highlights the large areas affected by heatwaves, notably the heatwave that affected California and the western USA. Figure 5 shows that, while significant, the total exposure of new-borns is much smaller than for over-65s - in large part due simply to the much larger size of the over-65 age band (encompassing over 35 years of demographics) compared to the new-borns (only 1 year). Indeed, Figure 6 shows the days of exposure normalised by the total number of humans in the corresponding demographic and shows that the trends for over-65s and under-1s are almost identical. However, there is a significant difference in the geographic distribution of the impacts for these two age groups, as highlighted in the figure in the main text of the report.

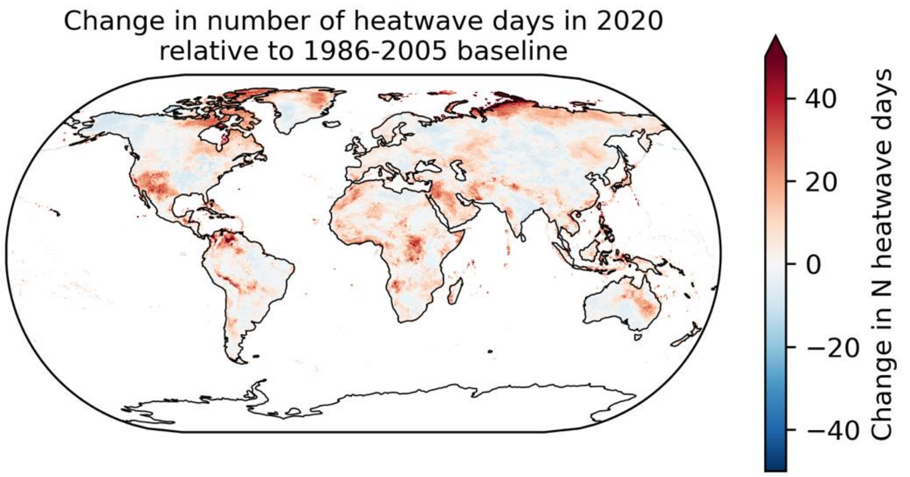

Figure 4. Map of the change in number of heatwave days over land in 2020 relative to the 1986-2005 baseline.

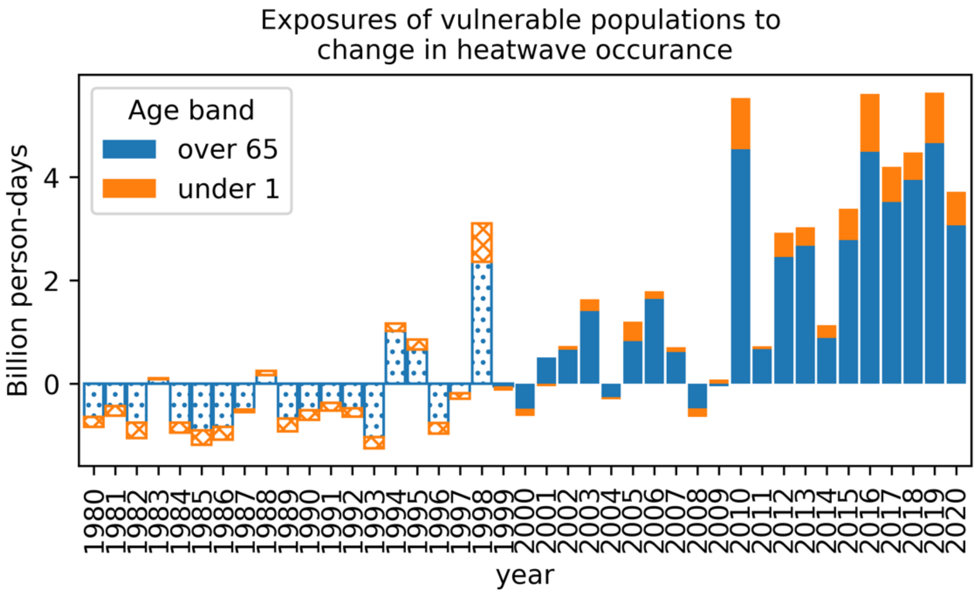

Figure 5. Exposure of people over 65 and infants under 1 year old to change in number of heatwave days relative to the 1986-2005 baseline mean number of days. Hatched bars indicate calculations using population data from ISIMIP for 1980-2000. Block shaded bars indicate calculations using GPWv4 for 2000-2020.

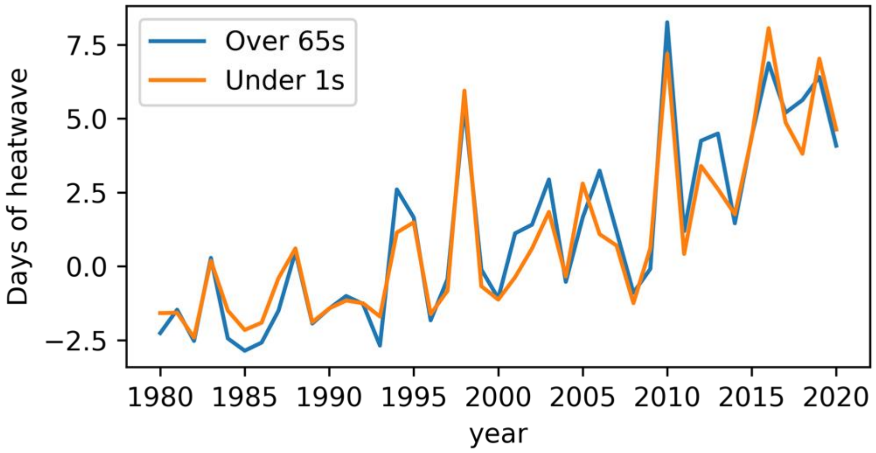

Figure 6. Comparison of time series of change in weighted number of days of heatwave experienced by people over 65 and infants under 1 year old relative to the 1986-2005 baseline, using population data from ISIMIP for 1980-2000 and from GPWv4 for 2000-2020.

Indicator 1.1.3: Heat and Physical Activity

Methods

Wet bulb globe temperature (WBGT) based on hourly temperature and dew point temperature was calculated and stored in the European Centre for Mid-Range Weather Forecasts' ERA5 database. Historical climate records are available from 1979, and for the purposes of this analysis data from the years 1991 to 2020 were considered. The number of hours with a recorded WBGT above 28ºC was tabulated for each year from 1991 to 2020 and for available grid cell. 28ºC is a threshold above which the national, sports medicine authorities of the USA, Australia, and Japan recommend outdoor physical activities are done with discretion.[1920-22]

The resulting metric, total activity hours lost per year in each grid cell, was then weighted by population. Population weighting was performed by multiplying the number of activity hours lost by the population in the respective grid cell. The population-weighted potential activity hours lost in a single year were added up for all grid cells in a given HDI country group, and this value was divided by the total population in this country group and by 365 to calculate the activity hours lost per person (AHLpp) per day.

Data

1. The climate data used for this study were obtained from the European Centre for Medium-Range Weather Forecasts' ERA5 database, which provides global coverage at the resolution of 0.25x0.25 degree grid cells. Reanalysis data from the years 1991 to 2020 were included.[23]
2. Grid cell-based population data were obtained from NASA's Socioeconomic Data and Applications Center, hosted by the Center for International Earth Science Information Network at Columbia University, New York.[24]

Caveats

Setting 26ºC as the WBGT cutoff precluded robust analysis of regions where this threshold is not typically exceeded. We also acknowledge that simple heat indices like WBGT do not fully capture the effect on exercise capacity of determinants such as age, physiology, and clothing,[25] and that a more robust index would incorporate these inter-individual factors.

Future forms of the indicator

We will continually update our results based on each new year of available climate data, and as more regional sports authorities issue their own guidelines for WBGT thresholds, we will be able to explore the results of setting our cutoff at different points.

Additional analysis

While countries were grouped by HDI for the purposes of this analysis, it is also possible to classify our indicator according to other categories, such as climate regions. An additional analysis was performed where average AHLpp was calculated for tropical, subtropical and temperate regions, as well as the global average, from the years 1980 to 2020 (Figure 7. Average potential activity hours lost per person (AHLpp) per day in three climate regions, 1980-2020. Rates of increase for each region are denoted by the slopes, while the x represents one-year interval increases.). The rates of increase for AHLpp were 0.34 hours per decade in tropical regions and 0.18 hours per decade globally. The rate of increase in tropical regions was nearly double that of the average global rate. These results are consistent with those of the HDI groups, as there is broad overlap between low and medium HDI countries and tropical climates.

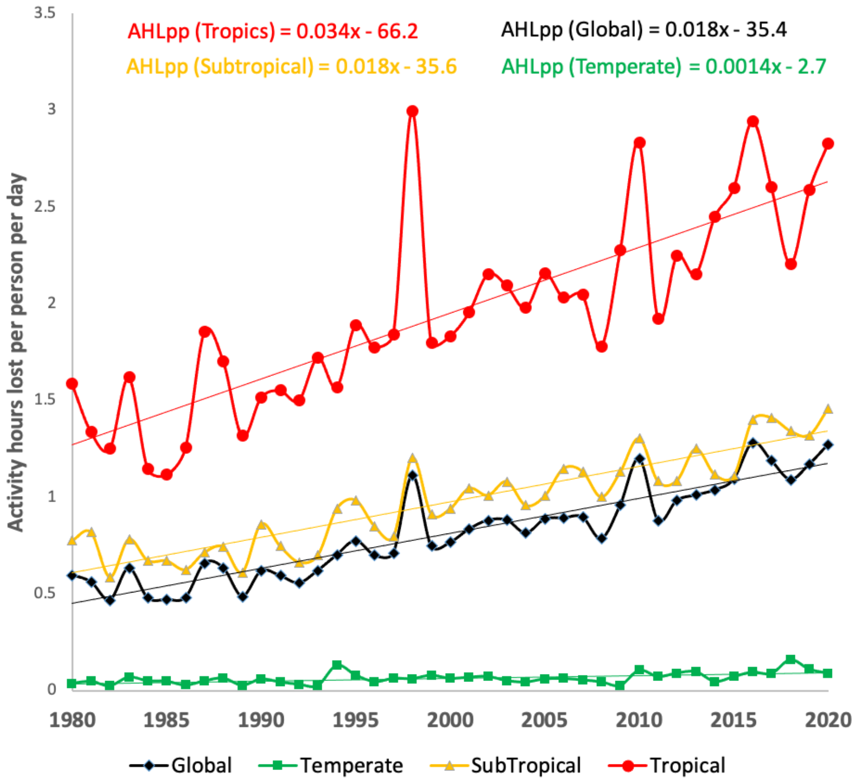

Figure 7. Average potential activity hours lost per person (AHLpp) per day in three climate regions, 1980-2020. Rates of increase for each region are denoted by the slopes, while the x represents one-year interval increases.

Indicator 1.1.4: Change in Labour Capacity

Methods

The methodology for this indicator has been improved from the 2020 report of the Lancet Countdown.[1]

It is based on 68,940 grid cell data (0.5 x 0.5 degrees with boundaries exactly on the degree line and half degree line) for climate and population. The focus is on trends since the end of the 20th century and on a method that can calculate labour capacity loss at country level. The model data chosen for the calculations was the ERA5 reanalysis hourly data on single levels (3A edition downloaded July 2020), and the analysis method is described in detail in the paper by Kjellstrom et al., 2018.[26]

Analysis starts from hourly estimates of temperature (t2m) and dew point (d2m). These inputs are used to derive hourly estimates of the heat stress index Wet Bulb Globe Temperature (WBGT) and work loss factor (WLF) at three different metabolic rates under the assumptions that workplace heat exposure is in the shade and air movement is 1 m/s (approximately the speed at which arms and legs move during work). The data was aggregated to provide estimates of mean annual WBGT and mean annual WLF between the hours of 7 am - 7 pm local solar time for each grid-cell.

Exposure was assumed to be atmospheric heat in the shade or indoors (i.e. incoming heat radiation from the sun is absent) without effective air conditioning. The impact of heat on labour capacity depends on clothing (assuming light clothing for all) and metabolic rate based on physical work activity. The methodology considers 3 metabolic rates: 200W (light work, sitting or moving around slowly), 300W (medium intensity work) and 400W (heavy labour).

The function relating WLF (the fraction of work hours lost) to an hourly WBGT level is given by the cumulative normal distribution (ERF) function:

$$Loss\ fraction = \frac{1}{2}\left(1 + \mathrm{ERF}\left(\frac{\mathrm{WBGThourly} - \mathrm{WBGTaver}}{\mathrm{WBGT}_{\mathrm{SD}} * \sqrt{2}}\right)\right)$$

where WBGTaver and $\mathrm{WBGT}_{\mathrm{SD}}$ are the parameters (Table 1) in the function for a given activity level.

Table 1. Input values for labour loss fraction calculation

Metabolic rate	WBGTaver	$\mathrm{WBGT}_{\mathrm{SD}}$
200 Watts	35.533	3.948
300 Watts	33.492	3.948
400 Watts	32.465	4.1607

For each grid cell, the working age population (15+ years old, from UN demographic data) for each time period is used as input data as well as the percentages of people in this age range working in 4 sectors: agriculture, construction, manufacturing and “other” sectors, which include the service sector (based on ILOSTAT data). Populations in grid cells that overlap country borders have been apportioned to the countries involved based on population distribution within the cell.

The total yearly work hours lost for each sector and country are calculated by first, for each grid cell, multiplying each employment sector population by the relevant work loss factor and then, second, summing the resulting sector work hours lost over all grid locations in each country (and in all countries together).

For the total-shade work hours lost (WHL), ILO sector proportions rates are assigned to the metabolic rate as shown in Table 2:

Table 2. Employment sector to metabolic rate assignment

Metabolic rate	200W	300W	400W
Employment sector	Other	Manufacturing	Agriculture + Construction

All agricultural and construction work was assumed to be in the shade - the lower bounds of potential work hours lost.

Improved in 2021, work hours lost per person were estimated for only the employed population older than 15 years, unlike in earlier reports where these were related to the entire population in that age group. Specifically, the work hours lost per person (WHLpp) are arrived at by dividing the WHL for each year by the number of employed people globally (or per country). Employed people here are defined as the population age 15 and over that works in any of the 4 ILO sectors mentioned above. The calculations is:

Employed People = Σ(for each grid-cell and year): Pop15plus * (Agr% + Manuf% + Constr% + Other%) * CountryPop%

Data

1. Climate data from the European Centre for Medium-Range Weather Forecasts (ECMWF) ERA5 reanalysis.[17]
2. Population data from the NASA Socioeconomic Data and Applications Center (SEDAC) Gridded Population of the World (GPWv4).[5]
3. Sector employment data from ILOSTAT.[27]

Caveats

The distribution of agricultural, construction, manufacturing and other sector workers is only reported at country level, hence this proportion is distributed evenly to all grid cells within each country and thus does not capture the geographical differences in the proportion of people working in the different sectors.

Analysis performed with the above-described methodology has shown that the ERA5 data regularly understates temperatures, particularly maximum air temperatures. The ERA5 deviation from the ensemble average varies by location, especially pronounced in coastal regions, and is generally in the order of 1-4°C lower. Given the nature of the calculation method (risk curve shape, see above), small temperature variations have a disproportional effect on WHL estimations. Combined with often high population concentrations near the coast the WHL results presented here are conservative. As a comparison, when applying the WHL calculations to climate data input sourced from ISIMIP, show that the ERA5-based calculation underestimates WHL by 40%.

Future form of the indicator

The calculations for 2020 do not consider the changes in labour structure that may have resulted from lockdowns during the COVID pandemic, and from the associated economic crisis. Future versions of this indicator will aim to capture this effect.

This indicator will be updated in future to include WBGT and WHL *in the sun*, which can be associated with 400W outdoor labour, like agriculture and construction.

Additional analysis

The global distribution of work hours lost (WHL) in the four sectors is shown in Figure 8. Agriculture dominates but stays largely constant due to reductions of the agricultural workforce in many low- and middle-income countries. The impact of rising heat is increasing the fastest in construction and *other* sectors (mainly in the service industry).

Because of its definition this indicator is influenced by the changes in population numbers and the distribution of the workforce within countries as well as climate change. WLF (work loss factor) is defined as the fraction of work hours lost for one worker at a specific metabolic rate, and thus describes work capacity loss due to heat independently from population and employment statistics. Figure 9 shows global WLF trends attributable to climate alone.

Country-specific WHL trends vary greatly depending on whether demographic trends are included. For example, between 1990 and 2020 the WHL in India (where the most losses occur) without population or sector changes

since 1990 increased from 57 to 84 billion hours (+46%), rather than to 113 billion hours (+98%) if population and sector changes are included.

Most of the global WHL results in this report are the total-shade WHL, which include effects of climate, population and workforce sector trends. By keeping populations, sector distributions, or both, constant at 1990 values (in the calculation as described above), one can compare the effects of climate change alone with climate and population, climate and sector distributions and the full complement of all factors (see Figure 8). The climate alone (orange) curve has the second steepest slope (~12% increase per decade). Adding the influence of population growth further increased the slope (grey curve). The blue curve shows the combined WHL trend (climate & sector & population) and has been used for the WHL figures stated in this report (if not specified otherwise). Even with population growth included this curve has a lower trend than climate-only, as in most countries the proportion of agricultural workers greatly diminishes in favour of the "Other" category, which comprises predominantly the service industry. This category is assigned to the lowest metabolic rate this this method (see above) and thus contributes much less to total WHL than the agriculture and construction workforce.

Some features of labour capacity trends were analysed for groups of countries classified by their HDI level in 2019.[28] An example is shown in Table 3, showing the potential annual work hours lost per employed person (WHLpep) in different country groups. The latitude categories are based on main areas of countries being in tropical, sub-tropical or temperate zones. As India and China have very large populations, they influence the means for the subtropical-medium HDI group (India with 216 WHLpep) and the subtropical-high HDI group (China with 36 WHLpep). The medium level HDI countries have the highest WHLpep and the very high category is least affected. However, a confounding factor is latitude and a very strong impact related to latitude is seen in very high-tropical countries, which reflects the naturally occurring high heat levels in those countries.

Table 3.Annual potential work hours per employed person lost (WHLpp) due to heat in 2020.

Latitude	Low HDI	Medium HDI	High HDI	Very high HDI	All countries
Tropical;<br>< 23.4°	93	200	93	114	146
Subtropical;<br>23.4-40°	18	184	35	17	42
Temperate;<br>> 40°	0	0	2	1	1
All countries	89	198	54	14	89

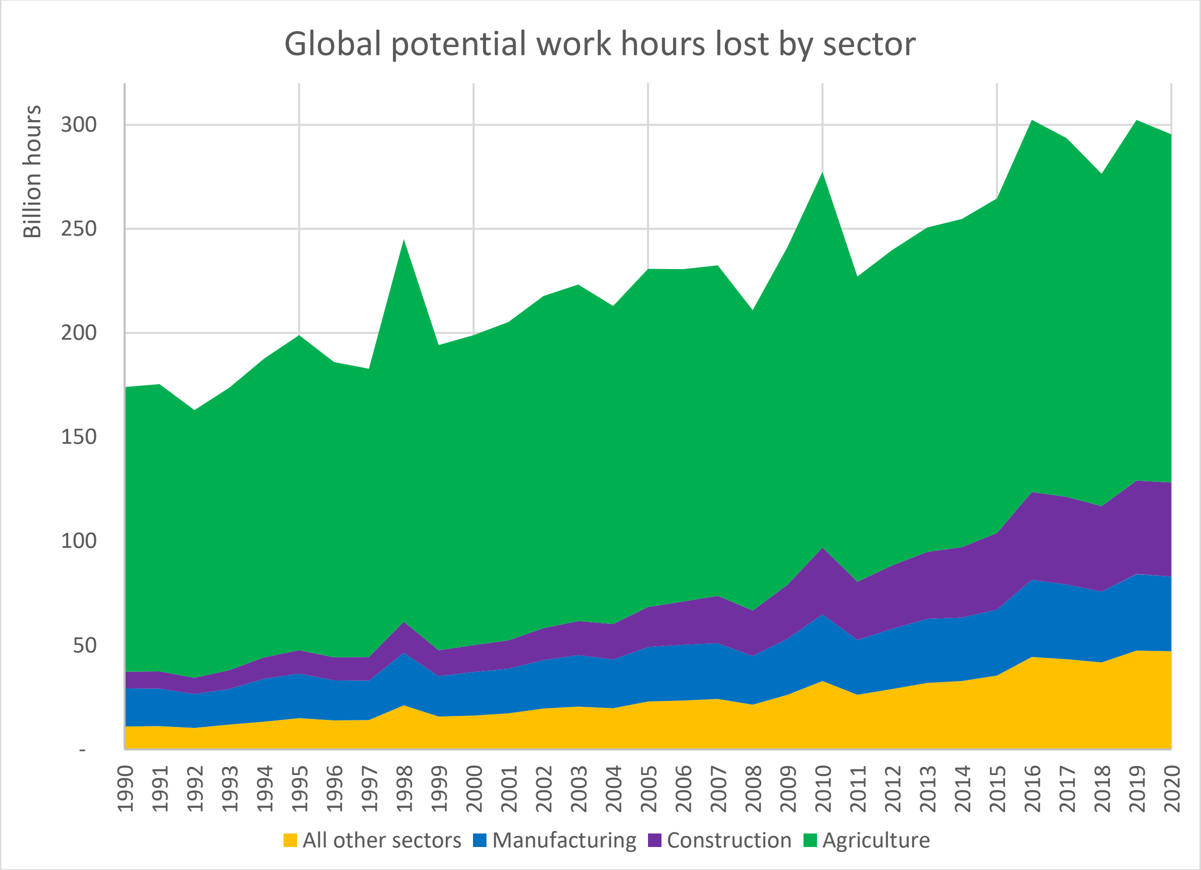

Figure 8. Global potential work hours lost (billions) due to heat by employment sector, 1990-2020.

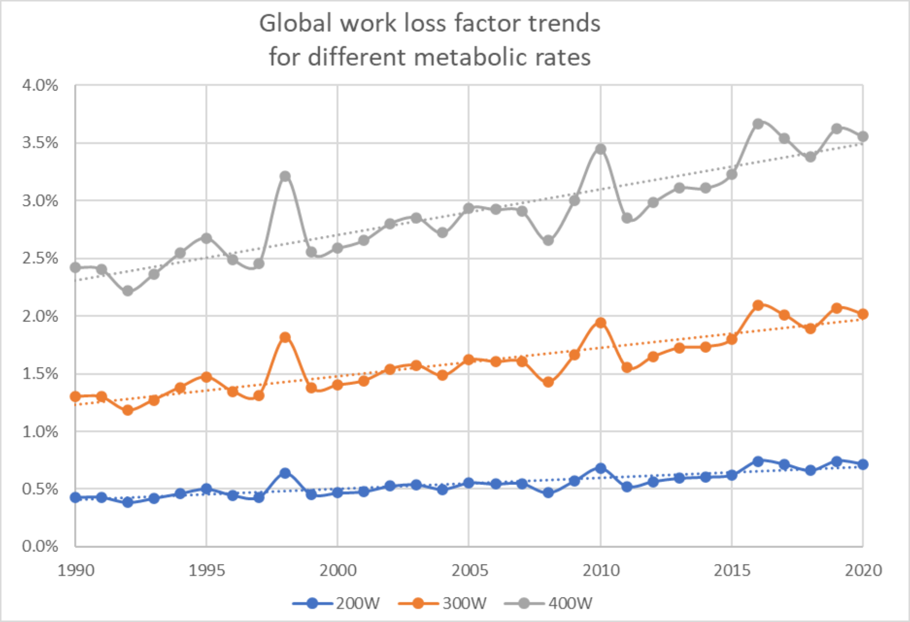

Figure 9. Work hours lost (% of annual hours) depending on physical work intensity, global means, 1990-2020.

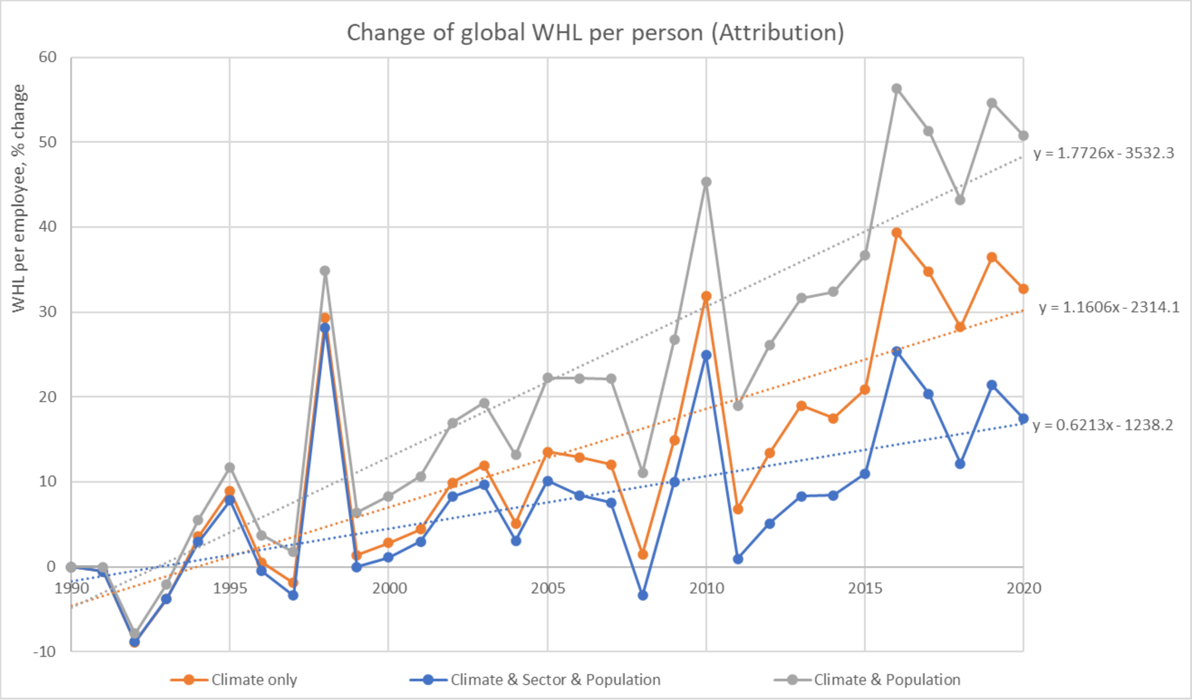

Figure 10. Global changes of work hours lost per employed person, 1990-2020.

Table 4: Annual heat-related work hours lost per employed person (WHLpp, in shade or indoors) and total WHL in populous countries. Twelve countries with largest populations (three in each HDI category) ranked by WHLpp in 2020.

	ISO3 code	Human development level	Latitude	Work hours lost per employed person in 2000	Work hours lost per employed person in 2020	Billions of work hours lost in 2020	% of global
Global				75.8	88.1	295.4	100.0%
Pakistan	PAK	Medium	Sub-trop	232.3	261.2	18.7	6.3%
Bangladesh	BGD	Medium	Trop	229.6	234.8	16.6	5.6%
India	IND	Medium	Trop	192.9	216.2	113.4	38.4%
Nigeria	NIG	Low	Trop	122.0	132.4	9.3	3.2%
Indonesia	IDN	High	Trop	113.1	129.2	16.3	5.5%
Democratic Republic of Congo	COD	Low	Trop	70.1	110.6	3.7	1.2%
China	CHN	High	Sub-trop	47.7	36.3	26.9	9.1%
Brazil	BRA	High	Trop	38.8	33.6	3.2	1.1%
Japan	JAP	Very High	Sub-trop	14.2	18.2	1.1	0.4%
Ethiopia*	ETH	Low	Trop	11.0	14.6	0.7	0.2%
USA	USA	Very High	Sub-trop	8.7	11.6	1.9	0.6%
Russia	RUS	Very High	Temp	1.3	0.9	0.1	0.0%
Rest of the world				17.6	24.9	83.6	28.3%

* The low impact per employee is linked to the high altitude (with cooler climate) of most of this country

Indicator 1.1.5: Heat and Sentiment

Methods

This is the first year this indicator has been included in a Lancet Countdown report.

The indicator tracks the effect of heatwaves, as defined in Indicator 1.1.2, on the sentiment of billions of expressions across millions of global Twitter users.

It builds from Twitter data consisting of 6.14 billion geolocated tweets collected via Amazon Web Services servers from the Twitter Streaming API between 2015 and 2020. These tweets spanned the globe, with a median number of unique active daily users of approximately 900,000 (Figure 11).

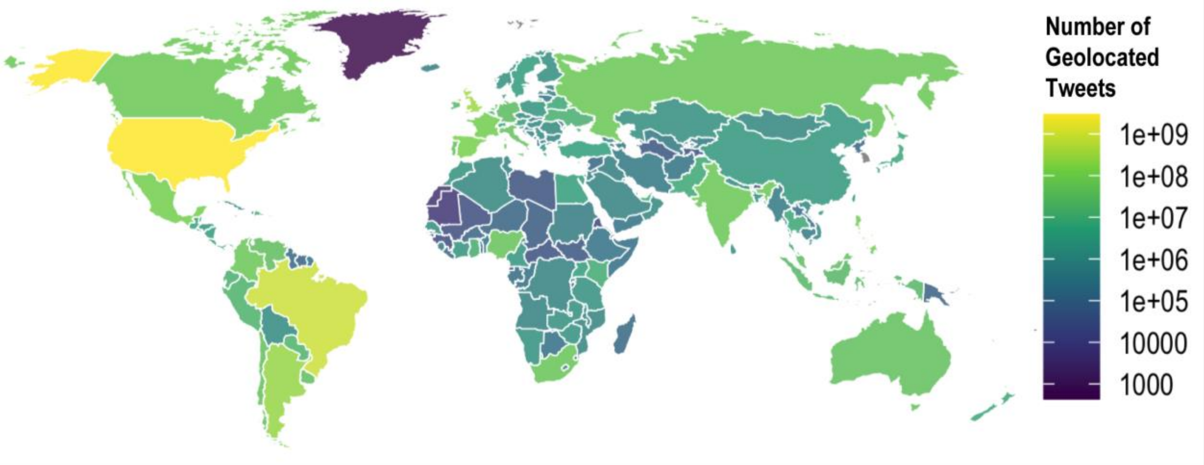

Figure 11. Country-level count of geolocated tweets, 2015-2020.

The positive and negative valence[29] of each collected Twitter posts was classified using the Linguistic Inquiry Word Count (LIWC) sentiment classification tool, a psychometrically validated text-based sentiment measure that classifies certain words as indicating particular valences of sentiment (positive and negative) using a dictionary-based approach. Their multilingual dictionaries are developed via a combination of both translation and expert curation from native-speaking psychologists in those languages.[30,31] Eleven available languages were used: Dutch, English, French, German, Italian, Portuguese, Romanian, Russian, Serbian, Spanish, and Ukrainian, which provided broad geographic coverage for the indicator. Table 5 presents the by-language breakdown in the distribution of collected tweets. Tweets with a 'lang' field matching each respective language were classified using that language's dictionary.

Table 5. By-language breakdown in the distribution of collected tweets.

Language	% of Tweets
English	71.90%
Portuguese	12.46%
Spanish	11.18%
French	1.81%
Russian	1.23%
Italian	0.65%
German	0.36%
Dutch	0.35%
Ukrainian	0.03%
Romanian	0.02%
Serbian	0.01%

ECMWF ERA5 data were retrieved from 2015 to 2020 to calculate the heatwave exposure metric as described for the indicator 1.1.2. Daily precipitation, cloud cover, relative humidity, diurnal temperature range and wind

speed from the same dataset were also incorporated. The primary spatial unit of analysis was the second administrative division-level; the temporal unit of analysis was the calendar date, resulting in second-administrative-unit-by-day analyses.

To aggregate meteorological variables to these units of analysis, ECWMF ERA5 data were extracted to the shapefile boundaries of the second administrative units for each day in the data.

To aggregate sentiment measures, the procedures outlined in Baylis et al. 2018[32] were followed. Namely, for both positive and negative sentiment dimensions, as two separate variables, each tweet was coded as either zero if the tweet contained no matching sentiment terms or one if it contained terms that matched the corresponding sentiment. A tweet could express both positive and negative sentiment, only one of the two, or neither. For each day in the data the average positive sentiment and the average negative sentiment for each unique user on that day, multiplying by 100 to produce a percentage, were calculated. Users' scores were then averaged within the same second-division administrative unit together to produce daily administrative sentiment measures. These measures ranged between 0 and 100.

To estimate the effect of exposure to heatwaves on positive and negative sentiment ordinary least squares models drawn from climate econometrics were employed. The dependent variables were positive and negative sentiment; the primary independent variable was an indicator of whether or not an administrative-unit-day was experiencing a heatwave (with the latter controlled for the afore-mentioned meteorological conditions). To control for potentially confounding factors that may vary over time across different locations calendar-month-by-2nd-administrative region unit fixed effects were included in the models. To account for idiosyncratic day-specific effects, calendar date fixed effects were included as well.[33-36]

The employed ordinary least squares model largely replicated that in Baylis et al. 2018[32] and is as follows:

$$Y_{jmt} = \beta HEAT_{jmt} + h(\mu) + \gamma_t + \nu_{jm} + \epsilon_{jmt}$$

where j indexes 2nd-level administrative region units, m indexes unique calendar months, and t indexes unique calendar dates. Y_{jmt} represents the dependent variables of positive and negative sentiment rates, respectively, $HEAT_{jmt}$ represents the binary heatwave indicator, which equaled one if the date was classified as a heatwave in location m and equaled zero otherwise. β is the effect of a heatwave on positive and negative expressed sentiment rates in percentage points. $h(\mu)$ represents meteorological controls, which include 20 percentage point percentile-bin controls for temperature observations (with the omitted category of the 40th-60th temperature percentile bin serving as the baseline reference category for $HEAT_{jmt}$). $h(\mu)$ also includes flexibly binned control variables for precipitation amounts, cloud cover percentages, relative humidity, and wind speed. Further, γ_t represents date-specific fixed effects that controlled for any idiosyncratic shocks in the data as well as factors that trended similarly over time across all locations. ν_{jm} indicates second-administrative-unit-by-calendar-month fixed effects that controlled for any location-specific seasonal and secular trends that might confound inference. ϵ_{jmt} represents an error term. Errors were clustered on administrative-unit-by-month and date. Finally, the regression was weighted by the number of unique Twitter posts in each administrative-unit-day and the model estimated for each year within the collected Twitter, giving a β for each year.

Data

1. Climate data from the European Centre for Medium-Range Weather Forecasts (ECMWF) ERA5 reanalysis.[17]
2. Geolocated tweets collected via the Twitter Streaming API.

Caveats

While this indicator has many strengths, particularly as compared to existing survey-based and surveillance-based methods, it is neither a perfect nor exhaustive measure of sentiment during heatwaves. Importantly, while sentiment is related to mental wellbeing, it should not be confused as a measure of it and should be interpreted as an indicative proxy of the mental implications of extremes of heat.

Countries that did not have Twitter broadly available to the public – such as China – were underrepresented. Furthermore, geo-tagged tweets constituted approximately two percent of all tweets and thus may be somewhat limited in their generalizability due to opt-in geo-localization. Finally, the vast majority of the Twitter observations were posted in wealthy countries. Since higher income populations likely have greater access to adaptive amenities (air conditioning, etc.), the estimates produced by our identification strategy may have been conservative (downward biased) for those disproportionately exposed to some of the hottest conditions in poorer socioeconomic contexts.

Future form of the indicator

Since climate change affects other dimensions of mental wellbeing not registered by the current indicator, the indicator is well-positioned to house further psychosocial indicators that can complement the broad-scope measures of mental wellbeing tracked here.

Additional analysis

Negative sentiment increased nearly three times as much during heatwaves in low HDI countries compared to very high HDI countries during non-heatwave days (Figure 12). By contrast, positive sentiment only substantively and significantly declined for those exposed to heatwaves in high HDI contexts. The annual estimates presented in this indicator provide evidence that local exposure to heatwaves reduced the positive expressions and increased the negative expressions of those exposed. The magnitude of the reductions in positive sentiment expression, taken in context of other effects observed within the data, were substantively meaningful and mirrored the effects observed via other social media data in other contexts.[32,37] For instance, the average reduction in public positive sentiment from a single heatwave day during the period of 2015-2020 was approximately a third of the average magnitude of decline observed during the 2015 Carolinas Flooding event in the USA (Figure 13). Similarly, the additional percentage point increase (+0.12) in negative sentiment observed in 2020 (0.20) above the 2015-2019 baseline level (0.08) was 75% of the total effect size of the rise in negative sentiment observed during the 2015 Carolinas Flooding event (0.16).

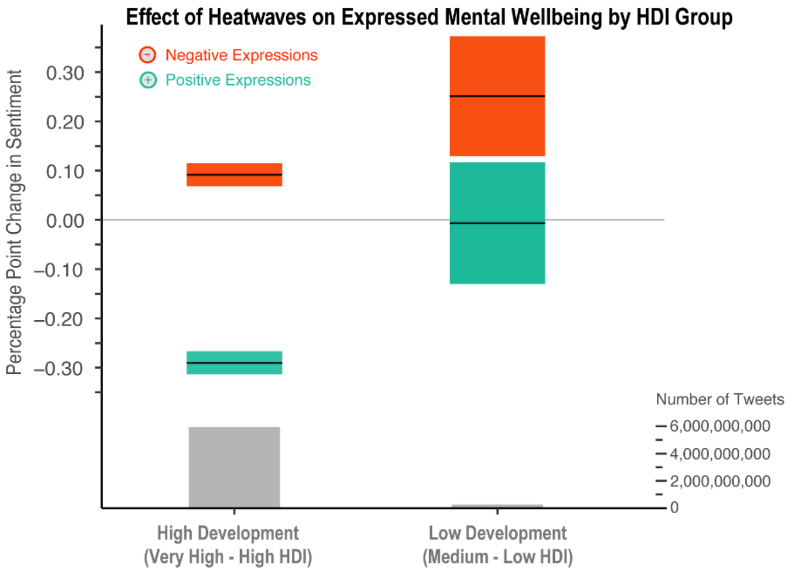

Figure 12. Annual effect of heatwave exposure on the sentiment of online expressions from 2015-2020. Coloured intervals depict 95% CIs of the estimated average change in positive (green) and negative (orange) sentiment expressions during days with heatwaves, relative to the median daily maximum temperature baseline range for each location and year. Grey bars depict the geolocated Tweet count by year of observation, 2015-2020.

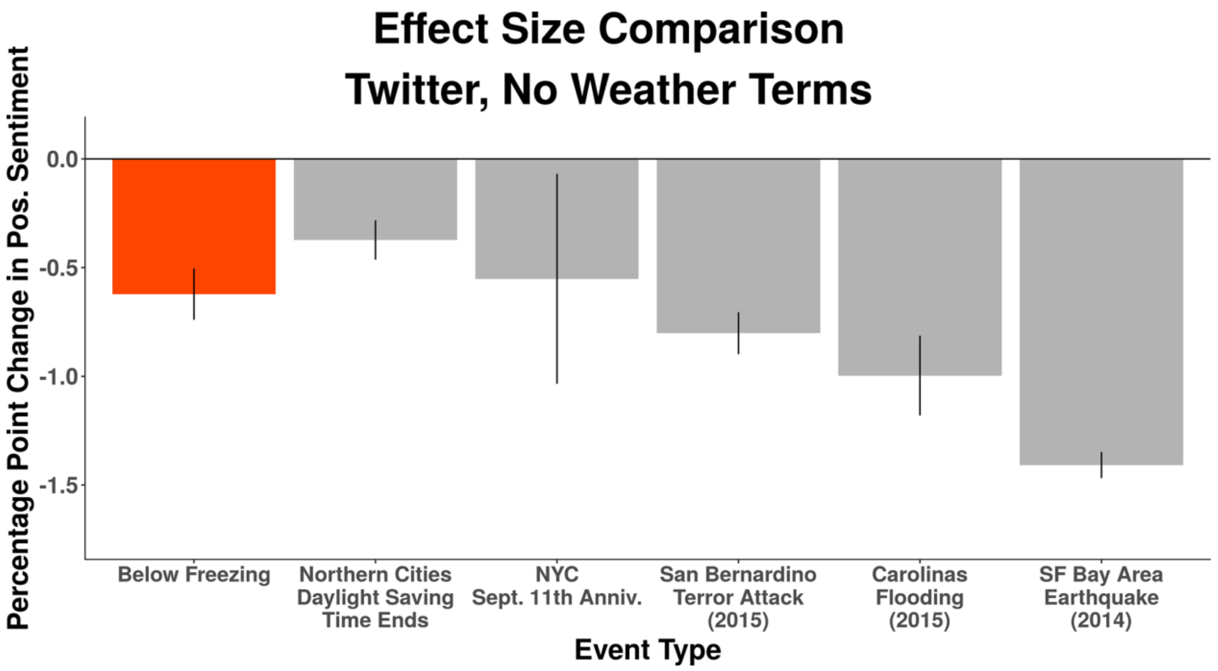

Figure 13. Effect size comparison for positive sentiment expressed on Twitter during various benchmarking events.

Indicator 1.1.6: Heat-Related Mortality

Methods

The indicator tracks the global total number and spatial pattern of heat-related mortality from 2000 to 2019. The method is as follows.

The heat-related excess mortality in one day E is expressed as

$$E = y_0 \times Pop \times AF \qquad (1)$$

where y_0 is the non-injury mortality rate on that day, Pop is the population size and AF is the attributable fraction on that day. Because every day's mortality rate is hard to obtain, y_0 is computed as the yearly non-injury mortality rate from the Global Burden of Disease data, divided by 365.

AF is calculated via the relative risk (RR) which represents the increase in the risk of mortality resulting from the temperature increase. RR is regressed as $RR = exp^{\beta(t-OT)}$, so AF is calculated as

$$AF = \frac{RR-1}{RR} = 1 - exp^{-\beta(t-OT)} \qquad (2)$$

where t is the daily maximum temperature, β is the exposure-response factor and OT is optimum temperature, and both parameters were adopted from Honda et al. (2014).[38] The method was applied to gridded daily temperature data from ECMWF ERA5 dataset, and gridded population data from NASA GPWv4 population dataset and ISIMIP Histsoc records, as with Indicator 1.1.2. As the indicator focuses on population that is 65 years old or older, age-structure data from United Nation World Population Prospects was also used.

According to the WHO website, years of life lost (YLL) is calculated as:

$$YLL = \sum_{m=65-69}^{100+} E_m \times LE_m \qquad (3)$$

where YLL is the annual YLL of a certain grid cell, E_m is annual heat-related excess mortality in age group m of the grid, and LE_m represents the standard life expectancy at the age of death in years of age group m. The life expectancy data was obtained from the Global Burden of Disease Study 2019 (GBD 2019) Results by Institute for Health Metrics and Evaluation (IHME), same with the mortality rate data.[39]

The heat-related mortality and YLL was first calculated at grid level at 0.5° spatial resolution. Then it was accumulated to global level to produce a time-series analysis.

Data

1. Climate data from the European Centre for Medium-Range Weather Forecasts (ECMWF) ERA5 reanalysis.[17]
2. Population data from the NASA Socioeconomic Data and Applications Center (SEDAC) Gridded Population of the World (GPWv4) and The Inter-Sectoral Impact Model Intercomparison Project (ISIMIP) Histsoc dataset.[5,6]
3. Demographic data from the United Nation World Population Prospects (UN WPP).[40]
4. Mortality rate and life expectancy data are from the Global Burden of Disease.[39]

Caveats

This indicator applies a unique exposure-response function across all locations and times. While its use has been demonstrated in different geographies, it does not capture local differences in the health impacts from heat exposure, which can be significant. Also, this analysis assumes exposure-response function is constant. It does

not capture changes in response to heat exposure that might happen over time, as a result of acclimation and adaptation. Not capturing these changes could result in an over-estimation of heat-related deaths in later calendar years. Annual average mortality rates are used, rather than daily mortality rates (y_0). Given baseline mortality can be higher in colder months, this may lead to an overestimation of overall mortalities. Nonetheless, the trends of change in mortality due to heat exposure should still be conserved.

Only the heat-related mortality of the 65-and-older population was calculated this time, but more work needs to be done to include working group people.

Future form of the indicator

Work is underway to develop localised exposure-response functions, partly building on the substantial contributions that the Multi-Country Multi-City (MCC) Collaborative Research Network has done to this field of work.[41] Another improvement could be to calculate the mortality for all ages, not only for people over 65 years old.

Additional analysis

The change in global heat-related mortality and years of life lost is presented in Figure 14. In 2019, heat-related mortality increased in all WHO regions except the European region, and the increase in South-East Asian is the most (Table 6). In terms of HDI categories heat-related mortality had increased very quickly from 2018 to 2019 in high, medium and low HDI regions (Table 7).

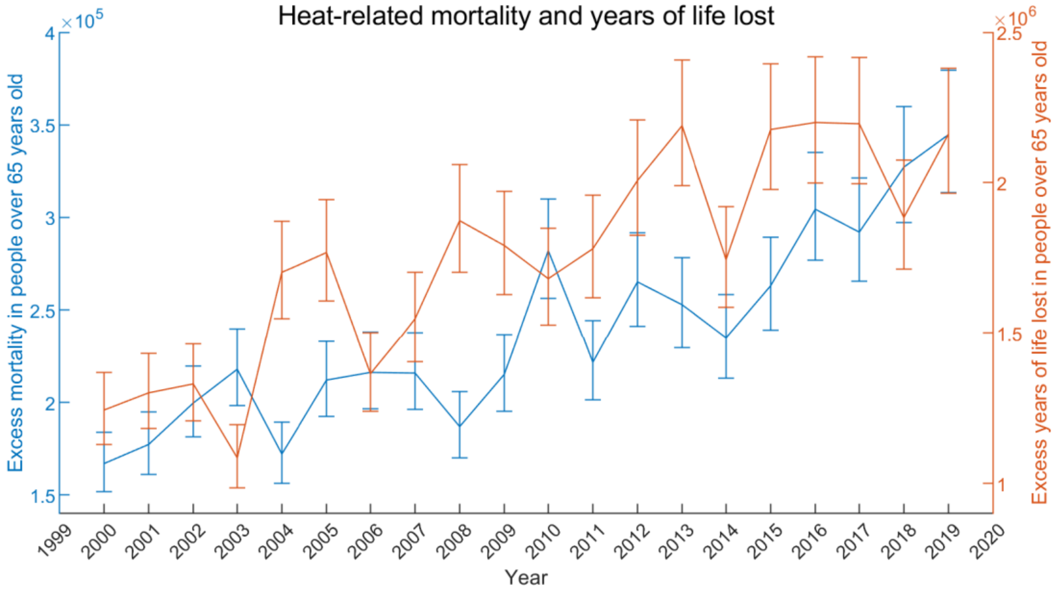

Figure 14. Global heat-related mortality and years of life lost in the 65-and-older population, 2000-2019. The error bars were calculated on the basis of the 95% CIs of the exposure-response function described by Honda et al (2014).[38]

Table 6. Change of heat-related mortality the 65-and-older population between 2019 and 2018 for different WHO regions.

WHO Region	2018	2019	Change in mortality
African	11,067	14,561	3,493
Americas	33,556	38,884	5,328
Eastern Mediterranean	13,365	13,726	360
European	109,654	94,610	-15,044
South-East Asian	35,792	55,817	20,025
Western Pacific	84,242	85,449	1,207

Table 7. Change of heat-related mortality the 65-and-older population between 2019 and 2018 for different level of HDI.

HDI Category	2018	2019	Change in mortality
Very High	138,297	119,805	-18,491
High	99,175	110,663	11,488
Medium	40,192	60,252	20,060
Low	8,635	10,998	2,363

Because higher HDI countries tend to have a higher proportion of their population aged over 65, these countries tend to be particularly vulnerable to heat-related mortality. However, many lower HDI countries are experiencing both growing and aging populations and increasing extreme temperatures. It is perhaps not surprising therefore that in many of these countries, the change in heat-related mortality for people over 65 is relatively high (see Table 7).

1.2: Health and Extreme Weather Events

Indicator 1.2.1: Wildfires

Methods

The methodology for this indicator remains similar to that described in the 2020 report of the Lancet Countdown.[1]

The change in population exposure to wildfire is represented as the change in the average annual number of days people were exposed to wildfire in each country. Satellite-observed active fire spots were aggregated and spatially joined with gridded global population data on a global 10km x 10km resolution grid. Grid cells with a population density ≥ 400 persons/km^2 were excluded to remove urban heat sources unrelated to wildfires. The mean annual number of person-days exposed to wildfire during the most recent four years was compared with the baseline period of 2001-2004.

The fire danger is represented in terms of the Fire Danger Index (FDI). Provided by ECMWF ERA5 atmospheric reanalysis, FDI is a numeric rating with values 1-6 representing very low, low, medium, high, very high and extreme fire danger, respectively. Daily FDI data, available from 3rd January 1979 through 26th December 2019 worldwide, was aggregated so as to obtain the yearly number of days of each fire danger level at every 0.25° x 0.25° grid cell. The changes in mean number of days exposed to very high or extremely high fire danger (defined as FDI ≥ 5) were collected for the most recent available period, 2017 to 2020, and compared with a baseline from 2001 to 2004.

Gridded population density data (i.e., population count per square kilometre) from NASA SEDAC GPW v4.11 dataset, was retrieved for the years 2000, 2005, 2010, 2015, and 2020. The data set with a spatial resolution of 2.5' × 2.5' (around 5km x 5km) was used. Population density data was re-gridded to the spatial resolution of the fire danger data using a conservative method (i.e., the total population is conserved) and further linearly interpolated for each year from 2000-2020. The re-gridded population data was used to calculate population-weighted mean days of fire risk. Similar to wildfire exposure, grid cells with a population density ≥ 400 persons/km^2 were excluded in the calculation of changes in mean number of days exposed to very high or extremely high fire danger.

Data

1. Collection 6 active fire product from the Moderate Resolution Imaging Spectroradiometer (MODIS); this contains both Terra (from November 2000) and Aqua (from July 2002) pixels in the same annual file.[42]
2. Fire danger indices historical data produced by the Copernicus Emergency Management Service for the European Forest Fire Information System (EFFIS).[43]
3. Population data from the NASA Socioeconomic Data and Applications Center (SEDAC) Gridded Population of the World (GPWv4).[5]

Caveats

Cloud cover may introduce spatial biases into fire exposure estimates. While observing the same fire, Terra and Aqua may report slightly different coordinates of the fire centroid, therefore introducing a double counting issue. This indicator does not quantify the populations exposed to wildfire smoke, which increases morbidity and mortality of cardiovascular and respiratory diseases.[44] Estimating the distribution and population exposure to wildfire remains challenging.

The fire danger index represents a potential fire risk calculated by meteorological parameters. It does not represent actual fire events. The actual fire events can be also influenced by anthropogenic factors, such as human-induced land use and land cover changes, industrial-scale fire suppression, and human induced ignition.

The fire danger index does not account for the potential fertiliser effect of CO_2 and the associated changes in vegetation and thus the fuel load of fire.

The fire danger index does not consider potential changes in lightning ignitions, which can be affected by climate change, but the effect is highly uncertain.

Future form of the indicator

This indicator will be improved to reduce the impact of cloud cover on active fire data.

Indicator 1.2.2: Drought

Methods

The data source for this indicator has been updated for the 2021 report.

The drought indicator was improved in the 2021 report, using the Standard Precipitation Evapotranspiration Index (SPEI)[45], to measure meteorological drought. The index can measure drought severity according to intensity and duration and can identify the onset and end of drought episodes. It also allows comparison of drought severity through time and space, since it can be calculated over a wide range of climates. SPEI extends the Standard Precipitation Index (SPI), used in previous reports, by considering the effect of potential evapotranspiration on drought severity.

SPEI data are obtained from the SPEI Global Drought monitor. The Global Drought monitor uses mean temperature data from the NOAA NCEP CPC GHCN_CAMS gridded dataset[46] and monthly precipitation data from the 'first guess' Global Precipitation Climatology Centre (GPCC)[28]. GCPC data, which have an original spatial resolution of 0.5º x 0.5°, are interpolated to the resolution of 1° x 1°. Potential evapotranspiration is calculated using the Thornthwaite equation.

The Global Drought monitor calculates SPEI values using constantly updated climate data at a global scale with a 1° x 1° spatial resolution and a monthly time resolution. SPEI time-scales between 1 and 48 months are provided. For the indicator the 1-monthly SPEI value is used and the calibration period is set to January 1950 to December 2010. The 2020 report presented similar work but using and alternative SPEI dataset provided by the Global Drought monitor based on CRU 4.03 dataset, which is only available until 2018 and is not as regularly updated.[1] The new up-to-date SPEI data used in the 2021 report are regularly updated (up to recent months) and, as such, are useful for tracking drought trends. SPEI 1-month index data for 1950-present were downloaded from the Global Drought Monitor site.

Droughts were defined according to three severity levels using the SPEI thresholds indicated in Table 8, as defined by the Federal Office of Meteorology and Climatology MeteoSwiss.[47] In order to detect excess (unusual) drought events, "excess severe drought events" were defined as yearly counts of months in drought for each grid cell which exceed 2 standard deviations above the mean of the yearly counts of months in drought for the baseline period of 1986-2005. The excess events were defined for each SPEI severity level of drought independently, and the percentage of land area exposed to excess drought events at the different severity levels were calculated.

Table 8 Summary of drought severity thresholds.

**SPEI value**	**description**	**frequency of event in respective month**
< -1.3	severe drought	1-2 x in 20 years (i.e. 10% if the time)
< -1.6	extreme drought	1-2 x in 40 years (i.e. 5% of the time)
< - 2	Exceptional drought	1 x in 50 years or less (i.e. ≤2% of the time)

For total drought events, the more areas affected severe droughts are strict subsets of the areas affected by milder droughts in that year. However, for excess droughts, the excess area defined *with respect to that drought's severity level*. As the baseline distributions of drought events are independent for each severity level, the resulting trends are also independent, and the areas affected by excess severe droughts are no longer strict subsets of areas affected by excess mild droughts.

Data

1. The SPEI index was taken from the global SPEI database, SPEIbase (Consejo Superior de Investigaciones Cientificas).[48]

Caveats

A limitation of this indicator is that it only captures the impacts of climate change on meteorological drought but does not capture the impacts of climate change on hydrological or agricultural drought, which can have major health impacts too. Moreover, it does not measure the direct relationship between a drought and the population living in drought-affected areas. It is not possible to do a population-based weighting because many people affected by a drought may not live in the area affected, e.g., in the case of droughts affecting agricultural areas (which are generally sparsely populated) with impacts on the food supply. It is therefore difficult to determine trends in persons affected by drought from the trends of severe drought area.

Further work is required to link reported drought damages in societies to climatic indicators. This would require a better understanding of the exposure factors of populations.

Future form of the indicator

Further development of the indicator will focus on using a combination of indices that capture agricultural hydrological drought, and meteorological drought, and better capture the health implication of drought events.

Additional analysis

A more negative value of SPEI corresponds to dryer conditions. Figure 15 highlights that the SPEI value from the Global Drought Monitor becomes significantly more negative on average over the 1950-2020 period. There is also some divergence between the regularly updated values based on NOAA/GPCC data and the 1950-2018 values based on the CRU 4.03 dataset, even though the same calculation method is applied in both cases. This highlights the challenges in accurately tracking precipitation and drought patterns globally.

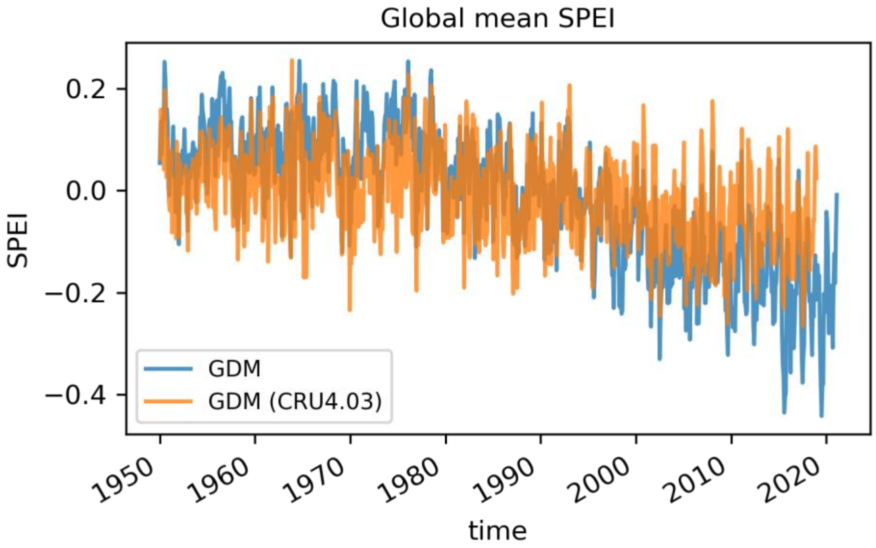

Figure 15. Global mean SPEI over the 1950-2020 period from the Global Drought Monitor (GDM) comparing the live-updated datasets and the CRU4.03-based historical dataset. More negative values correspond to dryer conditions.

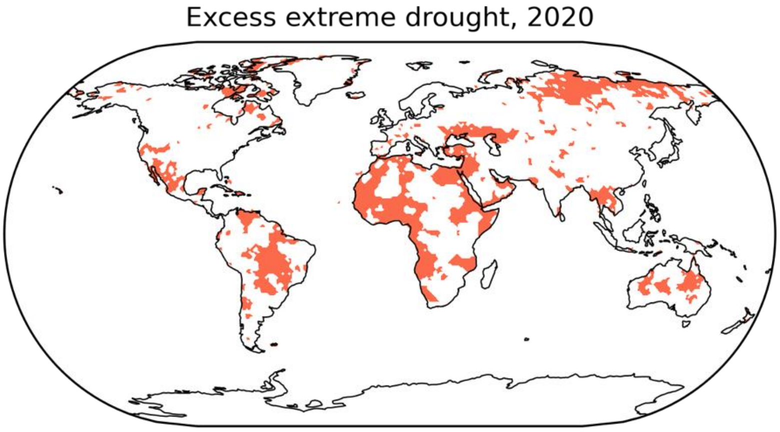

Figure 16. Land surface area in excess extreme drought in 2020.

Indicator 1.2.3: Lethality of Extreme Weather Events

Methods

The methodology for this indicator remains similar to that described in the 2020 report of the Lancet Countdown.[1] The number of occurrences of weather-related disasters (drought, storms, wildfires, floods and extreme temperatures), the number of people affected in each disaster, and the lethality of these events have however been grouped according to the 2019 HDI level for each country over the period from 1990 to 2020.

The methodology uses data from the Centre for Research on the Epidemiology of Disasters (EM-DAT).[49] Here, deaths, as proxy of the lethality of weather-related disasters, are defined as the number of people who lost their life because the disaster happened. People affected are defined as those requiring immediate assistance during a period of emergency; hence requiring basic survival needs such as food, water, shelter, sanitation and immediate medical assistance.

Data

1. EM-DAT at the Centre for Research on the Epidemiology of Disasters (CRED) at the Université Catholique de Louvain, Belgium.[49]
2. Human Development Index (HDI) at the United Nations Development Programme, Human Development Reports.[50]

Caveats

The EM-DAT database contains a number of possible biases. Firstly, there is a possible bias in missing some disaster events because of under-reporting. EM-DAT classifies an event as a disaster if 10 or more people die; 100 or more people are affected; there is a declaration of a state of emergency; or a call for international assistance. Similarly, there are likely biases in how countries report both the number of deaths and people affected. Numbers of deaths for example may not include mortality from the cascading risks of natural hazards or those that occur as a result of longer causal chains from the hazard. Secondly, estimates of the numbers of people affected have different biases for different countries because of how the concept of "affected people" is defined. This must be considered when comparing countries.

Additional analysis

The number of weather-related disasters and its progression for each HDI category is shown in the figures below. For trends, data are presented as standardised anomalies (Z scores), representing the difference between the variable that year and average of the variable from 1990-2020, normalised by the standard deviation of the variable over the same period. Only statistically significant (at 0.05 significance level) linear trends over time are shown. Additionally, Table 9 reveals that floods and storms make up the highest number of weather-related disasters in EM-DAT.

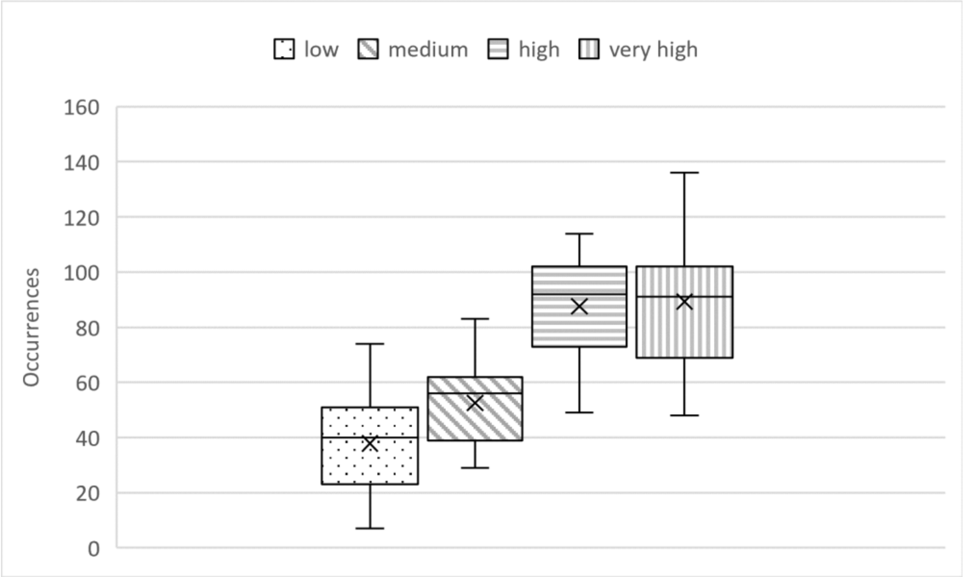

Figure 17. Box plot of annual number of occurrences of weather-related disasters (drought, temperature extremes, floods, storms and wildfires) from 1990-2020.

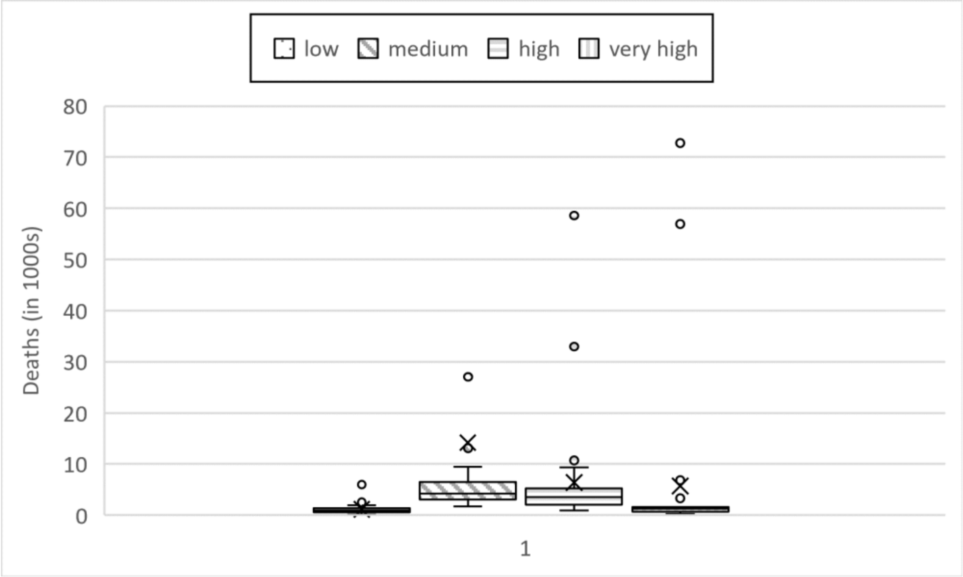

Figure 18. Box plot of annual number of deaths caused by weather-related disasters. Note: one outlier of 141801 under the medium HDI box plot, is not shown.

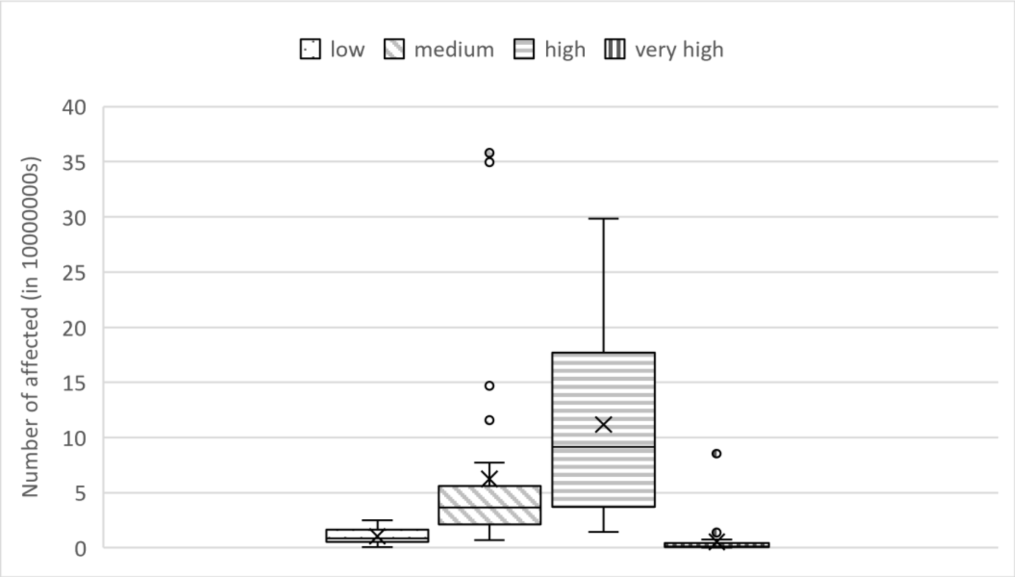

Figure 19. Box plot of annual number of people affected by weather-related disasters.

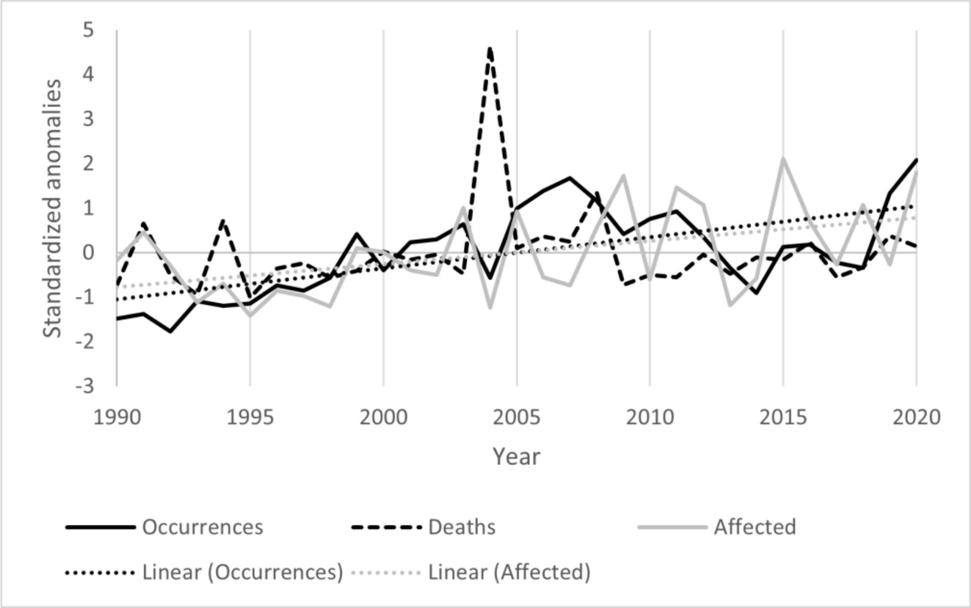

Figure 20. Time series of standardized anomalies of the number of occurrences of weather-related disasters, deaths caused by these disasters and numbers of people affected by them, for low HDI countries. Significant trends, at the 0.05 level, are shown for occurrences (y = 0.0699x - 140.22, R^2 = 0.40) and the number of people affected (y = 0.0518x - 103.94, R^2 = 0.22). The standardizing period for anomalies is from 1990-2020.

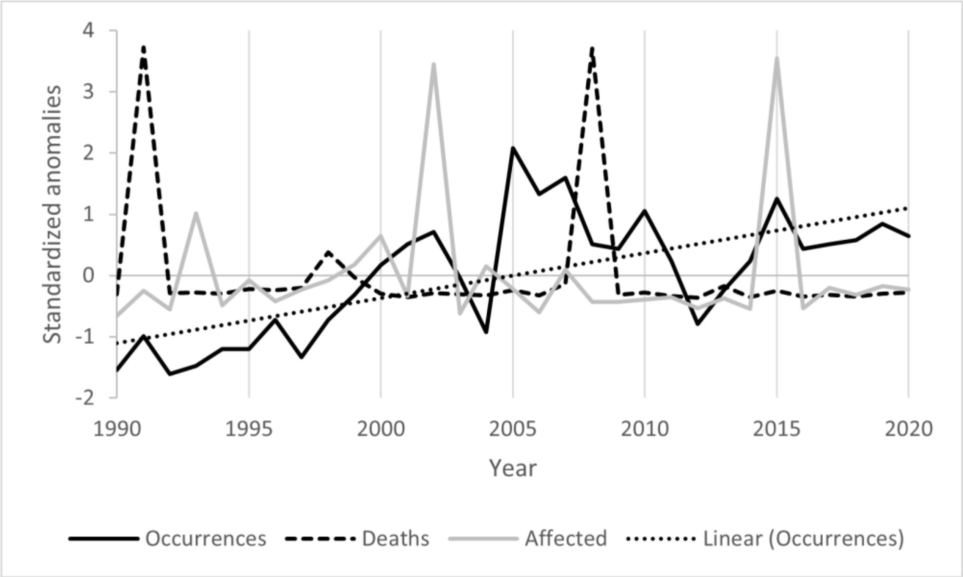

Figure 21. Time series of standardized anomalies of the number of occurrences of weather-related disasters, deaths in these disasters and numbers of people affected, for medium HDI countries. A significant trend, at the 0.05 level, is shown for occurrences (y = 0.0735x - 147.44 R^2 = 0.45). The standardizing period for anomalies is from 1990-2020.

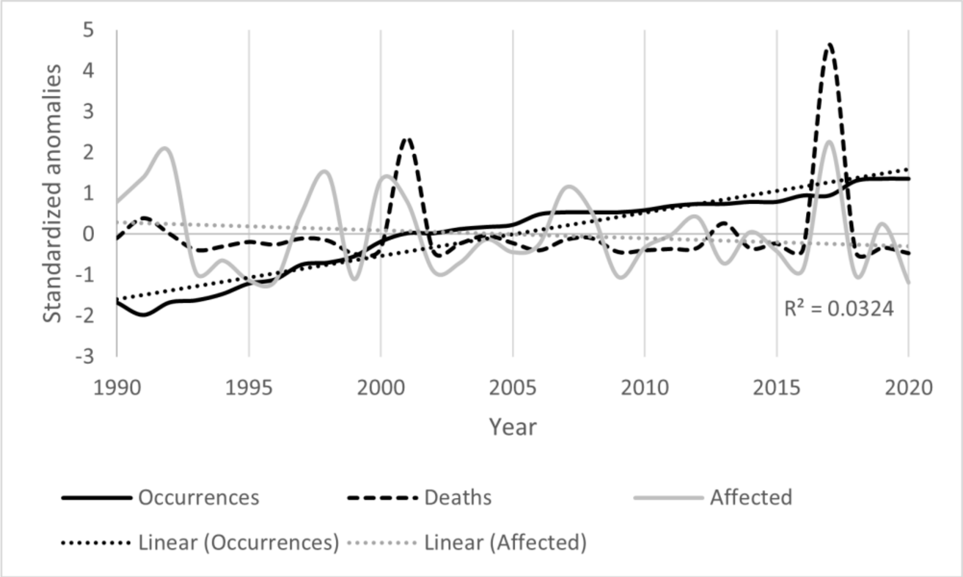

Figure 22. Time series of standardized anomalies of the number of occurrences of weather-related disasters, deaths in these disasters and numbers of people affected, for high HDI countries. A significant trend, at the 0.05 level, is shown for occurrences (y = 0.1063x - 213.2 R^2 = 0.94). The standardizing period for anomalies is from 1990-2020.

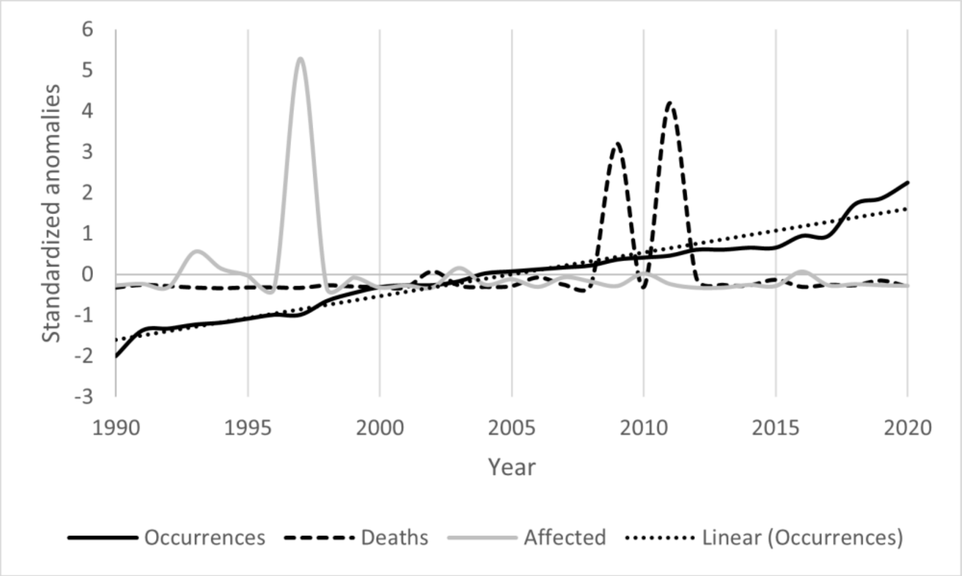

Figure 23. Time series of standardized anomalies of the number of occurrences of weather and climate related disasters, deaths in these disasters and numbers of people affected, for very high HDI countries. A significant trend, at the 0.05 level, is shown for occurrences ($y = 0.107x - 214.63$ $R^2 = 0.95$). The standardizing period for anomalies is from 1990-2020.

Table 9. Percentage of type of event (droughts, extreme temperature, floods, storms and wildfires) by HDI level in the EM-DAT database from 1990-2020.

	Droughts	Extreme Temperatures	Floods	Storms	Wildfires
Low HDI	12	1	68	17	2
Medium HDI	7	6	57	29	1
High HDI	5	4	54	35	2
Very high HDI	2	11	34	44	9

1.3: Climate-Sensitive Infectious Diseases

Indicator 1.3.1: Climate Suitability for Infectious Disease Transmission

Dengue, Chikungunya and Zika

Methods

R_0, ie the basic reproduction number, which is the expected number of secondary infections resulting from one single primary infected person case in a totally susceptible population, was computed using the formula below:[51]

$R_0 = Vb_h/r_h$

Where b_h is the human infection probability when bitten, and $1/r_h$ is the infectious period length.

The vectoral capacity (V), which express the average daily reproductive rate of subsequent cases in a susceptible population resulting from one infected case, was computed using the formula below from[51]:

$V = ma^2 b_m p^n / -\ln p$

Here, a is the average vector biting rate, b_m *is* probability of vector infection and transmission of virus to its saliva, n is the extrinsic incubation period, and p is the daily survival probability. All these parameters are temperature dependent and are further described in Rocklöv et al [51-53].

The ratio between number of mosquitoes to the number of humans, is central to V and the R_0 value (m). Here, a model to estimate mosquito populations of *Aedes aegypti* and *Aedes albopictus* separately was used. The original mosquito-population models provide results in terms of the number of individuals of *A. aegypti* per breeding site (X), or the number of *A. albopictus* per hectare (Y).[54,55] In order to appropriately estimate m, (that is, the mosquito population density per human population density (p)), X was multiplied by $f(p,a,c) = a * g(p,c)$ where a equals to the number of breeding-sites per human, and Y by $f(p,a/b,c) = a * g(p,c)/b$ where b equals the average number of breeding sites per hectare. The function $g(p,c) = p^2/(c^2 + p^2)$ is an increasing sigmoidal function that equals the viability of domesticated mosquito-populations in relation to human population density. Accordingly, $f(p,a,c)$ is the multiplicative factor m in V, which allowed us to straightforwardly estimate correct values for a, a/b and c by fitting R_0 to R_0-data that was available for a subset of the spatiotemporal points.[56]

Numerically V and abundance estimates were computed at 0.5°x0.5° spatial resolution based on CRU TS vs 4.0.5[57]. V and vector abundance were run for both *Aedes* aegypti and *Aedes* albopictus vectors. The gridded population data based on HYDE3.2 [58] was used in the computation of R_0. For Dengue and Zika *Aedes aegypti* vector abundance estimates were used in the computation of m, and also separately estimated it for Dengue and Chikungunya using *Aedes albopictus* abundance estimates.

The annual average R_0 were extracted values per grid cell for Dengue (*Aedes aegypti*), Dengue (*Aedes albopictus*), Chikungunya (*Aedes albopictus*) and Zika (*Aedes aegypti*) *based* and averaged these values by country, by HDI and by WHO regions. For the country-specific trends in R_0, monthly and yearly time steps from 1950-2020 were computed. Global R_0 indicates globally averaged values across all countries.

Data

1. Climate research unit (CRU) TS vs 4.0.5 precipitation and temperature data[57]
2. HYDE 3.2 gridded population data [58]

Caveats

Key caveats and limitations of the V model and its parameterisation are fully described in Liu-Helmersson et al.[59,60] and Rocklöv et al.[61] The predicted R0 should not be confused with actual dengue cases, although it is an indicator of the potential for outbreaks.[52,53]

Malaria

Methods

The methodology for this indicator remains similar to that described in the 2020 report of the Lancet Countdown.[1]

The malaria indicator focuses on determining global changes in the length of the malaria transmission season over time between highland and lowland areas according to different categories of the UNDP Human Development Index.

The length of the transmission season, measured as the number of months suitable for malaria transmission per year from 1950–2019, was calculated for each 0.5 deg grid cell over land. Suitability is based on empirically derived thresholds of precipitation, temperature, and relative humidity for *Plasmodium falciparum*, the parasite causing malaria via the *Anopheles* mosquito vectors.

Monthly observations of temperature, precipitation and vapour pressure data from the Climate Research Unit (CRU TS4.04[57]) were downloaded using the Koninklijk Nederlands Meteorologisch Instituut (KNMI) Climate Explorer. The variables were extracted at a 0.5° x 0.5° spatial resolution over land. Elevation data at a 0.5° x 0.5° spatial resolution was obtained from the University of Washington Joint Institute for the Study of the Atmosphere and Ocean (JISAO).

Relative humidity (RH) was estimated using the formula:[62]

$$RH = \frac{e}{e_{sat}} \times 100,$$

where e is vapour pressure and e_{sat} is saturated vapour pressure (in hPa) at mean air temperature T in °C, given by:

$$e_{sat} = 6.108 \exp\left[17.27\, T / (237.3 + T)\right] .$$

The length of the transmission season was defined as the number of months with the coincidence of precipitation accumulation greater than 80 mm, average temperature between 18°C and 32°C, and relative humidity greater than 60%.[63] The combined values are an indication of the lower limit for potential transmission of *Plasmodium falciparum*.

The mean length of the transmission season (i.e., number of months per year with suitable climate conditions) was then calculated at the global level (Figure 24).

The spatial analysis of length of the transmission season of *Plasmodium falciparum* per sub-national HDI was carried out by overlaying the gridded data of the length of transmission season on the sub-national level HDI data for 2018. Ug Moran's I test, the null hypothesis of no spatial autocorrelation between the length of the malaria transmission season and HDI was rejected, suggesting that there is a positive spatial correlation between these two variables.

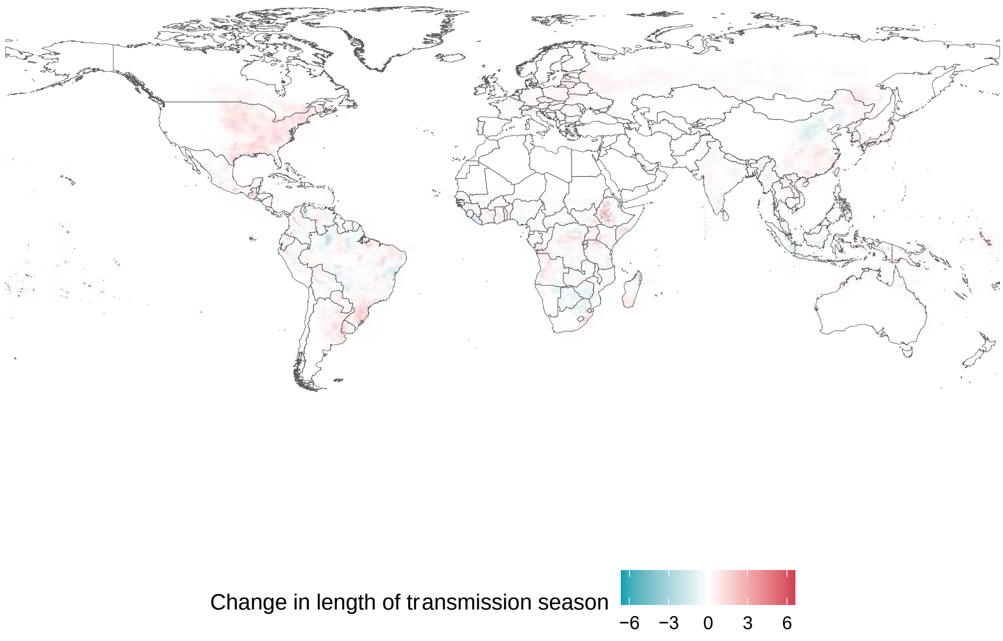

Figure 24. Change in length of transmission season from 1950-1959 to 2010-2019. Change in length of the malaria transmission season measured as number of months per year with precipitation accumulation greater than 80 mm, average temperature between 18°C and 32°C and relative humidity greater than 60%.

Data

1. Climate Research Unit monthly observations of temperature, precipitation and vapour pressure data (CRU TS4.04) from the Koninklijk Nederlands Meteorologisch Instituut (KNMI) Climate Explorer.[57,64]
2. Elevation data from the University of Washington Joint Institute for the Study of the Atmosphere and Ocean (JISAO).[65]

Caveats

These results are based on climatic data, not malaria case data. The malaria suitability climate thresholds used are based on a consensus of the literature. In practice, the optimal and limiting conditions for transmission are dependent on the particular species of the parasite and vector.[66] Control efforts might limit the impact of these climate changes on malaria or conversely, the climate suitability may either enhance or hamper control efforts.[67]

Additional analysis

The percentage change figures reported in the main text were calculated relative to a 1950s baseline (10-year average, 1950-1959 compared to 10-year average, 2010-2019) to illustrate the overall trend, accounting for interannual variability (Figure 25).

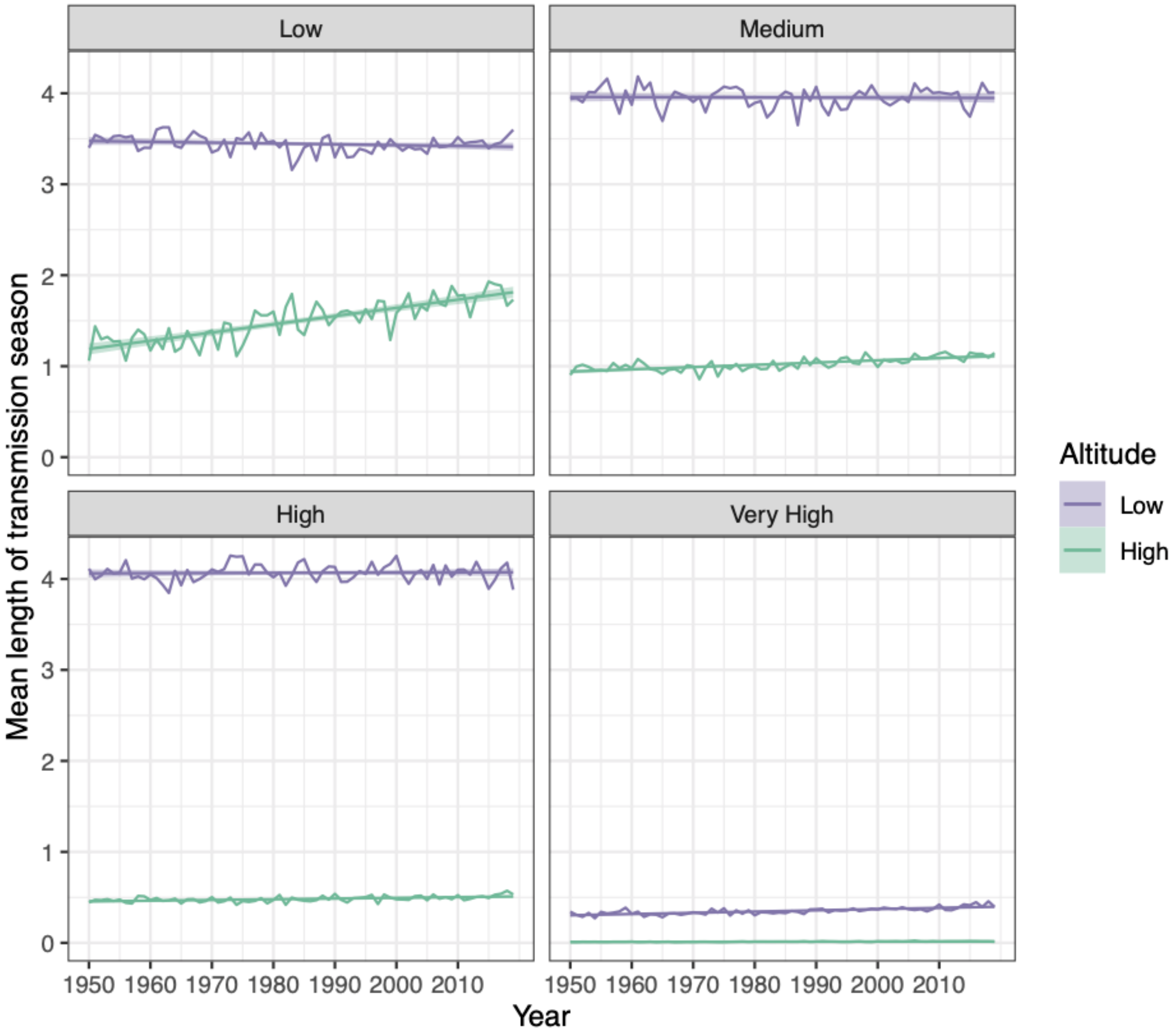

Figure 25. Mean length of the transmission season for malaria 1950 to 2019, grouped by HDI categories and elevation (high ≥1500m, low <1500m). The length of the transmission season is calculated as the number of months per year with precipitation accumulation greater than 80 mm, average temperature between 18°C and 32°C and relative humidity greater than 60%.

Vibrio

Methods

The methodology for this indicator remains similar to that described in the 2020 report of the Lancet Countdown.[1]

This indicator focuses on mapping environmental suitability for pathogenic *Vibrio* spp. in coastal zones globally (<30km from coast). *Vibrio* spp. are globally distributed aquatic bacteria that are ubiquitous in warm estuarine and coastal waters with low to moderate salinity. *V. parahaemolyticus*, *V. vulnificus*, and non-toxigenic *V. cholerae* (non-O1/non-O139) are pathogenic in humans. These *Vibrio* species are associated with sporadic cases of gastroenteritis, wound infections, ear infections, or septicaemia in circumscribed localities.

Vibrio ecology, abundances, distributions, and patterns of infection are often strongly mediated by environmental conditions. The indicator uses thresholds of >18°C for Sea Surface Temperature (SST) and <30 PSU for Sea Surface Salinity (SSS). These values were derived on the basis of a consensus in the literature on what environments *Vibrio* infections may thrive.[68-70] Estimates for SST were obtained from NOAA Optimum Interpolation 1/4 Degree Daily Sea Surface Temperature (OISST) Analysis version 2 for the period 1982-2020. Estimates of SSS were created from daily data obtained from Mercator Ocean Reanalysis.[71]

Here suitability is reported at two levels. First, it was calculated the percentage of coastline globally that experienced suitable conditions for *Vibrio* infections and summarised the results across three latitudinal bands (northern latitudes = 40-70°N; tropical latitudes = 25°S-40°N; and southern latitudes = 25-40°S). Second, suitability in three focal regions in which human *Vibrio* infection is frequently observed, the Baltic Sea, the Pacific northwest and the northeastern coast of the USA (36-50°N) were calculated. For the Baltic (main text), Pacific NW and northeastern coast of the United States, the percentage of coastline suitable for *Vibrio* infections are presented. In addition, the number of days per year suitable for outbreaks is presented for the Baltic (Figure 26). The percentage change figures reported in the main text were calculated relative to a 1980s baseline (8-year average, 1982-89), either an average for the 2010s (10-year average, 2011-2020) to illustrate the overall trend accounting for interannual variability or for the most recent year for which data were available (2020).

Data

1. Sea surface temperature data from the NOAA Optimum Interpolation 1/4 Degree Daily Sea Surface Temperature (OISST) Analysis version 2 for the period 1982-2019.[72]
2. Sea surface salinity data from the Mercator Ocean Reanalysis.[71]

Caveats

The results are derived on the basis of suitable SST and SSS conditions only, and do not include other potentially important drivers (e.g. globalisation), environmental predictors of pathogenic *Vibrio* infections (e.g., cholorphyll-*a*, turbidity) nor disease case data. Nevertheless, these associations have been explored and are reported in the supporting references included above.

In the global analysis, the slope of the trendlines over the time series is mostly flat for the tropical/subtropical region and the southern Hemisphere. However, the SST-only suitability shows a strong upward trend in the southern hemisphere, indicating that on average temperature conditions are also improving growth conditions for *Vibrio* in these areas, while SSS is generally limiting. However, locally suitable SSS conditions will also occur in these regions on the basis of, for example, variation in local rainfall and river runoff, which can make these regions sporadically suitable for *Vibrio* infections.

Future form of the indicator

The *Vibrio* indicator considers environmental factors only, seawater temperature and salinity. Socioeconomic and demographic aspects have however been identified as key elements in disease transmission of *Vibrio* illness. Future developments could include the use of climate models, such as those from the Inter-Sectoral Impact Model Intercomparison Project (ISIMIP), to include key socioeconomic drivers (economic growth, demography, education and technological development) in *Vibrio* suitability estimates.

Additional analysis

In addition to the area suitable for *Vibrio* outbreaks, the number of days suitable per year has doubled in the Baltic region, extending the highest risk season by around 6.4 weeks (from 38.4 days 1982-1989 to 83.3 days in 2011-2020, Figure 26).

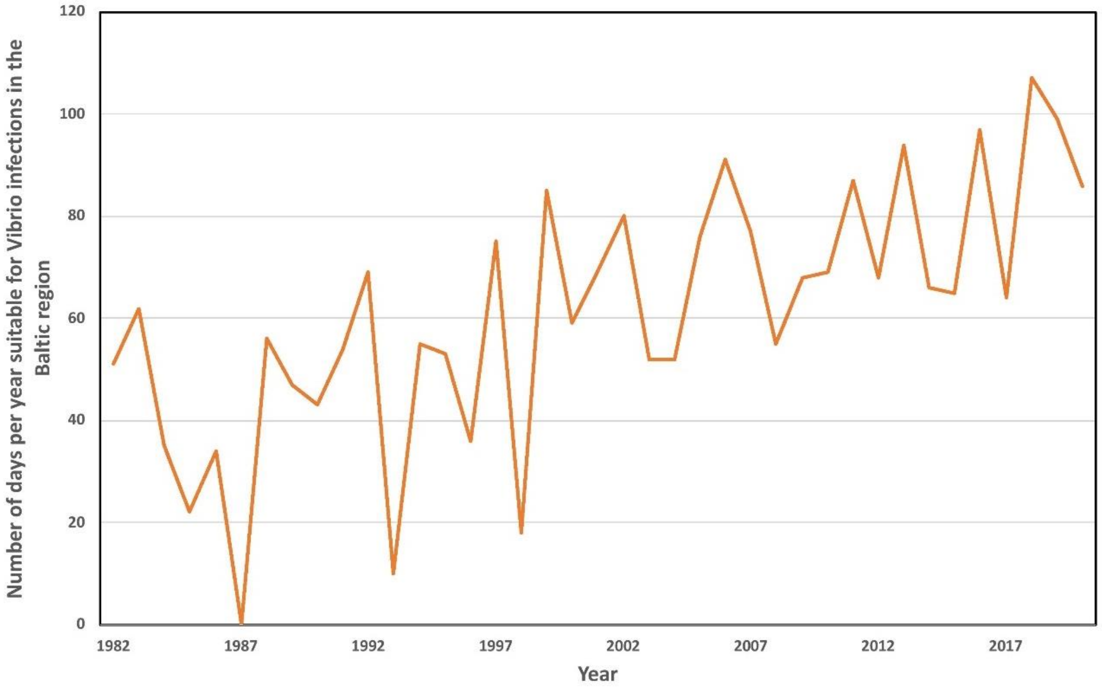

Figure 26. Annual number of days suitable for Vibrio infections in the Baltic Region.

This Latitude-time plot (Hovmoller diagram, Figure 27) indicates poleward expansion of suitable environments for *Vibrio* spp. in this region. For latitudes >39 and similarly to the Baltic Sea, there is a general widening of the *Vibrio* spp. season as well as an increase in the amount of shoreline affected.

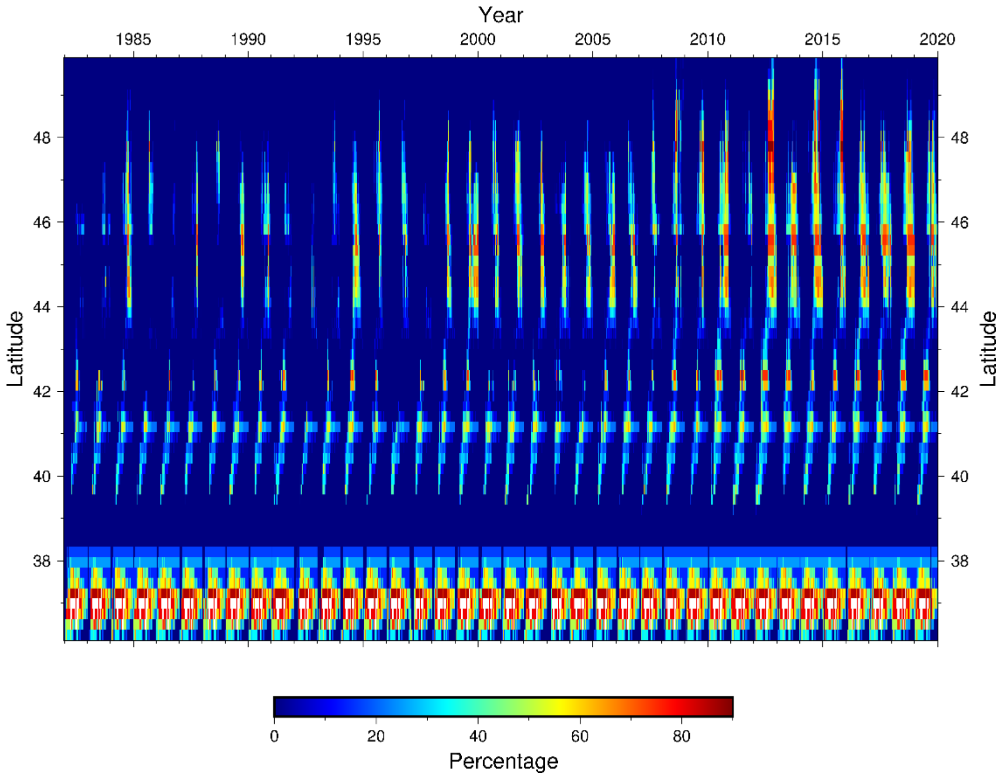

Figure 27. Percentage coastline suitable for Vibrio spp., V. parahaemolyticus, V. vulnificus, and non-toxigenic V. cholerae (non-O1/non-O139), by latitude along the USA northeast coastal region (36N-50N).

Vibrio cholerae

Methods

The methodology for this indicator was improved from the 2019 report of the Lancet Countdown, where it last appeared.[73]

Cholera is a water-borne disease caused by the bacterium *Vibrio cholerae*, which generally occurs in brackish riverine, estuarine, and coastal waters.[74] Toxigenic *V. cholerae* is responsible for epidemic cholera, while non-toxigenic *Vibrio cholerae* is responsible for sporadic cases of mild gastroenteritis, but not cholera.

Cholera prevention requires the understanding of the distribution and availability of its pathogen, toxigenic *V. cholerae*, and the role of the environmental conditions that facilitate or limit *V. cholerae* emergence and persistence. This indicator focuses on the areas suitable for the *V. cholerae* pathogen in coastal areas around the world. The abundance of *V. cholerae* is known to associated with increases in sea surface temperature and phytoplankton in coastal waters.[75] Thus, the distribution *V. cholerae sensu lato* was reconstructed using an ecological niche modelling approach that links *V. cholerae* reports and fine-scale sea surface temperature and phytoplankton in coastal waters during the period 2003-2009, and assumes niche conservatism among toxigenic and non-toxigenic lineages.[76] The analysis was performed in the exclusive economic zone of coastal marine areas globally. A distance of ~200 miles was calculated off the coast of each country to resemble the exclusive economic zone defined by the United Nations for country borders defined elsewhere (Figure 28).[77]

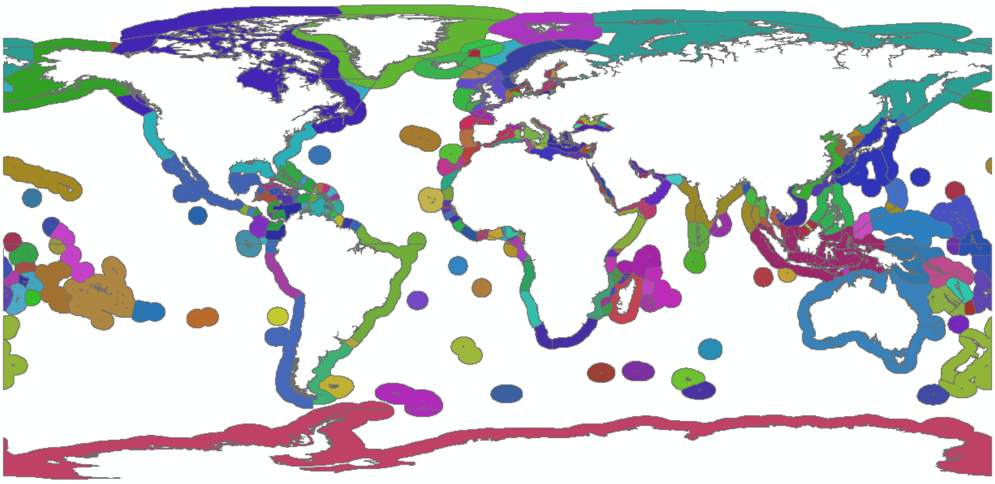

Figure 28. Exclusive economic zone of each country and territory around the world. Colours denote a different country or territory.

Analyses were performed following a novel ecological niche modelling protocol described in Figure 29. Specifically, (**a**) a comprehensive dataset of *V. cholerae* occurrence and seawater data was ensembled for the last two decades; (**b**) each *V. cholerae* record was carefully curated following standardized data-cleaning protocols to reduce bias and errors[78,79] and records were linked to seawater data of the site and date of *V. cholerae* sample collection; (**c**) an ecological niche model of *V. cholerae* was developed using a novel hypervolume model approach[80]; (**d**) the model was projected to seawater conditions globally between 2003 and 2019; (**e**) models were projected to the geography to identify the specific coastal areas suitable for *V. cholerae*; (**f**) historical *V. cholerae* suitable conditions were compared with a baseline of average conditions between 2003-2005 to identify variation across time.

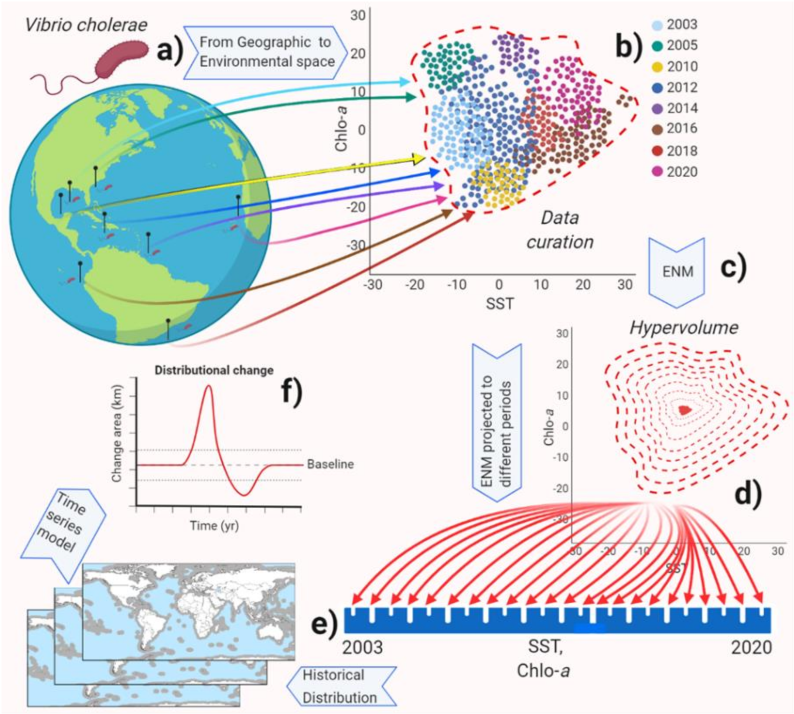

Figure 29. Analytical framework of the estimation of suitable coastal areas for V. cholerae. a) Geolocation of Vibrio cholerae records in coastal areas around the globe, limited by the exclusive economic zone in each country. b) Identification of seawater conditions where V. cholerae occurs using satellite-derived data of seawater temperature (SST) and chlorophyll-a (Chlo-a). Note that specific conditions of the year of the report is considered (points). c) Ecological niche modelling of Vibrio cholerae reports (dashed lines) using hypervolume modeling. d) Projection of the final model to the seawater conditions of every year since 2003. d) Projection of the models to the geography to measure the areas suitable for V. cholerae. f) Evaluation of changes in suitable areas across time.

V. cholerae records used to quantify the environmental tolerances of the species were recovered from the literature (Table 10).

Table 10. Sources of Vibrio cholerae records.

1. Atlantic, S., Martinelli Filho, J. E., Lopes, R. M., Rivera, I. N. G. & Colwell, R. R. *Vibrio cholerae* O1 detection in estuarine and coastal zooplankton. *J. Plankton Res.* **33**, 51–62 (2010).
2. Barbera, A. La *et al.* Aislamiento de *Vibrio* spp. y evaluación de la condición sanitaria de los moluscos bivalvos *Arca zebra* y *Perna perna* procedentes de la costa nororiental del Edo, Sucre, Venezuela. *FCU-LUZ* **14**, 513–521 (2004).
3. Batabyal, P., Mookerjee, S., Einsporn, M. H., Lara, R. J. & Palit, A. Environmental drivers on seasonal abundance of riverine-estuarine *V. cholerae* in the Indian Sundarban mangrove. *Ecol. Indic.* **69**, 59–65 (2016).
4. Binsztein, N. *et al.* Viable but nonculturable *Vibrio cholerae* O1 in the aquatic environment of Argentina. *Appl Env. Microbiol* **70**, 7481–7486 (2004).
5. Bliem, R., Reischer, G., Linke, R., Farnleitner, A. & Kirschner, A. Spatiotemporal dynamics of *Vibrio cholerae* in turbid alkaline lakes as determined by quantitative PCR. *Appl. Environ. Microbiol.* **84**, 1–14 (2018).
6. Dalusi, L., Lyimo, T. J., Lugomela, C., Hosea, K. M. M. & Sjöling, S. Toxigenic *Vibrio cholerae* identified in estuaries of Tanzania using PCR techniques. *FEMS Microbiol. Lett.* **362**, fnv009 (2015).
7. de Menezes, F. G. R. *et al.* Detection of virulence genes in environmental strains of *Vibrio cholerae* from estuaries in northeaster Brazil. *Rev. Inst. Med. Trop. Sao Paulo* **56**, 427–432 (2014).
8. de Menezes, F. G. R. *et al.* Pathogenic *Vibrio* species isolated from estuarine environments (Ceará, Brazil) - antimicrobial resistance and virulence potential profiles. *An. Acad. Bras. Cienc.* **89**, 1175–1188 (2017).
9. Dheenan, P. S. *et al.* Spatial variation of physicochemical and bacteriological parameters elucidation with GIS in Rangat Bay, Middle Andaman, India. *J. Sea Res.* **85**, 534–541 (2014).
10. Di, D. Y. W., Lee, A., Jang, J., Han, D. & Hur, H. G. Season-specific occurrence of potentially pathogenic *Vibrio* spp. on the southern coast of South Korea. *Appl. Environ. Microbiol.* **83**, 1–13 (2017).
11. Escobar, L. E. *et al.* A global map of suitability for coastal *Vibrio cholerae* under current and future climate conditions. *Acta Trop.* **149**, 202–211 (2015).
12. Esteves, K. *et al.* Rapid proliferation of *Vibrio parahaemolyticus*, *Vibrio vulnificus*, and *Vibrio cholerae* during freshwater flash floods in french mediterranean coastal lagoons. *Appl. Environ. Microbiol.* **81**, 7600–7609 (2015).
13. Esteves, K. *et al.* *Vibrio cholerae* during freshwater flash floods in french mediterranean coastal lagoons. *Front. Microbiol.* **81**, 7600–7609 (2015).

14. Fang, L., Ginn, A. M., Harper, J., Kane, A. S. & Wright, A. C. Survey and genetic characterization of Vibrio cholerae in Apalachicola Bay, Florida (2012–2014). *J. Appl. Microbiol.* **126**, 1265–1277 (2019).
15. Fernández-Delgado, M. *et al.* Occurrence and virulence properties of *Vibrio* and *Salinivibrio* isolates from tropical lagoons of the southern Caribbean Sea. *Antonie Van Leeuwenhoek* **110**, 833–841 (2017).
16. Fri, J., Ndip, R. N., Njom, H. A. & Clarke, A. M. Occurrence of virulence genes associated with human pathogenic vibrios isolated from two commercial Dusky Kob (*Argyrosmus japonicus*) farms and kareiga estuary in the Eastern Cape Province, South Africa. *International Journal of Environmental Research and Public Health* **14**, (2017).
17. Gardade, L. & Khandeparker, L. Spatio-temporal variations in pathogenic bacteria in the surface sediments of the Zuari estuary, Goa, India. *Curr. Sci.* **113**, 1729–1738 (2017).
18. Gdoura, M. *et al.* Molecular detection of the three major pathogenic vibrio species from seafood products and sediments in Tunisia using real-Time PCR. *J. Food Prot.* **79**, 2086–2094 (2016).
19. Grothen, D. C., Zach, S. J. & Davis, P. H. Detection of intestinal pathogens in river, shore, and drinking water in Lima, Peru. *J. Genomics* **5**, 4–11 (2017).
20. Gyraite, G., Katarzyte, M. & Schernewski, G. First findings of potentially human pathogenic bacteria *Vibrio* in the south-eastern Baltic Sea coastal and transitional bathing waters. *Mar. Pollut. Bull.* **149**, 110546 (2019).
21. Hackbusch, S., Wichels, A., Gimenez, L., Döpke, H. & Gerdts, G. Potentially human pathogenic *Vibrio* spp. in a coastal transect: Occurrence and multiple virulence factors. *Sci. Total Environ.* **707**, 136113 (2020).
22. Izumiya, H. *et al.* A double-quadratic model for predicting *Vibrio* species in water environments of Japan. *Arch. Microbiol.* **199**, 1293–1302 (2017).
23. Khamesipour, R. M., Rahimi, E. & Khodadoostan, A. Occurrence of *Vibrio* spp., Aeromonas hydrophila, *Escherichia coli* and *Campylobacter* spp. in crayfish (*Astacus leptodactylus*) from Iran. *Iran. J. Fish. Sci.* **13**, 944–954 (2014).
24. Kim, J. Y. & Lee, J. L. Multipurpose assessment for the quantification of *Vibrio* spp. and total bacteria in fish and seawater using multiplex real-time polymerase chain reaction. *Journal of the Science of Food and Agriculture* **94**, 2807–2817 (2014).
25. Kokashvili, T. *et al.* Occurrence and Diversity of clinically important *Vibrio* species in the aquatic environment of Georgia. *Front. Public Heal.* **3**, 1–12 (2015).
26. Lipp, E. K. E. *et al.* Direct detection of *Vibrio cholerae* and ctxA in Peruvian coastal water and plankton by PCR. *Appl. Env. Mircrobiol* **69**, 3676 (2003).
27. López, L. *et al.* Estudio piloto para el aislamiento de *Vibrio* spp. en ostras (*Crassostera rhizophorae*) capturadas en la Ciénaga de la Virgen, Cartagena, Colombia. *RSPYN* **11**, 1–6 (2010).
28. Louis, V. R. *et al.* Predictability of *Vibrio cholerae* in Chesapeake Bay. *Appl Env. Microbiol* **69**, 2773–2785 (2003).
29. Machado, A. & Bordalo, A. A. Detection and quantification of *Vibrio cholerae*, *Vibrio parahaemolyticus*, and *Vibrio vulnificus* in Coastal Waters of Guinea-Bissau (West Africa). *Ecohealth* **13**, 339–349 (2016).
30. Main, C. R., Salvitti, L. R., Whereat, E. B. & Coyne, K. J. Community-level and species-specific associations between phytoplankton and particle-associated *Vibrio* species in Delaware's Inland Bays. *Appl. Environ. Microbiol.* **81**, 5703–5713 (2015).
31. Matteucci, G., Schippa, S., Di Lallo, G., Migliore, L. & Thaller, M. C. Species diversity, spatial distribution, and virulence associated genes of culturable vibrios in a brackish coastal Mediterranean environment. *Ann. Microbiol.* **65**, 2311–2321 (2015).
32. Meena, B. *et al.* Studies on diversity of *Vibrio* sp. and the prevalence of hapA, tcpI, st, rtxA&C, acfB, hlyA, ctxA, ompU and toxR genes in environmental strains of *Vibrio cholerae* from Port Blair bays of South Andaman, India. *Marine Pollution Bulletin* 105–116 (2019). doi:10.1016/j.marpolbul.2019.05.011
33. Meyer, J. L., Gunasekera, S. P., Scott, R. M., Paul, V. J. & Teplitski, M. Microbiome shifts and the inhibition of quorum sensing by Black Band Disease cyanobacteria. *ISME J.* **10**, 1204–1216 (2016).
34. Ming, H. *et al.* Enterococci may not present the pollution of most enteric pathogenic bacteria in recreational seawaters of Xinghai bathing Beach, China. *Ecol. Indic.* **110**, 105938 (2020).
35. Mukhopadhyay, A. K. A. *et al.* Molecular epidemiology of reemergent *Vibrio cholerae* O139 Bengal in India. *J. Clin. Microbiol.* **36**, 2149–2152 (1998).
36. Neogi, S. B. *et al.* Environmental and hydroclimatic factors influencing *Vibrio* populations in the estuarine zone of the Bengal delta. *Environ. Monit. Assess.* **190**, 565 (2018).
37. Orozco, R. *et al. Evaluación de la constaminación y calidad microbiológica del agua de mar en las bahías de Ferrol y Samanco*. **56**, (Instituto del Mar del Peru, 1996).
38. Pal, B. B., Khuntia, H. K., Samal, S. K., Das, S. S. & Chhotray, G. P. Emergence of *Vibrio cholerae* O1 biotype El Tor serotype Inaba causing outbreaks of cholera in Orissa, India. *Jpn J infect Dis* **59**, 266–9 (2006).
39. Pascual, M., Rodó, X., Ellner, S. P., Colwell, R. R. & Bouma, M. J. Cholera dynamics and El Nino-Southern Oscillation. *Science* **289**, 1766–1769 (2000).
40. Perkins, T. L. *et al.* Sediment composition influences spatial variation in the abundance of human pathogen indicator bacteria within an estuarine environment. *PLoS ONE* **9**, e112951 (2014).
41. Sack, R. B. *et al.* A 4-year study of the epidemiology of *Vibrio cholerae* in four rural areas of Bangladesh. *J. Infect. Dis.* **21205**, 96–101 (2003).
42. Siboni, N., Balaraju, V., Carney, R., Labbate, M. & Seymour, J. R. Spatiotemporal dynamics of *Vibrio* spp. within the Sydney harbour estuary. *Front. Microbiol.* **7**, 460 (2016).

43. Silva, M. M. *et al.* Dispersal of potentially pathogenic bacteria by plastic debris in Guanabara Bay, RJ, Brazil. *Mar. Pollut. Bull.* **141**, 561–568 (2019).
44. Sneha, K. G. *et al.* Distribution of multiple antibiotic resistant *Vibrio* spp across Palk Bay. *Reg. Stud. Mar. Sci.* **3**, 242–250 (2016).
45. Sulca, M. A., Orozco, R. & Alvarado, D. E. Antimicrobial resistance not related to 1,2,3 integrons and Superintegron in *Vibrio* spp. isolated from seawater sample of Lima (Peru). *Mar. Pollut. Bull.* **131**, 370–377 (2018).
46. Wong, Y. Y. *et al.* Environmental control of *Vibrio* spp. abundance and community structure in tropical waters. *FEMS Microbiol. Ecol.* **95**, fiz176 (2019).
47. Xu, M., Kan, B. & Wang, D. Identifying environmental risk factors of cholera in a coastal area with geospatial technologies. *Int. J. Environ. Res. Public Health* **12**, 354–370 (2015).
48. Yue, Y. *et al.* Influence of climate factors on *Vibrio cholerae* dynamics in the Pearl River estuary, South China. *World J. Microbiol. Biotechnol.* **30**, 1797–1808 (2014).
49. Zaw, M. T. *et al.* Genetic diversity of toxigenic *Vibrio cholerae* O1 from Sabah, Malaysia 2015. *J. Microbiol. Immunol. Infect.* **52**, 563–570 (2019).
50. Zhang, X., Song, Y., Liu, D., Keesing, J. K. & Gong, J. Macroalgal blooms favor heterotrophic diazotrophic bacteria in nitrogen-rich and phosphorus-limited coastal surface waters in the Yellow Sea. *Estuar. Coast. Shelf Sci.* **163**, 75–81 (2015).

V. cholerae occurrence data included reports from coastal regions, corroborated in laboratory facilities, and geolocated with an uncertainty <2km following the protocol described by Escobar et al. 2015.[81] To account for geolocation uncertainty and water displacement, seawater conditions were collected in immediate pixels neighbouring each *V. cholerae*. All cells with duplicate environmental values were removed to reduce bias (Figure 30).

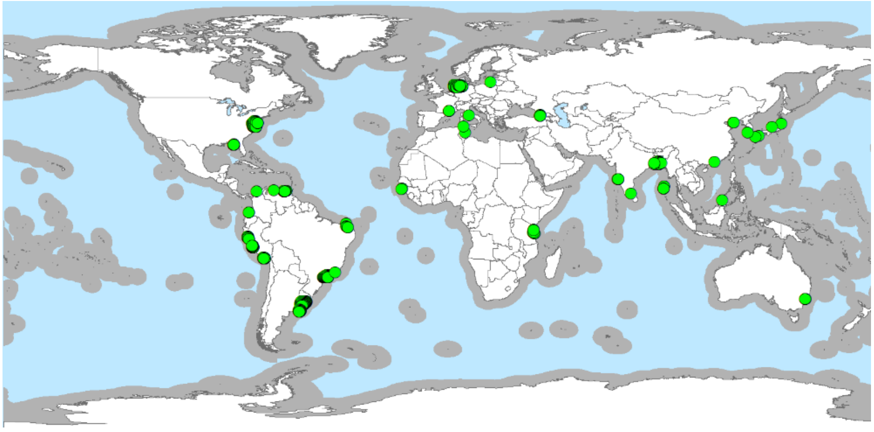

Figure 30. Study area and geolocation of Vibrio cholerae in coastal areas. Exclusive economic zone around the world (grey) was used to limit the satellite-derived data of seawater conditions in coastal areas. Vibrio cholerae reports (green points) in these areas were used for model calibration.

To reconstruct the seawater conditions where *V. cholerae* could survive, sea surface temperature was considered as a proxy of seawater temperature and chlorophyll-*a* as a proxy of phytoplankton. Sea surface temperature and chlorophyll-*a* have been found to be main drivers of *V. cholerae* occurrence.[75,81,82] Annual mean, range, standard deviation, maximum, and minimum values of these oceanographic variables were estimated between 2003 and 2019. Variables were compiled at 4 km^2 pixel size in the exclusive economic zone of each country around the world. Correlated variables (correlation coefficient $\geq |0.3|$) were removed to reduce redundancy and dimensionality in the final model.

Suitable seawater conditions for *V. cholerae* were determined by estimating the realized ecological niche of the bacterium.[78] The realized ecological niche was reconstructed by linking each *V. cholerae* report with sea surface temperature and chlorophyll-*a* values of the year of the sampling. Temperature and phytoplankton proxies resembling abiotic and biotic factors shaping the species ecology were included, respectively.[83] Niche models were developed in an environmental space using one-class support vector machine (SVM). SVM is a machine-learning algorithm that determines clusters of the coastal conditions where *V. cholerae* has occurred.[84] In ecological niche modelling, SVM is a presence-only modelling method that allows a smooth fit of the model around data, and is insensitive to outliers during the estimation of a boundary that classifies coastal conditions data as "in" or "out" of the high-dimensional hypervolume of *V. cholerae*.[80] In other words, one-class SVM only

needs sites with *V. cholerae* lover reports, overcoming data limitations of classic data-hungry machine-learning classification methods that require information of sites with and without *V. cholerae* for model calibration. The SVM model was built using the *hypervolume* package in R following the developers' protocol.[85]

The final ecological niche model was then projected to all years to generate a time-series analysis of suitable coastal areas for *V. cholerae* between 2003 and 2019. The coastal areas predicted suitable for *V. cholerae* by country was used as a proxy of sites where the bacterium could successfully survive and establish if introduced.

Sea surface temperature and chlorophyll-*a* data across coastal areas were collected from the Moderate Resolution Imaging Spectroradiometer (MODIS) sensor in the Aqua satellite at 4 km^2 spatial resolution and monthly temporal resolution during the period from January 2003 to December 2019. Monthly and annual summary statistics from each variable, including mean, range, standard deviation, maximum, and minimum values, were estimated and are openly available.[86] These variables coupled with *Vibrio cholerae* coastal reports were used during model calibration.

Data

1. Sea surface temperature and chlorophyll-*a* data from the Moderate Resolution Imaging Spectroradiometer (MODIS) sensor in the Aqua satellite.[87,88]

Caveats

Vibrio cholerae is not habitually surveyed in coastal waters or in environmental samples in general.[82] Instead, *V. cholerae* reports generally originate from human cases in inland areas. The limited number of reports used in this modelling framework could result in an underestimation of the epidemiological potential of *V. cholerae* in coastal waters around the world. To mitigate this limitation, on the environmental conditions most likely suitable for the species were considered using an hypervolume model calibrated with three parameters: annual mean chlorophyll, and annual maximum and range of sea surface temperature.

This coarse-scale assessment focused in only one component of cholera transmission risk: the environmental conditions where *V. cholerae* could establish in coastal waters. Nevertheless, while the model predicts conditions where *V. cholerae* could survive, it does not assume or predict *V. cholerae* presence. Similarly, fine-scale variables, such as water sanitation, play a key role in the expression of cholera disease in areas where *V. cholerae* actually occurs. Finally, *V. cholerae* at the species level were modelled assuming that toxigenic and non-toxigenic lineages would respond similarly to environmental conditions.

Future form of the indicator

Sea surface temperature and chlorophyll-*a* conditions in future years will allow to determine *V. cholerae* suitability, change in suitability across time, and the location and intensity of the change in coastal waters around the world. New satellite-derived data will allow determining whether trends observed in this analysis are consistent in the coming years.

Additional analysis

Whereas some locations show stability or decrease in their suitability for *V. cholerae*, a consistent trend to increase *V. cholerae*'s coastal suitability was detected in some regions, with a particularly strong signal for the past five years (Figure 31). The European region shows an exceptional inconsistency with regards to *V. cholerae*'s coastal suitability. A more detailed analysis of coastal Europe suggests abrupt variation in seawater condition (Figure 32), which could explain the signal captured in the time-series analysis.

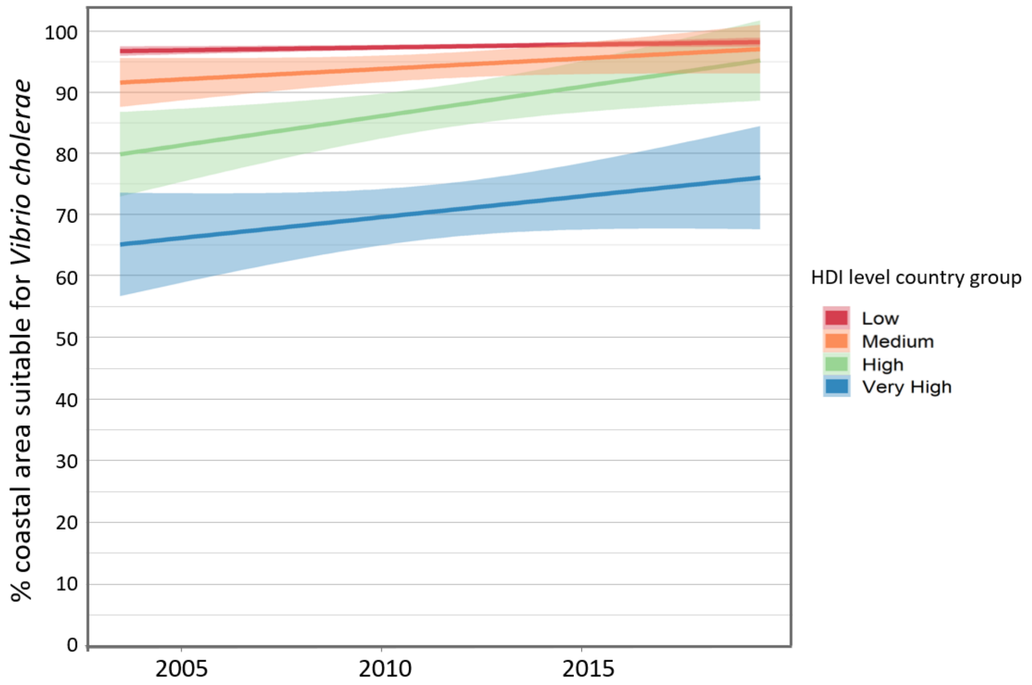

Figure 31. Change in environmental suitability for Vibrio cholerae based on changes in sea surface temperatures and chlorophyll-a concentration between 2003 and 2019 by country HDI group. Shaded areas represent 95% confidence intervals

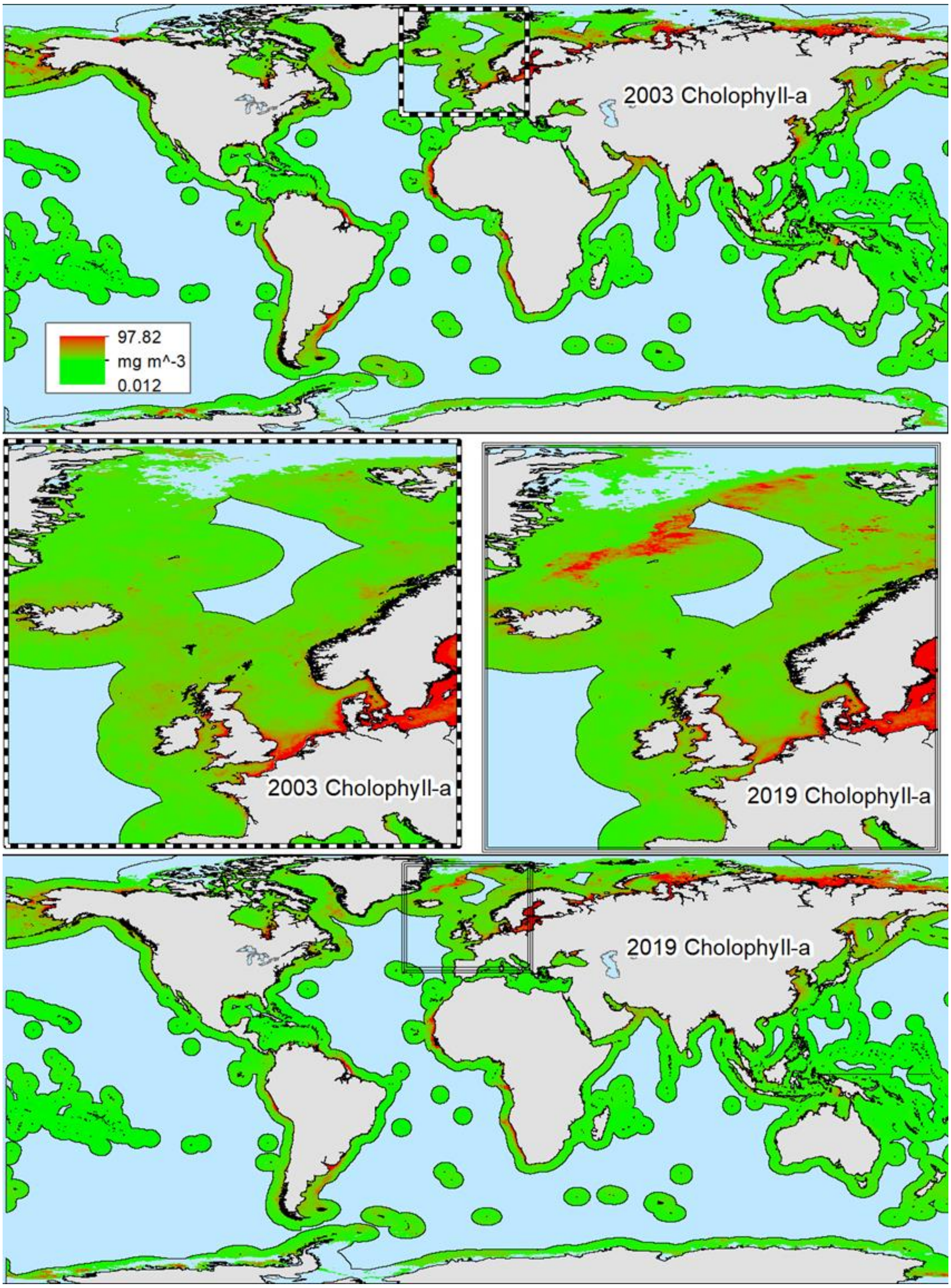

Figure 32. Chlorophyll-a conditions 2003 vs. 2019 in coastal areas globally. Note the abrupt changes in environmental conditions in coastal areas of the European region.

Indicator 1.3.2: Vulnerability to Mosquito-Borne Diseases

Methods

This indicator computes the Vulnerability Index (VI) of a given country to dengue, considering susceptibility and coping capacity variables. Specifically, this indicator displays vulnerabilities overlayed with the basic reproduction number (R_0) for the transmission of dengue by *Aedes aegypti* and *Aedes albopictus* for each country, as described in Indicator 1.3.1. Values were aggregated by WHO region and HDI levels. Vulnerability was calculated by scaling the $R_0$for to range between 0 and 100 and then multiplying by the geometric mean of the following vulnerability indicators: percentage of population without access to drinking water services (Pop_{DWS}),[89] the Gross National Income per capita (GNI),[90] and the percentage of deaths by communicable diseases and maternal, prenatal and nutrition conditions as a proxy of healthcare access (HCA).[91]

$$VI = scaled\ R_0\ \ x\ (Pop_{DWS} * GNI * HCA) \wedge 1/3$$

Data

1. Climate research unit (CRU) TS vs 4.0.3 precipitation and temperature data.[57]
2. WHO/UNICEF Joint Monitoring Programme (JMP) for Water Supply, Sanitation and Hygiene.[89]
3. Gross national income (GNI) per capita (constant 2017 PPP$) data are taken from the United Nations Development Programme.[90]
4. Data on maternal, prenatal and nutrition conditions were taken from the 2019 Global Burden of Disease Study 2019.[91]

Caveats

The abundance models generate predictions and not observed frequencies in relation to climate conditions, and by so should be considered a potential abundance estimate. A reduction of this indicator while keeping the vector hazard constant does not correspond to full protection but indicates rather that the situation has improved by important improvements in core capacities.

Future form of the indicator

This indicator will be updated to capture a more comprehensive risk index, including further measurements of population vulnerability alongside adaptive capacity. It may also be updated to include vectorial capacity for the transmission of Yellow Fever, Chikungunya, and Zika. It will also strive to include more model estimates of vector to human densities (m), going beyond the presence/absence approach of vectors used here.

Additional analysis

Figure 33 presents vulnerability transmission, grouped by 2019 HDI levels, for 2000 to 2017.

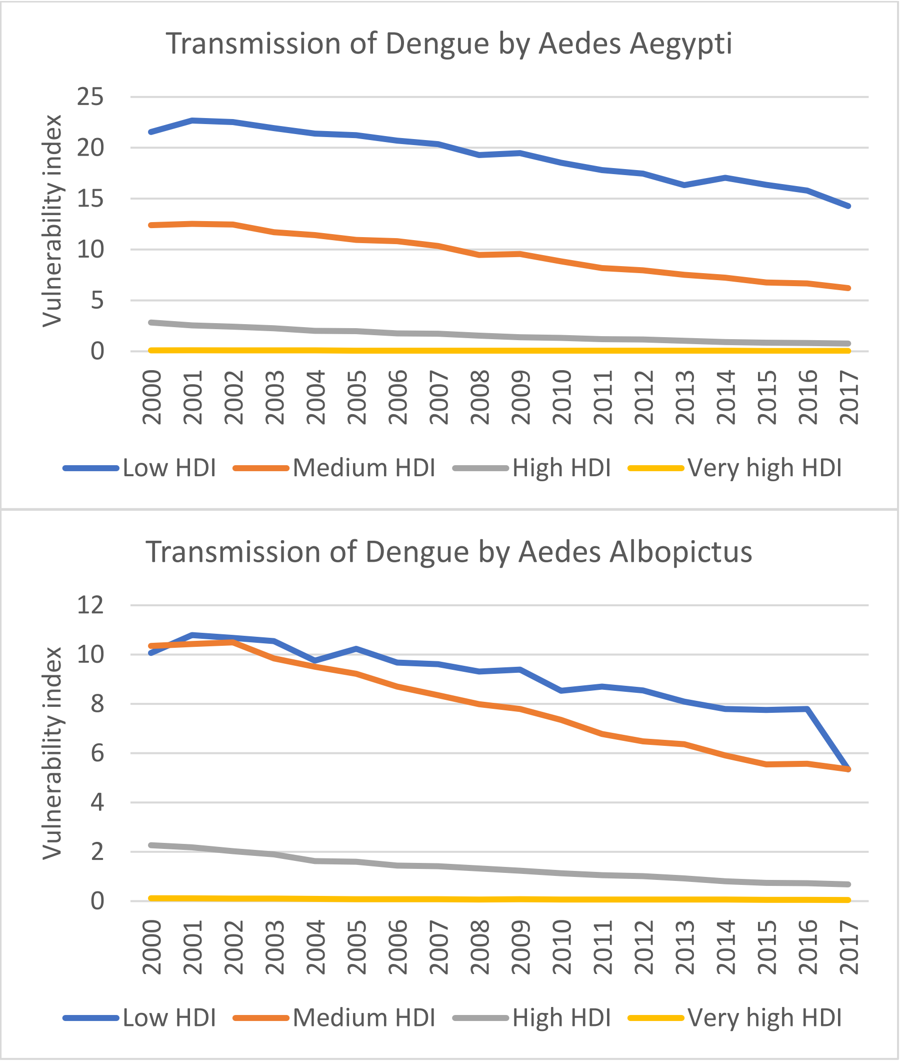

Figure 33. vulnerability to dengue transmission by A. albopictus and A. aegypti, by 2019 country HDI level

1.4: Food Security and Undernutrition

Indicator 1.4.1: Terrestrial Food Security and Undernutrition

Methods

The methodology for this indicator has been updated and extended from the 2020 report. This year it presents the indicator as described in the previous report (accumulated temperature total as a proxy for crop yield potential), augmented with a first attempt of tracking the impact of climate change and inequality on incidence of severe food insecurity.

The crop yield potential is track with a methodology that remains similar to that described in the 2020 report of the Lancet Countdown.[1] Briefly, crop duration is defined as the time taken in a year to accumulate the reference period (1981-2010) average growing season accumulated temperature total (ATT).[92] If the ATT is reached early, then the crop matures too quickly and yields are lower than average.[92,93] Here, the crop duration loss was defined as the percentage change in the time taken (in days) to accumulate the average growing season accumulated temperature.

Crop yield potential is calculated across the area of land under cultivation[94] at 0.25° x 0.25°, and then area-weighted averaged. Climate data is taken from the monthly historical records from ECMWF ERA5 climate reanalysis dataset between January 1980 and December 2020, and synthetic daily data is estimated for each grid cell by applying a regional average daily anomaly to the monthly value. The plot in the paper shows the global average annual change in crop growth duration. The horizontal line shows the average difference in crop growth duration over the reference period 1981-2010.

New to 2021, the indicator is extended to track the proportion of the population suffering from climate-related severe food insecurity.

This indicator tracks the association between temperature increase and food insecurity. It uses a panel data regression with coefficients which vary over time, to estimate the association between temperature anomaly in °C and food insecurity for every year. The dependent variable is the probability of severe food insecurity in a country (see below). The model controls for temperature anomaly (annual temperature difference from mean temperature of a 30-year period between 1989-2019, ERA5-Land reanalysis data), HDI, and location (country) and time (year) fixed-effects to account for unobserved heterogeneity. For each regression coefficient, 95% confidence intervals and standard p-values are reported.

Prevalence of moderate or severe food insecurity based on the Food Insecurity Experience Scale (FIES) provides internationally-comparable estimates of the proportion of the population facing difficulties in accessing food. The FIES-based indicators are compiled using the FIES survey module, containing eight questions, which are then used to compute the proportion of the population experiencing moderate or severe food insecurity and the proportion of the population experiencing severe food insecurity.

The HDI database contains data for 1,625 regions within 161 countries, with national and subnational values of HDI. Aggregated subnational HDI were used in the analysis.

Countries included in the analysis are: Afghanistan, Angola, Albania, Argentina, Armenia, Australia, Austria, Azerbaijan, Belgium, Bangladesh, Bulgaria, Bosnia and Herzegovina, Brazil, Botswana, Switzerland, Costa Rica, Czechia, Germany, Denmark, Algeria, Egypt, Spain, Estonia, Ethiopia, Finland, France, United Kingdom, Georgia, Guinea, Gambia, Greece, Guatemala, Honduras, Croatia, Hungary, Ireland, Islamic Republic of Iran, Iceland, Italy, Japan, Cambodia, Republic of Korea, Kuwait, Liberia, Libya, Lithuania, Luxembourg, Latvia, Republic of Moldova, Mexico, North Macedonia, Malta, Montenegro, Mongolia, Mozambique, Mauritania, Mauritius, Malaysia, Netherlands, Norway, Nepal, New Zealand, Peru, Philippines, Poland, Portugal, Romania, Russian Federation, Senegal, Singapore, El Salvador, Serbia, Slovakia, Slovenia, Sweden, Eswatini, Tajikistan, Tunisia, Ukraine, Uruguay, Uzbekistan, South Africa and Zimbabwe

Data

1. Climate data from the European Centre for Medium-Range Weather Forecasts (ECMWF) ERA5 and ERA-Land reanalysis.[4,95]
2. Data for crop areas from MIRCA2000.[94]
3. SAGE crop calendar from Sacks et al 2010.[96]
4. Food insecurity data from the FAO Food Insecurity Experience Scale.[97]

Caveats

The crop yield potential, as calculated here, does not take into account water shortage, and therefore characterises long-term change in yield potential rather than year to year variability.

Future forms of the indicator

In future reports the spatial coverage of the Food Insecurity Experience Scale data will be expanded.

Additional analysis

In depth analysis by subnational HDI reveals that a one unit (0.1 on a scale of 1) increase in the HDI is associated with a 2.3% decrease in probability of food insecurity. The median increase in HDI in a sample of 83 countries over 30-years is 0.11. A higher probability of food insecurity among women compared to men was reported in 54% of the countries included in this analysis Figure 35.

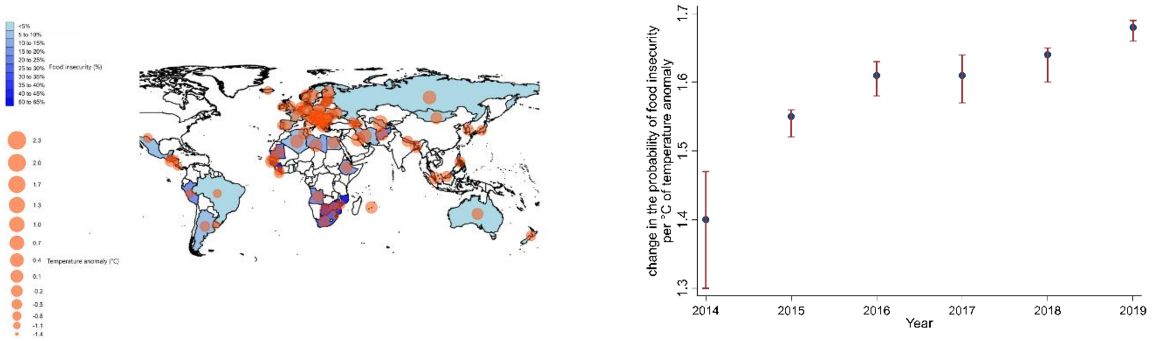

Figure 34. Left-panel: Temperature anomaly (°C) and probability of food insecurity. Right-panel: Time-varying impact of temperature anomaly and probability of food insecurity. Whiskers represent 95% confidence intervals.

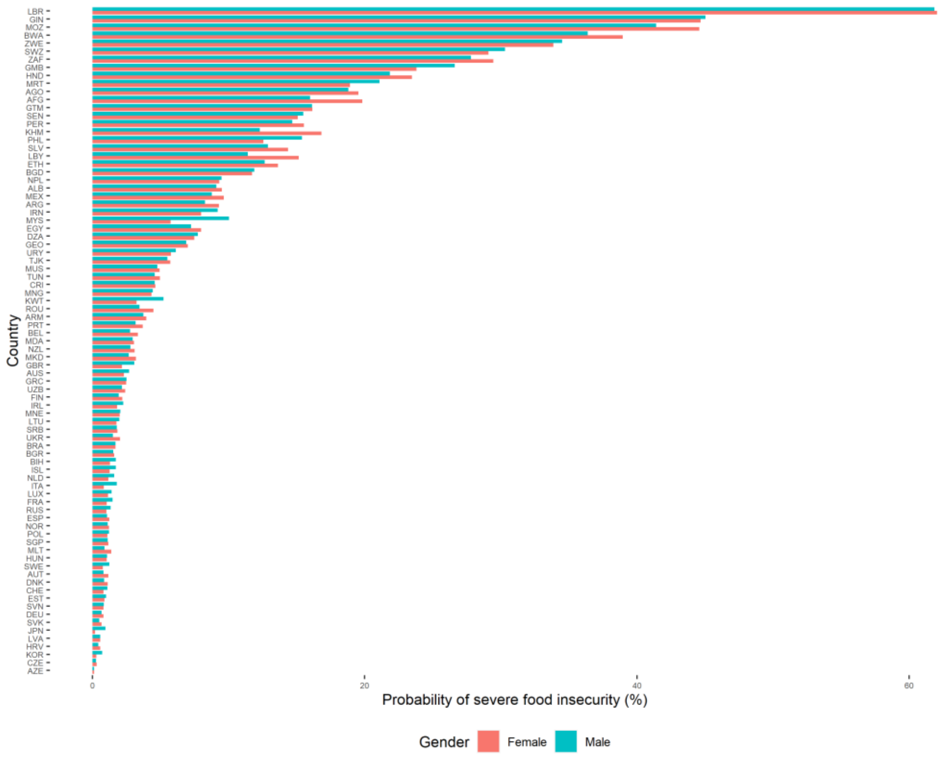

Figure 35. Probability of severe food insecurity by sex and country.

Indicator 1.4.2: Marine Food Security and Undernutrition

Methods

A large proportion of the global population, especially in low-income and middle-income countries, are largely dependent on fish sources of protein.[98] Sea surface temperature rise is among the well accepted consequences of climate change.[99] The resultant thermal stress can sequentially impair marine fish capacity and capture including through bleaching of coral reefs. To compensate for the reduced marine fish production, countries have geared up farm-based fish production.

The methodology for this indicator applies to the same marine basins and countries as described in the 2019 Lancet Countdown report.[73] Sixteen FAO (Food and Agriculture Organization of the United Nations) fishing areas (3 areas located in Antarctica were excluded) which are important in terms of projected impacts and vulnerabilities associated with climate change were selected. One-hundred thirty-six countries located in these basins were chosen in order to assess changes in Sea Surface Temperature SST, as well as the deterioration of major coral reef sites and the decreased consumption of capture-based fish.

Data

1. Data for SST was obtained from NASA Earth Observations (NEO) and covers the period from 2003 to 2020.[100]
2. The location of coral reef sites and data on annual maximum bleaching alert area caused by thermal stress was obtained from NOAA Coral Reef Watch Zones. Data is provided in five-year intervals from 1985 to 2020.[101]
3. Data on capture-based and farmed-based fish consumption per capita from 1980 to 2017 was collected from FAO.[102]

Caveats

There is a lack of information and data in the available databases such as FAO on fish species composition of the captured and farmed fish products. This could in turn lead to some concerns about the methodological approach used to calculate ω3 intake. More specifically, most of the approaches are based on fish intake, which usually ignore or underestimate variations in ω3 contents of different types of fishes, and especially capture-based compared with farmed-based fish.

The territorial waters of a number of countries investigated, namely Canada, Nicaragua, Spain, Australia, and Indonesia, are located in more than one marine basin. Since fish capture data is reported based on countries and not marine basins, this could potentially introduce a level of uncertainty in the association between SST and fish capture.

Future form of the indicator

Future form of the indicator may combine spatiotemporal data on SST, capture, and types of captured and consumed fish species. This will allow to better estimate the exposure to a diet low in ω3 contents and its attributable health burden. Since the geographical coordinates for some of the data are not available, i.e., fish capture and health data, in the next step, the level of details on location will need to be defined. For instance, marine basin will be included in fish capture analysis as a variable.

Additional analysis

As explained in previous reports,[1,73] despite a general increase in per capita fish consumption globally, the share of marine capture-based in total fish consumption has been decreasing (Figure 36). The increasing sea surface temperature well supports the decline in marine capture and the consequent thermal stress-induced deteriorating coral bleaching (Figure 37, Figure 38). Despite total per capita fish consumption, exposure to diet low in seafood ω3 has remained stable at the global level between 1990-2019 and is still very high in 2019 (1990 exposure level: 96.4%, 95% uncertainty interval: 93.3%-99.9% *vs.* 2019 exposure level: 93.5%, 95% uncertainty interval: 88.7-

99.4%). Notably, in countries showing improvements (declines) in exposure to diet low in seafood ω3, which are mainly among the countries with very high/high, gender inequality in exposure is widening against women (Figure 39). In other countries, exposure to this risk factor is still close to 100% in both genders.

Figure 41 presents the trend of capture-based per capita fish consumption, a key source of ω3 fatty acids, as well as the corresponding trend for farm-based per capita fish consumption for the 136 countries considered in the indicator. The two trends are different: farm-based fish consumption has increased constantly during the last 4 decades whereas capture-based fish consumption has been decreasing since the mid-90s. It is worth noting, however, that the consumption trends are heterogeneous among different countries.

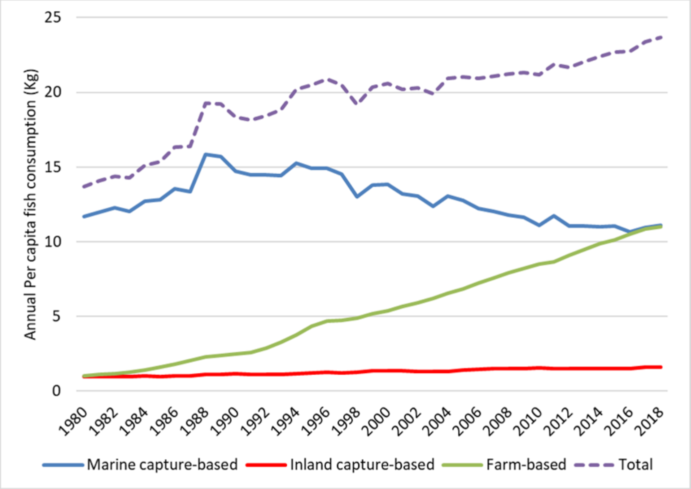

Figure 36. Population weighted average fish consumption per capita in 136 selected countries, separated by the origin of fish (marine capture-based, inland capture-based, and farm-based).

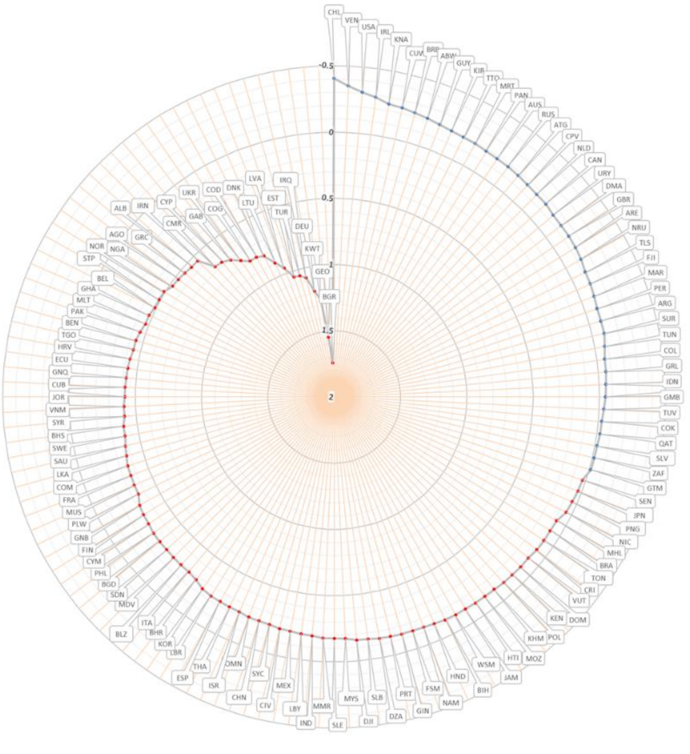

Figure 37: Changes in sea surface temperature (°C) for the territorial waters of the 136 countries investigated located in different basins (FAO fishing areas): 2018-20 average compared to 2003-05. Source: Sea Surface Temperature (MODIS), NASA Earth Observations (NEO); available at https://neo.sci.gsfc.nasa.gov/

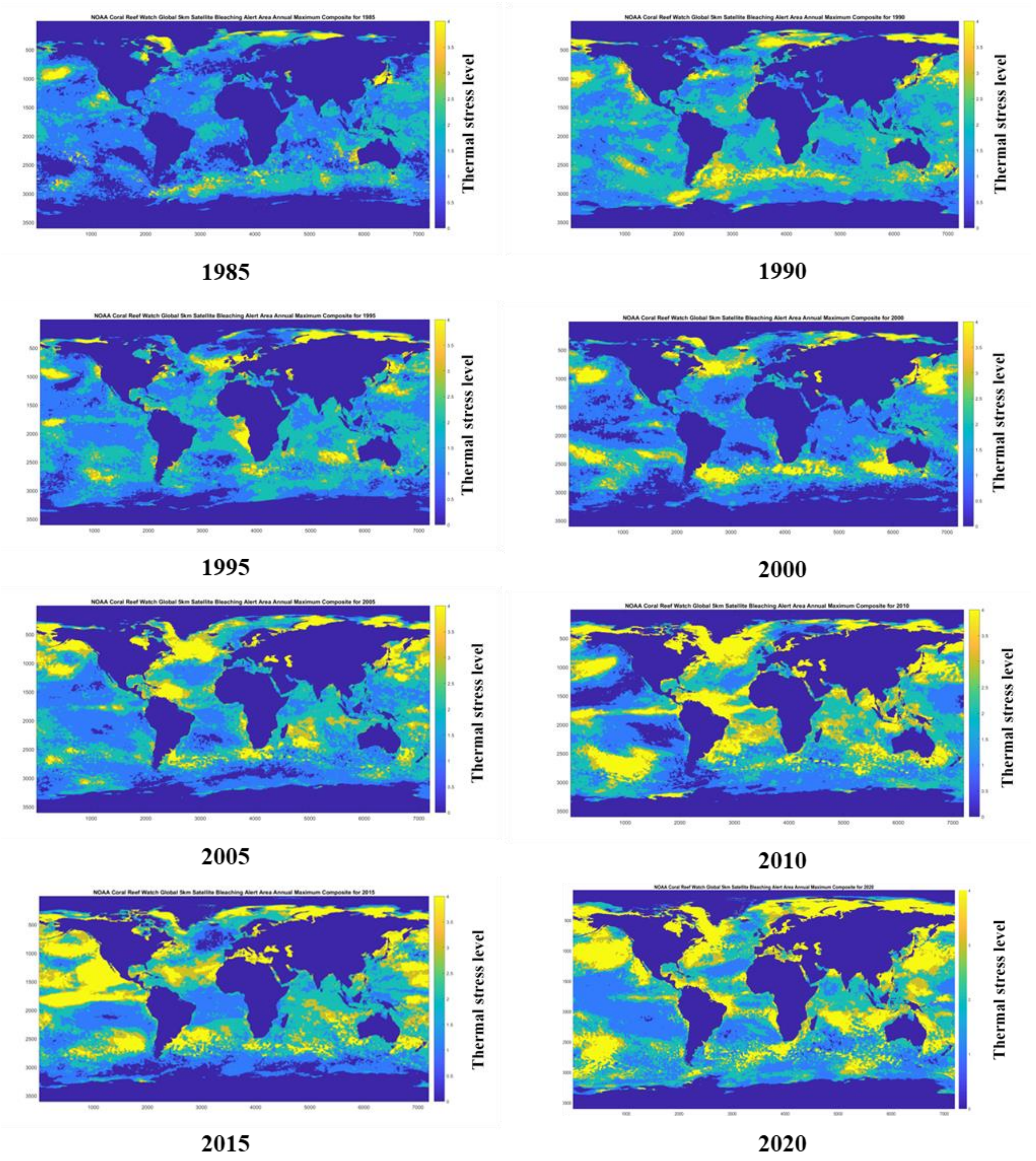

Figure 38: Comparing annual maximum Bleaching Alert Area caused by thermal stress in five-year intervals (1985-2020). Source: NOAA Coral Reef Watch. 1985, updated daily. NOAA Coral Reef Watch Global 5km Satellite Bleaching Alert Area Annual Maximum Composite Version 3.1, Jan. 01, 2020-Jan. 01, 2021. College Park, Maryland, USA: NOAA Coral Reef Watch. Data set accessed 2021-01-16 at https://coralreefwatch.noaa.gov/product/5km/index.php.

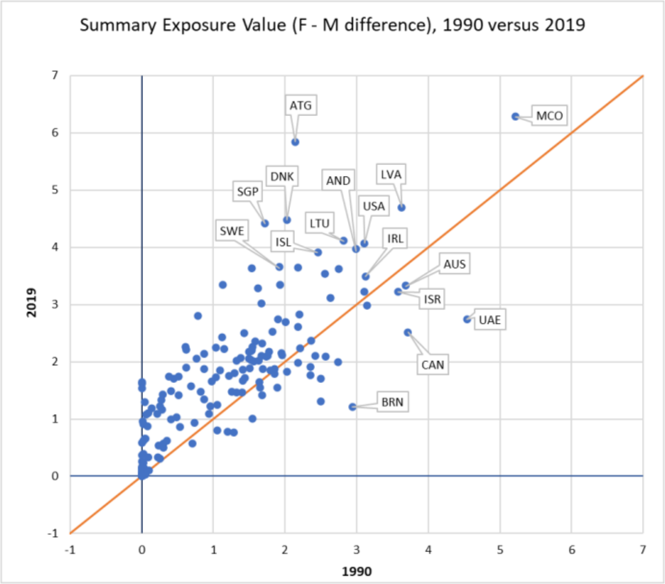

Figure 39: Absolute inequality in Summary Exposure Value (Female minus Male difference) to diet low in seafood Omega-3, 1990 vs. 2019. Developed based on the GBD 2019 study[103].

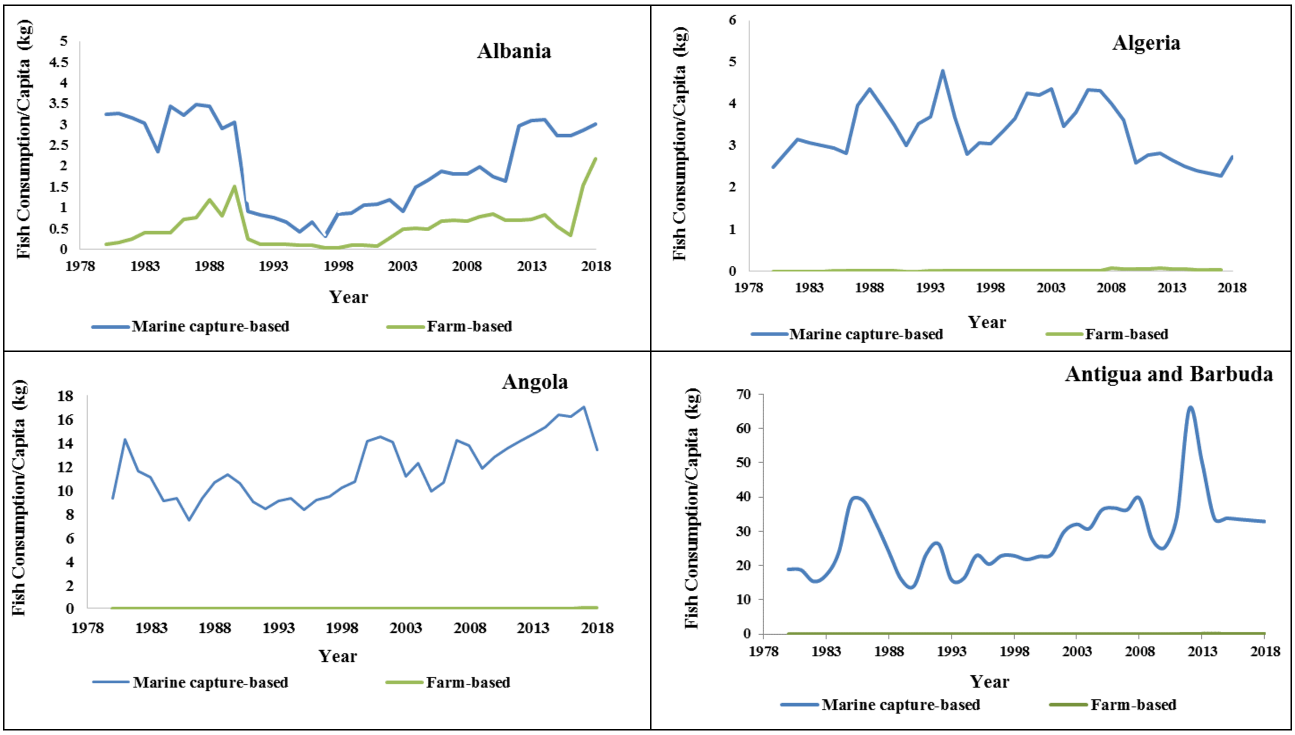

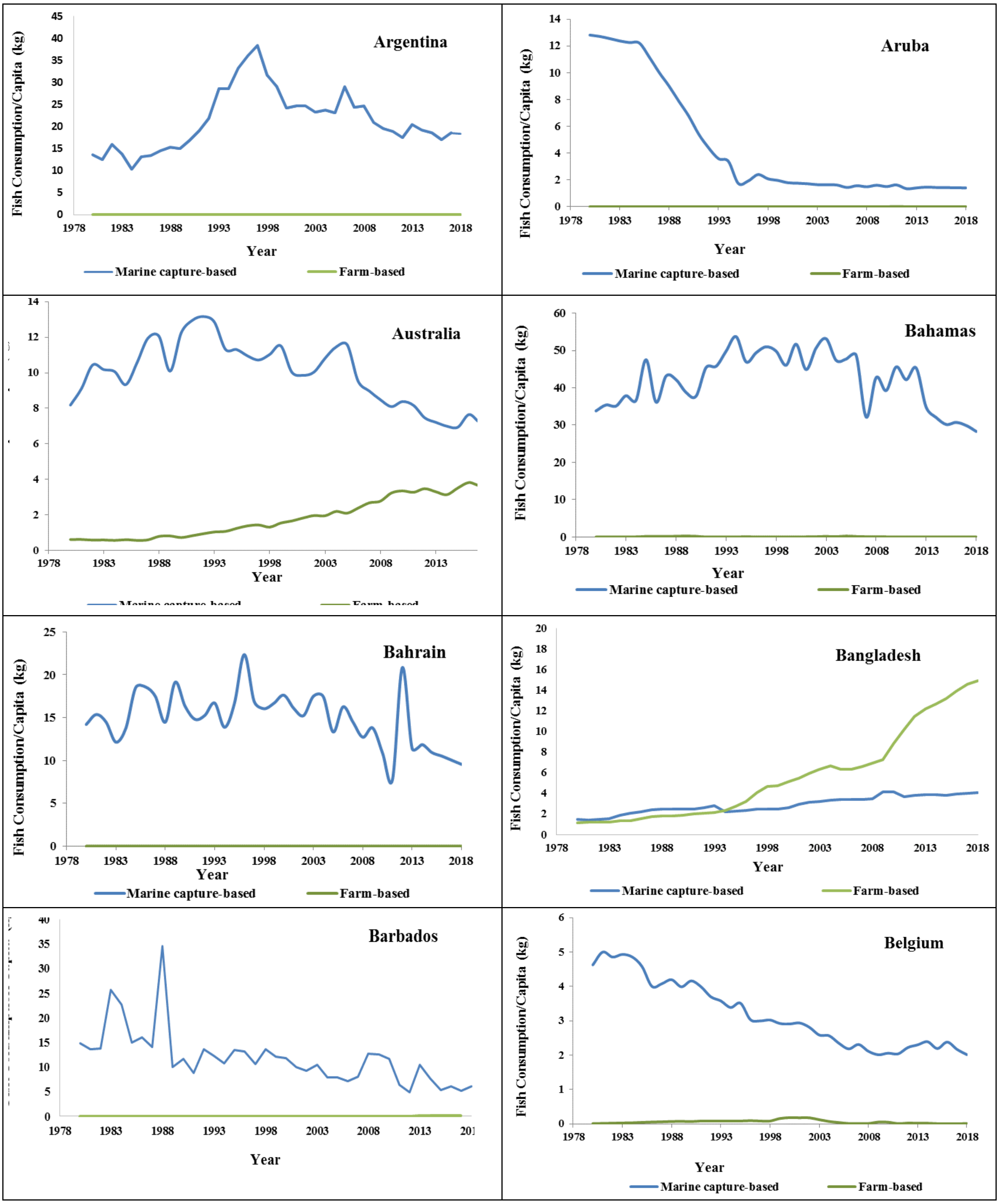
Argentina
Aruba
Australia
Bahamas
Bahrain
Bangladesh
Barbados
Belgium
Fish Consumption/Capita (kg)
Year
Marine capture-based
Farm-based

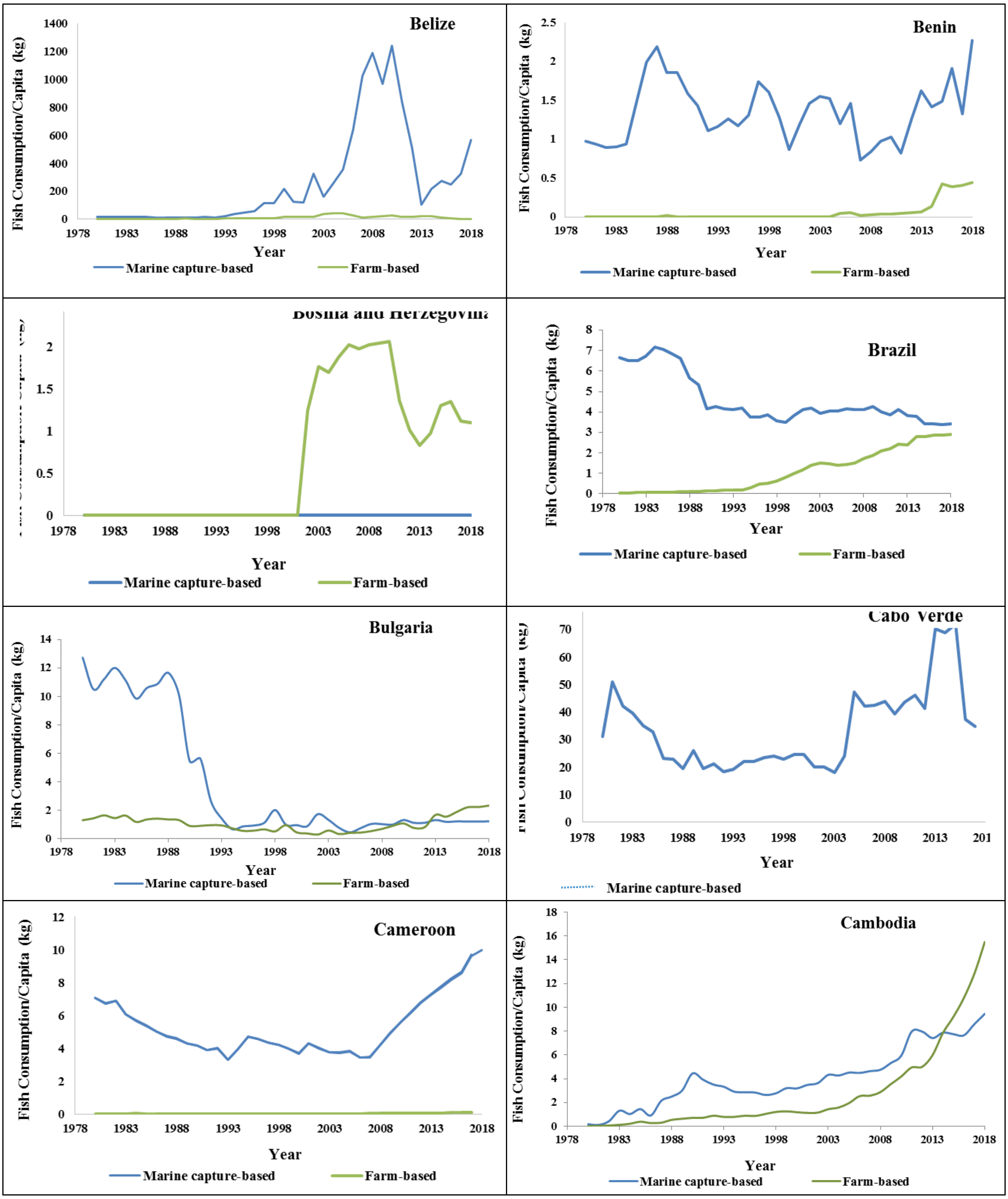
Belize
Fish Consumption/Capita (kg)
1400
1200
1000
800
600
400
200
0
1978 1983 1988 1993 1998 2003 2008 2013 2018
Year
Marine capture-based
Farm-based
Benin
Fish Consumption/Capita (kg)
2.5
2
1.5
1
0.5
0
1978 1983 1988 1993 1998 2003 2008 2013 2018
Year
Marine capture-based
Farm-based
2
1.5
1
0.5
0
1978 1983 1988 1993 1998 2003 2008 2013 2018
Year
Marine capture-based
Farm-based
Brazil
Fish Consumption/Capita (kg)
8
7
6
5
4
3
2
1
0
1978 1983 1988 1993 1998 2003 2008 2013 2018
Year
Marine capture-based
Farm-based
Bulgaria
Fish Consumption/Capita (kg)
14
12
10
8
6
4
2
0
1978 1983 1988 1993 1998 2003 2008 2013 2018
Year
Marine capture-based
Farm-based
70
60
50
40
30
20
10
0
1978 1983 1988 1993 1998 2003 2008 2013
Year
Marine capture-based
Cameroon
Fish Consumption/Capita (kg)
12
10
8
6
4
2
0
1978 1983 1988 1993 1998 2003 2008 2013 2018
Year
Marine capture-based
Farm-based
Cambodia
Fish Consumption/Capita (kg)
18
16
14
12
10
8
6
4
2
0
1978 1983 1988 1993 1998 2003 2008 2013 2018
Year
Marine capture-based
Farm-based

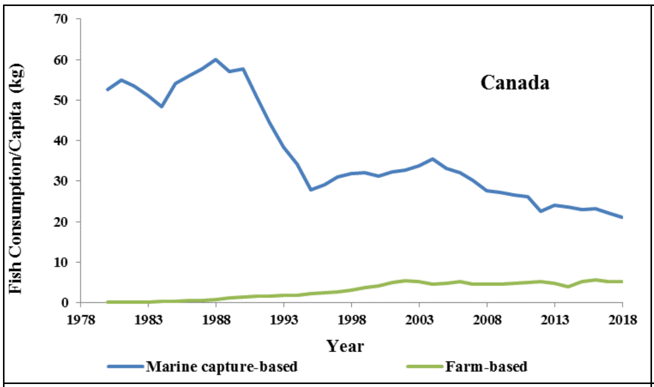
Canada
Fish Consumption/Capita (kg)
Year
Marine capture-based
Farm-based

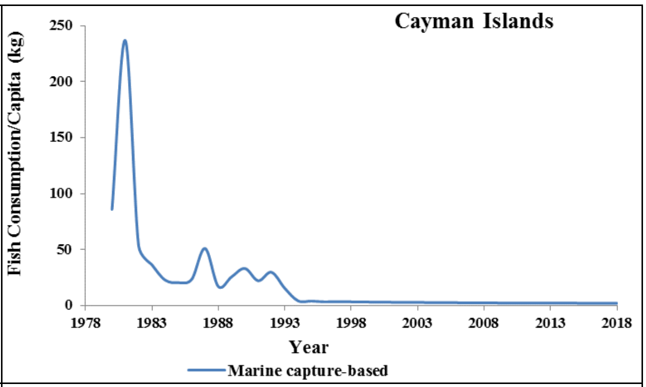
Cayman Islands
Fish Consumption/Capita (kg)
Year
Marine capture-based

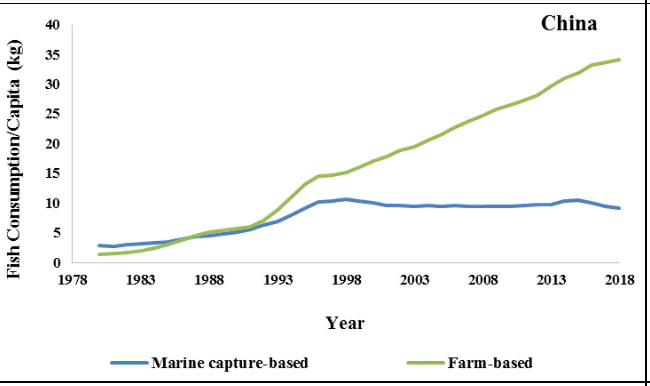
China
Fish Consumption/Capita (kg)
Year
Marine capture-based
Farm-based

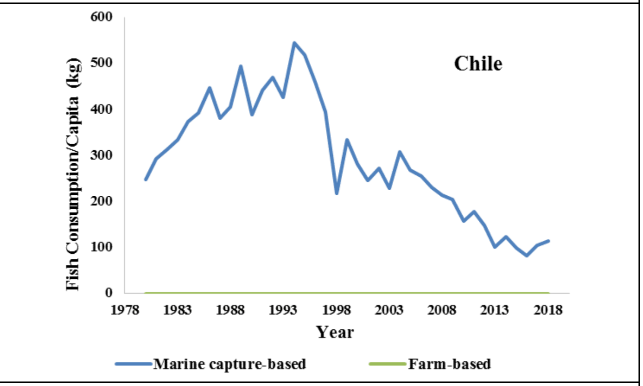
Chile
Fish Consumption/Capita (kg)
Year
Marine capture-based
Farm-based

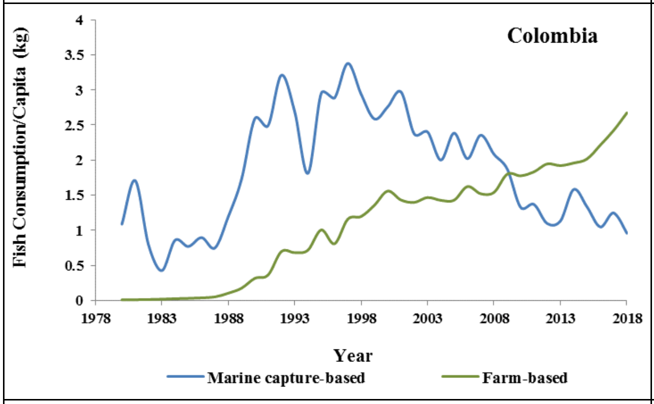
Colombia
Fish Consumption/Capita (kg)
Year
Marine capture-based
Farm-based

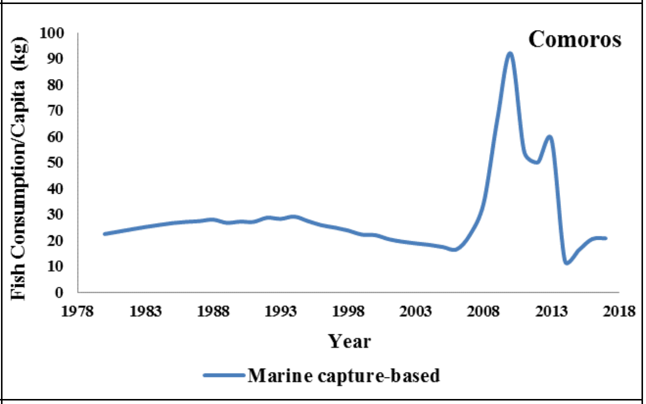
Comoros
Fish Consumption/Capita (kg)
Year
Marine capture-based

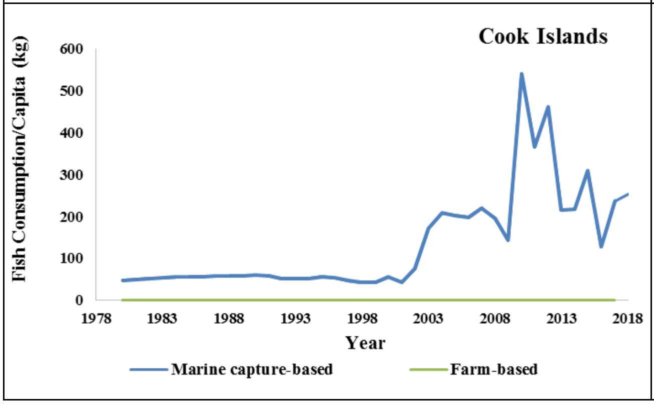
Cook Islands
Fish Consumption/Capita (kg)
Year
Marine capture-based
Farm-based

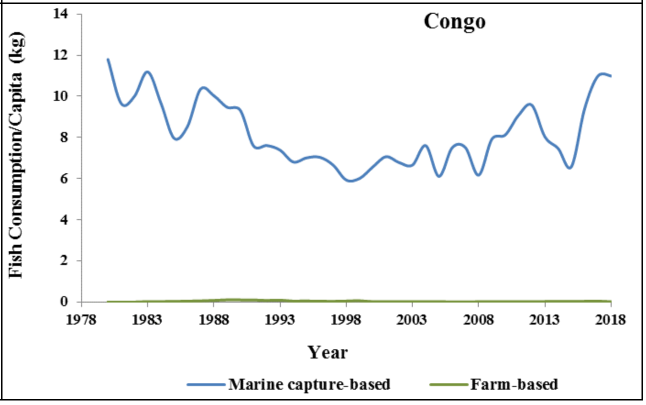
Congo
Fish Consumption/Capita (kg)
Year
Marine capture-based
Farm-based

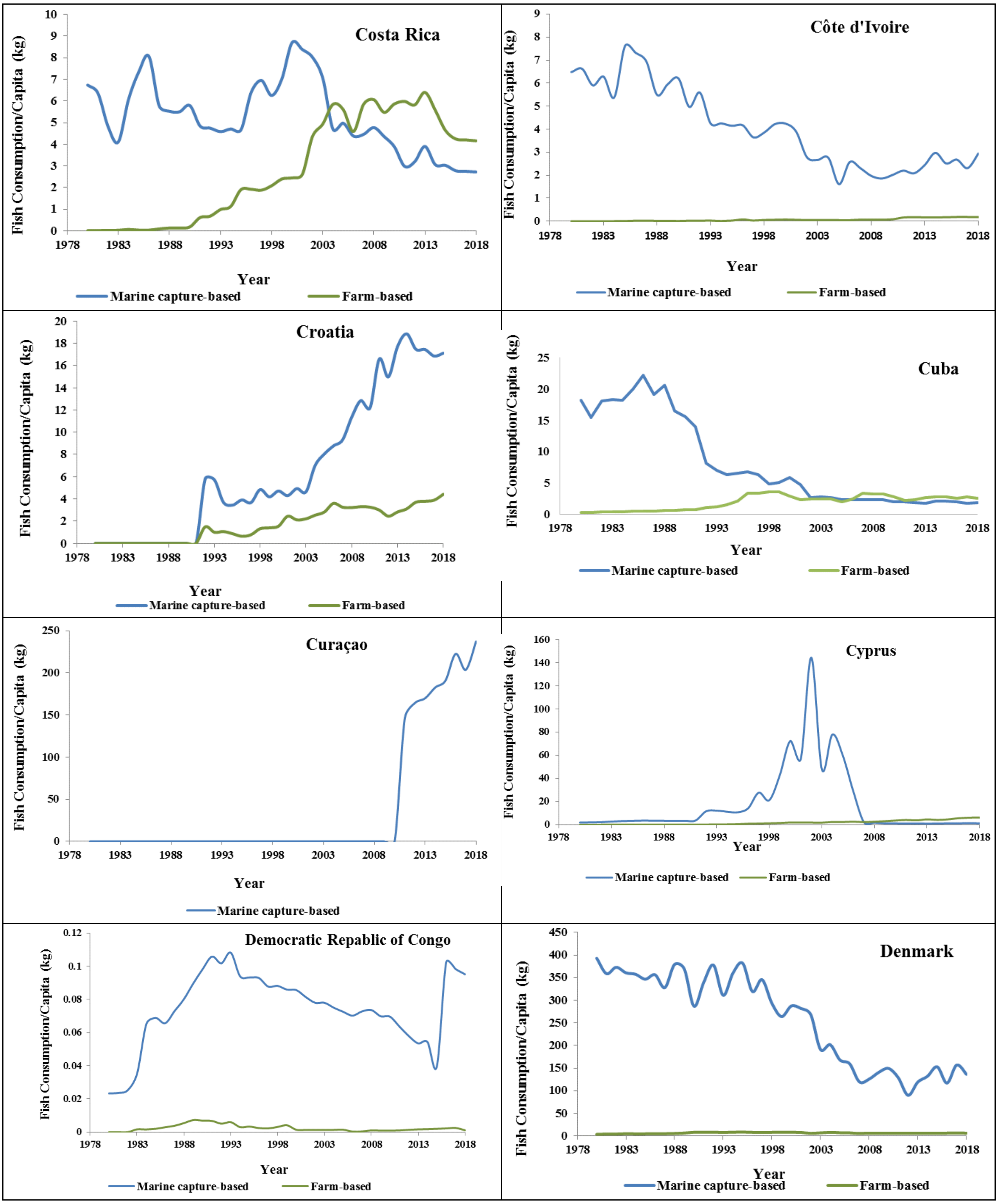

Costa Rica
Fish Consumption/Capita (kg)
Year
Marine capture-based
Farm-based
Côte d'Ivoire
Fish Consumption/Capita (kg)
Year
Marine capture-based
Farm-based
Croatia
Fish Consumption/Capita (kg)
Year
Marine capture-based
Farm-based
Cuba
Fish Consumption/Capita (kg)
Year
Marine capture-based
Farm-based
Curaçao
Fish Consumption/Capita (kg)
Year
Marine capture-based
Cyprus
Fish Consumption/Capita (kg)
Year
Marine capture-based
Farm-based
Democratic Repablic of Congo
Fish Consumption/Capita (kg)
Year
Marine capture-based
Farm-based
Denmark
Fish Consumption/Capita (kg)
Year
Marine capture-based
Farm-based

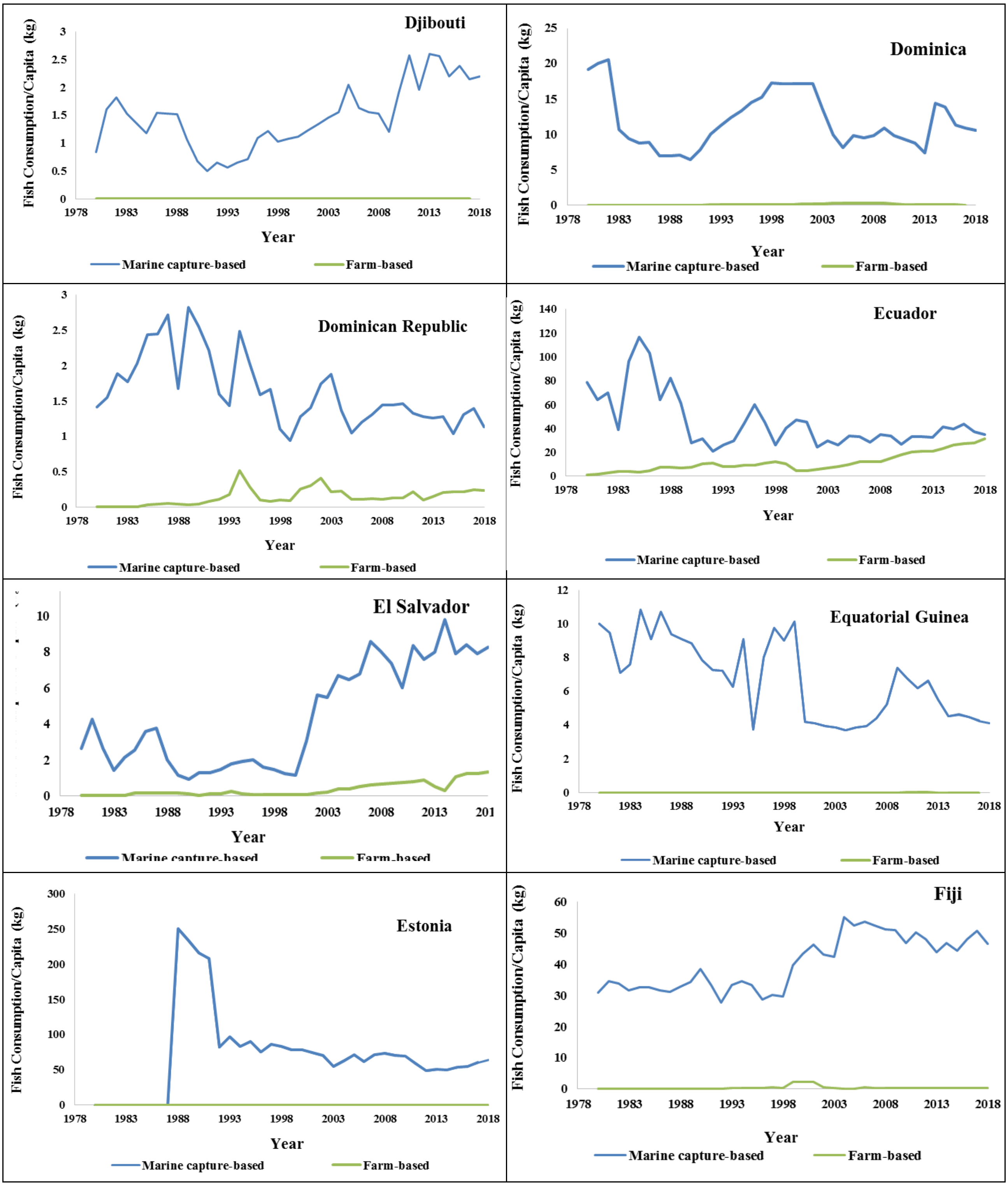
Djibouti
Dominica
Dominican Republic
Ecuador
El Salvador
Equatorial Guinea
Estonia
Fiji
Fish Consumption/Capita (kg)
Year
Marine capture-based
Farm-based

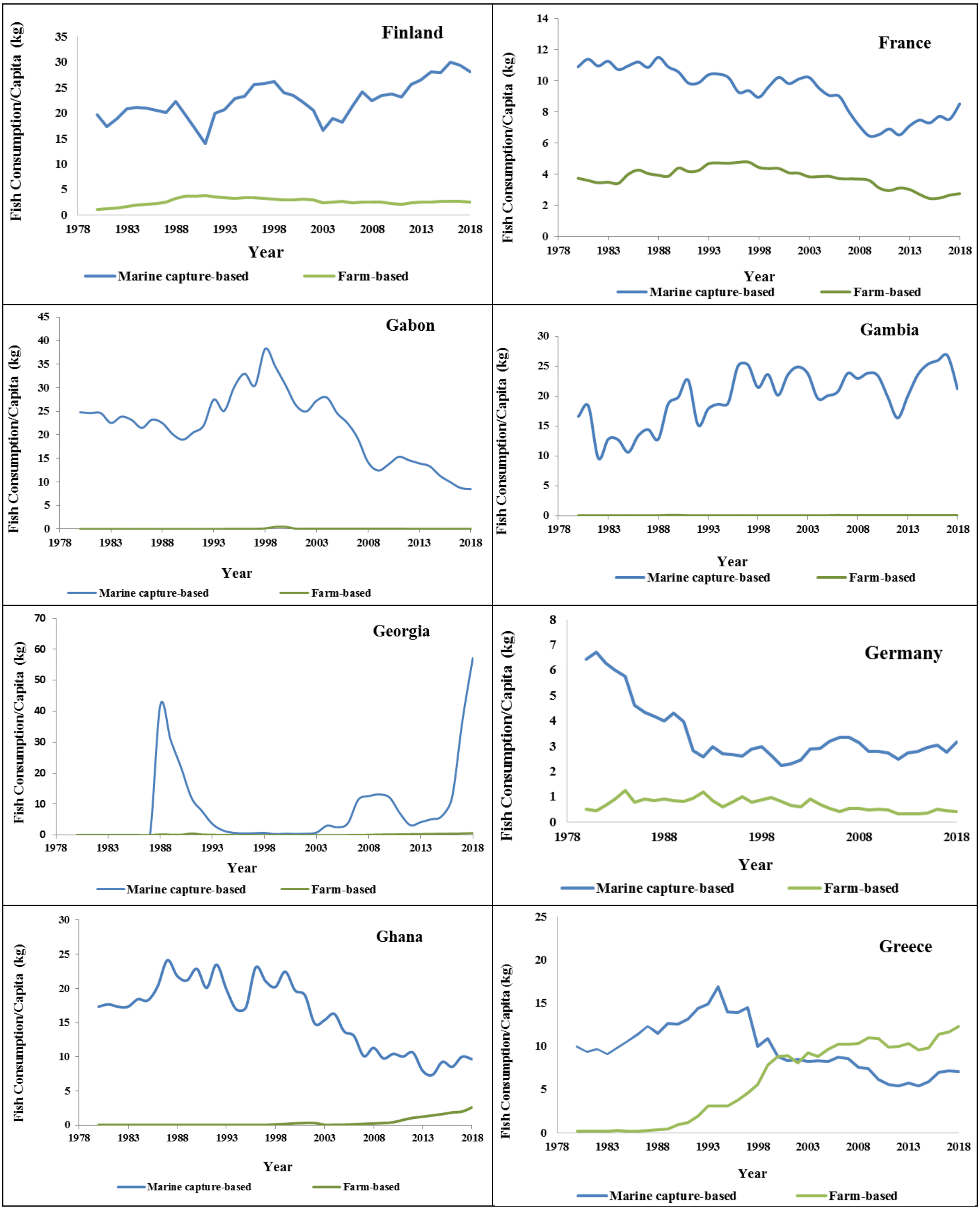
Finland
Fish Consumption/Capita (kg)
Year
Marine capture-based
Farm-based
France
Fish Consumption/Capita (kg)
Year
Marine capture-based
Farm-based
Gabon
Fish Consumption/Capita (kg)
Year
Marine capture-based
Farm-based
Gambia
Fish Consumption/Capita (kg)
Year
Marine capture-based
Farm-based
Georgia
Fish Consumption/Capita (kg)
Year
Marine capture-based
Farm-based
Germany
Fish Consumption/Capita (kg)
Year
Marine capture-based
Farm-based
Ghana
Fish Consumption/Capita (kg)
Year
Marine capture-based
Farm-based
Greece
Fish Consumption/Capita (kg)
Year
Marine capture-based
Farm-based

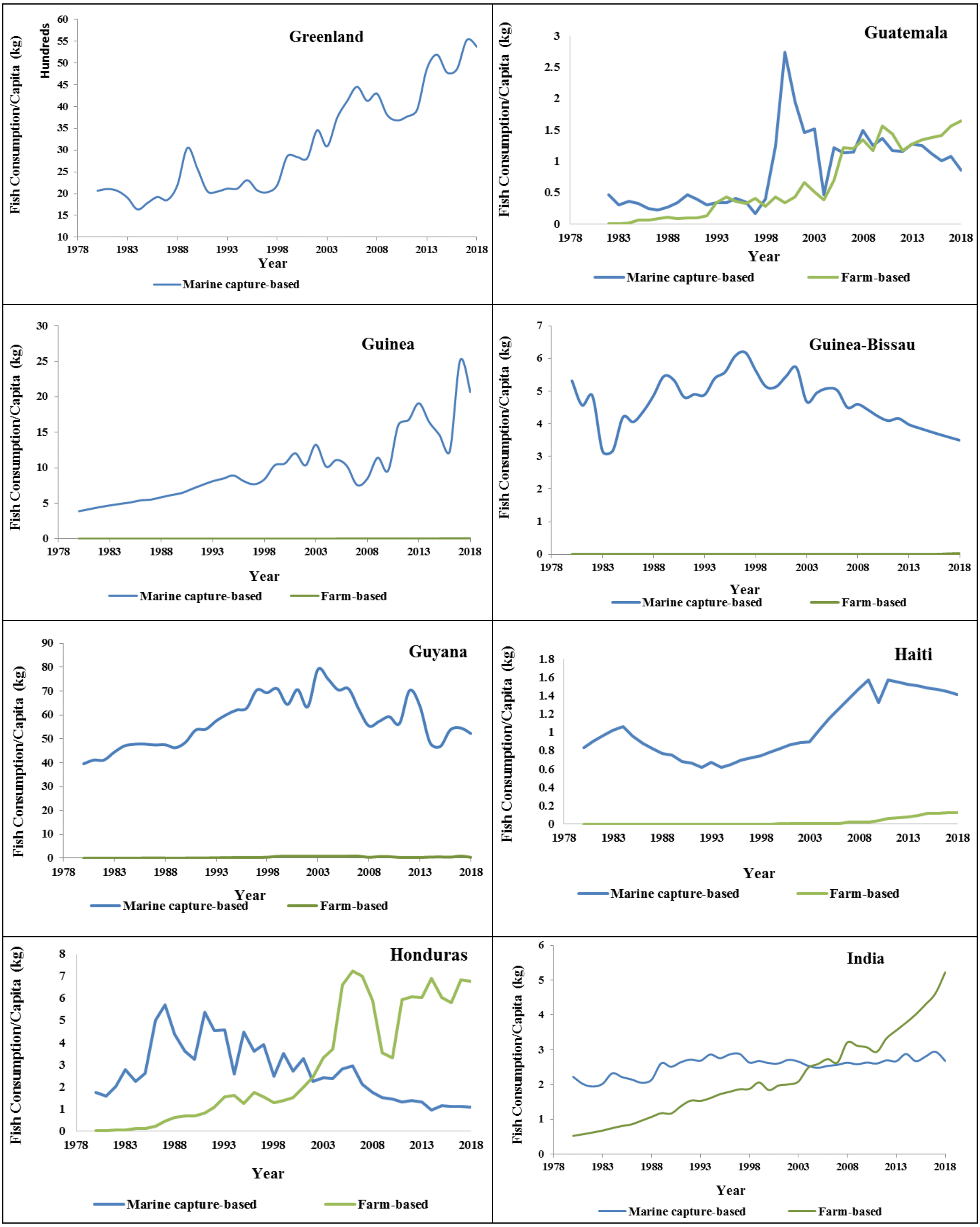
Greenland
Fish Consumption/Capita (kg)
Hundreds
Year
Marine capture-based
Guatemala
Fish Consumption/Capita (kg)
Year
Marine capture-based
Farm-based
Guinea
Fish Consumption/Capita (kg)
Year
Marine capture-based
Farm-based
Guinea-Bissau
Fish Consumption/Capita (kg)
Year
Marine capture-based
Farm-based
Guyana
Fish Consumption/Capita (kg)
Year
Marine capture-based
Farm-based
Haiti
Fish Consumption/Capita (kg)
Year
Marine capture-based
Farm-based
Honduras
Fish Consumption/Capita (kg)
Year
Marine capture-based
Farm-based
India
Fish Consumption/Capita (kg)
Year
Marine capture-based
Farm-based

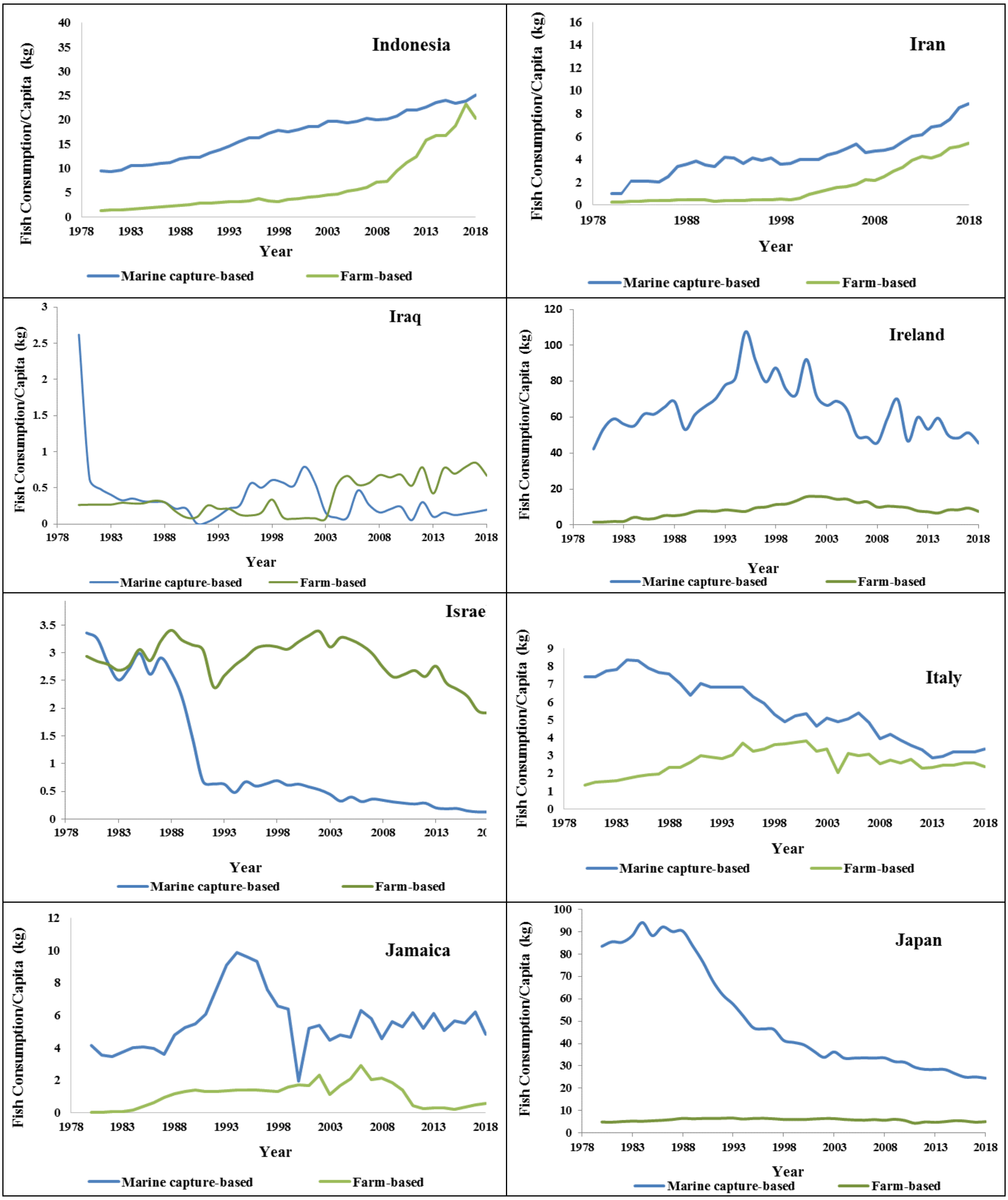
Indonesia
Fish Consumption/Capita (kg)
40
35
30
25
20
15
10
5
0
1978
1983
1988
1993
1998
2003
2008
2013
2018
Year
Marine capture-based
Farm-based
Iran
Fish Consumption/Capita (kg)
16
14
12
10
8
6
4
2
0
1978
1988
1998
2008
2018
Year
Marine capture-based
Farm-based
Iraq
Fish Consumption/Capita (kg)
3
2.5
2
1.5
1
0.5
0
1978
1983
1988
1993
1998
2003
2008
2013
2018
Year
Marine capture-based
Farm-based
Ireland
Fish Consumption/Capita (kg)
120
100
80
60
40
20
0
1978
1983
1988
1993
1998
2003
2008
2013
2018
Year
Marine capture-based
Farm-based
Israe
3.5
3
2.5
2
1.5
1
0.5
0
1978
1983
1988
1993
1998
2003
2008
2013
20
Year
Marine capture-based
Farm-based
Italy
Fish Consumption/Capita (kg)
9
8
7
6
5
4
3
2
1
0
1978
1983
1988
1993
1998
2003
2008
2013
2018
Year
Marine capture-based
Farm-based
Jamaica
Fish Consumption/Capita (kg)
12
10
8
6
4
2
0
1978
1983
1988
1993
1998
2003
2008
2013
2018
Year
Marine capture-based
Farm-based
Japan
Fish Consumption/Capita (kg)
100
90
80
70
60
50
40
30
20
10
0
1978
1983
1988
1993
1998
2003
2008
2013
2018
Year
Marine capture-based
Farm-based

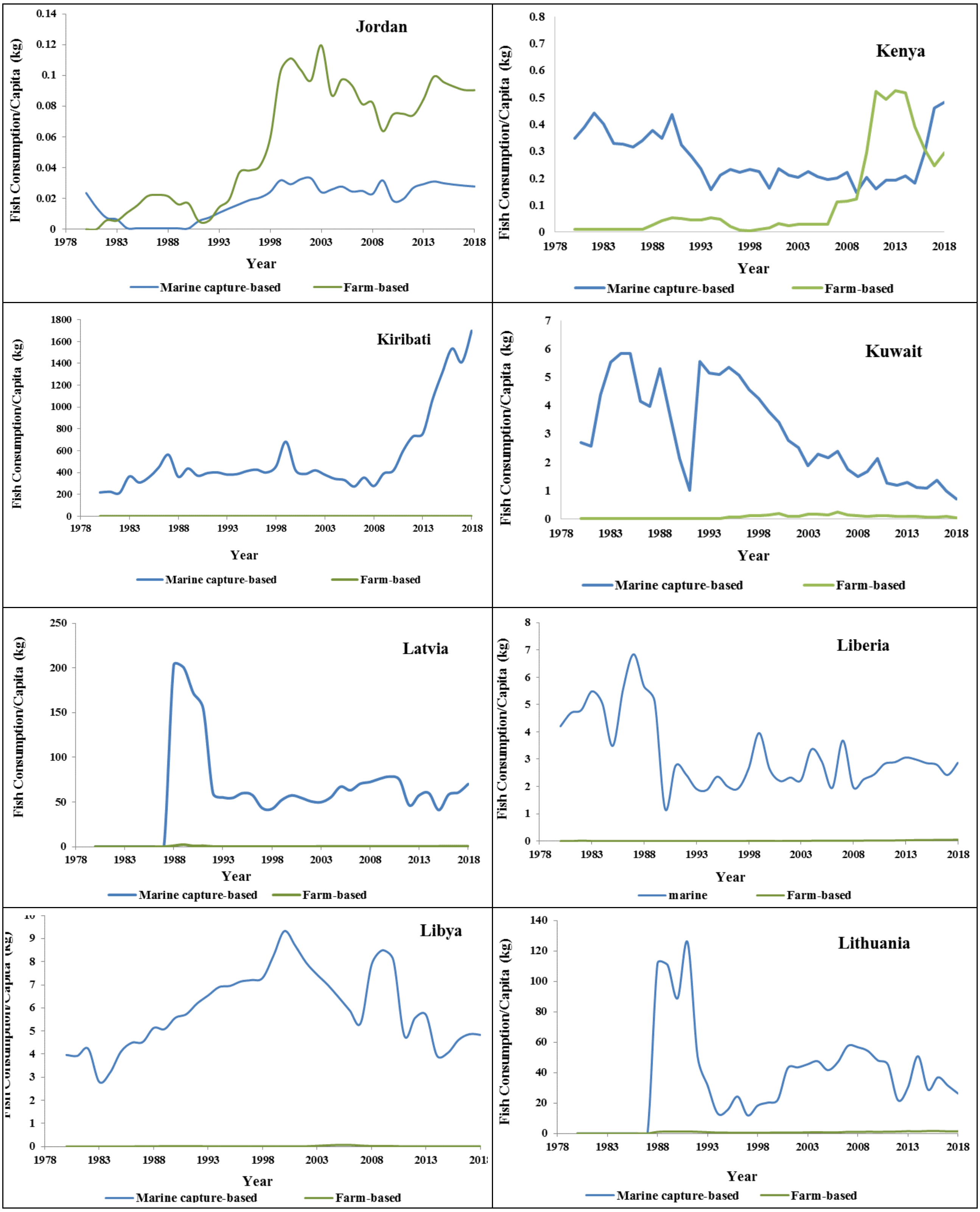
Jordan
Kenya
Kiribati
Kuwait
Latvia
Liberia
Libya
Lithuania
Fish Consumption/Capita (kg)
Year
Marine capture-based
Farm-based
marine

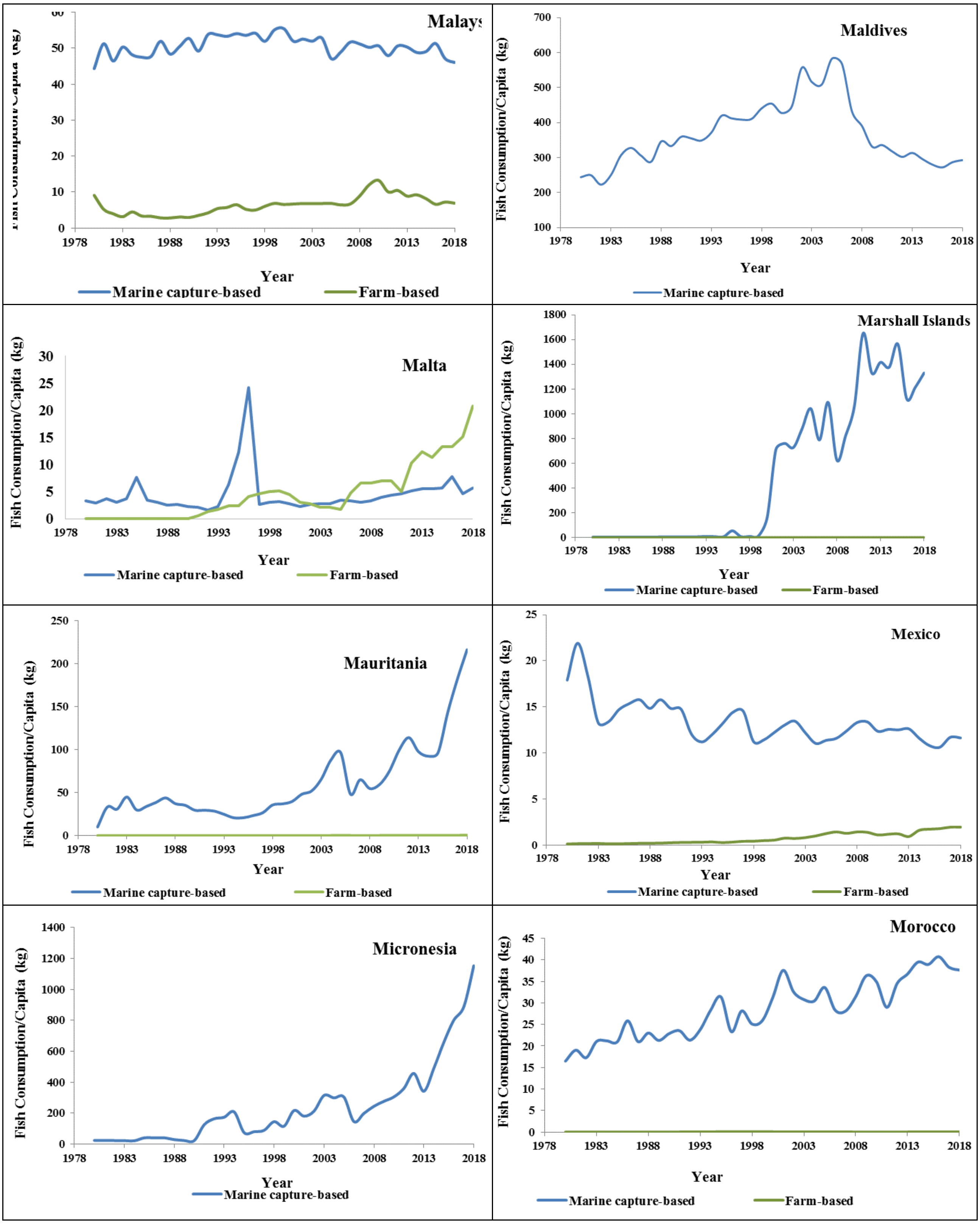
Malays
Fish Consumption/Capita (kg)
Year
Marine capture-based
Farm-based
Maldives
Fish Consumption/Capita (kg)
Year
Marine capture-based
Malta
Fish Consumption/Capita (kg)
Year
Marine capture-based
Farm-based
Marshall Islands
Fish Consumption/Capita (kg)
Year
Marine capture-based
Farm-based
Mauritania
Fish Consumption/Capita (kg)
Year
Marine capture-based
Farm-based
Mexico
Fish Consumption/Capita (kg)
Year
Marine capture-based
Farm-based
Micronesia
Fish Consumption/Capita (kg)
Year
Marine capture-based
Morocco
Fish Consumption/Capita (kg)
Year
Marine capture-based
Farm-based

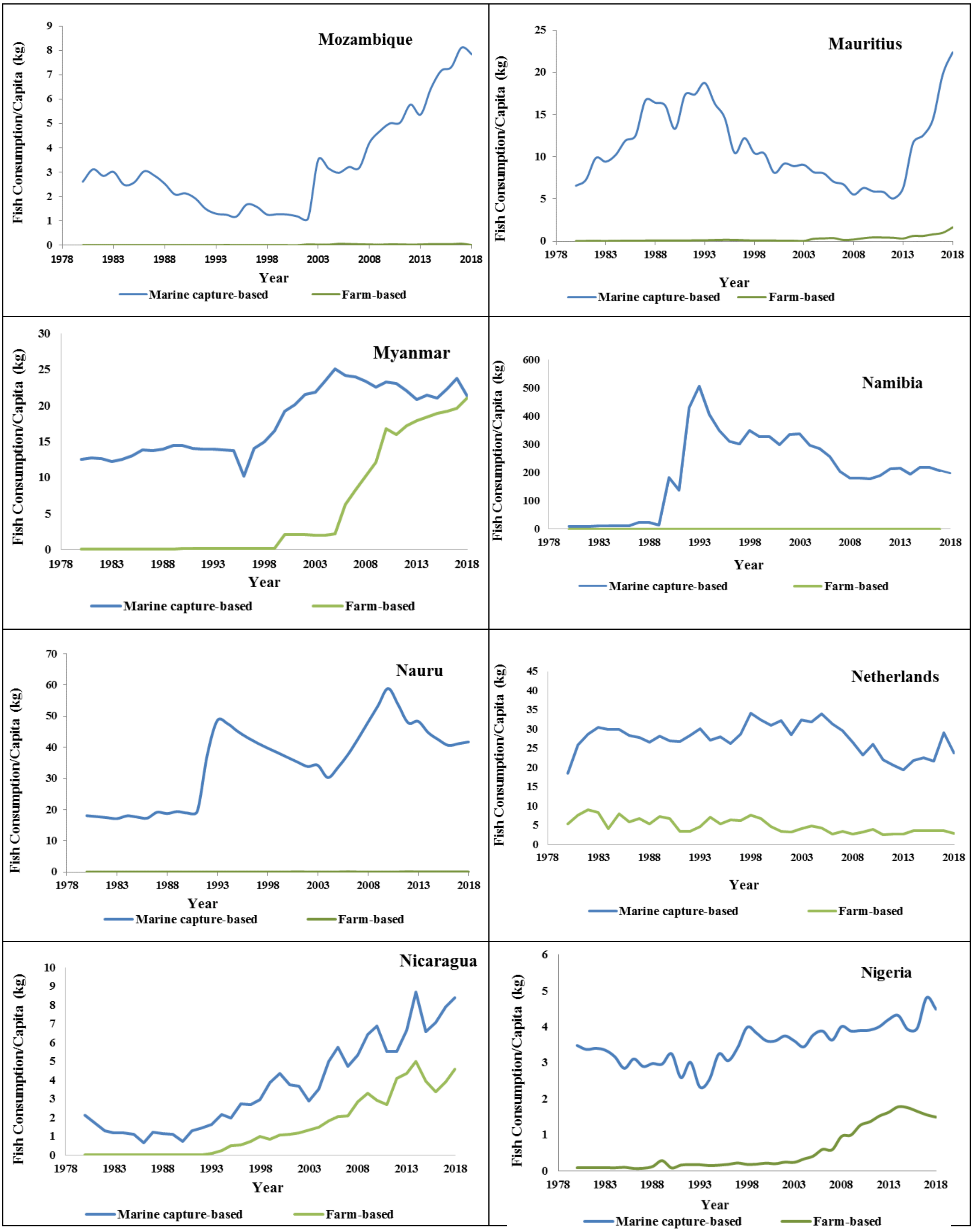
Mozambique
Mauritius
Myanmar
Namibia
Nauru
Netherlands
Nicaragua
Nigeria
Fish Consumption/Capita (kg)
Year
Marine capture-based
Farm-based
1978
1983
1988
1993
1998
2003
2008
2013
2018

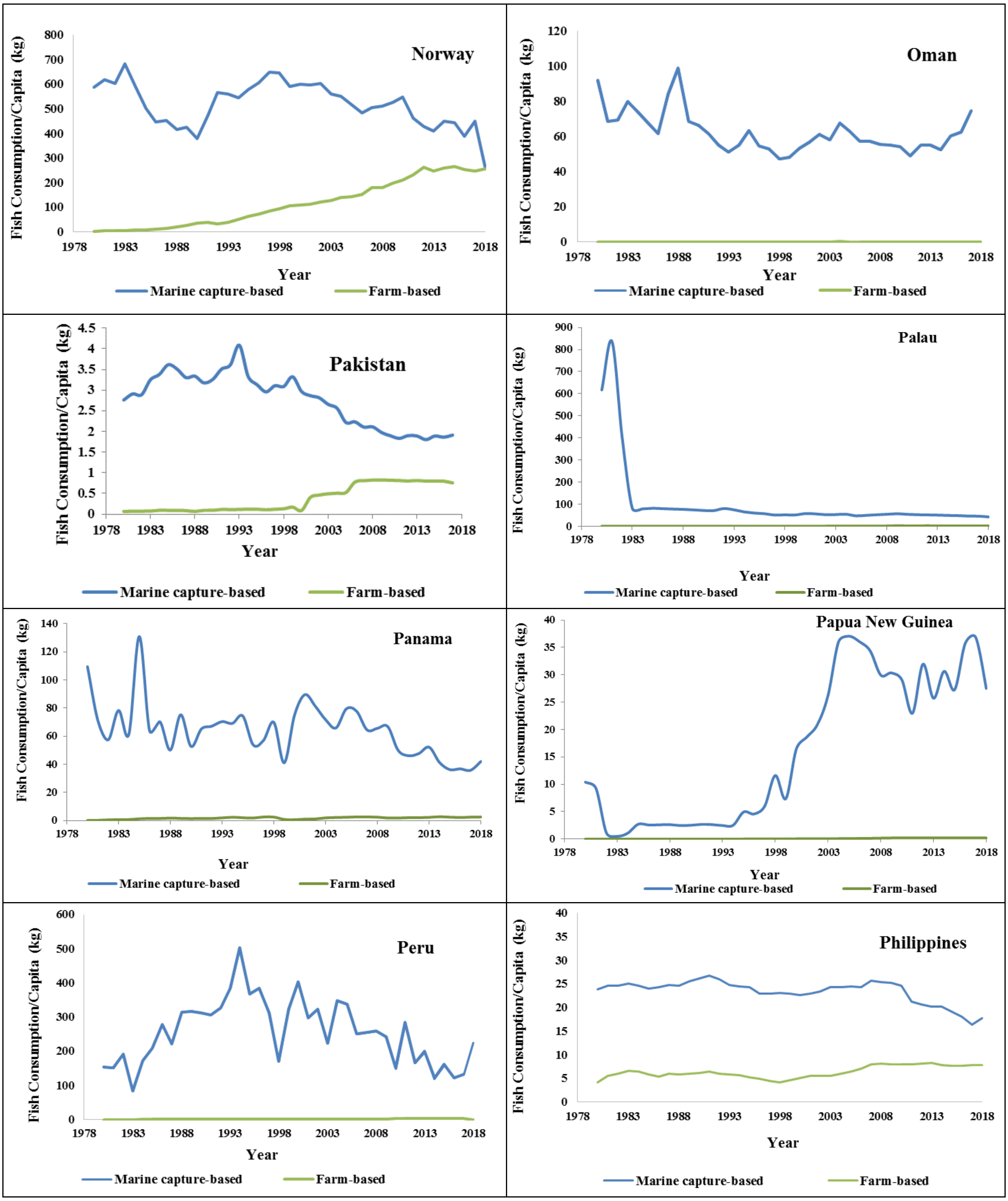
Norway
Fish Consumption/Capita (kg)
Year
Marine capture-based
Farm-based
Oman
Fish Consumption/Capita (kg)
Year
Marine capture-based
Farm-based
Pakistan
Fish Consumption/Capita (kg)
Year
Marine capture-based
Farm-based
Palau
Fish Consumption/Capita (kg)
Year
Marine capture-based
Farm-based
Panama
Fish Consumption/Capita (kg)
Year
Marine capture-based
Farm-based
Papua New Guinea
Fish Consumption/Capita (kg)
Year
Marine capture-based
Farm-based
Peru
Fish Consumption/Capita (kg)
Year
Marine capture-based
Farm-based
Philippines
Fish Consumption/Capita (kg)
Year
Marine capture-based
Farm-based

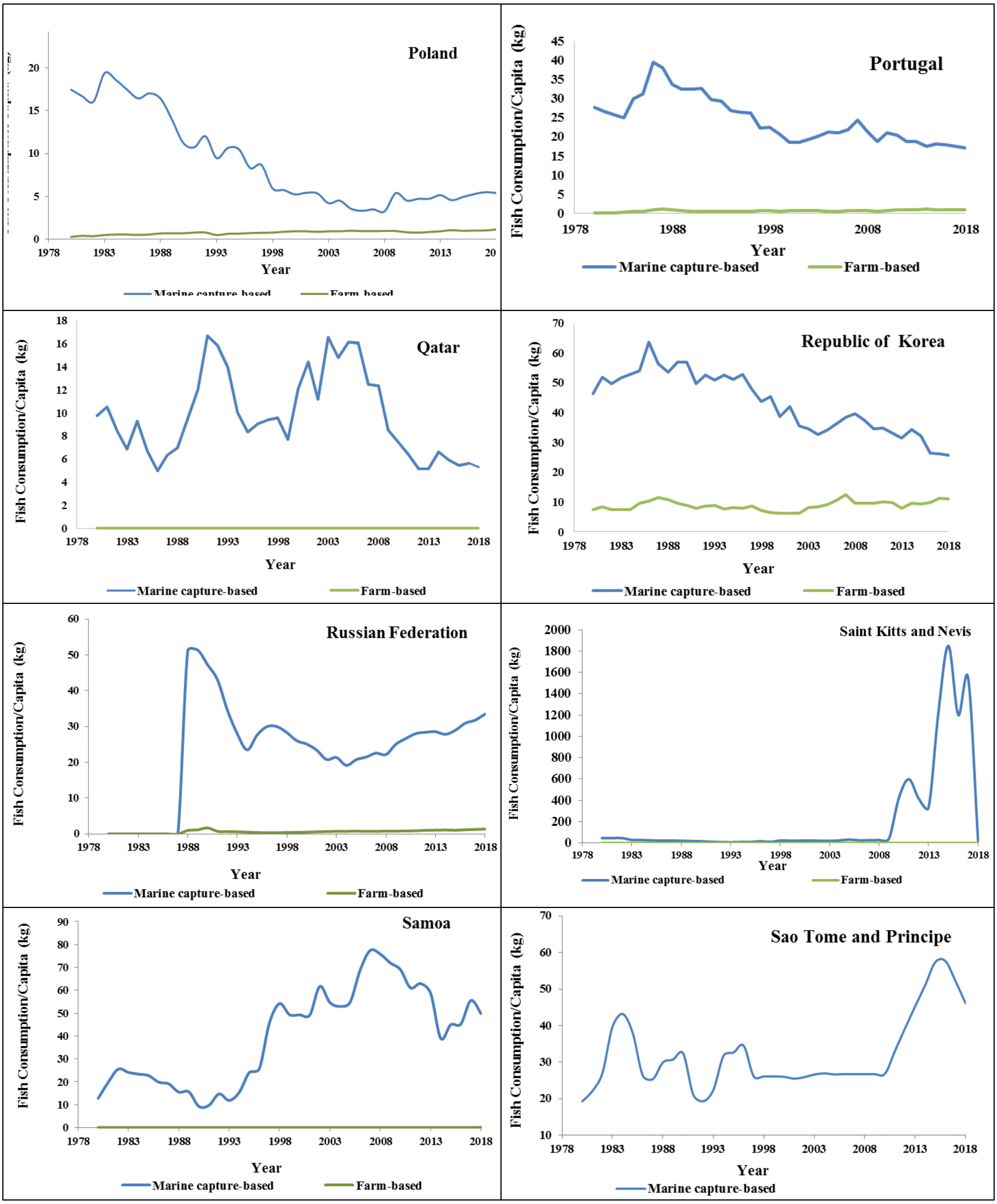

Poland
Year
Marine capture-based
Farm-based
Portugal
Fish Consumption/Capita (kg)
Year
Marine capture-based
Farm-based
Qatar
Fish Consumption/Capita (kg)
Year
Marine capture-based
Farm-based
Republic of Korea
Fish Consumption/Capita (kg)
Year
Marine capture-based
Farm-based
Russian Federation
Fish Consumption/Capita (kg)
Year
Marine capture-based
Farm-based
Saint Kitts and Nevis
Fish Consumption/Capita (kg)
Year
Marine capture-based
Farm-based
Samoa
Fish Consumption/Capita (kg)
Year
Marine capture-based
Farm-based
Sao Tome and Principe
Fish Consumption/Capita (kg)
Year
Marine capture-based

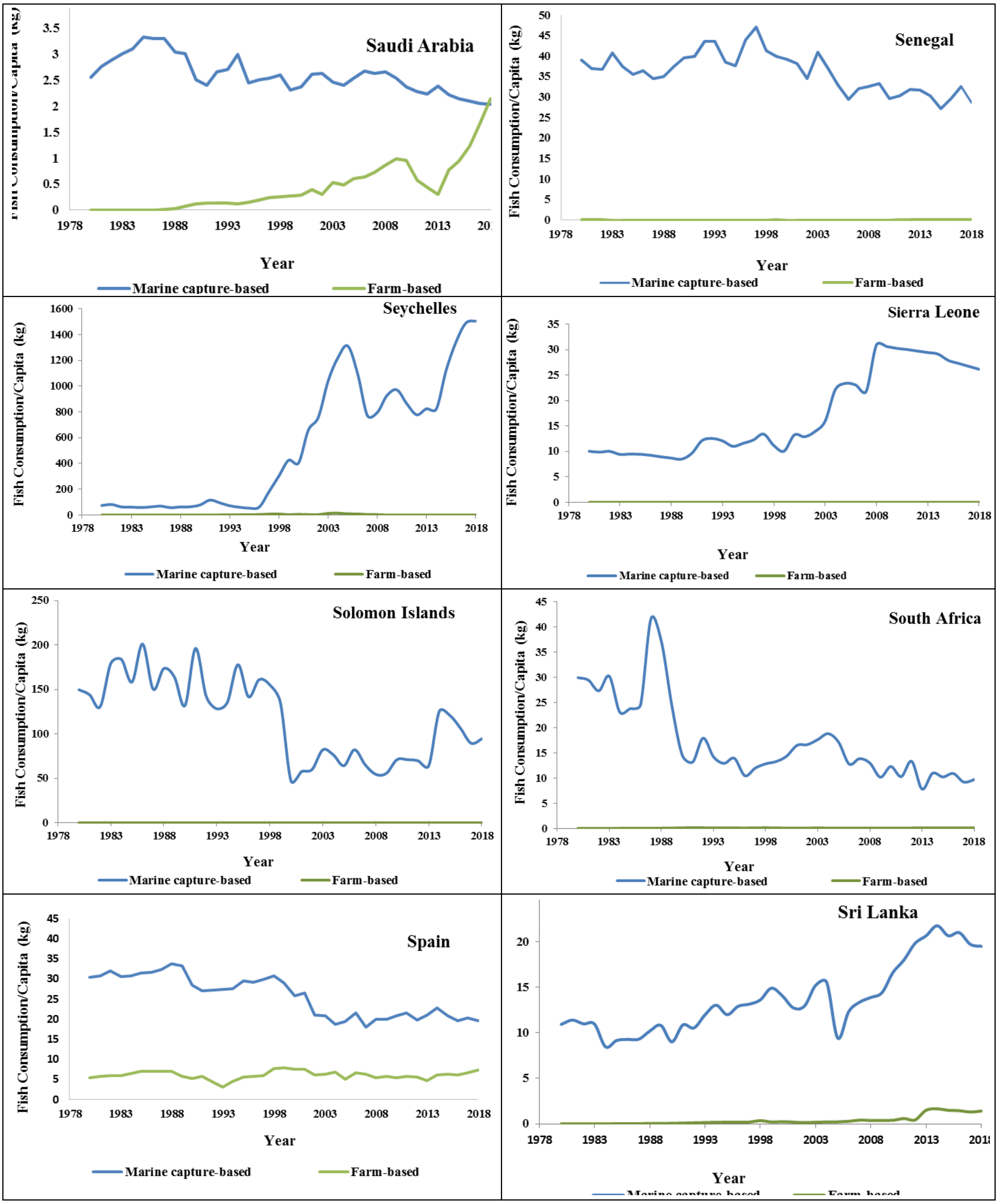
Saudi Arabia
Senegal
Seychelles
Sierra Leone
Solomon Islands
South Africa
Spain
Sri Lanka
Fish Consumption/Capita (kg)
Year
Marine capture-based
Farm-based

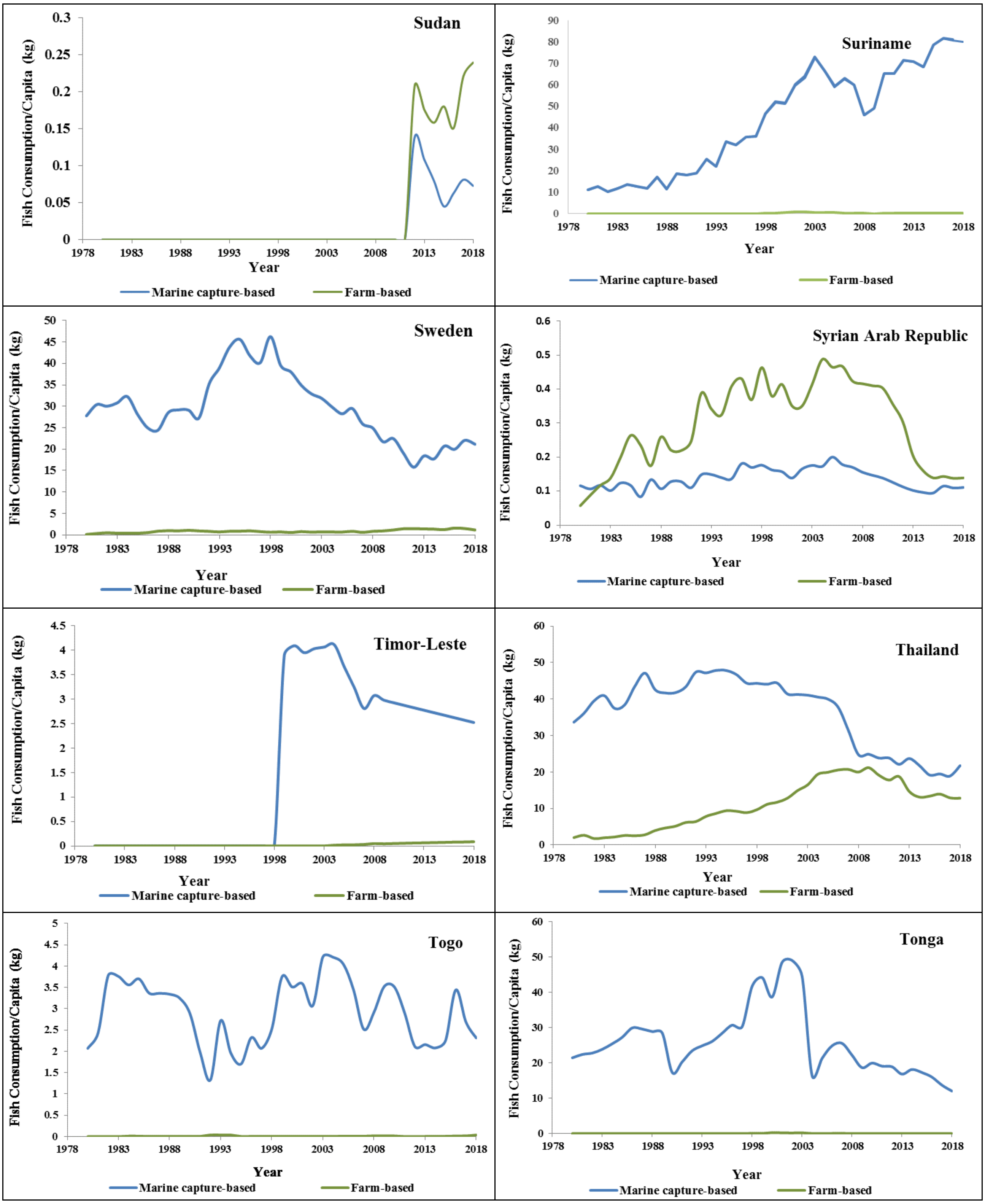
Sudan
Suriname
Sweden
Syrian Arab Republic
Timor-Leste
Thailand
Togo
Tonga
Fish Consumption/Capita (kg)
Year
Marine capture-based
Farm-based

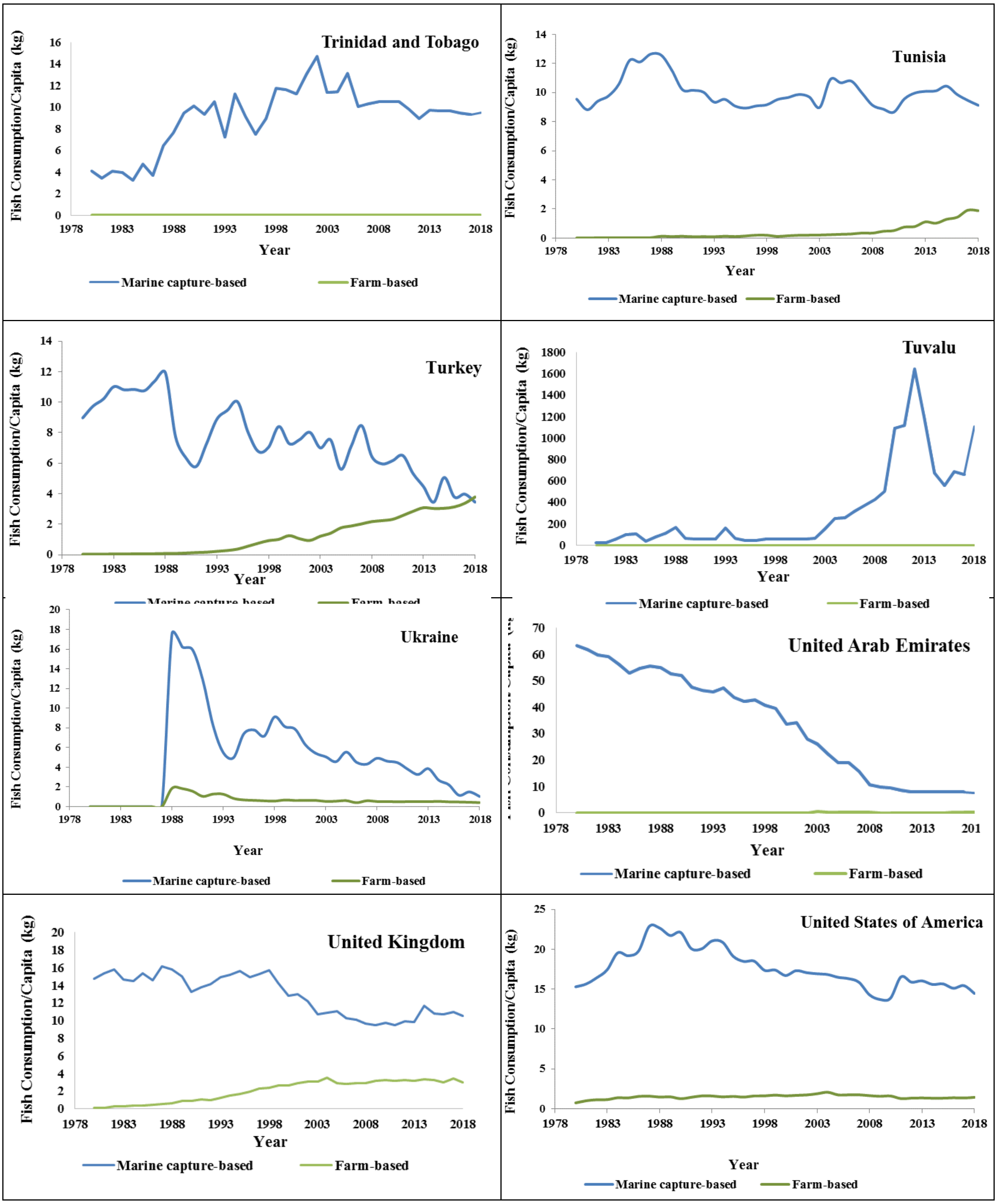
Trinidad and Tobago
Tunisia
Turkey
Tuvalu
Ukraine
United Arab Emirates
United Kingdom
United States of America
Fish Consumption/Capita (kg)
Year
Marine capture-based
Farm-based

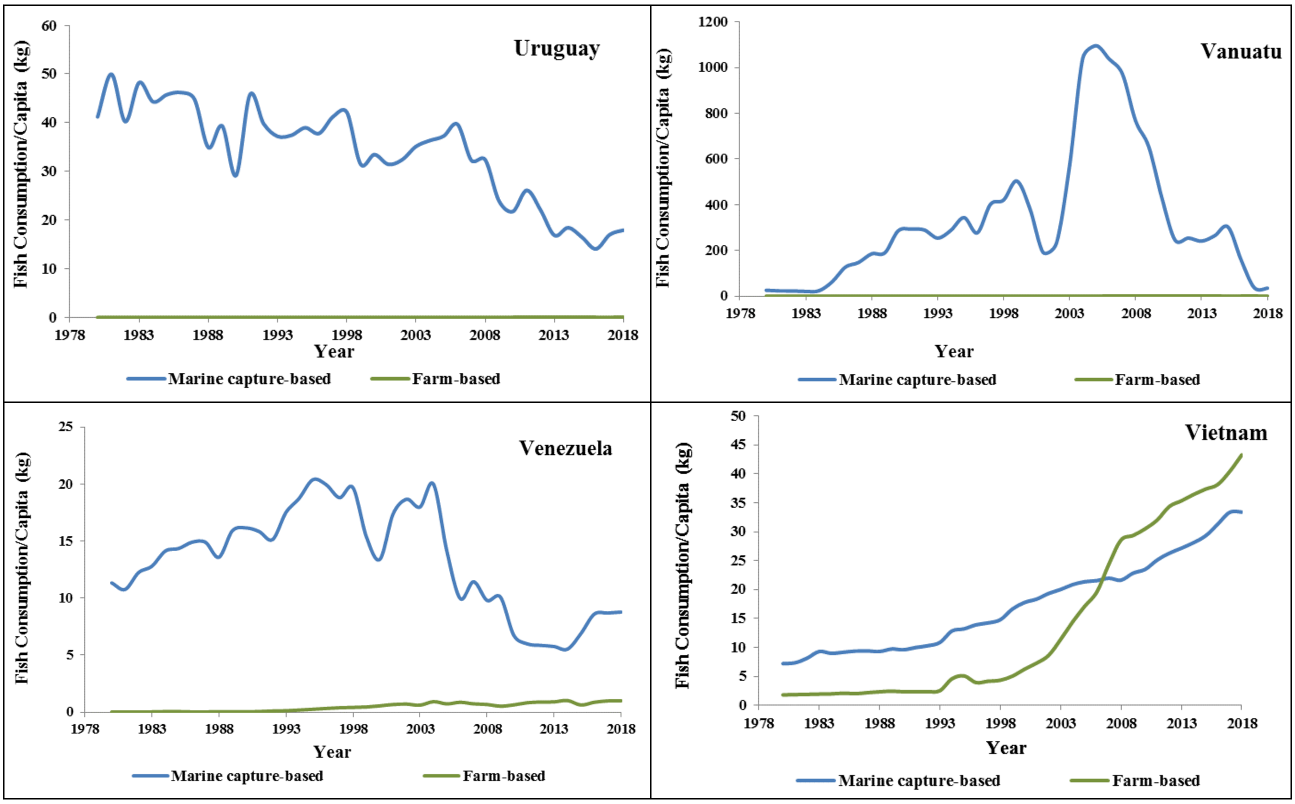

Figure 40: Trends of capture-based and farmed-based per capita fish consumption in the 136 countries investigated from 1980 through 2018

Indicator 1.5: Migration, Displacement and Rising Sea Levels

Population exposure to global mean sea level rise

Methods

The methodology for this indicator remains similar to that described in the 2020 report of the Lancet Countdown.[1] By using a bathtub model, this indicator overlays future Global Mean Sea Level Rise (GMSLR, of 1m and 5m) with coastal elevation value grid-cells to delineate areas of potential inundation and current global population distribution grid-cells to delineate populations living in areas that will be exposed to absolute global mean sea level rise.

In the first step, the Coastal Digital Elevation Model (CoastalDEM) dataset was used to categorise inundated grid-cells under two scenarios of global mean sea level rise (GMSLR): i.e., 1m and 5m of GMSLR. In the second step, the gridded population dataset (LandScan) was overlaid on the grid-cells identified in step one to estimate population exposure values. These grid-cells are then matched with country boundaries using Global Administrative Areas (GADM) 3.6 Data Set. Grid-cell level data were then aggregated to country level (population numbers exposed to GMSLR; proportion of population exposed to GMSLR). In the third step, the population exposed to 1m and 5m of GMSLR data were overlaid on the sub-national HDI to identify the socioeconomic status of populations exposed to GMSLR. The inclusion of HDI into the indicator methodology, new to 2021, reflects the current literature on the topic, specifically on socioeconomic contexts shaping adaptive options and responses to climate impacts, including sea level rise.[104,105]

Data

1. GMSLR data were taken from estimated global mean increases in sea-levels.[106]
2. Elevation data were taken from Coastal Digital Elevation Model (CoastalDEM).[107]
3. Population distribution was taken from LandScan 2019.[108]
4. Global Administrative Areas (GADM) version 3.6.[109]
5. The Sub-national Human Development Index database.[110]

Caveats

Between 1902 and 2015, the global mean sea level increased by 0.12–0.21m.[111] Relative to 1986–2005, additional GMSLR of 0.43-0.84m is projected by 2100 (0.29-1.10m, likely range),[111] although it depends particularly on the rate of Greenland and Antarctic ice sheet melting.[112]

Estimates of population exposure to GMSLR vary according to the input datasets, timeframes and geographic scales, the parameters that are set for about emissions and socioeconomic scenarios, and methods of analysis.[113] Results should be regarded within the context of the methods and datasets used. As such underlying errors and uncertainties in the input datasets (GMSLR, elevation, and population) are limitations of this analysis. The datasets employed for this indicator are global, reputable and widely used in analyses of exposure to SLR. CoastalDEM (3-arc second; 90m) is a new global coastal digital elevation model that has been adjusted to reduce SRTM error.[114] LandScan disaggregates census data within administrative boundaries based on weightings derived from land cover data, proximity to roads, slope, and populated areas.[115] As for the HDI, the database contains the national and subnational values of the three-dimension indices on the basis of which the subnational HDI is constructed – education, health, and standard of living. Values are provided for 1,625 regions within 161 countries. Aggregated subnational HDI was used for the indicator.

Population exposure to SLR is not a proxy indicator for SLR-related population displacement. Mobility and migration away from sites of coastal risk can be prevented or delayed through other adaptive strategies. Further, migration decisions are shaped by social, economic, political, and demographic factors as well as environmental factors; some people may be unable or unwilling to move; and people continue to move into low-lying coastal sites.[113,116] Nonetheless, human mobility away from some places with SLR-related risks is likely in the absence of other adaptation pathways.

For populations exposed to SLR, initial health impacts may emerge from changes in water and food security, disease ecology, flooding and saltwater intrusion, and the psychosocial impacts of disrupted livelihoods.[117,118] For those who move, health outcomes represent an important measure of adaptation. Several case studies identify health risks and opportunities associated with managed retreat away from sites of coastal change, including for mental health, food security, water supply, sanitation, infectious diseases, injury, and health care access.[119-121]

Future form of the indicator

As newer and higher spatial resolution and more precise datasets become available, methods will be updated accordingly to produce robust estimates of population exposure to GMSLR.

National policies

Methods

The methodology for this indicator remains similar to that described in the 2020 report of the Lancet Countdown.[1]

This component of this indicator on national policies reports:

1a. The number of currently valid national-level policies including legislation for migrants, migration, displacement, displaced people, relocation, and relocated people specifically related to climate change (not climate or disasters), including immobility (trapped populations/non-migration/non-displacement).

1b. The number of such policies mentioning health or wellbeing along with a qualitative analysis of how health and/or wellbeing are/is mentioned.

2a. The number of countries with at least one such policy.

2b. The number of such countries whose policies mention health or wellbeing along with a qualitative discussion of how health or wellbeing is mentioned.

“Country” refers sovereign state or autonomous non-sovereign territory (not just a sub-national jurisdiction). Multi-lateral, inter-governmental, and international policies are specifically excluded. Explicit mentions of “climate change” and “health” or “wellbeing” must be present, not implied definitions or references to wider contexts which might (or might not) encompass these points, e.g., “climate”, “climate disasters”, “humanitarian”, and “environment”.

The method for identifying national-level policies is:

1. A systematic review, using the keywords which define the indicator.
2. Crowd-sourcing and expert queries (e.g. IOM).[122]

Because this search can never know what might have been missed, the numbers reported for this indicator represent minimum counts. Each policy included is also categorised by:

1. (a) Migration/mobility/displacement/relocation from a location, (b) migration/mobility/displacement/relocation to a location, and (c) immobility/trapped populations.
2. (a) Domestic migration/mobility/displacement/relocation and (b) international migration/mobility/displacement/relocation.

(All immobility, by definition, is domestic.)

A given policy might be counted in more than one category for 1abc and for 2ab. Some policies do not have an end date and some do, with both included. Policies which are now out-of-date are retained in a separate list as well as a list of policies considered but not included in this indicator.

Data

Table 11: list of identified national-level policies on migration

Country	Policy	Title	Website or other source
Australia		National Climate Resilience and Adaptation Strategy	https://www.environment.gov.au/system/files/resources/3b44e21e-2a78-4809-87c7-a1386e350c29/files/national-climate-resilience-and-adaptation-strategy.pdf
Austria		The Austrian Strategy for Adaptation to Climate Change. Part 1: Context	https://www4.unfccc.int/sites/NAPC/Documents%20NAP/The%20Austrian%20Strategy%20for%20Adaptation%20to%20Climate%20Change.pdf
Bangladesh		Third National Communication of Bangladesh to the United Nations Framework Convention on Climate Change	https://unfccc.int/sites/default/files/resource/TNC%20Report%20(Low%20Resolation)%2003_01_2019.pdf
Bangladesh		National Strategy on the Management of Disaster and Climate Induced Internal Displacement	https://www.preventionweb.net/files/46732_nsmdciidfinalversion21sept2015withc.pdf
Brazil	NAP	National Adaptation Plan to Climate Change — General Strategy<br><br>National Adaptation Plan to Climate Change — Sectoral and Thematic Strategies	https://www4.unfccc.int/sites/NAPC/Documents/Parties/Brazil%20NAP%20English.pdf
Burkina Faso	NAP	Burkina Faso National Climate Change Adaptation Plan (NAP)	https://www4.unfccc.int/sites/NAPC/Documents/Parties/PNA_Version_version%20finale%5bTransmission%5d.pdf
Cameroon	NAP	Plan National d'Adaptation aux Changements Climatiques du Cameroun	https://www4.unfccc.int/sites/NAPC/Documents/Parties/PNACC_Cameroun_VF_Valid%c3%a9e_24062015%20-%20FINAL.pdf
Chad	INDC/NDC	Intended Nationally Determined Contribution (INDC) for the Republic of Chad	https://publications.iom.int/system/files/indcs_and_ndcs.pdf and https://www4.unfccc.int/sites/NDCStaging/Pages/All.aspx and https://www4.unfccc.int/sites/submissions/indc/Submission%20Pages/submissions.aspx
Chile	NAP	Plan Nacional de Adaptación al Cambio Climático<br><br>Plan de Adaptación al Cambio Climático del Sector Silvoagropecuario<br><br>Plan de Adaptación al Cambio Climático en Biodiversidad<br><br>Plan de Adaptación al Cambio Climático para Pesca y Acuicultura	https://www4.unfccc.int/sites/NAPC/Documents/Parties/Chile%20NAP%20including%20sectoral%20plans%20Spanish.pdf
Comoros	INDC/NDC	Contributions Prévues Déterminées au niveau	https://publications.iom.int/system/files/indcs_and_ndcs.pdf and https://www4.unfccc.int/sites/NDCStaging/Page

		National de l'Union des Comores	s/All.aspx and https://www4.unfccc.int/sites/submissions/indc/Submission%20Pages/submissions.aspx
Egypt	INDC/NDC	Egyptian Intended Nationally Determined Contribution	https://publications.iom.int/system/files/indcs_and_ndcs.pdf and https://www4.unfccc.int/sites/NDCStaging/Pages/All.aspx and https://www4.unfccc.int/sites/submissions/indc/Submission%20Pages/submissions.aspx
Fiji		Displacement Guidelines in the Context of Climate Change and Disasters	https://www.adaptationcommunity.net/wp-content/uploads/2020/03/Displacement-Guidelines-Fiji-2019.pdf
Fiji		Planned Relocation Guidelines: A framework to undertake climate change related relocation	https://cop23.com.fj/wp-content/uploads/2018/12/CC-PRG-BOOKLET-22-1.pdf
Fiji	NAP	Republic of Fiji National Adaptation Plan: A pathway towards climate resilience	https://www4.unfccc.int/sites/NAPC/Documents/Parties/National%20Adaptation%20Plan_Fiji.pdf
Germany		German Strategy for Adaptation to Climate Change	https://www.bmu.de/fileadmin/bmu-import/files/english/pdf/application/pdf/das_gesamt_en_bf.pdf
Ghana		Ghana National Climate Change Policy	https://www.un-page.org/files/public/ghanaclimatechangepolicy.pdf
Ghana		National Migration Policy for Ghana	http://www.migratingoutofpoverty.org/files/file.php?name=national-migration-policy-for-ghana.pdf&site=354
Haiti	INDC/NDC	Contribution Prévue Déterminée au niveau National	https://publications.iom.int/system/files/indcs_and_ndcs.pdf and https://www4.unfccc.int/sites/NDCStaging/Pages/All.aspx and https://www4.unfccc.int/sites/submissions/indc/Submission%20Pages/submissions.aspx
Haiti		Politique Migratoire d'Haiti 2015-2030	https://www.academia.edu/16745864/Migration_Policy_of_Haiti_2015-2030_Politique_migratoire_dHa%C3%AFti_2015-2030_
Ireland		National Adaptation Framework - Building Resilience to Climate Change	https://www.dccae.gov.ie/documents/National%20Adaptation%20Framework.pdf
Kenya		Kenya National Adaptation Plan 2015-2030	https://www4.unfccc.int/sites/NAPC/Documents%20NAP/Kenya_NAP_Final.pdf
Kiribati		Kiribati National Framework for Climate Change and Climate Change Adaptation	http://www.president.gov.ki/wp-content/uploads/2014/08/National-Framework-for-Climate-Change-Climate-Change-Adaptation.pdf
Kiribati		Kiribati National Labour Migration Policy	https://www.unescap.org/sites/default/files/Kiribati%20National%20Labour%20Migration%20Policy.pdf
Kiribati	INDC/NDC	Republic of Kiribati Intended Nationally Determined Contribution	https://publications.iom.int/system/files/indcs_and_ndcs.pdf and https://www4.unfccc.int/sites/NDCStaging/Pages/All.aspx and https://www4.unfccc.int/sites/submissions/indc/Submission%20Pages/submissions.aspx
Mali		Programme d'Action National d'Adaptation aux Changements Climatiques	https://www.uncdf.org/article/4754/local-mali-programme-daction-national-dadaptation-aux-changements-climatiques

Mauritius	INDC/NDC	Intended Nationally Determined Contribution for the Republic of Mauritius	https://publications.iom.int/system/files/indcs_and_ndcs.pdf and https://www4.unfccc.int/sites/NDCStaging/Pages/All.aspx and https://www4.unfccc.int/sites/submissions/indc/Submission%20Pages/submissions.aspx
Myanmar	INDC/NDC	Myanmar's Intended Nationally Determined Contribution - INDC	https://publications.iom.int/system/files/indcs_and_ndcs.pdf and https://www4.unfccc.int/sites/NDCStaging/Pages/All.aspx and https://www4.unfccc.int/sites/submissions/indc/Submission%20Pages/submissions.aspx
Nigeria		National Migration Policy 2015	https://publications.iom.int/system/files/pdf/national_migration_policy_2015.pdf
Papua New Guinea	INDC/NDC	Intended Nationally Determined Contribution (INDC) Under the United Nations Framework Convention on Climate Change	https://publications.iom.int/system/files/indcs_and_ndcs.pdf and https://www4.unfccc.int/sites/NDCStaging/Pages/All.aspx and https://www4.unfccc.int/sites/submissions/indc/Submission%20Pages/submissions.aspx
Philippines		National Climate Change Action Plan	http://extwprlegs1.fao.org/docs/pdf/phi152934.pdf
Poland		Polish National Strategy for Adaptation to Climate Change (NAS 2020) - With the perspective by 2030	https://klimada.mos.gov.pl/wp-content/uploads/2014/12/ENG_SPA2020_final.pdf
Rwanda	INDC/NDC	Intended Nationally Determined Contribution (INDC) for the Republic of Rwanda	https://publications.iom.int/system/files/indcs_and_ndcs.pdf and https://www4.unfccc.int/sites/NDCStaging/Pages/All.aspx and https://www4.unfccc.int/sites/submissions/indc/Submission%20Pages/submissions.aspx
Solomon Islands	NAPA	National Adaptation Programmes of Action	https://unfccc.int/resource/docs/napa/slb01.pdf
Somalia	INDC/NDC	Somalia's Intended Nationally Determined Contributions (INDCs)	https://publications.iom.int/system/files/indcs_and_ndcs.pdf and https://www4.unfccc.int/sites/NDCStaging/Pages/All.aspx and https://www4.unfccc.int/sites/submissions/indc/Submission%20Pages/submissions.aspx
South Sudan	INDC/NDC	Intended Nationally Determined Contribution (Draft)	https://publications.iom.int/system/files/indcs_and_ndcs.pdf and https://www4.unfccc.int/sites/NDCStaging/Pages/All.aspx and https://www4.unfccc.int/sites/submissions/indc/Submission%20Pages/submissions.aspx
Sri Lanka	NAP	National Adaptation Plan for Climate Change Impacts in Sri Lanka: 2016-2025	https://www4.unfccc.int/sites/NAPC/Documents/Parties/SLU-NAP-May-2018.pdf
St. Lucia		Saint Lucia's Climate Change Research Strategy 2020-2030	https://napglobalnetwork.org/wp-content/uploads/2020/11/napgn-en-2020-Saint-

			Lucia-Climate-Change-Research-Strategy-2020-2030.pdf
St. Lucia	NAP	Saint Lucia's National Adaptation Plan (NAP) 2018–2028	https://www4.unfccc.int/sites/NAPC/Documents/Parties/SLU-NAP-May-2018.pdf
Sudan	NAP	National Adaptation Plan	https://www4.unfccc.int/sites/NAPC/Documents%20NAP/National%20Reports/Sudan%20NAP.pdf
Suriname	INDC/NDC	Intended Nationally Determined Contribution Under UNFCCC	https://publications.iom.int/system/files/indcs_and_ndcs.pdf and https://www4.unfccc.int/sites/NDCStaging/Pages/All.aspx and https://www4.unfccc.int/sites/submissions/indc/Submission%20Pages/submissions.aspx
Tuvalu		Climate Change Policy 2012-2021	https://reliefweb.int/sites/reliefweb.int/files/resources/TUV_2012_Te_Kaniva_CCpolicy.pdf
Vanuatu		National Policy on Climate Change and Disaster-Induced Displacement	https://ndmo.gov.vu/imhttps://ndmo.gov.vu/images/download/Vanuatu-National-Policy-on-Climate-Change-and-Disaster-Induced-Displacement-2018-published.pdfages/download/Vanuatu-National-Policy-on-Climate-Change-and-Disaster-Induced-Displacement-2018-published.pdf
Zambia		National Climate Change Learning Strategy: Background Report	https://www.uncclearn.org/wp-content/uploads/library/zambia.pdf

Caveats

As documented in previous Lancet Countdown reports[1,73,123,124] and supporting publications[116,125-130], the main problems with using migration or displacement as a climate change and health indicator are:

1. Attributing movement or immobility to climate change or climate change impacts is not straightforward.
2. Attributing health outcomes to movement or immobility is not straightforward.

The evidence to back each of these two attribution relationships is currently weak and it is highly debated in the literature whether or not (i) there are or will be links between climate change and migration, displacement, (im)mobility, relocation and (ii) there are or will be links between migration, displacement, (im)mobility, relocation and health/wellbeing.

This indicator assists in overcoming the attribution problem by:
1. Examining written policies, so attribution is not a concern, because the policies exist, even if attribution is inappropriate.
2. Examining how policies mention health/wellbeing, so again actual attribution is not a concern, because the text on health or wellbeing either exists or does not exist, even if attribution is inappropriate.

If spurious attributions are made in the policies between (i) climate change and migration / displacement / immobility or (ii) migration/displacement/immobility and health or wellbeing, then this indicator can analyse those attributions and why they might not be defensible, based on the scientific literature. Thus, this indicator provides what is happening at the national level and the appropriateness of these policies in terms of the scientific literature. The key to this approach and to overcoming the caveats is keeping the indicator simple and straightforward, which is why the indicator has been designed in the proposed manner.

Selecting policies, and in particular national policies, does not cover all possibilities, but it serves as an indicator. As well, it is an indicator of how national governments perceive the climate change / (im)mobility / health links, without making a statement on the actual links, which the literature explains is exceptionally difficult. This approach to the indicator also means that misattributions are easily filtered out, such as reporting migration and health links to disasters or climate, both of which are different from links to climate change. Using 'climate

change' synonymously with 'climate', 'climate-related disasters', and/or 'disasters', is a common mistake in many policies reviewed as well as in the academic literature.

The main caveat is that most of the data is confined to documents in English, with a few other languages on occasion. The advantage is that policies which are not available in English have typically been discussed in English publications, including blogs and news reports, suggesting that much relevant material has been captured. Nonetheless, the numbers reported can only be taken as the minimum, as in 'at least so many' policies match the criteria stated. One minor caveat is that the number of countries sometimes changes year-to-year, providing a different baseline. These changes are rarely more than 1-2 countries per year out of a sample of around 200. Substantial changes to the numbers of countries will be reported if this occurs.

The indicator design helps in overcoming these caveats by reporting that the counts provided must be only minimum numbers, because it cannot be known what would have been missed. Through publicity, publication, crowdsourcing, and expert connections, this limitation will be overcome because people will provide examples of what has been missed. As an indicator, it is important to accept that the numbers are not comprehensive but provide only minimum numbers as a lower-bound baseline.

Section 2: Adaptation, Planning, and Resilience for Health

2.1: Adaptation Planning and Assessment

Indicator 2.1.1: National Adaptation Plans for Health

Methods

The collection of data for this exercise included a voluntary national survey, the WHO Health and Climate Change Global Survey (2021) that was sent to all WHO Member States and a small number of non-Member territories. The survey was completed by ministry of health focal points. Of the 194 WHO member states and non-Member territories, 70 participated in the survey, providing representation from all 6 WHO regions.Survey participation has grown substantially from the 40 Member States that completed the 2015 WHO Health and Climate Change Global Survey. The survey was planned to be conducted every two years, although global circumstances have resulted in a three-year gap between surveys.

Validation of the 2021 country reported data was undertaken in multiple steps. First, survey responses were reviewed for missing information or inconsistencies with follow-up questions directed to survey respondents. A summary of responses was shared with WHO regional focal points and key informants for review, comments and validation. Source documents including national health strategies and plans, and climate change and health vulnerability and adaptation assessments were collected. A desktop review of these source documents was conducted to compare with survey results with follow-up to survey respondents to seek clarification or additional documentation. Findings were also cross referenced with existing external publications. Data detailing all the ministries, institutions and national stakeholders that provided contributions to or review of the survey responses were collected in order to provide insight into the national consultation process of each survey submission. Finally, all respondents reviewed and acknowledged the WHO data policy statement on the use and sharing of data collected by WHO in Member States outside the context of public health emergencies.

Of note, due to the ongoing pandemic, the standard data collection procedures were modified to reduce reporting burden on countries that wished to participate in the global survey but that were facing human resource constraints due to pandemic response. In 8 cases, WHO prepared pre-filled survey questionnaires with data provided by ministries of health in the previous 2018 survey cycle or using data the countries had published in the 2020/2021 WHO UNFCCC health and climate change country profile when available. These countries were requested to review, revise, and complete the hard copy questionnaires. These hard copy questionnaires were then entered into the online platform by WHO. The same data validation steps as described above were then followed. Additionally, a number of countries requested an extension of the reporting period. As such, there may be a slight increase in the total number of participating countries and the WHO Health and Climate Change Global Survey Report and associated dynamic data dashboard will provide the definitive summary of findings.

Further information on the WHO Health and Climate Change Global Survey, its methodology and the WHO UNFCCC Health and Climate Change Country Profile Project can be found at

https://www.who.int/activities/monitoring-science-and-evidence-on-climate-change-and-health/health-and-climate-global-survey

Data

1. 2021 WHO Health and Climate Change Survey.[131]

Caveats

The survey sample is not a representative sample of all countries as this survey was voluntary, however, the inclusion of 70 countries in this survey, despite a global pandemic, demonstrates significant global coverage.

Future form of the indicator

The WHO Health and Climate Change Global Survey will continue to be the primary source of data to track this indicator.

The future evolution of this indicator will explore the monitoring and review of the existing strategies/plans and progress on level of implementation of strategies/plans. With more countries initiating the national adaptation plan (NAP) process, alignment of the health component with the overall NAP will also be more closely monitored. Interim information regarding the specific content of national strategies/plans, as explored in this qualitative analysis, may be re-assessed in the future.

Additional analysis

Full list of countries participating in the 2021 WHO Health and Climate Change Global Survey: Argentina, Azerbaijan, Bahamas, Bahrain, Barbados, Belize, Benin, Bhutan, Bolivia (Plurinational Bolivia State of), Brazil, British Virgin Islands, Brunei Darussalam, Bulgaria, Cambodia, Cameroon, Canada, Cabo Verde, China, Colombia, Comoros, Costa Rica, Côte d'Ivoire, Croatia, Cuba, Cyprus, Czech Republic, Dominica, Dominican Republic, Egypt, El Salvador, Eritrea, Estonia, Ethiopia, Ghana, Grenada, Guatemala, Guinea, Guyana, Haiti, India, Iran (Islamic Republic of), Israel, Jamaica, Jordan, Kazakhstan, Kenya, Kyrgyzstan, Lebanon, Lithuania, Madagascar, Marshall Islands, Micronesia (Federated States of), Mozambique, Netherlands, Nicaragua, Nigeria, North Macedonia, occupied Palestinian territory, Oman, Palau, Papua New Guinea, Paraguay, Peru, Philippines, Poland, Portugal, Rwanda, Republic of Moldova, Saint Kitts and Nevis, Saint Lucia, San Marino, Sao Tome And Principe, Saudi Arabia, Serbia, Seychelles, Sierra Leone, Slovakia, South Africa, Sri Lanka, Suriname, Sweden, Thailand, Togo, Trinidad and Tobago, Turkmenistan, United Republic of Tanzania, Uruguay, Vanuatu, Yemen, Zimbabwe.

Indicator 2.1.2: National Assessments of Climate Change Impacts, Vulnerability, and Adaptation for Health

Methods

The collection of data for this exercise included a voluntary national survey, the WHO Health and Climate Change Global Survey (2021) that was sent to all WHO Member States and a small number of non-Member territories. The survey was completed by ministry of health focal points. Of the 194 WHO member states and non-Member territories, 70 participated in the survey, providing representation from all 6 WHO regions.

Survey participation has grown substantially from the 40 Member States that completed the 2015 WHO Health and Climate Change Global Survey. The survey was planned to be conducted every two years, although global circumstances have resulted in a three-year gap between surveys.

Validation of the 2021 country reported data was undertaken in multiple steps. First, survey responses were reviewed for missing information or inconsistencies with follow-up questions directed to survey respondents. A summary of responses was shared with WHO regional focal points and key informants for review, comments and validation. Source documents including national health strategies and plans, and climate change and health vulnerability and adaptation assessments were collected. A desktop review of these source documents was conducted to compare with survey results with follow-up to survey respondents to seek clarification or additional documentation. Findings were also cross referenced with existing external publications. Data were collected detailing all the ministries, institutions and national stakeholders that provided contributions to or review of the survey responses in order to provide insight into the national consultation process of each survey submission. Finally, all respondents reviewed and acknowledged the WHO data policy statement on the use and sharing of data collected by WHO in Member States outside the context of public health emergencies.

Of note, due to the ongoing pandemic, the standard data collection procedures were modified to reduce reporting burden on countries that wished to participate in the global survey but that were facing human resource constraints due to pandemic response. In 8 cases, WHO prepared pre-filled survey questionnaires with data provided by ministries of health in the previous 2018 survey cycle or using data the countries had published in the 2020/2021 WHO UNFCCC health and climate change country profile when available. These countries were requested to review, revise, and complete the hard copy questionnaires. These hard copy questionnaires were then entered into the online platform by WHO. The same data validation steps as described above were then followed. Additionally, a number of countries requested an extension of the reporting period. As such, there may be a slight increase in the total number of participating countries and the WHO Health and Climate Change Global Survey Report and associated dynamic data dashboard will provide the definitive summary of findings.

Further information on the WHO Health and Climate Change Global Survey, its methodology and the WHO UNFCCC Health and Climate Change Country Profile Project can be found at https://www.who.int/activities/monitoring-science-and-evidence-on-climate-change-and-health/health-and-climate-global-survey

Data

1. 2021 WHO Health and Climate Change Survey.[131]

Caveats

The survey sample is not a representative sample of all countries as this survey was voluntary, however, the inclusion of 70 countries in this survey, despite a global pandemic, demonstrates significant global coverage.

Future form of the indicator

The WHO Health and Climate Change Global Survey will continue to be the primary source of data to track this indicator. The future evolution of this indicator will explore the coverage and comprehensive of the assessments, the consideration of vulnerable population groups and the use of findings to inform national policy.

Additional analysis

Full list of countries participating in the 2021 WHO Health and Climate Change Global Survey: Argentina, Azerbaijan, Bahamas, Bahrain, Barbados, Belize, Benin, Bhutan, Bolivia (Plurinational Bolivia State of), Brazil, British Virgin Islands, Brunei Darussalam, Bulgaria, Cambodia, Cameroon, Canada, Cabo Verde, China, Colombia, Comoros, Costa Rica, Côte d'Ivoire, Croatia, Cuba, Cyprus, Czech Republic, Dominica, Dominican Republic, Egypt, El Salvador, Eritrea, Estonia, Ethiopia, Ghana, Grenada, Guatemala, Guinea, Guyana, Haiti, India, Iran (Islamic Republic of), Israel, Jamaica, Jordan, Kazakhstan, Kenya, Kyrgyzstan, Lebanon, Lithuania, Madagascar, Marshall Islands, Micronesia (Federated States of), Mozambique, Netherlands, Nicaragua, Nigeria, North Macedonia, occupied Palestinian territory, Oman, Palau, Papua New Guinea, Paraguay, Peru, Philippines, Poland, Portugal, Rwanda, Republic of Moldova, Saint Kitts and Nevis, Saint Lucia, San Marino, Sao Tome And Principe, Saudi Arabia, Serbia, Seychelles, Sierra Leone, Slovakia, South Africa, Sri Lanka, Suriname, Sweden, Thailand, Togo, Trinidad and Tobago, Turkmenistan, United Republic of Tanzania, Uruguay, Vanuatu, Yemen, Zimbabwe.

Indicator 2.1.3: City-Level Climate Change Risk Assessments

Methods

The CDP serves as an official reporting platform for the Compact of Mayors. It administrates, collects, and analyses a global survey of city based environmental and climate change data on an annual basis. It includes questions on mitigation, on emissions, adaptation assessments and plans.

In 2020, 776 cities participated in the relevant questions of the survey for this indicator, with 670 reporting publicly. This represents a 4.7% decrease in cities responding to question 2 of the CDP survey overall and a 15.1% decrease in cities reporting publicly that they included questions on emissions, adaptation assessments and plans from 2019 to 2020 (814 and 789 cities respectively in 2019).[1]

Respondents to the survey were asked to describe the magnitude of the impact of climate-based hazards (extremely serious, serious, less serious) and identify three critical assets or services that may be most impacted. In the 2020 survey, respondents were also asked additional questions relating to health risks, including to:

- Identify the climate hazards most significantly impacting their select critical assets
- Identify the climate-related health issues faced by their city
- Identify the vulnerable populations affected by the climate-related impacts
- Identify the timescale of climate-related issues for their selected health area

These data were collected in partnership by CDP and ICLEI - Local Governments for Sustainability.

Based on these data two indicators can be developed:

- The first is a global cities-based indicator of government areas that have undertaken a climate change risk or vulnerability assessment;
- The second is global cities-based indicator of the perceived vulnerability of public health assets and service to climate change, along with further analysis of the climate-related health issues and vulnerable populations identified by cities.

Data

1. 2020 CDP Annual Cities Survey.[132]

Caveats

This is a sample survey and cities are under no obligation to respond. As such the survey may suffer from selection bias. The majority of responding cities are also from countries rated as high or very high HDI (94%). As such, the results are not representative of all cities.

Future form of the indicator

The CDP collect this data annually and it is foreseen that the data collection will continue to 2030. Additional analyses may be conducted using data from the CDP annual survey to monitor associations between city-level health vulnerabilities and track reporting trends over time.

Additional analysis

Table 12: Cities that responded to the 2020 CDP survey, by country-level HDI categories

	Cities who responded to the 2020 CDP survey question 2.0, regarding completion of a climate change risk assessment, by country-level of HDI		Cities who responded to the 2020 CDP survey question 2.3, regarding climate-related health, by country-level of HDI	
**HDI Category**	**Frequency**	**Percentage**	**Frequency**	**Percentage**
NA	1	0.1%	1	0.2%
Low	10	1.5%	11	2.2%
Medium	28	4.2%	21	4.2%
High	196	29.3%	145	29.2%
Very High	435	64.9%	318	64.1%
**Total**	**670**	**100.0%**	**496**	**100.0%**

Table 13: Cities that responded to the 2020 CDP survey, by WHO regions

	Cities who responded to the 2020 CDP survey question 2.0, regarding completion of a climate change risk assessment, by WHO regions		Cities who responded to the 2020 CDP survey question 2.3, regarding climate-related health, by WHO regions	
**WHO Region**	**Frequency**	**Percentage**	**Frequency**	**Percentage**
Western Pacific	68	10.1%	53	10.7%
African	26	3.9%	24	4.8%
Americas	410	61.2%	279	56.3%
European	144	21.5%	122	24.6%
South-East Asian	15	2.2%	15	3.0%
Eastern Mediterranean	7	1.0%	3	0.6%
**Total**	**670**	**100.0%**	**496**	**100.0%**

Table 14: Frequency of cities reporting undertaking a climate-change risk assessment by WHO region

**Country**	**Not intending to undertake**	**Intending to undertake in the next 2 years**	**In progress**	**Yes**	**Do not know**
Western Pacific	3	4	7	54	0
African	2	1	4	18	1
Americas	3	62	47	272	26
European	6	10	14	113	1
South-East Asian	0	3	1	11	0
Eastern Mediterranean	1	1	2	3	0
**Total**	**15**	**81**	**75**	**471**	**28**

Table 15: Frequency of cities reporting climate-related health issues by WHO region

Country	No	Do Not Know	Yes
Western Pacific	10	5	38
African	2	1	21
Americas	46	61	172
European	32	23	67
South-East Asian	5	1	9
Eastern Mediterranean	1	1	1
**Total**	**96**	**92**	**308**

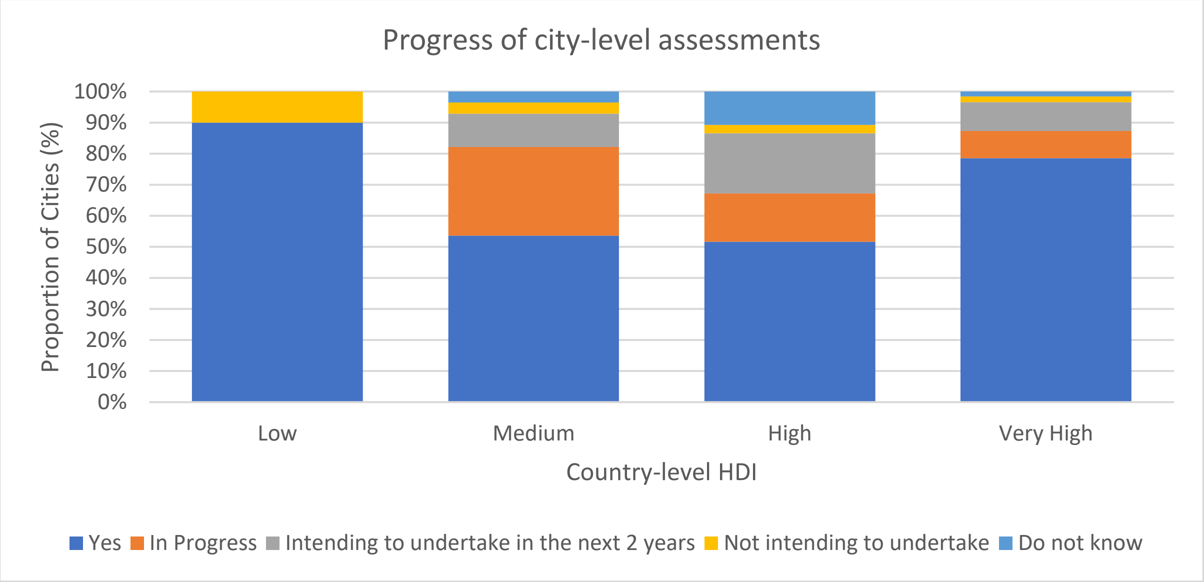

Figure 41: Proportion of cities that have conducted climate change risk assessments, by country-level HDI grouping

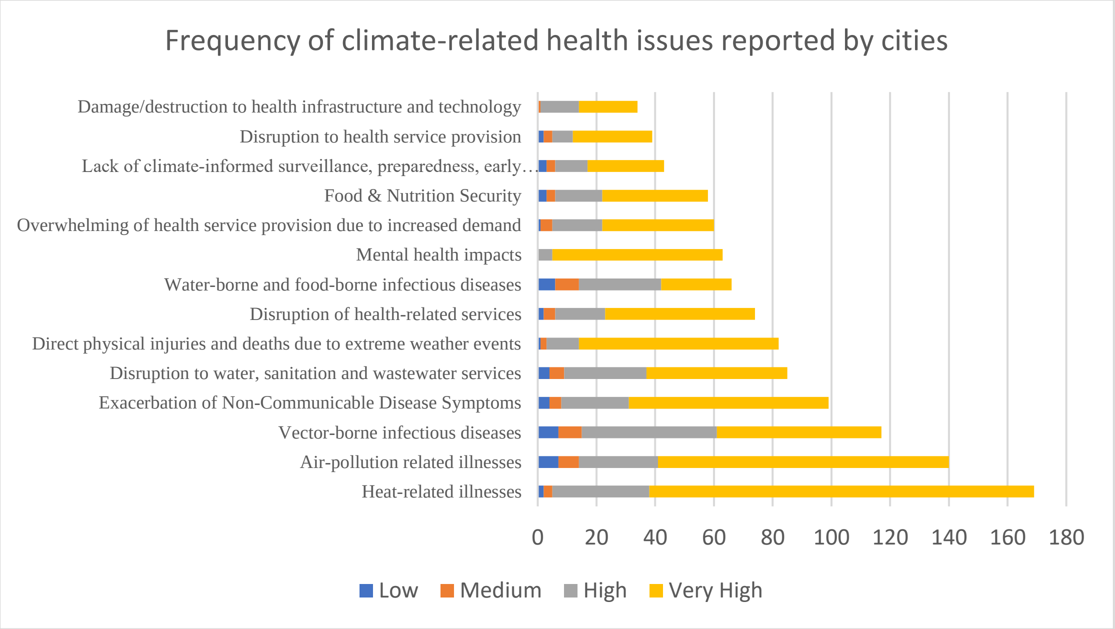

Figure 42: Frequency of climate-related health issues, reported by the 308 cities in the 2020 CDP survey who identified climate-related health risks, per country-level HDI

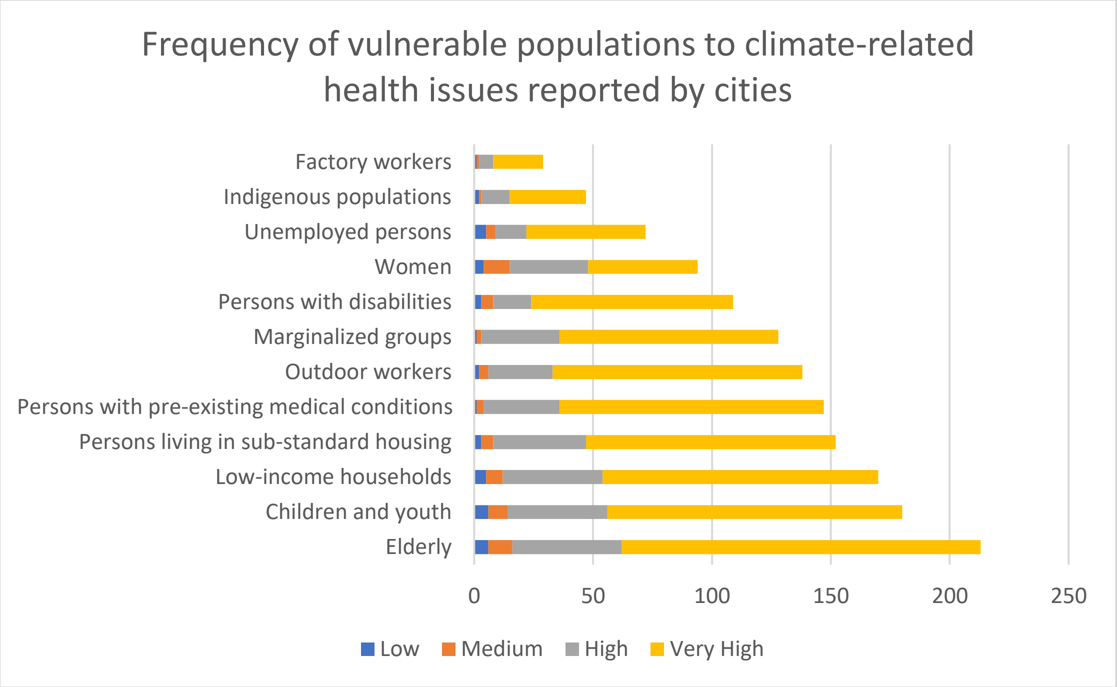

Figure 43: Frequency of vulnerable populations to climate-related health issues, reported by the 308 cities in the 2020 CDP survey who identified climate-related health risks, per country-level HDI

Indicator 2.2: Climate Information Services for Health

Methods

The number of World Meteorological Organization (WMO) national meteorological and hydrological services (NMHS) providing climate services to the health sector is calculated based on self-reported information provided by NMHS through the Country Profile Database Integrated questionnaire. The questionnaire is one of the main sources of information to the WMO Country Profile database and is open all year round for WMO members to update their profile information. Data for the 2021 Lancet Countdown report represent a data update from November 2020.

Reported data reflects answers to Question number 7.6 of this questionnaire: "Please indicate which user communities/sectors your NMHS provides with climate products/information and estimate the extent to which these products are used to improve decisions". "Human Health" is one of multiple sectors which can be chosen.

For countries who identified that their NMHS engaged with the health sector, data were also collected on the level of engagement between the NMHS and the health sector. Respondents were asked to rate their level of engagement with the health sector on a scale from one to six, according to the definitions in the table below:

Table 16: level of engagement between NMHS and health sector

Level of Engagement	Definition
1	Initial engagement with sector
2	Definition of needs
3	Co-design of products
4	Tailored products accessible for use
5	Climate services guide policy decisions and investment plans in sectors
6	Documentation of socio-economic benefits

Data

1. World Meteorological Organization Country Profile database.[133]

Caveats

The current data source from WMO only considers climate services provided by NMHS. It is unclear the degree to which other providers, such as academic institutions and research projects, private sector products, products from other Ministries, or regional and global products and services are being used, in proportion to services made available by NMHS.

The open questionnaire can be updated at any time by WMO members, therefore the figures reported here may change over the year. As each country may update their profile information at different moments in time, snapshots do not reflect progress for any given year but rather information provided until a certain date.

The current questionnaire does not record the number of WMO members that do not provide climate services to the health sector.

The questionnaire captures information on the provision of climate services, the status of service provision to the health sector (divided in 5 categories) and the type of services provided (divided in 5 categories as well). However, only the provision and status of climate service has been reported here due to uncertainties over the quality of the data on the type of services provided. Questions do not capture the source or quality of the service and only one of the answer options covers the utility of the climate services. They do not capture whether data originates from national meteorological observations or is resulting from regional or global products. They do not capture the potential use of all-sector forecasts or outlooks which are accessed and used by the health sector.

The WMO and WHO have some differences in their individual Member States. Responses collected from WMO Member States were reclassified according to WHO Region. WMO members that are not individual WHO members were excluded from the analyses and include Macao and Hong Kong (reported as China), Curaçao,

French Polynesia, and St. Maartens. The following WHO Members are not members of WMO (and therefore representative data is not available): Andorra, Equatorial Guinea, Marshall Islands, Nauru, Palau, San Marino.

Future form of the indicator

In 2019, WMO began implementation of new survey instruments to provide greater insight on the status of climate service provision for the health sector and the type of service provided. Other complementary WMO surveys capturing specific product types, user satisfaction, and application areas, may be publicly available in the future to inform future editions of this indicator.

The WHO Health and Climate Change Country Survey now contains indicators on the inclusion of meteorological information in integrated risk monitoring and early warning systems for climate-sensitive diseases. This information may be used to improve this indicator in future publications.

Additional analysis

Table 17: Provision of climate services from national meteorological and hydrological services by sector

**Sector**	**Number of countries providing climate services**
Government	105
Agriculture	99
Local authorities	95
Water resources	94
Scientific	90
Energy industry	86
**Human health**	**86**
Emergency planning and response	86
Transport	81
Environmental protection	76
Building	76
Aviation	75
Commercial	73
Tourism (incl. coastal zone)	71
Finance and insurance	71
Forestry	67
Fisheries	59
Recreation, sport	59
Maritime transport	54

66 out of 86 responding countries that reported providing climate information services to the health sector provided additional information on how well the climatic services are integrated in the health sector:

Table 18: Reported level of engagement between NMS and health sector

**Level of Engagement**	**Number of Countries Reporting this Level of Engagement**
1 Initial engagement with sector	12
2 Definition of needs	18
3 Co-design of products	12
4 Tailored products accessible for use	19
5 Climate services guide policy decisions and investment plans in sector	5
6 Documentation of socio-economic benefits	0
**Total**	**66**

Full list of WHO member states reporting providing climate services to the health sector: Angola, Argentina, Armenia, Antigua and Barbuda, Australia, Austria, Belgium, Bosnia and Herzegovina, Brazil, Barbados, Brunei Darussalam, Bhutan, Canada, Chile, China, Côte d'Ivoire, Cameroon, Cook Islands, Colombia, Cyprus, Germany, Djibouti, Dominica, Ecuador, Egypt, Spain, Finland, Fiji, France, United Kingdom of Great Britain and Northern Ireland, Georgia, Guinea-Bissau, Croatia, Hungary, Indonesia, India, Ireland, Iraq, Iceland, Jamaica, Japan, Kazakhstan, Kenya, Republic of Korea, Sri Lanka, Lesotho, Latvia, Morocco, Madagascar, Maldives, Mexico, North Macedonia, Mali, Myanmar, Mozambique, Malawi, Malaysia, Namibia, Niger, Nigeria, Niue, Nepal, Peru, Philippines , Qatar, Russian Federation, Saudi Arabia, Sudan, Senegal, Singapore, Solomon Islands, El Salvador, Serbia, Sao Tome and Principe, Slovenia, Chad, Thailand, Trinidad and Tobago, Turkey, United Republic of Tanzania, Ukraine, United States of America, Venezuela (Bolivarian Republic of), Vanuatu, South Africa, Zimbabwe

2.3: Adaptation Delivery and Implementation

Indicator 2.3.1: Detection, Preparedness and Response to Health Emergencies

Methods

This indicator takes data from the International Health Regulations (IHR (2005)) State Party Self-Assessment Annual Reporting Tool (SPAR).

Under the IHR (2005) all States Parties are required to have or to develop minimum core public health capacities to implement the IHR (2005) effectively. IHR (2005) also states that all States Parties should report to the World Health Assembly annually on the implementation of IHR (2005). In order to facilitate this process, WHO developed an IHR Monitoring questionnaire, interpreting the Core Capacity Requirements in Annex 1 of IHR (2005) into 20 indicators for 13 capacities. Since 2010, this self-reporting IHR monitoring questionnaire is sent annually to National IHR Focal Points (NFPs) for data collection. It contains a checklist of 20 indicators specifically developed for monitoring the development and implementation of 13 IHR capacities. The method of estimation calculates the proportion/percentage of attributes (a set of specific elements or functions which reflect the level of performance or achievement of a specific indicator) reported to be in place in a country.

The core capacities to implement the IHR (2005) have been established by a technical group of experts, as those capacities required to detect, assess, notify and report events, and to respond to public health risks and emergencies of national and international concern. To assess the development and strengthening of core capacities, a set of components are measured for each of the core capacities, by considering a set of one to three indicators that measure the status and progress in developing and strengthening the IHR core capacities. Each indicator is assessed by using a group of specific elements referred to as ‘attributes’ that represents a complex set of activities or elements required to carry out this component. The annual questionnaire has been conducted since 2010 with a response rate of 72% in 2012, 66% in 2016 and 85% in 2017, and 100% of countries reporting at least once since 2010. Annual reporting results are complemented by after action reviews, exercises, and joint external evaluation (JEE).

At the beginning of 2018, in compliance with the recommendations of the IHR Review Committee on Second Extensions for Establishing National Public Health Capacities and on IHR Implementation and following formal global consultations with States Parties held in 2015, 2016, and 2017, and 2018, the WHO Secretariat replaced the IHR Monitoring questionnaire by the “IHR State Party Self-assessment Annual Reporting (SPAR) Tool”. This has strong implication for the future of this indicator: preparedness and response capacities have now been merged into one capacity called “C8: National health emergency framework”; one capacity relevant to climate adaptation and resilience has been added (“C9: Health services provision”); and capacity grading has been introduced, which requires countries to grade their capacity indicators in progressive levels from 0 to 5 as opposed to the previous “Yes/No/Not know” answers options. C8 contains three components. A full breakdown of the 0-5 scale for each of the three components is provided in the 2019 Lancet Countdown report appendix.

To obtain an implementation rating, data were classified according to the table below:

Table 19. Categorisation of 'Level of Implementation' of Core Capacity 8 of the IHR SPAR tool

Level of Implementation Classification	Score
Low	0-24%
Medium-Low	25-49%
Medium-High	50-74%
High	75-100%

Data

1. International Health Regulations (2005) Annual Reporting. Data is available through the Global Health Observatory Data Repository for 2010-2017,[134] and through the SPAR interactive for 2020.

Caveats

There are some limitations to considering these capacities as proxies of health system adaptive capacity and system resilience. Most importantly, IHR monitoring questionnaire responses are self-reported. Secondly, the countries that report IHR implementation differ from year to year within these regional aggregate scores. Thirdly, IHR Core Capacity Requirements are not specific to climate change, and hence whilst they provide a proxy baseline, they do not directly measure a country's adaptive capacity in relation to climate driven risk changes. Fourthly, these findings capture potential capacity – not action. Finally, the quality of surveillance for early detection and warning is not shown and neither is the impact of that surveillance on public health. Response systems have been inadequate in numerous public health emergencies and thus the presence of such plans is not a proxy for their effectiveness. Nevertheless, these capacities provide a useful starting point to consider the potential adaptive capacity of health systems globally.

Future form of the indicator

The World Health Assembly resolution WHA73.1 requested the WHO Director-General to initiate a process of impartial, independent and comprehensive evaluation of the WHO-coordinated international health response to COVID-19, including the mechanisms in place under the IHR. Future forms of this indicator will need to evolve along with the outputs of this review.

Multiple different indices exist which measure different elements of health emergency preparedness. This indicator will be improved in collaboration with the WHO, to identify if any of these complementary indices can be integrated to provide a more holistic evaluation of the capacity of health systems to respond to different types of global health emergencies.

Indicator 2.3.2: Air Conditioning Benefits and Harms

Methods

The Lancet Countdown 2019 and 2020 reports presented calendar-time trends in the prevented fraction of heatwave-related deaths due to air conditioning, which is the percent reduction in heatwave-related deaths due to a given proportion of the population having household air conditioning, compared with a counterfactual scenario of complete absence of household air conditioning, for the world, selected countries, and regions. Although the prevented fraction is an informative metric, it does not provide information on the absolute number of heatwave- (or heat-) related deaths prevented by air conditioning (the distinction between heatwave-related deaths and heat-related deaths is discussed in the Caveats below; henceforth the term heat-related deaths is used). Thus, if the number of heat-related deaths that would occur in the complete absence of household air conditioning were very low in a given country or region, the number of deaths averted by air conditioning also would be low, even if the prevented fraction were high.

The 2021 report overcomes the limitation of using the prevented fraction by utilising results from Indicator 1.1.6, Heat-related mortality, introduced in the 2020 report, which estimated heat-related deaths for the world and by country in the population aged 65 years and older. In the 2021 report, these results are utilised, in combination with prevented fraction estimates, to estimate the number of heat-related deaths prevented by air conditioning in the 65-and-older population for the world and by selected countries and regions.

Prevented fraction

The prevented fraction (PF) is the percent reduction in an adverse health outcome due to a preventive exposure, compared with the scenario of complete absence of the exposure.[135] The prevented fraction is determined by two factors: 1) the relative risk of the adverse health outcome in exposed persons compared with unexposed persons; and 2) the prevalence of the exposure. The prevented fraction increases with decreasing relative risk below the null and with increasing prevalence of exposure. The formula for prevented fraction is simply:

Pe(1 – RR)

Where Pe is the prevalence of the exposure and RR is the relative risk of the adverse health outcome in exposed persons compared with unexposed persons.

For the air conditioning indicator, the prevented fraction is the percent reduction in heat-related deaths due to a given proportion of the population having household air conditioning, compared with a scenario of complete absence of household air conditioning. Thus, the prevented fraction is simply:

Pac(1 – RRac)

Where Pac is the proportion of the population having household air conditioning and RRac is the relative risk of heat-related death among persons who have household air conditioning compared with persons who do not have household air conditioning.

As intuitively expected, according to this formula, the stronger the protection against heat-related mortality conferred by household air conditioning (i.e., the lower the relative risk of heat-related mortality in persons living in a household with air conditioning versus persons living in a household without air conditioning), the greater the prevented fraction, and the higher the proportion of the population with access to household air conditioning, the greater the prevented fraction.

The International Energy Agency (IEA) kindly provided data on the proportion of households with air conditioning for the entire world and for 22 individual countries and 9 IEA-defined regions that did not include the 22 individual countries. The countries and regions together constituted the entire world. The proportion of households with air conditioning is assumed to be a reasonable estimate of the proportion of the population ≥65 years of age having household air conditioning (Pac).

RRac was estimated using the results of the same meta-analysis conducted for the 2020 report. Briefly, a literature search was conducted for non-ecologic, analytical epidemiologic studies that examined the relationship between

availability of household air conditioning and heat-related mortality, and identified 9 eligible studies.[136-144] In a random-effects meta-analysis, RRac was calculated to be 0.24 (95% confidence interval: 0.15, 0.39), which was used to calculate the prevented fraction for the world and each country and region.

Thus, the formula for prevented fraction is:

Pac(1 – RRac) = Pac(1 – 0.24) = Pac(0.76)

The prevented fraction could range from 0 for a country or region with no household air conditioning (i.e., Pac = 0) to 76% for a country or region in which every household has air conditioning (i.e., Pac = 1.0).

Number of heat-related deaths averted by air conditioning in the 65-and-older population

Estimation of the number of heat-related deaths averted[145] by air conditioning is based on estimates of country- and region-specific prevented fractions and on estimates of the number of calendar-year- and country-specific number of heat-related deaths in persons aged 65 years and older, taken from Indicator 1.1.6, Heat-related mortality. These latter estimates constitute the “observed” number of heat-related deaths, given the proportion of the population having household air conditioning in each calendar year and country (Do). To calculate Do for each IEA-defined region, Do’s across the countries classified into each region were summed.

The number of heat-related deaths that would be expected in the complete absence of household air conditioning (De) was then estimated as:

De = Do/(1-PF)

Finally, the number of heat-related deaths averted due to the presence of household air conditioning (Da) was estimated as:

Da = De – Do

To calculate the 95% confidence interval for Da, the uncertainty in RRac and in Do were accounted for using the delta method.[146]

Da estimates for the world were calculated by summing the Da estimates over the 22 individual countries and 9 IEA-defined regions for which IEA provided data on the proportion of households with air conditioning. These countries and regions together constituted the entire world.

Premature deaths from ambient $PM_{2.5}$ exposure due to electricity use for air conditioning in 2019

To estimate country/region-specific premature deaths from ambient $PM_{2.5}$ exposure due to electricity use for air conditioning in 2019, the proportion of total electricity final consumption used for air conditioning (obtained from IEA) was multiplied by the estimated country/region-specific premature deaths due to $PM_{2.5}$ emissions from electric power plants, taken from Indicator 3.3. Indicator 3.3 estimated premature deaths from ambient $PM_{2.5}$ exposure for 138 countries. To calculate premature deaths from ambient $PM_{2.5}$ exposure for each IEA-defined region, premature deaths from ambient $PM_{2.5}$ exposure across the countries classified into each region were summed.

Data

The IEA kindly provided data for 2000-2019, including revisions based on improved IEA analyses of its 2000-2018 data used in the 2020 Lancet Countdown report. These data included the proportion of households with air conditioning (used for the prevented fraction calculation); CO_2 emissions due to air conditioning (megatons); and proportion of total electricity final consumption used for air conditioning (used in the calculation of premature deaths from ambient $PM_{2.5}$ exposure due to electricity use for air conditioning).

Proportion of households with air conditioning and CO_2 emissions due to air conditioning were provided for the entire world and for 22 individual countries and 9 IEA-defined regions that did not include the 22 individual countries. The countries and regions together constituted the entire world.

The following are the individual countries: Canada, United States, Russia, Australia, New Zealand, Italy, France, Denmark, Finland, Sweden, United Kingdom, Norway, Iceland, Germany, Mexico, Japan, South Korea, Brazil, China, India. Indonesia, and South Africa

The following are the 9 regions (the 22 individual countries were not included in the regions):

a. Caspian: Armenia, Azerbaijan, Georgia, Kazakhstan, Kyrgyzstan, Tajikistan, Turkmenistan, Uzbekistan

b. Other Europe: Albania, Austria, Belarus, Belgium, Bosnia and Herzegovina, Bulgaria, Croatia, Cyprus, Czech Republic, Estonia, Former Yugoslav Republic of Macedonia, Gibraltar, Greece, Holy See, Hungary, Ireland, Israel, Kosovo, Latvia, Lithuania, Luxembourg, Malta, Moldova, Monaco, Montenegro, Netherlands, Poland, Portugal, Romania, San Marino, Serbia, Slovak Republic, Slovenia, Spain, Switzerland, Turkey, Ukraine

c. North Africa: Algeria, Egypt, Libya, Morocco, Tunisia

d. Other Africa: Angola, Benin, Botswana, Burkina Faso, Burundi, Cameroon, Cape Verde, Central African Republic, Chad, Comoros, Congo, Côte d'Ivoire, Dem. Republic of the Congo, Djibouti, Equatorial Guinea, Eritrea, Eswatini, Ethiopia, Gabon, Gambia, Ghana, Guinea, Guinea-Bissau, Kenya, Lesotho, Liberia, Madagascar, Malawi, Mali, Mauritania, Mauritius, Mozambique, Namibia, Niger, Nigeria, Réunion, Rwanda, Sao Tome and Principe, Senegal, Seychelles, Sierra Leone, Somalia, South Sudan, Sudan, Togo, Uganda, Tanzania, Zambia, Zimbabwe

e. Chile and Colombia: Chile, Colombia

f. Other Latin America: Antigua and Barbuda, Argentina, Aruba, Bahamas, Barbados, Belize, Bermuda, Bolivia, British Virgin Islands, Cayman Islands, Costa Rica, Cuba, Curaçao, Dominica, Dominican Republic, Ecuador, El Salvador, Falkland Islands (Malvinas), French Guiana, Grenada, Guadeloupe, Guatemala, Guyana, Haiti, Honduras, Jamaica, Martinique, Montserrat, Netherlands (Caribbean), Netherlands Antilles, Nicaragua, Panama, Paraguay, Peru, Saint Kitts and Nevis, Saint Lucia, Saint Pierre and Miquelon, Saint Vincent and the Grenadines, Sint Maarten (Dutch part), Suriname, Trinidad and Tobago, Turks and Caicos Islands, Uruguay, Venezuela

e. Middle East: Bahrain, Iran, Iraq, Jordan, Kuwait, Lebanon, Oman, Qatar, Saudi Arabia, Syria, United Arab Emirates, Yemen

h. Association of Southeast Asian Nations (ASEAN) countries: Brunei Darussalam, Cambodia, Laos, Malaysia, Myanmar, Philippines, Pitcairn, Singapore, Thailand, Vietnam

i. Other Asia: Afghanistan, Bangladesh, Bhutan, China (Macau, China (Taiwan), Cook Islands, Fiji, French Polynesia, Kiribati, Kuwait, Lebanon, Maldives, Mongolia, Nepal, New Caledonia, North Korea, Pakistan, Palau, Papua New Guinea, Samoa, Solomon Islands, Sri Lanka, Timor-Leste, Tonga, Vanuatu

In the estimation of number of heat-related deaths averted by air conditioning, the following countries were not included in the calculation of IEA-defined-region-level Do's because, although they were included in IEA regions, they were not included in the assessment of number of heat-related deaths:

Other Europe: Gibraltar, Holy See, Kosovo, Malta, Monaco, San Marino

Other Africa: Réunion, Seychelles

Other Latin America: Antigua and Barbuda, Aruba, Barbados, Bermuda, British Virgin Islands, Cayman Islands, Curacao, Dominica, Falkland Islands (Malvinas), Grenada, Guadeloupe, Martinique, Montserrat, Netherlands Antilles, Saint Kitts and Nevis, Saint Lucia, Saint Pierre and Miquelon, Sint Maarten (Dutch part), Turks and Caicos Islands

Middle East: Bahrain

ASEAN countries: Brunei Darussalam, Pitcairn, Singapore

Other Asia: China (Macau), China (Taiwan), Cook Islands, French Polynesia, Maldives, New Caledonia, Palau, Tonga, Vanuatu

In addition, the following countries or territories were not included in the calculation of IEA-defined-region-level Do's because, although they were included in the assessment of number of heat-related deaths, they were not included in IEA regions: Puerto Rico, U.S. Virgin Islands, Western Sahara.

For the estimation of premature deaths from ambient $PM_{2.5}$ exposure due to electricity use for air conditioning, the data provided by the IEA on proportion of total electricity final consumption used for air conditioning grouped Australia and New Zealand; grouped Italy, France, and Germany; and included Denmark, Finland, Iceland, Norway, and Sweden in "Other Europe. In addition, the following countries were not included in the calculation of IEA-defined-region-level number of premature deaths due to $PM_{2.5}$ emissions from electric power plants because, although they were included in IEA regions, they were not included in the assessment of number premature deaths due to $PM_{2.5}$ emissions from electric power plants in Indicator 3.3:

Caspian: Tajikistan, Turkmenistan, Uzbekistan

Other Europe: Gibraltar, Holy See, Kosovo, Monaco, San Marino

Other Africa: Réunion, Sao Tome and Principe, Seychelles, South Sudan

Other Latin America: Antigua and Barbuda, Aruba, Bahamas, Barbados, Belize, Bermuda, British Virgin Islands, Cayman Islands, Costa Rica, Cuba, Curaçao, Dominica, Dominican Republic, El Salvador, Falkland Islands (Malvinas), French Guiana, Grenada, Guadeloupe, Guatemala, Guyana, Haiti, Honduras, Jamaica, Martinique, Montserrat, Netherlands Antilles, Nicaragua, Panama, Saint Kitts and Nevis, Saint Lucia, Saint Pierre and Miquelon, Saint Vincent and the Grenadines, Sint Maarten (Dutch part), Suriname, Trinidad and Tobago, Turks and Caicos Islands

Middle East: Bahrain, Iraq, Jordan, Oman, Qatar, Syria, United Arab Emirates, Yemen

ASEAN countries: Pitcairn

Other Asia: China (Macau), Cook Islands, Kuwait, Lebanon, Fiji, French Polynesia, Kiribati, Maldives, New Caledonia, Palau, Papua New Guinea, Samoa, Solomon Islands, Timor-Leste, Tonga, Vanuatu

Caveats

Estimate of number of heat-related deaths averted by air conditioning

There were a number of limitations to the estimate of number of heat-related deaths averted by air conditioning in the 65-and-older population, such that this is considered to be a "ballpark" estimate that will need considerable refinement in future years:

1. The prevented fraction calculation was based on a pooled RRac of 0.24 from a meta-analysis that included nine studies: four from the United States; two from France; one from Italy, one from Greece, and one from Australia. This RRac may differ in other parts of the world, but studies of the relationship between availability of household air conditioning and heat-related mortality are sparse, such that it is not currently possible to make region-specific estimates of RRac.

2. The target population for four of the nine studies included in the meta-analysis was the general population, whereas the target population for five of the studies was the elderly (persons ≥65 years of age in two studies, persons ≥75 years of age in one study, and nursing home residents in two studies). Because the target population of Indicator 1.1.6, Heat-related mortality was ≥65 years of age, restricting the meta-analysis to the five studies that focused on the elderly was considered. However, when it was found that one of the five studies contributed 73% of the weight in the restricted meta-analysis, the decision was made not to apply this restriction so as not to allow a single study to have such a high amount of influence on the estimate of RRac. For the nine studies, to build greater uncertainly into the analysis, a random-effects meta-analysis, that assumes heterogeneity among studies and results in a wider 95% confidence interval than would a fixed-effects meta-analysis, was conducted. Nevertheless, it is possible that RRac differs between persons ≥65 years of age and younger persons.

3. Eight of the nine studies in the meta-analysis estimated the relative risk of heat-related death among persons who have household air conditioning compared with persons who do not have household air conditioning (i.e., RRac) during heatwaves (e.g., the 2003 French heatwave, the 1995 Chicago heatwave). However, in Indicator 1.1.6, Heat-related mortality, heat-related deaths constitute excess deaths attributable to temperatures over the "optimal" (i.e., minimum mortality) temperature. It is possible that RRac for heat-related deaths defined in this way differs from RRac during heatwaves.

4. Because the meta-analysis is based on observational studies, it is possible that the RRac estimate was distorted by confounding in some or all of the 9 studies included in the meta-analysis. That is, having household air conditioning may be associated with other characteristics that prevent heat-related mortality (e.g., good baseline health status, not living alone) for which there was no adjustment in some or all of the 9 studies.

However, although caution should be observed in claiming causality from observational studies, some observational associations are so strong and consistent, and are supported by toxicological, physiological, and/or other experimental studies (such as the association between smoking and lung cancer), that causality can, in fact, be claimed. In this case, it is likely that the strong negative association between air conditioning and heat-related mortality observed in the meta-analysis (RRac = 0.24) does represent a causal association. Most of Hill's classic criteria for causality are met, including strength of association, consistency across studies, temporality, and plausibility. Based on physiological considerations alone, it is highly likely that having access to a cool indoor environment, by virtue of air conditioning or other means, confers protection against heat-related mortality.

5. The proportion of households with air conditioning was used to estimate the proportion of the 65-and-older population having household air conditioning. This estimate did not take into account the size of households with air conditioning versus those without air conditioning or the vulnerability to heat stress of persons living in households with air conditioning versus those without air conditioning. In addition, the presence of air conditioning in a household does not guarantee the use of air conditioning in that household.

6. To estimate the number of heat-related deaths prevented by air conditioning, the finer the spatial resolution, the more accurate the estimates. The data available on proportion of the population having household air conditioning was at the country or region level. Thus, in this estimation, it was by necessity assumed that the proportion of the population having household air conditioning is homogeneous within each country/region. This assumption may not be accurate, especially for larger countries/regions.

7. The estimation of calendar-year-specific and country-specific heat-related deaths from Indicator 1.1.6, Heat-related mortality, had its own limitations, described in the indicator 1.1.6 section of this appendix. In particular, potential over-estimation of heat-related deaths in later calendar years would result in over-estimation of number of heat-related deaths averted by air conditioning in later calendar years.

Estimate of number of premature deaths (all ages) due to $PM_{2.5}$ emissions from air conditioning

Similar to estimation of the number of heat-related deaths prevented by air conditioning, to estimate the number of premature deaths due to $PM_{2.5}$ emissions from air conditioning, the finer the spatial resolution, the more accurate the estimates. Again, the data available for electricity final consumption for air conditioning was at the country or region level. Thus, in a given country/region, it was by necessity assumed that the electricity market is completely connected, so that the share of electricity used for air conditioning can be equally applied to power plant emissions throughout the country/region. This assumption may not be accurate, especially for larger countries/regions.

Notably, the sustainability of air conditioning could be increased through generation of electricity by renewable energy and more efficient air conditioning technology. These measures would reduce both CO_2 emissions and the number of premature deaths due to $PM_{2.5}$ emissions from air conditioning. Greenhouse gas emissions from air conditioning could be further reduced through phase-out of hydrochlorofluorocarbon refrigerants in favor of refrigerants that are not greenhouse gases, as called for in the 2016 Kigali Amendment to the Montreal Protocol. Technology to capture and recycle waste heat would make air conditioning even more efficient and would reduce its contribution to the urban heat island effect.

Future form of the indicator

As new studies become available, the meta-analysis of the relationship between having household air conditioning and heat-related mortality will be updated. Ideally, enough studies would become available to enable assessment of heterogeneity according to region, age, and measurement of heat exposure (e.g., heatwaves vs. departure from minimum mortality temperature). Validity of analyses would be improved through analyses with finer spatial resolution, which could be achieved through a combination of expanded collection of empirical data and modelling. In addition, city-level case studies to estimate number of lives saved from air conditioning versus premature deaths from exposure to $PM_{2.5}$ due to air conditioning may be performed. The indicator may be updated each year as new data become available on air conditioning use. Trends in country-level or finer vulnerability to heat-related mortality could be assessed with cooling degree days. Finally, metrics related to more efficient cooling (e.g., national building codes, minimum energy performance standards, labelling rules for air conditioners) and progress on implementing the Kigali Amendment may be tracked in the future. The "Future form of the indicator" section for Indicator 1.1.6, Heat-related mortality, in this Appendix discusses how examination of heat-related mortality could be improved, which would also improve the validity of Indicator 2.3.2 being discussed here.

Additional analysis

The percent of households with air conditioning in selected countries is presented in Figure 44 and CO_2 emissions from air conditioning use is presented in Figure 47, with the percent change in these two metrics from 2000 to 2019 presented in Table 20. Deaths averted by household air conditioning in the in the 65-and-older population, globally, and in selected countries are presented in Figure 45 and Figure 46.

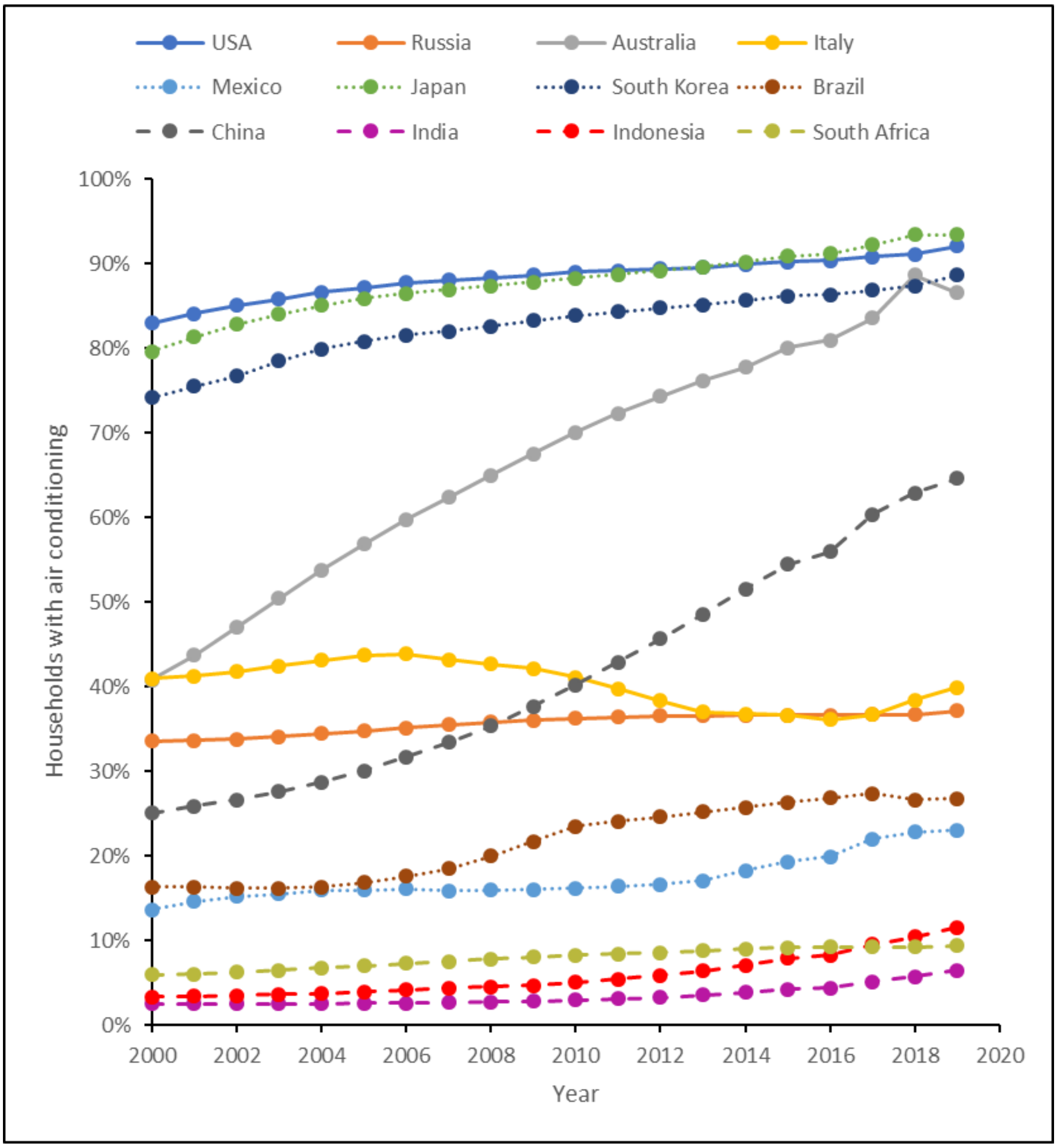

Figure 44. Percent of households with air conditioning, by selected countries, 2000-2019.

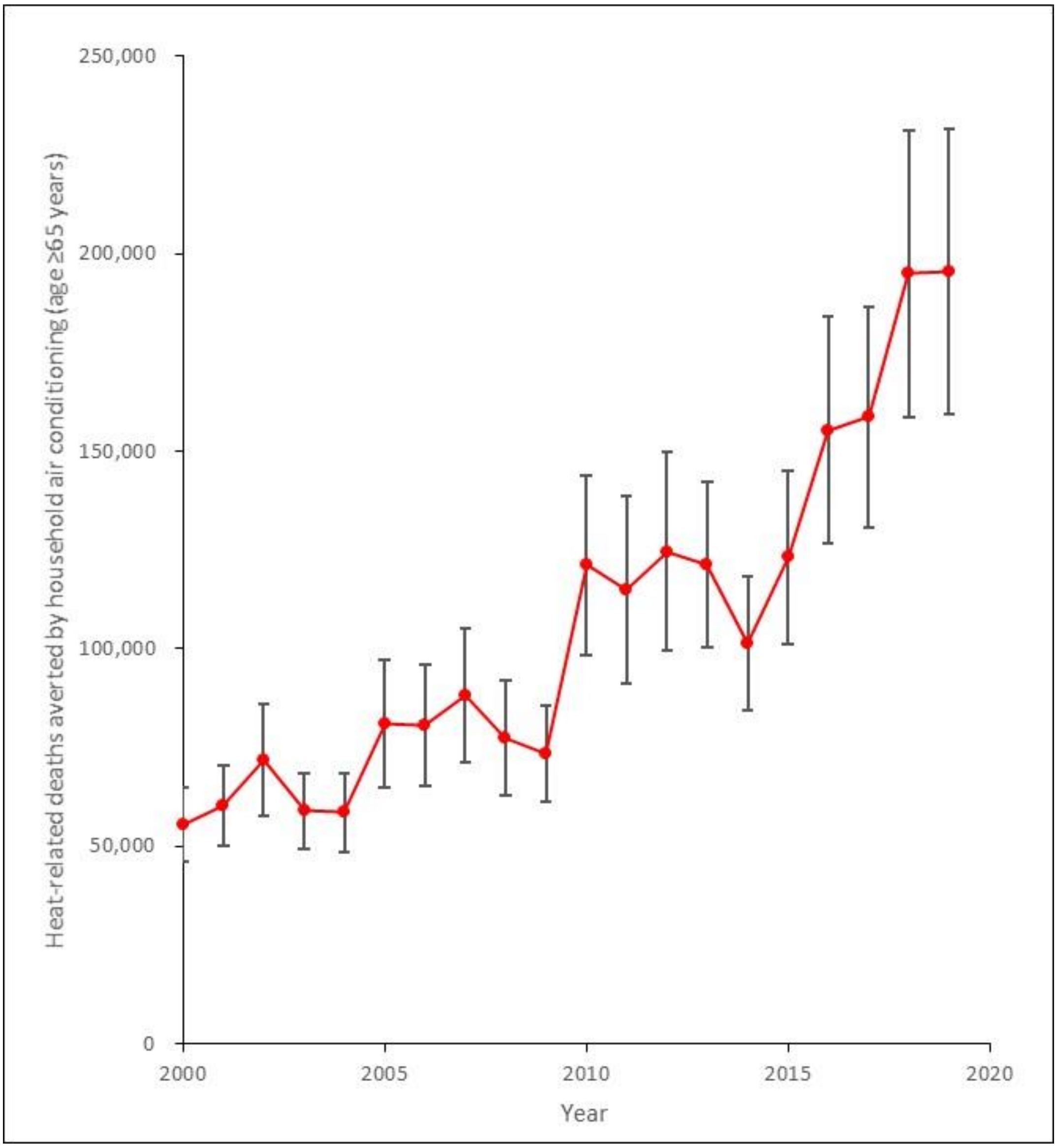

Figure 45: Global heat-related deaths averted by household air conditioning in the 65-and-older population (red line) with 95% confidence intervals (black error bars), 2000-2019.

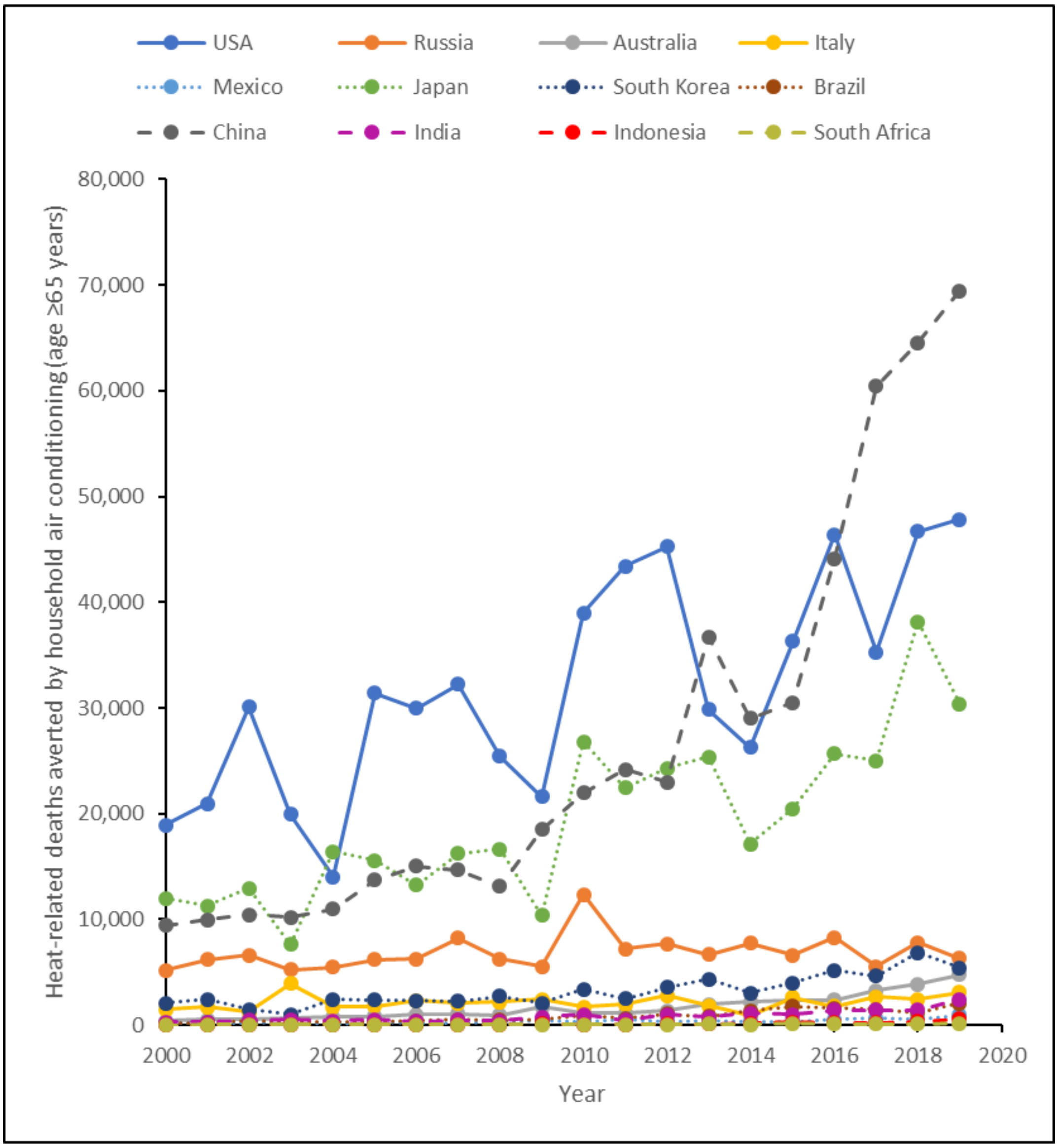

Figure 46: Heat-related deaths averted by household air conditioning in the 65-and-older population, by selected countries, 2000-2019.

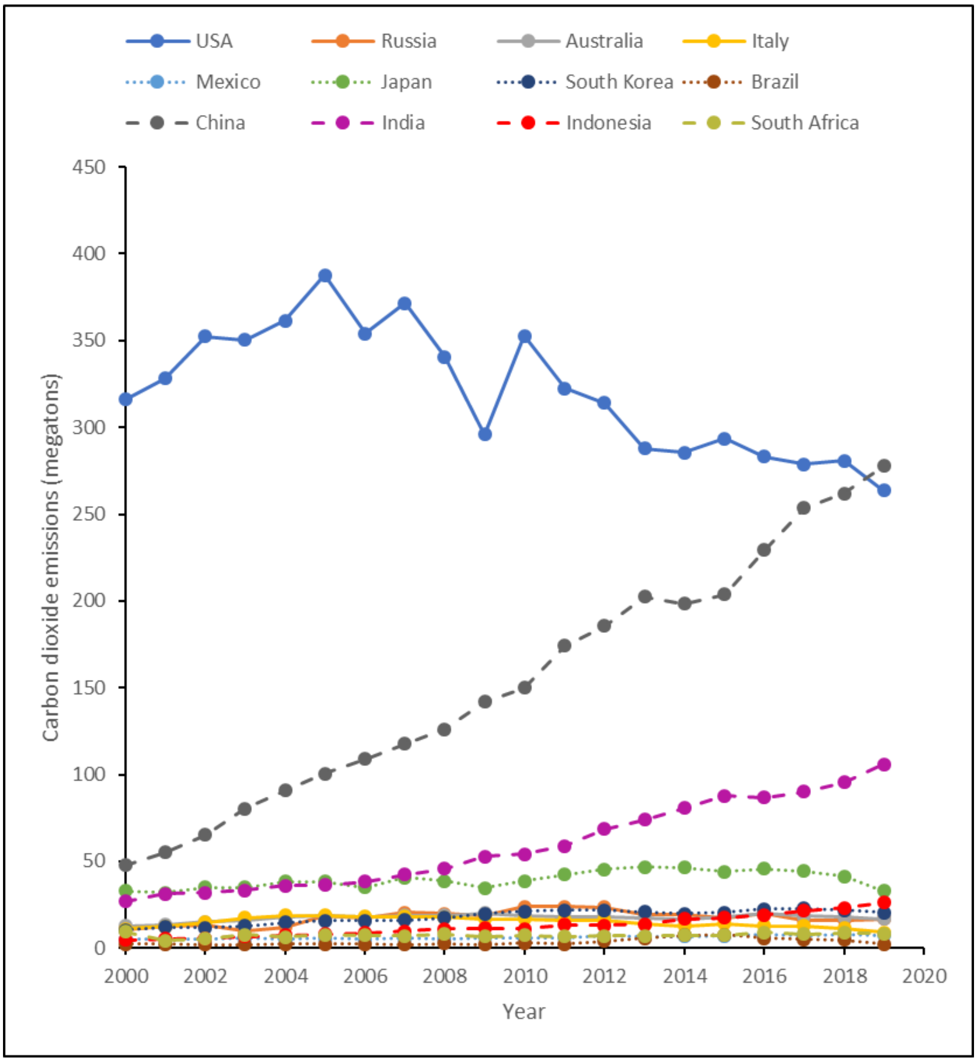

Figure 47: CO_2 emissions from air conditioning, by selected countries, 2000-2019.

Table 20: Percentage change in proportion of households with air conditioning and CO2 emissions from air conditioning by IEA region 2000-2019.

Country/region	Change in proportion of households with air conditioning (2000-2019)	Change in CO_2 emissions from air conditioning (2000-2019)
Australia	112%	33%
Brazil	64%	-1%
Canada	33%	-16%
China	158%	480%
Denmark	22%	-57%
Finland	162%	84%
France	102%	-64%
Germany	122%	-29%
Iceland	19%	-78%
India	158%	293%
Indonesia	244%	447%
Italy	-2%	-18%
Japan	17%	0%
Mexico	69%	48%
New Zealand	89%	-3%
Norway	6%	550%
Russian Federation	11%	57%
South Africa	59%	-10%
South Korea	20%	94%
Sweden	48%	-27%
United Kingdom	11%	-70%
United States	11%	-17%
Chile, Columbia	322%	200%
ASEAN Countries	126%	155%
CASPIAN Countries	522%	48%
Middle East	67%	102%
North Africa	38%	250%
Other Africa	60%	204%
Other Asia	79%	152%
Other Europe	83%	0%
Other Latin America	57%	117%
World Total	57%	61%

Indicator 2.3.3: Urban Green Space

Methods

Urban areas were defined by the Global Human Settlement (GHS) program of the European Commission, which uses remote sensing and demographic data to define more than 10,000 urban centres worldwide.[147] To choose cities for the green space indicator, 996 urban centres larger than 500,000 inhabitants were selected, plus the most populated cities of the countries that were unrepresented using the 500,000 threshold, giving a final count of 1,029 urban centres across 168 countries. A total of 27 countries were not represented in the analysis because of missing data in GHS (26 countries) and in Normalized Difference Vegetation Index (NDVI) data (1 country).

Data on population size for 2010, 2015 and 2020 were collected from the Center for International Earth Science Information Network - CIESIN - Columbia University,[148] which models the distribution of human population (counts and densities) on a continuous global raster surface with an output resolution of 30 arc-seconds (approximately 1 km at the equator).

Green space was estimated using the NDVI, the most commonly used satellite image–based vegetation index. Chlorophyll in plants absorbs red light (with wavelengths of 0.635–0.700 µm) for use in photosynthesis, whereas leaves reflect near-infrared light (0.7–1.1 µm). NDVI calculates the ratio of the difference between near-infrared radiation and visible radiation by the sum of these two measures. NDVI ranges from –1.0 to 1.0, with negative values indicating water and values closer to 1 indicating higher levels of vegetative density.[149] Publicly available data from the Landsat satellite, a joint program of the USGS and NASA was used.[150] Landsat images the entire Earth's surface at a 30-meter resolution about once every two weeks. To account for seasonal fluctuations, the following time periods for each year were used when downloading Landsat data, based on four seasons in the northern hemisphere:

- Winter – December 1 of the previous year to March 1
- Spring – March 1 to June 1
- Summer – June 1 to September 1
- Autumn – September 1 to December 1

For each year, a seasonally time-varying measure based on the NDVI for each season was created. Four exposure metrics were calculated for each year and city: peak NDVI (maximum NDVI across all seasons); annual mean based on the four-season average NDVI; population-weighted average based on peak NDVI; and population-weighted average based on annual mean NDVI (accounts for population size per raster). The population-weighted NDVI was estimated by multiplying each NDVI value (peak or four-season average) by the population size of the corresponding year within the same 1x1 km raster, summing up over the weighted values, and dividing by the sum of the weights. Google Earth Engine was used to generate raw data and R Statistical Software to compute the four metrics described above per year per city: peak NDVI, annual mean NDVI, and both population weighted averages NDVI (based on peak and four-season average) for each city. R Statistical Software was also used for data analysis and data management.

‘Level of Greenness’ was defined according to the table below:

Table 21: Categorisation of 'Level of Greenness'

Level of Greenness	Population-Weighted Peak NDVI
Very low	<0.3
Low	0.30-0.39
Moderate	0.40-0.49
High	0.50-0.59
Very High	>0.6

Data

1. Urban areas were obtained from the Global Human Settlement Programme of the European Commission;[147]
2. Population size per urban centre were the NASA GPWv4;[148]
3. Satellite data were downloaded from the publicly available Landsat satellite, a joint program of the US Geological Survey and NASA.[150]

Caveats

Some limitations of this analysis must be noted. First, although satellite-based measures of vegetation have been used extensively to measure greenness, NDVI does not provide information on the quality of greenness (curated park vs vacant lot), the type of green space (park vs forest), the type of vegetation (trees vs flowers) or social characteristics (e.g. perceived level of security). Nevertheless, a validation study demonstrated that NDVI performs adequately when compared with environmental psychologists' evaluations of green spaces.[151] Moreover, previous reviews of the literature on greenness and health have been undertaken by this group,[152,153] and found consistent and strong evidence of associations for higher greenness measured by NDVI, with improvements in birth weights and physical activity, lower mortality rates, as well as lower levels of depression. In addition, NDVI is a publicly available commonly used metric that is gathered consistently across the globe over time, which will enable comparisons across locations and between studies. Second, missing values from GHS or from Landsat data due to cloud cover or other factors limit the generalizability of the findings.

Future form of indicator

Future versions of this indicator will continue to examine trends over time. It will also aim to estimate the proportion of each city that is green space, in addition to the average greenness of a city. Finally, data from this indicator may be used to investigate associations between urban green space and reduction in heat-related mortality.

Additional analysis

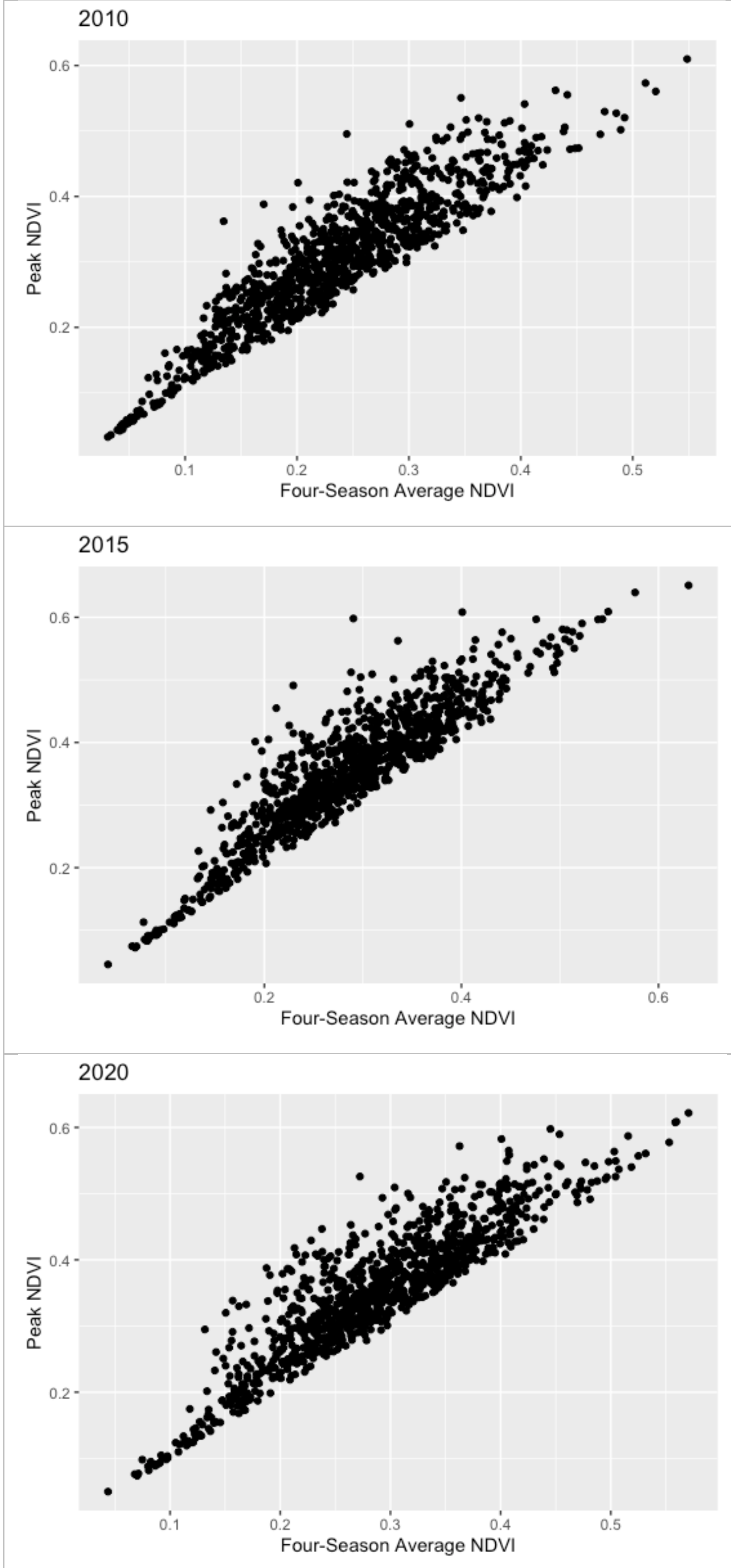

Figure 48: Scatterplot of Peak NDVI versus Annual mean NDVI by year

Table 22. Estimates of Urban Green Space by 2019 Human Development Index (HDI) in 2020

HDI	Peak NDVI	Four-season NDVI	Pop weighted Peak NDVI	Pop weighted Four-season NDVI
Low	0.31	0.25	0.27	0.22
Medium	0.38	0.32	0.33	0.28
High	0.34	0.28	0.30	0.24
Very High	0.36	0.29	0.34	0.27

Table 23. Estimates of Urban Green Space by 2019 Human Development Index (HDI) in 2015

HDI	Peak NDVI	Four-season NDVI	Pop weighted Peak NDVI	Pop weighted Four-season NDVI
Low	0.31	0.26	0.28	0.21
Medium	0.38	0.31	0.33	0.28
High	0.34	0.27	0.28	0.23
Very High	0.37	0.29	0.35	0.27

Table 24. Estimates of Urban Green Space by 2019 Human Development Index (HDI) in 2010

HDI	Peak NDVI	Four-season NDVI	Pop weighted Peak NDVI	Pop weighted Four-season NDVI
Low	0.27	0.21	0.23	0.15
Medium	0.32	0.24	0.26	0.17
High	0.29	0.22	0.23	0.17
Very High	0.33	0.26	0.30	0.21

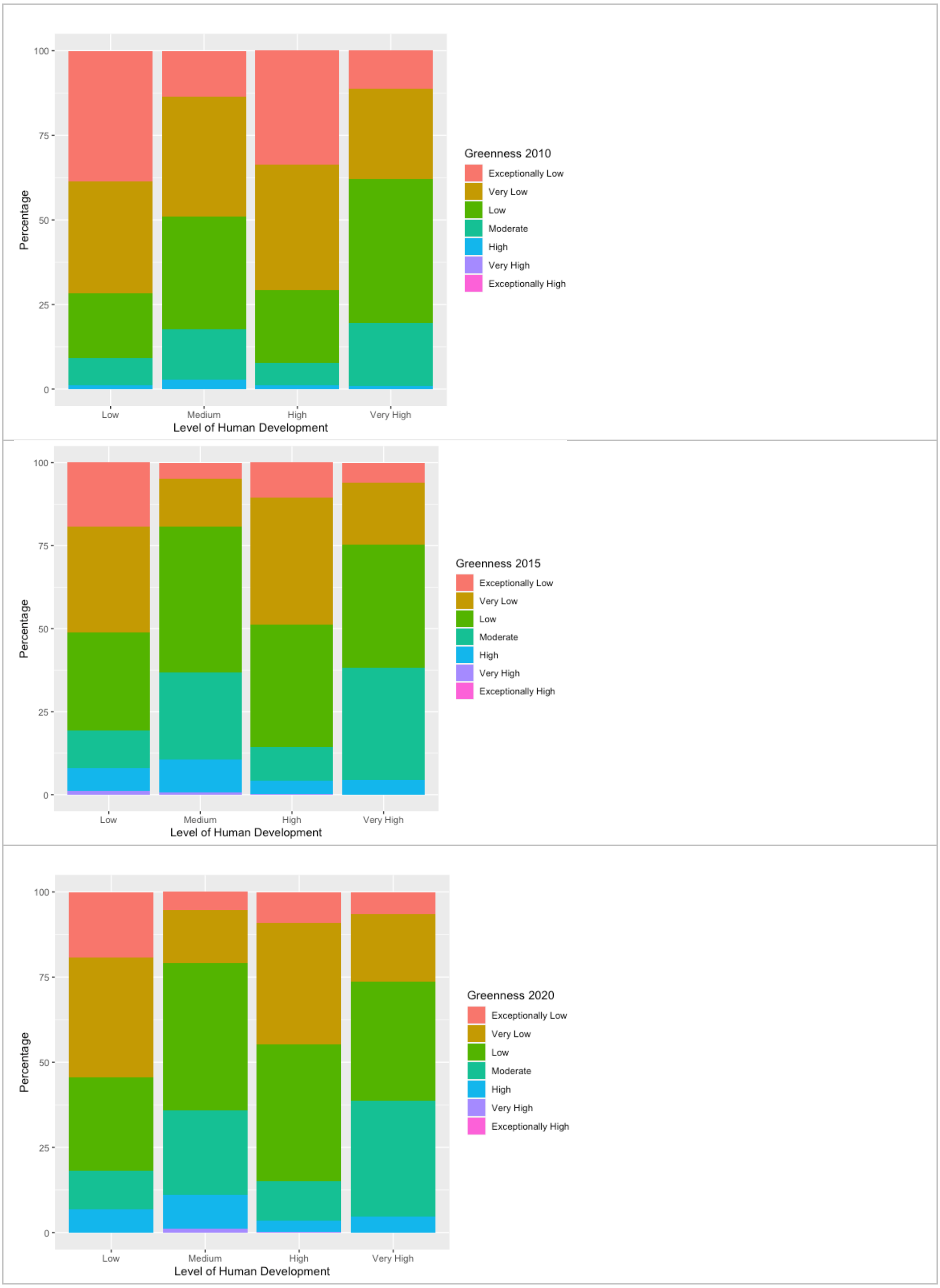

Figure 49. "Urban Greenness Levels" based on population-weighted peak NDVI by level of human development and year.

Indicator 2.4: Global Funding and Financial Transactions Related to Health Adaptation

Methods

Adaptation Funding

This indicator used the Climate Funds Update (CFU http://www.climatefundsupdate.org) data on total gross flows of all multilaterally governed funds focused on climate change from January 1 2003 to December 31 2020. For this indicator, the CFU data selected was for adaptation and for 'multiple foci', and the "approved funding" figure was used to calculate adaptation funding.

Using the CFU dataset, the following criteria were used to define adaptation funding proactively targeting health systems:

- Column G (Objective) is 'Adaptation' OR
- Column G (Objective) is 'Multiple Foci' AND
- Column H (Sector) is 'Health' OR
- Column J (Sub-Sector) is 'Health' OR
- Column N (Recipient) includes 'Health' OR
- Column N (Recipient) includes 'WHO'

It is recognised that other adaptation measures can have important health co-benefits. Drawing out how effectively adaptation projects benefitted and accounted for health is complex and developing a consistent methodology for doing so would require more resource. As a proxy-measure, the following criteria were used to define adaptation funding with potential secondary benefits for health systems:

- Column G (Objective) is 'Adaptation' OR
- Column G (Objective) is 'Multiple Foci' AND
- Column H (Sector) is 'Health' OR
- Column J (Sub-Sector) is 'Health' OR
- Column N (Recipient) includes 'Health' OR
- Column N (Recipient) includes 'WHO' OR
- Column F (Name) includes 'Health' OR
- Column K (Key Words) includes 'Health'
- Column L (Summary) includes 'Health'

Adaptation Financial Transactions

The methodology for obtaining the data for this indicator remains the same compared to the Lancet Countdown 2020 report,[1] where two significant changes were made to the analysis. To present a more cohesive full report, the data for this indicator was converted to USD. Additionally, the definition for health-related spending in non-health sectors was expanded.

The 'Adaptation and Resilience to Climate Change' (A&RCC) dataset is the same, annually updated, data source that was used in the 2017-2020 Lancet Countdown reports.[1,73,123,124] It measures spending on economic activities related to adaptation and resilience to climate change. It was developed by the data research firm kMatrix in partnership with numerous stakeholders.[154] It includes the key adaptation measure identified by the IPCC. This classification of adaptation activities was originally developed through attempts by the UK Department for Environment, Food and Rural Affairs to measure adaptation in 2009/2010. The definition of adaptation activities was extended through collaboration with the Greater London Authority in 2014 and updated through a project with Climate-KIC in 2017. This added several new industrial sectors as well as significantly expanding the activities under health and healthcare.

The methodology used for data acquisition and analysis is based on a system called as 'profiling', which was originally developed at Harvard Business School to track and analyse technical and industrial change.[155] This is the

basis for building taxonomies of economic activities and value chains, which can then be populated with estimates of key economic metrics like sales value and employment by triangulating transactional and operational business data to estimate economic values. This methodology is particularly valuable in areas where government statistics and standard industry classifications are not available. When measuring an industry or sector, the new taxonomy is populated from the bottom up, searching for evidence for the ideal definition and including only economic activities where sufficient evidence is available.

For each transaction listed in the adaptation economy data, a minimum of seven separate sources must independently record the transaction for it to be confirmed and included in the database. Triangulating data from multiple sources permits large volumes of unsorted, fragmented data of different types from different sources to be processed to arrive at more accurate estimates of transactional value that would not be possible using a single source. For the adaptation economy, data is produced to a confidence level of around 80%. Accessing and analysing multiple types of data is also key to identifying the 'purpose' behind an economic activity, which is key for accurately assigning economic activities to the adaptation dataset. Developing the new definition of adaptation and resilience to climate change involved the top-down taxonomy of the entire 'make and mend' economy, and then adaptation and resilience in all forms. Then these categories were filtered to isolate economic activities that can be strictly identified as being relevant to adaptation and resilience to climate change. The taxonomy of A&RCC is drawn from 11 sectors of the economy at-large: Agriculture & Forestry, Built Environment, Disaster Preparedness, Energy, Health/Health Care, ICT, Natural Environment, Professional Services, Transport, Waste, and Water.[156]

There are a number of activities across different sectors that are 'health-related' in the adaptation and resilience to climate change dataset, outside of the strictly defined healthcare sector. This indicator quantifies spending related to health adaptation in two categories – 1) all spend in health and healthcare sectors; 2) 'health-related' spend in other sectors.

For the 2020 Lancet Countdown report, the definition of health-related spending was developed in consultation with experts in climate change adaptation and health.[1] Health-related spending activities in non-health sectors were identified based on the following definition:

Health-related adaptation spend outside of the health sector is spend that:

1. Occurs in the following sectors: agriculture & forestry, built environment, disaster preparedness, energy, transportation, waste, or water sectors;
2. Directly impacts one or more basic determinant of health: food, water, air or shelter (these correspond closely with "physiological needs" in Maslow's Hierarchy of Needs). A broad definition of shelter is adopted, referring to social interconnectedness, domestic and public dwellings;
3. Must have an obvious and intuitive relationship to health.

Following the establishment of this definition of 'health-related' adaptation spend based on expert consultation, the complete spend dataset for a single country (the United Kingdom) for 2018/19 and two authors conducted a line-by-line assessment of each spend item (based on Level 5 of the dataset - Product or Service). The results were compared and then reviewed by a third author and then were discussed to reach agreement over any divergence as to whether or not an item should be considered as 'health-related'.

Geographical Coverage:

The A&RCC dataset has global coverage for 226 countries and territories. Data have been reported for a subset of countries and territories for whom adaptation spending data, regional and income classifications, and population estimates are available. This year's indicator covers 182 countries and territories with data reported in the A&RCC dataset, and that are assigned a region in the WHO regional classification and an HDI Classification in the UNDP's Human Development Report. Per capita values are based on 179 countries with population estimates from the IMF World Economic Outlook October 2020 update.[157]

Data

1. Adaptation Funding Dataset from Climate Fund Update.[158]
2. Adaptation and Resilience to Climate Change dataset from kMatrix Ltd, in partnership with University College London.[154]

3. Per capita values are based on 179 countries with population estimates from the IMF World Economic Outlook October 2020 update.[157]

Caveats

Due to limitations in data available, data on adaptation funding corresponds to the year that funds were approved, rather than the disbursement of funds. Consequently, it is anticipated that there can be several years of delay between approval and disbursement. Furthermore, projects under the GCF Readiness Fund are not included in the dataset applied for this analysis.

Economic activity or transactions are only measured where there is an economic 'footprint', i.e. where there is transactional/financial data available to be measured. Therefore, public sector spending without an economic 'footprint' (government spending on salaries, for example), cannot be measured. It also not possible to directly identify what percentage of measured spending is public versus private. Values are not currently adjusted for inflation. Values of sales generated are not directly comparable with values derived from national statistics.

The reference period is the financial years 2015/16 to 2019/20.

As the data is collected for Financial Years (ending in March), it is unlikely that the 2019/20 data for the 2021 report will show any impact of COVID-19.

Future form of the indicator

Further historical data could be available in the future.

Additional analysis

At the country level, estimated spend per capita varies between $17.93 (Luxembourg) and just $0.10 (Timor-Leste) for health adaptation, and between $86.75 and $0.52 (same countries) for health-related adaptation spending. As a share of all adaptation spending, health adaptation spending varies between 6.18% (United Kingdom) and 3.24% (Singapore), and between 30.1% (Haiti) and 20.81% (Singapore) for health-related adaptation spending.

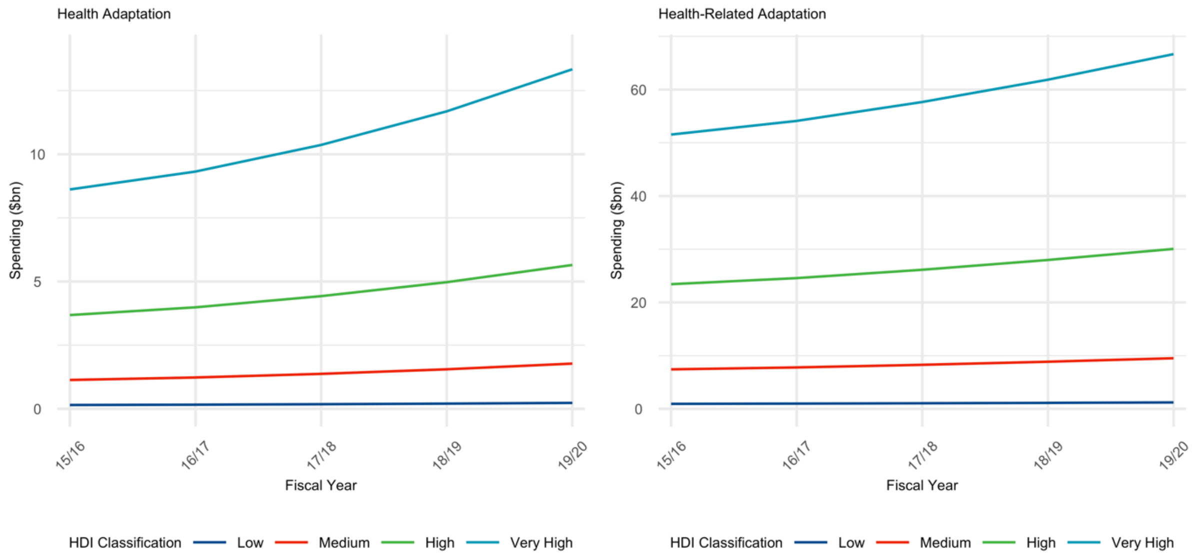

Figure 50. Health and health-related adaptation spending for financial years 2015/16 to 2019/20, grouped by HDI classification

Section 3: Mitigation Actions and Health Co-Benefits
Chapter notes

In the introductory paragraph, data from Le Quéré et al[159] was used to report CO_2 emissions drops during the COVID-19 lockdowns in 2020. These were reported by Le Quéré et al[159] as a function of World Bank Income grouping. Since the countries categorised as 'high income' do not correspond exactly to those with very high HDI, the data were reaggregated by HDI level. The median value CO_2 reductions during 2020, compared to 2019, was found to be 9.7% among countries with very high HDI. This compared a value 10.2% reported for high income countries – and therefore appears as the same value rounded to the nearest percentage.

3.1: Energy System and Health

Indicator 3.1.1: Carbon Intensity of the Energy System

Methods

This indicator contains two components:

Carbon intensity of the energy system, both at global and regional scales, (1971-2018), in tCO_2/TJ; and

Global CO_2 emissions from energy combustion by fuel, in $GtCO_2$ (1972-2018). Global emissions without fuel breakdown are also provided for 2019 and provisionally for 2020.

The technical definition is the tonnes of CO_2 emitted for each unit (TJ) of primary energy supplied.

The rationale for the indicator choice is that carbon intensity of the energy system will provide information on the level of fossil fuel use, which has associated air pollution impacts. Higher intensity values indicate a more fossil dominated system, and one that is likely to have a higher coal share. As countries pursue climate mitigation goals, the carbon intensity is likely to reduce with benefits for air pollution.

The indicator is calculated based on total CO_2 emissions from fossil fuel combustion divided by Total Energy Supply (TES). TES reflects the total amount of primary energy used in a specific country, accounting for the flow of energy imports and exports.

The data is available for most countries of the world, for the period 1971-2018.

Data

1. This indicator is based on based on the IEA dataset, CO_2 Emissions From Fuel Combustion: CO_2 Indicators, accessed via the UK data service,[160] and supplemented with additional data for 2018, 2019 and 2020.[161-163]

Caveats

IEA data are generated using both direct input from national governments and modelling. As such, while they represent the best available data on national CO_2 emissions from fuel, they are subject to caveats which vary by energy commodity and country. Full details are given in the CO_2 Emissions From Fuel Combustion documentation.[164]

Additional analysis

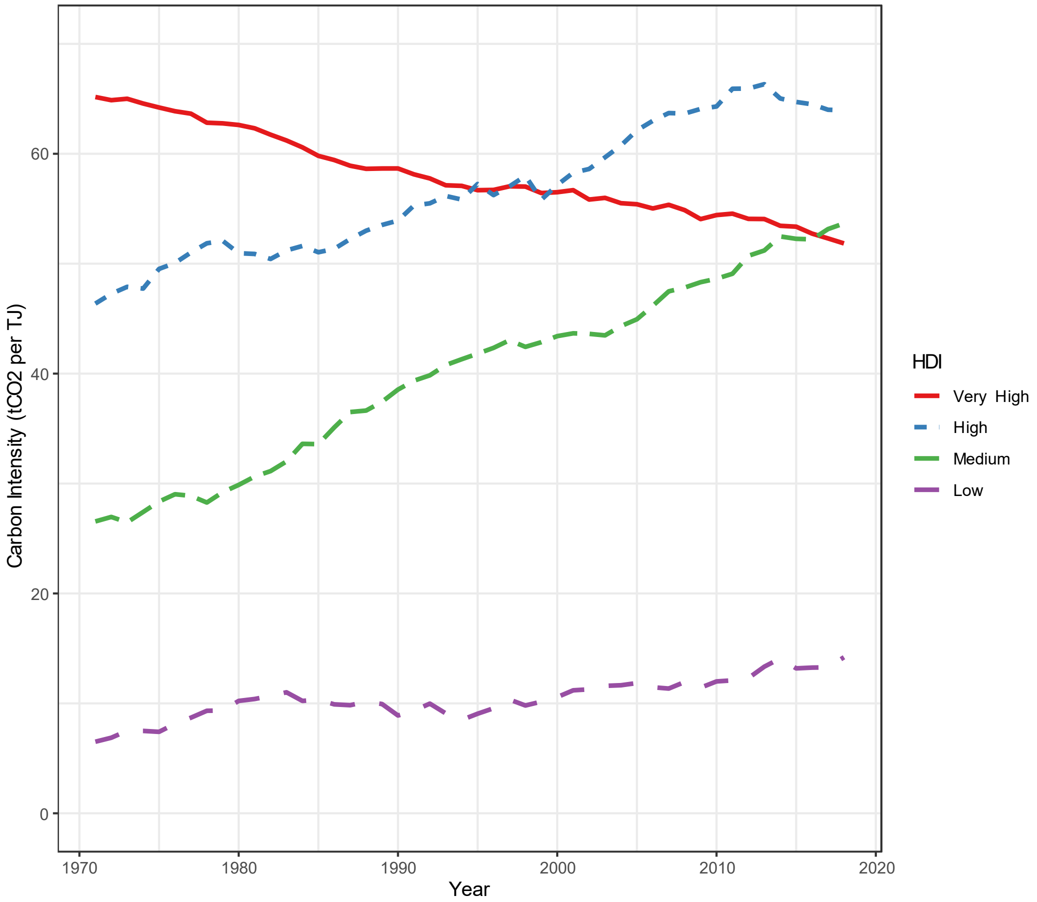

Figure 51: The carbon intensity of the energy system for 1970-2018, by 2019 Human Development Index country group

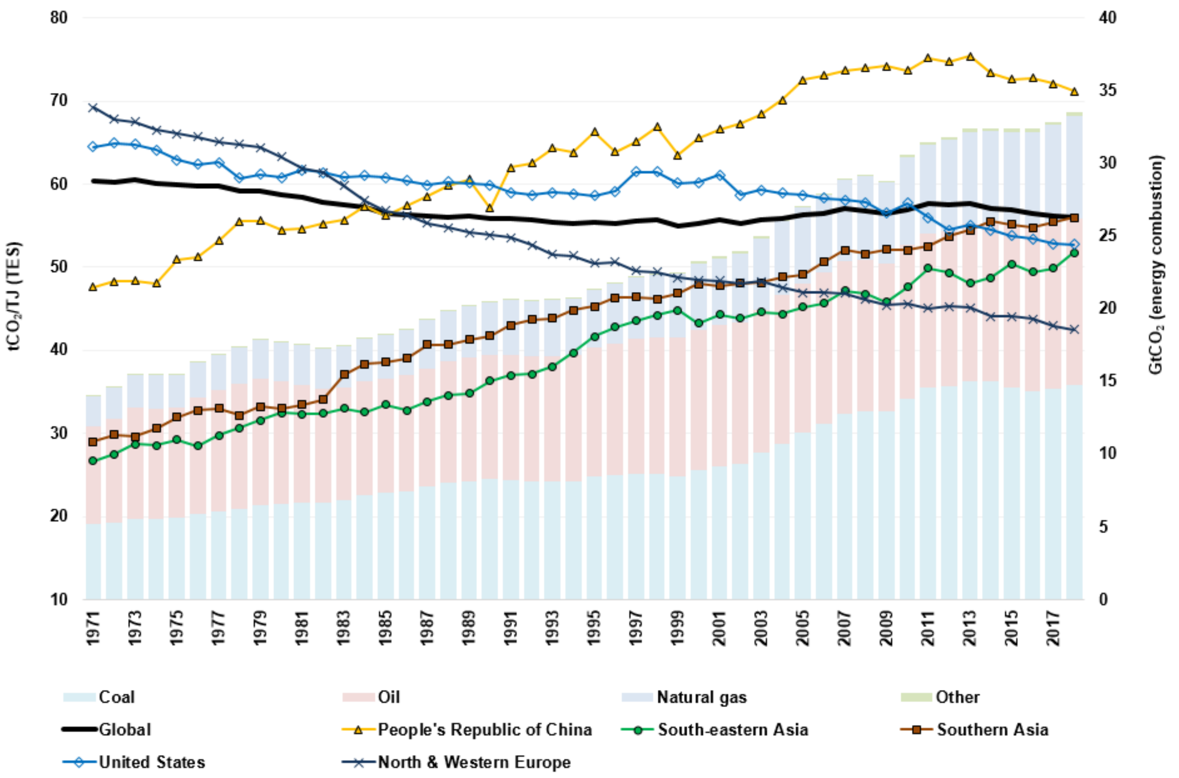

Figure 52. Carbon intensity of the energy system of regions, and total CO_2 production through energy consumption

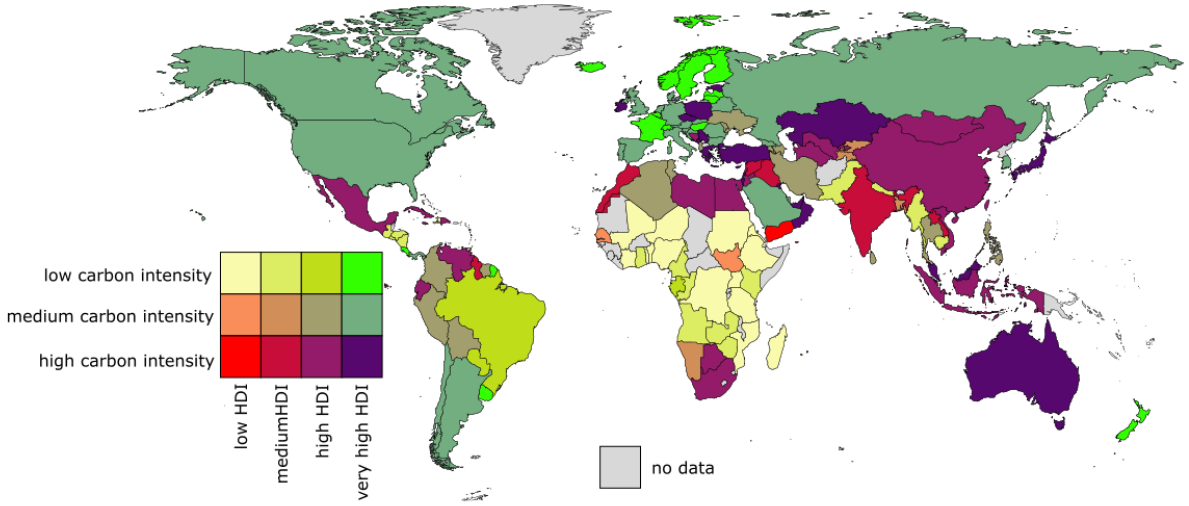

Figure 53. Carbon intensity in 2018 and HDI level per country

Indicator 3.1.2: Coal Phase-Out

Methods

Two indicators are used here:

1. Total primary coal supply by region / country (in exajoules, EJ);
2. Total and share of electricity generation from coal (% of total generation from coal) and global generation from coal (in TWh).

These indicators are important to enable tracking of changes in coal consumption at a regional and country level. Due to the level of coal used for power generation, a second indicator tracks the contribution to electricity generation from coal power plants in selected countries. As countries pursue climate mitigation goals, the use of coal is likely to reduce with resulting benefits for air pollution.

The indicator on primary energy coal supply is an aggregation of all coal types used across all sectors (from the IEA energy balances). The data are available for most countries of the world, for the period 1978-2019.

The indicator on the share of electricity generation from coal is estimated based on electricity generated from coal plant as a percentage of total electricity generated. Regional data are available from 1990-2019; pre-1990 data are not used due to incomplete time series.

Countries or regions with large levels of coal use (as a share of generation, or in absolute terms), have been selected to show in the figures.

The following types of coal are added to produce the total primary coal supply:

'Anthracite', 'Coking coal', 'Lignite', 'Other bituminous coal', 'sub-bituminous coal'

Data

1. This indicator is based on the extended energy balances from the International Energy Agency. The specific dataset is called World Extended Energy Balances (for 2020), and is sourced via the UK data service.[165]

Caveats

IEA data are generated using both direct input from national governments and modelling. As such, they are subject to caveats which vary by energy commodity and country. Full details are given in the IEA World Energy Balances documentation.[166] This documentation also covers changes to methodology in previous editions of IEA World Energy Balances. A typical example of the way data can be impacted by methodology updates by reporting countries is as follows, relating to Belgium 'New data on consumption cause breaks in time series for primary solid biofuels between 2011 and 2012'. However, since data are aggregated here by HDI level, the impacts on overall trends is minimal.

Additional analysis

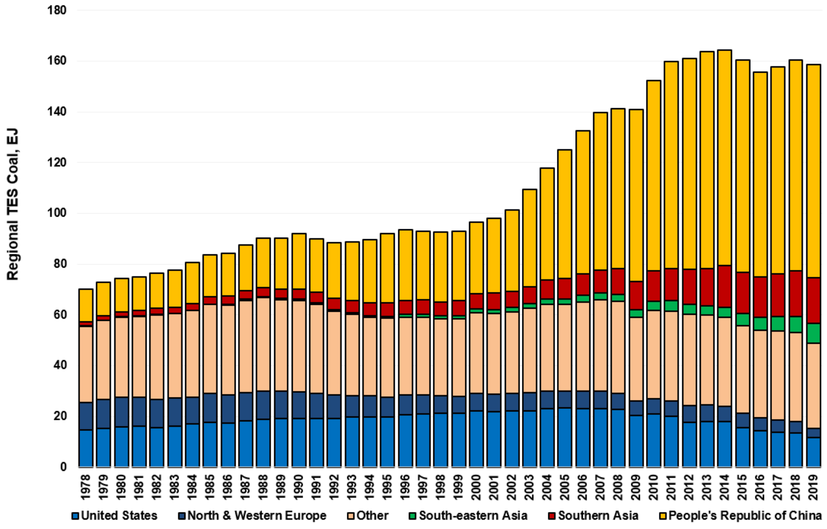

Figure 54. Total Energy Supply from coal in selected countries and regions, 1978-2019. Regional primary energy supply of coal is shown in bars of Exajoules

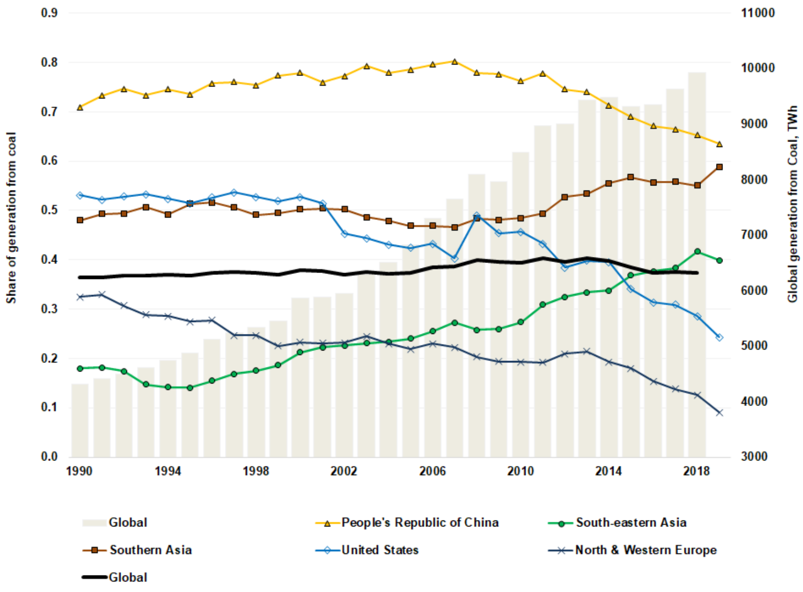

Figure 55: Share of electricity generated from coal (left axis, line graphs) and total global electricity generated from coal (right axis, bar chart)

Indicator 3.1.3: Zero-Carbon Emission Electricity

Methods

Two indicators are used here, and presented in two ways:

1. Total low carbon electricity generation, in absolute terms (TWh) and as a % share of total electricity generated (to include nuclear, and all renewables); and
2. Total renewable generation (wind and solar), in TWh, and as a % share of total electricity generated.

The increase in the use of low carbon and renewable energy for electricity generation will push other fossil fuels, such as coal, out of the mix over time, resulting in an improvement in air quality, with benefits to health.

The renewables (wind and solar) indicator has been used to allow for the tracking of rapidly emergent renewable technologies. For both indicators, generation, rather than capacity, has been chosen as a metric as the electricity generated from these technologies is what actually displaces fossil-based generation. Countries with large levels of low carbon generation (as shares, or in absolute terms), or with higher fossil dependency, have been selected.

The data are again taken from the IEA extended energy balances.[165] The absolute level indicators are total gross electricity generated aggregated from the relevant technology types. The share indicators are estimated as the low carbon or renewable generation as a % of total generation.

The data are available for most countries of the world, for the period 1971-2019. Only the period from 1990 has been used, due to data gaps for selected countries prior to 1990.

The following IEA variable names are added to produce total low carbon electricity generation:

'Nuclear, 'Hydro', 'Geothermal', 'Solar photovoltaics', 'Solar thermal', 'Tide, wave and ocean', 'Wind'

The following IEA variable names are added to produce total renewable electricity generation:

'Geothermal', 'Solar photovoltaics', 'Solar thermal', 'Tide, wave and ocean', 'Wind'

Data

1. This indicator is based on the extended energy balances from the International Energy Agency. The specific dataset is called World Extended Energy Balances, and is sourced via the UK data service (http://stats.ukdataservice.ac.uk/).[165]

Caveats

IEA data are generated using both direct input from national governments and modelling. As such, they are subject to caveats which vary by energy commodity and country. Full details are given in the IEA World Energy Balances documentation.[166] This documentation also covers changes to methodology in previous editions of IEA World Energy Balances. A typical example of the way data can be impacted by methodology updates by reporting countries is as follows, relating to Belgium 'New data on consumption cause breaks in time series for primary solid biofuels between 2011 and 2012'. However, since data are aggregated here by HDI level, the impacts on overall trends is minimal.

Additional analysis

It should be noted that a number of countries in 2019 had more than a quarter of their electricity coming from renewables (excluding hydro) including Costa Rica (30%), Denmark (58%), Portugal (30%), El Salvador (32%), Germany (28%), Iceland (31%), Ireland, (31%), Lithuania (47%), Luxembourg (38%), Spain (26%) and Uruguay (32%).

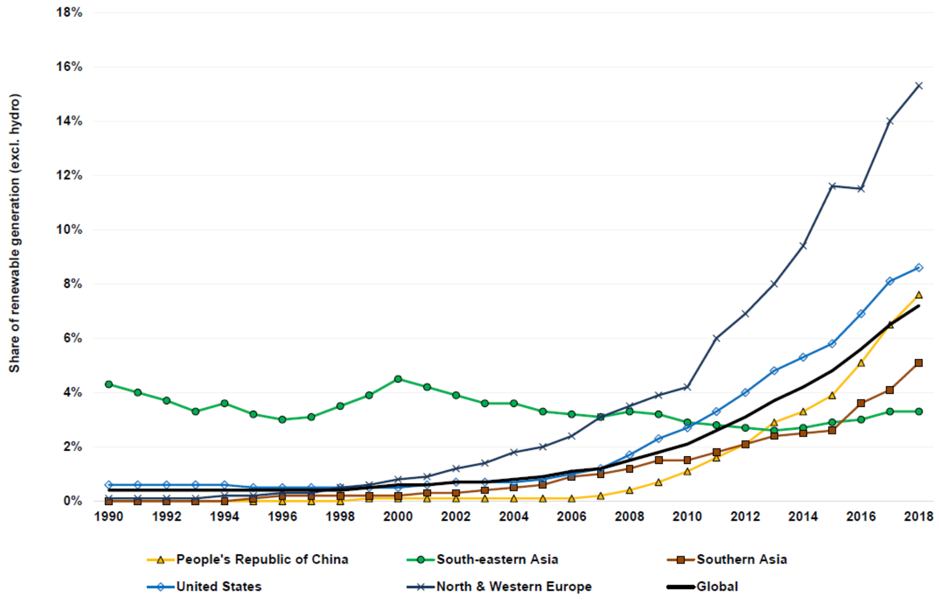

Figure 56. Share of electricity generation provided by modern renewables (wind, solar and geothermal) by IEA region

Indicator 3.2: Clean Household Energy

Methods

The 2021 report presents a combination of data from the WHO which feeds into the Sustainable Development Goal 7 and fuel consumption in the residential sector produced by the IEA.

Access to clean energy is defined by the IEA (2020) as:

"a household having reliable and affordable access to both clean cooking facilities and to electricity, which is enough to supply a basic bundle of energy services initially, and then an increasing level of electricity over time to reach the regional average".[167]

Within SDG 7.1.2 (proportion of population with primary reliance on clean fuels and technology) "Clean" fuels are defined by emission rate targets and specific fuel recommendations included in the WHO guidelines for indoor air quality: household fuel combustion.[168]

This indicator is modelled with household survey data compiled by WHO,[169] which uses Bayesian methods to impute yearly estimates of primary reliance on clean fuels and technologies for cooking, heating and lighting, using survey-based estimates between 2000 and 2017 and modelling projections for 2018 and 2019.[170,171] The data were aggregated to HDI grouping by multiplying the percentage access rates for a given country by the UNDP estimates for rural/urban population[172] and then summing by HDI group and dividing by the overall rural/urban/total population for these HDI groups.

The use of energy in the residential sector is drawn from the IEA extended global residential modelling produced in the World Energy Outlook from the 'World Extended Energy Balances' 2020 edition, which covers all countries or major regions in the world.[173] The values are measured in EJ and cover all fuels supplied for consumption within the residential sector (IEA flow code QGFLOW076) final energy demand.

The specific IEA variables were combined in the following way:

`Solid biofuels` = Charcoal + `Primary solid biofuels`

`Coal, coke and peat` = `Hard coal (if no detail)` + BKB + `Petroleum coke` + `Patent fuel`+`Coke oven coke`+`Brown coal (if no detail)`+Peat+`Gas coke`+`Peat products`+`Coking coal`+`Sub-bituminous coal`+`Other bituminous coal`+Lignite+Anthracite+Bitumen

`Other biofuels` = `Other liquid biofuels` + Biogasoline + `Non-specified primary biofuels and waste`+`Biogases`+`Biodiesels`

`Liquid fossil fuels` =`Paraffin waxes`+`Other oil products`+`Naphtha`+ `Gas/diesel oil excl. biofuels` + Lubricants + `Natural gas liquids`+ `Other kerosene`+`Liquefied petroleum gases (LPG)`+`Fuel oil`+`Motor gasoline excl. biofuels`+`Crude oil`

`Waste & other` = `Municipal waste (non-renewable)`+`Municipal waste (renewable)`+`Industrial waste`+`Refinery gas`+ `Blast furnace gas`+`Gas works gas`+`Coke oven gas`+`Oil shale and oil sands`,

Finally, Natural gas, Heat, Solar thermal, Geothermal and Electricity variables were provided directly from IEA flow QGFLOW076.

The indoor $PM_{2.5}$ concentration estimates use data from WHO, which uses national surveys to estimate the proportions of rural households using clean and different forms of solid fuels for cooking in 2018.[174] For each polluting fuel type, the proportion of rural households using traditional or improved cookstoves was obtained from the GAINS model (see Indicator 3.3 for full details of GAINS). These were then combined to estimate the frequency of different fuel and stove combinations for rural households in a selection of countries. Modelled annual average 24 hour kitchen concentrations[175] for the different fuel and stove types were then weighted by these frequencies in order to estimate national average rural indoor concentration estimates. Household air pollution-attributable mortality estimates were obtained from the GBD for 2019 for the same selection of countries for men and women.

Data

1. Healthy fuels for cooking were provided by the WHO.[169-171]
2. The additional energy usage and access is based on data from the IEA World Energy Balances 2020.[176]
3. Demographic data from the United Nation World Population Prospects (UN WPP).[177]
4. % Urban population from World Urbanization Prospects: 2018 Revision.[172]
5. Integrated exposure-response (IER) functions and mortality rates were taken from the 2019 GBD Project.[178]

Caveats

The data from the IEA on residential energy flows and energy access provide an indication of both the access to electricity and the proportion of the different types of energy used within the residential sector. These provide an important picture on how access and use might be interacting.

IEA data are generated using both direct input from national governments and modelling. As such, they are subject to caveats which vary by energy commodity and country. Full details are given in the IEA World Energy Balances documentation.[166] This documentation also covers changes to methodology in previous editions of IEA World Energy Balances. A typical example of the way data can be impacted by methodology updates by reporting countries is as follows, relating to Belgium 'New data on consumption cause breaks in time series for primary solid biofuels between 2011 and 2012'. However, since data are aggregated here by HDI level, the impacts on overall trends is minimal.

Future form of the indicator

Future development of the health impacts fuel use in the home is expected for the 2022 report.

Additional analysis

The latest data for indicator 3.2 are available up to 2018. In low HDI countries, the share of domestic energy provided by electricity has remained static at around 1.8% for the 5 years up to 2018. The use of liquid fossil fuels fell very slightly from 1.73% in 2010 to 1.15% in 2018. Total per capita domestic energy use fell by 3.5% in the same period. Primary reliance on clean fuels for cooking grew slightly from 8% in 2010 to 10% of the population in 2018. Taken together these figures suggest progress was already too slow prior to the pandemic and has since been reversed.

In medium and high HDI countries, progress since 2010 has been better. Share of solid biofuel use in homes fell from 77% to 68% between 2010 and 2018 in medium HDI countries, and by 36% to 21% in high HDI countries in the same period. Electricity share grew from 8% to 14% in low HDI countries, and 18% to 25% in medium HDI countries. There was also growth in primary reliance on clean fuels for cooking (medium HDI: 35% to 47%, high HDI: 64% to 75%).

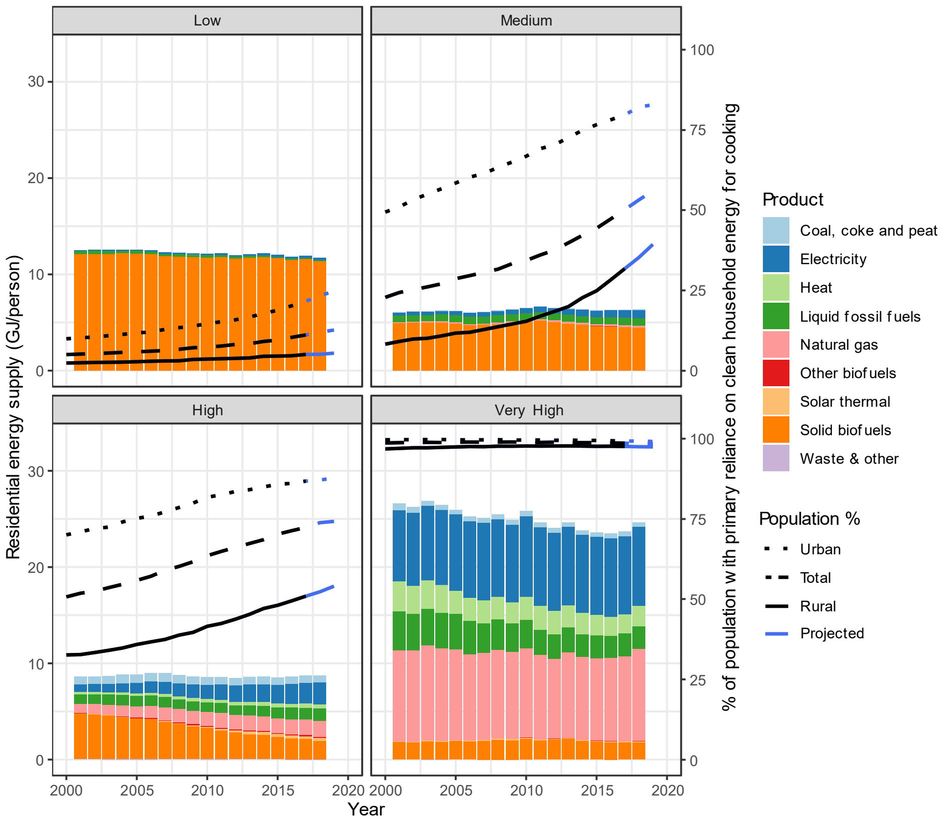

Figure 57: Residential energy supply by 2019 Human Development Index country group for 2000 to 2019. Primary axis: per capita fuel type (coloured bars). Secondary axis: percentage of population with primary reliance on clean fuels and technology for cooking. Data kindly provided by the WHO and the IEA[180-182]

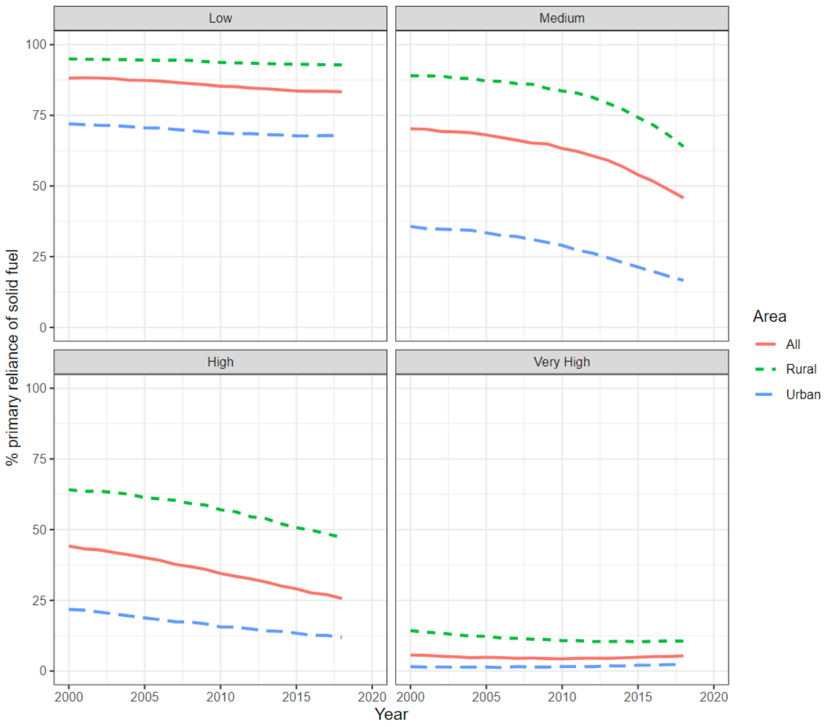

Figure 58. Primary reliance of solid fuel for cooking by HDI grouping11. Data do not include all 'High Income' countries

Figure 58 and Figure 57 give the survey/imputed aggregated data for the percentage of households who rely on solid fuel for cooking – for which solid fuel is defined as “charcoal, coal, crop residues, dung, or wood”. [170] The following countries comprise the very high HDI countries included: Argentina, Austria, Belarus, Chile, Costa Rica, Croatia, Czechia, Estonia, Georgia, Greece, Kazakhstan, Republic of Korea, Latvia, Malaysia, Mauritius, Montenegro, Oman, Panama, Qatar, Romania, Russia, Saudi Arabia, Serbia, Slovakia, Slovenia, Spain, Turkey, United Arab Emirates, and Uruguay. Other HDI groupings are well represented.

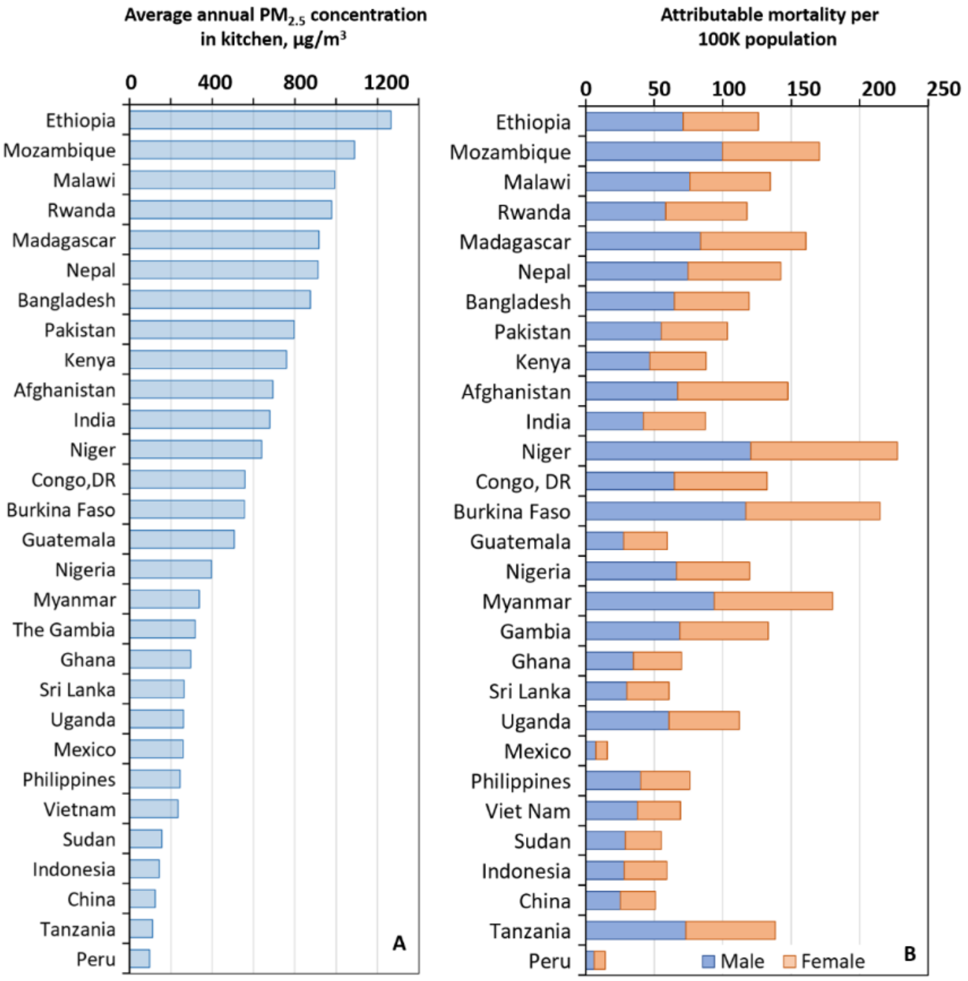

Figure 59. Household air pollution in select countries. A) Estimated average concentration of $PM_{2.5}$ from fuel combustion in the main indoor cooking area in rural regions, B) Estimated national disease burden attributable to exposure to $PM_{2.5}$ from household cooking fuels from the GBD 2019.[11,179]

Indicator 3.3: Mortality from Ambient Air Pollution by Sector

Methods

This indicator quantifies contributions of individual source sectors to ambient $PM_{2.5}$ exposure and its health impacts. Contributions from coal have been highlighted across all sectors.

Estimates of sectoral source contributions to annual mean exposure to ambient $PM_{2.5}$ were calculated using the GAINS model,[180] which combines bottom-up emission calculations with atmospheric chemistry and dispersion coefficients.

Energy statistics are taken from the IEA World Energy Statistics for 2015, from the IEA World Energy Outlook 2019[181] for 2018, and World Energy Outlook 2020[182] for 2019. Data on energy consumption in individual sectors are imported into GAINS, matching the sectors of the World Energy Statistics and downscaling to the 180 GAINS global regions. They are then merged with GAINS information on application of emission control technologies in each region and their emission factors to calculate emissions of $PM_{2.5}$ and its precursor gases SO_2, NOx, NH_3, and non-methane volatile organic compounds (VOC).

Ambient $PM_{2.5}$ concentrations are calculated from the region and sector specific emissions by applying atmospheric transfer coefficients, which are a linear approximation of full chemistry-transport models. Atmospheric transfer coefficients in GAINS are based on full year perturbation simulations with the EMEP Chemistry Transport Model[183] at 0.1°×0.1° resolution (for low-level sources) / 0.5°×0.5° resolution (for all other sources) using meteorology of 2015. In Europe, the resolution is slightly different but the principle is the same. Calculations for Europe are described in detail by Kiesewetter et al. (2015),[184] calculations for the rest of the world are described by Amann et al.[185] Calculated ambient $PM_{2.5}$ concentrations have been validated against in-situ observations from the latest version of the WHO's Urban Ambient Air Pollution Database (2018 update)[186] and other sources where available (e.g. Chinese statistical yearbook) and show in general good agreement with monitoring data up to urban background level (local variation at roadside stations is not captured by the resolution of a few kilometres).

Deaths from total ambient $PM_{2.5}$ for regions other than Europe are calculated following the methodology of the GBD studies. Exposure-response relationships have been updated for this report to be consistent with the GBD 2019 study.[103] The MR-BRT curves were obtained from the public release site[187] and relative risks for five diseases: ischaemic heart disease; chronic obstructive pulmonary disease; stroke; lung cancer; and acute lower respiratory tract infection calculated from them. 1000 draws of the MR-BRT curve for each disease and age group (where age specific) were used and were scaled to have RR=1 at the theoretical minimum-risk exposure level (taken from 1000 corresponding draws, average 4.15μgm^{-3}). Exposure levels below the TMREL level are assigned RR=1.

The update to the GBD 2019 exposure-response relationships resulted in a significant increase in attributable mortality beyond the numbers published in the previous editions of the Lancet Countdown, which were based on the IER relationships developed within the GBD 2013 study.[188]

Disease and age specific baseline mortality rates are taken from the GBD Results database[91] The shares of different diseases were applied to age-specific total deaths taken from UN World Population Prospects (2017 update);[177] for 2018 and 2019, the statistics were interpolated linearly between 2015 and 2020.

For Europe, this indicator follows the WHO Europe methodology and apply Exposure-response relationships for all-cause mortality among population over 30 years of age as reported under the REVIHAAP assessment.[189] (WHO, 2013). Details are described in Kiesewetter et al. (2015).[184]

Attribution of estimated deaths from ambient air pollution to polluting sectors was done proportional to the contributions of individual sectors to population-weighted mean $PM_{2.5}$ in each country.

Data

1. IEA World Energy Outlook 2019 (for the year 2018) and World Energy Outlook 2020 (for the year 2019)[190]
2. WHO's Urban Ambient Air Pollution Database (2018 update)[186]
3. UN World Population Prospects (2017 update)[177]

4. Global Burden of Disease 2019 study,[103] MR-BRT curves obtained from the public release site[187]

Caveats

The indicator relies on model calculations which are inherently uncertain. The resolution of approximately 7 to 10 km is deemed appropriate for urban background levels of $PM_{2.5}$ but may underestimate exposure in case of strong local $PM_{2.5}$ increments. The meteorology year is fixed to 2015.

Uncertainty in the shape of IER relationships make the quantification of health burden inherently uncertain.

Different dose-response relationships are used for Europe (REVIHAAP, recommended by WHO-Europe) and Asia (WHO-Global).

The non-linearity of the IERs used for non-European countries complicates the translation between the mortality burden attributed to an individual source, which is calculated proportional to the source contribution to ambient $PM_{2.5}$, and the effect of mitigating this source. While a reduction of emissions would lead to a (roughly) proportional reduction of ambient $PM_{2.5}$, this would not necessarily result in a proportional reduction of the health burden. In highly polluted environments, the health benefits of a marginal reduction of emissions would be disproportionately smaller than the relative change in concentrations.

Indicator 3.4: Sustainable and Healthy Transport

Methods

Fuel use data (by fuel type) from the IEA World Extended Energy Balances are divided by corresponding population statistics from the UN Department of Economic and Social Affairs, Population Division.

The fuel flows from the IEA are combined in the following way:

Biofuels = Biodiesels +Biogasoline +Biogases + Other liquid biofuels

Fossil fuels = Natural gas liquids+Natural gas+Motor gasoline excl. biofuels+Liquefied petroleum gases (LPG)+Refinery gas+White spirit & SBP+Kerosene type jet fuel excl. biofuels+Gas/diesel oil excl. biofuels+Lubricants+Naphtha+Fuel oil+Other kerosene+Other oil products+Bitumen

Electricity is given by the existing IEA total.

Totals for a given year and country are then divided by the corresponding country population, and then summed to produce the final estimate. This avoids including the population of the countries that are not covered by the IEA.

Data

1. Fuel use data is from the IEA, World Extended Energy Balances [191]
2. UN Population estimates, 2019 edition [192]

Caveats

This indicator captures change in total fuel use and type of fuel use for transport, but it does not capture shifts in modes of transport used. In particular, it does not capture walking and cycling for short trips, which can yield substantial health benefits through increased physical activity.[193]

Future form of the indicator

An ideal fuel use indicator would capture the direct health impacts of the use of transport fuels, with country- and urban-level specificity within the global coverage. In turn, the co-benefits of transitioning to less-polluting fuels would be quantified directly in terms of reduced exposures to air pollution and their corresponding health impact.

To capture sustainable uptake more fully, a future indicator could collate information on the proportion of total distance travelled by different modes of transport based on comprehensive local survey data. Other data on sustainable travel infrastructure, for instance the presence of cycle schemes, would also be useful.

Additional analysis

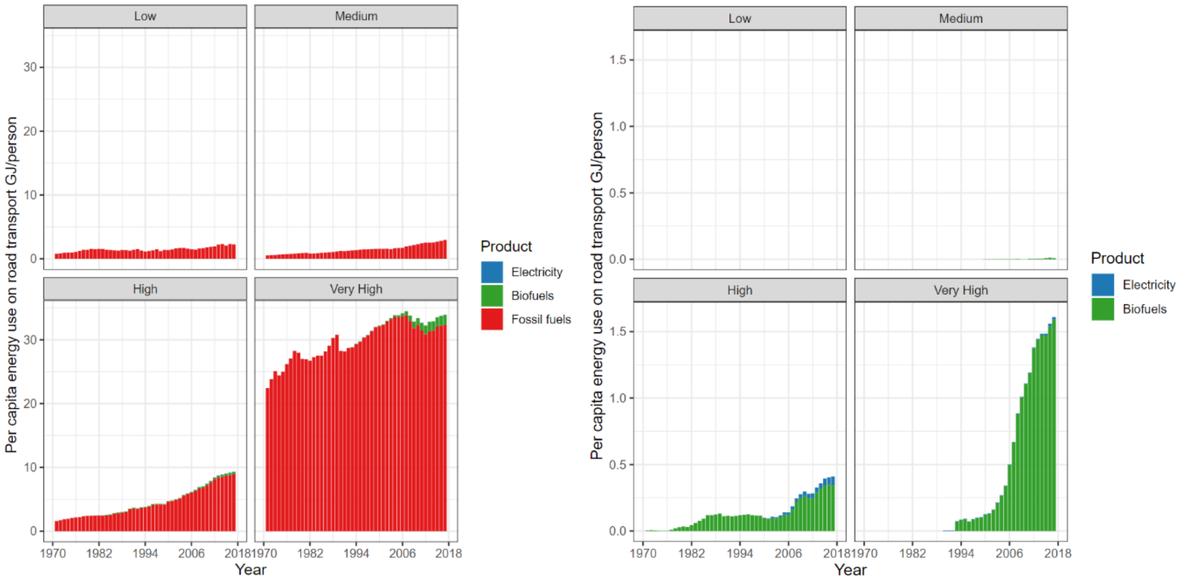

Figure 60. Per capita energy use on road transport, by fuel type and HDI. The right panel shows the data with fossil fuels excluded.

3.5: Food, Agriculture, and Health

Indicator 3.5.1: Emissions from Agricultural Production and Consumption

Methods

The method of this indicator has been improved since the 2020 edition to consider the gradual changes in GHG emissions intensity of agri-food production over time. These changes are described below. For production-related emissions, the overall method calculates emissions for each commodity, country and year using the GHG emissions intensity of production (tonnes CO_2e per tonne of commodity produced) for that commodity multiplied by the amount produced (tonnes). For consumption values, the GHG emissions associated with consumption (supply) of a commodity after trade balances are resolved for the given country and year. The details of these approaches, including data sources and assumptions, are given below.

Livestock products

Emissions intensities for the year 2000 are calculated in the following manner. The following livestock are included:

Ruminant	Non-Ruminant
Cattle, dairy<br>(FAO Item Code 960)	Chicken, broilers<br>(FAO Item Code 1053)
Cattle, non-dairy<br>(FAO Item Code 961)	Chicken, layers<br>(FAO Item Code 1052)
Buffaloes<br>(FAO Item Code 946)	Swine, market<br>(FAO Item Code 1049)
Goats<br>(FAO Item Code 1016)	Swine, breeding<br>(FAO Item Code 1079)
Sheep<br>(FAO Item Code 976)	

For livestock, all categories also include secondary products (such as cheese in the case of milk) where data were available. Cattle products comprise beef meat and milk and buffalo meat and milk. Sheep and goat products comprise meat and milk. Poultry products comprise meat and eggs of chickens, geese, ducks, and turkeys. Pig products include pork and secondary processed commodities, such as ham and bacon.

Emissions from enteric fermentation, manure management and manure left on pasture are obtained from Herrero et al.[194] This information is presented in tonne carbon dioxide equivalent (CO_2e) per tropical livestock unit (tlu), which is converted to livestock head using the table below. The emissions per head are then multiplied by the number of animals per country obtained from the FAO database[195] to calculate the total emissions per livestock type per country.

	Head per tlu
Bovine (Buffalo, Cattle (dairy), Cattle<br>(non-dairy)	1.43
Small Ruminants (Goats, Sheep)	10

Poultry (Chicken)	100
Swine	5

The emissions per head are divided into world regions (as in the GLOBIOM model) and, for ruminants, livestock system (combination of climates from arid to humid, and practices from rangeland to feedlots, c.f. Herrero et al. 2013).[194] To convert these emissions to country values, an average is made across the region-system pairs within each country, weighted by the number of animals.

To obtain the emissions from grazing, the fertilizer applied to grassland from Chang et al.[196] is used. These overall emissions are then added to the direct livestock emissions to provide overall emissions rates for each commodity in the year 2000. Animal products' emissions the feed crop-related emissions were also incorporated (see next paragraph) proportionally to the feed ingredients consumed by animals - by species, region and systems - using feed data from Herrero et al. 2013.[194]

Finally, emissions intensity values for each commodity (egg, meat, milk) and country are obtained by dividing CO_2e values by the output of milk/meat/egg per head from Herrero et al. 2013.[194]

For Crops:

The emissions from fertilizer (synthetic and manure) application, rice cultivation and cultivation from organic soils for maize, rice, wheat, soybean and other crops for the year 2000 are obtained from Carlson et al. 2017,[197] who use IPCC methodology and a non-linear N_2O emission model. The full list of crops considered is as follows:

Main crops comprise wheat rice and maize, and each incorporate as many secondary commodities as were available. Other crops comprise barley, beans (dry and green), broad beans and horse beans, cassava, chickpeas, cotton, groundnuts, millet, mustard, oats, peas (dry and green), potatoes, rapeseed, rye, sesame seed, sorghum, soybeans, sweet potatoes, oil palm fruit, sugarcane and sugar beet, yams.

Crops used for livestock feed are excluded from the "crops" emissions, as they are included in the intensity of livestock production; the FAO reports this in the following way "Cereal crops harvested for hay or harvested green for food, feed or silage or used for grazing are therefore excluded".[195]

Production values 2001 – 2018

Since the emission intensity of production is not constant over time, its values by commodity (for both animal and crop products) were scaled using the FAO values as an index. The FAO produces GHG emissions intensity values by animal commodity and broad crop category (distinguishing rice, which, unlike other crops, emits large amounts of methane) for the countries covered by their analysis. However, these values are volatile at the country level, so regional values were used here. The percentage change from the year 2000 value was applied to the values derived from Herrero et al.,[194] Chang et al.[196] and Carlson et al.,[197] outlined above (methodology from Dalin et al.[198]). At the time of publication, the values for 2018 had not been published, so the intensity scaling was assumed to be the same as in 2017. This will be updated in future years. Any missing values in scaling factor were assumed to be 1. Any intensity values missing for a given country were given the regional average for that year and commodity, although practically this had little impact, because missing values only corresponded to countries which had very low or no production of the commodity in question.

Consumption emissions

The GHG emissions associated with agricultural commodity consumption uses FAO production and trade data to estimate the total GHG emissions footprint associated with each of the commodities considered in a given country. This method is used by Dalin et al.[199] for tracing water consumption in global food networks but is adapted here to calculated GHG footprint. The basic equation the indicator follows is:

Consumption = production + imports - exports

FAO production and trade data are used in the following manner. For a given commodity the national production values in tonnes are converted into CO_2e values using the GHG emissions intensity values supplied by indicator 3.5.1 GHG production estimates (via Carlson et al. 2017[197]) associated with producing that tonnage of the commodity. Next, secondary commodities are converted in primary equivalent values by multiplying the trade tonnage by the value derived from Dalin et al. 2017.[199] For example, the primary equivalences for wheat products are as follows:

Bran, wheat	1.01
Bread	0.88
Bulgur	1.05
Cereals, breakfast	1.18
Flour, wheat	1.01
Macaroni	1.01
Pastry	0.88
Wafers	0.88
Wheat	1.00

These values are then converted into GHG emissions equivalent, based on the GHG emissions intensity. For a given year, the trade balances are corrected to take into account that a given commodity may have been produced in one country, processed in another and finally imported into a third, using an algorithm developed by Kastner et al 2011.[200]

Data

1. National annual production of animal products items (tonnes) – FAOSTAT.[195]
2. National annual trade (country-country) of animal products items (tonnes) – FAOSTAT.[195]
3. Correspondence of items across item lists with different grouping – FAOSTAT.[195]
4. GHG emissions intensity per country of animal products – provided by LC 3.7 GHG production estimates including grassland and feed crop emissions (via Herrero et al. 2013 and Dalin et al. 2019).[194,198] Definitions: Animal types: bovine cattle (beef and buffalo), sheep and goat ruminants, pigs, poultry (chicken, ducks, geese and turkeys).
5. National annual production of crops (tonnes) – FAOSTAT.[195]
6. National annual trade (country-country) of crop products (tonnes) – FAOSTAT.[195]
7. GHG emissions intensity of crop products for each country– provided by Carlson et al. (2017).[197]

Caveats

In the context of this indicator, *consumption* refers to the net balance of food products entering a country within a given year, i.e. national production and net imports together, which could also be referred to as “national supply”. It does not refer to the total GHG emissions attributable to food consumed by individuals. Indeed, at present, this indicator only considers the emissions associated with food production described above and does not take into account emissions associated with food transport and processing, storage and waste, land use change and deforestation.[201]

For livestock, data on stock numbers has been extracted from FAO database, however, some data is missing for some years, most notably Somalia (missing data 2000-2011) for non-dairy cattle. Data on grazing emissions from small islands is also missing, and therefore imputed using regional average values as described above.

The emission factors differ from FAO numbers:

- For livestock, this is due to calculation of emissions of enteric fermentation, manure management and manure left on pasture at GLOBIOM region (n=29) and livestock system (n=8) level whereas the FAO use subcontinental (n=9) and climatic level (n=3).[195]

- For crops, this is due to the FAO assuming slightly higher synthetic N application, greater manure N inputs, and a linear emissions factor of 1%, in contrast to a mean of 0.77% used by the non-linear model of Carlson et al. (2017).[197]

Agricultural consumption emissions estimates are derived directly from FAO trade values (re-organised as producer-consumer trade only with the algorithm), as described above. Therefore, these values differ from the production estimates, which are based on extrapolating year 2000 figures. On average across all years, the estimate of total emissions due to consumption are 2.25% above production values, and do not differ by more than 10% in any given year. The sole exception to this is the estimates of the differences between production and consumption by WHO region and 2019 country HDI level.

Future form of the indicator

As highlighted above, the indicator does not take into account emissions associated with food transport and processing, storage and waste, land use change and deforestation. While these likely contribute small percentages of total emissions associated with food production,[201] they are important to understanding the overall picture of emissions, and so future forms of the indicator will incorporate estimates of these aspects.

Additional analysis

In terms of individual countries in 2018, India (0.7 $GtCO_2e$), China (0.6 $GtCO_2e$) and the USA (0.4 $GtCO_2e$) have the highest production emissions in absolute terms. In per capita terms, small countries which produce large amounts of ruminant animal products have the highest emissions. New Zealand had the highest total per capita agriculture emissions of 15 tCO_2e/person in 2018, followed by Uruguay (7.8 tCO2e/person), despite New Zealand having a lower-than-average production intensity of cattle and sheep meat. In New Zealand, total production emissions were 7% lower in 2018 compared to 2000, but consumption emissions have fallen 33% in the same period, with Uruguay seeing a similar fall in consumption emissions. Since these nations export large quantities of animal products, they consequently have the highest agricultural GHG exports per capita of 10.4 tCO2e/person and 5.0 tCO2e/person, respectively.

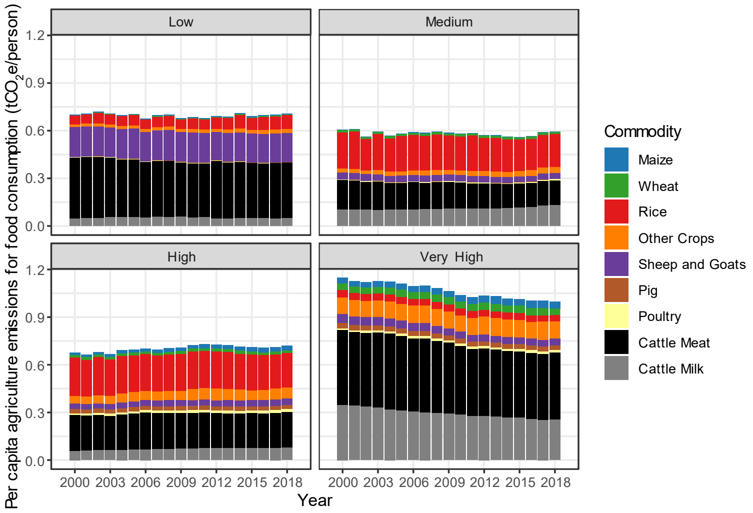

Figure 61: Per capita yearly greenhouse gas emissions associated with consumption of agri-food products, by 2019 Human Development Index country group and commodity, 2000-2018.

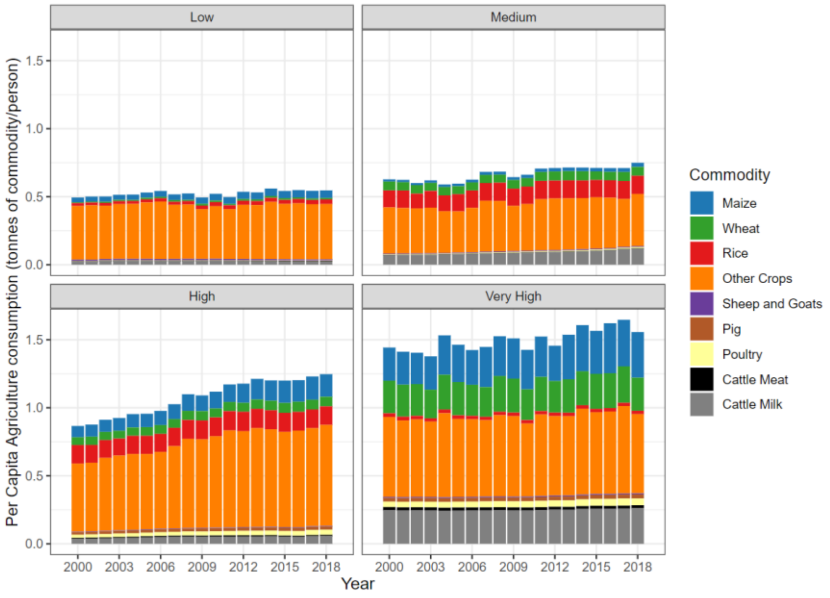

Figure 62. Total commodity consumption in kg/person by HDI group. This shows the total food commodity values input into each country with a given HDI. The figure demonstrates the levels of wastage in higher HDI countries, since a large proportion of food which enters a country's economy does not get eaten. These values are multiplied by the carbon intensity of each commodity for each country and year.

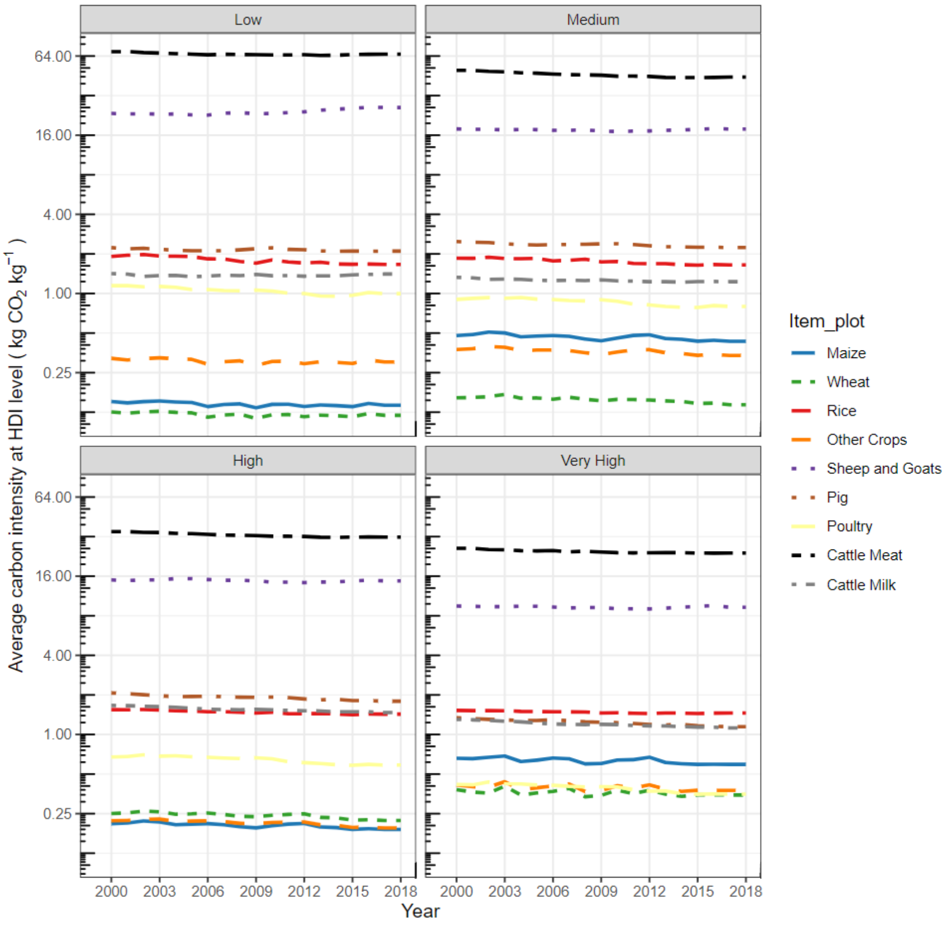

Figure 63. Average carbon intensity of each commodity group within the given 2019 HDI group by year in kgCO2/kg. The Y axis is a logarithmic scale.

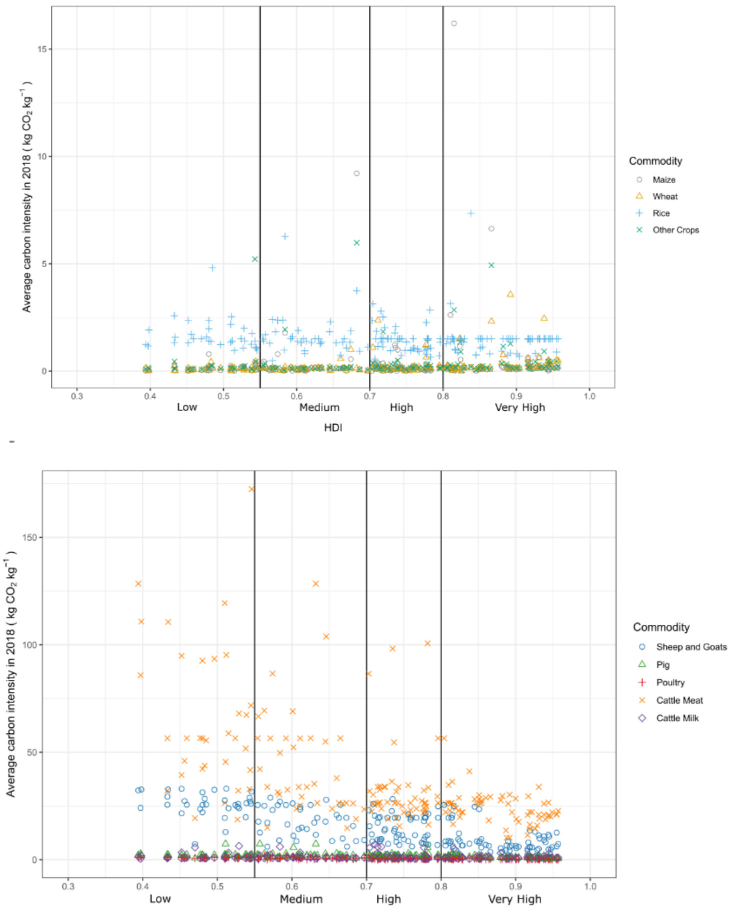

Figure 64. 2019 country HDI level of a country against the average carbon intensity of each commodity for that country in 2018. The upper panel shows crop commodities and the lower animal products. Note the Y-axis of the lower panel covers 10 times the emissions intensity as the upper. Along with Figure 63, this shows that production intensities for beef are much higher in low HDI countries than in very high HDI countries.

Indicator: 3.5.2 Diet and Health Co-Benefits

Methods

Several updates have been made to this indicator compared with the indicator described in the 2020 report of the Lancet Countdown. Consumption estimates are now differentiated by sex and age, as are weight estimates, and mortality rates have been updated by one year and are now differentiated by sex and age, using updated data from the GBD project.[202] Further detail is provided in the methods description below.

Baseline consumption data

Baseline food consumption was estimated by adopting estimates of food availability from the FAO's food balance sheets, and adjusting those for the amount of food wasted at the point of consumption.[203,204] This proxy for food consumption was disaggregated by age and sex by adopting the same age and sex-specific trends as observed in dietary surveys.[205]

An alternative would have been to rely on a set of consumption estimates that has been based on a variety of data sources, including dietary surveys, household budget and expenditure surveys, and food availability data.[206,207] However, neither the exact combination of these data sources, nor the estimation model used to derive the data have been made publicly available. For some individual countries, using dietary surveys would also have been an alternative. However, underreporting is a persistent problem in dietary surveys,[208,209] and regional differences in survey methods would have meant that the results would not be comparable between countries. In contrast to dietary surveys, waste-adjusted food-availability estimates indicate levels of energy intake per region that reflect differences in the prevalence of overweight and obesity across regions.[210]

Food balance sheets report on the amount of food that is available for human consumption.[204] They reflect the quantities reaching the consumer, but do not include waste from both edible and inedible parts of the food commodity occurring in the household. As such, the amount of food actually consumed may be lower than the quantity shown in the food balance sheet depending on the degree of losses of edible food in the household, e.g., during storage, in preparation and cooking, as plate-waste, or quantities fed to domestic animals and pets, or thrown away.

The waste-accounting methodology developed by the FAO was followed to account for the amount of food wasted at the household level that was not accounted for in food availability estimates.[203] Table 25 provides and overview of the parameters used in the calculation.

For each commodity and region, food consumption was estimated by multiplying food availability data with conversion factors (*cf*) that represent the amount of edible food (e.g. after peeling) and with the percentage of food wasted during consumption (*1-wp(cns)*). For roots and tubers, fruits and vegetables, and fish and seafood, the differences in wastage between the proportion that is utilised fresh (pct_{frsh}) and the proportion that utilised in processed form (pct_{prcd}) was also accounted for. The equation used for each food commodity and region was:

$$Consumption = Availability \cdot \frac{pct_{frsh}}{100} \cdot cf_{frsh} \cdot \left(1 - \frac{wp(cns_{frsh})}{100}\right) + Availability \cdot \frac{pct_{prcd}}{100} \cdot cf_{prcd} \cdot \left(1 - \frac{wp(cns_{prcd})}{100}\right)$$

Table 25. Percentage of food wasted during consumption (cns), and percentage of processed utilisation (pctprcd). The percentage of fresh utilisation is calculated as 1-pctprcd. Conversion factors to edible portions of foods are provided below the table.

Food group	Item	Region						
		Europe	USA, Canada, Oceania	Indus-trialized Asia	Sub-Saharan Africa	North Africa, West and Central Asia	South and Southeast Asia	Latin America
cereals	wp(cns)	25	27	20	1	12	3	10
roots and tuber	pct_{prcd}	73	73	15	50	19	10	80
	wp(cns)	17	30	10	2	6	3	4
	wp(cns_{prcd})	12	12	12	1	3	5	2
oilseeds and pulses	cns	4	4	4	1	2	1	2
fruits and vegetables	pct_{prcd}	60	60	4	1	50	5	50
	wp(cns)	19	28	15	5	12	7	10
	wp(cns_{prcd})	15	10	8	1	1	1	1
milk and dairy	wp(cns)	7	15	5	0.1	2	1	4
eggs	wp(cns)	8	15	5	1	12	2	4
meat	wp(cns)	11	11	8	2	8	4	6
fish and seafood	pct_{prcd}	*40% for low-income countries, and 96% for all others.*						
	wp(cns)	11	33	8	2	4	2	4
	wp(cns_{prcd})	10	10	7	1	2	1	2

Conversion factors: maize, millet, sorghum: 0.69; wheat, rye, other grains: 0.78; rice: 1; roots: 0.74 (0.9 for industrial processing); nuts and seeds: 0.79; oils: 1; vegetables: 0.8 (0.75 for industrial processing); fruits: 0.8 (0.75 for industrial processing); beef: 0.715; lamb: 0.71; pork: 0.68; poultry: 0.71; other meat: 0.7; milk and dairy: 1; fish and seafood: 0.5; other crops: 0.78

Comparative risk assessment

The mortality and disease burden attributable to dietary and weight-related risk factors was estimated by calculating population impact fractions (PIFs) which represent the proportions of disease cases that would be avoided when the risk exposure was changed from a baseline situation to a counterfactual situation. For calculating PIFs, the general formula was used:[211-213]

$$PIF = \frac{\int RR(x)P(x)dx - \int RR(x)P'(x)dx}{\int RR(x)P(x)dx}$$

where $RR(x)$ is the relative risk of disease for risk factor level x, $P(x)$ is the number of people in the population with risk factor level x in the baseline scenario, and $P'(x)$ is the number of people in the population with risk factor level x in the counterfactual scenario. It was assumed that changes in relative risks follow a dose-response relationship,[212] and that PIFs combine multiplicatively, i.e. $PIF = 1 - \prod_i(1 - PIF_i)$ where the i's denote independent risk factors.[212,214]

The number of avoided deaths due to the change in risk exposure of risk *i*, $\Delta deaths_i$, was calculated by multiplying the associated PIF by disease-specific death rates, *DR*, and by the number of people alive within a population, *P*:

$$\Delta deaths_i(r,s,a,d) = PIF_i(r,s,a,d) \cdot DR(r,s,a,d) \cdot P(r,s,a)$$

Where PIFs are differentiated by region *r*, sex *s*, age group *a*, and disease/cause of death *d*; the death rates are differentiated by region, sex, age group, and disease; the population groups are differentiated by region, sex, and age group; and the change in the number of deaths is differentiated by region, sex, age group, and disease.

Publicly available data sources were used to parameterize the comparative risk analysis. Mortality and population data were adopted from the GBD project.[202] Baseline data on the weight distribution in each country were adopted from a pooled analysis of population-based measurements undertaken by the NCD Risk Factor Collaboration.[210]

The relative risk estimates that relate the risk factors to the disease endpoints were adopted from meta-analyses of prospective cohort studies for dietary and weight-related risks.[215-221] In line with the meta-analyses, non-linear dose-response relationships were included for fruits, vegetables, and nuts and seeds, and linear dose-response relationships were assumed for the remaining risk factors. As the analysis was primarily focused on mortality from chronic diseases, the focus was on adults aged 20 years or older, and the relative risk estimates were adjusted for attenuation with age based on a pooled analysis of cohort studies focussed on metabolic risk factors, [222] in line with other assessments.[213,223]

Table 26 provides an overview of the relative risk parameters used. For the counterfactual scenario, defined minimal risk exposure levels (TMRELs) were defined as follows: 300 g/d for fruits, 500 g/d for vegetables, 100 g/d for legumes, 20 g/d for nuts and seeds, 0 g/d for red meat, and no underweight, overweight, or obesity. The TMRELs are in line with those defined by the Nutrition and Chronic Diseases Expert Group (NutriCoDE),[223] with the exception that higher value for vegetables was used, and zero as minimal risk exposure for red meat was used, in each case based on a more comprehensive meta-analysis.[217,218]

The selection of risk-disease associations used in the health analysis was supported by available criteria used to judge the certainty of evidence, such as the Bradford-Hill criteria used by the Nutrition and Chronic Diseases Expert Group (NutriCoDE),[223] the World-Cancer-Research-Fund criteria used by the GBD project,[224] as well as NutriGrade (Table 27).[225] The certainty of evidence supporting the associations of dietary risks and disease outcomes as used here were graded as moderate or high with NutriGrade,[218-220] and/or assessed as probable or convincing by the Nutrition and Chronic Diseases Expert Group,[223] and by the World Cancer Research.[226] The certainty of evidence grading in each case relates to the general relationship between a risk factor and a health outcome, and not to a specific relative risk value.

Not all available risk-disease associations that were graded as having a moderate certainty of evidence and showed statistically significant results in the meta-analyses that included NutriGrade assessments were included.[218-220] That was because for some associations, such as for milk and fish, more detailed meta-analyses (with more sensitivity analyses) were available that indicated potential confounding with other major dietary risks or health status at baseline.[227-229] Such sensitivity analyses were not presented in the meta-analyses that included NutriGrade assessments, but they are important for health assessments that evaluate changes in multiple risk factors.

Table 26. Relative risk parameters (mean and low and high values of 95% confidence intervals) for dietary risks and weight-related risks.

Food group	Endpoint	Unit	RR mean	RR low	RR high	Reference
Red meat	CHD	100 g/d	1.15	1.08	1.23	Bechthold et al (2019)
	Stroke	100 g/d	1.12	1.06	1.17	Bechthold et al (2019)
	Colorectal cancer	100 g/d	1.12	1.06	1.19	Schwingshackl et al (2018)
	Type 2 diabetes	100 g/d	1.17	1.08	1.26	Schwingshackl et al (2017)
Fruits	CHD	100 g/d	0.95	0.92	0.99	Aune et al (2017)
	Stroke	100 g/d	0.77	0.70	0.84	Aune et al (2017)
	Cancer	100 g/d	0.94	0.91	0.97	Aune et al (2017)
Vegetables	CHD	100 g/d	0.84	0.80	0.88	Aune et al (2017)
	Cancer	100 g/d	0.93	0.91	0.95	Aune et al (2017)
Legumes	CHD	57 g/d	0.86	0.78	0.94	Afshin et al (2014)
Nuts	CHD	28 g/d	0.71	0.63	0.80	Aune et al (2016)
Underweight	CHD	15<BMI<18.5	1.17	1.09	1.24	Global BMI Collab (2016)
	Stroke	15<BMI<18.5	1.37	1.23	1.53	Global BMI Collab (2016)
	Cancer	15<BMI<18.5	1.10	1.05	1.16	Global BMI Collab (2016)
	Respiratory disease	15<BMI<18.5	2.73	2.31	3.23	Global BMI Collab (2016)
Overweight	CHD	25<BMI<30	1.34	1.32	1.35	Global BMI Collab (2016)
	Stroke	25<BMI<30	1.11	1.09	1.14	Global BMI Collab (2016)
	Cancer	25<BMI<30	1.10	1.09	1.12	Global BMI Collab (2016)
	Respiratory disease	25<BMI<30	0.90	0.87	0.94	Global BMI Collab (2016)
	Type 2 diabetes	25<BMI<30	1.88	1.56	2.11	Prosp Studies Collab (2009)
Obesity (grade 1)	CHD	30<BMI<35	2.02	1.91	2.13	Global BMI Collab (2016)
	Stroke	30<BMI<35	1.46	1.39	1.54	Global BMI Collab (2016)
	Cancer	30<BMI<35	1.31	1.28	1.34	Global BMI Collab (2016)
	Respiratory disease	30<BMI<35	1.16	1.08	1.24	Global BMI Collab (2016)
	Type 2 diabetes	30<BMI<35	3.53	2.43	4.45	Prosp Studies Collab (2009)
Obesity (grade 2)	CHD	30<BMI<35	2.81	2.63	3.01	Global BMI Collab (2016)
	Stroke	30<BMI<35	2.11	1.93	2.30	Global BMI Collab (2016)
	Cancer	30<BMI<35	1.57	1.50	1.63	Global BMI Collab (2016)
	Respiratory disease	30<BMI<35	1.79	1.60	1.99	Global BMI Collab (2016)
	Type 2 diabetes	30<BMI<35	6.64	3.80	9.39	Prosp Studies Collab (2009)
Obesity (grade 3)	CHD	30<BMI<35	3.81	3.47	4.17	Global BMI Collab (2016)
	Stroke	30<BMI<35	2.33	2.05	2.65	Global BMI Collab (2016)
	Cancer	30<BMI<35	1.96	1.83	2.09	Global BMI Collab (2016)
	Respiratory disease	30<BMI<35	2.85	2.43	3.34	Global BMI Collab (2016)
	Type 2 diabetes	30<BMI<35	12.49	5.92	19.82	Prosp Studies Collab (2009)

Table 27. Overview of existing ratings on the certainty of evidence for a statistically significant association between a risk factor and a disease endpoint. The ratings include those of the Nutrition and Chronic Diseases Expert Group (NutriCoDE),46 the World Cancer Research Fund,[226] and NutriGrade, [218-220] The ratings relate to the risk-disease associations in general, and not to the specific relative risk factor used for those associations in this analysis.

Food group	Endpoint	Association	Certainty of evidence
Fruits	CHD	reduction	NutriCoDE: probable or convincing;<br>NutriGrade: moderate quality of meta-evidence
	Stroke	reduction	NutriCoDE: probable or convincing<br>NutriGrade: moderate quality of meta-evidence
	Cancer	reduction	WCRF: strong evidence (probable) for some cancers<br>NutriGrade: moderate quality of meta-evidence for colorectal cancer
Vegetables	CHD	reduction	NutriCoDE: probable or convincing<br>NutriGrade: moderate quality of meta-evidence
	Cancer	reduction	WCRF: strong evidence (probable) for non-starchy vegetables and some cancers<br>NutriGrade: moderate quality of meta-evidence for colorectal cancer
Legumes	CHD	reduction	NutriCoDE: probable or convincing<br>NutriGrade: moderate quality of meta-evidence
Nuts and seeds	CHD	reduction	NutriCoDE: probable or convincing<br>NutriGrade: moderate quality of meta-evidence
Red meat	CHD	increase	NutriGrade: moderate quality of meta-evidence
	Stroke	increase	NutriGrade: moderate quality of meta-evidence
	Cancer	increase	WCRF: strong evidence (probable) for colorectal cancer<br>NutriGrade: moderate quality of meta-evidence for colorectal cancer
	Type-2 diabetes	increase	NutriCoDE: probable or convincing<br>NutriGrade: high quality of meta-evidence

NutriCoDE: Nutrition and Chronic Diseases Expert Group
NutriGrade: Grading of Recommendations Assessment, Dvelopment, and Evaluation (GRADE) tailored to nutrition research
WCRF: World Cancer Research Fund

For the different diet scenarios, uncertainty intervals associated with changes in mortality based on standard methods of error propagation and the confidence intervals of the relative risk parameters were calculated. For the error propagation, the error distribution of the relative risks by a normal distribution was approximated and that side of deviations from the mean which was largest was used. This method leads to conservative and potentially larger uncertainty intervals as probabilistic methods, such as Monte Carlo sampling, but it has significant computational advantages, and is justified for the magnitude of errors dealt with here (<50%) (see e.g. IPCC Uncertainty Guidelines).

Data

Type	Coverage	Source
Exposure data:		
Food consumption data	Country-level	Food availability data were taken from the FAO, adjusted for food waste at the household level and for age and sex-specific trends.[203,205,230] Estimates of energy intake were in line with trends in body weight across countries.[210]
Weight estimates	Country-level	Baseline data from pooled analysis of measurement studies differentiated by sex and age with global coverage. [210]
Health analysis:		

Relative risk estimates	General	Adopted from meta-analysis of prospective cohort studies. [215-221] The certainty of evidence for the risk-disease associations were rated as moderate to high by NutriGrade. [218-220]
Mortality and population data	Country-level	Adopted from the Global Burden of Disease project by country, sex, and age group.[202]

Caveats

In the comparative risk assessment, relative risk factors that are subject to the caveats common in nutritional epidemiology, including small effect sizes and potential measurement error of dietary exposure, such as over and underreporting and infrequent assessment were used.[231] For the calculations, it was assumed that the risk-disease relationships describe causal associations, an assumption supported by the existence of statistically significant dose-response relationships in meta-analyses, the existence of plausible biological pathways, and supporting evidence from experiments, e.g. on intermediate risk factors.[215,217-221,223,232-235] However, residual confounding with unaccounted risk factors cannot be ruled out in epidemiological studies. Additional aspects rarely considered in meta-analyses are the importance of substitution between food groups that are associated with risks, and the time lag between dietary exposure and disease.

To address potential confounding, risk-disease associations that became non-significant in fully adjusted models were omitted, in particular those related milk intake,[227,228] and to fish intake.[229,236-239] The quality of evidence in meta-analyses that covered the same risk-disease associations as used here was graded with NutriGrade as moderate or high for all risk-disease pairs included in the analysis (Table 27).[218-220] In addition, the Nutrition and Chronic Diseases Expert Group and the World Cancer Research Fund graded the evidence for a causal association of ten of the 12 risk-disease associations included in the analysis as probable or convincing,[223,226] The relative health ranking of leading risk factors found in our analysis was similar to existing rankings that relied on different relative risk parameters and exposure data.[224,240]

As exposure data, a proxy of food consumption that was derived from estimates of food availability that were adjusted for the amount of food wasted at the point of consumption was used.[203,204] An alternative would have been to rely on a set of consumption estimates that has been based on a variety of data sources, including dietary surveys, household budget and expenditure surveys, and food availability data.[206,207] However, neither the exact combination of these data sources, nor the estimation model used to derive the data have been made publicly available. For some individual countries, using dietary surveys would also have been an alternative. However, underreporting is a persistent problem in dietary survey,[208,209]and regional differences in survey methods would have meant that results would not be comparable between countries. In contrast to dietary surveys, waste-adjusted food-availability estimates indicate levels of energy intake per region that reflect differences in the prevalence of overweight and obesity across regions.[210]

Additional analysis

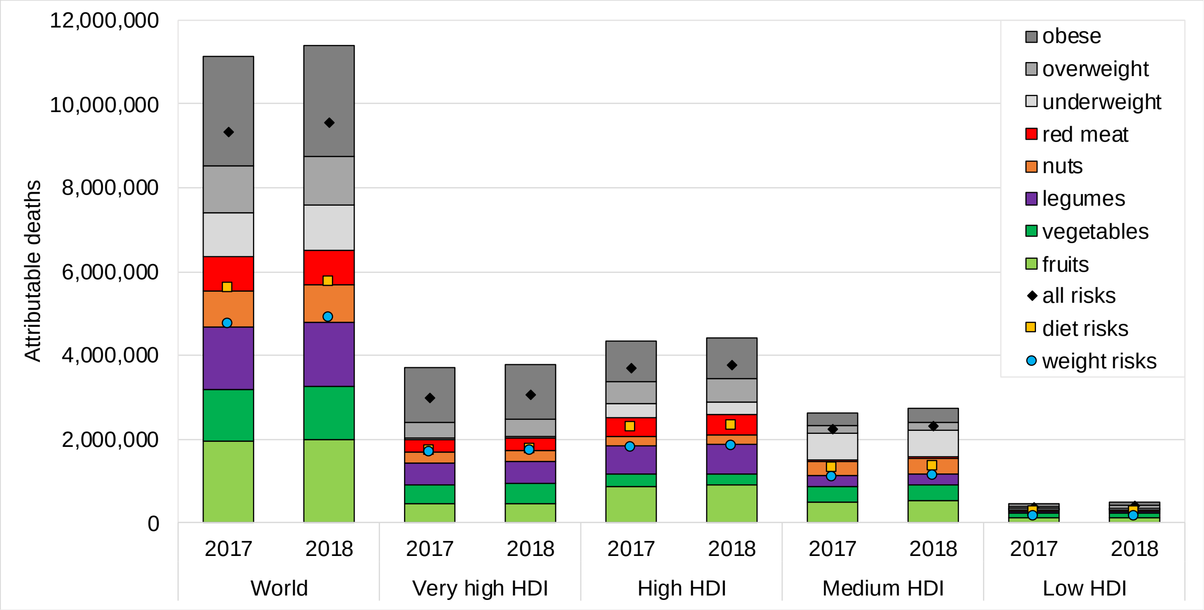

Figure 65 Deaths attributable to imbalanced diets in 2017 and 2018 by risk factor and 2019 country HDI level. The risk factors include low intake of fruits, vegetables, legumes, and nuts, high intake of red meat, as well as being underweight, overweight, or obese.

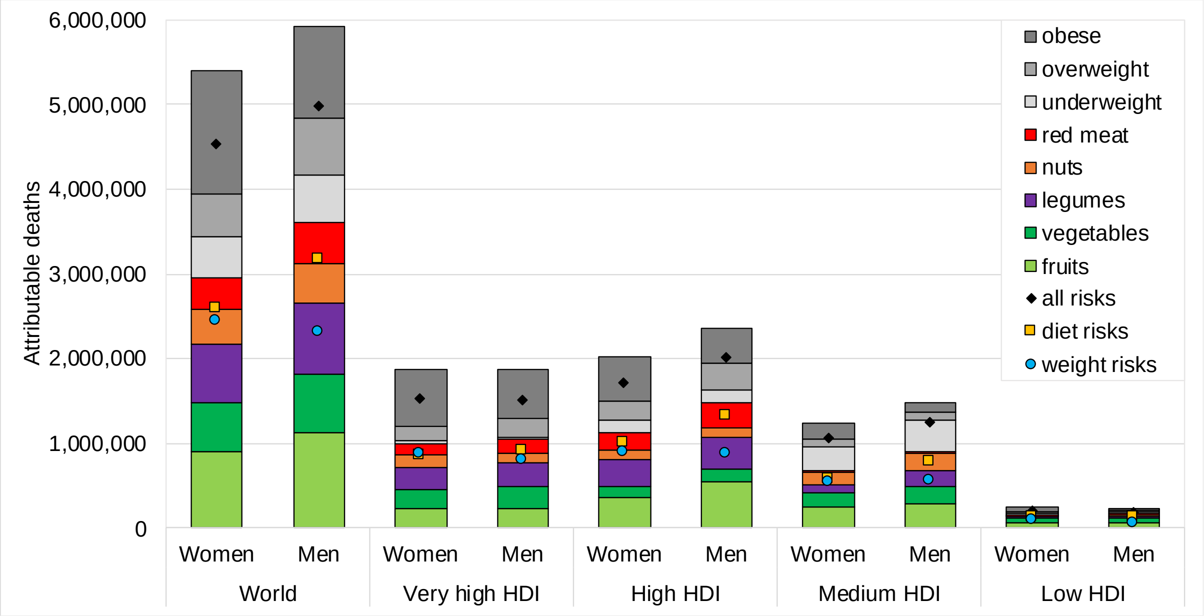

Figure 66 Deaths attributable to imbalanced diets in 2018 by risk factor, sex, and 2019 country HDI level. The risk factors include low intake of fruits, vegetables, legumes, and nuts, high intake of red meat, as well as being underweight, overweight, or obese.

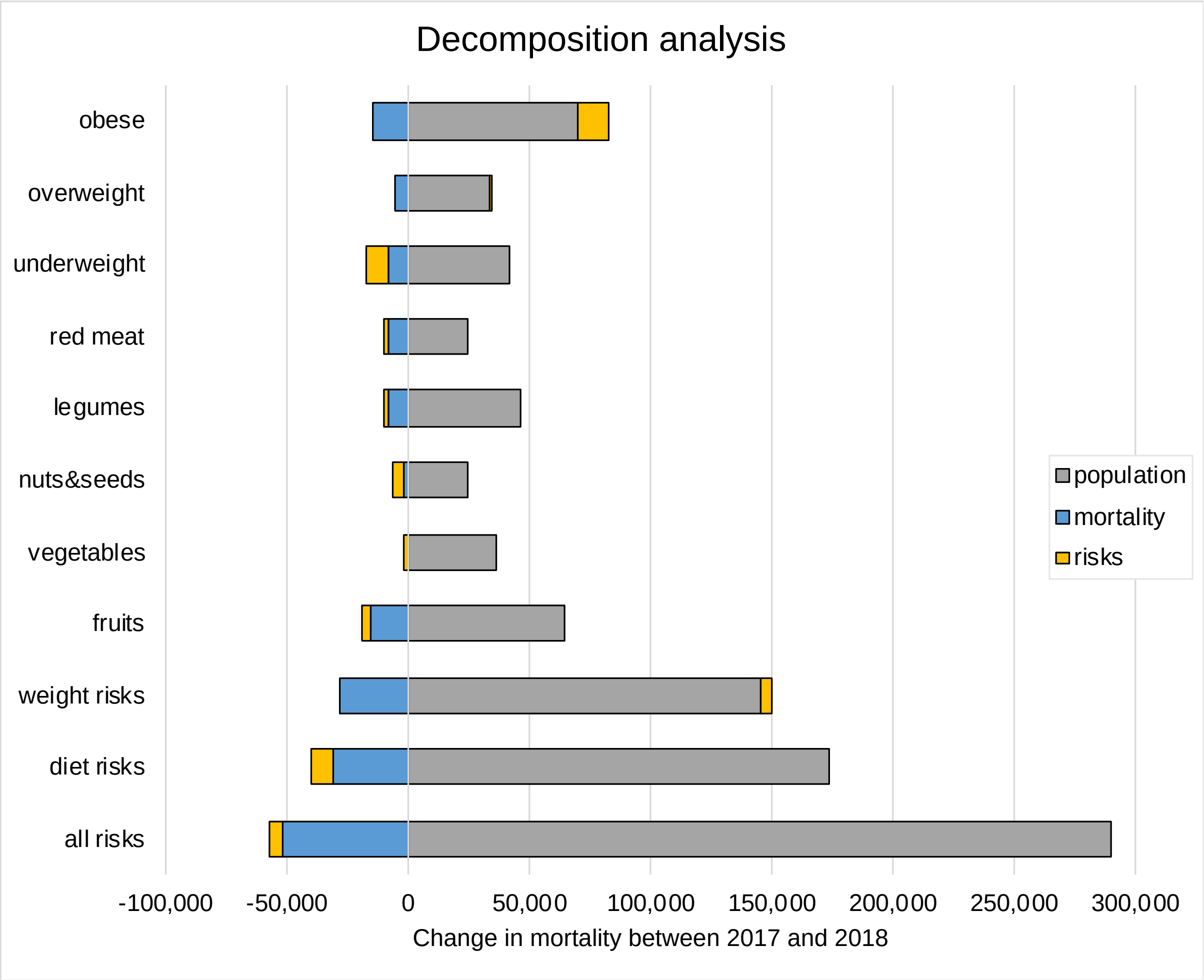

Figure 67 Change in deaths attributable to imbalanced diets between 2017 and 2018 by risk factor and changing parameter. The risk factors include low intake of fruits, vegetables, legumes, and nuts, high intake of red meat, as well as being underweight, overweight, or obese. The parameters include changes in population, mortality rates, and risk factors.

Table 28 Overview of risk factors by sex and 2019 country HDI level.

Risk factor by sex	Region by HDI				
	World	Very high	High	Medium	Low
diet (intake in grams per person per day)					
**fruits**					
BTH	141	165	171	110	76
FML	146	172	179	107	75
MLE	137	157	162	112	78
**legumes**					
BTH	23	12	16	37	36
FML	25	12	16	41	37
MLE	22	13	16	33	36
**nuts_seeds**					
BTH	10	13	13	4	12
FML	10	12	13	4	12
MLE	10	13	13	4	12
**red_meat**					
BTH	52	72	80	11	21
FML	51	67	78	13	21
MLE	53	76	81	9	20
**vegetables**					
BTH	265	205	421	156	109
FML	267	211	421	157	109
MLE	264	199	420	155	109
weight (proportion)					
**obese**					
BTH	0.13	0.27	0.13	0.06	0.08
FML	0.16	0.29	0.16	0.08	0.11
MLE	0.11	0.26	0.11	0.04	0.04
**overweight**					
BTH	0.26	0.35	0.29	0.18	0.19
FML	0.25	0.30	0.27	0.19	0.22
MLE	0.27	0.41	0.31	0.18	0.16
**underweight**					
BTH	0.09	0.02	0.05	0.18	0.11
FML	0.09	0.02	0.06	0.19	0.11
MLE	0.09	0.01	0.04	0.18	0.12
**normal**					
BTH	0.51	0.36	0.53	0.57	0.62
FML	0.50	0.39	0.52	0.54	0.57
MLE	0.53	0.33	0.54	0.60	0.68

Indicator 3.6: Mitigation in the Healthcare Sector

Methods

This indicator is in the form of healthcare-associated GHG emissions per capita per year, including direct emissions from healthcare facilities as well as emissions from the consumption of goods and services supplied by other sectors. Results are calculated by assigning aggregate national health expenditures from WHO to final demand for 'Health and Social Work' sectors in the multi-regional input-output (MRIO) model. Environmental satellite accounts including GHG emissions accompany each MRIO model. Consumption-based GHG emissions are then calculated using the standard Leontief inverse technique.[241]

Results for years after the MRIO model year are achieved through deflation of healthcare expenditure data. Both WIOD and EXIOBASE3 MRIO models were run for this analysis and results compared; WIOD results are shown following the prior year report methods, while EXIOBASE3 results showed severe year-to-year volatility in some results that could not be readily explained.[242,243] WIOD tables are in US dollars, while EXIOBASE3 tables are in euros. For expenditure years after the model baseline, WHO expenditure data in nominal US dollars expenditures are converted to nominal national currencies using market exchange rates, deflated in national currencies to baseline year using consumer price indices from the World Bank, and converted to baseline model year euros currency (dollars or euros) using market exchange rates.[244,245]

The Lancet Countdown reported healthcare sector GHG emissions for the first time in 2019.[73] In that report, global healthcare emissions were found to contribute approximately 4.6% of global emissions, with large disparities in per capita emissions of more than 40x across the countries studied. Independent research by Pichler et al. on CO_2 emissions (excluding other GHGs) associated with health care in OECD countries (excluding Chile) as well as India and China found a contribution of 4.4% in 2014, while an NGO effort covering all GHG emissions estimated 4.4% in 2014.[246,247] The Pichler et al. work considered temporal trends and introduced adjustments into the emissions satellite accounts of the MRIO model EORA to reflect shifts in major GHG emissions sources that occurred between the baseline model year and when each healthcare expenditure occurred. Based on this suggestion, the Lancet Countdown modelling approach has been updated in 2020 in the same way, using the PRIMAP database of national GHG emissions to adjust emissions by sector relative to the baseline year.[248]

Data

1. Environmentally extended multi-region input-output tables: WIOD 2013 release with environmental accounts, latest model year 2011, latest emissions account year 2009, air emissions include CO_2, CH_4, N_2O, NO_x, SO_x, CO, NMVOC, and NH_3;
2. Per capita health expenditure data is from the World Health Organization's Global Health Expenditure Database; the latest reporting year is 2018.[249] Population data is also from the WHO.[245]
3. Market exchange rates are from UN Statistics Division.[250]
4. Consumer price indices are from the World Bank.[244]

Caveats

As only total health expenditure data are available from WHO, all expenditures are assigned to Final Demand, with no separation for investment.

MRIO models are built from aggregated top-down statistical data. Results do not reflect individual health care systems' power purchase agreements for renewable energy or any offsetting activities. Results do not include direct emissions of waste anaesthetic gases from clinical operations nor emissions from metered dose inhalers, as these are not currently reported consistently in national emissions inventories.

Future form of the indicator

This indicator could be expressed in future years by sectoral contributions, in order to isolate the contribution of specific healthcare supplies and activities such as pharmaceutical manufacturing.

Additional analysis

The results of the analysis of per capita healthcare emissions and HDI are similar to the comparison of per capita healthcare emissions and healthcare access and quality (HAQ) undertaken in last year's report.

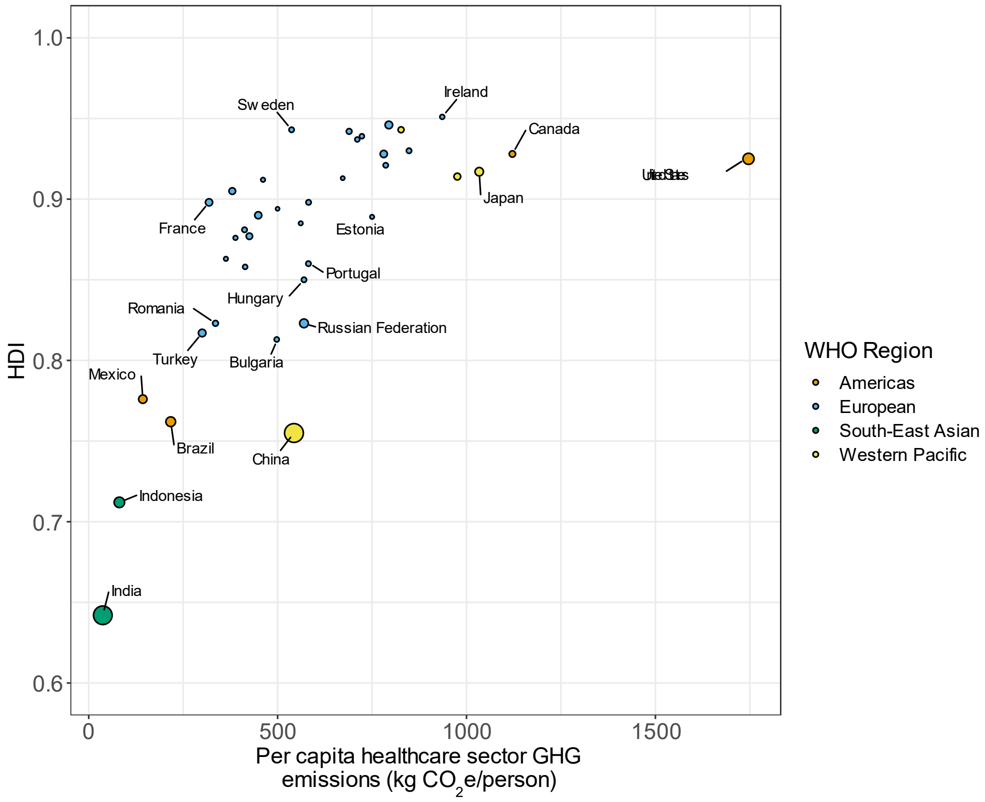

Figure 68:National per capita healthcare greenhouse gas emissions for 2018 against 2019 country Human Development Index level. Dot size is proportional to population

Section 4: Economics and Finance

4.1: Health and Economic Costs of Climate Change

Indicator 4.1.1: Economic Losses due to Climate-Related Extreme Events

Methods

The Swiss Re Institute provided the data for this indicator. The Swiss Re Institute sigma catastrophe database is an international commercial database recording both natural and man-made disasters from 1970 and has over 12,000 entries.

The term 'natural catastrophe' refers to an event caused by natural forces. Such an event generally results in a large number of individual losses involving many insurance policies. The scale of the losses resulting from a catastrophe depends not only on the severity of the natural forces concerned, but also on man-made factors, such as building design or the efficiency of disaster control in the afflicted region.

Natural catastrophes are categorised as follows:

Category	Peril Group	Peril
	Earthquake	Earthquake
		Tsunami
		Volcano eruption
	Weather-related	Storm
		Flood
		Hail
		Cold, frost
		Drought, bush fires, heat waves
		Other natural catastrophes

For this indicator, only data for 'weather-related' events are presented.

Total (insured and uninsured) economic losses reported by Swiss Re are all the financial losses directly attributable to a major event, i.e. damage to buildings, infrastructure, vehicles etc. This also includes losses due to business interruption as a direct consequence of the property damage. Insured losses are gross of any reinsurance, be it provided by commercial or government schemes. Total loss figures do not include indirect financial losses – i.e. loss of earnings by suppliers due to disabled businesses, estimated shortfalls in GDP and non-economic losses, such as loss of reputation or impaired quality of life. Insured losses refer to all insured losses except liability. To calculate uninsured losses, insured losses are subtracted from total losses.

Data are collected from a variety of sources, both internal and external. These include professional insured claims aggregators as well as insurance associations. Among the sources are also official government data, when available. Economic loss data can be estimated on the basis of Swiss Re proprietary catastrophe risk models. Also, if insured loss data are available, economic loss data are estimated on the basis of the local insurance penetration and other event-specific information (such as damages to public infrastructure, number of buildings damaged or destroyed etc.).

Minimum thresholds apply to inclusion in the database. At least one of the following must apply, for events recorded in 2020 (with economic values changing each year following changes to US CPI):

- **Insured losses (claims):** USD 52.7 million (maritime disasters), UDS 42.9 million (aviation), USD 53.3 million (other)
- **Economic losses**: USD 10.4 million
- **Casualties**: Dead or missing: 20; Injured: 50; Homeless: 2000

Loss values are presented in US$, or if initially expressed in local currency, converted to US$ using year-end exchange rates.

In previous years, country data were then summed into the four World Bank income groups. For this report, country data are summed into the four HDI classifications (very high, high, medium, low). Further information on the methodology of the sigma explorer database can be found here: https://www.sigma-explorer.com/documentation/Methodology_sigma-explorer.com.pdf.Total insured and uninsured losses are then divided by total GDP for each year and HDI group. GDP data are taken from the IMF's World Economic Outlook (April 2021 Edition). All values are in current prices.

Data

1. Swiss Re Institute sigma catastrophe database.[251]
2. IMF World Economic Outlook (April 2021).[252]

Caveats

Only events with measurable economic losses above the threshold levels are included. Each natural catastrophe event recorded is assigned a direct economic loss, and where applicable, an insured loss. Where available, data are taken from official institutions, but where not, estimates are calculated. The process for estimation depends on what data is available. For example, if loss estimates from insurance market data is available, this data may be combined with data on insurance penetration and other event-specific information to estimate total economic losses. If only low-quality information is available, such as a description of the number of homes damaged or destroyed, assumptions on value and costs are made.

Additional analysis

Table 29. Insured and uninsured losses from climate-related extreme events 2010-2020, by 2019 HDI group.

		Number of Events	Insured Losses/$1000 GDP	Uninsured Losses/$1000 GDP
2010	Very High	126	0.58	0.90
	High	67	0.10	4.88
	Medium	44	0.06	5.54
	Low	23	0.00	0.22
2011	Very High	92	0.96	0.77
	High	68	0.98	3.04
	Medium	24	0.01	2.15
	Low	17	0.03	0.50
2012	Very High	105	1.20	1.21
	High	68	0.06	1.44
	Medium	22	0.00	0.89
	Low	28	0.07	0.89
2013	Very High	117	0.60	0.58
	High	67	0.17	2.37
	Medium	27	0.19	1.37
	Low	11	0.00	0.05
2014	Very High	121	0.48	0.40
	High	71	0.11	1.60
	Medium	28	0.25	3.88
	Low	16	0.00	0.10
2015	Very High	115	0.50	0.42
	High	65	0.06	1.05
	Medium	44	0.32	1.63
	Low	19	0.00	0.84
2016	Very High	109	0.69	0.63
	High	67	0.11	2.48
	Medium	29	0.13	1.59
	Low	20	0.11	2.04

2017	Very High	146	2.51	2.77
	High	59	0.12	1.28
	Medium	33	0.02	1.11
	Low	18	0.00	0.89
2018	Very High	119	1.38	0.93
	High	44	0.05	0.75
	Medium	32	0.08	1.28
	Low	20	0.01	0.23
2019	Very High	132	0.90	0.64
	High	39	0.04	1.18
	Medium	37	0.12	3.25
	Low	29	0.15	3.20
2020	Very High	138	1.37	0.70
	High	49	0.12	1.52
	Medium	38	0.18	5.60
	Low	17	0.00	0.58

Indicator 4.1.2: Monetized value of Heat-related Mortality

Methods

This indicator used the value of statistical life-year (VSLY) to monetise the years of life lost (YLL) caused by heat-related mortality (data for which is provided by indicator 1.1.6). Compared to last year's method that used the value of a statistical life (VSL) to monetise mortality, the usage of VSLY can reflect age structure differences of heat-related mortalities across countries. VSLY measures how people value the discounted years of remaining life.[253] VSL can be interpreted as the discounted sum of VSLY of each year remained in life, therefore, mathematically, the VSLY can be derived from the VSL and how many remaining years people are expected to live at certain age (Eq.2). As for the change of VSLY to age, some studies assumed that VSLY is constant across age span, while others assumed that VSLY will increase before mid-age and then decrease till death, which is an Inverted-U shape.[254]169 countries spanning six World Health Organization (WHO) regions were included in the estimation. Population and GDP per capita are taken from the World Bank[255]and OECD[256] statistics. The life table used to derive remaining years of life, was taken from WHO.[257]

The same ratio between VSLY and GDP-per-capita is assumed for each country for years 2000-2019, and data from OCED countries was used as the basis to derive the ratio on account of data availability and method consistency across reports in different years. The assumption is shown in Eq. (1), where *Y* denotes the gross domestic product (GDP) per capita, *i* denotes the country *i* in WHO regions, *t* denotes time.

$$\frac{VSLY_{it}}{Y_{it}} = \frac{VSLY_{OECD}}{Y_{OECD}} \qquad (1)$$

The relationship between VSL and VSLY can be obtained by years of remaining life at death (L) and discount rate (r), which was demonstrated in Eq.(2). The average VSLY applicable for the OECD countries (VSL_{OECD}) was estimated US$3.83 million ($2015) in 2015, and average GDP per capita for OECD countries was $40,494 ($2015) in 2015. Here it is assumed the VSLY remains constant for each remaining life year because only mortality of people aging over 65 is considered, where the fluctuations of VSLYs are very small even under the Inverted-U assumption[254]. The discount rate used here is 3%.

$$VSLY_{it} = \frac{VSL_{it} \cdot r}{1-(1+r)^{-L}} \qquad (2)$$

In order to calculate the monetised value of years of life loss (YLL) relative to per-capita GDP (*R*), Eq.(3) was applied, where YLL is multiplied by the fixed VSLY-to-GDP per capita-ratio produced by Eq.(1).

$$R_{it} = \frac{VSLY_{it} * YLL_{it}}{Y_{it}} = \frac{VSLY_{OECD}}{Y_{OECD}} * YLL_{it} \qquad (3)$$

In order to calculated to monetised value of years of life loss as a proportion of GDP (V), Eq.(4) was applied, where YLL as a proportion of total population (P) is multiplied by the fixed VSLY-to-GDP per capita-ratio in OECD countries.

$$V_{it} = \frac{VSLY_{it} * YLL_{it}}{GNI_{it}} = \frac{VSLY_{it} * YLL_{it}}{Y_{it} * P_{it}} = \frac{VSLY_{OECD}}{Y_{OECD}} * \frac{YLL_{it}}{P_{it}} \qquad (4)$$

This year GDP was used instead of GNI used last year to keep pace with other economic costs valuation indicators. However, the differences caused by using GDP or GNI per capita in results are very small, as GDP and GNI per capita values for the OECD countries are very close to each other.

Country-level results are aggregated according both to WHO regions and HDI level. Considering data availability, some countries in WHO regions are not included: Cabo Verde, Sao Tome and Principe, Saint Vincent and the Grenadines, US Virgin Islands, Samoa, Eritrea, Andorra, Antigua and Barbuda, Bahrain, Barbados, Cook Islands, Dominica, Grenada, Kiribati, Maldives, Malta, Marshall Islands, Micronesia, Monaco, Montenegro, Nauru, Niue, Palau, Saint Kitts and Nevis, Saint Lucia, San Marino, Seychelles, Singapore, South Sudan, Tonga, Tuvalu. The population of these countries accounts for 0.3% of total population in WHO regions.

Data

1. Heat-related mortality data is provided by Indicator 1.1.6 in section 1.
2. Population in each country are taken from World Bank.[255]
3. GDP per capita in OECD members are taken from OECD statistics.[256]
4. VSL in OECD are taken from OECD report on Mortality Risk Valuation in Environment, Health and Transport Policies.[258]
5. Years of remaining life are obtained from WHO.[257]

Caveats

The caveats of this indicator would mainly be in two aspects. Since VSLY is derived from VSL, the uncertainties and ethical concerns on VSL mentioned in last year's caveats also applies to the usage of VSLY. On the other hand, here it is assumed the VSLY is constant at different ages, while some studies argue that the distribution of VSLY to age is Inverted-U shaped.[254] If people under 65 are also taken into account, then the Inverted-U assumption should be considered. The relationship between economic costs of heat-related mortality, per capita GNI across countries, and carbon emissions, was analysed and the correlations were not statistically significant. In the future, with heat-related mortality data with more detailed social groups aggregations, this indicator might explore further inequalities.

Indicator 4.1.3: Potential Loss of Earnings from Heat-Related Labour Capacity Reduction

Methods

Indicator 1.1.4 provides data on heat-related labour capacity loss, in terms of lost work hours, at country scale across four sectors (services, manufacturing, construction and agriculture) for the years 1990-2020 inclusive. In order to calculate potential loss of earnings from this labour capacity loss, it was necessary to compile a dataset of average earnings per hour for each of these countries, sectors and years.

Earnings and income statistics were compiled from the ILOSTAT databases held by the ILO, within the category 'Statistics on Wages'.[259] ILOSTAT includes a number of indicators which are of potential relevance to deriving the average annual hourly wages for the required countries and years. There are variations in the coverage of these indicators, with none having an entirely comprehensive coverage of the countries, sectors and years required for this indicator. Multiple ILOSTAT indicators were therefore used to fill as many gaps as possible. The three main indicator sets used were:

- Mean nominal monthly earnings of employees by sex and economic activity: annual
- Mean nominal monthly earnings of employees by sex and occupation: annual
- Mean nominal hourly earnings of employees by sex and occupation: annual

Within each of these indicator sets, the employment activities most accurately reflecting the four required sectors were selected. In some cases, more than one such activity was available, due to different reporting conventions (for example, the set of activities under ISCO-08 being an update from ISCO-88). Full descriptions of ILO indicators and classifications are available on the ILOSTAT website [260].

Each indicator and activity was available in US dollar and local currency units. US dollar units were preferred, however in each indicator and activity case, the number of returns in local currency units was slightly higher, so these were selected as well in case more data points could be covered by doing so.

The following tables set out for each of the four employment sectors, the ILOSTAT indicators and activity definitions that were selected in order to supply as much of used the required data as possible. In each table the indicator, activity and currency combinations are arranged in the order of preference with which they were used.

Table 30: Indicators, activity classes and currencies selected to gather data from the ILOSTAT databases on earnings in the services sector, in order of preference

	Indicator	Activity	Currency
1	Mean nominal monthly earnings of employees by sex and economic activity: annual	Aggregate: Trade, transportation, accommodation and food, and business and administrative services	US Dollars
2		Aggregate: Trade, transportation, accommodation and food, and business and administrative services	Local currency
3	Mean nominal monthly earnings of employees by sex and occupation: Annual	ISCO-08: 5. Service and sales workers	US Dollars
4		ISCO-08: 5. Service and sales workers	Local currency
5		ISCO-88: 5. Service workers and shop and market sales workers	US Dollars
6		ISCO-88: 5. Service workers and shop and market sales workers	Local currency
7	Mean nominal hourly earnings of employees by sex and occupation: Annual	ISCO-08: 5. Service and sales workers	US Dollars
8		ISCO-08: 5. Service and sales workers	Local currency
9		ISCO-88: 5. Service workers and shop and market sales workers	US Dollars
10		ISCO-88: 5. Service workers and shop and market sales workers	Local currency
11	Mean nominal monthly earnings of employees by sex and economic activity: annual	ISIC Rev.4: N. Administrative and support service activities	US Dollars
12		ISIC Rev.4: N. Administrative and support service activities	Local currency
13		ISIC Rev. 3.1: K. Real estate, renting and business activities	US Dollars
14		ISIC Rev. 3.1: K. Real estate, renting and business activities	Local currency
15		ISIC Rev.2: 8. Financing, insurance, real estate and business services	US Dollars
16		ISIC Rev.2: 8. Financing, insurance, real estate and business services	Local currency

Table 31: Indicators, activity classes and currencies selected to gather data from the ILOSTAT databases on earnings in the manufacturing sector, in order of preference

	Indicator	Activity	Currency
1	Mean nominal monthly earnings of employees by sex and economic activity: annual	Aggregate: Manufacturing	US Dollars
2		Aggregate: Manufacturing	Local currency
3		ISIC Rev.4: C. Manufacturing	US Dollars
4		ISIC Rev.4: C. Manufacturing	Local currency
5		ISIC Rev. 3.1: D. Manufacturing	US Dollars
6		ISIC Rev. 3.1: D. Manufacturing	Local currency
7		ISIC Rev.2: 3. Manufacturing	US Dollars
8		ISIC Rev.2: 3. Manufacturing	Local currency
9	Mean nominal monthly earnings of employees by sex and occupation: Annual	ISCO-08: 8. Plant and machine operators, and assemblers	US Dollars
10		ISCO-08: 8. Plant and machine operators, and assemblers	Local currency
11		ISCO-88: 8. Plant and machine operators and assemblers	US Dollars
12		ISCO-88: 8. Plant and machine operators and assemblers	Local currency
13	Mean nominal hourly earnings of employees by sex and occupation: Annual	ISCO-08: 8. Plant and machine operators, and assemblers	US Dollars
14		ISCO-08: 8. Plant and machine operators, and assemblers	Local currency
15		ISCO-88: 8. Plant and machine operators and assemblers	US Dollars
16		ISCO-88: 8. Plant and machine operators and assemblers	Local currency

Table 32: Indicators, activity classes and currencies selected to gather data from the ILOSTAT databases on earnings in the agricultural sector, in order of preference

	**Indicator**	**Activity**	**Currency**
1	Mean nominal monthly earnings of employees by sex and economic activity: annual	Aggregate: Agriculture	US Dollars
2		Aggregate: Agriculture	Local currency
3		ISIC Rev.4: A. Agriculture; forestry and fishing	US Dollars
4		ISIC Rev.4: A. Agriculture; forestry and fishing	Local currency
5		ISIC Rev.3.1: A. Agriculture, hunting and forestry	US Dollars
6		ISIC Rev.3.1: A. Agriculture, hunting and forestry	Local currency
7		ISIC Rev.2: 1. Agriculture, hunting, forestry and fishing	US Dollars
8		ISIC Rev.2: 1. Agriculture, hunting, forestry and fishing	Local currency
9	Mean nominal monthly earnings of employees by sex and occupation: Annual	ISCO-08: 6. Skilled agricultural, forestry and fishery workers	US Dollars
10		ISCO-08: 6. Skilled agricultural, forestry and fishery workers	Local currency
11		ISCO-88: 6. Skilled agricultural and fishery workers	US Dollars
12		ISCO-88: 6. Skilled agricultural and fishery workers	Local currency
13	Mean nominal hourly earnings of employees by sex and occupation: Annual	ISCO-08: 6. Skilled agricultural, forestry and fishery workers	US Dollars
14		ISCO-08: 6. Skilled agricultural, forestry and fishery workers	Local currency
15		ISCO-88: 6. Skilled agricultural and fishery workers	US Dollars
16		ISCO-88: 6. Skilled agricultural and fishery workers	Local currency

Table 33: Indicators, activity classes and currencies selected to gather data from the ILOSTAT databases on earnings in the manufacturing sector, in order of preference

	Indicator	Activity	Currency
1		Aggregate: Construction	US Dollars
2		Aggregate: Construction	Local currency
3		ISIC Rev.4: F. Construction	US Dollars
4		ISIC Rev.4: F. Construction	Local currency
5		ISIC Rev. 3.1: F. Construction	US Dollars
6		ISIC Rev. 3.1: F. Construction	Local currency
7	Mean nominal monthly earnings of employees by sex and economic activity: annual	ISIC Rev.2: 5. Construction	US Dollars
8		ISIC Rev.2: 5. Construction	Local currency
9		ISCO-08: 9. Elementary occupations	US Dollars
10		ISCO-08: 9. Elementary occupations	Local currency
11	Mean nominal monthly earnings of employees by sex and occupation: Annual	ISCO-88: 9. Elementary occupations	US Dollars
12		ISCO-88: 9. Elementary occupations	Local currency
13		ISCO-08: 9. Elementary occupations	US Dollars
14		ISCO-08: 9. Elementary occupations	Local currency
15	Mean nominal hourly earnings of employees by sex and occupation: Annual	ISCO-88: 9. Elementary occupations	US Dollars
16		ISCO-88: 9. Elementary occupations	Local currency

A spreadsheet tool was developed to select the relevant data points for all available countries in order of indicator preference – if there was no data point for a given country, year and sector in the first priority indicator, the data point was sought in the next indicator, and so on until a data point was found, or all indicators had been tried.

Monthly earnings data were converted to hourly values using a standard assumption of 40 hours per week and 4.33 weeks per month, i.e. 173.2 hours per month.

Data in nominal local currency units were converted to nominal US dollars at market exchange rates using IMF International Financial Statistics.[261] Nominal US dollar values were converted to real 2020 US dollar values using the US dollar consumer price index from the IMF World Economic Outlook database.[262]

Even after searching 16 variations of ILO indicator, activity and reporting currency for each sector (as shown in Table 30-Table 33), there were still considerable gaps, with around two thirds of required data points unfilled. In addition, there was a small number of clearly erroneous data points – e.g. with hourly earnings rates orders of magnitude too high, possibly caused by incorrect recording of the currency in which the data were reported, or by episodes of rapid inflation and currency devaluation, with which the recorded market exchange rates were not keeping track.

In order to fill the gaps with no data, as well as to correct data points that were clearly erroneous, a gap filling process was undertaken, using other data points to stand in for the missing or erroneous data. This process was undertaken after all of the data had been corrected to real 2020 US dollar values, so that all of the data were already expressed in constant values. Wherever possible, gaps were filled using data from a different year but from the same sector and country. As a first choice, data were backfilled using data from the nearest future year in the same sector and country. If no future year was available, data were filled using the nearest past year. If there were no data points available at all for a certain sector or country, the data were taken from the same sector of a different country that was as comparable as possible to the country with missing data. Identification of a reasonably comparable country was achieved primarily by selecting one as close as possible on the HDI scale. Ideally a country that was within five places on the HDI scale to the country with missing data, and also within the same or similar world region, and which had its own data points, was identified to provide the missing data. If there were no countries from a similar world region within 5 places of the HDI ranking of the country in question, the immediately closest country on the HDI scale was selected, regardless of its geographic proximity.

A small number of countries have not been given an HDI value and hence could not be included in the analysis.

This process resulted in estimates of hourly earnings for the four sectors, for the years 1990-2020 inclusive, for 186 countries. These hourly earnings data were multiplied by the corresponding values for work hours lost in each country, sector and year, to provide a quantification of potential earnings lost.

These total lost earnings were expressed as a percentage of the country's GDP in each relevant year. GDP data in nominal US dollars at market exchange rates were downloaded from the IMF World Economic Outlook database [262], and rendered in constant 2020 US dollars using the GDP deflator index from the same source. Gaps in this GDP data for some countries and years imposed a small further restriction on the coverage of this indicator, and not all of the same countries are available for all years. The maximum country-coverage of the indicator is 183 countries, during the years 2002-2019 inclusive.

Data

1. Data on working hours lost from indicator 1.1.4
2. Data on earnings by country and sector from ILOSTAT.[259]
3. Exchange rate data from IMF International Financial Statistics.[261]
4. US Dollar CPI and GDP deflator index from the IMF World Economic Outlook database.[262]
5. Country GDP data from the IMF World Economic Outlook database.[262]

Caveats

There are several important caveats associated with the analysis:

- The ILOSTAT data do not cover all of the countries, years and sectors required, hence some gap filling was required, as described above. Whilst reasonable care has taken to identify appropriate estimates, these gap filled data are subject to uncertainties.
- Whilst reasonable efforts have been made to correct for clearly erroneous data points, the analysis is dependent on the reliability of the ILOSTAT data, which could be subject to uncertainties in reporting, collection and processing.
- The use of different combinations of ILOSTAT indicators and activity classes, rather than one single indicator and one activity class per sector, was necessary to increase data coverage as much as possible. Nonetheless this entails risks of inconsistencies, for example associated with different classifications and reporting methods.
- The conversion of monthly data to hourly was carried out on the basis of a standard assumption of 4.33 weeks per month, and 40 hours per week. Real monthly working times will vary from these assumptions to a greater or lesser extent in different countries.

All of these issues mean that caution should be exercised when examining results for any particular country. In addition, it must be emphasised that the results produced are the *potential* loss of earnings, rather than actual. The indicator is not based on evidence as to whether time off work was in fact taken. Further, if time was taken off work, the bearer of the costs of the lost labour could have varied between countries and sectors. In some instances workers may have been able to claim sick pay, in which case the losses would have been borne by the employer through paying for non-productive time. In other instances, no arrangements for sick pay may have been in place, in which case it would have been the worker who would have borne the cost through a direct loss of earnings due to the inability to work.

Finally, the indicator by definition is an estimate of potential loss of earnings from formal paid sectors. In many countries informal and unpaid labour is also significant. Such activities could include domestic work and small-scale agriculture.[263-265] The impacts on productivity and health of extreme heat on workers involved in such so-called informal sectors, would be in addition to the monetised estimates quantified by this indicator.

Additional analysis

The main Lancet Countdown report text provides the results of the analysis for low HDI countries. The following graphs present the same analysis as applied to medium, high, and very high HDI countries.

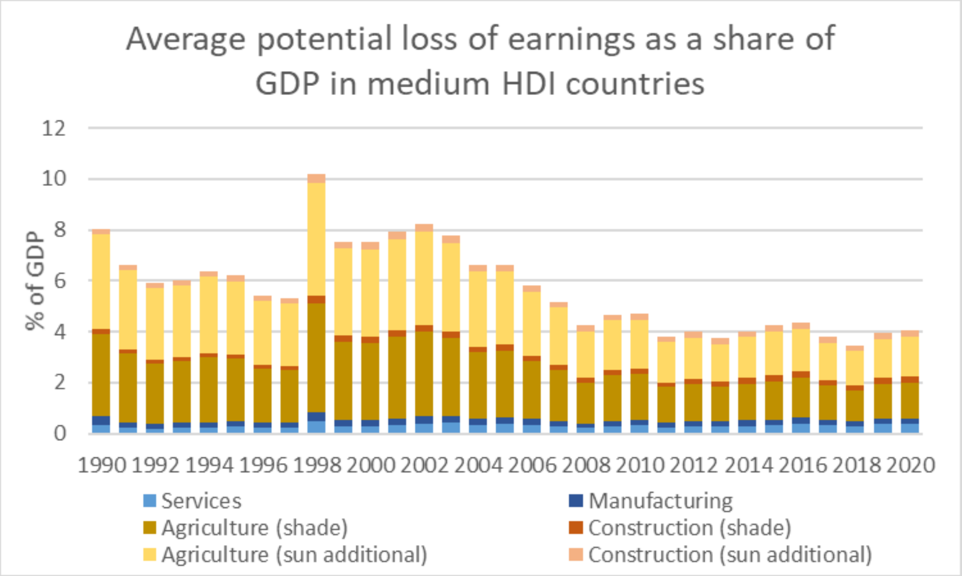

Figure 69. Average potential loss of earnings as a share of GDP in medium HDI countries, by sector of employment. The agriculture and construction (sun additional) blocks represent the losses that would have been incurred in addition to those from agriculture and construction (shade) if all of the activities in these sectors had been carried out in direct sunlight.

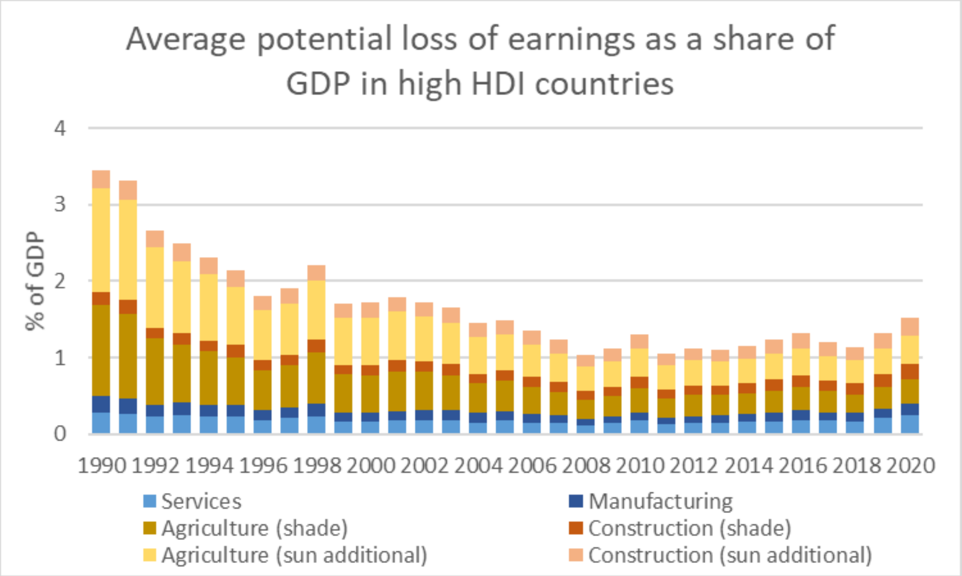

Figure 70. Average potential loss of earnings as a share of GDP in high HDI countries, by sector of employment. The agriculture and construction (sun additional) blocks represent the losses that would have been incurred in addition to those from agriculture and construction (shade) if all of the activities in these sectors had been carried out in direct sunlight.

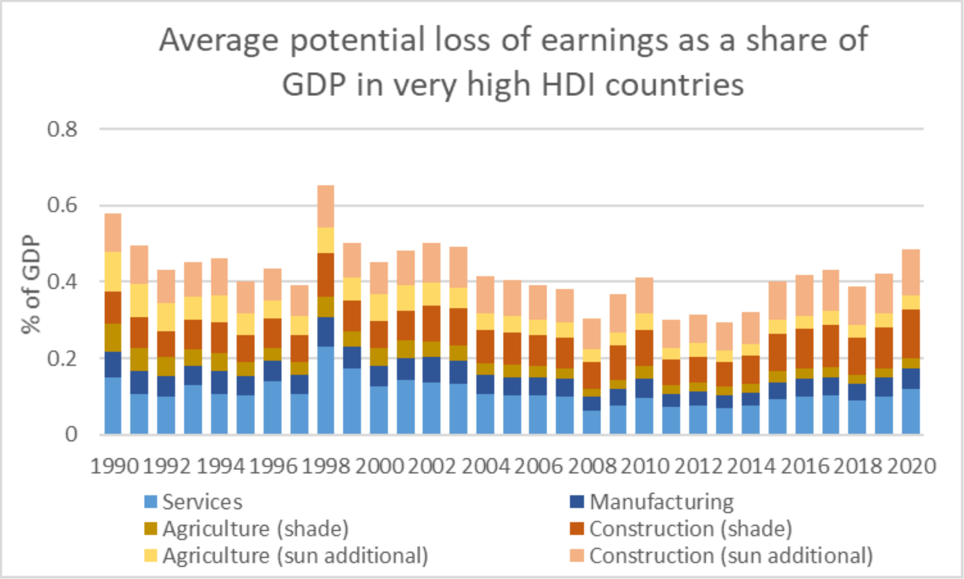

Figure 71. Average potential loss of earnings as a share of GDP in very high HDI countries, by sector of employment. The agriculture and construction (sun additional) blocks represent the losses that would have been incurred in addition to those from agriculture and construction (shade) if all of the activities in these sectors had been carried out in direct sunlight.

Indicator 4.1.4: Costs of the Health Impacts of Air Pollution

Methods

Indicator 3.3 provides data on deaths attributable to both natural and anthropogenic ambient air pollution. YLLs were calculated from the age-specific attributable deaths by summing over the remaining life expectancy at the age of death for each attributable death. To determine YLLs attributable to anthropogenic causes only, the total YLLs are reduced to the country- and year-specific proportion of total deaths attributable to anthropogenic sources only in Indicator 3.3. The YLLs calculated this way are a conservative estimate since the remaining life expectancy in real world conditions are used, rather than hypothetical clean conditions, which would be larger. YLLs were calculated for 137 individual countries, for 2015 and 2019. Each country was then classified according to both its HDI category and WHO region (see Table 34 and Table 35 below for country classifications). For the WHO region calculations, four 'rest of world' regions were also added (see Table 36). It was not possible to use these regions for the HDI classification, due the heterogeneity of classifications of the countries that constitute each region.

The YLLs for each category and region were then summed. To determine the economic value of the YLLs for each category and region relative to per capita average annual income in each, the results were multiplied by the fixed ratio of the VSLY to GDP per capita derived by Indicator 4.1.2. To calculate the economic value of the YLLs relative to total GDP for each year, the results of this first calculation were multiplied by average GDP per capita (calculated from the sum of GDP for each category and region, inflated to US$ 2020 from 2015 and 2019 current prices, divided by the sum of the population for each category and region,), and then divided by the sum of GDP in US$2020 for the category or region in question.

GDP and GDP inflator data were taken from the International Monetary Fund (IMF), and population data were taken from the UN. The data and methods used to calculate the fixed ratio between VSLY and GDP per capita are described in Indicator 4.1.2

Table 34. Countries in each HDI group included in the calculation of costs of air pollution.

HDI	Country
Very High	Argentina, Australia, Austria, Belarus, Belgium, Brunei, Darussalam, Bulgaria, Canada, Chile, Croatia, Cyprus, Czechia, Denmark, Estonia, Finland, France, Georgia, Germany, Greece, Hungary, Iceland, Ireland, Israel, Italy, Japan, Kazakhstan, Latvia, Lithuania, Luxembourg, Malaysia, Malta, Mauritius, Montenegro, Netherlands, New Zealand, Norway, Poland, Portugal, Republic of Korea, Romania, Russian Federation, Saudi Arabia, Serbia, Singapore, Slovakia, Slovenia, Spain, Sweden, Switzerland, Turkey, United Kingdom, United States of America, Uruguay
High	Albania, Algeria, Armenia, Azerbaijan, Bolivarian Republic of Venezuela, Bolivia, Bosnia and Herzegovina, Botswana, Brazil, China, Colombia, Ecuador, Egypt, Gabon, Indonesia, Islamic Republic of Iran, Libya, Mexico, Mongolia, North Macedonia, Paraguay, Peru, Philippines, Republic of Moldova, South Africa, Sri Lanka, Thailand, Tunisia, Ukraine, Vietnam
Medium	Angola, Bangladesh, Bhutan, Cabo Verde, Cambodia, Cameroon, Comoros, Congo, Equatorial Guinea, Eswatini, Ghana, India, Kenya, Kyrgyzstan, Lao People's Democratic Republic, Morocco, Myanmar, Namibia, Nepal, Pakistan, Zambia, Zimbabwe
Low	Afghanistan, Benin, Burkina Faso, Burundi, Central African Republic, Chad, Cote d'Ivoire, Democratic Republic of the Congo, Djibouti, Eritrea, Ethiopia, Gambia, Guinea, Guinea Bissau, Lesotho, Liberia, Madagascar, Malawi, Mali, Mauritania, Mozambique, Niger, Nigeria, Rwanda, Senegal, Sierra Leone, Sudan, Togo, Uganda, United Republic of Tanzania

Table 35. Countries in each WHO region group included in the calculation of costs of air pollution.

WHO	Country
African	Algeria, Angola, Benin, Botswana, Burkina Faso, Burundi, Cabo Verde, Cameroon, Central African Republic, Chad, Comoros, Congo, Cote d'Ivoire, Democratic Republic of the Congo, Equatorial Guinea, Eritrea, Eswatini, Ethiopia, Gabon, Gambia, Ghana, Guinea, Guinea Bissau, Kenya, Lesotho, Liberia, Madagascar, Malawi, Mali, Mauritania, Mauritius,

	Mozambique, Namibia, Niger, Nigeria, Rwanda, Senegal, Sierra Leone, South Africa, Togo, Uganda, United Republic of Tanzania, Zambia, Zimbabwe
Americas	Argentina, Bolivarian Republic of Venezuela, Bolivia, Brazil, Canada, Chile, Colombia, Ecuador, Mexico, Paraguay, Peru, United States of America, Uruguay
Eastern Mediterranean	Afghanistan, Djibouti, Egypt, Islamic Republic of Iran, Libya, Morocco, Pakistan, Saudi Arabia, Somalia, Sudan, Tunisia
European	Albania, Armenia, Austria, Azerbaijan, Belarus, Belgium, Bosnia and Herzegovina, Bulgaria, Croatia, Cyprus, Czechia, Denmark, Estonia, Finland, France, Georgia, Germany, Greece, Hungary, Iceland, Ireland, Israel, Italy, Kazakhstan, Kyrgyzstan, Latvia, Lithuania, Luxembourg, Malta, Montenegro, Netherlands, North Macedonia, Norway, Poland, Portugal, Republic of Moldova, Romania, Russian Federation, Serbia, Slovakia, Slovenia, Spain, Sweden, Switzerland, Turkey, Ukraine, United Kingdom
South-East Asian	Bangladesh, Bhutan, Democratic People's Republic of Korea, India, Indonesia, Myanmar, Nepal, Sri Lanka, Thailand
Western Pacific	Australia, Brunei Darussalam, Cambodia, China, Japan, Lao People's Democratic Republic, Malaysia, Mongolia, New Zealand, Philippines, Republic of Korea, Singapore, Vietnam

Table 36. Countries in each 'rest of word' region group included in the calculation of costs of air pollution under WHO calculations.

Region	WHO	Country
1	Americas	Aruba, Barbados, Bahamas, Cuba, Dominican Republic, Grenada, French Guiana*, Guadeloupe*, Guyana, Haiti, Jamaica, Saint Lucia, Martinique*, Puerto Rico*, Suriname, Trinidad and Tobago, Saint Vincent and the Grenadines, United States Virgin Islands*
2	Americas	Belize, Costa Rica, Guatemala, Honduras, Nicaragua, Panama, El Salvador
3	Eastern Mediterranean	Tajikistan, Turkmenistan, Uzbekistan
4	European	United Arab Emirates, Bahrain, Iraq, Jordan, Kuwait, Lebanon, Oman, Occupied Palestinian Territory, Qatar, Syrian Arab Republic*, Yemen

*Population and GDP excluded from the calculations due to lack of data of either one or other data point.

Data

1. IMF World Economic Outlook (April 2021).[252]
2. UN World Population Prospects 2019.[192]

Caveats

See Indicator 3.3, for caveats related to the calculation of reduced life expectancy.

Caveats regarding the calculation of VSLY are discussed under Indicator 4.1.2. Countries not listed in the tables above have been excluded from the analysis, due to the lack of individual characterisation in the model used to calculate YLLs. Democratic People's Republic of Korea is excluded from the WHO regional analysis due to the lack of reliable GDP data (and is not classified under the HDI). Somalia is excluded from the HDI analysis, as it is not classified.

Additional analysis

Table 37 and Table 38 tabulates the results for each approach, for 2015 and 2019, for the HDI classification and WHO regions, respectively.

Table 37. Economic value of YLLs attributable to $PM_{2.5}$ exposure from anthropogenic sources by 2019 HDI group

	Relative to average annual per-capita income		GDP-equivalent	
	(2015)	(2019)	(2015)	(2019)
Very High	38,119,789	35,347,820	2.6%	2.3%
High	100,231,280	99,123,204	3.6%	3.5%
Medium	74,645,528	78,128,953	3.8%	3.8%
Low	12,421,050	12,624,065	1.6%	1.5%

Table 38: economic value of YLLs attributable to $PM_{2.5}$ exposure from anthropogenic sources by WHO region

	Relative to average annual per-capita income		GDP-equivalent	
	(2015)	(2019)	(2015)	(2019)
African	13,558,069	13,697,617	1.4%	1.3%
Americas	8,418,635	8,283,630	0.9%	0.8%
Eastern Mediterranean	17,562,262	18,054,689	2.7%	2.6%
European	34,966,559	32,265,274	3.8%	3.5%
South-East Asian	72,406,251	76,853,581	3.8%	3.9%
Western Pacific	81,611,316	79,719,386	4.4%	4.2%

4.2: The Economics of the Transition to Zero-Carbon Economies

Indicator 4.2.1: Clean energy investment

Investment in New Coal Capacity

Methods

The data for investment in new coal-fired power plants is sourced from the IEA World Energy Investment 2021 report.

'Investment' is defined as ongoing capital spending on assets. For investment in new coal-fired power plants this investment is spread out evenly from the year in which a new plant or upgrade of an existing one begins its construction to the year in which it becomes operational. This definition applies to 2017 data onwards and differs from the definition previously employed by the IEA, in which investment was defined as overnight capital expenditure.

Data reported in previous Lancet Countdown reports for global investment may have been updated with improved data. As investment in new coal capacity in South Africa was zero in 2006, a low positive value was entered to allow an index for future years to be calculated. Actual data cannot be reported for confidentiality reasons.

Data

1. IEA World Energy Investment 2021.[266]

Caveats

Other areas of expenditure, including operation and maintenance, research and development, financing costs, mergers and acquisitions or public markets transactions, are not included. Investment estimates are derived from IEA data for energy demand, supply and trade, and estimates of unit capacity costs. For more information, see IEA World Energy Investment 2021.

Additional analysis

Table 39: Annual investment in new coal-fired power capacity 2006-2020 (an index score 100 corresponds to 2006 levels)

	2006	2007	2008	2009	2010	2011	2012	2013	2014	2015	2016	2017	2018	2019	2020
**Global**	100	106	111	118	124	126	123	115	106	96	88	80	77	72	63
**China**	100	95	91	84	79	78	78	78	75	66	54	44	35	26	25
**USA**	100	121	130	120	89	57	29	7	2	0	0	0	0	5	13
**EU**	100	128	148	172	216	248	248	220	196	152	96	72	76	76	72
**India**	100	185	254	376	466	436	371	290	231	203	188	180	188	190	112
**Japan**	100	93	113	133	113	100	107	80	67	107	140	147	147	127	247
**Republic of Korea**	100	89	56	28	31	89	144	142	131	117	97	50	25	22	22
**Russia**	100	167	200	233	367	500	500	467	400	467	500	600	800	933	1300
**Southeast Asia**	100	85	122	181	285	385	367	348	326	296	315	348	363	352	204
**South Africa**	100	500	1000	1000	1000	1000	1000	1000	1000	900	1100	1300	1200	1200	800
**Brazil**	100	150	250	300	300	250	200	100	0	50	50	50	50	50	50

Investments in Zero-Carbon Energy and Energy Efficiency

Methods

The data for this indicator are sourced from the annual IEA World Energy Investment publication. Five categories of investment are defined:

***Hydropower* –** investment in small, large and pumped-hydropower.

Bioenergy – investment in bioenergy.

Other Renewables – investment in all other renewable energy (excluding hydropower and bioenergy), and renewable transport and heating (including solar thermal heating).

Nuclear Power – investment in nuclear power.

Energy Efficiency – See below

Electricity Networks & Storage – investment in electricity transmission and distribution infrastructure, and battery storage (excludes pumped-hydro).

Fossil Fuels – including oil, gas and coal, upstream mining, drilling and pipeline infrastructure, and coal, gas and oil power and other fossil fuel-based energy generation capacity.

For most sectors, 'investment' is defined as ongoing capital spending on assets. For some sectors, such as power generation, this investment is spread out evenly from the year in which a new plant or upgrade of an existing one begins its construction to the year in which it becomes operational. For other sources, such as upstream oil and gas and liquefied natural gas (LNG) projects, investment reflects the capital spending incurred over time as production from a new source ramps up or to maintain output from an existing asset. This definition and differs from the definition previously employed by the IEA before 2019, in which investment was defined as overnight capital expenditure. Investment data are inflated from USD 2019 to USD 2020 using GDP inflator taken from the International Monetary Fund (IMF)

Data

1. IEA World Energy Investment 2021.[266]
2. IMF World Economic Outlook (April 2021).[252]

Caveats

Other areas of expenditure, including operation and maintenance, research and development, financing costs, mergers and acquisitions or public markets transactions, are not included. Investment estimates are derived from IEA data for energy demand, supply and trade, and estimates of unit capacity costs. For more information, see IEA World Energy Investment 2021.

Additional analysis

Table 40: Energy investments 2015-2020, billions US$2020.

	**2015**	**2015**	**2016**	**2017**	**2018**	**2020**
Fossil Fuels	1,165	986	985	989	1,000	736
Nuclear	57	53	51	54	53	52
Hydropower	38	39	39	37	35	36
Bioenergy	256	264	262	269	288	308
Other Renewables	29	33	35	33	40	42
Electricity Networks & Storage	303	314	307	301	277	269
Energy Efficiency	258	283	272	273	272	270
**Total**	**2,106**	**1,973**	**1,952**	**1,955**	**1,964**	**1,712**

Indicator 4.2.2: Employment in Low-Carbon and High-Carbon Industries

Methods

The data for this indicator is sourced from IRENA (renewables) and IBISWorld (fossil fuel extraction).[267,268] Renewable industries included are:

- Large hydropower;
- Solar heating/cooling;
- Solar photovoltaic;
- Wind energy;
- Bioenergy;
- Other technologies.

Bioenergy includes liquid biofuels, soil biomass and biogas. ‘Other technologies’ includes geothermal energy, ground-based heat pumps, concentrated solar power, municipal and industrial waste, and ocean energy. Fossil fuel extraction values include direct employment, whereas renewable energy jobs include direct and indirect employment (e.g. equipment manufacturing), except for large hydropower (direct employment only).

Due to an improvement in data collection and estimation methodology, employment values reported for fossil fuel extraction are in some years substantially higher than those reported in the 2018 Lancet Countdown report. Similarly, an improvement to the methodology for estimating hydropower has altered historic values for Hydropower (previously called ‘large’ hydropower), and Other Technologies (which previously included small hydropower). For the 2018 data, ‘Other Technologies’ now also includes employment related to ground-based heat pumps.

Data

1. Data for employment in renewables from IRENA. Data are available through to 2018.
2. Data for employment in fossil fuel extraction from IBISWorld: oil and gas exploration and production; and coal mining.[267,268]

Caveats

Fossil fuel extraction values include direct employment, whereas renewable energy jobs include direct and indirect employment (e.g. equipment manufacturing).

Additional analysis

Table 41: Employment in renewable energy and fossil fuel extraction industries.

	Million Jobs								
	2012	2013	2014	2015	2016	2017	2018	2019	2020
Hydropower	1.66	2.21	2.04	2.16	2.06	1.99	2.05	1.96	
Other Technologies	0.22	.023	0.19	0.2	0.24	0.16	0.18	0.13	
Solar Heating/Cooling	0.89	0.5	0.76	0.94	0.83	0.81	0.8	0.82	
Wind Energy	0.75	0.83	1.03	1.08	1.16	1.15	1.16	1.17	
Bioenergy	2.4	2.5	2.99	2.88	2.74	3.06	3.18	3.58	
Solar Photovoltaic	1.36	2.27	2.49	2.77	3.09	3.37	3.61	3.76	
Fossil Fuel Extraction	11.95	12.25	12.50	12.41	12.36	11.73	11.67	11.61	9.93

Bioenergy includes liquid biofuels, soil biomass and biogas. ‘Other technologies’ includes geothermal energy, ground-based heat pumps, concentrated solar power, municipal and industrial waste, and ocean energy. Fossil fuel extraction values include direct employment, whereas renewable energy jobs include direct and indirect employment (e.g. equipment manufacturing), except for large hydropower (direct employment only).

Fossil fuel extraction data have been updated for all years.

Indicator 4.2.3: Funds Divested from Fossil Fuels

Methods

The data for this indicator is collected and provided by 350.org.[269] They represent the total assets (or assets under management, AUM) for institutions that have publicly committed to divest (for which data are available), with non-US$ values converted using the market exchange rate when the commitment was made, and thus do not directly represent the actual sums divested from fossil fuel companies. A company is committed to 'divestment' if it falls into any of the following five categories:

- 'Fossil Free' - An institution or corporation that does not have any investments (direct ownership, shares, commingled mutual funds containing shares, corporate bonds) in fossil fuel companies (coal, oil, natural gas) and committed to avoid any fossil fuel investments in the future

- 'Full' - An institution or corporation that made a binding commitment to divest (direct ownership, shares, commingled mutual funds containing shares, corporate bonds) from any fossil fuel company (coal, oil, natural gas).

- 'Partial' - An institution or corporation that made a binding commitment to divest across asset classes from some fossil fuel companies (coal, oil, natural gas), or to divest from all fossil fuel companies (coal, oil, natural gas), but only in specific asset classes (e.g. direct investments, domestic equity).

- 'Coal and Tar Sands' - An institution or corporation that made a binding commitment to divest (direct ownership, shares, commingled mutual funds containing shares, corporate bonds) from any coal and tar sands companies.

- 'Coal only' - An institution or corporation that made a binding commitment to divest (direct ownership, shares, commingled mutual funds containing shares, corporate bonds) from any coal companies.

Eight organisations that were originally recorded as non-healthcare institutions have been considered as such for the purpose of this indicator (London School of Hygiene and Tropical Medicine, The Royal College of General Practitioners, New Zealand Nurses Organisation, HESTA, HCF, Berliner Ärzteversorgung, Doctors for the Environment Australia, and the Royal College of Emergency Medicine). Divestment commitments by the American Medical Association, which divested in 2018, was not included in the data provided by 350.org, and was added separately.

Data

1. 350.org Divestment Commitments dataset.[269]

Caveats

Data on the number of institutions that have divested and the value of their assets is dependent on institutions reporting this information to 350.org.

Additional analysis

The cumulative value of divestment (both global total and for healthcare institutions) is presented below (Table 42). Organisations that have divested but for which no date of divestment has been recorded (a total of $504.42 million) are recorded in a separate column, with the total assumed to begin in 2008.

Table 42. Cumulative fossil fuel divestment.

	US$ billion (current prices)		
	**Global**	**Global (including data with no divestment date)**	**Healthcare Institutions**
2008	$ 0.00	$ 504.42	$ -
2009	$ 0.00	$ 504.42	$ -
2010	$ 0.00	$ 504.42	$ -
2011	$ 0.09	$ 504.51	$ -
2012	$ 2.11	$ 506.53	$ -
2013	$ 16.13	$ 520.54	$ -
2014	$ 303.46	$ 807.87	$ 27.82
2015	$ 2,997.82	$ 3,502.24	$ 27.94
2016	$ 4,079.53	$ 4,583.95	$ 30.34
2017	$ 5,366.86	$ 5,871.27	$ 41.04
2018	$ 7,502.16	$ 8,006.57	$ 41.90
2019	$ 11,513.63	$ 12,018.05	$ 41.92
2020	$ 14, 012.50	$ 14,516.92	$ 41.97

Due to confidentiality issues, the full dataset is not available for publication. However, interested readers may visit the 350.org website for further information.

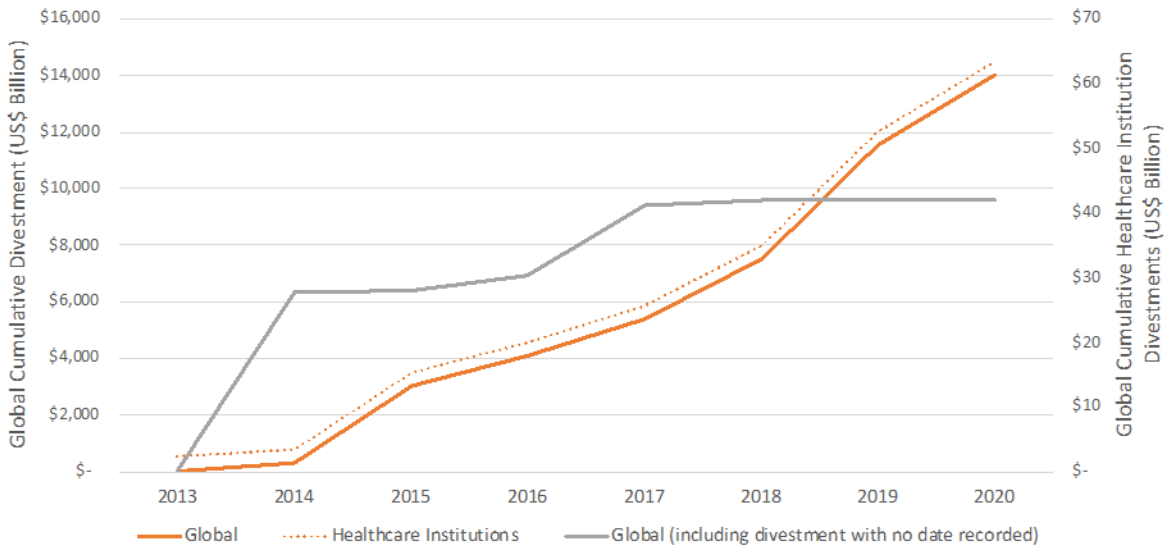

Figure 72. Cumulative divestment – Global total and in healthcare institutions

Indicator 4.2.4: Net Value of Fossil Fuel Subsidies and Carbon Prices

Methods

Fossil Fuel Subsidies

Data for fossil fuel subsidies were taken from two sources. The IEA provides data on fossil fuel consumption subsidies for 42 countries,[270] calculated using its 'price gap' approach – the difference between the end-user prices paid for fossil fuels in the country, and reference prices that account for the full cost of supply.[271] However, the countries provided in this list are mainly non-OECD. The OECD itself provides estimates of fossil fuel subsidies within the 37 OECD countries, plus Argentina, Armenia, Azerbaijan, Belarus, Brazil, China, Georgia, India, Indonesia, Moldova, Russia, South Africa and Ukraine - a total of 50 countries.[272] OECD's estimates are derived from a bottom-up inventory of subsidy mechanisms within each country, and include production and consumption support, infrastructure investments, incentives and R&D. It divides the type of support into three broad categories: Consumer Support Estimate (CSE), Producer Support Estimate (PSE) and General Services Support Estimate (GSE).

Combining the IEA and OECD datasets allows a coverage of 80 countries, after accounting for overlaps and the omission of countries not covered by the Lancet Countdown. The OECD describes an approach for combining these two datasets, and reconciling different estimates for the countries covered by both.[273] This involves selecting line items in the OECD inventory that correspond to the price-gap definition of subsidies that is the basis of the IEA data – i.e. measures that bring about reduced consumer prices: 'conceptually, an OECD estimate derived from individual measures that capture transfers to consumers from producers and taxpayers should match the IEA price-gap estimates' (p.22-3).[273]

The description of this approach suggests that in the few cases of countries whose subsidies have been calculated by both OECD and IEA, the OECD estimate would be expected to be the larger of the two.[273] However, analysis of overlapping countries suggests that it is in fact more often the IEA estimate that is larger. This analysis is described in more detail in the appendix of the 2020 Lancet Countdown report. The conclusion drawn from this is that attempting to separate some line items from the OECD estimates that seem more directed at consumers is not a reliable way of reconciling the two estimates – on the contrary, in several cases it makes the gap between the two larger by making the OECD estimate smaller. Consequently, in considering countries that overlap between the two datasets as part of preparing this indicator, a comparison was made simply between the total OECD estimate and the total IEA estimate.

Following a simple rule of thumb proposed by OECD, in order to decide which estimate to use in overlapping cases, the source that produces the larger cumulative total for a given country over the years being considered, was the one chosen as the source for that country for this indicator [273].

Carbon prices and revenues

Information on carbon prices and carbon pricing revenues was sourced from the World Bank Carbon Pricing Dashboard.[274] Revenues from each recorded instrument were allocated to the nation state within which the instrument operated. Shares of the EU ETS revenues were allocated to each of the participants in the EU ETS – that is the 28 members of the EU (which included the UK for the years considered in this analysis), plus Iceland and Norway. Liechtenstein is also an EU ETS member but could not be included in this analysis due to lack of CO_2 emissions data. The allocation of EU ETS revenues was made to participating states on the basis of their share of the emissions of all EU ETS states, calculated using IEA CO_2 emissions data.[275] This was considered an acceptable simplification given that for the period 2013-2020, 88% of allowances were allocated for auction to participating states in proportion to their emissions.[276]

Countries were included in the analysis if data were available for CO_2 emissions, and either fossil fuel subsidies or carbon pricing instruments. This yielded a list of 84 countries, accounting for 92% of global CO_2 emissions in 2018.[275]

Net carbon price and revenue calculations

In reality at present, both carbon prices and fossil subsidies are typically applied to individual sectors or fuels, and do not cover the entire economy. Within different particular jurisdictions the sectors covered by subsidies and

carbon prices are often not identical. As such the only way of producing a consistent indicator across multiple countries was to average out both subsidies and prices across the CO_2 emissions of the whole economy, resulting in net average economy-wide carbon prices and revenues. Each country's total fossil fuel subsidies were subtracted from its total carbon price revenues to produce a net carbon revenue. These figures were divided by the relevant total country CO_2 emissions for each year, using data from the IEA,[275] resulting in the net carbon price. The net carbon revenue was expressed as a proportion of national expenditure on health, using current annual (i.e. not including capital) health expenditure data from the WHO's Global Health Expenditure Database.[277]

Currency standardisation

All money values are expressed in real 2020 US$. Both the OECD Inventory and the IEA fossil fuel subsidy database provide data in real 2019 US$. These units were corrected to real 2020 values, using the GDP deflator for the US dollar, from the IMF.[262] The World Bank carbon pricing revenue data and the WHO health expenditure data are given in nominal US dollars, so again the US GDP deflator from IMF[262] was applied to correct to real 2020 values.

Data

1. Fossil fuel subsidies data from the IEA [270] and OECD.[272]
2. Carbon pricing data from the World Bank Carbon Pricing Dashboard.[274]
3. CO_2 emissions from fuel combustion from IEA.[275]
4. Health expenditure data from WHO.[277]
5. US Dollar GDP deflator index from the IMF World Economic Outlook database.[262]

Caveats

The principal caveat is that the indicator is strongly dependent on the reliability of the main datasets from the IEA, OECD and World Bank. It is possible that data on individual countries may not be fully comprehensive due to reporting errors, lack of information or other issues, as indeed is acknowledged by OECD.[273] The indicator should be considered as a way of illustrating global trends, and caution should be exercised in attempting to draw out specific conclusions relating to individual countries covered by the indicator.

The nature of indicators that draw on multiple datasets is that the most recent year on which they can report is defined by the most recent year that is common to all datasets used. In this case that year was 2018, which was due to this being the most recent complete year for both CO_2 emissions from fuel combustion and health expenditure.

The economy-wide net carbon price was derived by dividing fossil fuel subsidies and carbon pricing revenues by total CO_2 emissions. This fits well with the subsidies, as these are for fossil fuels, the principal source of CO_2. However, some of the carbon pricing instruments from which the revenue was assessed are not only for fossil fuel combustion but apply to other sectors and non-CO_2 gases. There is therefore a slight inconsistency between the sectoral coverage of the subsidies and the carbon pricing instruments.

Additional analysis

The relevant section in the main report shows net carbon prices, net carbon revenues, and net carbon revenues as a proportion of health spending, by 2019 HDI grouping, for the year 2018. The following graphs show results for the same three indicators with all countries grouped together, and for the years 2010-2018 inclusive. Results for years 2016-2017 differ from those reported in the 2020 Countdown report due to an increased number of countries included in the analysis.

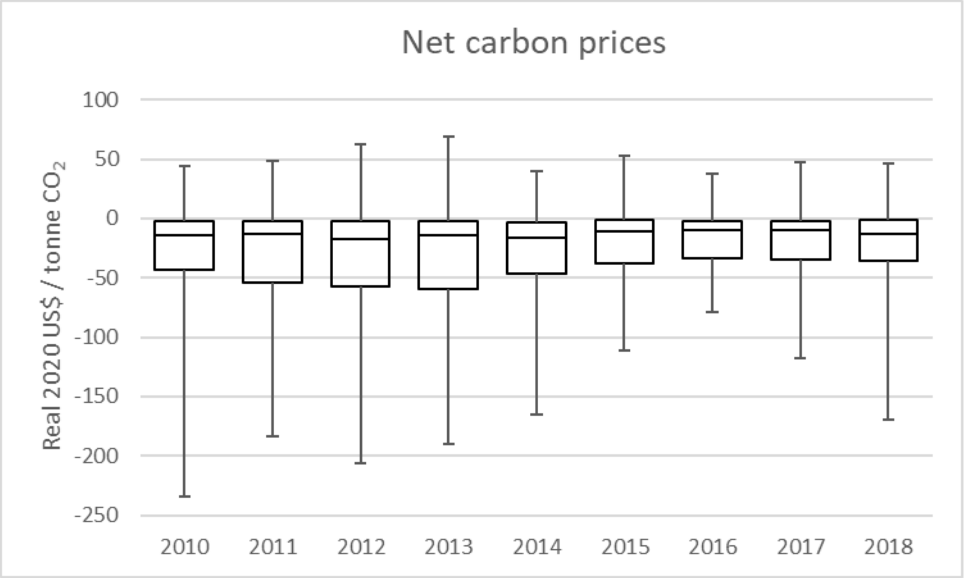

Figure 73. Net carbon prices for all countries included in the analysis, 2010-2018 inclusive. Boxes show the interquartile range (IQR), horizontal lines inside the boxes show the medians, and the brackets represent the full range from minimum to maximum.

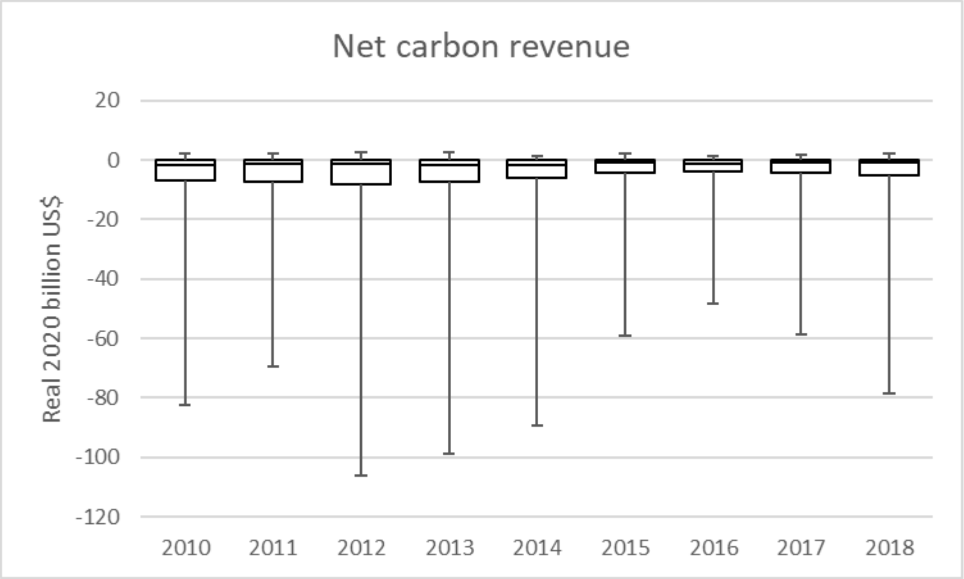

Figure 74. Net carbon revenue for all countries included in the analysis, 2010-2018 inclusive. Boxes show the interquartile range (IQR), horizontal lines inside the boxes show the medians, and the brackets represent the full range from minimum to maximum.

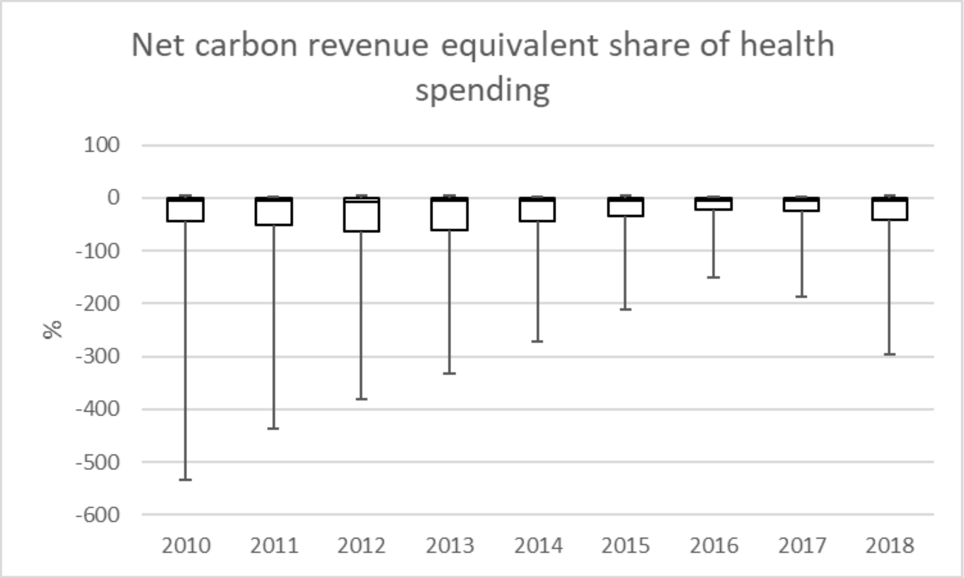

Figure 75. Net carbon revenue expressed as the equivalent share of current (i.e. not capital) annual health spending, for all countries included in the analysis, 2010-2018 inclusive. Boxes show the interquartile range (IQR), horizontal lines inside the boxes show the medians, and the brackets represent the full range from minimum to maximum.

Indicator 4.2.5: Production and Consumption-based Attribution of CO_2 and $PM_{2.5}$ Emissions

Methods

Environmentally Extended Multi-Regional Input-Output Analysis

There are two approaches to measure emissions: production-based (sometimes referred to as territorial-based) accounting and consumption-based accounting. Production-based emissions occur within the geographical territory of a nation, while consumption-based emissions encompass the emissions from the nation's domestic final consumption, as well as those caused by the production of its imports. Since both CO_2 emissions via climate change, and air pollution directly, are detrimental to human health, understanding of the responsibilities of emissions across borders is crucial in the globalised world. This indicator estimates $PM_{2.5}$ and CO_2 emissions embodied in international trade, and then calculates national $PM_{2.5}$ and CO_2 emissions from the consumption perspective. Thus, the responsibility of these emissions and the associated environmental and human health consequences can be distributed for international environmental policy formulation.

Environmentally extended multi-regional input-output (EEMRIO) analysis is used in the calculation of consumption-based emissions.[278] The EEMRIO analysis can reflect production and consumption structures and interdependencies between economic sectors across regions. The relationships between final use and emissions are estimated via Leontief inverse matrix, which is expressed as follows in equation (1):

$$\mathrm{C} = \mathrm{E} \cdot \mathrm{L} \cdot \mathrm{F} = \mathrm{E} \cdot (\mathrm{I} - \mathrm{A})^{-1} \cdot \mathrm{F} \qquad (1)$$

C is the total consumption-based emissions, CO_2 or $PM_{2.5}$ emissions in our case. It is mapped directly to emissions inventories. *E* is the row vector of the production-based emission intensity defined as the emissions per unit of output. *F* is the vector of final demand. and *L* is the Leontief inverse matrix calculated by $(I\text{-}A)^{-1}$, where *I* is the identity matrix, and *A* is the technical coefficient matrix describing the inter-sectoral and inter-regional flows per unit of output.

Consumption-based accounting encompasses emissions from domestic final consumption and those caused by the production of its imports, while production-based accounting measures emissions which take place within national territory. The above relationship can also be expressed as follows:

$$\mathrm{C_{CBA}} = \mathrm{C_{PBA}} - \mathrm{C_{exp}} + \mathrm{C_{imp}} \qquad (2)$$

where C_{CBA} is the consumption-based emissions, C_{imp} is the emissions embodied in imports, C_{PBA} is the production-based emissions, and C_{exp} is the emissions embodied in exports.

Emission Inventory Mapping with GAINS

To construct the production-based $PM_{2.5}$ emission inventory with the GAINS model, the workflow illustrated in Figure 76 is followed. First, an intermediary aggregation level to which emissions from the GAINS source categories are aggregated is defined.[1] In a second step these aggregated or grouped emissions are distributed among the relevant MRIO sectors according to a specific rule. This process is repeated until the emissions from all relevant GAINS source categories have been mapped to the relevant MRIO sectors.

[1] In most cases GAINS sectors are used. However, in a few cases the relevant source categories are sector-fuel combinations in the GAINS system: for example, in the power plant sectors, coal-, oil-, gas-, and biomass-fired plants are distinguished [and combustion free generation] so as to be able to map directly to the corresponding MRIO sectors.

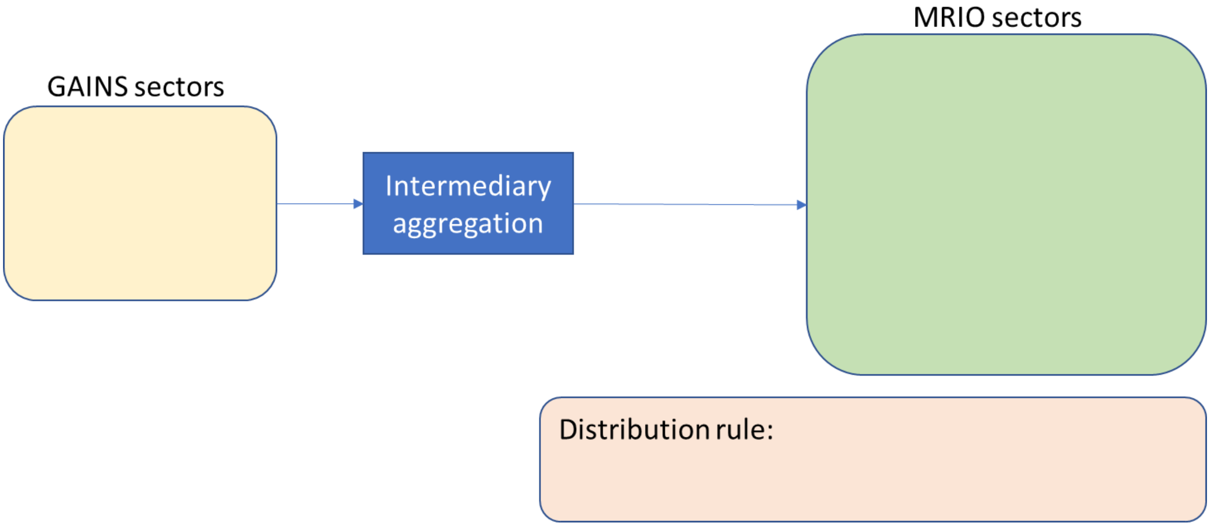

Figure 76. Generic approach for mapping the GAINS sectoral emissions to MRIO sectors.

In practice, the GAINS source categories are clustered into three groups, so that there are three rounds of mappings. These groupings correspond to energy-related emissions (except trucking, see below), process-related emissions, and trucking-related emissions. In a final step, for each MRIO sector the contributions from the three rounds of mappings are summed so that a total emission can be associated with each MRIO sector. In all calculations determining the relative energy share of an MRIO sector in the total energy, the use of electricity is ignored, since the emissions from electricity production are accounted for elsewhere.

On the GAINS side, trucking is related to the sectors TRA_RD_HDT and TRA_RD_LD4T and the fuel-related activities, such as diesel, gasoline, LPG etc, as well as km-related emissions such as abrasion, tyres and braking. On the MRIO side, diesel consumption from road transport by MRIO sector is used to determine the share of each sector in the total. In some countries significant amounts of diesel is also used by cars, a fact that is neglected here. Figure 77 illustrates the mapping process for trucking-related emissions between GAINS and the MRIO sectors.

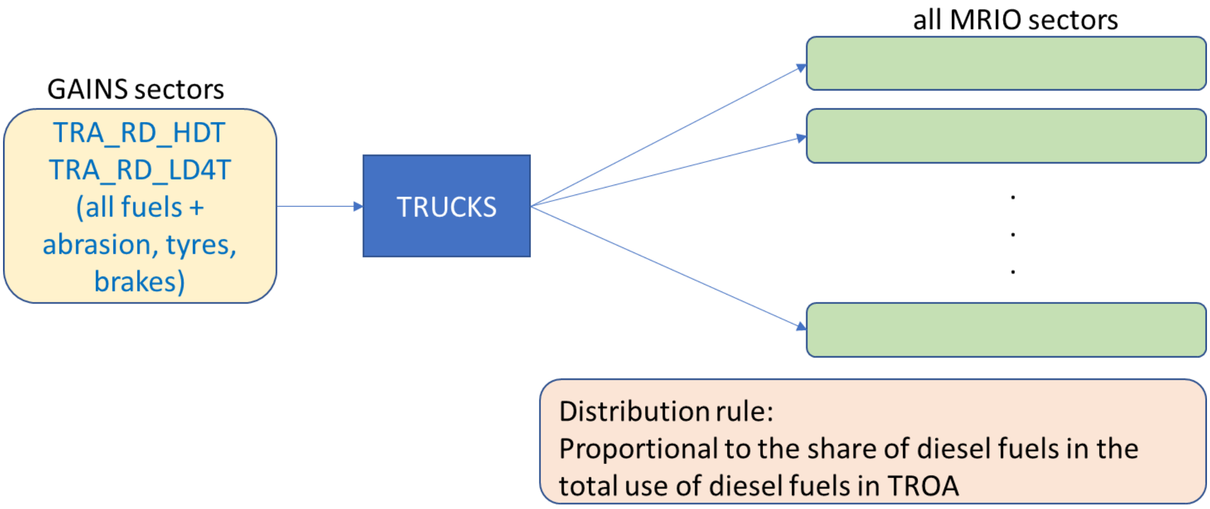

Figure 77 Mapping of trucking-related emissions

The trucking-related emissions in region *r* for MRIO sector *m* are thus calculated as:

$$\mathrm{Em}_r(m,t) = \mathrm{Em}_r(\mathrm{TRUCKS}) \cdot \mathrm{sh}_r(m,\mathrm{diesel}) \qquad (3)$$

where

$$\mathrm{sh}_r(m,\mathrm{diesel}) = \frac{\mathrm{TROA}_r(m,\mathrm{diesel})}{\sum_{m'} \mathrm{TROA}_r(m',\mathrm{diesel})} \qquad (4)$$

is the share of sector m in the road transport related diesel consumption in region r, and $\mathrm{Em}_r(\mathrm{TRUCKS})$ are the total trucking related emissions in region r as calculated by GAINS.

Once the trucking-related emissions and energy use has been separated out what is relevant for distributing the remaining energy (but not trucking-related emissions) is generally the total final energy consumption minus the diesel consumption in TROA. Thus, non-trucking related final energy consumption excluding electricity is referred to as the relevant final energy consumption in each MRIO sector that is used to determine the shares for distributing energy-related emissions into MRIO sectors.

In the mapping of energy-related emissions, intermediary clusters for energy-related emissions are defined as follow:

Table 43. Aggregated energy-related sectors, their description and coverage in terms of GAINS sectors as well as MRIO clusters.

**Label**	**Description**	**GAINS sector coverage**	**MRIO clusters**
ELE_COAL	Coal-fired power plants	All power plants combusting coal or solid biomass[2]	coal_electricity
ELE_OIL	Oil-fired power plants	All power plants combusting heavy fuel oil or diesel	oil_electricity
ELE_GAS	Gas-fired power plants	All power plants combusting natural gas	gas_electricity
AGR_MACH	Agricultural machinery	TRA_OT_AGR, DOM_OTH	cultivation + livestock_farming + items_dom_oth
IND_IS	Iron and steel industry	IN_OC_ISTE	manuf_is
IND_NFME	Non-ferrous metals	IN_OC_NFME	manuf_nfme
IND_NMMI	Non-metallic minerals	IN_OC_NMMI[3]	manuf_bricks + manuf_cem + manuf_nmmi
IND_CHEM	Chemical industries	IN_BO_CHEM, IN_OC_CHEM	manuf_chem + manuf_fert + manuf_chem_nec
IND_CON	Conversion industries, incl. refineries	IN_BO_CON, CON_COMB	ind_conversion
PPAPER	Pulp and paper	IN_BO_PAP, IN_OC_PAP	manuf_paper
OTH_IND	Other industries	All IN_XX_OTH sectors	other_industries
SERVICES	Services	DOM_COM subsectors, MSW	items_services
RAIL	Trains	TRA_OT_RAI	rail
Ships	Sea-going ships	TRA_OTS_X	ships
INW	Ships on inland waterways	TRA_OT_INW	inw
CONSTRUCTION	Construction machinery	TRA_OT_CNS, TRA_OT_LD2, TRA_OTH_LB	construction

[2] It seems that no specific provision for biomass was made and thus it is included here.
[3] In GAINS energy-related emissions in NMMI (largely cement production) are all absorbed into process-related emissions, see below.

The following approach is used for the mapping. Emissions from GAINS sectors (third column Table 43) are aggregated to an intermediary sector (first column) and then distributed among the MRIO sectors belonging to the clusters in the final column using their relative shares in the energy consumption. This is illustrated further for agricultural machinery and combustion devices in Figure 78.

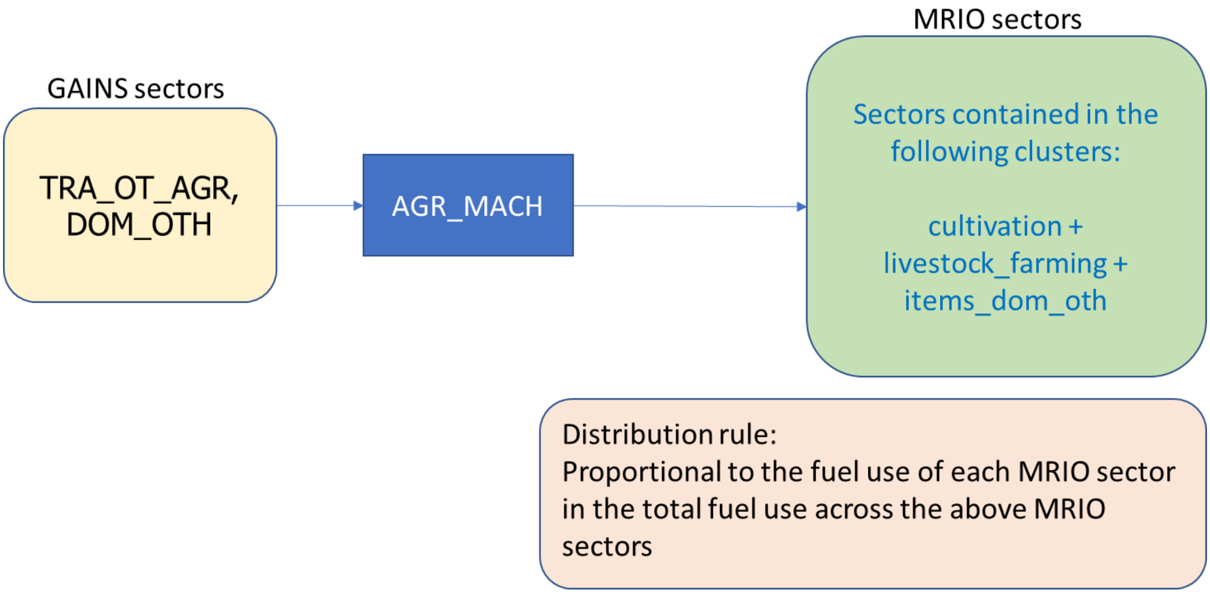

Figure 78 Approach for distributing emissions from agricultural machinery and devices (mobile and stationary) to MRIO sectors.

The energy related emissions in region *r* for MRIO sector *m* are thus:

$$\mathrm{Em}_r(m,e) = \sum_{label} \mathrm{Em}_r(\mathrm{label}, \mathrm{e}) \cdot \mathrm{sh}_r(m, \mathrm{e}, \mathrm{label}) \tag{5}$$

Where the sum is running over all labels given in Table 43 and the share

$$\mathrm{sh}_r(m, \mathrm{label}, \mathrm{e}) = \frac{\mathrm{FE}_r^*(m, label)}{\sum_{m'} \mathrm{FE}_r^*(m', label)} \tag{6}$$

is the share of MRIO sector *m* in the final energy demand (minus trucking) in the total final energy demand (minus trucking) in cluster *label* in region *r*.

Process-related emissions are calculated in GAINS separately from energy-related emissions, i.e. there are separate source categories for these in GAINS. Again, intermediary aggregation sectors, this time relevant for the processes, are defined as follows:

Table 44 Aggregated process-related sectors, their description and coverage in terms of GAINS sectors as well as MRIO clusters.

**Label**	**Description**	**GAINS sector coverage**	**MRIO clusters**
AGR_PROC	Process emissions related to cultivation	FCON_X, AGR_ARABLE, WASTE_AGR, APPLIC_X, GRAZE_X, STH_NPK, STH_AGR	cultivation
PROC_CATTLE	Emissions related to cattle farming	AGR_COWS, AGR_BEEF	Cattle farming (single sector)
PROC_PIG	Emissions related to pig farming	AGR_PIGS	Pigs farming (single sector)
PROC_POULT	Emissions related to poultry farming	AGR_POULT	Poultry farming (single sector)

PROC_OTANI	Emissions related to farming of other animals	AGR_OTANI	Meat animals nec (single sector)
PROC_BRICK	Emissions related to brick production	PR_BRICK	manuf_bricks
PROC_CEM	Emissions related to cement production	PR_CEM, PR_LIME	manuf_cem
PROC_NMMI	Emissions related to other non-metallic minerals	PR_NMMI, PR_GLASS	manuf_nmmi
PROC_IS	Emissions related to iron and steel production	PR_EARC, PR_BAOX, PR_HEARTH, PR_CAST, PR_SINT, PR_SINT_F, PR_PIGI, PR_PIGI_F, PR_CAST_F	manuf_is
PROC_ALU	Emissions related to aluminium production	PR_ALPRIM, PR_ALSEC	manuf_alu
PROC_FERT	Emissions related to fertilizer production	PR_FERT, FERTPRO	manuf_fert
PROC_CHEM	Emissions related to other chemical processes	PR_SUAC, PR_CBLACK	manuf_chem
PROC_PULP	Emissions related to paper and pulp production	PR_PULP	manuf_paper
PROC_CONVERSION	Emissions related to energy conversion	PR_REF, PR_COKE, STH_COAL, PR_PELL	ind_conversion
PROC_COAL_MINE	Emissions related to coal mining	MINE_HC, MINE_BC, PR_BRIQ	mining_coal_io
PROC_OTHER_MINE	Emissions related to other mining	STH_FEORE, MINE_OTH, STH_OTH_IN	mining_other_io
PROC_SM_IND	Emissions related to other small industries	PR_SMIND_F, OTHER_VOC, PR_OT_NFME, PR_OTHER, OTHER_PM	other_industries
PROC_CONSTRUCT	Emissions related to construction activities	CONSTRUCT	construction

The process related emissions in region *r* for MRIO sector *m* are thus:

$$\mathrm{Em}_r(m,p) = \sum_{label} \mathrm{Em}_r(\mathrm{label},\mathrm{p}) \cdot \mathrm{sh}_r(m,\mathrm{e},\mathrm{label}) \qquad (7)$$

Where the sum is running over all labels given in Table 44 and the share

$$\mathrm{sh}_r(m,\mathrm{label},\mathrm{e}) = \frac{\mathrm{FE}_r^*(m,label)}{\sum_{m'} \mathrm{FE}_r^*(m',label)} \qquad (8)$$

is the share of MRIO sector *m* in the final energy demand (minus trucking) in the total final energy demand (minus trucking) in cluster *label* in region *r*. The main difference to the energy related emissions is that the clusters are different, and thus the shares for each sector within a cluster may be different.

As noted above it is a simplification to distribute the process emissions proportional to the energy use in the MRIO sector within its corresponding cluster, and refinements could be made on the basis of information which of the MRIO sectors within a cluster are mostly related to the process emissions and in which proportion.

The total emissions associated with MRIO sector *m* is then simply the sum of the above energy-related, process-related, and trucking-related emissions of $PM_{2.5}$:

$$\mathrm{Em}_r(m) = \mathrm{Em}_r(m,e) + \mathrm{Em}_r(m,p) + \mathrm{Em}_r(m,t) \quad (9)$$

Data

1. EXIOBASE [243] is used for the global MRIO table and CO_2 emission inventory for the year 2019. In EXIOBASE, 44 territories and 5 rest of the world regions are covered in the resolution of 163 industrial sectors. The associated CO_2 emission inventory is mapped on a one-to-one sectorial resolution. Hence, consumption-based CO_2 can be easily obtained using equation (1).
2. To present the results in HDI country groups, the 44 territories are aggregated in accordance with HDI classification developed by UNDP. In the case of the 5 rest of the world regions, disaggregation of both consumption-based and production-based CO_2 inventories has been conducted in proportion to the national total 2018 production-based CO_2 emissions provided by the Global Carbon Project 2020 [279].
3. Similarly, upon the derivation of production-based $PM_{2.5}$ emission inventory using GAINS model, consumption-based $PM_{2.5}$ emission inventory can be easily obtained using equation (1). As for the 5 rest of the world regions, production-based emissions are disaggregated in proportion to 2015 $PM_{2.5}$ emission inventory of EDGAR database.[280] Consumption-based emission ratio of the 5 rest of the world regions is estimated based on CO_2 emission inventories.
4. Having consumption-based and production-based inventories for both CO_2 and $PM_{2.5}$ emissions ready, countries are grouped according to HDI levels for results analysis.

Caveats

The GAINS model separating $PM_{2.5}$ emissions into three groupings appears necessary for the following reasons. First, a simplification here is done just on the basis of the total fuel use, rather than on the basis of fuel specific data, though this could be further refined in the next version of this mapping tool. Second, process-related emissions are typically related to specific sectors and thus distributing the emissions among the same cluster as the energy-related emissions seems to introduce a smearing out that is not justified. Thus, process emissions from GAINS are distributed not across all MRIO sectors, but only across those that can be clearly identified with a particular process, and those for which a process emission cannot be further resolved. Finally, trucking-related emissions are distributed among all sectors on the basis of their diesel consumption. It is assumed that the relative share of diesel consumption for road transport in each MRIO sector is generally a good proxy for the relative share in the trucking-related emissions.

In the stage of emission inventory disaggregation, simplifications and assumptions may bring uncertainties into the results. When disaggregating the five rest of the world regions, unavailable data are either filled by emissions from previous years or estimated based on the structure of embodied emissions of other pollutants. The analysis can be updated when more accurate emission inventory becomes available in the future.

Additional Analysis

One of the main contributions of this work is a mapping between GAINS sectors and MRIO tables via the EXIOBASE energy extension. This is a powerful tool that allows us to map production-based accounts of primary $PM_{2.5}$ to MRIO tables and thus easily to consumption-based accounting schemata. So far, the analysis has focused on historical data, but the GAINS framework offers also prominently future perspectives in the form of scenarios. Thus, in conjunction with methods to project MRIO tables, the present methodology could be used to combine future emissions scenarios with future MRIO tables to assess future consumption patterns.

A number of simplifications have been made that could be refined in the next version of the mapping tool to increase the accuracy of the mapping. The mapping in this exercise is a viable tool to relate process-based calculations to consumption-based accounting frameworks. However, it is understood that the linking of frameworks that were built with different purposes (MRIO as an inventory relating economic inputs to economic outputs; GAINS as an integrated tool for air quality policy decision support based on forward looking scenarios) may result in conceptual anomalies. Furthermore, while numerical results are provided at high sectoral and regional resolution, it is important to keep in mind that at this level the results are more uncertain than at an

aggregated level. Further to the mapping process, assumptions and estimations made due to unavailable data points in the inventories will exacerbate uncertainties. In the future, the present methodology will be refined to reflect additional insights that will arise through the application of the method to different circumstances or updated inventories.

Section 5: Public and Political Engagement

5.1: Media Coverage of Health and Climate Change

Indicator 5.1.1: Global Coverage of Health and Climate Change

Methods

Intersecting trends in coverage of climate change and health were identified in 66 newspaper sources from January 2007 through December 2020. The 66 sources are located across 36 countries, in four languages, and spanning the six WHO regions. These sources were monitored through Nexis Uni, Proquest and Factiva databases accessed via the University of Colorado libraries.

The 2021 report of the Lancet Countdown adopts the search strategy developed for the 2020 Lancet Countdown report within these three databases.[1] The search strategy was revised for the 2020 report to increase the precision of the indicator; that is, to reduce the number of 'false positives', while retaining the maximum number of 'true positives'. This was done by retaining those terms that a) produced relevant data, and b) had a low degree of polysemy (i.e. words that have fewer meanings *or* words used in fewer disciplines/domains). Testing for interaction *between* terms also enabled fewer terms to be used (for example, it was found that the term 'morbidity' would usually pull in the term 'mortality', when related to humans).

The terms were translated once the strategy had been finalised with certain terms presenting difficulties in translation. The English terms 'hay-fever' and 'West Nile', for example, correlated with more than one term in Spanish and Portuguese and the decision was made to include all relevant terms in the respective search strategies.

For the final strategy, search functions were compared across databases to ensure consistency, as different databases utilise different search filter operators. The searches were conducted with the following key words in English, Spanish, Portuguese and German respectively:

English:

(climate change OR global warming) AND (health OR illness OR epidemiolog* OR malnutrition OR morbidity OR fatalit* OR diarrh* OR malaria OR chikungunya OR west nile OR dengue OR hay-fever OR zika)

German:

(Klimawandel OR Globale Erwärmung) AND (Gesundheit OR Krankheit OR Epidemiolog* OR Mangelernährung OR Morbidität OR Sterblich* OR Durchfall* OR Malaria OR Chikungunya OR West-Nil-Virus OR Dengue-Fieber OR Heuschnupfen OR Zika)

Portuguese:

(mudanças climáticas OR aquecimento global) AND (saúde OR doença OR epidemiologi* OR desnutrição OR morbilidade OR fatalidade* OR diarr* OR malária OR chikungunya OR nilo do oeste OR vírus do nilo OR dengue OR febre dos fenos OR rinite alérgica OR zika)

Spanish:

(cambio climático OR calentamiento global) AND (salud OR enfermedad* OR epidemiología OR epidemiólog* OR desnutrición OR malnutrición OR morbosidad OR muert* OR diarrea* OR malaria OR paludismo OR chikungunya OR nilo del oeste OR nilo occidental OR virus del nilo OR dengue OR fiebre del heno OR rinitis alérgica OR zika)

The signal of the search strategies above was found to be strong enough (over 80% relevance in a systematically randomised sample of 500) to allow a more parsimonious approach to this indicator, requiring no screening of articles during the extraction of the data.

Two separate searches were also undertaken with the inclusion of gender ("AND gender") and COVID-19 ("AND (covid* OR corona* OR sars-cov-2")) terms to the search strategies in English above. Only the searches in English

sources (in Nexis Uni and Factiva) were undertaken, though it is envisaged that the 2022 report will extend this to all sources. The health, climate change and gender search was undertaken for the same time period as the core health and climate change search (2007-2020) and the health, climate change and COVID-19 search was undertaken for 2020.

Results were obtained from the databases by entering the relevant search strategy along with the relevant date. Counting occurred month by month and the number of returns for each source was recorded on a Microsoft Excel spreadsheet. Primary counting took place for each source along with a secondary independent count of a systematically randomised 20% sample by another researcher. Tertiary counts were undertaken where any mismatch occurred between primary and secondary counts. All counts were agreed by the whole research team.

Using the Excel spreadsheet constructed through the phases of counting, the data was organised in numerous ways for a better understanding of the patterns in coverage. These included by WHO region, by the most recent (2020) Human Development Index categories, and by individual source. The average scores for each month (and aggregated into annual averages) were used as an adjustment for the number of sources selected per region or index category.

Data

1. Three databases were used for the core health and climate change search strategy: Nexis Uni; Proquest; and Factiva databases accessed via the University of Colorado libraries. The 66 newspaper sources are located across 36 countries, in four languages, and spanning the six World Health Organization (WHO) regions.
2. Two databases were used for the health, climate change and gender search strategy: Nexis Uni; and Factiva databases accessed via the University of Colorado libraries. The 50 newspaper sources are located across 23 countries and span the six World Health Organization (WHO) regions.
3. Two databases were used for the health, climate change and COVID-19 search strategy: Nexis Uni; and Factiva databases accessed via the University of Colorado libraries. The 50 newspaper sources are located across 23 countries and span the six World Health Organization (WHO) regions.

Caveats

In developing the search strategy for the 2020 Lancet Countdown report, it was found that a significant portion of articles may mention both climate change and health but do not engage with them as integrated issues. For example, as identified in indicator 5.1.3, an article could cover an election candidate's political priorities, including a discussion of the health sector and a candidate's response to climate change. However, including this coverage remains important as it brings both sets of issues – health and climate change – onto the public agenda and into public awareness. For the 2022 report, indicator leads will explore ways to isolate reports with a meaningful connection between health and climate change.

Future form of the indicator

The 2022 Lancet Countdown report will look to extend the gender and COVID-19 keyword searches to all sources, regardless of language. Before the 2022 report, authors will also explore ways to isolate reports with a meaningful connection between health and climate change.

Additional analysis

Figure 80 shows the total newspaper co-coverage of health and climate change and health, climate change and COVID-19 in 2020, for which further description is provided in the main text of the 2020 Report of the Lancet Countdown. Figure 75 shows the total co-coverage of health and climate change across all sources between 2007 and 2020. For the third year in a row, 2019-2020 shows an increase in co-coverage of health and climate change over the previous year. This increase is far smaller than the previous year's, however, with a +6% percentage change compared with +96% between 2018 and 2019.

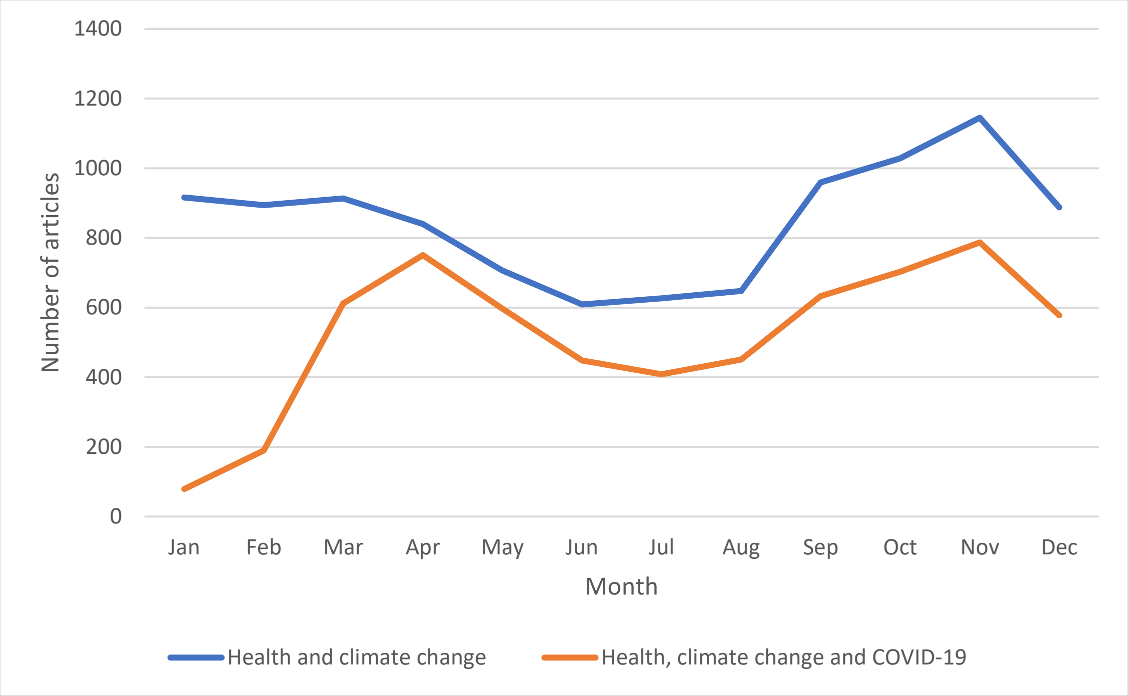

Figure 79. Newspaper coverage of (i) health and climate change and (ii) health, climate change and COVID-19 in 2020 in 24 countries.

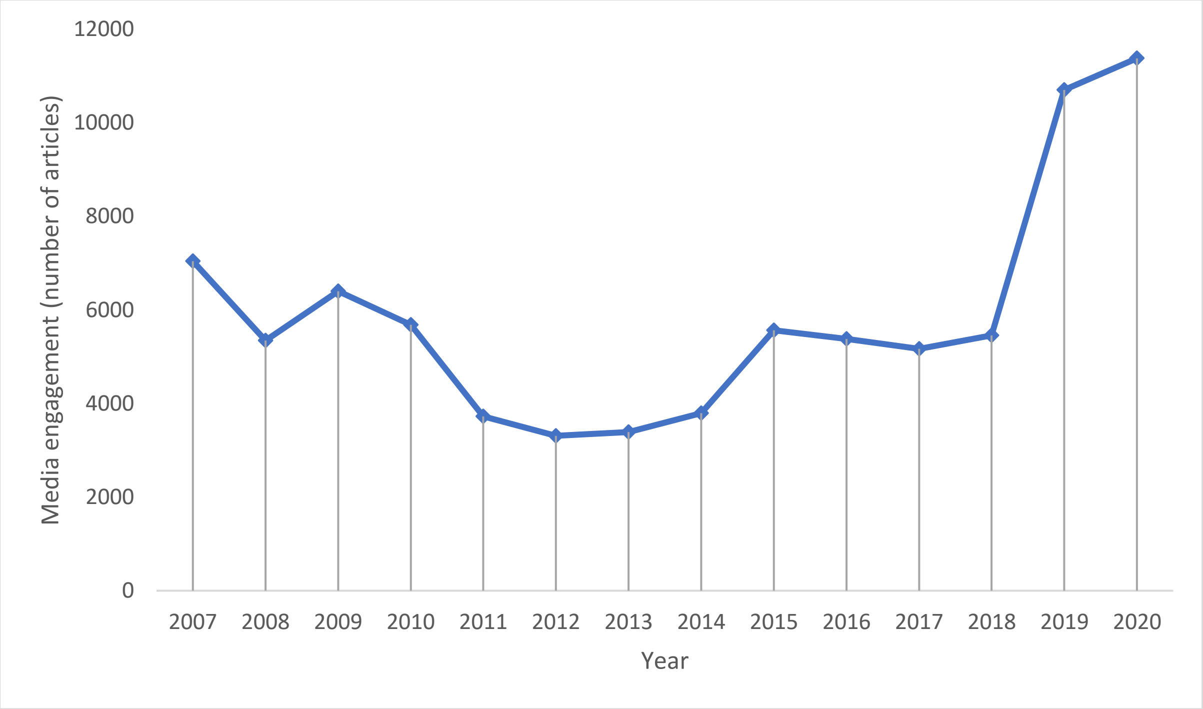

Figure 80. Total media engagement measured by number of articles with health and climate change co-coverage, across all sources (2007-2020).

Total media engagement across all sources in 2020

Figure 81 shows the total number of articles containing both a health and climate change key word in 2020. From March there is a notable dip in co-coverage, reaching the year's lowest point in June (687), and only recovering in September (1099). As discussed below, this is likely to be related to enhanced global coverage of COVID-19 after March. The highest point of co-coverage is November with 1278 articles containing both a health and climate change term.

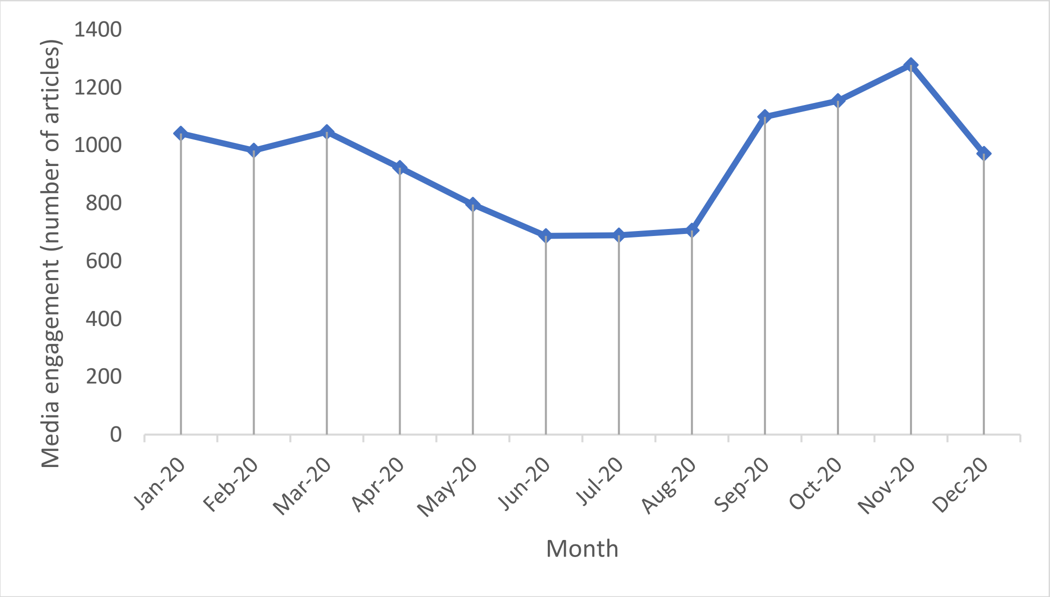

Figure 81. Total media engagement measured by number of articles with health and climate change co-coverage, across all sources in 2020.

Geographical distribution of newspaper coverage

Figure 82 describes the average number per year of articles with co-coverage of health and climate change by WHO region. It demonstrates that the Americas have the highest average co-coverage in 2020 with 205 articles containing a health and climate change keyword per source. Four regions increased in their average co-coverage of health and climate change from 2019 to 2020: the African region (+46%), the Americas (+18%), Western Pacific (+8%), and the Eastern Mediterranean (+8%). The remaining two regions had a lower average of co-coverage from 2019 to 2020: the European (-5%) and South East Asian (-7%) regions.

Between 2007 and 2020, the Eastern Mediterranean region shows by far the highest increase in engagement (+1185%, from an average of 4 per year in 2007 to 56 in 2020). This is followed by the South East Asian (+158%) and African (+145%) regions and the Americas (+122%). The European region has a 59% increase in average co-coverage from 2007 to 2020, whereas the Western Pacific actually shows, because of its early spikes in co-coverage, a very small percentage decrease of 0.3%.

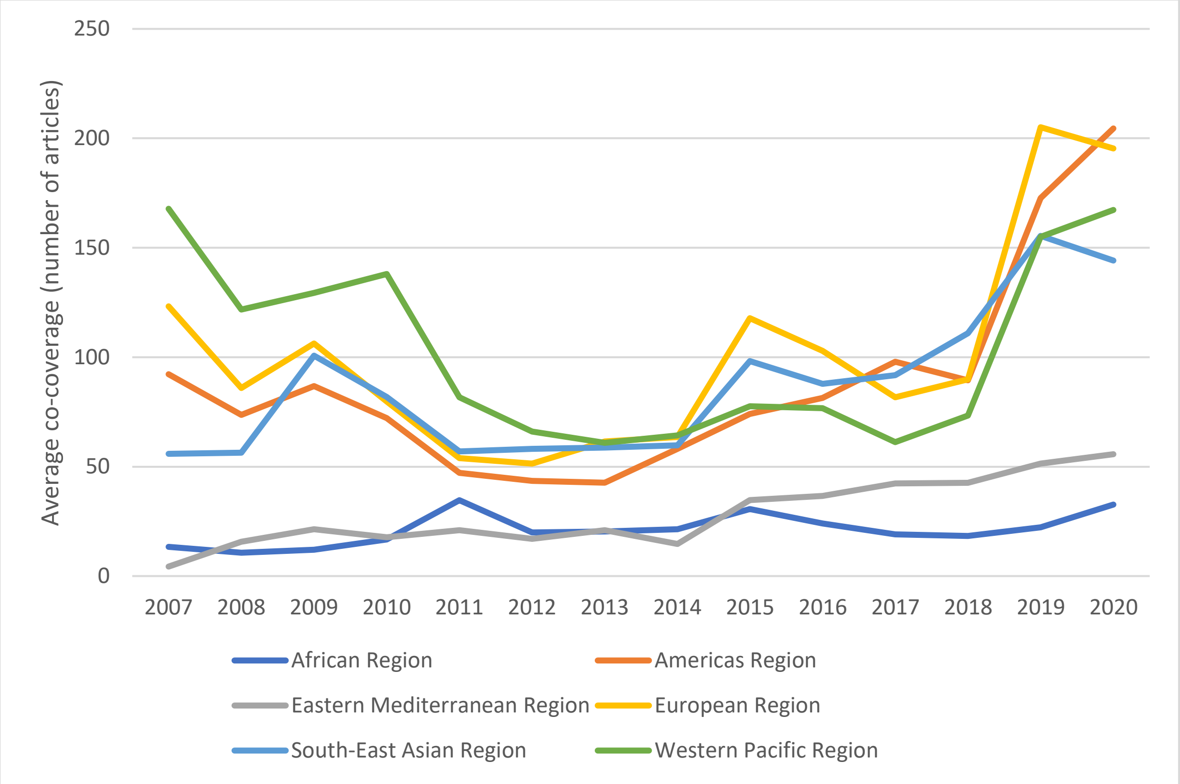

Figure 82. Average annual media engagement by WHO region from 2007 to 2020.

Distribution of newspaper coverage by Human Development Index

Figure 83 presents the average number of articles per year containing health and climate change key words by HDI (2020) classification group (very high, high and medium; no included newspaper sources were from the low HDI group). These all follow similar trends across the 14-year period with peaks around 2009, 2015 and 2019 and a general dip from 2011 to 2014. The Very High HDI group makes the biggest contribution to co-coverage overall (2007-2020) with 47%, followed by the medium (31%) and high (22%) HDI groups.

From 2019 to 2020, increases in average engagement are clear in both the very high (+5%) and the high (+48%) HDI groups. Although there is a decrease in engagement in the medium HDI group (-14%) between 2019 and 2020, across the 14-year period this group sees the largest overall increase (+470%, from an average of 23 articles per year to 132), far higher than the very high (+57%) and high (+22%) HDI groups.

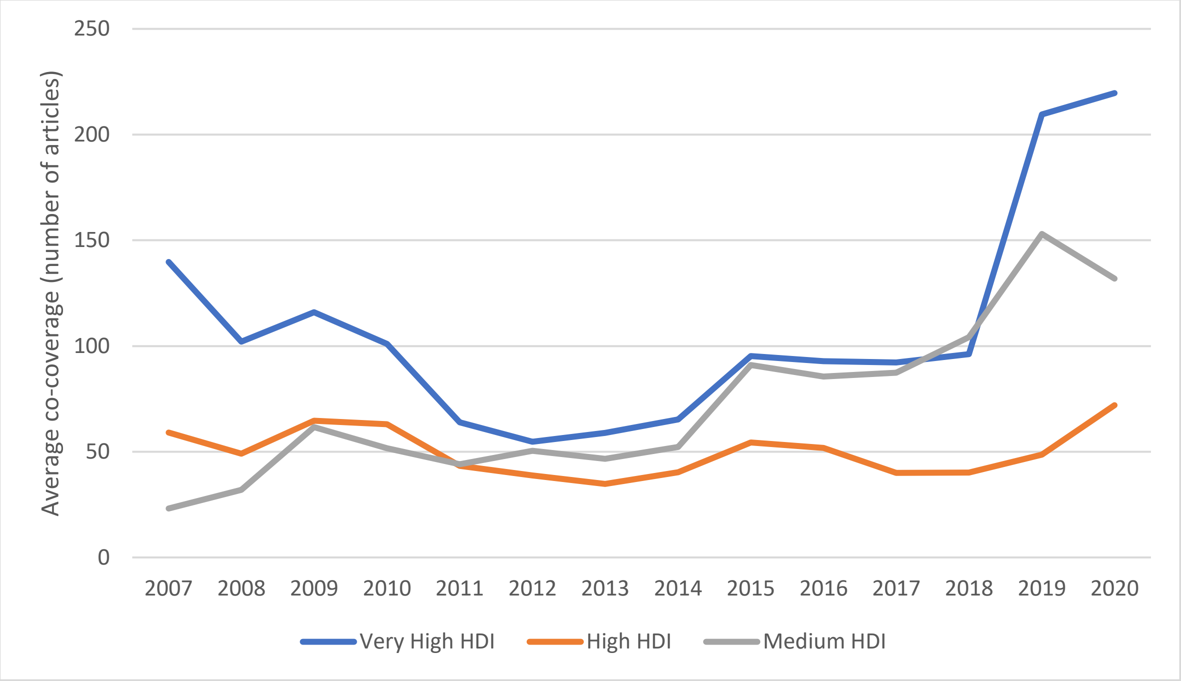

Figure 83. Average annual media engagement by HDI classification group from 2007 to 2020.

Figure 84 shows the average media engagement across 2020. Where very high HDI countries appear to dip in their co-coverage after March, contributing heavily to overall engagement which does the same, the dip in the high and medium HDI countries is less pronounced, dropping from March to April but picking up once again by May. The end of the year sees an overall decline in engagement, but an increase in medium HDI contribution.

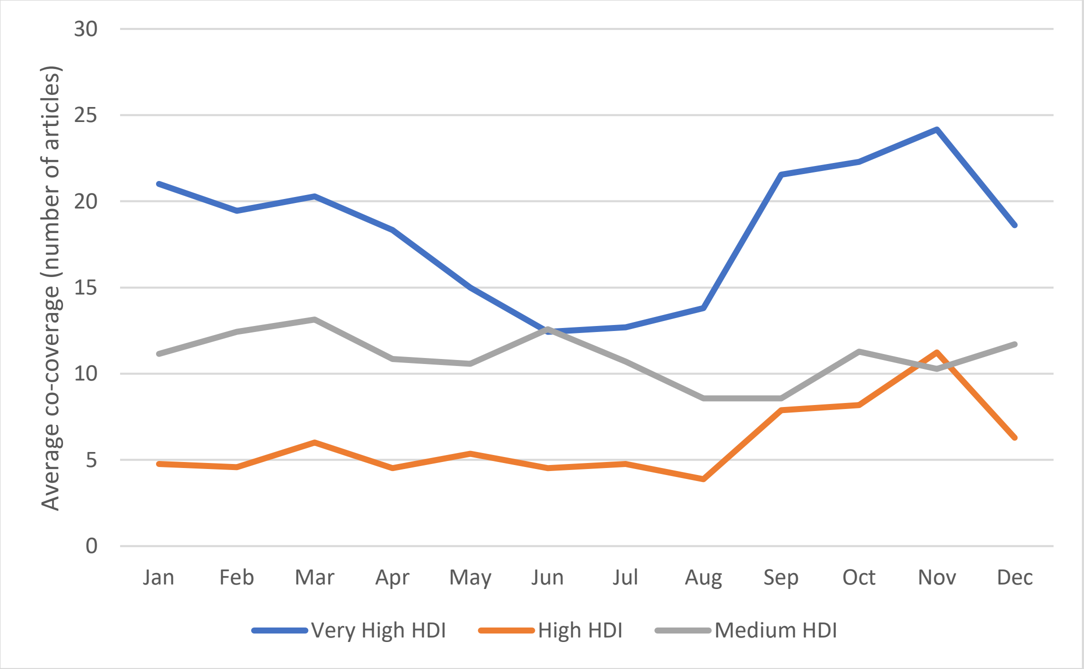

Figure 84. Average media engagement by HDI classification group in 2020.

Health, climate change and COVID-19

Figure 85 shows the proportion of the articles containing health and climate change key words as well as at least one COVID-19 keyword in 2020 (in the 50 English-language news sources). An inverse pattern, particularly around March and April, can be observed between the trend here and overall engagement with health and climate change: where a dip can be observed in overall engagement (Figure 84above), a peak can be seen in proportion of articles containing a COVID-19 key word (Figure 85). This might suggest an effect of COVID-19 on the coverage of other issues, including health more generally and climate change. From February to March the proportion also containing a COVID-19 key word increases from 21% to 67%. This reaches its highest point, of 89%, in April. This is followed by a steady drop, at its lowest in July (65%), before a second peak in August (70%).

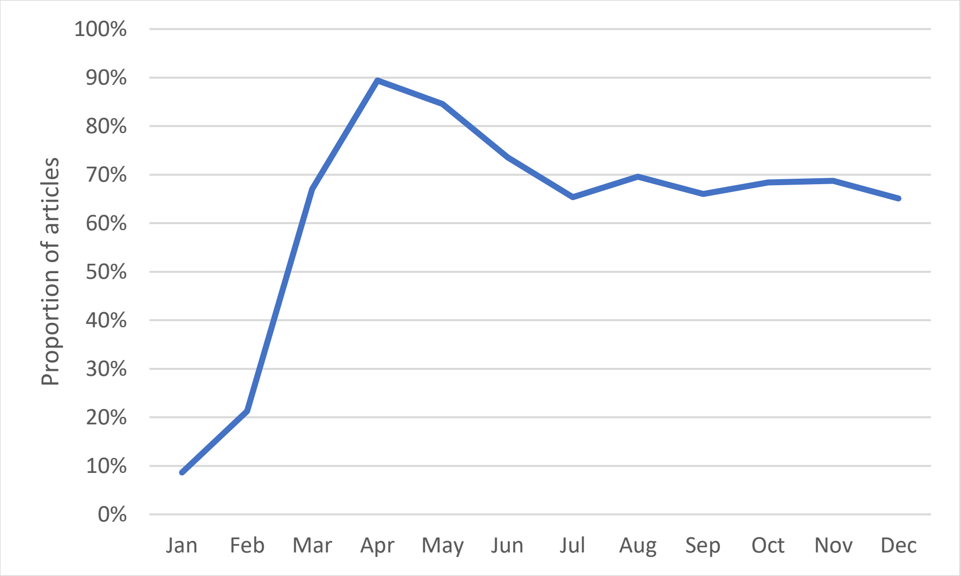

Figure 85. Proportion of 2020 articles containing both health and climate change key words and a COVID-19 key word (out of 49 English-language news sources).

Broken down by WHO region, as demonstrated in Figure 86, the Americas (here made up of only North American countries, Canada and the USA) make the largest average contribution to co-coverage of health, climate change and COVID-19, with an average of 35 articles a month and 36 in both months with the highest overall number, April and November, respectively. In the South East Asian region, co-coverage starts off, in March through until July, as strong as the European and Western Pacific regions but drops substantially in the second half of the year.

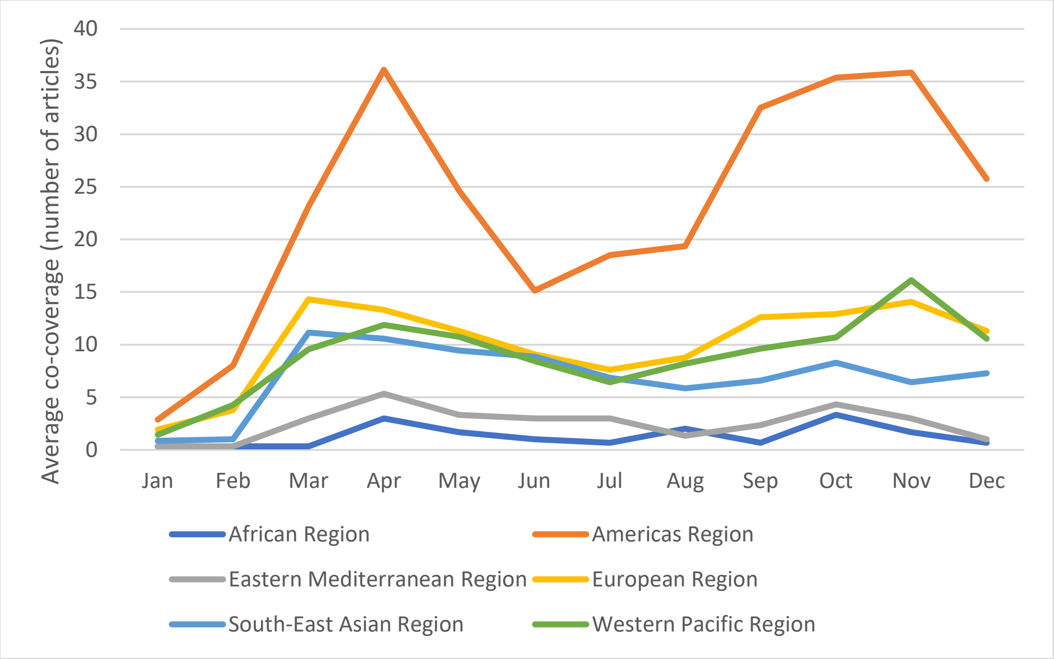

Figure 86. Average co-coverage of health, climate change and COVID-19 key words in 2020, by WHO region.

Broken down, instead, by HDI classification categories – as in Figure 87– countries in the very high HDI group contribute most to co-coverage of health, climate change and COVID-19. This is perhaps not surprising given that this group contains both North American countries, which Figure 86 already demonstrates as dominant contributors. It appears that those countries in the medium HDI group have a higher peak in March than the high HDI group, before slowly dropping prior to a second smaller peak in October, whereas countries in the high HDI group demonstrates a more gradual rise throughout the year before peaking in November and dropping in December.

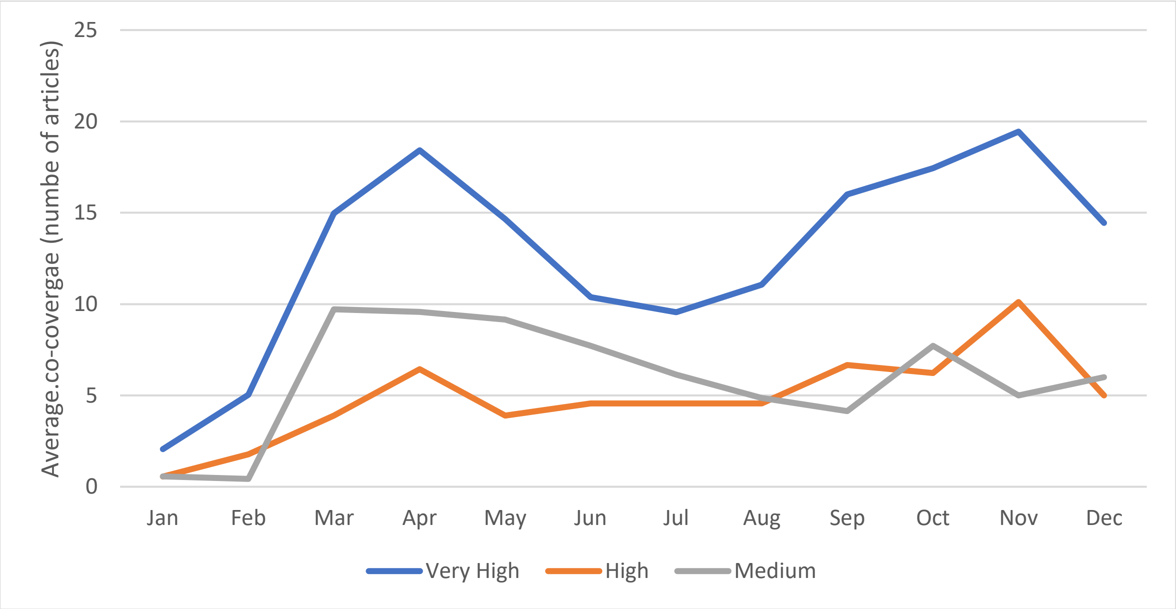

Figure 87. Average co-coverage of health, climate change and COVID-19 key words in 2020, by HDI classification group.

Health, climate change and gender

Figure 88 and Figure 89 show co-coverage of health, climate change and gender key words across the 14-year period. Figure 88 demonstrates the vast distance between the number of articles with health and climate change key words and the number also including 'gender'. It also shows, however, that this number is rising. In fact, where health and climate change display an increase of 68% from 2007 to 2020, health, climate change and gender demonstrate a greater percentage increase of 491%. Though based on small numbers, the linear trendline in Figure 89 highlights this general increase. These results need to be interpreted with some caution, however, particularly given lower numbers, as co-coverage does not necessarily signal a meaningful connection.

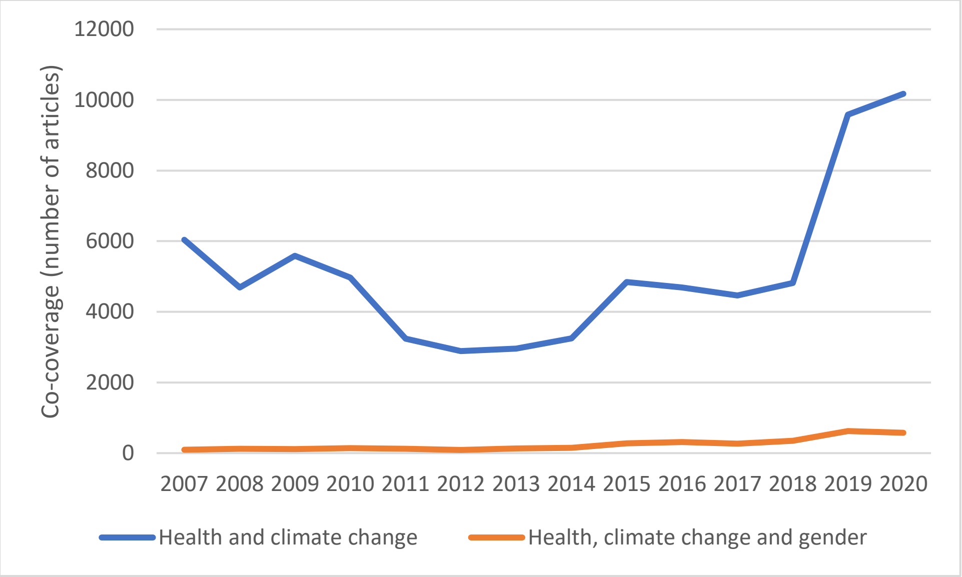

Figure 88. Co-coverage of health and climate change and health, climate change and gender from 2007 to 2020.

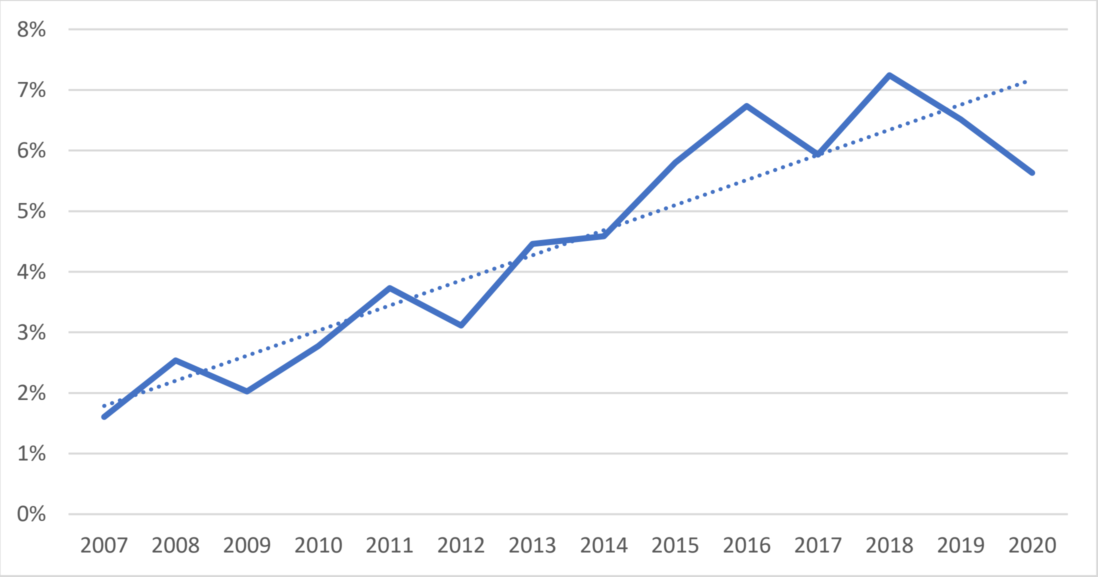

Figure 89. Proportion of health and climate change articles also containing the word 'gender', from 2007 to 2020.

Figure 90 and Figure 91 show average co-coverage of health, climate change and gender across WHO regions and HDI classification groups, respectively. Both demonstrate similar numbers between groups until 2014 before a) a general rise in co-coverage aligned with the overall numbers (Figure 88), and b) a spike in one particular group: the Americas WHO region which, given its constitution of North America for this search strategy, contributes heavily to the very high HDI group.

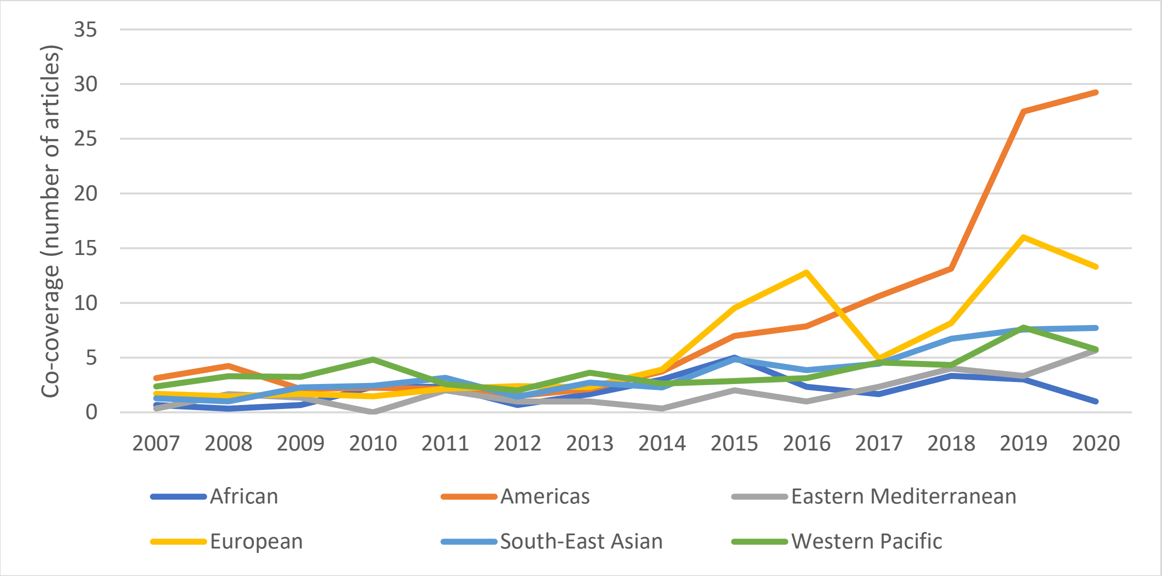

Figure 90. Average co-coverage of health, climate change and gender by WHO region, 2007-2020.

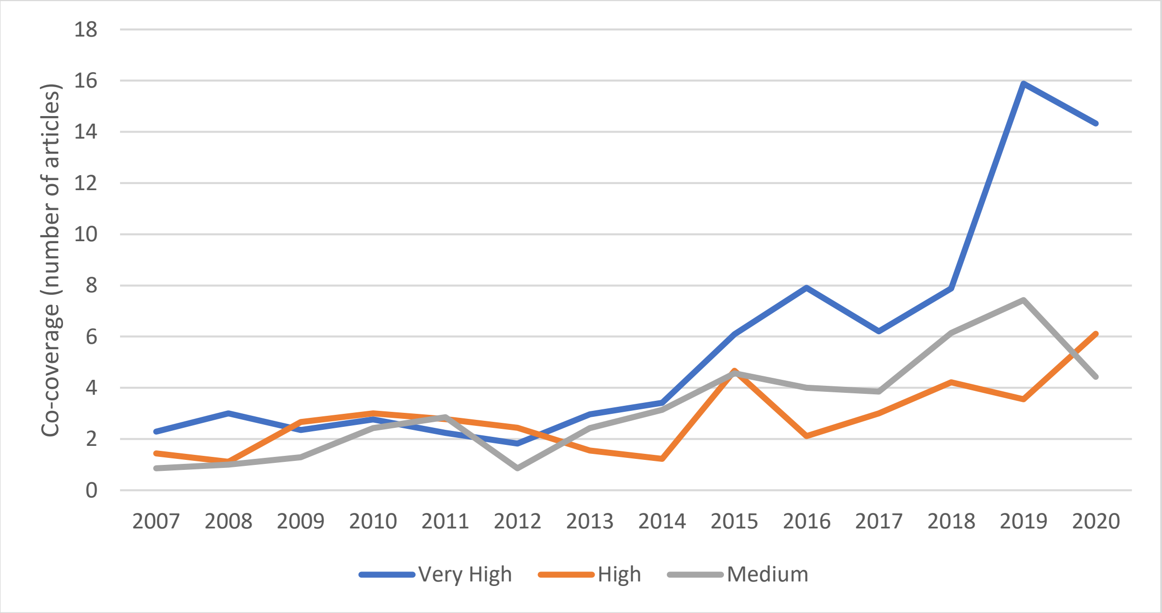

Figure 91. Average co-coverage of health, climate change and gender by HDI classification group, 2007-2020.

Indicator 5.1.2: Coverage of Health and Climate Change in China's People's Daily

Methods

The 2021 Lancet Countdown Report used the methodology used in 2020. This involved, firstly, trawling all articles and then searching the keywords in the text with the filtration process by score and keywords ratio as filtration criteria. The detailed explanation of "score" and "keywords ratio" can be found in step 4 of the method. Also explored were the number of articles related to gender in the health and climate change coverage articles from 2008 to 2020 as described in step 6-7, as well as the number of articles related to COVID-19 in the health and climate change coverage articles from 2019 to 2020 as described in step 8.

The detailed steps of the method used in 2021 are shown as below:

Step 1 Trawling all articles in 2020

All articles that were published in "People's Daily" in 2020 were trawled (http://paper.people.com.cn/rmrb/html/2021-03/05/nbs.D110000renmrb_01.htm)

Step 2 Searching for "Climate Change" topic articles

Focus was concentrated on articles containing the keywords in the topic of "Climate Change". The keywords presented in the first column of Table 45 were searched. The keywords in the first column of Table 45 are identical to the keywords from the 2020 Lancet Countdown report to ensure comparability over time. The result is shown in Figure 92.

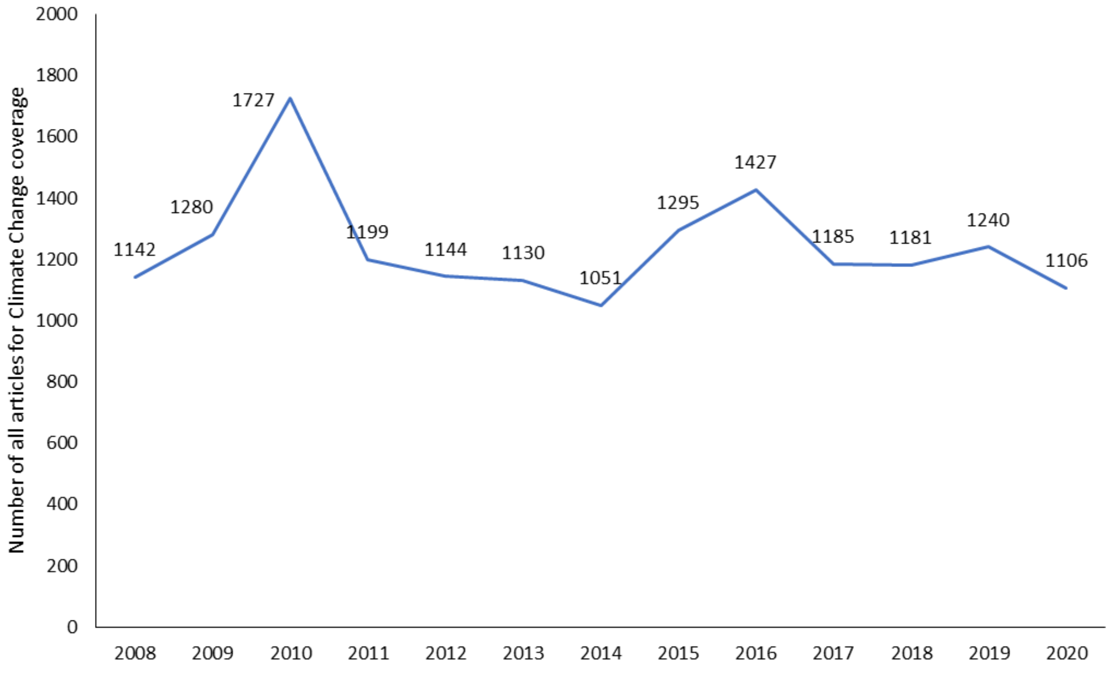

Figure 92. Number of articles identified in People's Daily by searching the keywords from topic Climate Change (first column of Table 45).

Step 3 Identifying articles that have both climate change and health keywords (first-round search)

First-round filtration was then carried out to identify articles that have both climate change and health keywords. These were then used as the basis for the second-round search in step 4. Health-related keywords can be found in column 3 of Table 45.

Step 4 Machine filtration of the results from step 3 by score and ratio (second-round search)

The articles obtained from step 3 were first scored based on the times of appearance of the keywords shown in the articles. For example, if the keywords of climate change and health have appeared 12 times in one article, then the score for this article would be 12. If the keyword found was a "mis-hit word" (defined as a phrase containing a keyword but with a different meaning), the appearance was not counted as one score (lists of "mis-hit words" can be found on pages 143-144 of the 2020 Lancet Countdown report).[1] At the same time, the ratio of times of appearance of the keywords to the total number of characters in the article (short for "the ratio" thereafter) was also calculated. When the score and the ratio of one article were both higher than the manually-set thresholds, the article was considered relevant. Via this step, the numbers of relevant articles are illustrated by the grey line in Figure 93.

The threshold of score for each article was set at 10, meaning the times of appearance of the keywords from both climate change and health in one article should be no less than 10. The threshold of ratio for each article was set to be no less than 1%, meaning in every 100 characters in the article, there should be no less than 1 keyword.

If the two thresholds were set too low, it would increase the workload of manual screening and increase the "false positive rate" of machine filtration. And if the two thresholds were set too high, it would possibly exclude the "true positive" articles. So after several trial tests, it was determined that the thresholds for score and the ratio were best set as no less than 10 and 1% respectively.

Step 5: Manual screening of the results after machine filtration

The fifth step was manually screening the filtered articles. Articles where it was confirmed that the topic is health and climate change were retained. The orange line in Figure 93 shows the number of articles that passed the manual screening.

In Figure 93, the number of health and climate change coverage articles in 2020 was different before and after manual screening. Before manually screening, there were 93 articles, which was the highest in the previous 12 years. However, only 14 articles were truly related to the topic identified by manual screening, which was lower than the average. The results indicated that there were a lot of false positive articles identified by the machine filtration process in 2020. After manually checking the false positive articles, it was found that many items of epidemic news were inserted into the media articles in 2020. The wording used in the epidemic news had a large overlap with our health topic keywords, which were used to identify health topic article in step 4. However, the epidemic news had no links with climate change. Therefore, these articles were regarded as "negative" after manual screening.

Titles of the 14 positive articles were presented in the additional analysis as Table 49.

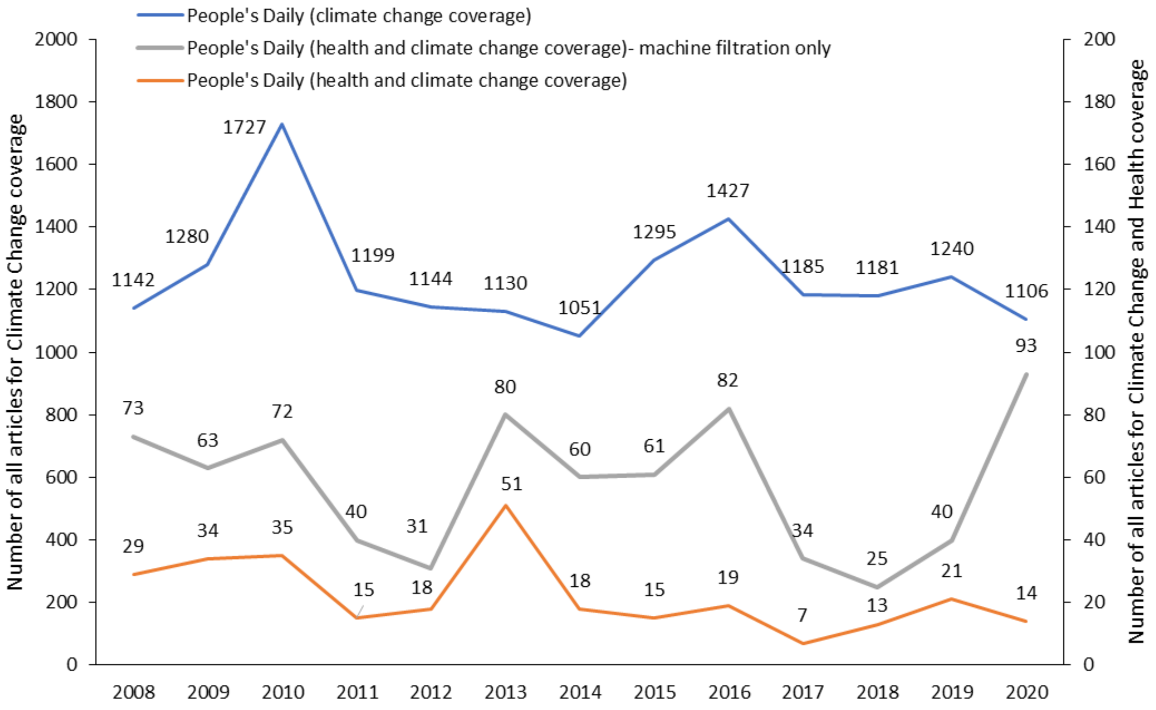

Figure 93. Numbers of all articles for climate change only (blue line), for both health and climate change after machine filtration only (grey line), and for both health and climate change after machine filtration and manual screening (orange line).

Step 6 Searching for gender topic articles

In this step, a further filtration was undertaken, which aimed to identify articles that have at least one gender keyword in the set of screened articles that talked about health and climate change. The gender keywords are presented in Table 47. The result showed that there were 11 articles that contained gender keywords. Titles of the 11 articles are presented in the additional analysis as Table 50.

Step 7 Manual screening of the results of gender topic after machine filtration

Next, the filtered articles were manually screened. If the manual screening confirmed that the topic is a gender topic, it was retained. However, 11 articles were found to be 'false positives'.

The false positive articles were mainly of two types. Firstly, gender inequality is directly linked to health, and is only distantly linked with climate change. For this type, the keywords setup could be adjusted to lower the manual screening workload. The second reason was the keyword setting; for example, the word "daughter" also takes the form of "female kid" in the Chinese language. Words like "daughter" were labelled ambiguous keywords. The same problem was met in the filtration process for the health and climate change coverage articles. The resolution was to make the ambiguous keywords into a list of "removal words" once enough sample keywords had been collected, the removal words not counted in any score in the machine filtration process (Step 1).

Step 8 Manual screening of the articles of COVID-19 topic

The number of articles related with COVID-19 was explored in the health and climate change coverage in this step. Since there were 17 health and climate change articles from the end of 2019 to 2020, these were manually screened to search for the COVID-19 topic. Although some articles mentioned the epidemic (COVID-19 keywords in Table 48), the linkage of the articles with health and climate change was very weak.

Table 45. Chinese keywords for the search in People's Daily.

气候变化关键词	气候变化二级关键词	健康关键词	剔除词
气候变化	霾	疟疾	口蹄疫
全球变暖	空气污染	腹泻	黑烂病
温室	大气污染	感染	珊瑚死亡
极端天气		肺炎	沙虫死亡
全球环境变化		流行病	高温加热
低碳		公共卫生	低碳水
可再生能源		卫生	健康发展
碳排放		发病	生态健康
二氧化碳排放		营养	河流健康
气候污染		精神障碍	生态环境健康
气候		发育	
全球升温		传染	
再生能源		疾患	
CO2排放		症	
污染		瘟疫	
极端气候		流感	
高温		流行感冒	
变暖		治疗	
排放		保健	
环境变化		健康	
升温		死亡	
全球温升		精神疾病	
热浪		精神病	
暴雨		登革热	
气温		饥饿	
洪水		粮食	
洪灾		有害	
气候反常		皮肤病	
野火		风湿	
山火		呼吸系统疾病	
雪灾		人类健康	
低温		人体健康	
年代际		身体健康	
冰雪		心脏病	
可持续发展		糖尿病	
海洋酸化		疾病	
静稳		热死	
温室气体		口罩	
		防护	

Table 46. English translation of the Chinese keywords.

Keywords of "Climate Change"	Sub-level keywords of "Climate Change"	Keywords of "Health"	Removal words
**Climate change**	Haze	Malaria	Aftosa
**Global worming**	Air pollution	Diarrhea	Black shank
**Greenhouse**	Atmospheric Pollution	Infected	Coral death
**Extreme weather**		Pneumonia	Sandworm death
**Global environment change**		Epidemic	Heating to higher temperature
**Low carbon**		Public health	Low carbohydrate
**Carbon dioxide emissions**		Hygiene	Healthy development
**Renewable energy**		Disease outbreak	Ecological health
**Carbon Production**		Nutrition	River health
**Air pollution**		Mental disorders	Eco-environmental health
**Climate**		Growth	
**Global worming**		Infection	
**Renewable energy**		Affection	
**CO2 emissions**		Symptom	
**~~Pollution~~**		Epidemic	
**Extreme weather**		Flu	
**High temperature**		Influenza	
**Warming**		Treatment	
**Emission**		Health care	
**Environmental change**		Health	
**Warming**		Death	
**Global warming**		Mental disease	
**Heat wave**		Mental illness	
**Rainstorm**		Dengue	
**Temperature**		Hunger	
**Flood**		Food	
**Flood**		Harmful	
**Abnormal weather**		Skin disease	
**Wildfire**		Rheumatism	
**Mountain fire**		Respiratory diseases	
**Snowstorm**		Human health	
**Low temperature**		Body health	
**Interdecadal**		Heart disease	
**Ice and snow**		Diabetes	
**Sustainable development**		Illnesses	
**Ocean acidification**		Heat death	
**Stagnant**		Mask	
**Greenhouse gas**		Protection	
		Survive	

Table 47. Gender keywords for the search in People's Daily.

性别关键词	Gender Keywords
性别	Gender
女	Female
男	Male

Table 48. COVID-19 keywords for the search in People's Daily.

疫情关键词	COVID-19 Keywords
COVID*（COVID-19，COVID 19，COVID19）	COVID*（COVID-19，COVID 19，COVID19）
新冠*（新冠病毒，新冠病毒肺炎，新冠疫情，新冠病毒肺炎疫情，新型冠状肺炎，新型冠状病毒，冠状病毒）	Corona (Corona Epidemic)
新型冠状病毒	Coronavirus
SARS	SARS
2019-nCoV	2019-nCoV

Data

All the articles from 2008 to the present published on People's Daily (from the official website of People's Daily).

Additional analysis

Titles of the articles in 2020:

Table 49. Title of the health and climate change articles in People's Daily.

文章名字	Titles of the article
印尼洪灾已造成16人死亡	The flood in Indonesian has caused 16 deaths
南部非洲遭遇持续干旱威胁	Southern Africa is threatened by the persistent drought
非洲粮食安全面临严峻挑战（国际视点）	Food security in Africa faces serious challenges (international perspective)
根据传染病防治规律采取有效举措 科学打赢疫情防控阻击战	Effectively take infectious disease prevention and control, win the battle against the epidemic
反应迟缓缺统筹 应对不力遭诟病 澳大利亚林火肆虐数月损失巨	The forest fire in Austria has continued for months and suffer great losses. Government is criticized by slow respond, lack of coordination and ineffective in responding.

《2019年中国气候公报》发布 去年我国气温偏高降水偏多	*China Climate Bulletin 2019* release<br>---- A higher temperature and a heavier precipitation was observed last year in China
印尼雅加达极端天气现象频发	Extreme weather happens in Jakarta frequently
微阅读	Readings (A section of People's Daily)
合理利用气候与水资源 发挥气象趋利避害作用 ——写在二〇二〇	Use the climate and water sources wisely ---written in 2020
联合国政府间气候变化专门委员会发布预警	The United Nations Intergovernmental Panel on Climate Change issued an early warning
欧盟谋求提高生态系统可持续性	The EU seeks for improving the Ecosystem sustainability
微阅读	Readings
阿富汗东部洪水致死人数升至70人	70 deaths were caused by the flood in Eastern Afghan
加强国际合作 分享减贫经验 推进可持续发展 ——"联合	Strength international cooperation, share reduction experience and promote sustainable development----combine
美国西部山火持续蔓延	The wildfire in western America continues to spread
汇聚起可持续发展的强大合力	Gather a strong force for sustainable development
加大投资力度 强化国际合作 非洲多国全力保障农业生产	Increasing investment and strengthening the international cooperation, many African countries devoted to ensure the agricultural production

Table 50. Titles of the false positive articles of gender coverage in People's Daily.

文章名字	Title of the article
砸不垮的脊梁	Unbreakable spine
粮食安全关乎非洲发展前景	Food security is vital to Africa's development
"我们一定能战胜洪灾"	We can overcome the flood
当地回应称，不存在任何隐瞒	The local responded that there is no concealment
澳大利亚热议环境治理困境	Australia hotly discusses environmental governance
治雾霾，谁和谁在博弈？	Who is playing against the smog?
非洲多国粮食安全问题依然严峻	There are still many serious problems about food security in Africa countries

源头精细管控按尾气排放限行	Fine control at the source according to exhaust emission limits
今夏要防“南涝北旱”	Prevent "Southern floods and North droughts" this summer
百年最强厄尔尼诺形成	The strongest El Nino in a century
今夏为啥这么热	Why is this summer so hot?

Indicator 5.1.3: Content of Coverage in US and Indian Newspapers

Methods

This indicator complements the tracking of media engagement by focusing on the *content* of media coverage of health and climate change, enabling some understanding of qualitative direction as well as the levels of coverage. The 2021 Lancet Countdown iteration adopts a different methodology from previous reports, utilising automated machine learning functions in R, specifically, probabilistic topic modelling. This is used in conjunction with an iterative qualitative process for topic or theme validation.

Media sources and timeframe

The focus was on the elite media in two countries representing very different contexts. Two English-language newspapers from India and two from the US were examined, across the whole of 2020. The media sources considered are the *Hindustan Times* (HT), *Times of India* (TOI), *Washington Post* (WP), and *New York Times* (NYT).

Search terms

Media articles were obtained in conjunction with researchers developing indicators for section 5.1.1 (trends in media coverage). Search terms developed by this team of researchers, that were designed to return articles at the intersection of health and climate change, were used, incorporated into the following search strategy:

(climate change OR global warming) AND (health OR illness OR epidemiolog* OR malnutrition OR morbidity OR fatalit* OR diarrh* OR malaria OR chikungunya OR west nile OR dengue OR hay-fever OR zika)

Both Indian media sources (HT and TOI) and one American source (WP) were searched via Factiva. The remaining American source was searched via the Nexis Uni database.

Pre-processing, pre-screening and key-words-in-context

The initial search string returned 2088 articles across the four media sources. These were uploaded into R and pre-processed for analysis with the removal of punctuation, symbols, numbers, stopwords, and URLs and by lowercasing all tokens. This was achieved using the “quanteda” package.[281]

The data contained multiple duplicates, including the same news stories repeated with a slightly different title. Duplicates were therefore excluded using the *distinct* function from the *dplyr* package,[282] which enabled exclusion based on multiple variables within a data-frame.

Following pre-processing, all data were be sorted into a data-frame consisting of one article per observation/row and included a text identification code, the main body of the text, the publication date, the source and its country of origin, and other meta data, such as the title of the article.

quanteda’s Key-Word-in-Context (*KWIC*) function was then used to capture uses of health-related terms in combination with climate change-terms (as per the search strategy). KWICs are concordances that enable terms to be found while capturing a specified number of words around them, allowing some understanding of context. In line with other WG5 indicators using text-mining approaches (e.g. 5.4.1), this indicator will use a 25 word ‘window’ around the keyword, which corresponds to approximately half a paragraph of text. This was selected to capture enough text for an understanding of context, but not so much as to draw in other potentially unrelated stories, as multi-story articles often appear in the news media.

The resulting KWICs went through another deduplication procedure, as they were likely to contain more than one health or climate change term and therefore return similar passages. As numbers were relatively low at this point, these were then manually pre-screened to ensure a meaningful connection between health and climate change within the KWIC, with reference back to the main text of each article where necessary. During this stage, KWICs, with reference back to full articles, were qualitatively coded.

Perhaps the most important finding from the indicator was that the vast majority of KWICs demonstrated little meaningful connection between health and climate change key terms. Instead, they tended to be lists of issues where both health and climate change are present in some form, as demonstrated by the examples below:

- "…the company one of the world's most valuable technology firms stepped down from the board on Friday to focus on philanthropic works related to global **health**, education and **climate change**. The billionaire and his wife Melinda run one of the world's largest charities the Gates Foundation which has billions in assets and…" (HT, 15th March)[283]
- "Trumps whole presidency is built on denying basic realities such as **global warming** and Russian attacks on our politics. Rather than focus on real threats such as **pandemics, climate change** and Russian aggression, the administration is fixated on politically convenient boogeymen such as 'criminal aliens' and Nigerian immigrants" (WP, 27th February)[284]
- "…the California Democrat who is Joseph R. Biden Jr.'s running mate, delivered a comprehensive denunciation of the Trump administration's policies, ranging from the economy and **climate change** to **health care** regulation and taxes. As Ms. Harris attacked Mr. Trump, the vice president..." (NYT, 7th October)[285]

Despite being an important finding, and worthy of reporting here, such observations were regarded as false positives as the only connection between them was their shared status as a key issue. With these taken into account, 331 of the 403 (82%) deduplicated KWICs were discarded and only 18% (72) retained.

Structural topic models

Employing the *stm* package,[286] the remaining KWIC results, within a data-frame, were then used for topic modelling. The specific form of topic model employed was a structural topic model, as this not only discovers topics across documents, but also 'estimate[s] their relationship to document metadata', such as news source and date, which help to 'explain topic prevalence'.[286]

Using a combination of automated functions (*SearchK* in *stm*, for example) and iterative manual searching of model results, the number of topics was selected. For the final model, eight topics were selected as this seemed to provide the most stable and consistent probabilities for terms and could be effectively corroborated in manual searching and were in line with the earlier qualitative coding.

The results of a number of models (with a different number of topics) were shared and discussed by indicator authors and the lowest number was settled upon. Also shared and discussed, for validation purposes, were the original articles and the qualitative coding. Both authors agreed on the final model and its representativeness of the data, the labels for which can be seen in Table 51.

Table 51. Topic labels for final topic model (k=8).

Topic Number	Topic Content
1	Infectious diseases and climate change: particular focus on expanded range
2	Biodiversity loss/ecology change and climate change: infectious diseases and health
3	Health impacts of heat: heatwaves
4	Science and scientific reports on climate change and health
5	Health impacts of heat, wildfires and wildfire smoke
6	Heat-related deaths/mortality
7	COVID-19, pandemics and climate change
8	General impact miscellaneous

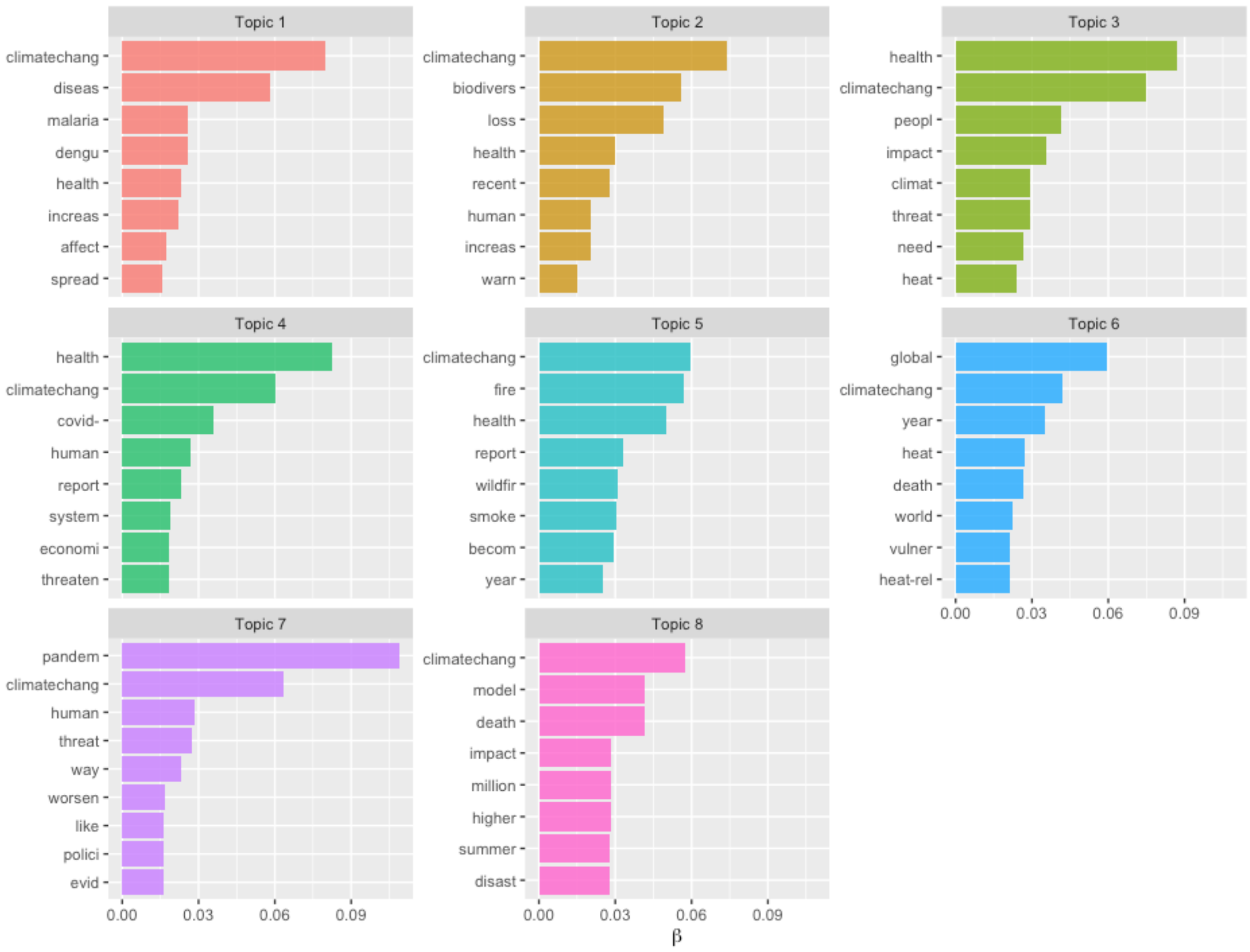

Figure 94. Eight highest word probabilities (beta) for each topic.

Data

1. Newspaper articles in *Hindustan Times*, *Times of India*, *New York Times*, *Washington Post*. Articles analysed for the whole of 2020. This cannot be made publicly available due to copyright restrictions, however the full search strings applied, together with the databases used, are detailed above.

Caveats

This analysis is able to provide a broad picture of how health and climate change are being reported in the target news sources and time points. The selected newspapers cannot be taken to be representative of reporting across the two countries (USA and India) or the WHO regions in which they are located, given that different media sources are known to have widely diverging positions on climate change. The procedure used is intended to identify themes in reporting at the intersection of health and climate change; it is not intended to provide insights into the more general ways in which climate change and/or health are reported in news media.

The articles returned are necessarily those in which there was found to be a conjunction of a pre-selected health term and climate change term. The exact search terms used are likely to have influenced the types of articles obtained.

There are significant differences between the new methodological approach and the previous one, with each having relative advantages and disadvantages. The advantages of the new approach include a less labour-intensive process, the capacity to work with more data in one iteration and the potential to observe objective trends from year to year. A disadvantage is that the KWIC 25-word 'window' utilised in the new methodological approach may well miss some intersections between health and climate change because the distance between these terms exceeds 25 words. This may be because an article has a latent health and climate change throughout and may be

more likely, therefore, to use more passive terms than those included in the keywords. The 2022 iteration of the report will explore ways to ensure these are included within the analysis.

Future form of the indicator

For the 2022 Lancet Countdown report, further sources from different countries will be added to the indicator. Enabled by the new methodology, this will extend the range of the indicator and, with more data, will add greater substance to the topic models. Before the next iteration, the indicator leads will explore options regarding the number of false positives, particularly regarding lists of important global issues, that are generated by the search terms.

Additional information

Topic detail and examples

The single topic with the highest prevalence (see Figure 95 below for topic prevalence), **Topic 7**, relates to the coronavirus pandemic, its relation to climate change, and the increasing threat of future pandemics. For example, the Washington Post[287] reports that ‘former U.S. secretary of state John F. Kerry pointed to evidence suggesting climate change could be a "threat multiplier" for zoonotic and pandemic diseases’. The New York Times (27th October) cites Harvard School of Public Health, saying that ‘the same forces that are worsening climate change are also increasing the risk of future pandemics’.[288] In a clear illustration of the links between the risks from pandemics and climate change, the Washington Post (27th April) reports: ‘In a mere four months, the world has been brought to its knees by a previously unknown virus. But covid-19 won't be the last, or perhaps even the deadliest, pandemic… At the same time, population growth, urbanization, globalization, climate change, the relentless destruction of wildlife habitats and the harvesting of wild species have brought these viruses in closer contact with humans than ever before. Pandemics may become the new normal’.[289]

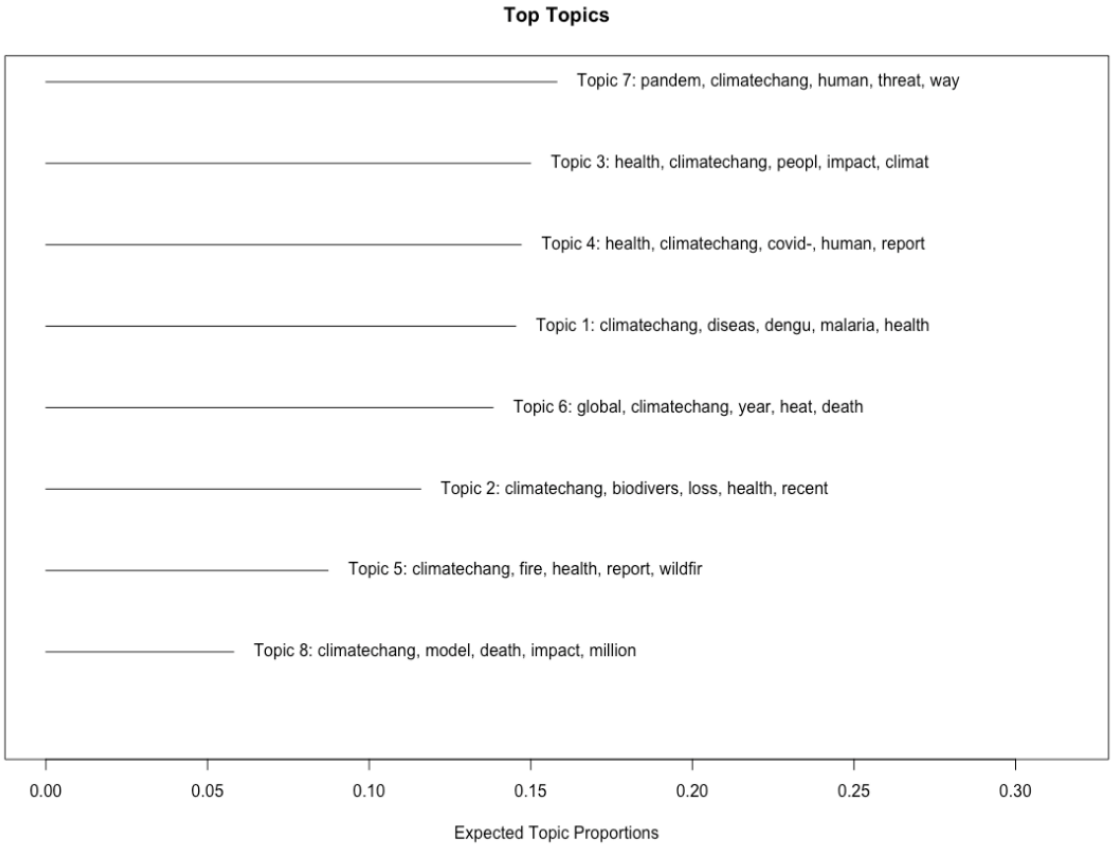

Figure 95. Topic prevalence.

Topics 1, 2, and 4 also overlap to some extent with **Topic 7**, in that they deal with they deal with the spread and ranges of infectious diseases (**1**), the impact of biodiversity loss coinciding with climate change that together expands favourable conditions for disease vectors (**2**) and scientific reports that illustrate these points (**4**). As is clear in Figure 96 **Topic 1** and **Topic 2** are significantly associated (p<.001) with Indian sources.

Topic 1 relates predominantly to the rise and spread of infectious diseases. For example, citing a specialist, the Hindustan Times (25th February) reports that "climate change may revert back successes of controlling infectious diseases. Diseases like dengue and malaria, even cholera, are on the rise".[290] Elsewhere the Hindustan Times (19th July) reports that 'Aedes aegypti, the mosquito that spreads dengue, is most prevalent in cities, and experts have warned in the past that increased urbanisation and warming temperatures due to climate change means that its range will keep increasing'.[291]

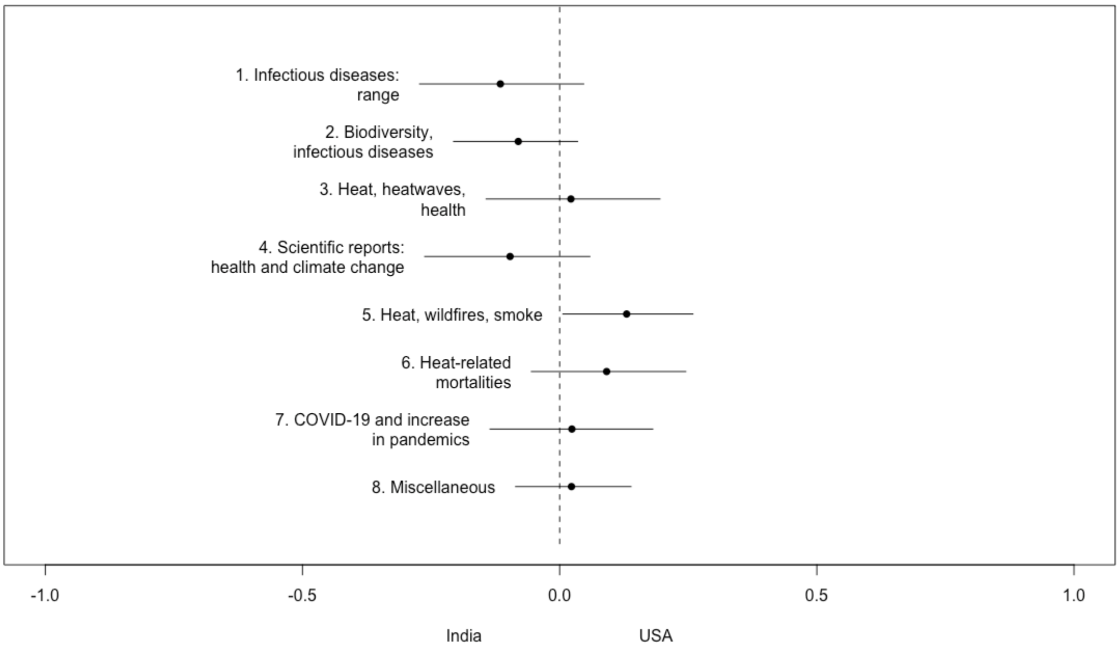

Figure 96. Graphic display of topical prevalence contrast between Indian and USA sources.

Topic 2 focuses more heavily on the role biodiversity loss plays in this process: for example, citing the World Wildlife Fund, the Washington Post (11th September) discusses how this is driven primarily by 'dysfunctional ecosystems', themselves a result of 'habitat loss… along with pollution, invasive species, overhunting and overfishing and, increasingly, climate change'.[292] As with other articles, and the basis of much of **Topics 4** and **7**, the article goes on to cite the director general of WWF who argues that "Covid-19 is a clear manifestation of our broken relationship with nature and highlights the deep interconnection between the health of both people and the planet". The Hindustan Times (15th March) relates this specifically to other infectious diseases: 'There is consensus among scientists that there has been a rise in zoonotic diseases - Nipah, Ebola, Zika, Corona viruses - in recent decades and scientific analysis is increasingly suggesting these are driven by biodiversity loss and climate change'.[293]

Topic 4 is built predominantly around scientific reports. The Lancet Countdown is the most prominent academic source cited and its discussion of interlinking crises (COVID-19 and climate change) permeates the media accounts. For example, in the Hindustan Times (3rd December) a section of the Lancet Countdown is recounted verbatim before saying: 'The report underlined that Covid-19 and climate change were interlinked crises'.[294] Other articles approached the science on climate change and health from a more general stance: 'Science is screaming to us that we are close to running out of time — approaching a point of no return for human health, which depends on planetary health. Addressing climate change and Covid-19 simultaneously and at enough scale requires a response stronger than any seen before to safeguard lives and livelihoods' (NYT 28th April).[295]

Topic 3, 5 and **6** relate predominantly to heat-related impacts (including mortalities) and the health implications of wildfires and wildfire smoke.

Topic 3 largely pertains to the health impacts of heatwaves. The New York Times (18th June), for example, explains that ‘people with health issues, older people and young children are especially susceptible to the effects of extreme heat’, and that ‘it’s a threat that grows as climate change continues’.[296] Likewise, the Washington Post (30th June) picks up on a specific vulnerability to heatwaves, citing the director of Harvard’s School of Public Health: “Heatwaves are getting worse with climate change… We need to be mindful when medications mix with heat. Too much heat can make an otherwise safe and effective drug dangerous”.[297]

Topic 5 involves the impact of extreme events, such as wildfires, and is significantly associated ($p<0.05$) with USA sources (Figure 96. Graphic display of topical prevalence contrast between Indian and USA sources.). For example, the New York Times (16th September) report on their coverage ‘that focused heavily on the links between wildfires and climate change’, looking at ‘the health risks associated with all of that choking smoke’.[298] They later report on the Lancet Countdown’s United States policy brief which ‘presents climate change as a public health risk’, pointing to ‘the immediate dangers of extreme heat, wildfires and air pollution’ (NYT 2nd December).[299] The Washington Post (12th November) outline the anger of Trump’s White House with the suggestion from National Oceanic and Atmospheric Administration that ‘human-caused climate change already was fueling deadlier wildfires, increasingly intense hurricanes and brutal heat waves’ likely to pose a ‘severe threat to American’s health and pocketbooks’.[300] They also report on the championing of the coal industry by the Australian Prime Minister: ‘Meanwhile, [Scott] Morrison and his allies have approved a huge new coal mine in Queensland. The debate about climate change does not seem abstract to people who cannot go for a jog without risking their health because of the heavy smoke that hangs over the country's most populated cities’ (WP 9th January).[301]

Topic 6 is less clear than **Topics 3** and **5** but includes strong links to heat-related deaths and the risks of extreme temperatures. For example, the Washington Post (23rd August) report that ‘[o]fficials in Maricopa County, Ariz., where the mercury has hit 115 degrees a record-breaking eight times this summer, are already investigating at least 243 deaths linked to heat illness - 111 more cases than were recorded at this point last year. "This is climate change," said Susan Clark, a heat expert and director of the Sustainability Initiative at the University at Buffalo’.[302] Data from the 2020 Lancet Countdown report is utilised by two separate sources to make similar points, though narrowed to their own national context. The first is from the New York Times (2nd December): ‘In an annual report on climate change and health published by The Lancet, authors collaborating from around the world say that global warming has already caused a 50 percent increase in heat-related deaths of people older than 65, especially in Japan, China, India and parts of Europe. In the U.S., rising temperatures, combined with pollution and wildfires, are endangering the health of Americans, with fatal consequences for many older people’.[303] The second is from the Times of India (2nd December): ‘India reported over 31,000 heat-related deaths of people older than 65 years in 2018, the second highest in the world after China (62,000), a new Lancet Countdown report on health and climate change shows. While heat-related mortality among people above 65 has increased by 53.7% globally during the past 20 years, it more than doubled in India’.[303,304]

Indicator 5.2: Individual Engagement in Health and Climate Change

Methods

This indicator provides an individual-level indicator of public engagement. It tracks engagement with climate change and health through people's usage of the online encyclopedia Wikipedia. Over the years, Wikipedia has grown to be a major and trusted source of information that has outpaced traditional encyclopedias in terms of reach, coverage, and comprehensiveness.[305] It is regularly listed among the ten most-visited websites worldwide.[306] The English edition covers more than six million articles and over 130,000 active editors. People around the world use it to engage in topics they are interested in. Fortunately, the traffic that goes to Wikipedia – and even that which goes to individual articles of the encyclopedia – can be analysed over time because the Wikimedia foundation makes these statistics available to everyone for free. This makes it a global indicator of what people pay attention to on a daily basis. What is more – and of particular relevance in the context of this report – the platform's health content makes it one of the most frequently used resources for information on health on the internet.[307]

The indicator

To investigate to what extent people do not only pay attention to climate change and human health in isolation, but also to the connection between both, *clickstream statistics* from the English Wikipedia were drawn upon.

Clickstream refers to a dataset provided by the Wikimedia foundation.[4] It reports "streams of clicks", or in other words: how people get to a Wikipedia article and what links they click on. This is reported on a monthly basis and in pairs of resources, the first being where the visit came from, the second which page was visited. This provides an indicator of monthly-level global attention towards one issue (if both articles are representative of the same issue) or two issues (if articles come from different domains, such as climate change and health). By looking at climate change – health articles pairs, an indicator of attention towards climate change consequences for human health over time can be generated.

Measurement strategy

The approach to using clickstream data as an indicator of public engagement in climate change and health is based on the following premises: (1) The Wikipedia platform is a globally used source for information on a multitude of topics.[5] (2) Citizens use the platform to inform themselves about topics they are interested in. (3) By tracking engagement with Wikipedia articles that are related to climate change as wells as with articles on health, it is possible to identify public engagement with the relationship between both topics.

The following behavioural patterns are relevant for the validity of the measure as a proxy for public engagement with climate change and health:

a) A person is generally interested in the nexus between climate change and public health and informs her/himself about the topic online by, e.g., reading the Wikipedia article on *Effects of global warming on human health* (https://en.wikipedia.org/wiki/Effects_of_global_warming_on_human_health).
b) A person is interested in climate change and the consumption of information about the topic then sparks interest in its consequences for human health. For instance, the person reads the article on *Global warming* (https://en.wikipedia.org/wiki/Global_warming) and then turns to the article on *Malnutrition* (https://en.wikipedia.org/wiki/Malnutrition) .
c) A person is interested in a certain aspect of human health or consequences of climate change with an immediate impact on human health, and then turns its attention to climate change issues. For instance, the person reads the article on *Malaria* (https://en.wikipedia.org/wiki/Malaria) and then turns to the article on *Global warming* (https://en.wikipedia.org/wiki/Global_warming).

[4] See https://meta.wikimedia.org/wiki/Research:Wikipedia_clickstream.
[5] See https://stats.wikimedia.org/wikimedia/squids/SquidReportPageViewsPerCountryOverview.htm for an overview of Wikipedia usage by country and languages.

Indicator construction

In order to use the Wikipedia viewership statistics as a proxy for public engagement with climate change and health, it is key to select articles that are representative of these topics. To generate the populations of articles related to climate change on the one hand and health on the other, a semi-automated approach was implemented. Based on an initial set of keywords[6], related articles were searched for using the internal Wikipedia search.

For each search using one of the keywords, the first 100 results were extracted and identified that led to an article with a minimum word count of 300, ensuring that the articles that were chosen as seed articles had been given a certain degree of attention by Wikipedia editors, therefore being more likely to link to other relevant articles.

Next, the articles collected via the Wikipedia search for categories were screened, which are used on the Wikipedia to categorize pages in a meaningful way (e.g., using categories such as *Climate change* or *Effects of global warming*). Those categories were then themselves screened for relevant articles. All additional articles were once more filtered such that those with a title matching one of the initial keywords was chosen. For the health-related articles, several articles were excluded manually that turned out to be irrelevant for our purposes. Health topics are covered extensively on the Wikipedia, but it was important to prioritize articles and topics that, in principle, can be related to climate change. In addition, the fact that the Wikipedia page on the effects of global warming on human health[7] offers a variety of links to further health-related articles was exploited, as it can be seen as a curated list of relevant health articles and added those links to the list. All in all, 551 articles related to climate change and 1,414 articles related to health were identified that were seen as being representative for either of the issues. The complete list of articles is listed under *Additional Information*.

For the clickstream analysis, the set of articles were extended by also taking "second-level pages" into account, that is pages that are linked to in the set of 610 climate change or 1414 health articles and that are also somewhat related to climate change or health. Sometimes, people might not directly jump from one of the major articles on climate change to another one on health, but travel through an intermediary page (e.g., a possible individual stream of clicks could be: *Climate change* → *Human impact on the environment* → *Respiratory disease*). The clickstream data only allow the identification of click volume for pairs of articles, but by extending the network, clickstreams could also be captured involving relevant pages that are linked in the original set of articles. After taking these additional articles into account, 2,855 articles related to climate change and 11054 articles related to health were identified. This compares to 1,837 articles related to climate change and 6902 articles related to health identified in the 2020 report.

Technically, the fact that the number of health articles is far larger than the number of climate change articles does not invalidate the measurement strategy. It seems plausible that there are many more articles on health-related than on climate change-related topics because the health field is much broader (which is one reason why the health articles cluster in the network plot is not that dense – some health topics are really far apart from each other, although both could be covering health issues that are affected by climate change). But this should not directly affect the metrics. Even if there are many more health than climate change articles, it could still be that health topics are mentioned (and clicked on) much more often in climate change articles than the other way around. To sum up, what is key in the analysis is not that one or the other topic is more extensively covered on the platform, but the co-visit patterns.

Data

Publicly available data from the Wikimedia foundation were drawn upon. Data from all platforms, i.e. accesses to the Wikipedia via desktop machines, mobile browsers, and mobile apps were considered.

The clickstream data were downloaded from the Wikimedia Dumps (https://dumps.wikimedia.org/other/clickstream/). Spider traffic (i.e. traffic generated by automated bots crawling

[6] For climate change articles, the keywords were *climate change, warming, ipcc*, and green house, and *greenhouse*. For health articles, the seed keywords were *epidemy, disease, malaria, diarrhoea, infection, sars, measles, pneumonia, epidemic, pandemic, public health, health care, healthcare, epidemiology, mortality, morbidity, nutrition, illness, infectious, ncd, non-communicable disease, noncommunicable disease, communicable disease, air pollution, nutrition, malnutrition, mental disorder,* and *stunting*.
[7] See https://en.wikipedia.org/wiki/Effects_of_global_warming_on_human_health.

the platform) is excluded. Referer-resource pairs (i.e. the pairs of the article of origin and the target article) that had less than 10 clicks were removed in the original dataset, suggesting a slight underreporting in the actual clickstream traffic. However, this is not expected to add any systematic bias to the indicators, in particular since interest is mainly in changes of engagement over time.

Clickstream data are available from November 2017 onwards. In this report, the indicator focuses on data from 2018 to 2020. The analyses are limited to the English Wikipedia.

The benefits of the Wikipedia usage metadata for the purpose of tracking public engagement in climate change and health are that these data (a) are globally available, (b) cover the time period of interest, (c) are collectible at virtually no cost, and, most importantly, (e) have high face validity to measure engagement in this very specific topic. Reading articles on Wikipedia is motivated by attention towards a particular issue. Individuals invest time to inform themselves about a topic, which is one manifestation of engagement. Aggregate reading behaviour can therefore be seen as an a priori valid approximation of public issue engagement.

Caveats

All clickstream information is only available at the aggregate level. It is not possible to link the data to information about individuals who visited the platform. Also, the data are not geo-referenced, so it is not possible to infer where page visits came from. Although the English Wikipedia is predominantly used in English-speaking countries (according to the Wikimedia Traffic Analysis Report[8], about 40% of the traffic on the English Wikipedia comes from the United States), it is a globally popular resource. It makes up for 50% of the global traffic to all Wikipedia language editions. Therefore, it can be seen as a global indicator of public attention that is somewhat biased towards attention from countries such as the United States, United Kingdom, India, Canada, and Australia. Extending the analyses to other language editions will help to remedy this bias and uncover potential geographic engagement heterogeneity in the future.

More generally, the measure represents an online proxy for an offline phenomenon. In addition, it is sensitive towards the selection of articles used to capture engagement. The global popularity of the platform, which consistently ranks among the ten most visited websites worldwide, speaks in favour of its usefulness for this application. However, more direct indicators of public engagement, such as survey-based measures, might provide a useful supplement and source for validation in the future.

While the data are available for free, access to future data depends on the Wikimedia API. There is no indication of Wikimedia restricting access in the future. Instead, Wikimedia has invested in data quality and making access more robust and convenient.

Future form of indicator

Beyond the 2021 Lancet Countdown report, analyses of individual-level engagement using pageview data from Wikimedia will be undertaken. In time, the indicator may draw on both clickstream and pageview data.

There is potential for other steps that will help increase the precision, scope, and value of this indicator for next year's report.

First, a plan is in place to increase the number of articles used. With an ever-growing Wikipedia, more relevant articles might become available. This requires a joint automated and human classification effort to ensure that the coverage of relevant articles (true positives) is as large as possible and the number of irrelevant articles (false positives) in the sample minimal.

Second, the extension of data collection and analysis efforts to other language editions (both for the pageviews and the clickstream data). This would make it possible to track more fine-grained trends at the regional level. It is likely that there is heterogeneity in public engagement in climate change and health, as different regions of the world are currently affected by health consequences of climate change to a varying degree. Studying engagement in different language versions of the Wikipedia could at least partly pick up this heterogeneity.

---

[8] See https://stats.wikimedia.org/wikimedia/squids/SquidReportPageViewsPerLanguageBreakdown.htm.

Third, the indicator team will explore complementary data to track and validate public attention, such as survey, experimental, and other online data.

Additional information

List of English Wikipedia articles used to track public engagement in climate change

100% renewable energy, 1998 United Nations Climate Change Conference, 2001 United Nations Climate Change Conference, 2002 United Nations Climate Change Conference, 2003 United Nations Climate Change Conference, 2004 United Nations Climate Change Conference, 2005 United Nations Climate Change Conference, 2006 United Nations Climate Change Conference, 2007 United Nations Climate Change Conference, 2008 United Nations Climate Change Conference, 2009 United Nations Climate Change Conference, 2010 United Nations Climate Change Conference, 2011 United Nations Climate Change Conference, 2012 United Nations Climate Change Conference, 2013 United Nations Climate Change Conference, 2014 United Nations Climate Change Conference, 2015 United Nations Climate Change Conference, 2016 United Nations Climate Change Conference, 2017 United Nations Climate Change Conference, 2018 United Nations Climate Change Conference, 2019 in climate change, 2019 UN Climate Action Summit, 2019 United Nations Climate Change Conference, 2020 in climate change, 2021 in climate change, 2021 United Nations Climate Change Conference, Abrupt climate change, Academy of Climate Change Education and Research, Advisory Group on Greenhouse Gases, Alice, the Zeta Cat and Climate Change, Attribution of recent climate change, Australian Greenhouse Office, Australian Renewable Energy Agency, Aviation and climate change, Avoiding Dangerous Climate Change (2005 conference), Bangladesh Climate Change Resilience Fund, Bangladesh Climate Change Trust, Boulder Climate Action Plan, Business action on climate change, California Climate Action Registry, Campaign against Climate Change, Campus carbon neutrality, Carbon dioxide, Carbon dioxide (data page), Carbon dioxide angiography, Carbon dioxide cleaning, Carbon dioxide flooding, Carbon dioxide in Earth's atmosphere, Carbon Dioxide Information Analysis Center, Carbon dioxide laser, Carbon dioxide reforming, Carbon dioxide removal, Carbon dioxide scrubber, Carbon neutrality, Carbon-neutral fuel, CCS and climate change mitigation, Center for the Study of Carbon Dioxide and Global Change, Centre for Climate Change Economics and Policy, Centre for Renewable Energy, Centre for Renewable Energy Systems Technology, Chesapeake Climate Action Network, Chicago Climate Action Plan, Civil Society Coalition on Climate Change, Climate Action Network, Climate Action Network Latin America, Climate and Clean Air Coalition to Reduce Short-Lived Climate Pollutants, Climate change, Climate Change – The Facts, Climate Change (Scotland) Act 2009, Climate Change Accountability Act (Bill C-224), Climate change acronyms, Climate Change Act 2008, Climate change adaptation, Climate change adaptation strategies on the German coast, Climate Change Agreement (UK), Climate change and agriculture, Climate change and agriculture in the United States, Climate change and birds, Climate change and cities, Climate change and ecosystems, Climate Change and Emissions Management Amendment Act, Climate change and fisheries, Climate change and gender, Climate change and indigenous peoples, Climate change and infectious diseases, Climate change and invasive species, Climate change and potatoes, Climate change and poverty, Climate Change and Sustainable Energy Act 2006, Climate change art, Climate Change Authority, Climate Change Commission, Climate Change Committee, Climate change denial, Climate Change Denial, Climate Change Denial Disorder, Climate change education, Climate change feedback, Climate change in Alabama, Climate change in Alaska, Climate change in Algeria, Climate change in American Samoa, Climate change in Antarctica, Climate change in Argentina, Climate change in Arizona, Climate change in Arkansas, Climate change in Australia, Climate change in Bangladesh, Climate change in Belgium, Climate change in Brazil, Climate change in California, Climate change in Canada, Climate change in China, Climate change in Colorado, Climate change in Connecticut, Climate change in Cyprus, Climate change in Delaware, Climate change in Europe, Climate change in Finland, Climate change in Florida, Climate change in France, Climate change in Georgia (U.S. state), Climate change in Germany, Climate change in Ghana, Climate change in Greenland, Climate change in Grenada, Climate change in Guam, Climate change in Guatemala, Climate change in Honduras, Climate change in Idaho, Climate change in Illinois, Climate change in India, Climate change in Indiana, Climate change in Indonesia, Climate change in Iowa, Climate change in Iraq, Climate change in Japan, Climate change in Jordan, Climate change in Kansas, Climate change in Kentucky, Climate change in Kenya, Climate change in Kyrgyzstan, Climate change in Liberia, Climate change in Louisiana, Climate change in Luxembourg, Climate change in Maine, Climate change in Maryland, Climate change in Massachusetts, Climate change in Mexico, Climate change in Michigan, Climate change in Minnesota, Climate change in Mississippi, Climate change in Missouri, Climate change in Montana, Climate change in Morocco, Climate change in Nebraska, Climate change in Nepal, Climate change in Nevada, Climate change in New Hampshire, Climate change in New Jersey, Climate change in New Mexico, Climate change in New York (state), Climate change in New York City, Climate change in New Zealand, Climate change in Nigeria, Climate change in North Carolina, Climate change in North Dakota, Climate change in North Korea, Climate change in Norway, Climate change in Ohio, Climate change in Oklahoma, Climate change in Oregon, Climate change in Pakistan, Climate change in popular culture, Climate change in Puerto Rico, Climate change in Rhode Island, Climate change in Russia, Climate change in Saskatchewan, Climate change in Scotland, Climate change in Senegal, Climate change in South Africa, Climate change in South Asia, Climate change in South Carolina, Climate change in South Dakota, Climate change in South Korea, Climate change in Sri Lanka, Climate

change in Suriname, Climate change in Sweden, Climate change in Taiwan, Climate change in Tanzania, Climate change in Tennessee, Climate change in Texas, Climate change in the Arctic, Climate change in the Caribbean, Climate change in the Gambia, Climate change in the Middle East and North Africa, Climate change in the Netherlands, Climate change in the United Kingdom, Climate change in the United States, Climate change in Turkey, Climate change in Tuvalu, Climate change in Utah, Climate change in Vermont, Climate change in Vietnam, Climate change in Virginia, Climate change in Washington, Climate change in Washington, D.C., Climate change in West Virginia, Climate change in Wisconsin, Climate change in Wyoming, Climate Change Levy, Climate change mitigation, Climate change mitigation framework, Climate change mitigation scenarios, Climate change opinion by country, Climate Change Performance Index, Climate change policy of California, Climate change policy of the George W. Bush administration, Climate change policy of the United States, Climate Change Response (Emissions Trading) Amendment Act 2008, Climate Change Response (Zero Carbon) Amendment Act, Climate Change Response Act 2002, Climate change scenario, Climate Change Science Program, Climate Change TV, Climate change vulnerability, Climate Change: Global Risks, Challenges and Decisions, Climate crisis, Climate emergency declaration, Climate emergency declarations in Australia, Climate emergency declarations in the United Kingdom, Climate variability and change, Cloud formation and climate change, Committee on Climate Change Science and Technology Integration, Cool It: The Skeptical Environmentalist's Guide to Global Warming, Copper in renewable energy, Debate over China's economic responsibilities for climate change mitigation, Decarbonisation measures in proposed UK electricity market reform, Deep Decarbonization Pathways Project, Deforestation and climate change, Economic impacts of climate change, Economics of climate change, Economics of climate change mitigation, Economists' Statement on Climate Change, Effects of climate change, Effects of climate change on human health, Effects of climate change on humans, Effects of climate change on island nations, Effects of climate change on marine mammals, Effects of climate change on oceans, Effects of climate change on plant biodiversity, Effects of climate change on terrestrial animals, Effects of climate change on wine production, Effects of global warming, Effects of global warming on human health, Effects of global warming on humans, Effects of global warming on the United Arab Emirates, Electrochemical reduction of carbon dioxide, Euro-Mediterranean Center on Climate Change, European Climate Change Programme, European Renewable Energy Council, Extreme weather, ExxonMobil climate change controversy, G8 Climate Change Roundtable, Garnaut Climate Change Review, German Climate Action Plan 2050, German Renewable Energy Sources Act, Global Climate Action (portal), Global Climate Action Summit, Global Roundtable on Climate Change, Global temperature record, Global warming conspiracy theory, Global warming controversy, Global warming game, Global warming hiatus, Global Warming Policy Foundation, Global Warming Pollution Reduction Act of 2007, Global warming potential, Global Warming Solutions Act of 2006, Global Warming: The Signs and The Science, Global Warming: What You Need to Know, Glossary of climate change, Great March for Climate Action, Greenhouse and icehouse Earth, Greenhouse debt, Greenhouse Development Rights, Greenhouse effect, Greenhouse gas, Greenhouse gas emissions by Australia, Greenhouse gas emissions by China, Greenhouse gas emissions by Russia, Greenhouse gas emissions by the United Kingdom, Greenhouse gas emissions by the United States, Greenhouse gas emissions by Turkey, Greenhouse gas emissions in Kentucky, Greenhouse gas inventory, Greenhouse gas monitoring, Greenhouse Gas Pollution Pricing Act, Greenhouse Gases Observing Satellite, Greenhouse Mafia, Gussing Renewable Energy, History of climate change policy and politics, History of climate change science, How Global Warming Works, How to Prepare for Climate Change, Human rights and climate change, Hybrid renewable energy system, Illustrative model of greenhouse effect on climate change, Index of climate change articles, Indian Network on Climate Change Assessment, Indigenous Peoples Climate Change Assessment Initiative, Individual action on climate change, Individual and political action on climate change, Intergovernmental Panel on Climate Change, Interim Climate Change Committee, International Climate Change Partnership, International Conference on Climate Change, International Indigenous Peoples Forum on Climate Change, International Journal of Greenhouse Gas Control, International Renewable Energy Agency, IPCC list of greenhouse gases, Johannesburg Renewable Energy Coalition, King Abdullah City for Atomic and Renewable Energy, Life-cycle greenhouse gas emissions of energy sources, Liquid carbon dioxide, List of authors of Climate Change 2007: The Physical Science Basis, List of books about renewable energy, List of climate change books, List of climate change initiatives, List of countries by carbon dioxide emissions, List of countries by carbon dioxide emissions per capita, List of countries by greenhouse gas emissions, List of countries by greenhouse gas emissions per person, List of extreme temperatures in Australia, List of extreme temperatures in Denmark, List of extreme temperatures in Finland, List of extreme temperatures in France, List of extreme temperatures in Germany, List of extreme temperatures in Italy, List of extreme temperatures in Portugal, List of extreme temperatures in Spain, List of extreme temperatures in Sweden, List of extreme temperatures in Vatican City, List of films about renewable energy, List of ministers of climate change, List of people associated with renewable energy, List of renewable energy companies by stock exchange, List of renewable energy organizations, List of renewable energy topics by country and territory, List of U.S. states and territories by carbon dioxide emissions, Lists of renewable energy topics, London Climate Change Agency, Long-term effects of global warming, Mandatory renewable energy target, Mayors National Climate Action Agenda, Media coverage of climate change, Mercator Research Institute on Global Commons and Climate Change, Midwestern Greenhouse Gas Reduction Accord, Migration and global environmental change (Report), Minister for Climate Change (New Zealand), Ministry of Climate Change (Pakistan), Ministry of Environment, Forest and Climate Change, Muslim Seven Year Action Plan on Climate Change, Mycorrhizae and changing climate, National Action Plan for Climate Change, National Climate Change Secretariat, National Renewable Energy

Action Plan, National Solar Conference and World Renewable Energy Forum 2012, New England Governors and Eastern Canadian Premiers Climate Change Action Plan 2001, New South Wales Greenhouse Gas Abatement Scheme, New York City Panel on Climate Change, Nigeria Renewable Energy Master Plan, Nongovernmental International Panel on Climate Change, Nuclear power proposed as renewable energy, Office of Energy Efficiency and Renewable Energy, Ozone depletion and climate change, Pan-African Media Alliance on Climate Change, Pan-Canadian Framework on Clean Growth and Climate Change, Pastoral Greenhouse Gas Research Consortium, Photochemical reduction of carbon dioxide, Photoelectrochemical reduction of carbon dioxide, Physical impacts of climate change, Physical properties of greenhouse gases, Poland National Renewable Energy Action Plan, Politics of climate change, Portal:Climate change, Portal:Renewable energy, Post–Kyoto Protocol negotiations on greenhouse gas emissions, Premier's Climate Change Council, Presbyterian Church (U.S.A.) Carbon Neutral Resolution, Presidential Climate Action Plan, Psychological impact of climate change, Psychology of climate change denial, Public opinion on climate change, Punjab Renewable Energy Systems Pvt. Ltd., R20 Regions of Climate Action, Rapid Climate Change-Meridional Overturning Circulation and Heatflux Array, Regional climate change initiatives in the United States, Regional effects of climate change, Regional Greenhouse Gas Initiative, Regulation of greenhouse gases under the Clean Air Act, Renewable energy, Renewable Energy (journal), Renewable energy and mining, Renewable Energy Association, Renewable Energy Certificate (United States), Renewable Energy Certificate System, Renewable Energy Certificates Registry, Renewable energy commercialization, Renewable energy cooperative, Renewable energy debate, Renewable energy in Brunei, Renewable energy in developing countries, Renewable energy in Luxembourg, Renewable energy industry, Renewable Energy Payments, Renewable energy policy of Bangladesh, Renewable energy sculpture, Renewable Energy Sources and Climate Change Mitigation, Renewable energy transition, Ringed seals and climate change, Runaway greenhouse effect, San Diego Climate Action Plan, San Francisco Climate Action Plan, Scientific consensus on climate change, Scorcher: The Dirty Politics of Climate Change, Seawater greenhouse, Soft climate change denial, Solar Renewable Energy Certificate, Space-based measurements of carbon dioxide, Special Report on Climate Change and Land, Special Report on Global Warming of 1.5 °C, Special Report on the Ocean and Cryosphere in a Changing Climate, State Agency on Alternative and Renewable Energy Sources (Azerbaijan), Supercritical carbon dioxide, Sweden National Renewable Energy Action Plan, Tarawa Climate Change Conference, Template:Climate change in Canada, Template:United Nations climate change conferences, Territorial Approach to Climate Change, The Great Derangement: Climate Change and the Unthinkable, The Great Global Warming Swindle, The Greenhouse Conspiracy, The Islamic Declaration on Global Climate Change, The Real Global Warming Disaster, Tropical cyclones and climate change, U.S. Climate Action Partnership, U.S. Climate Change Technology Program, United Kingdom Climate Change Programme, United Kingdom National Renewable Energy Action Plan, United Nations Climate Change conference, United Nations Special Envoy on Climate Change, United States federal register of greenhouse gas emissions, United States House Select Committee on Energy Independence and Global Warming, United States House Select Committee on the Climate Crisis, UNSW School of Photovoltaic and Renewable Energy Engineering, Urbanization and Global Environmental Change Project, Variable renewable energy, Wadebridge Renewable Energy Network, Waterborne disease and climate change, Weyburn-Midale Carbon Dioxide Project, White House Office of Energy and Climate Change Policy, Women in climate change, World Climate Change Conference, Moscow, World Mayors Council on Climate Change, World People's Conference on Climate Change, World Renewable Energy Network, World Wide Views on Global Warming, Yale Program on Climate Change Communication, Young Voices on Climate Change

List of English Wikipedia articles used to track public engagement in health

1493 Hispaniola influenza epidemic, 1510 influenza pandemic, 1557 influenza pandemic, 1775–1782 North American smallpox epidemic, 1793 Philadelphia yellow fever epidemic, 1812–1819 Ottoman plague epidemic, 1813–1814 Malta plague epidemic, 1817–1824 cholera pandemic, 1826–1837 cholera pandemic, 1837 Great Plains smallpox epidemic, 1846–1860 cholera pandemic, 1847 North American typhus epidemic, 1862 Pacific Northwest smallpox epidemic, 1863–1875 cholera pandemic, 1881–1896 cholera pandemic, 1889–1890 pandemic, 1896 Gloucester smallpox epidemic, 1899–1923 cholera pandemic, 1906 malaria outbreak in Ceylon, 1915 typhus and relapsing fever epidemic in Serbia, 1918 flu pandemic in India, 1924–1925 Minnesota smallpox epidemic, 1929–1930 psittacosis pandemic, 1957–1958 influenza pandemic, 1961–1975 cholera pandemic, 1974 smallpox epidemic in India, 1985 World Health Organization AIDS surveillance case definition, 1994 expanded World Health Organization AIDS case definition, 1998 Winter Olympics flu epidemic, 2002–2004 SARS outbreak, 2002–2004 SARS outbreak among healthcare workers, 2009 swine flu pandemic, 2009 swine flu pandemic actions concerning pigs, 2009 swine flu pandemic by country, 2009 swine flu pandemic in India, 2009 swine flu pandemic tables, 2009 swine flu pandemic timeline, 2009 swine flu pandemic timeline summary, 2009 swine flu pandemic vaccine, 2013 Swansea measles epidemic, 2015–2016 Zika virus epidemic, 2018 Madagascar measles outbreak, 2019 New York measles outbreak, 412 BC epidemic, 735–737 Japanese smallpox epidemic, Abrazo Community Health Network, Academy of Nutrition and Dietetics, Access Health CT, Accredited Social Health Activist, Action on Smoking and Health, Acute eosinophilic pneumonia, Adenovirus infection, Adult-onset Still's disease, AdventHealth, AdventHealth Celebration, AdventHealth Lake Wales, AdventHealth Ocala, AdventHealth Orlando, AdventHealth Shawnee Mission, AdventHealth Wauchula, Adventist Health,

Adventist Health Bakersfield, Adventist Health Feather River, Adventist Health Glendale, Adventist Health Portland, Affordable Medicines Facility-malaria, Africa Centres for Disease Control and Prevention, Africa Fighting Malaria, Africa/Harvard School of Public Health Partnership for Cohort Research and Training, African Malaria Network Trust, African Nutrition Leadership Programme, Against Malaria Foundation, Aging-associated diseases, Air pollution, Air pollution and traffic congestion in Tehran, Air pollution forecasting, Air pollution in Hong Kong, Air pollution in Macau, Air pollution sensor, Air Quality Health Index (Canada), Airport malaria, Akureyri disease, Alcohol and health, Alexander disease, Alliance for Health Policy and Systems Research, Alliance for Healthy Cities, AllianceHealth Durant, Alveolar hydatid disease, Alzheimer's disease, Alzheimer's disease biomarkers, Alzheimer's Disease Cooperative Study, Alzheimer's disease in the media, Alzheimer's Disease Neuroimaging Initiative, Amazon Malaria Initiative, American Association for the Study and Prevention of Infant Mortality, American Association of Public Health Dentistry, American Association of Public Health Physicians, American College of Epidemiology, American Journal of Epidemiology, American Public Health Association, American School Health Association, American Sexual Health Association, American Society for Nutrition, American Society for Parenteral and Enteral Nutrition, AMITA Health, Anaerobic infection, Andersen healthcare utilization model, Animal nutritionist, AnMed Health Medical Center, AnMed Health Women's & Children's Hospital, Annals of Epidemiology, Anterior horn disease, Anti-AQP4 disease, Anti-IgLON5 disease, Anti-neurofascin demyelinating diseases, Antidiarrhoeal, Antimalarial medication, Apparent infection rate, Arab Health, Area Health Education Centers Program, Asia Pacific Leaders Malaria Alliance, Aspiration pneumonia, Association for Community Health Improvement, Association of Medical Microbiology and Infectious Disease Canada, Association of Public Health Laboratories, Atrium Health, Atrium Health Cabarrus, Atrium Health Mercy, Atrium Health Pineville, Atrium Health Union, Atrium Health University City, Atypical pneumonia, Australian Health Protection Principal Committee, Australian Longitudinal Study on Women's Health, Australian Measles Control Campaign, Autoimmune disease, Autoimmune disease in women, Autoimmune inner ear disease, Autoimmune skin diseases in dogs, Autosomal dominant polycystic kidney disease, Autosomal recessive polycystic kidney disease, Avalere Health, Bachelor of Science in Public Health, Bacterial pneumonia, Balwadi Nutrition Programme, Bamrasnaradura Infectious Diseases Institute, Bandim Health Project, Bangladesh Institute of Child and Mother Health, Bangladesh Institute of Child Health, Bangladesh National Nutrition Council, Baptist Health System, Batten disease, Baumol's cost disease, Bayfront Health Punta Gorda, Bayfront Health St. Petersburg, Behavior change (public health), Behçet's disease, Belgian Health Care Knowledge Centre, BENTA disease, Bill of health, Bills of mortality, Binswanger's disease, Biochemistry of Alzheimer's disease, Biologically based mental illness, Biphasic disease, Black Maternal Health Caucus, Black Women's Health Study, Blackheart (plant disease), Blocq's disease, Blood-borne disease, Bloodstream infections, Blount's disease, Bluetongue disease, Bombay plague epidemic, Bone health, Borna disease, Boroughs of Montreal during the COVID-19 pandemic, Brazilian Health Regulatory Agency, Brill–Zinsser disease, British government response to the COVID-19 pandemic, Bronchopneumonia, Brookwood Baptist Health, Busselton Health Study, Caerphilly Heart Disease Study, Calcium pyrophosphate dihydrate crystal deposition disease, California Center for Public Health Advocacy, Camurati–Engelmann disease, Canada Health Act, Canada Health and Social Transfer, Canadian Classification of Health Interventions, Canadian Public Health Association, Canadian Society for Epidemiology and Biostatistics, Canavan disease, Cancer Epidemiology (journal), Cancer Epidemiology, Biomarkers & Prevention, Canine vector-borne disease, Capitation (healthcare), Caribbean Public Health Agency, Carlos III Health Institute, Caroli disease, Carolinas College of Health Sciences, Carolinas HealthCare System Blue Ridge Morganton, Carondelet Health Network, Carrion's disease, Case management (US healthcare system), Castleman disease, Cat-scratch disease, Catheter-associated urinary tract infection, Causes of mental disorders, Center for Global Infectious Disease Research, Center for Infectious Disease Research and Policy, Center for World Health and Medicine, Center of expertise for rare diseases, Central nervous system disease, Centre for Health and International Relations, Centre for Health Protection, Centre for History in Public Health, London School of Hygiene and Tropical Medicine, Certified Health Informatics Systems Professional, Chagas disease, CHAI disease, Charcot–Marie–Tooth disease, Charcot–Marie–Tooth disease classifications, Chicago 1885 cholera epidemic myth, Child Health and Nutrition Research Initiative, Child health in Uganda, Child mortality, Childhood chronic illness, Children's right to adequate nutrition in New Zealand, Chinese Center for Disease Control and Prevention, Chinese Classification of Mental Disorders, Chlamydia pneumoniae, Chloroquine and hydroxychloroquine during the COVID-19 pandemic, Cholera outbreaks and pandemics, Choosing Healthplans All Together, Chronic diseases, Chronic illness, Chronic kidney disease, Chronic Lyme disease, Chronic obstructive pulmonary disease, Cinematography in healthcare, Circle of Health International, Classification of mental disorders, Classification of pneumonia, Climate change and infectious diseases, Clinical epidemiology, Clinical Epidemiology (journal), Clinical Health Promotion, Clinical nutrition, Clinton health care plan of 1993, Clostridioides difficile infection, CNS demyelinating autoimmune diseases, Coalition for Epidemic Preparedness Innovations, Coeliac disease, Cognitive epidemiology, Cohorts for Heart and Aging Research in Genomic Epidemiology, Coinfection, Cold agglutinin disease, Colorado Department of Health Care Policy and Financing, Commission on Health Research for Development, Commission on the Accreditation of Healthcare Management Education, Common disease-common variant, CommonSpirit Health, Community Dentistry and Oral Epidemiology, Community health, Community health agent, Community health centers in the United States, Community Health Clubs in Africa, Community Health Systems, Community-acquired pneumonia, Comorbidity, Comparison of the healthcare systems in Canada and the United States, Compartmental models in epidemiology, Comprehensive Health Assessment Program, Compression of morbidity, Computational

epidemiology, Cone Health, Cone Health Behavioral Health Hospital, Cone Health Women's Hospital, Conflict epidemiology, Congenital cytomegalovirus infection, Congenital malaria, Consortium of Universities for Global Health, Contagious bovine pleuropneumonia, Contagious disease, Convention on Long-Range Transboundary Air Pollution, Core Cities Health Improvement Collaborative, Corn stunt disease, Coronary artery disease, Coronavirus disease 2019, Coronavirus diseases, Coughs and sneezes spread diseases, Council on Education for Public Health, COVID-19 pandemic, COVID-19 pandemic by country and territory, COVID-19 pandemic cases, COVID-19 pandemic death rates by country, COVID-19 pandemic deaths, COVID-19 pandemic in Abkhazia, COVID-19 pandemic in Afghanistan, COVID-19 pandemic in Akrotiri and Dhekelia, COVID-19 pandemic in Albania, COVID-19 pandemic in Algeria, COVID-19 pandemic in American Samoa, COVID-19 pandemic in Andorra, COVID-19 pandemic in Angola, COVID-19 pandemic in Antigua and Barbuda, COVID-19 pandemic in Argentina, COVID-19 pandemic in Armenia, COVID-19 pandemic in Australia, COVID-19 pandemic in Austria, COVID-19 pandemic in Azerbaijan, COVID-19 pandemic in Bahrain, COVID-19 pandemic in Bangladesh, COVID-19 pandemic in Barbados, COVID-19 pandemic in Belarus, COVID-19 pandemic in Belgium, COVID-19 pandemic in Belize, COVID-19 pandemic in Benin, COVID-19 pandemic in Bhutan, COVID-19 pandemic in Bolivia, COVID-19 pandemic in Bosnia and Herzegovina, COVID-19 pandemic in Botswana, COVID-19 pandemic in Brazil, COVID-19 pandemic in Brunei, COVID-19 pandemic in Bulgaria, COVID-19 pandemic in Burkina Faso, COVID-19 pandemic in Burundi, COVID-19 pandemic in Cambodia, COVID-19 pandemic in Cameroon, COVID-19 pandemic in Canada, COVID-19 pandemic in Cape Verde, COVID-19 pandemic in Chad, COVID-19 pandemic in Chile, COVID-19 pandemic in Colombia, COVID-19 pandemic in Costa Rica, COVID-19 pandemic in Crimea, COVID-19 pandemic in Croatia, COVID-19 pandemic in Cuba, COVID-19 pandemic in Cyprus, COVID-19 pandemic in Denmark, COVID-19 pandemic in Djibouti, COVID-19 pandemic in Dominica, COVID-19 pandemic in East Timor, COVID-19 pandemic in Easter Island, COVID-19 pandemic in Ecuador, COVID-19 pandemic in Egypt, COVID-19 pandemic in El Salvador, COVID-19 pandemic in England, COVID-19 pandemic in Equatorial Guinea, COVID-19 pandemic in Eritrea, COVID-19 pandemic in Estonia, COVID-19 pandemic in Eswatini, COVID-19 pandemic in Ethiopia, COVID-19 pandemic in Europe, COVID-19 pandemic in Fiji, COVID-19 pandemic in Finland, COVID-19 pandemic in France, COVID-19 pandemic in French Polynesia, COVID-19 pandemic in Gabon, COVID-19 pandemic in Gagauzia, COVID-19 pandemic in Georgia (country), COVID-19 pandemic in Germany, COVID-19 pandemic in Ghana, COVID-19 pandemic in Gibraltar, COVID-19 pandemic in Greece, COVID-19 pandemic in Greenland, COVID-19 pandemic in Grenada, COVID-19 pandemic in Guatemala, COVID-19 pandemic in Guernsey, COVID-19 pandemic in Guinea, COVID-19 pandemic in Guinea-Bissau, COVID-19 pandemic in Guyana, COVID-19 pandemic in Haiti, COVID-19 pandemic in Honduras, COVID-19 pandemic in Hungary, COVID-19 pandemic in Iceland, COVID-19 pandemic in India, COVID-19 pandemic in Indonesia, COVID-19 pandemic in Iran, COVID-19 pandemic in Iraq, COVID-19 pandemic in Israel, COVID-19 pandemic in Italy, COVID-19 pandemic in Ivory Coast, COVID-19 pandemic in Jamaica, COVID-19 pandemic in Japan, COVID-19 pandemic in Jersey, COVID-19 pandemic in Jordan, COVID-19 pandemic in Kazakhstan, COVID-19 pandemic in Kenya, COVID-19 pandemic in Kosovo, COVID-19 pandemic in Kuwait, COVID-19 pandemic in Kyrgyzstan, COVID-19 pandemic in Laos, COVID-19 pandemic in Latvia, COVID-19 pandemic in Lebanon, COVID-19 pandemic in Libya, COVID-19 pandemic in Liechtenstein, COVID-19 pandemic in Lithuania, COVID-19 pandemic in Luxembourg, COVID-19 pandemic in mainland China, COVID-19 pandemic in Malawi, COVID-19 pandemic in Malaysia, COVID-19 pandemic in Mali, COVID-19 pandemic in Malta, COVID-19 pandemic in Moldova, COVID-19 pandemic in Monaco, COVID-19 pandemic in Montenegro, COVID-19 pandemic in New Caledonia, COVID-19 pandemic in North Asia, COVID-19 pandemic in North Macedonia, COVID-19 pandemic in Northern Cyprus, COVID-19 pandemic in Northern Ireland, COVID-19 pandemic in Norway, COVID-19 pandemic in Poland, COVID-19 pandemic in Portugal, COVID-19 pandemic in Romania, COVID-19 pandemic in Russia, COVID-19 pandemic in San Marino, COVID-19 pandemic in Scotland, COVID-19 pandemic in Serbia, COVID-19 pandemic in Sevastopol, COVID-19 pandemic in Slovakia, COVID-19 pandemic in Slovenia, COVID-19 pandemic in South Ossetia, COVID-19 pandemic in Spain, COVID-19 pandemic in Sweden, COVID-19 pandemic in Switzerland, COVID-19 pandemic in the Åland Islands, COVID-19 pandemic in the Bahamas, COVID-19 pandemic in the Central African Republic, COVID-19 pandemic in the Comoros, COVID-19 pandemic in the Czech Republic, COVID-19 pandemic in the Democratic Republic of the Congo, COVID-19 pandemic in the Dominican Republic, COVID-19 pandemic in the Donetsk People's Republic, COVID-19 pandemic in the European Union, COVID-19 pandemic in the Faroe Islands, COVID-19 pandemic in the Federated States of Micronesia, COVID-19 pandemic in the Gambia, COVID-19 pandemic in the Guantanamo Bay Naval Base, COVID-19 pandemic in the Isle of Man, COVID-19 pandemic in the Kurdistan Region, COVID-19 pandemic in the Luhansk People's Republic, COVID-19 pandemic in the Netherlands, COVID-19 pandemic in the Regional Municipality of Peel, COVID-19 pandemic in the Republic of Artsakh, COVID-19 pandemic in the Republic of Ireland, COVID-19 pandemic in the Republic of the Congo, COVID-19 pandemic in the United Kingdom, COVID-19 pandemic in Transnistria, COVID-19 pandemic in Turkey, COVID-19 pandemic in Ukraine, COVID-19 pandemic in Vatican City, COVID-19 pandemic in Wales, COVID-19 pandemic in Wallis and Futuna, COVID-19 pandemic on Charles de Gaulle, COVID-19 pandemic on Diamond Princess, COVID-19 Pandemic Unemployment Payment, Creutzfeldt–Jakob disease, Critical illness insurance, Critical illness polyneuropathy, Crohn's disease, Cryptogenic organizing pneumonia, Cytomegaloviral disease, Cytomegalovirus infection, Dance and health, Deen Dayal Mobile Health Clinic, Defined contribution health benefits, Degenerative disc disease, Degenerative disease, Dejerine–Sottas disease, Dementia and Alzheimer's disease in Australia, Demyelinating disease, Dengue pandemic in Sri Lanka, Dental public health, Depression of Alzheimer disease, Desquamative interstitial pneumonia, Developmental disorder, Diagnosis of malaria, Diagnostic and Statistical Manual of Mental Disorders,

Diarrheal diseases, Digital media use and mental health, Dignity Health, Dignity Health St. Joseph's Hospital and Medical Center, Director-General of the World Health Organization, Directorate of Health, Disability and women's health, Discovery of disease-causing pathogens, Disease, Disease burden, Disease cluster, Disease Control Priorities Project, Disease diffusion mapping, Disease management (health), Disease outbreak, Disease package, Disease resistance, Disease surveillance, Disease transmission, Disease X, Disease-modifying osteoarthritis drug, Diseases, Diseases and epidemics of the 19th century, Diseases of abnormal polymerization, Diseases of affluence, Diseases of despair, Diseases of poverty, Disseminated disease, Doctor of Public Health, Dole Nutrition Institute, Drugs for Neglected Diseases Initiative, Dubai Health Authority, Dukes' disease, Dust pneumonia, E-epidemiology, Ear infection, Early-onset Alzheimer's disease, EcoHealth, Ecological health, Economic epidemiology, Economic impact of the COVID-19 pandemic in Russia, Economic impact of the COVID-19 pandemic in the Republic of Ireland, Ecosystem health, Effects of climate change on human health, EHealth Exchange, Ehrlichiosis ewingii infection, Electronic health records in the United States, EMBRACE Healthcare Reform Plan, Emerging infectious disease, Emerging Themes in Epidemiology, Endemic (epidemiology), Endogenous infection, Engineering World Health, Environmental disease, Environmental epidemiology, Environmental health, Environmental health ethics, Environmental health officer, Environmental health policy, Eosinophilic pneumonia, Ephialtes (illness), Epidemic, Epidemic curve, Epidemic Diseases Act, 1897, Epidemic Intelligence Service, Epidemic models on lattices, Epidemic polyarthritis, Epidemic typhus, Epidemics (journal), Epidemiology, Epidemiology (journal), Epidemiology and Infection, Epidemiology and Psychiatric Sciences, Epidemiology data for low-linear energy transfer radiation, Epidemiology in Country Practice, Epidemiology in Relation to Air Travel, Epidemiology of asthma, Epidemiology of attention deficit hyperactive disorder, Epidemiology of autism, Epidemiology of bed bugs, Epidemiology of binge drinking, Epidemiology of breast cancer, Epidemiology of cancer, Epidemiology of chikungunya, Epidemiology of child psychiatric disorders, Epidemiology of childhood obesity, Epidemiology of depression, Epidemiology of diabetes, Epidemiology of domestic violence, Epidemiology of HIV/AIDS, Epidemiology of malnutrition, Epidemiology of measles, Epidemiology of metabolic syndrome, Epidemiology of plague, Epidemiology of pneumonia, Epidemiology of schizophrenia, Epidemiology of syphilis, Epidemiology of typhoid fever, Epstein–Barr virus-associated lymphoproliferative diseases, Eradication of infectious diseases, Escape Fire: The Fight to Rescue American Healthcare, Essence (Electronic Surveillance System for the Early Notification of Community-based Epidemics), Establishment of the World Health Organization, EuroHealthNet, European Centre for Disease Prevention and Control, European Health Examination Survey, European Health Forum Gastein, European health management association, European Journal of Epidemiology, European Observatory on Health Systems and Policies, European Parliament Committee on the Environment, Public Health and Food Safety, European Programme for Intervention Epidemiology Training, European Prospective Investigation into Cancer and Nutrition, European Public Health Alliance, European Public Health Association, European Society for Clinical Nutrition and Metabolism, European Society for Paediatric Infectious Diseases, European Society of Clinical Microbiology and Infectious Diseases, European Union response to the COVID-19 pandemic, European Working Group for Legionella Infections, Evacuations by India related to the COVID-19 pandemic, Evaluation & the Health Professions, Evolution of Infectious Disease, Evolutionary epidemiology, Excess mortality, Face masks during the COVID-19 pandemic, Fair Share Health Care Act, Fazio–Londe disease, Febrile infection-related epilepsy syndrome, Federal aid during the COVID-19 pandemic in Canada, Federal Service for Surveillance in Healthcare, Federally Qualified Health Center, Federation of European Nutrition Societies, Federation of Health and Social Services, Feminist health centers, Field epidemiology, Fifth disease, Finnish Institute for Health and Welfare, Fire breather's pneumonia, First Nations nutrition experiments, Focus of infection, Food security during the COVID-19 pandemic, Foodborne illness, Foot-and-mouth disease, Free-market healthcare, Fungal pneumonia, Gamaleya Research Institute of Epidemiology and Microbiology, Gastrointestinal disease, Gender disparities in health, Genetic epidemiology, Genetic Epidemiology (journal), George Institute for Global Health, Germ theory of disease, Ghana Infectious Disease Centre, Ghanaian government response to the COVID-19 pandemic, GIS and public health, Global Acute Malnutrition, Global Alliance for Improved Nutrition, Global Alliance on Health and Pollution, Global Burden of Disease Study, Global Climate and Health Alliance, Global Coalition Against Pneumonia, Global Forum for Health Research, Global health, Global Health Council, Global Health Delivery Project, Global Health Innovative Technology Fund, Global Health Observatory, Global Health Security Agenda, Global Health Security Index, Global Health Security Initiative, Global Health Share Initiative, Global Infectious Disease Epidemiology Network, Global Malaria Action Plan, Global mental health, Global Network for Neglected Tropical Diseases, Global Public Health Intelligence Network, Global Research Collaboration for Infectious Disease Preparedness, Global Strategy for Women's and Children's Health, Globalization and disease, Glossary of the COVID-19 pandemic, Goal-oriented health care, Gold Coast Influenza Epidemic, Graduate School of Health Economics and Management, Gram-negative bacterial infection, Grand Challenges In Global Health, Graves' disease, Groningen epidemic, Grossman model of health demand, Group B streptococcal infection, Gulf War Health Research Reform Act of 2014, Handbook of Religion and Health, HCA Healthcare, Health, Health (Preservation and Protection and other Emergency Measures in the Public Interest) Act 2020, Health 21, Health Action International, Health administration, Health advocacy, Health Alliance International, Health and Social Protection Federation, Health and wealth, Health and welfare trust, Health belief model, Health Books International, Health Canada, Health care access among Dalits in India, Health Care Card, Health Care Compact, Health care efficiency measures, Health care finance in the United States, Health Care for Women International, Health care fraud, Health care in Argentina, Health care in

Australia, Health Care in Canada Survey, Health care in Colombia, Health care in Cyprus, Health care in France, Health care in Liberia, Health care in Mozambique, Health care in New Zealand, Health care in Poland, Health care in Saudi Arabia, Health care in Spain, Health care in Sweden, Health care in the Philippines, Health care in the United Kingdom, Health care in the United States, Health care in Turkey, Health care in Venezuela, Health care prices in the United States, Health care ratings, Health care rationing, Health care reforms proposed during the Obama administration, Health care system in Japan, Health care system of the elderly in Germany, Health care systems by country, Health care time and motion study, Health consequences of the Deepwater Horizon oil spill, Health crisis, Health Data Insight, Health departments in the United States, Health disaster, Health economics, Health Economics, Health economics (Germany), Health Economics, Policy and Law, Health education, Health Education & Behavior, Health effect, Health effects of chocolate, Health effects of phenols and polyphenols, Health effects of salt, Health effects of tea, Health effects of wine, Health Emergencies Programme (WHO), Health equity, Health fair, Health For All, Health Forecasting (UCLA), Health Foundation, Health geography, Health human resources, Health humanities, Health impact assessment, Health in All Policies, Health in Nigeria, Health in Northern Cyprus, Health informatics in China, Health information management, Health Information National Trends Survey, Health information on Wikipedia, Health insurance, Health insurance coverage in the United States, Health insurance in China, Health insurance marketplace, Health insurance premium index, Health literacy, Health maintenance organization, Health marketing, Health measures during the construction of the Panama Canal, Health Metrics Network, Health of Hillary Clinton, Health of Towns Association, Health policy, Health policy and management, Health policy in Bangladesh, Health politics, Health promotion, Health promotion in higher education, Health Promotion International, Health Promotion Practice, Health Protection Surveillance Centre, Health record trust, Health reimbursement account, Health risk assessment, Health savings account, Health Sciences Online, Health security, Health Security Express, Health services research, Health Spending Account, Health Star Rating System, Health surveillance, Health system, Health systems strengthening, Health technology, Health Threat Unit, Health Utilities Index, Health-related embarrassment, Healthcare availability for undocumented immigrants in the United States, Healthcare in Afghanistan, Healthcare in Albania, Healthcare in Algeria, Healthcare in Angola, Healthcare in Austria, Healthcare in Azerbaijan, Healthcare in Bahrain, Healthcare in Belgium, Healthcare in Brazil, Healthcare in Canada, Healthcare in Chennai, Healthcare in China, Healthcare in Croatia, Healthcare in Cuba, Healthcare in Denmark, Healthcare in Egypt, Healthcare in Estonia, Healthcare in Ethiopia, Healthcare in Finland, Healthcare in Georgia (country), Healthcare in Germany, Healthcare in Ghana, Healthcare in Greece, Healthcare in Hungary, Healthcare in Iceland, Healthcare in India, Healthcare in Indonesia, Healthcare in Iran, Healthcare in Iraq, Healthcare in Israel, Healthcare in Italy, Healthcare in Kenya, Healthcare in Kuwait, Healthcare in Luxembourg, Healthcare in Madagascar, Healthcare in Malawi, Healthcare in Malaysia, Healthcare in Malta, Healthcare in Mexico, Healthcare in Moldova, Healthcare in Nicaragua, Healthcare in Nigeria, Healthcare in Norway, Healthcare in Pakistan, Healthcare in Panama, Healthcare in Peru, Healthcare in Portugal, Healthcare in Qatar, Healthcare in Romania, Healthcare in Russia, Healthcare in Rwanda, Healthcare in Saint Helena, Healthcare in San Marino, Healthcare in Senegal, Healthcare in Serbia, Healthcare in Sierra Leone, Healthcare in Singapore, Healthcare in Slovakia, Healthcare in Slovenia, Healthcare in South Africa, Healthcare in South Korea, Healthcare in Switzerland, Healthcare in Taiwan, Healthcare in Tanzania, Healthcare in Thailand, Healthcare in the Czech Republic, Healthcare in the Isle of Man, Healthcare in the Netherlands, Healthcare in the Republic of Ireland, Healthcare in the State of Palestine, Healthcare in the United Arab Emirates, Healthcare in Tristan da Cunha, Healthcare in Uganda, Healthcare in Ukraine, Healthcare in Zambia, Healthcare number 1450, Healthcare rationing in the United States, Healthcare reform debate in the United States, Healthcare reform in China, Healthcare reform in the United States, Healthcare shortage area, Healthcare Spending Account, Healthcare transport, Healthcare UK, HealthCare Volunteer, HealthCare.gov, HealthConnect, Healthlink Worldwide, HealthMap, HealthONE Colorado, HealthRight International, Healthy city, Healthy community design, Healthy development measurement tool, Healthy Life Years, HealthyWomen, Heat illness, Hereditary CNS demyelinating disease, High-deductible health plan, Hispanic Health Council, History of health care reform in the United States, History of malaria, History of mental disorders, History of Tay–Sachs disease, History of USDA nutrition guides, Holozoic nutrition, Home health care software, Homosexuality as a disease, Hookworm infection, Hospital-acquired infection, Hospital-acquired pneumonia, Hospital, patients, health, territories, How to Have Sex in an Epidemic, Human genetic resistance to malaria, Human Heredity and Health in Africa, Human nutrition, Human papillomavirus infection, Human rights issues related to the COVID-19 pandemic, Hypertensive disease of pregnancy, Idiopathic disease, Idiopathic interstitial pneumonia, Idiopathic multicentric Castleman disease, Idiopathic pneumonia syndrome, IgG4-related disease, Illinois Health Benefits Exchange, Illness, Imagine No Malaria, Immigrant health care in the United States, Impact of the COVID-19 pandemic on education in Ghana, Impact of the COVID-19 pandemic on education in the Republic of Ireland, Impact of the COVID-19 pandemic on education in the United Kingdom, Impact of the COVID-19 pandemic on Gaelic games, Impact of the COVID-19 pandemic on human rights in Argentina, Indian government response to the COVID-19 pandemic, Indian migrant workers during the COVID-19 pandemic, Indian state government responses to the COVID-19 pandemic, Indigenous health in Australia, Indoor air pollution in developing nations, Inequality in disease, Infant mortality, Infant nutrition, Infection, Infection Control Society of Pakistan, Infection prevention and control, Infection rate, Infections associated with diseases, Infectious disease, Infectious disease (medical specialty), Infectious Disease (Notification) Act 1889, Infectious Disease Pharmacokinetics Laboratory, Infectious Diseases Institute, Infectious Diseases Society of America, Inflammatory bowel disease, Inflammatory demyelinating diseases of the central

nervous system, Influenza pandemic, Institute for Health Metrics and Evaluation, Institute of Health and Social Care Management, Institute of Public Health (Bangladesh), Integrated Disease Surveillance Programme, Integrated Management of Childhood Illness, Intentional contagion of infection, Interdisciplinary Association for Population Health Science, Intermountain Healthcare, International Association of National Public Health Institutes, International Centre for Migration and Health, International Classification of Diseases, International Classification of Functioning, Disability and Health, International Conference on Emerging Infectious Diseases, International health, International Health Partnership, International Health Regulations, International Journal of Epidemiology, International Journal of Men's Health, International Lyme and Associated Diseases Society, International Men's Health Week, International Network of Health Promoting Hospitals and Health Services, International Partnership on Avian and Pandemic Influenza, International reactions to the COVID-19 pandemic in Italy, International Society for Environmental Epidemiology, International Society for Infectious Diseases in Obstetrics and Gynaecology, International Society for Pharmacoepidemiology, International Union of Air Pollution Prevention and Environmental Protection Associations, International Volcanic Health Hazard Network, Intestinal infectious diseases, Intradialytic parenteral nutrition, Irish Nutrition and Dietetic Institute, Iron Triangle of Health Care, Isolation (health care), ITU-WHO Focus Group on Artificial Intelligence for Health, Ivey International Centre for Health Innovation, James C. Robinson (health economist), James Thornton (health economist), Jembrana disease, Jennifer McMahon (nutritionist), Johns Hopkins Center for Health Security, Joondalup Family Health Study, Journal of Clinical Epidemiology, Journal of Epidemiology, Journal of Epidemiology and Biostatistics, Journal of Epidemiology and Community Health, Journal of Exposure Science and Environmental Epidemiology, Journal of Health Economics, Journal of Urban Health, Jurosomatic illness, Kashin–Beck disease, Kawasaki disease, Kettering Health Network, Kids for World Health, Kivu Ebola epidemic, Korea Disease Control and Prevention Agency, Krabbe disease, Kuru (disease), Kyasanur Forest disease, Laboratory-acquired infection, Landscape epidemiology, Latent period (epidemiology), Legacy Health, Legionnaires' disease, Leveraging Agriculture for Improving Nutrition and Health, Lhermitte–Duclos disease, Lipid pneumonia, List of AdventHealth hospitals, List of autoimmune diseases, List of countries by health insurance coverage, List of countries by total health expenditure per capita, List of diseases eliminated from the United States, List of epidemics, List of foodborne illness outbreaks, List of foodborne illness outbreaks by death toll, List of foodborne illness outbreaks in the United States, List of ICD-9 codes 290–319: mental disorders, List of infections of the central nervous system, List of infectious diseases, List of infectious diseases causing flu-like syndrome, List of insect-borne diseases, List of Legionnaires' disease outbreaks, List of mental disorders, List of mental disorders in film, List of national public health agencies, List of nutrition guides, List of pneumonia deaths, List of sexually transmitted infections by prevalence, List of types of malnutrition, Liverpool Neurological Infectious Diseases Course, Lobar pneumonia, Localized disease, London Declaration on Neglected Tropical Diseases, Lower respiratory tract infection, Lung disease, Lutheran Health Network, Lyme disease, Lyme disease microbiology, Lymphocytic interstitial pneumonia, Lysosomal storage disease, Madras motor neuron disease, Malaria, Malaria and the Caribbean, Malaria antigen detection tests, Malaria Atlas Project, Malaria Consortium, Malaria Control Project, Malaria culture, Malaria Day in the Americas, Malaria Eradication Scientific Alliance, Malaria in Benin, Malaria in Madagascar, Malaria Journal, Malaria No More, Malaria No More UK, Malaria Policy Advisory Committee, Malaria prophylaxis, Malaria vaccine, Malarial nephropathy, MalariaWorld, Malnutrition, Malnutrition in India, Malnutrition in Kerala, Malnutrition in Peru, Malnutrition in South Africa, Malnutrition in Zimbabwe, Malnutrition–inflammation complex, Management of Crohn's disease, Managerial epidemiology, Marburg virus disease, Mass psychogenic illness, Massachusetts smallpox epidemic, Maternal health, Maternal health in Angola, Maternal health in Rwanda, Maternal Health Task Force, Maternal mortality, Maternal mortality ratio, Mayaro virus disease, Measles, Measles resurgence in the United States, Measles vaccine, Media coverage of the COVID-19 pandemic, Medical and Health Workers' Union of Nigeria, Medical officer of health, Medical students' disease, Medicines for Malaria Venture, Mekong Basin Disease Surveillance, Men's health, Men's Health, Men's health in Australia, Meningococcal disease, Mental disorder, Mental disorders and gender, Mental health, Mental health during the COVID-19 pandemic, Mental health law, Mental health literacy, Mental health triage, Mental illness, Mental illness portrayed in media, Michael Colgan (nutritionist), Micronutrient malnutrition, Mineral Nutrition of Plants: Principles and Perspectives, Minister of State for Health (UK), Minister of State for Mental Health, Suicide Prevention and Patient Safety, Ministry of Health (Panama), Ministry of Health (Spain), Ministry of Health and Welfare (South Korea), Ministry of Health Promotion and Sport (Ontario), Mission Health System, Mississippi Health Project, Mitochondrial disease, Mixed connective tissue disease, Mobile source air pollution, Modern Healthcare, Moldovan–Romanian collaboration during the COVID-19 pandemic, Molecular epidemiology, Morbidity and Mortality Weekly Report, Morris Heights Health Center, Mosquito-borne disease, Mosquito-malaria theory, Motor neuron disease, Motor Neurone Disease Association, Mount Sinai Health System, MRC Human Nutrition Research, Muesli belt malnutrition, Multifactorial diseases, Multimorbidity, Multiple complex developmental disorder, Music therapy for Alzheimer's disease, My Health Record, Mycobacterium avium-intracellulare infection, Mycoplasma hominis infection, Mycoplasma pneumonia, Mycoplasma pneumoniae infection, National Advisory Committee on SARS and Public Health, National Air Pollution Symposium, National Association for Public Health Policy, National Association of County and City Health Officials, National Center for Disease Control and Public Health (Georgia), National Centre for Disease Control, National Centre for Infectious Diseases, National Children's Center for Rural and Agricultural Health and Safety, National Comorbidity Survey, National Emerging Infectious Diseases Laboratories, National

Federation of Local Authority and Healthcare Workers, National Foundation for Infectious Diseases, National Fund for Health Insurance, National Health Accounts, National Health Act 1953, National Health and Nutrition Examination Survey, National health insurance, National Health Insurance (Japan), National Health Interview Survey, National Health Mission, National Health Policy, National Infection Service, National Institute for Communicable Diseases, National Institute for Health and Care Excellence, National Institute for Health and Disability Insurance, National Institute for Health Protection, National Institute for Public Health and the Environment, National Institute of Health, Islamabad, National Institute of Malaria Research, National Institute of Nutrition, Hyderabad, National Institute of Public Health of Japan, National Malaria Eradication Program, National Perinatal Epidemiology Unit, National Prostate Health Month, National Public Health Emergency Team (2020), National public health institutes, National Public Health Organization (Greece), National School of Public Health (Spain), Native American disease and epidemics, Native American Women's Health Education Resource Center, Navicent Health Baldwin, Necrotizing pneumonia, Neglected tropical disease research and development, Neglected tropical diseases, Neglected tropical diseases in India, Neonatal infection, Network for Capacity Development in Nutrition, Neurodevelopmental disorder, Neuroepidemiology (journal), Nevada Health Link, New Mexico Health Insurance Exchange, NewYork-Presbyterian Healthcare System, Nigel Edwards (health), Nigeria Centre for Disease Control, NINCDS-ADRDA Alzheimer's Criteria, Nipah virus infection, Noma (disease), Non-alcoholic fatty liver disease, Non-communicable disease, Non-communicable diseases, Non-pharmaceutical intervention (epidemiology), Non-specific interstitial pneumonia, Northwell Health, Norwegian Association of Health and Social Care Personnel, Norwegian Institute of Public Health, Notifiable disease, Notifiable diseases in Sweden, Notifiable diseases in Switzerland, Notifiable diseases in the United Kingdom, Novant Health, Novant Health Forsyth Medical Center, Nurses' Health Study, Nutrition, Nutrition analysis, Nutrition and cognition, Nutrition and Education International, Nutrition Foundation of the Philippines, Nutrition transition, Nutritional epidemiology, Nutritional genomics, Nutritional science, NutritionDay, Nutritionist, Occult pneumonia, Occupational exposure to Lyme disease, Occupational safety and health, Office on Women's Health, One Health, Opportunistic infection, Oregon Medicaid health experiment, OSF HealthCare, Ōtaki Health Camp, Ottawa Charter for Health Promotion, Outline of air pollution dispersion, Overnutrition, Overseas Student Health Cover, Overwhelming post-splenectomy infection, Oxford Brookes Centre for Nutrition and Health, Pacheco's disease, Pacific Society for Reproductive Health Trust, Paediatric and Perinatal Epidemiology, Paget's disease of bone, Pan American Health Organization, Pandemic, Pandemic H1N1/09 virus, Pandemic Influenza Preparedness Framework, Pandemic predictions and preparations prior to the COVID-19 pandemic, Pandemic Preparedness and Response Act, Pandemic prevention, Pandemic Severity Assessment Framework, Pandemic severity index, Pandemics, Papaya Bunchy Top Disease, Parasitic disease, Parasitic pneumonia, Parkinson's disease, Parliamentary Under-Secretary of State for Prevention, Public Health and Primary Care, Partnership for Maternal, Newborn & Child Health, Pathogens and Global Health, Patient Health Questionnaire, Pay for performance (healthcare), Pelvic inflammatory disease, Personally Controlled Electronic Health Record, Pervasive developmental disorder, Pervasive developmental disorder not otherwise specified, Peyronie's disease, Pinta (disease), Pinworm infection, Plague (disease), Plague City: SARS in Toronto, Plague epidemics in Malta, Plant nutrition, Pneumococcal infection, Pneumococcal pneumonia, Pneumocystis pneumonia, Pneumonia, Pneumonia (non-human), Pneumonia jacket, Pneumonia of unknown etiology (PUE) surveillance system, Pneumonia severity index, Pogosta disease, Political impact of the COVID-19 pandemic in Russia, Population health, Population Health Forum, Population health policies and interventions, Population, health, and the environment, Portal:Pandemics, Postorgasmic illness syndrome, Pravastatin or atorvastatin evaluation and infection therapy - thrombolysis in myocardial infarction 22, Prebiotic (nutrition), Pregnancy-associated malaria, President's Malaria Initiative, Prevalence of mental disorders, Prevention of Tay–Sachs disease, Price-Pottenger Nutrition Foundation, Primary Health Centre (India), Prime Healthcare Services, Priority-setting in global health, Private health services plan, Program for Jewish Genetic Health, Progressive disease, Protein–energy malnutrition, Providence Health & Services, Providence St. Joseph Health, Psychiatric epidemiology, Psychogenic disease, Public health, Public Health Agency of Canada, Public Health Agency of Sweden, Public Health Emergency of International Concern, Public Health England, Public health genomics, Public health informatics, Public health insurance option, Public health intervention, Public health law, Public health nursing, Public health observatory, Public health problems in the Aral Sea region, Public Health Scotland, Public health surveillance, Public health system in India, Public Health Wales, Publicly funded health care, Pullorum disease, Qapqal disease, Race and health, RAND Health Insurance Experiment, Rare disease, Real-time outbreak and disease surveillance, Refugee health care in Canada, Regional Forum on Environment and Health in Southeast and East Asian Countries, Regional Health Agency, Reproductive health, Reproductive health care for incarcerated women in the United States, Reproductive system disease, Respiratory diseases, Respiratory Syncytial Infection, Respiratory tract infection, Rheumatoid disease of the spine, Royal Commission on the Future of Health Care in Canada, Rural health care in Australia, Russian government responses to the COVID-19 pandemic, Salt and cardiovascular disease, Samaritan Health Services, SARS conspiracy theory, Scandinavian Journal of Work, Environment & Health, School health and nutrition services, School health education, School-based health centers, Science diplomacy and pandemics, Second plague pandemic, Self-rated health, Sentara Healthcare, Serratia infection, Services for mental disorders, Sexual and Reproductive Health Matters, Sexual health clinic, Sexually transmitted infection, Shona Holmes health care incident, Sickle cell disease, Single-payer healthcare, Sissel v. United States Department of Health & Human Services, Skin and skin structure infection, Skin infection, Smallpox epidemic, Social determinants of health, Social impact of

the COVID-19 pandemic in Russia, Social Psychiatry and Psychiatric Epidemiology, Societal and cultural aspects of Tay–Sachs disease, Society for Family Health Nigeria, Society of Infectious Diseases Pharmacists, South African Malaria Initiative, South Texas Center for Emerging Infectious Diseases, Southern tick-associated rash illness, Spanish National Health System, Spatial and Spatio-temporal Epidemiology, Spatial epidemiology, Specific replant disease, St. Patrick Hospital and Health Sciences Center, St. Vincent's Health System, Stateville Penitentiary Malaria Study, Statistics of the COVID-19 pandemic in Argentina, Statistics of the COVID-19 pandemic in Australia, Statistics of the COVID-19 pandemic in Brazil, Statistics of the COVID-19 pandemic in Germany, Statistics of the COVID-19 pandemic in India, Statistics of the COVID-19 pandemic in Indonesia, Statistics of the COVID-19 pandemic in Italy, Statistics of the COVID-19 pandemic in Japan, Statistics of the COVID-19 pandemic in Malaysia, Statistics of the COVID-19 pandemic in Poland, Statistics of the COVID-19 pandemic in Portugal, Statistics of the COVID-19 pandemic in Russia, Statistics of the COVID-19 pandemic in Scotland, Statistics of the COVID-19 pandemic in Tamil Nadu, Statistics of the COVID-19 pandemic in the United Kingdom, STOP Foodborne Illness, Strengthening the reporting of observational studies in epidemiology, Streptococcus pneumoniae, Study of Health in Pomerania, Study on Global Ageing and Adult Health, Suicide epidemic, Superinfection, Susan Parkinson (nutritionist), Susceptibility and severity of infections in pregnancy, Sutter Health, Sweating sickness epidemics, Swedish Association of Health Professionals, Swedish government response to the COVID-19 pandemic, Systemic disease, Taiwan Centers for Disease Control, Target Malaria, Targeting (international health), Tay–Sachs disease, Template:Ascension Health, Template:Autonomic diseases, Template:Central nervous system disease, Template:Cerebrovascular diseases, Template:COVID-19 pandemic data/Bangladesh medical cases by division, Template:COVID-19 pandemic in the Republic of Ireland, Template:Cranial nerve disease, Template:Demyelinating diseases of CNS, Template:Diseases of meninges, Template:Diseases of myoneural junction and muscle, Template:Epidemic-stub, Template:Eradication of infectious disease, Template:Gram-positive actinobacteria diseases, Template:Infectious-disease-stub, Template:Malaria, Template:Nervoussystem-disease-stub, Template:Peripheral nervous system disease, Template:Pervasive developmental disorders, Template:Plant nutrition, Template:Public health, Template:Tick-borne diseases and infestations, Template:Vertically transmitted infection, Template:Women's health, Tenet Healthcare, Texas Health Huguley Hospital Fort Worth South, The European Journal of Health Economics, The Far West Baby Health Clinic Cars, The Global Fund to Fight AIDS, Tuberculosis and Malaria, The Journal of Mental Health Policy and Economics, The Medical Center, Navicent Health, The Office of Health Economics, The Rockefeller Foundation Economic Council on Planetary Health, The Trøndelag Health Study, Theiler's disease, Third plague pandemic, Tick-borne disease, Tick-Borne Disease Alliance, Timeline of global health, Timeline of peptic ulcer disease and Helicobacter pylori, Timeline of the COVID-19 pandemic in Afghanistan, Timeline of the COVID-19 pandemic in Argentina, Timeline of the COVID-19 pandemic in Australia, Timeline of the COVID-19 pandemic in Bangladesh, Timeline of the COVID-19 pandemic in Belarus, Timeline of the COVID-19 pandemic in Brazil, Timeline of the COVID-19 pandemic in Canada, Timeline of the COVID-19 pandemic in Croatia, Timeline of the COVID-19 pandemic in Ghana, Timeline of the COVID-19 pandemic in India, Timeline of the COVID-19 pandemic in India (January–May 2020), Timeline of the COVID-19 pandemic in India (June–December 2020), Timeline of the COVID-19 pandemic in Indonesia, Timeline of the COVID-19 pandemic in Italy, Timeline of the COVID-19 pandemic in Japan, Timeline of the COVID-19 pandemic in Malaysia, Timeline of the COVID-19 pandemic in Mexico, Timeline of the COVID-19 pandemic in Nepal, Timeline of the COVID-19 pandemic in New Zealand, Timeline of the COVID-19 pandemic in Pakistan, Timeline of the COVID-19 pandemic in Romania, Timeline of the COVID-19 pandemic in Russia, Timeline of the COVID-19 pandemic in Singapore, Timeline of the COVID-19 pandemic in Spain, Timeline of the COVID-19 pandemic in Sweden, Timeline of the COVID-19 pandemic in Thailand, Timeline of the COVID-19 pandemic in the Philippines, Timeline of the COVID-19 pandemic in the Republic of Ireland, Timeline of the COVID-19 pandemic in the Republic of Ireland (2021), Timeline of the COVID-19 pandemic in the Republic of Ireland (January–June 2020), Timeline of the COVID-19 pandemic in the Republic of Ireland (July–December 2020), Timeline of the COVID-19 pandemic in the United Kingdom, Timeline of the COVID-19 pandemic in the United States, Timeline of the COVID-19 pandemic in Turkey, Timeline of the COVID-19 pandemic in Uruguay, Timeline of the COVID-19 pandemic in Vietnam, Timeline of the COVID-19 pandemic Nigeria, Tool for Influenza Pandemic Risk Assessment, Top dying disease, Transportation and health, Tropical disease, Two-tier healthcare, Typhus epidemic in Goose Village, Montreal, UCLA Health, UCSC Malaria Genome Browser, UK Health Alliance on Climate Change, Undernutrition, Undernutrition in children, Uni Health, Unicentric Castleman disease, Union of Healthcare, United Kingdom health law, UnityPoint Health, UnityPoint Health - Allen Hospital, Universal Declaration on the Eradication of Hunger and Malnutrition, University of Edinburgh School of Health in Social Science, Ureaplasma urealyticum infection, Usual interstitial pneumonia, Vaccine-preventable diseases, Value-based health care, Vanguard Health Systems, Vapours (disease), Variant Creutzfeldt–Jakob disease, Vector (epidemiology), Vector-borne disease, Venereal Disease Research Laboratory test, Ventilator-associated pneumonia, Vermont health care reform, Vertically transmitted infection, Very early onset inflammatory bowel disease, Veteran Health Identification Card, Veterans Health Administration, Victorian Health Promotion Foundation, Viral disease testing, Viral pneumonia, Virgin soil epidemic, Vogt–Koyanagi–Harada disease, Waterborne disease and climate change, Waterborne diseases, Weather and climate effects on Lyme disease exposure, Western African Ebola virus epidemic, Whipple's disease, Wilt disease, Women Health and Action Research Centre, Women's health, Women's health movement in the United States, Working Environment (Air Pollution, Noise and Vibration) Convention, 1977, Workplace health promotion, World Chagas Disease Day, World Health Assembly, World Health Organization, World Health

Organization Composite International Diagnostic Interview, World Malaria Day, World Pneumonia Day, Your Health Idaho, Zoonotic disease

List of English Wikipedia articles used to track public engagement in the COVID-19 pandemic

Boroughs of Montreal during the COVID-19 pandemic, British government response to the COVID-19 pandemic, Chloroquine and hydroxychloroquine during the COVID-19 pandemic, Coronavirus disease 2019, Coronavirus diseases, COVID-19 pandemic, COVID-19 pandemic by country and territory, COVID-19 pandemic cases, COVID-19 pandemic death rates by country, COVID-19 pandemic deaths, COVID-19 pandemic in Abkhazia, COVID-19 pandemic in Afghanistan, COVID-19 pandemic in Akrotiri and Dhekelia, COVID-19 pandemic in Albania, COVID-19 pandemic in Algeria, COVID-19 pandemic in American Samoa, COVID-19 pandemic in Andorra, COVID-19 pandemic in Angola, COVID-19 pandemic in Antigua and Barbuda, COVID-19 pandemic in Argentina, COVID-19 pandemic in Armenia, COVID-19 pandemic in Australia, COVID-19 pandemic in Austria, COVID-19 pandemic in Azerbaijan, COVID-19 pandemic in Bahrain, COVID-19 pandemic in Bangladesh, COVID-19 pandemic in Barbados, COVID-19 pandemic in Belarus, COVID-19 pandemic in Belgium, COVID-19 pandemic in Belize, COVID-19 pandemic in Benin, COVID-19 pandemic in Bhutan, COVID-19 pandemic in Bolivia, COVID-19 pandemic in Bosnia and Herzegovina, COVID-19 pandemic in Botswana, COVID-19 pandemic in Brazil, COVID-19 pandemic in Brunei, COVID-19 pandemic in Bulgaria, COVID-19 pandemic in Burkina Faso, COVID-19 pandemic in Burundi, COVID-19 pandemic in Cambodia, COVID-19 pandemic in Cameroon, COVID-19 pandemic in Canada, COVID-19 pandemic in Cape Verde, COVID-19 pandemic in Chad, COVID-19 pandemic in Chile, COVID-19 pandemic in Colombia, COVID-19 pandemic in Costa Rica, COVID-19 pandemic in Crimea, COVID-19 pandemic in Croatia, COVID-19 pandemic in Cuba, COVID-19 pandemic in Cyprus, COVID-19 pandemic in Denmark, COVID-19 pandemic in Djibouti, COVID-19 pandemic in Dominica, COVID-19 pandemic in East Timor, COVID-19 pandemic in Easter Island, COVID-19 pandemic in Ecuador, COVID-19 pandemic in Egypt, COVID-19 pandemic in El Salvador, COVID-19 pandemic in England, COVID-19 pandemic in Equatorial Guinea, COVID-19 pandemic in Eritrea, COVID-19 pandemic in Estonia, COVID-19 pandemic in Eswatini, COVID-19 pandemic in Ethiopia, COVID-19 pandemic in Europe, COVID-19 pandemic in Fiji, COVID-19 pandemic in Finland, COVID-19 pandemic in France, COVID-19 pandemic in French Polynesia, COVID-19 pandemic in Gabon, COVID-19 pandemic in Gagauzia, COVID-19 pandemic in Georgia (country), COVID-19 pandemic in Germany, COVID-19 pandemic in Ghana, COVID-19 pandemic in Gibraltar, COVID-19 pandemic in Greece, COVID-19 pandemic in Greenland, COVID-19 pandemic in Grenada, COVID-19 pandemic in Guatemala, COVID-19 pandemic in Guernsey, COVID-19 pandemic in Guinea, COVID-19 pandemic in Guinea-Bissau, COVID-19 pandemic in Guyana, COVID-19 pandemic in Haiti, COVID-19 pandemic in Honduras, COVID-19 pandemic in Hungary, COVID-19 pandemic in Iceland, COVID-19 pandemic in India, COVID-19 pandemic in Indonesia, COVID-19 pandemic in Iran, COVID-19 pandemic in Iraq, COVID-19 pandemic in Israel, COVID-19 pandemic in Italy, COVID-19 pandemic in Ivory Coast, COVID-19 pandemic in Jamaica, COVID-19 pandemic in Japan, COVID-19 pandemic in Jersey, COVID-19 pandemic in Jordan, COVID-19 pandemic in Kazakhstan, COVID-19 pandemic in Kenya, COVID-19 pandemic in Kosovo, COVID-19 pandemic in Kuwait, COVID-19 pandemic in Kyrgyzstan, COVID-19 pandemic in Laos, COVID-19 pandemic in Latvia, COVID-19 pandemic in Lebanon, COVID-19 pandemic in Libya, COVID-19 pandemic in Liechtenstein, COVID-19 pandemic in Lithuania, COVID-19 pandemic in Luxembourg, COVID-19 pandemic in mainland China, COVID-19 pandemic in Malawi, COVID-19 pandemic in Malaysia, COVID-19 pandemic in Mali, COVID-19 pandemic in Malta, COVID-19 pandemic in Moldova, COVID-19 pandemic in Monaco, COVID-19 pandemic in Montenegro, COVID-19 pandemic in New Caledonia, COVID-19 pandemic in North Asia, COVID-19 pandemic in North Macedonia, COVID-19 pandemic in Northern Cyprus, COVID-19 pandemic in Northern Ireland, COVID-19 pandemic in Norway, COVID-19 pandemic in Poland, COVID-19 pandemic in Portugal, COVID-19 pandemic in Romania, COVID-19 pandemic in Russia, COVID-19 pandemic in San Marino, COVID-19 pandemic in Scotland, COVID-19 pandemic in Serbia, COVID-19 pandemic in Sevastopol, COVID-19 pandemic in Slovakia, COVID-19 pandemic in Slovenia, COVID-19 pandemic in South Ossetia, COVID-19 pandemic in Spain, COVID-19 pandemic in Sweden, COVID-19 pandemic in Switzerland, COVID-19 pandemic in the Åland Islands, COVID-19 pandemic in the Bahamas, COVID-19 pandemic in the Central African Republic, COVID-19 pandemic in the Comoros, COVID-19 pandemic in the Czech Republic, COVID-19 pandemic in the Democratic Republic of the Congo, COVID-19 pandemic in the Dominican Republic, COVID-19 pandemic in the Donetsk People's Republic, COVID-19 pandemic in the European Union, COVID-19 pandemic in the Faroe Islands, COVID-19 pandemic in the Federated States of Micronesia, COVID-19 pandemic in the Gambia, COVID-19 pandemic in the Guantanamo Bay Naval Base, COVID-19 pandemic in the Isle of Man, COVID-19 pandemic in the Kurdistan Region, COVID-19 pandemic in the Luhansk People's Republic, COVID-19 pandemic in the Netherlands, COVID-19 pandemic in the Regional Municipality of Peel, COVID-19 pandemic in the Republic of Artsakh, COVID-19 pandemic in the Republic of Ireland, COVID-19 pandemic in the Republic of the Congo, COVID-19 pandemic in the United Kingdom, COVID-19 pandemic in Transnistria, COVID-19 pandemic in Turkey, COVID-19 pandemic in Ukraine, COVID-19 pandemic in Vatican City, COVID-19 pandemic in Wales, COVID-19 pandemic in Wallis and Futuna, COVID-19 pandemic on Charles de Gaulle, COVID-19 pandemic on Diamond Princess, COVID-19 Pandemic Unemployment Payment, Economic impact of the COVID-19 pandemic in Russia, Economic impact of the COVID-19 pandemic in the Republic of Ireland, European Union response to the COVID-19 pandemic, Evacuations by India related to the COVID-

19 pandemic, Face masks during the COVID-19 pandemic, Federal aid during the COVID-19 pandemic in Canada, Food security during the COVID-19 pandemic, Ghanaian government response to the COVID-19 pandemic, Glossary of the COVID-19 pandemic, Human rights issues related to the COVID-19 pandemic, Impact of the COVID-19 pandemic on education in Ghana, Impact of the COVID-19 pandemic on education in the Republic of Ireland, Impact of the COVID-19 pandemic on education in the United Kingdom, Impact of the COVID-19 pandemic on Gaelic games, Impact of the COVID-19 pandemic on human rights in Argentina, Indian government response to the COVID-19 pandemic, Indian migrant workers during the COVID-19 pandemic, Indian state government responses to the COVID-19 pandemic, International reactions to the COVID-19 pandemic in Italy, Media coverage of the COVID-19 pandemic, Mental health during the COVID-19 pandemic, Middle East respiratory syndrome–related coronavirus, Moldovan–Romanian collaboration during the COVID-19 pandemic, Pandemic predictions and preparations prior to the COVID-19 pandemic, Political impact of the COVID-19 pandemic in Russia, Russian government responses to the COVID-19 pandemic, Severe acute respiratory syndrome coronavirus, Severe acute respiratory syndrome coronavirus 2, Social impact of the COVID-19 pandemic in Russia, Statistics of the COVID-19 pandemic in Argentina, Statistics of the COVID-19 pandemic in Australia, Statistics of the COVID-19 pandemic in Brazil, Statistics of the COVID-19 pandemic in Germany, Statistics of the COVID-19 pandemic in India, Statistics of the COVID-19 pandemic in Indonesia, Statistics of the COVID-19 pandemic in Italy, Statistics of the COVID-19 pandemic in Japan, Statistics of the COVID-19 pandemic in Malaysia, Statistics of the COVID-19 pandemic in Poland, Statistics of the COVID-19 pandemic in Portugal, Statistics of the COVID-19 pandemic in Russia, Statistics of the COVID-19 pandemic in Scotland, Statistics of the COVID-19 pandemic in Tamil Nadu, Statistics of the COVID-19 pandemic in the United Kingdom, Swedish government response to the COVID-19 pandemic, Template:COVID-19 pandemic data/Bangladesh medical cases by division, Template:COVID-19 pandemic in the Republic of Ireland, Timeline of the COVID-19 pandemic in Afghanistan, Timeline of the COVID-19 pandemic in Argentina, Timeline of the COVID-19 pandemic in Australia, Timeline of the COVID-19 pandemic in Bangladesh, Timeline of the COVID-19 pandemic in Belarus, Timeline of the COVID-19 pandemic in Brazil, Timeline of the COVID-19 pandemic in Canada, Timeline of the COVID-19 pandemic in Croatia, Timeline of the COVID-19 pandemic in Ghana, Timeline of the COVID-19 pandemic in India, Timeline of the COVID-19 pandemic in India (January–May 2020), Timeline of the COVID-19 pandemic in India (June–December 2020), Timeline of the COVID-19 pandemic in Indonesia, Timeline of the COVID-19 pandemic in Italy, Timeline of the COVID-19 pandemic in Japan, Timeline of the COVID-19 pandemic in Malaysia, Timeline of the COVID-19 pandemic in Mexico, Timeline of the COVID-19 pandemic in Nepal, Timeline of the COVID-19 pandemic in New Zealand, Timeline of the COVID-19 pandemic in Pakistan, Timeline of the COVID-19 pandemic in Romania, Timeline of the COVID-19 pandemic in Russia, Timeline of the COVID-19 pandemic in Singapore, Timeline of the COVID-19 pandemic in Spain, Timeline of the COVID-19 pandemic in Sweden, Timeline of the COVID-19 pandemic in Thailand, Timeline of the COVID-19 pandemic in the Philippines, Timeline of the COVID-19 pandemic in the Republic of Ireland, Timeline of the COVID-19 pandemic in the Republic of Ireland (2021), Timeline of the COVID-19 pandemic in the Republic of Ireland (January–June 2020), Timeline of the COVID-19 pandemic in the Republic of Ireland (July–December 2020), Timeline of the COVID-19 pandemic in the United Kingdom, Timeline of the COVID-19 pandemic in the United States, Timeline of the COVID-19 pandemic in Turkey, Timeline of the COVID-19 pandemic in Uruguay, Timeline of the COVID-19 pandemic in Vietnam, Timeline of the COVID-19 pandemic Nigeria, UK Coronavirus Cancer Monitoring Project

Additional analysis

Complementing the analysis presented in the 2021 Lancet Countdown report, the Figures below provide additional evidence on dynamics in pageviews and co-click networks.

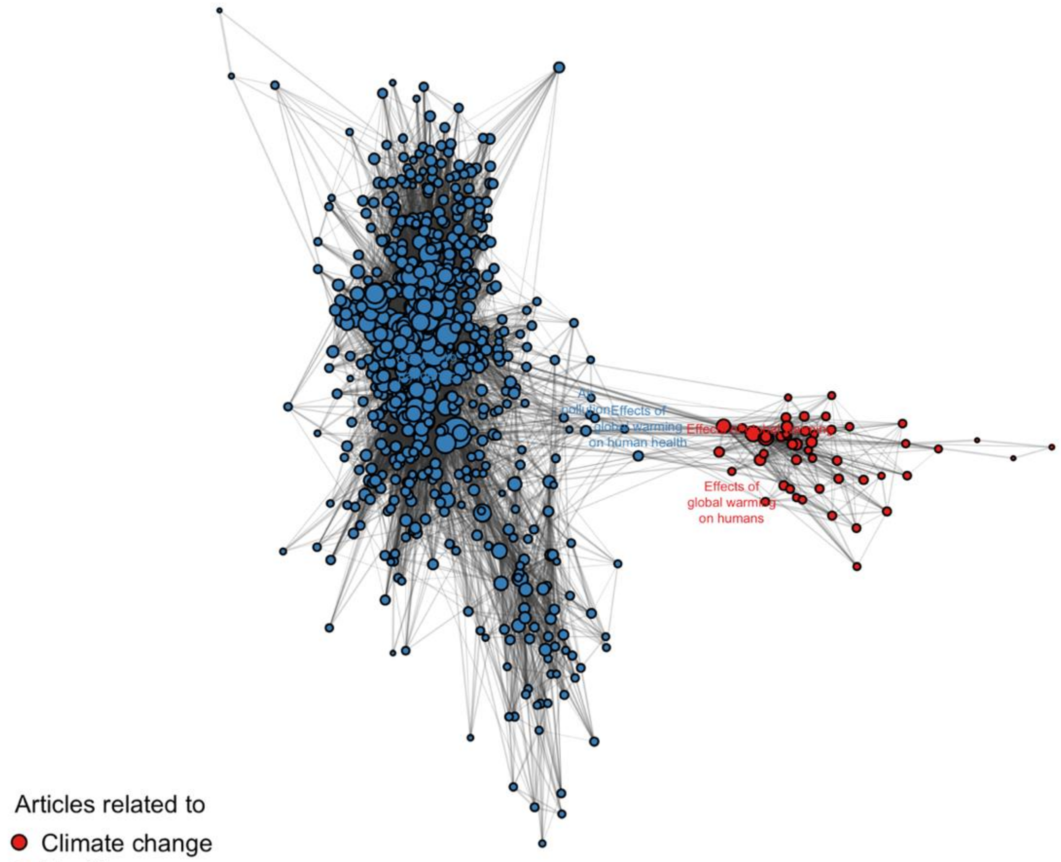

Figure 97. Connectivity graph of Wikipedia articles on climate change (red) and health (blue). Popularity of articles displayed by node size. Edges represent co-visits in the 2020 clickstream data.

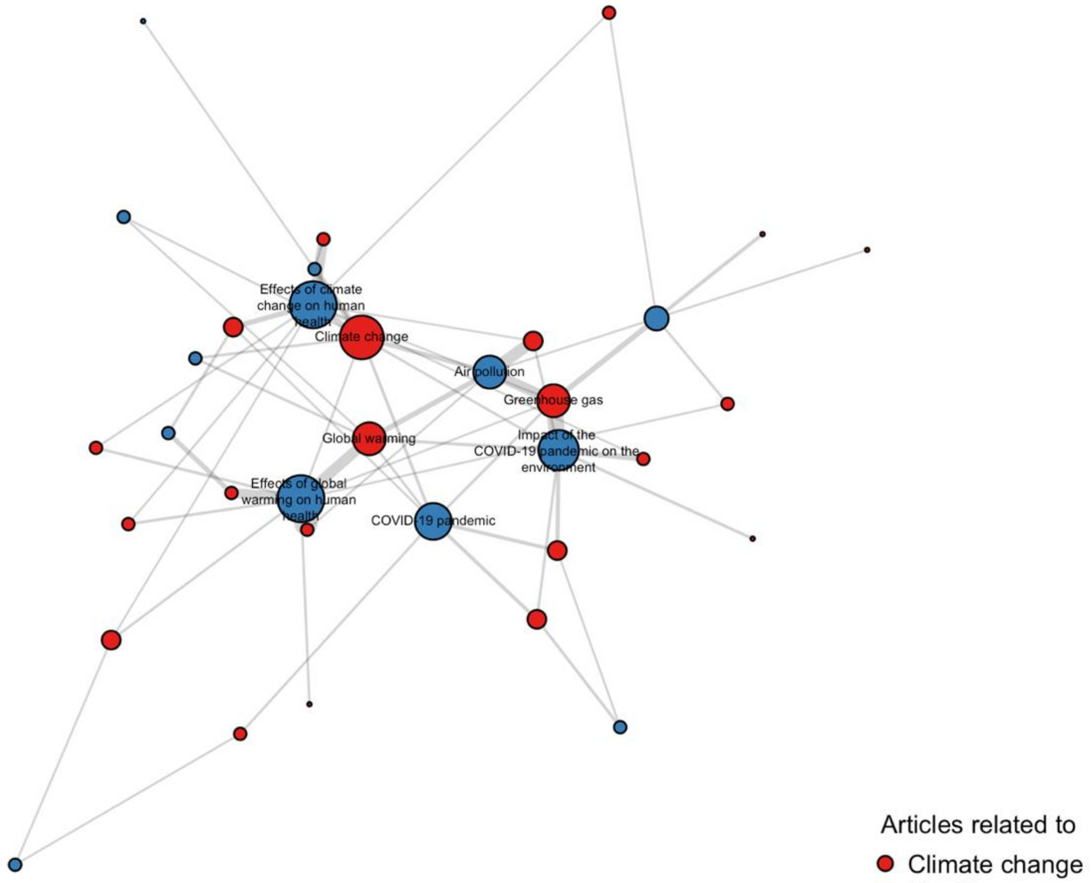

Figure 98. Connectivity graph of Wikipedia articles on climate change (red) and health (blue), filtered to co-click activity between the two domains. Popularity of articles displayed by node size. Edges represent co-visits in the 2020 clickstream data.

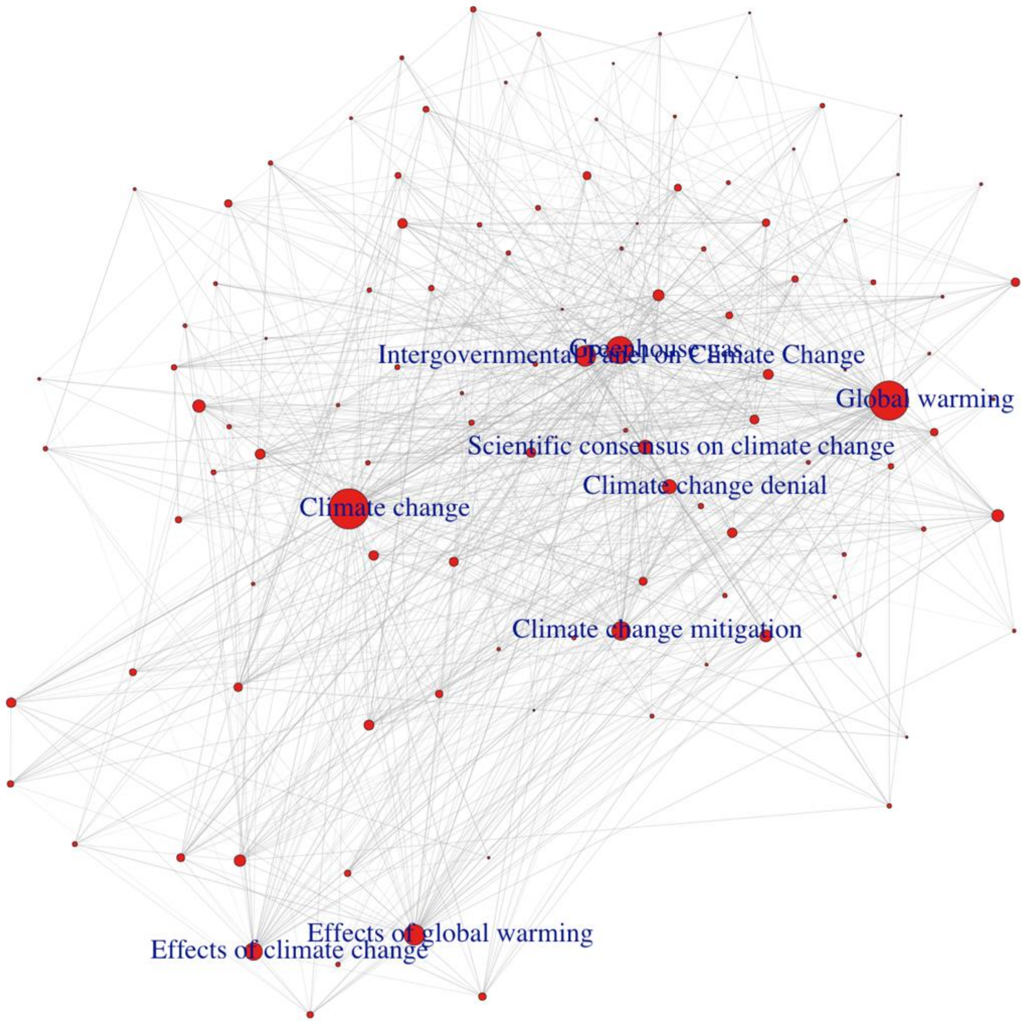

Figure 99. Connectivity graph of Wikipedia articles on climate change. Popularity of articles displayed by node size. Edges represent co-visits in the 2020 clickstream data.

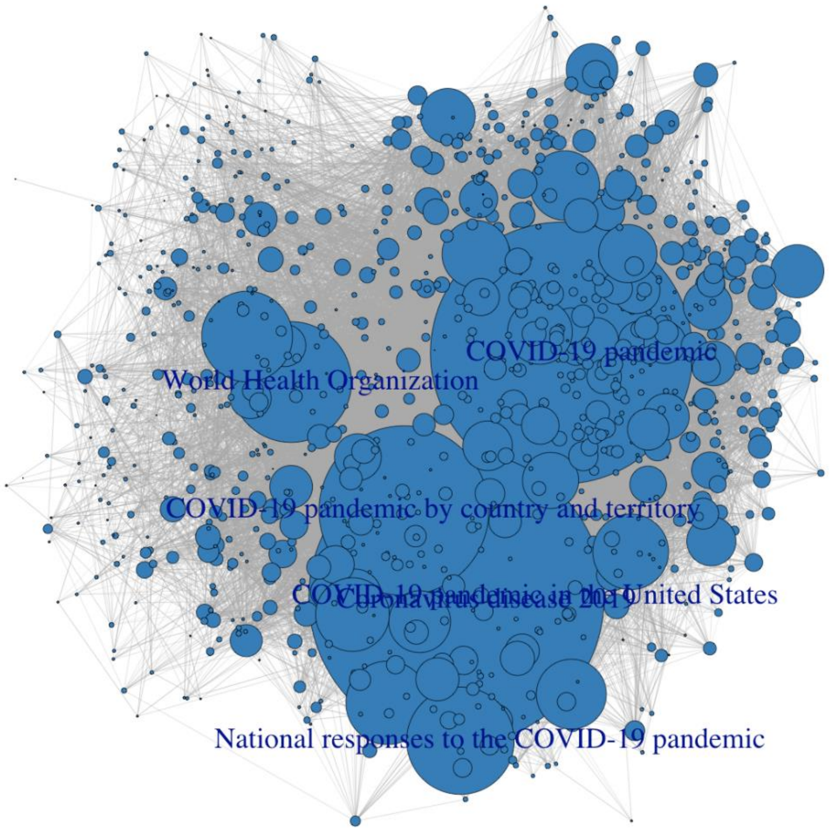

Figure 100. Connectivity graph of Wikipedia articles on health. Popularity of articles displayed by node size. Edges represent co-visits in the 2020 clickstream data.

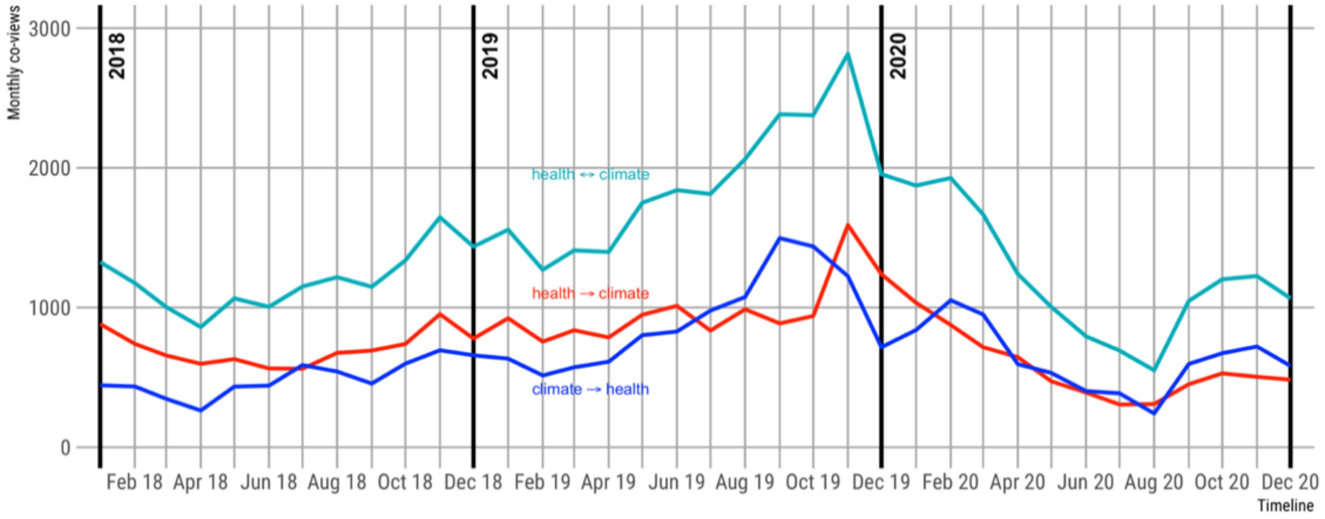

Figure 101. Aggregate monthly co-views of articles related to human health and climate change, 2018—2020 (excluding COVID-19 related articles).

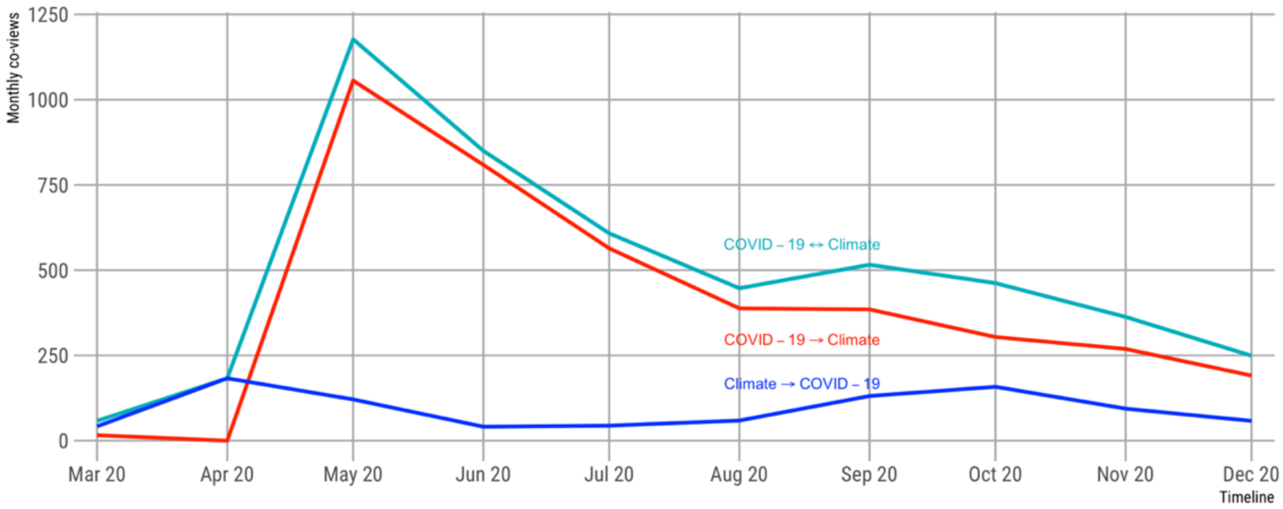

Figure 102. Aggregate monthly co-views of articles related to COVID-19 and climate change, 2020.

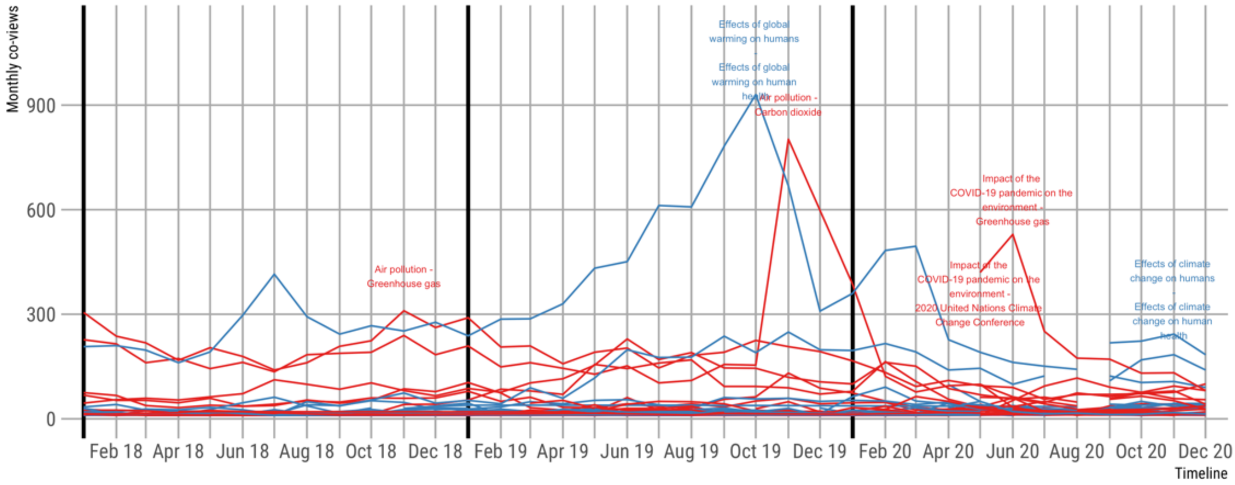

Figure 103. Co-views of climate change-health article pairs over time, 2018-2020. Dominant pairs labelled.

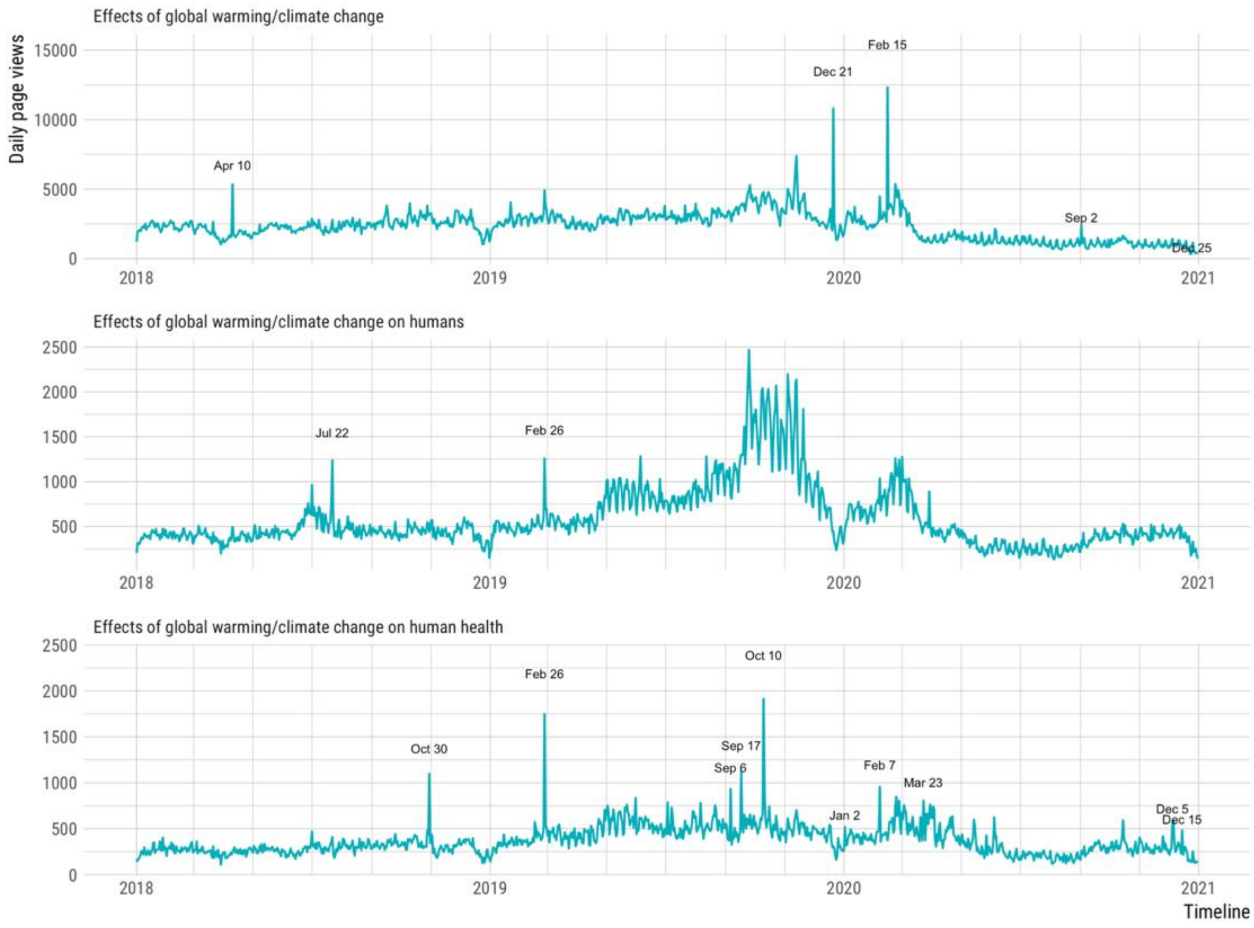

Figure 104. Daily page views 2018 to 2020 for Wikipedia articles directly related to the effects of global warming in general, on humans, and on human health.

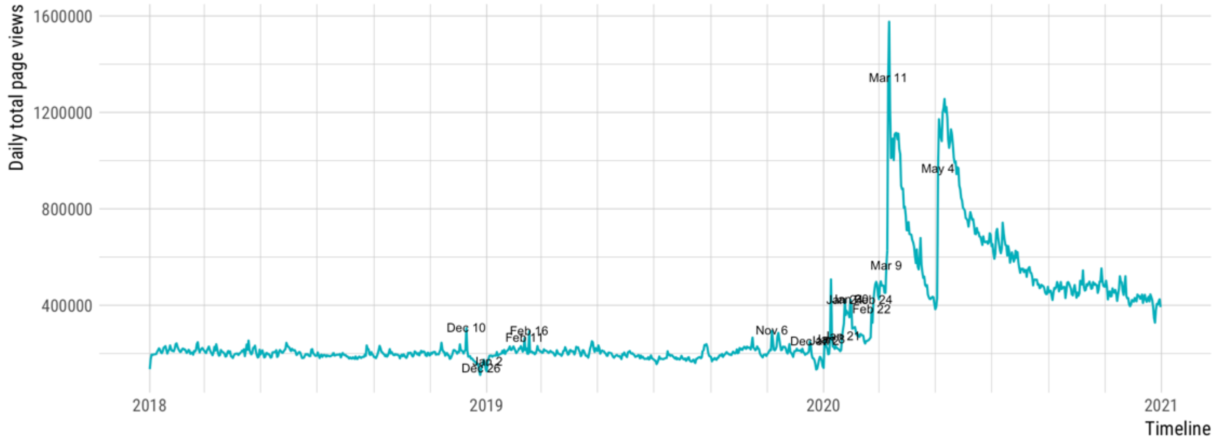

Figure 105. Aggregate daily page views 2018 to 2020 for all 1,414 selected articles on the English Wikipedia related to health.

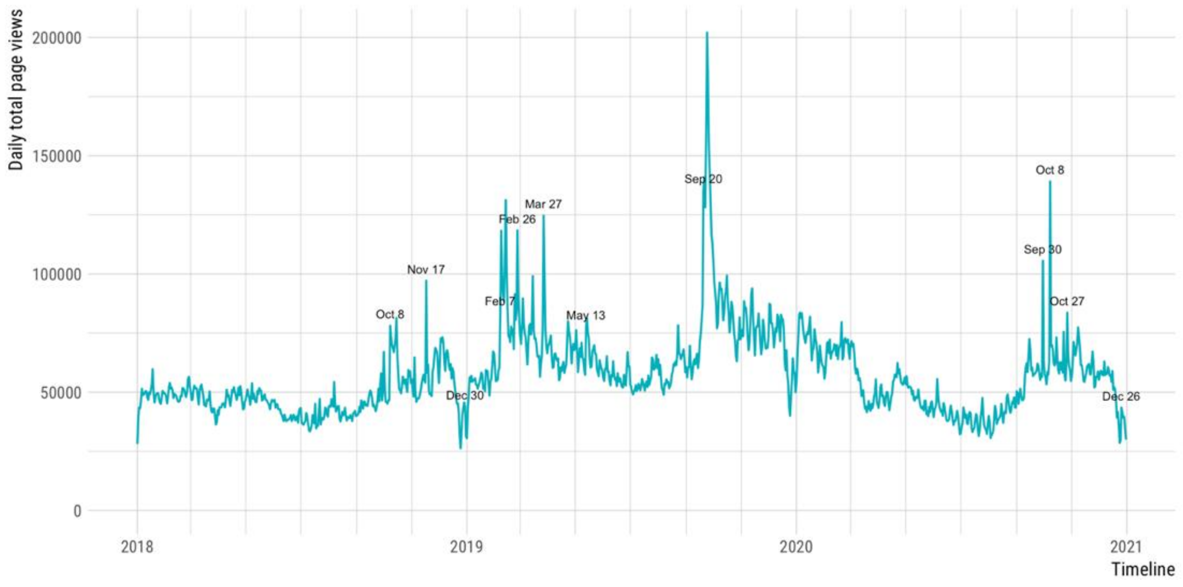

Figure 106. Aggregate daily page views 2018 to 2020 for all 610 selected articles on the English Wikipedia related to climate change.

Indicator 5.3: Coverage of Health and Climate Change in Scientific Journals

Methods

The inclusion of climate-related terms and their co-occurrence with health terms in scientific publications was tracked using a bibliometric search in both Ovid Medline (including Medline In-Process & Other Non-Indexed Citations for those citations not indexed) and Ovid Embase databases.

The Ovid Embase and Ovid Medline databases were selected due to their coverage of health, medical and biomedical sciences, with content that is predominantly journal articles. Ovid Medline contains 25 million citations from 5,600 journals, while Ovid Embase is bigger with 32 million citations from 8,500 journals. Where Medline is predominantly health and biomedicine, Embase has a greater pharmaceutical focus, all of which are relevant to health and climate change. Both databases are updated online daily and can thus provide the annual data (with a 31 December cut-off each year) needed for the indicator. These databases also function through the sophisticated Ovid interface and allow access to the comprehensive indexing systems and thesaurus of Medical Subject Headings (MeSH) for Medline and Emtree for Embase.

Also considered for use were Science Direct and the Web of Science suite of databases, but, with broad subject coverage, these would not enable the necessary search precision.

By screening the retrieved articles between 2007 and 2020, those articles that contained both health and climate change terms in their title or abstract, but do not make any meaningful link between them, were excluded. A meaningful link here means some association between climate change and an aspect of health. This link may be the focus of the article or tangential to it. As an example, climate change may be mentioned at the end of an abstract, where it is noted the health topic that is the focus of the article (e.g. dengue fever distribution) is expected to worsen or change under climate change scenarios.

Data were extracted using search filters that function via Boolean operators (AND, OR, NOT) (see below for final search strategies). For purposes of consistency and efficiency of analysis, the majority of each search filter is designed to produce results with the search terms in either the title or abstract. Indeed, indexing is also likely to be poorly assigned or inconsistently assigned to references. The search filter is designed to retrieve all relevant results (high sensitivity) while keeping irrelevant results, and therefore effort on the part of the researchers, to a minimum (high precision).

To identify articles where associations are made between climate change and health, the filter was split into two facets, one for climate change and one for health. As part of the 2019 Lancet Countdown report, terms that made up the filter were derived using both subjective and objective methods. Subjective methods included utilising terms already known by the research team, as well as those appearing in previous iterations of the Lancet Countdown. Objective methods included the use of online word frequency software (Writewords[9]). Articles looking at health and climate change were run through this software, which organises the words or phrases in order of frequency, allowing relevant terms to be extracted.

Though this process was iterative, the climate change facet was undertaken first, as this was considered to likely consist of fewer terms and be comparatively less complex. All terms were tested independently and alongside other terms: that is, each was input into the OVID databases, from which samples of 100 were drawn and screened for relevance. Terms with high relevance were either piloted or adapted, to be tested alongside other terms and to restrict inclusion to records referring to human health. With different indexing systems, these were then translated between the databases. In addition, terms to ascertain results for editorials, comment sections, and letters were used to compare the volume of these against journal articles.

Estimates of sensitivity for the strategies were established by running the climate change facet through the Ovid interface alone, without the health facet. Samples of 1000 were then extracted and screened for relevant articles. The number of relevant articles found that were also found by the whole search strategy were divided by the

[9] http://www.writewords.org.uk/

number of total relevant articles found, giving an estimate of sensitivity in percentage form. For this indicator, the 90% sensitivity threshold required for systematic reviews was used (Beynon, 2013).

With an acceptable estimate of sensitivity (>90%), results of the search strategies were downloaded into Excel and into two separate libraries: one for Medline (and Medline In-Process & Other Non-Indexed Citations), the other for Embase. The iteration for the 2021 Lancet Countdown report adopted a revised methodology, incorporating the use of base R and R dictionary functions, to improve accuracy in particular areas. These libraries were uploaded into R and duplicates were removed. The libraries were merged and duplicates, shared across both libraries, were also removed. The remaining records were screened for inclusion based on the inclusion and exclusion criteria outlined above for articles making a meaningful link between health and climate change. Results were screened twice by the same researcher. The stepwise process of the selection of articles can be seen in Figure 107.

One of the improvements on the 2020 Lancet Countdown report is in how the indicator deals with climate change-related or health-related article keywords. Where previous analyses included articles with article keywords depicting a relationship between health and climate change, the 2021 Lancet Countdown analysis does not. Though the numbers provided are therefore lower than previous years, this provides more accuracy and less room for researcher bias.

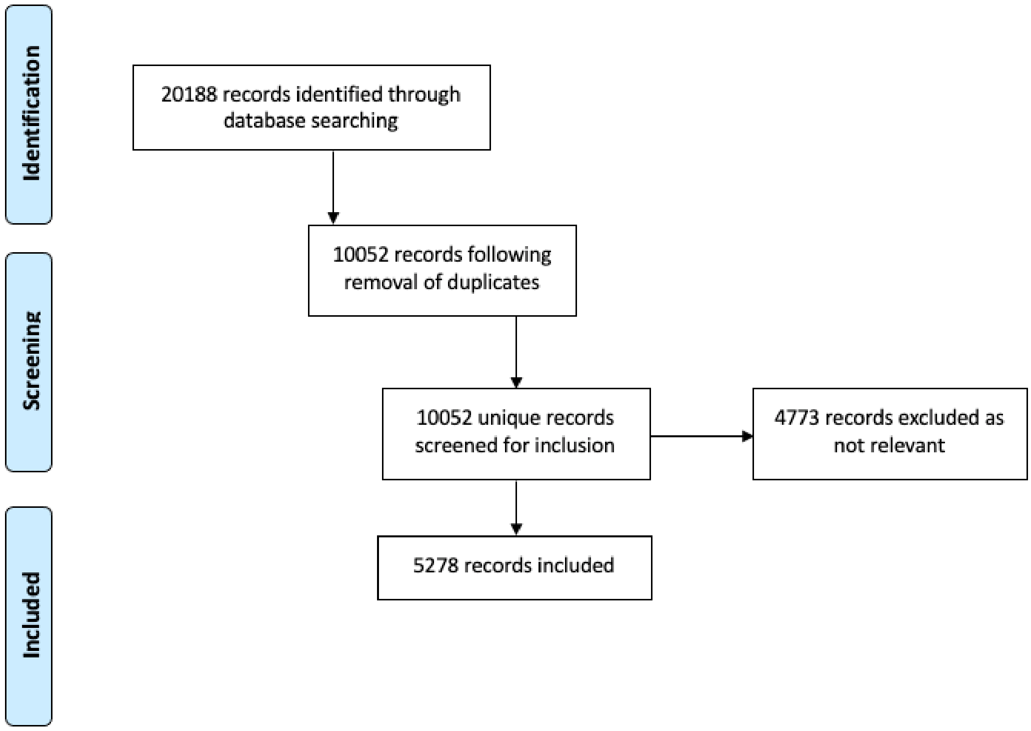

Figure 107. PRISMA flow diagram showing steps of selection process.

Numbers indicate the article count retained at each step of the process. With the applied search terms more than 20,000 scientific articles on health and climate change were identified for the period of 2007-2020. After the screening process, only 26.1% (n=5278) were retained and found to be relevant.

Following screening, precision was established by calculating the number of relevant records retrieved, divided by the total number of unique records retrieved. The development of the search strategy was repeated, and all of the necessary stages leading up to this point, until precision was established at over 50% for each database.

With an acceptable level of precision established for each database, the data were coded and organised in Excel and R. Utilising list and dictionary functions in R also improves the accuracy when finding the location of an

article's first author, as can be seen in the difference between the results in this report and those of previous ones. Lists of institutions, cities and countries are fed into R as dictionaries and these are matched with the information provided by the scientific databases, which is often scattered and difficult to interpret. This also reduces the time it takes to classify first authors based on institution.

Once first author classification was complete, these data were organised by WHO region and 2019 HDI to ascertain how scientific engagement is distributed geographically and in relation to the latest development measures.

The data were also organised according to *type of manuscript*. As with the first author location, text matching functions in R were utilised once more for this task. Primary studies, developing new research or systematically reviewing other research, were separated from research-related articles (i.e. those discussing the implications or direction of research, such as editorials, commentaries and letters, and those comparing research within and between fields, such as reviews). This enabled an appreciation of the volume of new research (including primary research and systematic reviews) as compared to the volume of discussion around that research.

Search terms

Medline		Medline (In-Process & Other Non-Indexed Citations)		Embase	
1	carbon footprint*.ti,ab.	1	(climat* adj3 chang*).ti,ab.	1	(climat* adj3 chang*).ti,ab.
2	carbon footprint/	2	climate variability.ti,ab.	2	Climate Change/
3	(climat* adj3 chang*).ti,ab..	3	(climat* adj3 warming).ti,ab.	3	Greenhouse Effect/
4	climat* cris?s.ti,ab.	4	global warming.ti,ab.	4	greenhouse gas*.ti,ab.
5	climat* variability.ti,ab.	5	greenhouse effect*.ti,ab.	5	global warming.ti,ab.
6	climat* warming.ti,ab.	6	green house effect*.ti,ab.	6	Carbon Footprint/
7	exp Climate Change/	7	greenhouse gas*.ti,ab.	7	Greenhouse Gas/
8	GHG*.ti,ab.	8	(greenhouse adj2 emission*).ti,ab.	8	(greenhouse adj2 emission*).ti,ab.
9	global warming.ti,ab.	9	climat* model*.ti,ab.	9	(climat* adj3 warming).ti,ab.
10	greenhouse effect*.ti,ab.	10	climat* scenario*.ti,ab.	10	GHG*.ti,ab.
11	greenhouse effect/	11	green house emission*.ti,ab.	11	climat* model*.ti,ab.
12	greenhouse emission*.ti,ab.	12	GHG*.ti,ab.	12	climat* variability.ti,ab.
13	greenhouse gas*.ti,ab.	13	carbon footprint*.ti,ab.	13	carbon footprint*.ti,ab.
14	Greenhouse Gases/	14	climate induced.ti,ab.	14	climat* scenario*.ti,ab.
15	climate induced.ti,ab.	15	climat* cris?s.ti,ab.	15	greenhouse effect*.ti,ab.
16	climat* scenario*.ti,ab.	16	health.ti.	16	climate induced.ti,ab.
17	climat* model*.ti,ab.	17	disease*.ti.	17	climat* cris?s.ti,ab.
18	exp Health/	18	infectious.ti.	18	Ep.fs.
19	Global Health/	19	mortality.ti.	19	exp Malignant neoplasm/
20	health status/	20	healthy.ti.	20	exp skin disease/
21	health status disparities/	21	mental.ti.	21	exp lung disease/
22	exp disease/	22	malaria.ti.	22	diabetes mellitus/
23	exp virus diseases/	23	dengue.ti.	23	Disease association/
24	exp viruses/ and human*.ab.	24	respiratory.ti.	24	Western blotting/
25	exp Communicable Diseases/	25	infection*.ti.	25	etiology/

26	Infection/	26	wellbeing.ti.	26	immunology/
27	aedes/	27	well being.ti.	27	Infection/
28	water/ps	28	outbreak*.ti.	28	Death/
29	allergens/	29	zika.ti.	29	Cardiovascular disease/
30	exp Disease Outbreaks/	30	undernutrition.ti.	30	Fever/
31	exp Mortality/	31	influenza.ti.	31	health/
32	mo.fs.	32	hospitali?ation*.ti.	32	Mental disease/
33	exp Malaria/	33	epidemic.ti.	33	Epidemiology/
34	exp disease transmission, infectious/	34	ecohealth.ti.	34	Cerebrovascular accident/
35	exp Neoplasms/	35	ebola.ti.	35	hospital admission/
36	exp Heat Stress Disorders/	36	death.ti.	36	anemia/
37	exp Fever/	37	kills.ti.	37	Chronic disease/
38	exp Metabolic Diseases/	38	cholera.ti.	38	public health/
39	exp Death/	39	foodborne.ti.	39	cancer risk/
40	exp Skin/re	40	epidemics.ti.	40	Virus infection/
41	exp Environmental Illness/	41	endemic.ti.	41	kidney failure/
42	Community-Acquired Infections/	42	pandemic.ti.	42	Mental health/
43	exp Mental Disorders/	43	syndrome.ti.	43	Neurologic disease/
44	Environmental Exposure/ae	44	asthma.ti.	44	Health status/
45	nutrition disorders/	45	illness*.ti.	45	exp Birth weight/
46	child nutrition disorders/	46	morbidity.ti.	46	Human immunodeficiency virus/
47	exp Rickettsiaceae/	47	cancer.ti.	47	exp zoonosis/
48	exp infant nutrition disorders/	48	malnutrition.ti.	48	prophylaxis/
49	exp malnutrition/	49	mental health.ti.	49	Disease transmission/
50	exp wasting syndrome/	50	mental disorder*.ti.	50	Gastrointestinal disease/
51	exp encephalitis/	51	(global adj2 nutrition*).ti.	51	Infection risk/
52	salmonella infections/	52	(population adj2 nutrition*).ti.	52	Mental stress/
53	Helminthiasis/	53	(security adj2 nutrition*).ti.	53	antivirus agent/
54	food contamination/	54	(insecurity adj2 nutrition*).ti.	54	exp allergen/
55	zoonoses/	55	(global adj2 food adj2 (supply or production)).ti.	55	Childhood disease/
56	Noncommunicable Diseases/	56	(security adj2 food).ti.	56	immunogenicity/
57	health.ti.	57	(insecurity adj2 food).ti.	57	malnutrition/
58	disease*.ti.	58	lyme disease.ti.	58	Pregnancy outcome/
59	infectious.ti.	59	Chikungunya.ti.	59	exp *malaria/
60	mortality.ti.	60	Hantavirus.ti.	60	Health hazard/

61	healthy.ti.	61	West Nile disease.ti.	61	Life expectancy/
62	mental.ti.	62	west nile fever.ti.	62	Child development/
63	mental.ti.	63	global disease*.ab.	63	dermatology/
64	malaria.ti.	64	global health.ab.	64	hygiene/
65	malaria.ti.	65	well being.ab.	65	virus detection/
66	dengue.ti.	66	wellbeing.ab.	66	genotoxicity/
67	respiratory.ti.	67	human health.ab.	67	Allergic rhinitis/
68	infection*.ti.	68	vector borne disease*.ab.	68	women's health/
69	wellbeing.ti.	69	health implication*.ab.	69	exp leishmania/
70	well being.ti.	70	public health.ab.	70	encephalitis/
71	outbreak*.ti.	71	health consequence*.ab.	71	Child health/
72	zika.ti.	72	mental health.ab.	72	Communicable disease/
73	undernutrition.ti.	73	reproductive health.ab.	73	virus vector/
74	influenza.ti.	74	health adaptation.ab.	74	infant mortality/
75	hospitali?ation.ti.	75	(mortality adj2 morbidity).ab.	75	Health disparity/
76	epidemic.ti.	76	infectious disease*.ab.	76	Psychological well being/
77	ecohealth.ti.	77	health outcomes.ab.	77	Reproductive health/
78	ebola.ti.	78	health vulnerability.ab.	78	Tropical medicine/
79	death.ti.	79	(health adj2 impact*).ab.	79	Vulnerable population/
80	kills.ti.	80	(health adj2 threat*).ab.	80	Allergic disease/
81	cholera.ti.	81	(burden adj2 disease*).ab.	81	Maternal welfare/
82	foodborne.ti.	82	(population adj2 health).ab.	82	Toxoplasma gondii/
83	epidemics.ti.	83	(health adj2 effect*).ab.	83	Disease burden/
84	endemic.ti.	84	(health adj2 risk*).ab.	84	Childhood mortality/
85	pandemic.ti.	85	(health adj2 benefit*).ab.	85	Dengue virus/
86	syndrome.ti.	86	(health adj2 co-benefit*).ab.	86	Infectious agent/
87	asthma.ti.	87	mental disorder*.ab.	87	respiratory tract allergy/
88	illness*.ti.	88	Noncommunicable Disease*.ab.	88	enterovirus/
89	morbidity.ti.	89	malaria.ab.	89	anopheles/
90	cancer.ti.	90	syndrome.ab.	90	pollen allergy/
91	malnutrition.ti.	91	(tree or trees or soil).ti.	91	campylobacter/
92	mental health*.ti.	92	(people or human* or public health or men or women or children or patients or students).af.	92	exp Heat injury/
93	(global adj2 nutrition*).ti.	93	(editorial or letter or comment).pt.	93	Global health/
94	(population adj2 nutrition*).ti.	94	or/1-15	94	Non communicable disease/
95	(security adj2 nutrition*).ti.	95	or/16-90	95	norovirus/
96	(insecurity adj2 nutrition*).ti.	96	94 and 95	96	Ebola hemorrhagic/
97	(global adj2 food adj2 (supply or production)).ti.	97	96 not 91	97	Health impact assessment/
98	(security adj2 food).ti.	98	97 and 92	98	Yellow fever/

99	(insecurity adj2 food).ti.	99	limit 98 to yr="2007 - 2019"	99	leptospira/
100	Chikungunya.ti.	100	limit 99 to abstracts	100	chikungunya/
101	Hantavirus.ti.	101	100 not 93	101	Arbovirus/
102	West Nile virus.ti.			102	tick-borne disease/
103	west nile fever.ti.			103	Food insecurity/
104	global disease*.ab.			104	Premature mortality/
105	global health.ab.			105	Trihalomethanes/
106	well being.ab.			106	population health/
107	wellbeing.ab.			107	Japanese encephalitis/
108	human health.ab.			108	Crimean-Congo hemorrhagic fever/
109	vector borne disease*.ab.			109	urban health/
110	health implication*.ab.			110	disease*.ti.
111	public health.ab.			111	cancer.ti.
112	health consequence*.ab.			112	health.ti.
113	mental health.ab.			113	infection*.ti.
114	reproductive health.ab.			114	mortality.ti.
115	health adaptation.ab.			115	respiratory.ti.
116	(mortality adj2 morbidity).ab.			116	death.ti.
117	infectious disease*.ab.			117	healthy.ti.
118	syndrome.ab.			118	mental.ti.
119	health outcomes.ab.			119	asthma.ti.
120	health vulnerability.ab.			120	influenza.ti.
121	(health adj2 impact*).ab.			121	illness*.ti.
122	(health adj2 threat*).ab.			122	malaria.ti.
123	(burden adj2 disease*).ab.			123	infectious.ti.
124	(population adj2 health).ab.			124	outbreak*.ti.
125	(health adj2 effect*).ab.			125	hospitali?ation*.ti.
126	(health adj2 risk*).ab.			126	epidemic.ti.
127	(health adj2 benefit).ab.			127	dengue.ti.
128	(health adj2 co-benefit*).ab.			128	endemic.ti.

129	mental disorder*.ab.			129	well being.ti.
130	Noncommunicable Disease*.ab.			130	pandemic.ti.
131	malaria.ab.			131	cholera.ti.
132	mycotoxins/ not food contamination/			132	ebola.ti.
133	respiratory tract diseases/			133	zika.ti.
134	Aspergillus/			134	west nile virus.ti.
135	Candida/			135	epidemics.ti.
136	exp candida/			136	wellbeing.ti.
137	exp aspergillus/			137	Hantavirus.ti.
138	Disease Susceptibility/			138	(insecurity adj2 food).ti.
139	encephalitis/			139	kills.ti.
140	HIV infections/			140	(global adj2 food adj2 (supply or production)).ti.
141	bacterial infection/			141	flavivirus.ti.
142	or/1-17			142	(global adj2 nutrition*).ti.
143	or/18-131			143	(security adj2 nutrition*).ti.
144	or/18-141			144	ecohealth.ti.
145	(tree or trees).ti.			145	(security adj2 food).ti.
146	soil.ti.			146	(mortality adj2 morbidity).ab.
147	exp animals/ not humans.sh.			147	public health.ab.
148	142 and 143			148	mental health.ab.
149	142 and 144			149	infectious disease*.ab.
150	148 not 145			150	well being.ab.
151	150 not 146			151	malaria.ab.
152	151 not 147			152	health outcomes.ab.
153	149 not 145			153	(health adj2 effect*).ab.
154	153 not 146			154	human health.ab.
155	154 not 147			155	mental disorder*.ab.
156	155 NOT 152			156	(burden adj2 disease*).ab.
157	limit 152 to yr="2007 - Current"			157	(health adj2 impact*).ab.
158	limit 155 to yr="2007 - Current"			158	wellbeing.ab.
159	(editorial or letter or comment).pt.			159	global health.ab.
160	157 not 159			160	gastroenteritis.ab.
161	158 not 159			161	(population adj2 health).ab.
				162	reproductive health.ab.
				163	(health adj2 threat*).ab.

				164	health consequence*.ab.
				165	health implication*.ab.
				166	flavivirus.ab.
				167	aeroallergens.ab.
				168	vector borne disease*.ab.
				169	(health adj2 co-benefit*).ab.
				170	health adaptation.ab.
				171	or/1-17
				172	or/18-170
				173	(tree or trees).ti.
				174	soil.ti.
				175	(exp animal/ or nonhuman/) not exp human/
				176	or/172-174
				177	171 and 172
				178	177 not 176
				179	limit 178 to yr="2007 - 2019"
				180	limit 179 to abstracts

With the libraries merged and organised, all abstracts and titles were searched for both gender (from 2007 to 2020) and COVID-19 (for 2020) keywords using text-matching functions in R. Occurrences of these were then counted for analysis.

<u>Gender and COVID-19 keywords</u>

Gender	**“gender”, “gendered”, “maternal”, “prenatal”, “postnatal”, “antenatal”, “pregnancy”, “pregnant”, “reproductive”, “domestic violence”**
COVID-19	**“covid19”, “covid-19”, “corona”, “coronavirus”, “corona virus”, “sars-cov-2”**

Data

Data were taken from Ovid Embase and Ovid Medline databases. The bibliometric search worked with specific inclusion and exclusion criteria that were applied to capture only the most relevant literature. This includes peer-reviewed scientific articles on health and climate change in English, with no direct restriction to country or population applied. All peer-reviewed articles reporting the findings of original qualitative and quantitative studies will be included, together with reviews, editorials, viewpoints, letters or comments; those in the latter category (reviews, editorials, viewpoints, letters, comments) will be filtered for analysis. This practice – of including reviews, editorials, viewpoints and comments – was followed in the 2017, 2018 and 2020 Lancet Countdown reports and provides an indication of scientific engagement outside of peer review (in analyses presented in these earlier Lancet Countdown reports, it was noted that apparent increases in engagement can reflect increases in comments and editorials rather than in original science).

Caveats

The methodology provided here enables a quantitative appraisal of the research question. The quality of the data and the specifics of its content are not assessed by the indicator team. However, with the outputs all published in peer-reviewed journals, there is a de facto check on quality. For this reason, the indicator does not cover grey literature.

Future form of the indicator

There remains scope to formulate add-ons to the indicator, for example focusing on trends in scientific coverage of particular climate-sensitive health outcomes and/or regions.

The validation of results will be subject to review prior to the 2022 Lancet Countdown report and a more robust process put in place to maintain accuracy.

In addition, for the 2022 iteration keywords for gender and COVID-19 will be revisited.

Additional analysis

Proportion of coverage in relation to Embase total publications

Set against a backdrop of annually increasing publications in both databases, the shift in scientific engagement, more generally, can be approximated by the number of articles in a scientific database as a whole compared to those for climate change and health and climate change. Figure 108 highlights that both health and climate change and climate change are increasing in proportion of scientific interest, though climate change has a steeper curve and therefore a greater rate of increase. Against 2019, 2020 continues the increasing trend of previous years for engagement in both climate change and health and climate change: both are at their highest in 2020, with climate change making up 0.8% and health and climate change making up 0.06% of all scientific engagement.

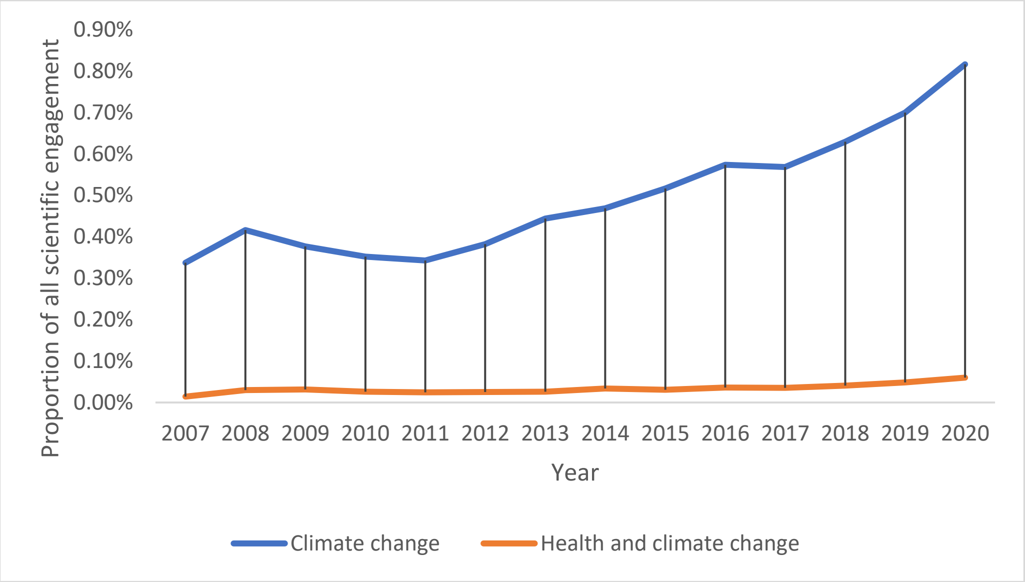

Figure 108. Climate change and health and climate change as proportion of overall scientific engagement. (The Embase scientific database is used here as proxy for overall scientific engagement).

Proportion of health and climate change engagement

Figure 109 demonstrates the total numbers of scientific publications focusing on health and climate change, while Figure 110 shows the same but in in relation to scientific publications based on climate change key words only.

Besides a dip in 2015, as can be seen in Figure 109, engagement with health and climate change has increased year on year, with an overall increase of 959%. 2020 sees a 28% increase in engagement from 2019, the biggest increase since 2014 (34%). As can be observed in Figure 110 most years see an increase in engagement with climate change too, with an overall increase of 318%. While this is smaller than health and climate change, it is based on far lower absolute numbers.

As for the proportion of climate change engagement that also engages with health, this is highest earlier on with 2008 and 2009 standing at 9% and 10%, respectively. However, in 2020 this once again reaches 10% despite the 290% increase in climate change numbers from 2009 to 2020.

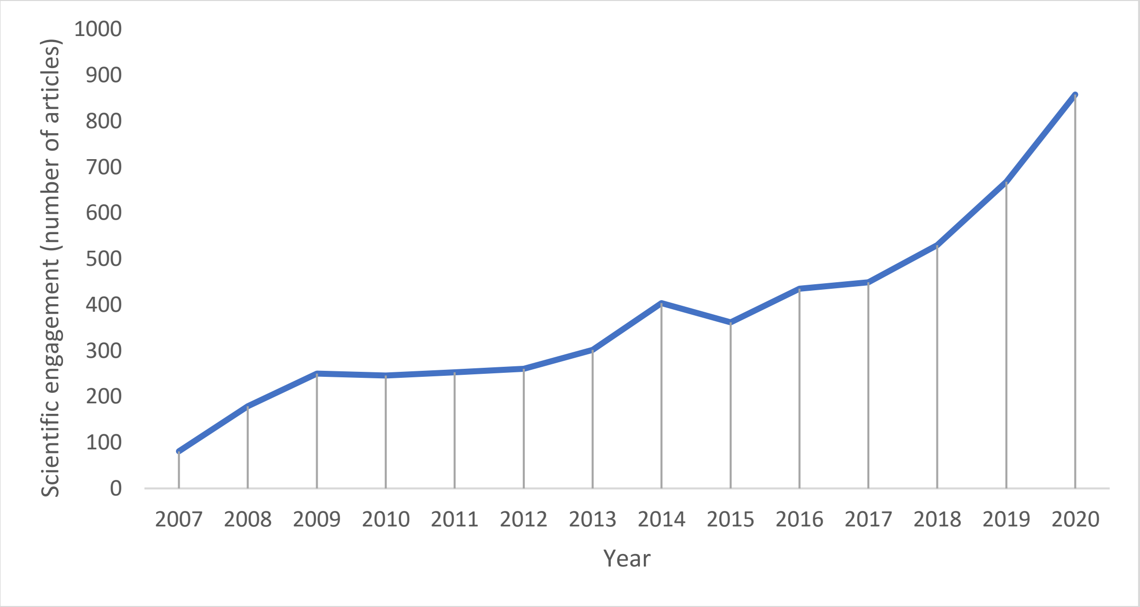

Figure 109. Engagement with health and climate change from 2007 to 2020.

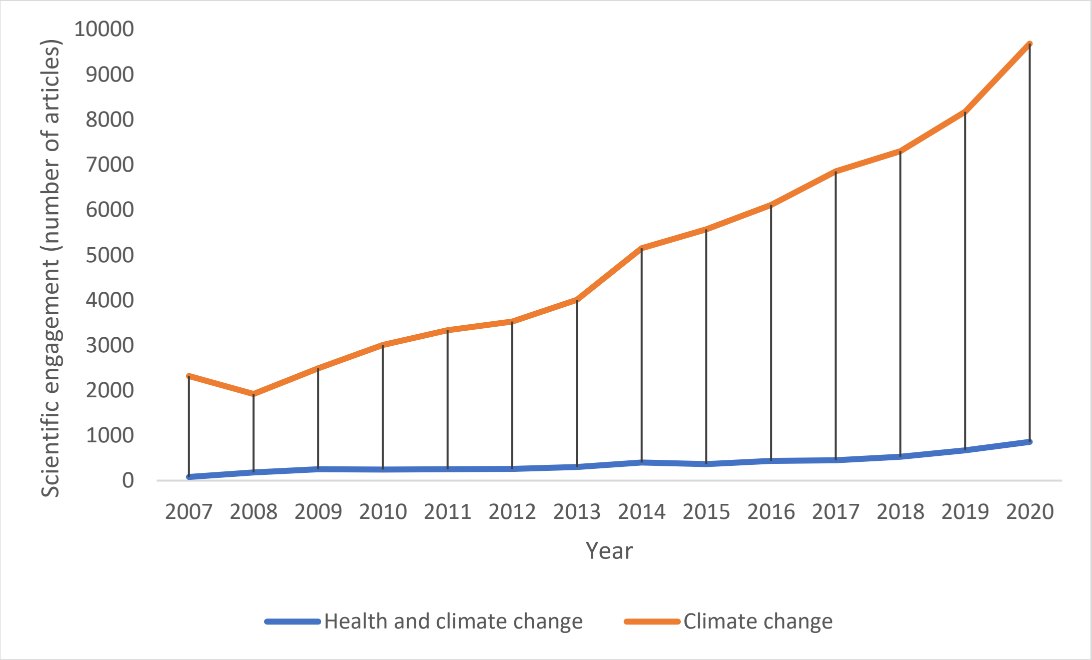

Figure 110. Engagement with climate change and health and climate change from 2007 to 2020.

Geographic distribution of scientific engagement

Figure 111 shows the average number of annual publications per WHO region. The European region and region of the Americas contribute most, with averages consistently above the Western Pacific region and well above the African, Eastern Mediterranean and South East Asian regions. In 2020, the European region has the highest average number of publications (314), followed closely by the Americas (289). All regions increase in average engagement from 2019 to 2020. The two highest contributors, Europe and the Americas, see increases of 52% and 10%, respectively, though the biggest increase, albeit from lower absolute numbers, is the South East Asian region (114%).

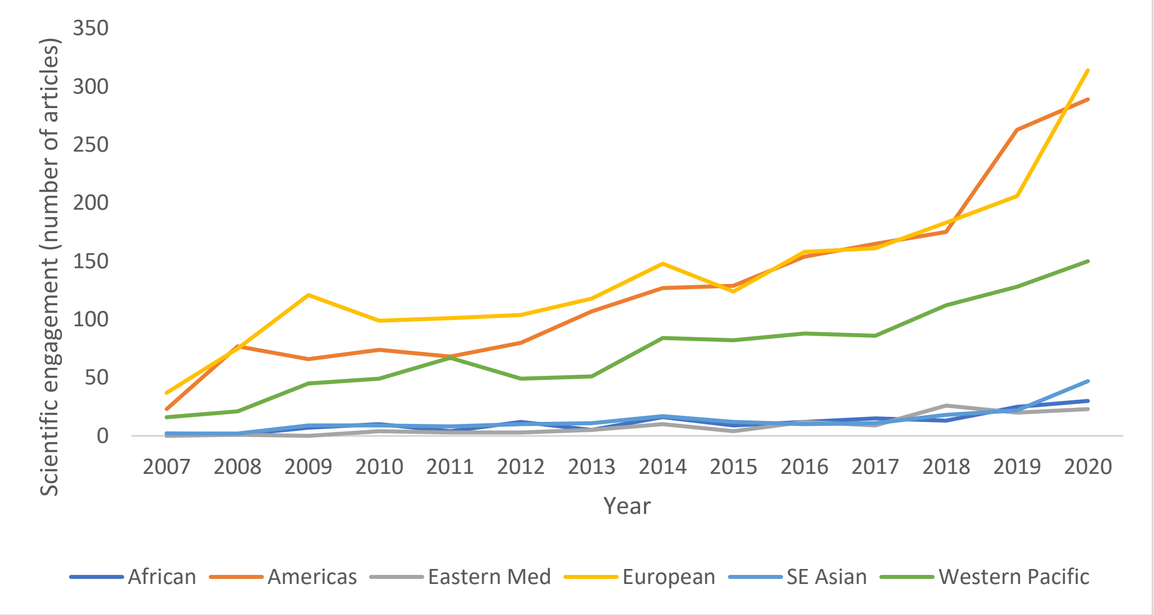

Figure 111. Scientific engagement with health and climate change broken down by WHO region, from 2007 to 2020.

Organising the data into HDI classification groups, as in Figure 112, demonstrates the bulk of engagement is carried by researchers in countries within the Very High HDI group. Very High HDI group countries make up 81% of all scientific engagement, with High HDI group countries contributing 14%, Medium HDI group countries 4% and Low HDI group countries just 1%.

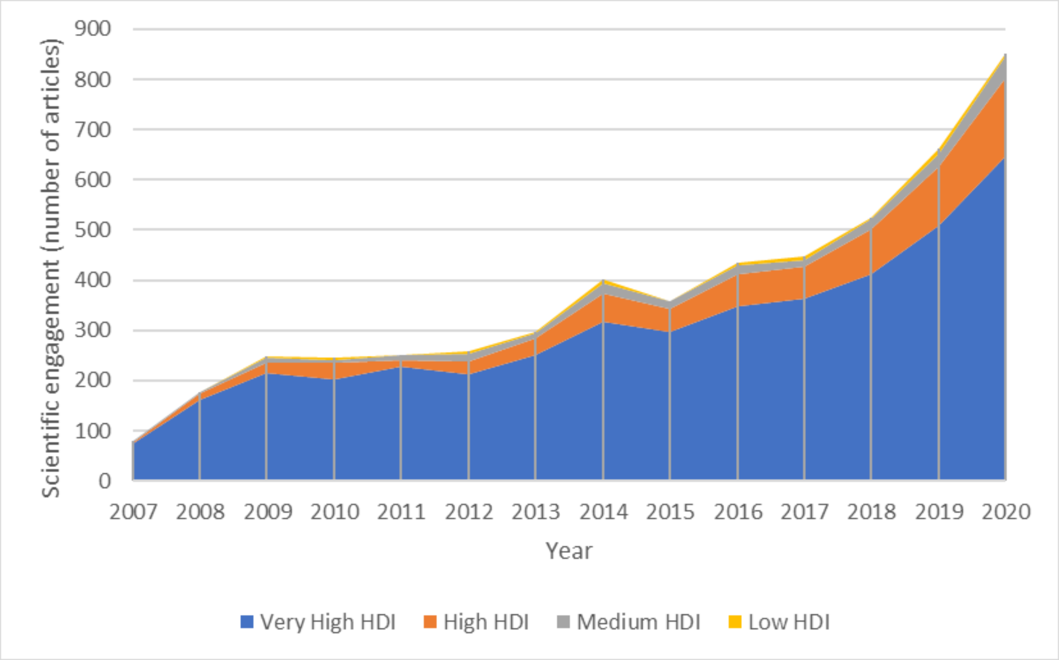

Figure 112. Total scientific engagement with health and climate change broken down by HDI classification group, from 2007 to 2020.

When these data are averaged by the numbers of observations per HDI group (see Figure 113), the general picture remains the same with Very High HDI group countries contributing 67% of all scientific engagement, High HDI group countries making up 20%, Medium HDI group countries 10%, and Low HDI group countries with 3%.

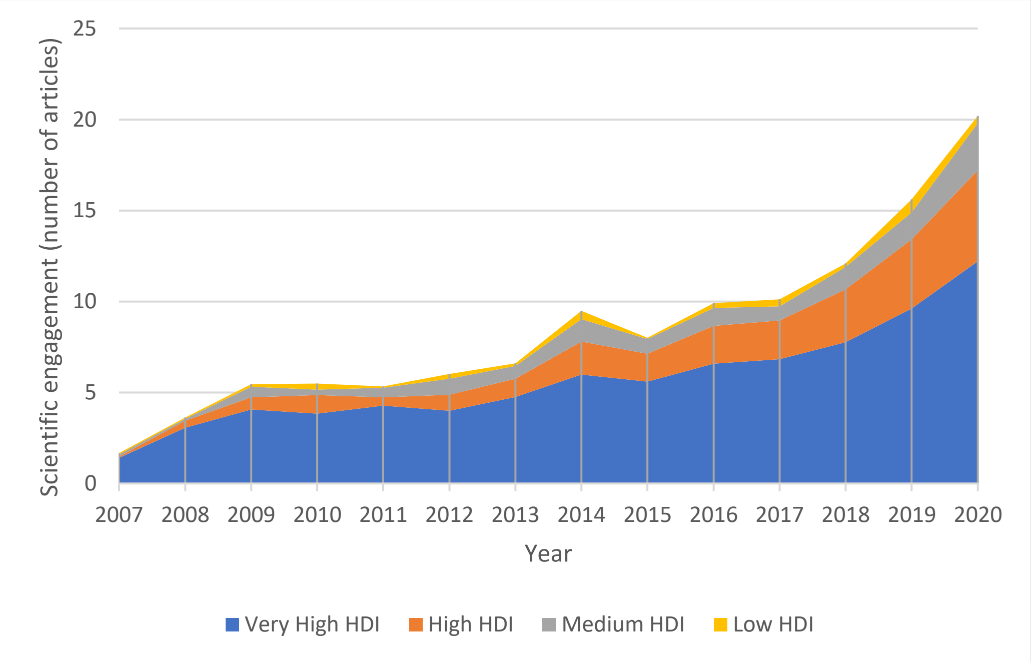

Figure 113. Average annual scientific engagement with health and climate change broken down by HDI classification group, from 2007 to 2020.

Scientific engagement with health, climate change and COVID-19

Figure 114 breakdown scientific engagement with health, climate change and COVID-19 by HDI classification group and WHO region. From **A** it is clear that the dominance of the Very High HDI groups in engagement with health and climate change is carried over into engagement with health, climate change and COVID-19, with this

group accounting for 78%. There is no observable engagement with health, climate change and COVID-19 for Low HDI group countries, with the remainder spread across High (17%) and Medium (5%) HDI groups. As is evident in **B**, this translates over to WHO regions, as those regions with more countries in the Very High HDI group, such as the Americas, Europe and the Western Pacific, contribute most to scientific engagement with health, climate change and COVID-19.

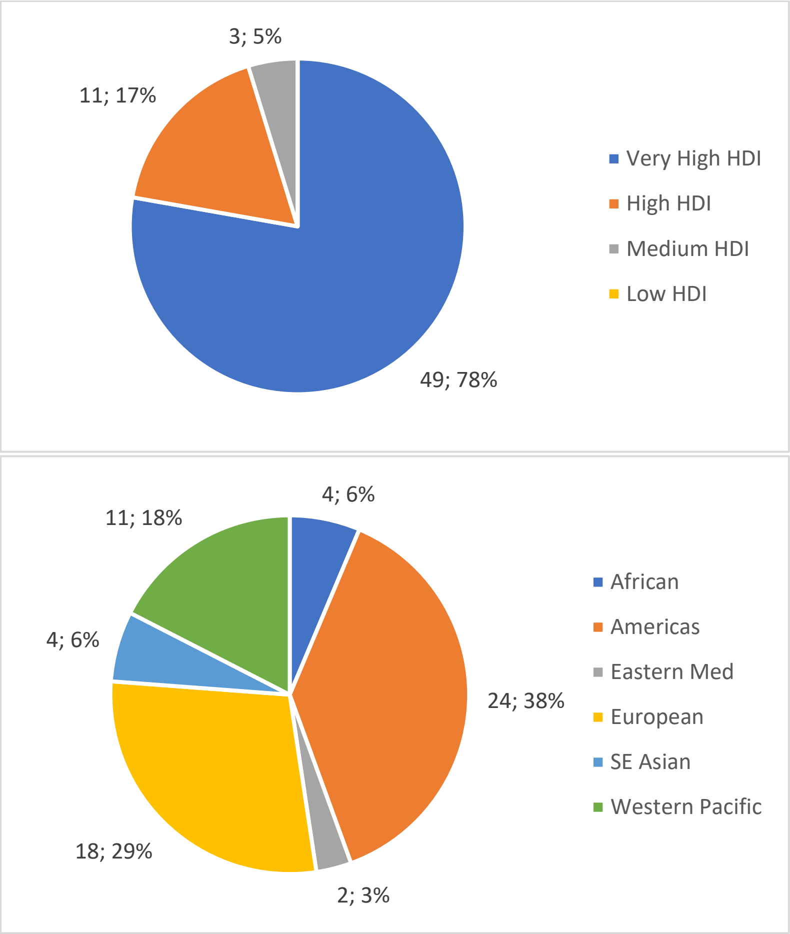

Figure 114. Scientific engagement with health, climate change and COVID-19 in 2020 broken down by HDI classification group (top) and WHO region (bottom).

Scientific engagement with health, climate change and gender

Scientific engagement with health, climate change and the topics delineated by the gender keywords was low overall across the 14-year period. However, as can be seen in Figure 115 which shows the proportion of health and climate change containing a gender keyword, the trend over this time represents a gradual increase, with highs of over 7% in 2017. When organised by WHO region or HDI classification group, there are few discernible features of interest, due to low numbers. However, the WHO region of the Americas is notably higher in 2020 with almost half (49%) of all engagement.

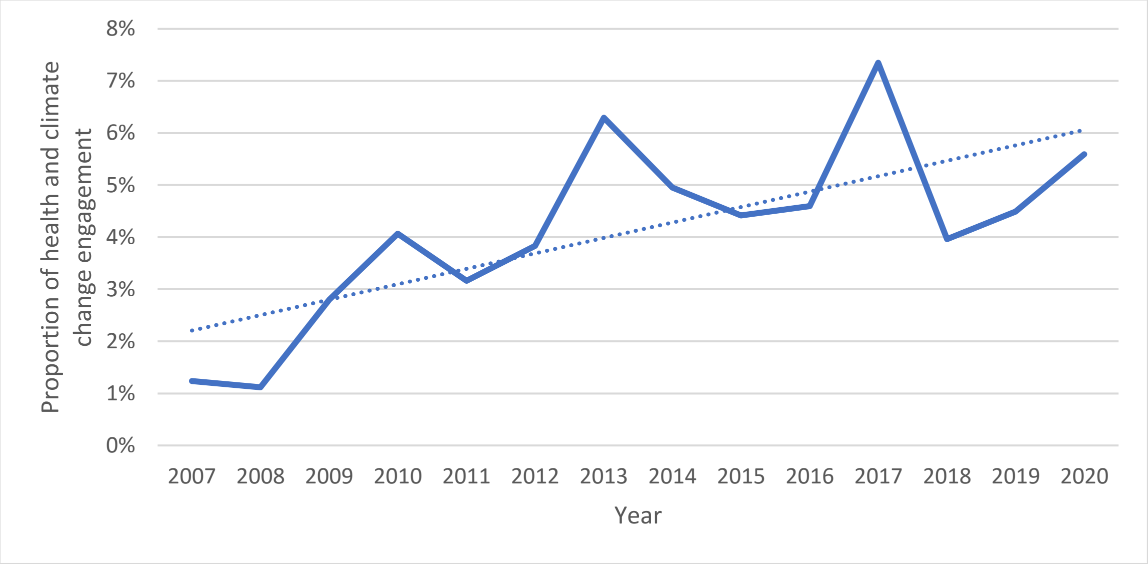

Figure 115. Proportion of health and climate change engagement including gender keyword in title or abstract, from 2007 to 2020.

Primary and secondary health and climate change engagement

Figure 116 shows the split between original and non-original research in relation to health and climate change. Whereas in previous iterations of the Lancet Countdown (2018, 2019), non-original research is higher than original research earlier on (2007-2013), the shift in methodology here demonstrates a lower level of non-original research. Here, though close up until 2008, original research remains the more substantial form of engagement over the whole 14-year period. Overall, original research makes up almost three quarters (74%) of all engagement, against 26% non-original engagement.

Engagement in the form of original research, since 2007, has increased by 1184%, compared with 577% for non-original research. 2020 also saw a larger increase in engagement, from 2019, for original research (+32%), compared with non-original research (+19%).

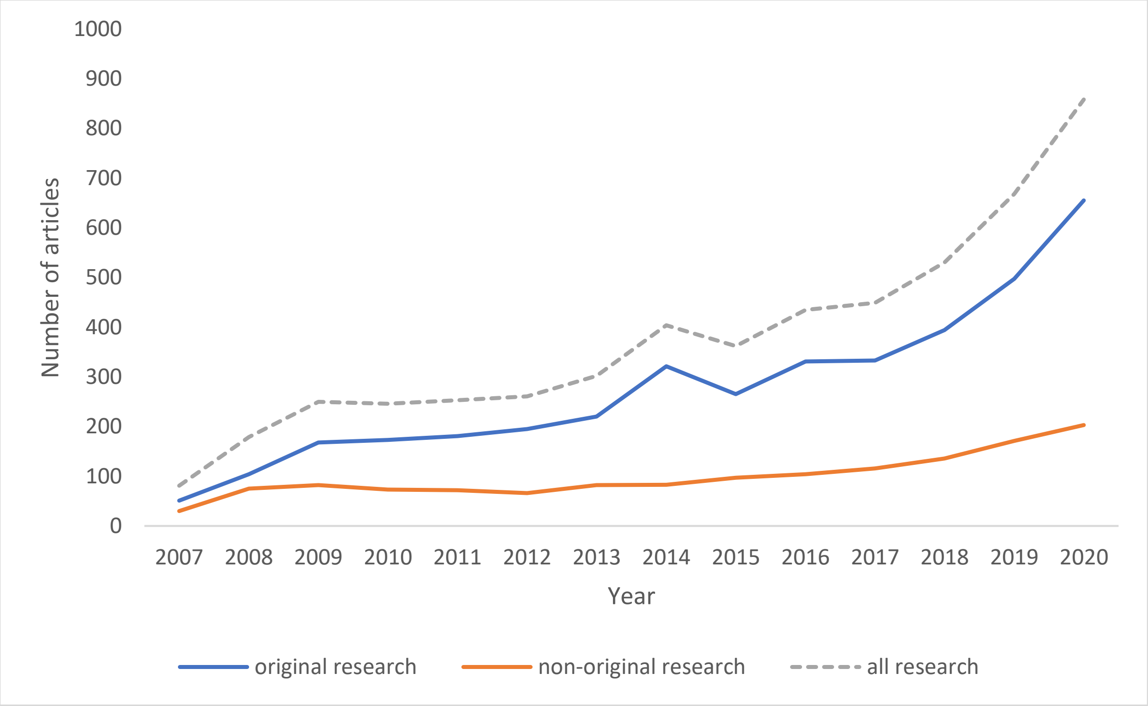

Figure 116. Scientific engagement with health and climate change broken down by original and non-original research.

Indicator 5.4: Government Engagement in Health and Climate Change

Methods

In order to produce the measure of high level political engagement with climate change and health in the UN General Assembly, a new dataset of UN General Debate (UNGD) statements is used, as discussed below. This approach to using UNGD statements to produce the indicators is based on the application of natural language processing to the corpus of UNGD statements. References to key search terms linked to (a) health, and (b) climate change are identified:

Health terms	Climate change terms
malaria	climate change
diarrhoea	changing climate
infection	climate emergency
disease	climate action
diseases	climate crisis
sars	climate decay
measles	global warming
pneumonia	green house
epidemic	temperature
epidemics	extreme weather
pandemic	global environmental change
pandemics	climate variability
epidemiology	greenhouse
healthcare	greenhouse-gas
health	low carbon
mortality	ghge
morbidity	ghges
nutrition	renewable energy
illness	carbon emission
illnesses	carbon emissions
ncd	carbon dioxide
ncds	carbon-dioxide
air pollution	co2 emission
nutrition	co2 emissions
malnutrition	climate pollutant
malnourishment	climate pollutants
mental disorder	decarbonization
mental disorders	decarbonisation
stunting	carbon neutral
	carbon-neutral
	carbon neutrality
	climate neutrality
	net-zero
	net zero

These key terms have been updated from previous years to reflect the changing terminology used to discuss climate change. In order to produce an indicator of engagement with the intersection of climate change and health, the indicator focused on whether any of the climate change related terms appeared immediately before or after any health terms in the UNGD statements. This was based on a search of the 25 words before and after a reference to a health-related term. The choice of 25-word window context corresponds to approximately half a paragraph of text. Given that UNGD statements are highly structured and methodically developed by governments over prolonged periods of time, it can be assumed that half a paragraph of text around public health terms captures a sufficiently narrow context. A search and count of the number of climate change term references in these contexts

then occurred to produce the measure of engagement with the link between health and climate change. A robustness analysis was conducted by varying the size of the context (5, 10, and 50 words). This substantively produced the same trends over time. An examination was also undertaken of a sample of the references produced by the search as an additional check to ensure that the references identified reflect engagement with the health impacts of climate change.

Data

To produce this indicator, a new and updated dataset of UNGD statements was drawn upon: *the United Nations General Debate corpus*, in which the annual UNGD statements have been pre-processed and prepared for the application of natural language processing to the official English versions of the statements.[308] The dataset contains all of the country speeches made in the UN General Debate between 1970 and 2020. It is worth noting that due to the COVID-19 pandemic, the 2020 UN General Debate consisted of governments providing pre-recorded statements. Table 52 presents summary of the data by year:

Table 52. Summary information for UN General Debate Corpus.

Year	General Debate statements	Total sentences	Total words
**1970**	70	11854	303791
**1971**	116	19901	508506
**1972**	125	21201	540994
**1973**	120	21450	536413
**1974**	129	22041	568739
**1975**	126	21365	534375
**1976**	134	23799	599949
**1977**	140	24799	606549
**1978**	141	25236	626163
**1979**	144	26462	654000
**1980**	149	27191	659225
**1981**	145	26063	633579
**1982**	147	23435	638691
**1983**	149	26803	643068
**1984**	150	27928	662654
**1985**	137	19258	592666
**1986**	149	19030	577525
**1987**	152	18336	563132
**1988**	154	18595	569493
**1989**	153	19440	574379
**1990**	156	17885	522197
**1991**	162	18552	538351
**1992**	167	18597	543138
**1993**	175	20165	587448
**1994**	178	19944	580530
**1995**	172	17870	536741

**1996**	181	18046	522699
**1997**	176	17701	514492
**1998**	181	18883	514836
**1999**	181	18529	531306
**2000**	178	16259	464312
**2001**	189	14748	414683
**2002**	188	13977	380481
**2003**	189	14716	399397
**2004**	192	14899	405290
**2005**	185	13012	353065
**2006**	193	14646	390476
**2007**	191	14586	387883
**2008**	192	14294	384881
**2009**	193	16029	423395
**2010**	189	14439	391954
**2011**	194	16293	429974
**2012**	195	16837	444519
**2013**	193	16400	440898
**2014**	194	15859	421947
**2015**	193	16129	436378
**2016**	194	15990	420155
**2017**	196	16806	439624
**2018**	196	16980	455205
**2019**	195	17526	466114
**2020**	193	15165	396548
**Total**	8481	955949	25732808

The data was pre-processed for analysis by removing punctuation, symbols, numbers, stopwords, and URLs. In addition, all tokens were normalised (lowercased). All pre-processing and analysis was carried out in R using the "quanteda" package.[281]

Caveats

The search for climate change terms in the context of public health references is a proxy for the semantic linkage between the two sets of terms in UNGD statements. This approach produces a scalable and reproducible measure with a high degree of reliability that does not involve human judgement or subjective biases. However, there may be examples of governments referring to climate change and health but not the direct linkages between the two, which are included in the count; and there may be examples of governments discussing the health impacts of climate change in their UNGD statements, which are not included in this measure because the distance between the mention of the climate change term and the health term exceeds 25 words. Based on an analysing a random sample of the speeches and references, such cases are relatively rare and do not have a significant bearing on the indicator or the trends uncovered.

It is also worth noting that the analysis here is based on a narrow range of search terms, which excludes reference to many of indirect links between climate change and health. A number of UNGD statements in this time period refer to such indirect connections, such as the effects of climate change on water and agriculture – however, these are not included here. Therefore, the results present a somewhat conservative estimate of high-level political

engagement with the intersection of climate change and health. Future work in this area will consider engagement with these indirect links.

Future form of indicator

In the future, the indicator team plans to look more closely at the references to indirect links between climate change and health. For example, what are the main ways in which governments view climate change impacting on health? It will be considered whether this changes over time based on awareness of the multiple ways in which climate change and health are connected. Some of the references to the indirect links between climate change and health made in UNGD statements have been highlighted in the main report.

Additional information

Additional findings and breakdowns are presented in this section.

Figure 117 shows the total number of references to health, climate change, and the intersection of the two between 1970 and 2020. Figure 118 presents the total number of references to the intersection in UNGD statements between 1970 and 2020. Figure 119 shows the proportion of countries that engage with the intersection of climate change and health between 1970 and 2020. The Figures shows the substantial increase in engagement with the health dimensions of climate change that occurred in 2020. In 2019 there were 109 separate references – which was significantly higher than in previous years – and in 2020 this more than doubled to 269 individual references to the intersection of climate change and health. As noted in the main report, this is primarily driven by countries discussing climate change and the COVID-19 pandemic together.

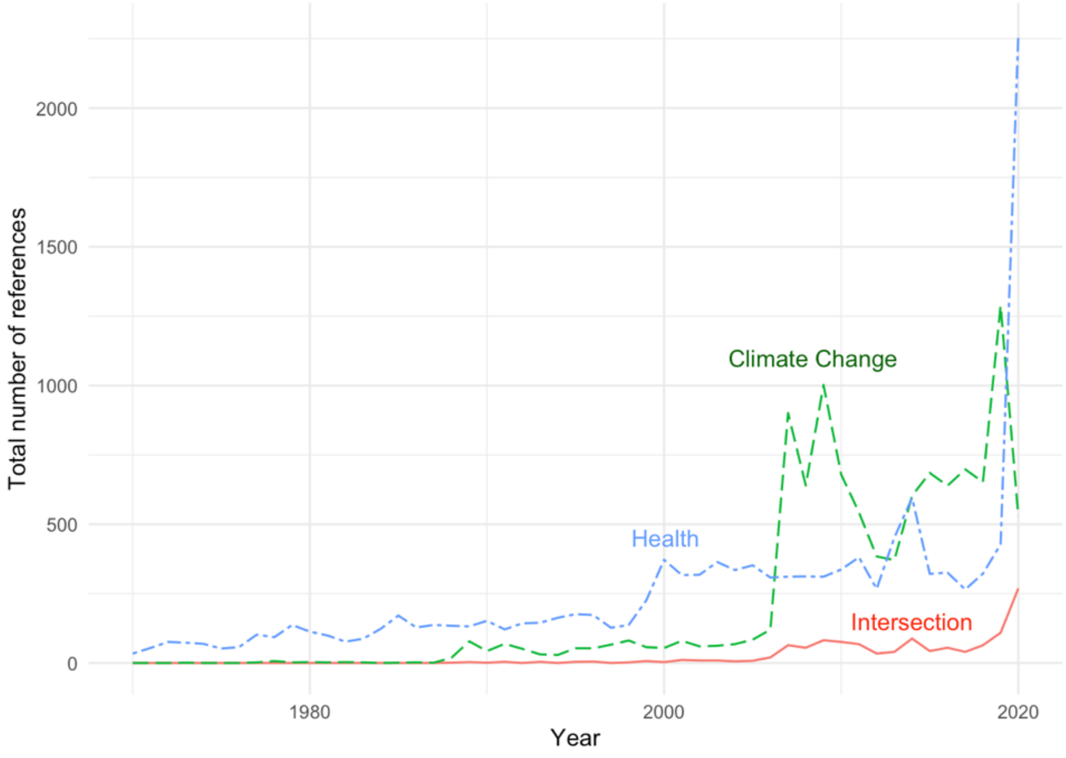

Figure 117.Total number of references to health, climate change, and intersection, 1970-2020.

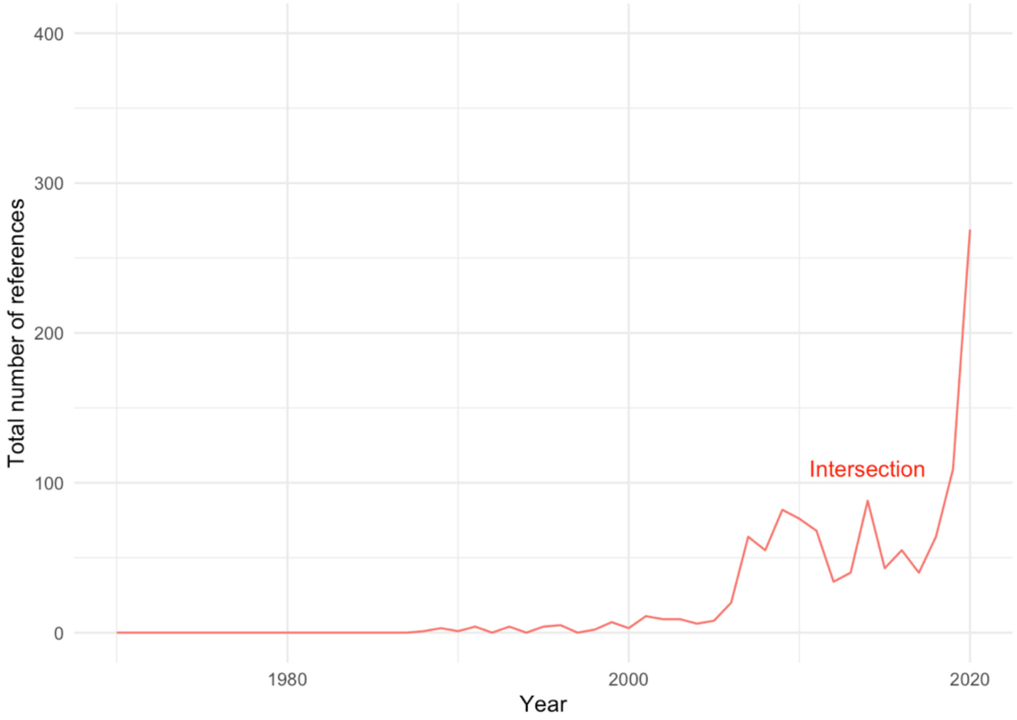

Figure 118. Total number of references to intersection, 1970-2020.

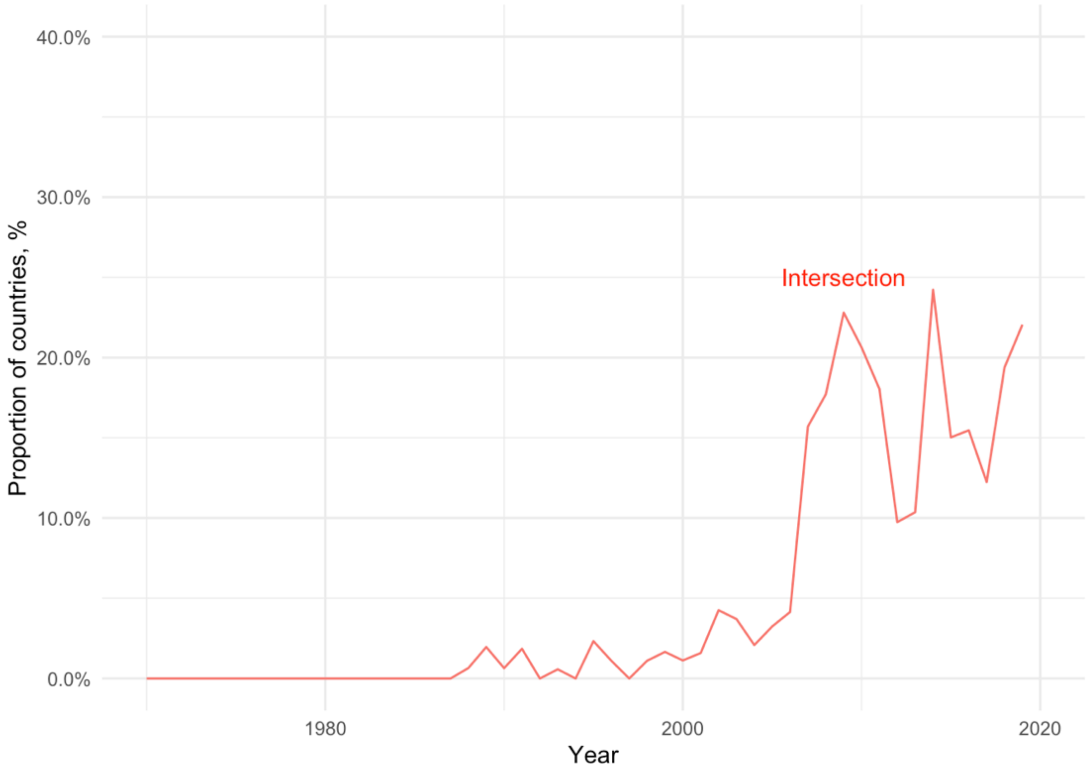

Figure 119. Proportion of countries referring to the intersection of health and climate, 1970-2020.

There is growing awareness of the gendered impacts of climate change and health. The indicator therefore considers the extent to which references to the health dimensions of climate change in countries UNGD statements engage with gender issues. This is achieved by further examining the references to the intersection of climate change and health. Once all references to this intersection in UNGD statements for 1970-2020 are identified, additional search terms related to gender are utilised to identify which of the intersection references also engaged

with gender issues. The gender-related search terms used were as follows: women, women's, maternal, inequality, inequalities, gender, empowerment, sex, sexual, violence, violent, girls, reproduction, reproductive. Hence, the analysis considers whether the 25 words of text identified in the primary search (for climate change and health terms) includes a reference to at least one of these gender-related keywords. Figure 120 shows that 10% of all references to the intersection of climate change and health also include a mention of gender. The Figure shows that this is lower than in previous years, with the 2013 seeing 26% of all climate change-health references including a gender mention.

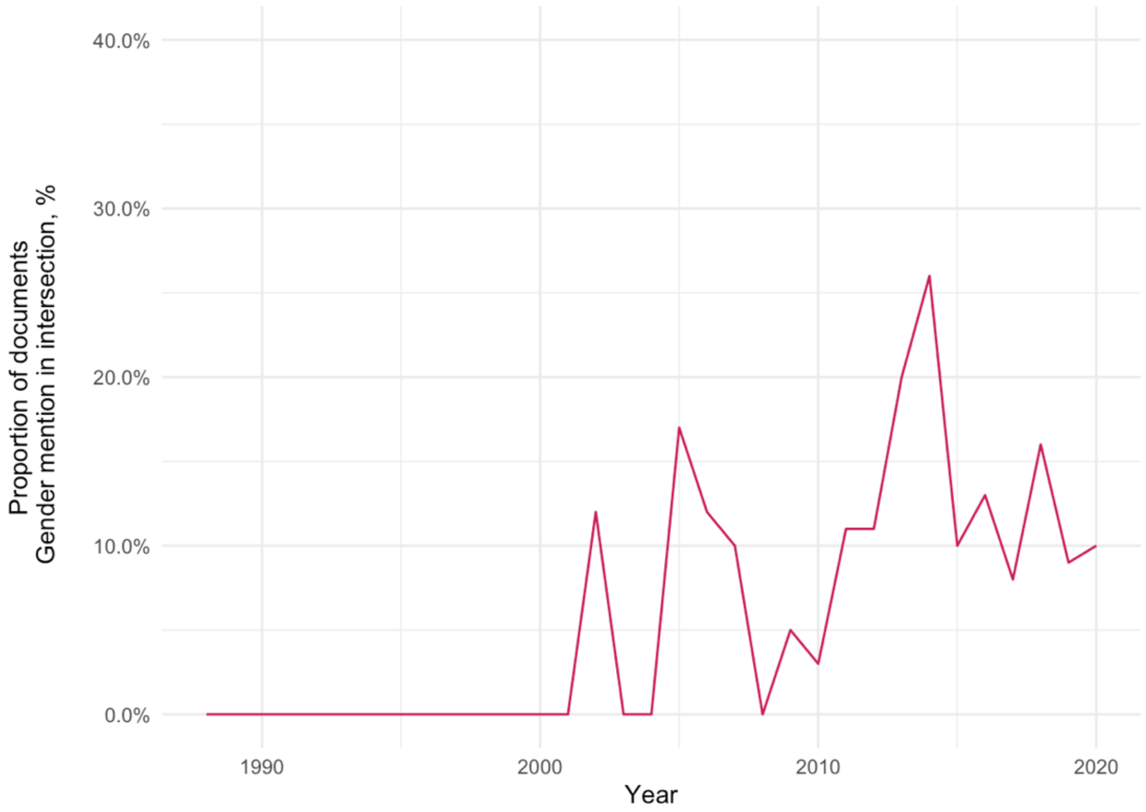

Figure 120. Proportion of references to the intersection of health and climate change that include a reference to gender, 1970-2020.

Figure 121 below presents the proportion of countries that engage with the intersection of climate change and health by WHO region. The significant increase in engagement in 2020 can be seen in all of the regions – with at least 30% of countries in all of the regions referring to the health dimensions of climate change in their 2020 UNGD statements. As in previous years the highest engagement is from countries in the Western Pacific region, with 75% of countries referring to the intersection of climate change and health. It is worth noting that the relatively higher level of political engagement by countries in the Western Pacific is especially driven by the small island development states (SIDS) in this region. The lowest engagement is by countries in the Eastern Mediterranean regions with 30% of countries in this region referring to the intersection of climate change and health.

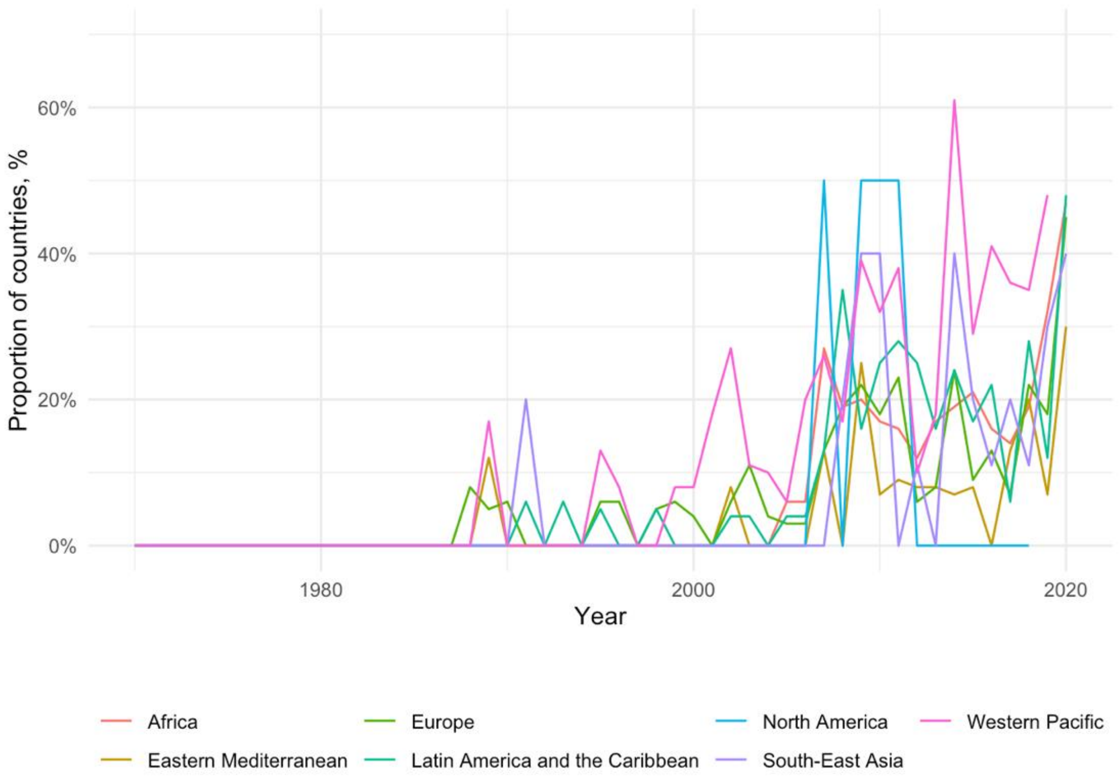

Figure 121. Proportion of countries referring to intersection of health and climate change by region, 1970-2020.

Figure 122, below, presents the total number of references to the climate change-health link between 1970 and 2020 by WHO region. The Figure shows that the highest number of references to the intersection of climate change and health come from four regions: Africa, Europe, Latin America and the Caribbean, and the Western Pacific. In general, the Figure suggests that there is lower engagement among countries in the Eastern Mediterranean, North America, and South-East Asia.

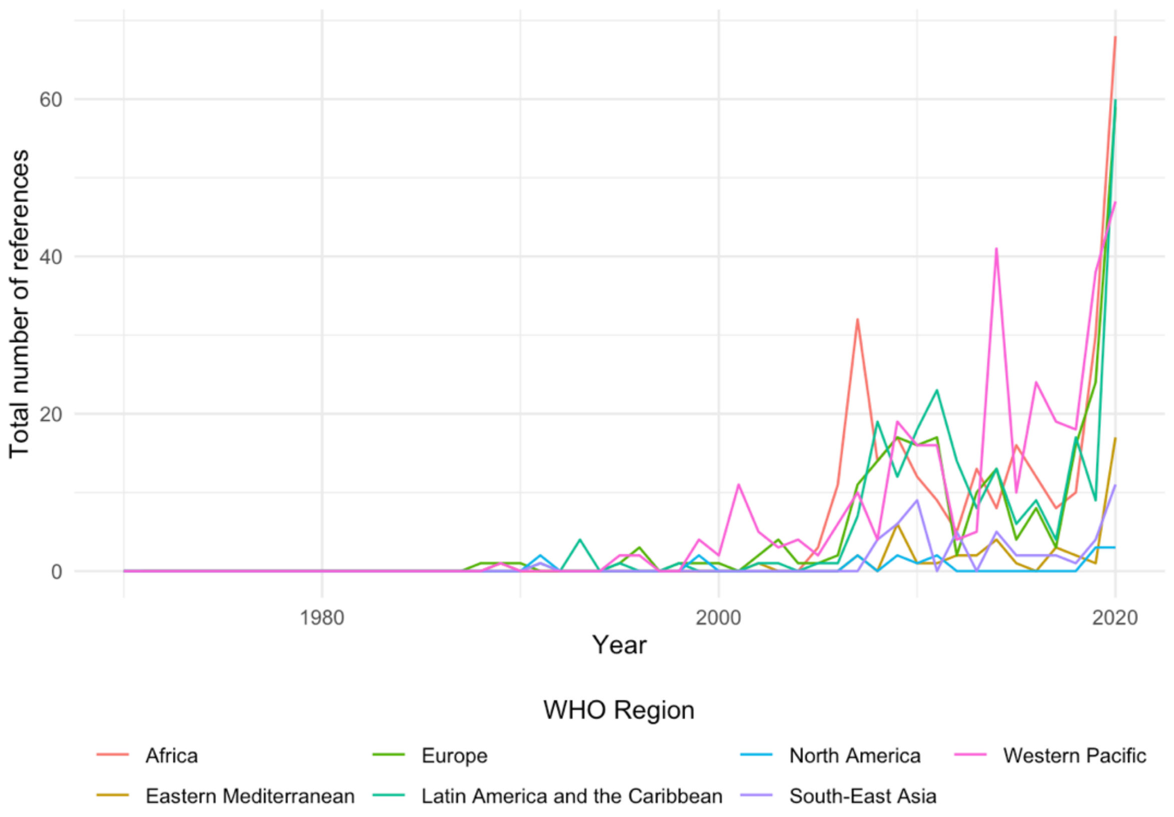

Figure 122. Total number of references to intersection by region, 1970-2020.

In addition to grouping countries by WHO region, the indicator also considers different types of countries in terms of their potential importance and role in addressing issues related to climate change. This is provided in Figure 123 and Figure 124. As noted in previous years' reports, the SIDS have driven much of the engagement with the health impacts of climate change, as well as climate change more generally, in the UN General Assembly. As such, a SIDS grouping is included. Arguably the three most important countries/unions in addressing climate change are USA, China, and the EU. This is both in terms of their carbon dioxide emissions and their power within the international system. This group is referred to as Tier 1 countries in Figure 123 and Figure 124. Finally, the indicator also considers an additional grouping of countries that are also important in terms of their CO2 emissions, their influence in international politics, and their potential impact on addressing climate change. This grouping, referred to as Tier 2 countries, includes: Poland, Australia, South Africa, Brazil, India, France, Germany, and Indonesia.

Figure 123 shows the proportion of countries that engage with the intersection of climate change and health based on these country groupings. Figure 124 shows the total number of references to the climate change-health intersection according to these groupings. Both Figures demonstrate the higher level of engagement with the climate change-health linkages by SIDS than by Tier 1 or Tier 2 countries. However, it is worth noting that Figure 120 shows that a growing number of Tier 1 and Tier 2 countries are engaging with the climate change-health intersection.

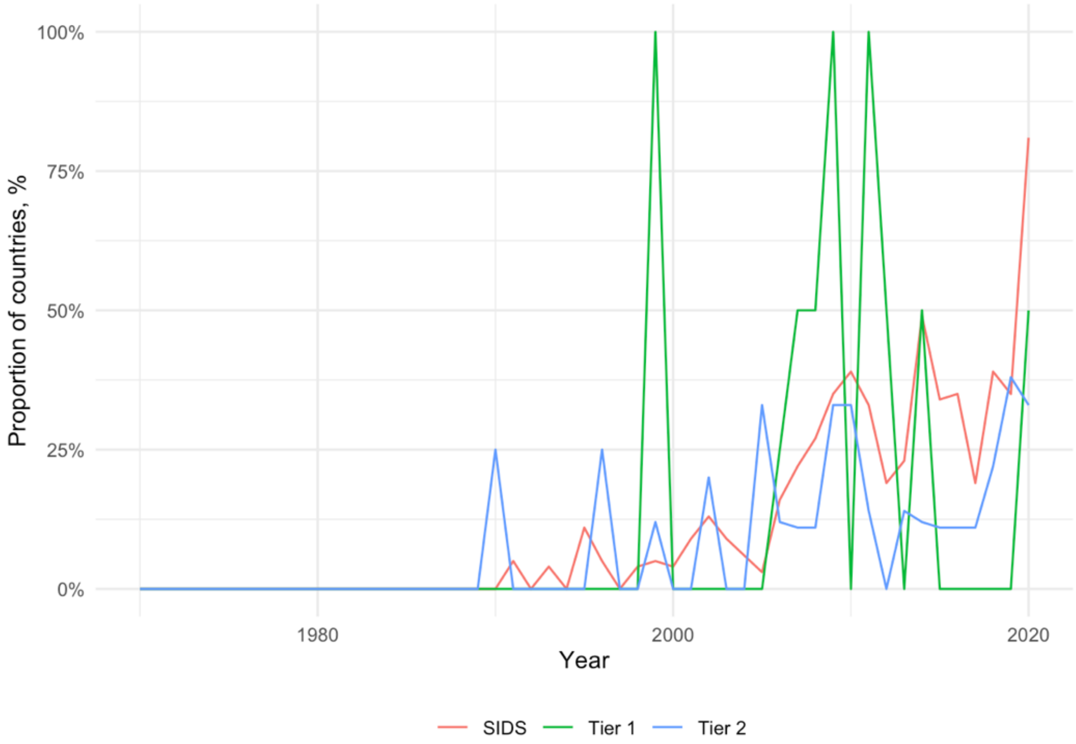

Figure 123. Proportion of countries referring to intersection of health and climate change by country grouping, 1970-2020.

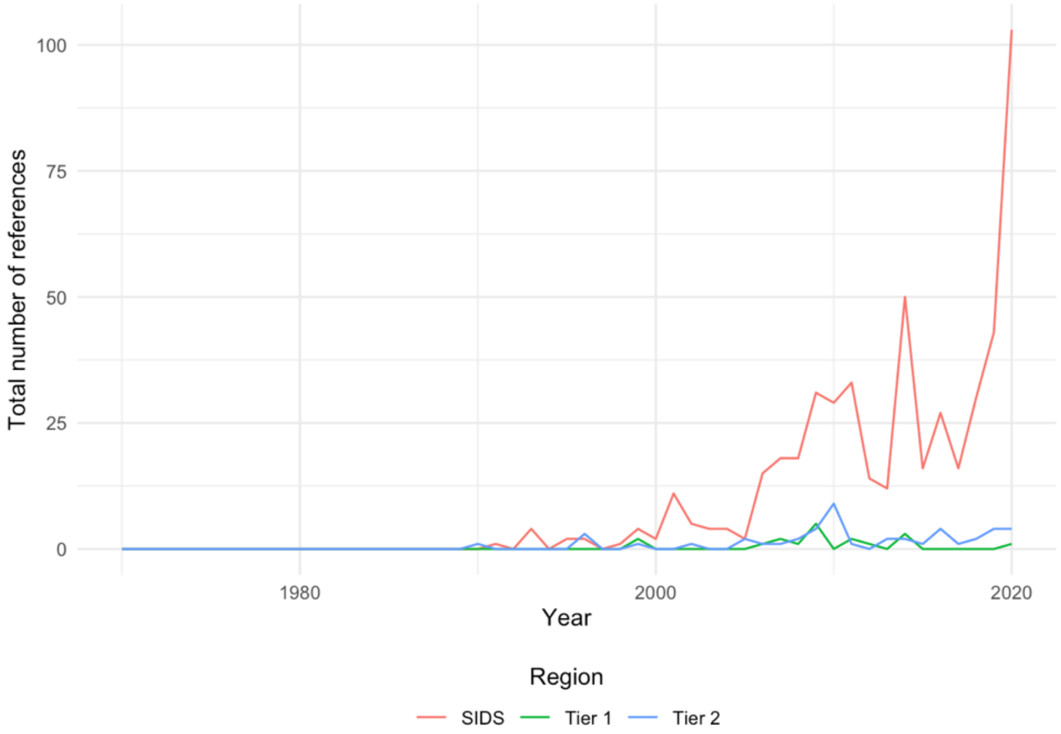

Figure 124. Total number of references to intersection by country grouping, 1970-2020.

The indicator also consider government engagement with the health dimensions of climate according to countries' Human Development Index (HDI) categories. Figure 125 shows the proportion of countries engaging with the intersection of climate change and health by HDI category, and Figure 126 shows the total number of references

by countries' HDI categories. Both Figures show the significant increase in engagement across different HDI groupings.

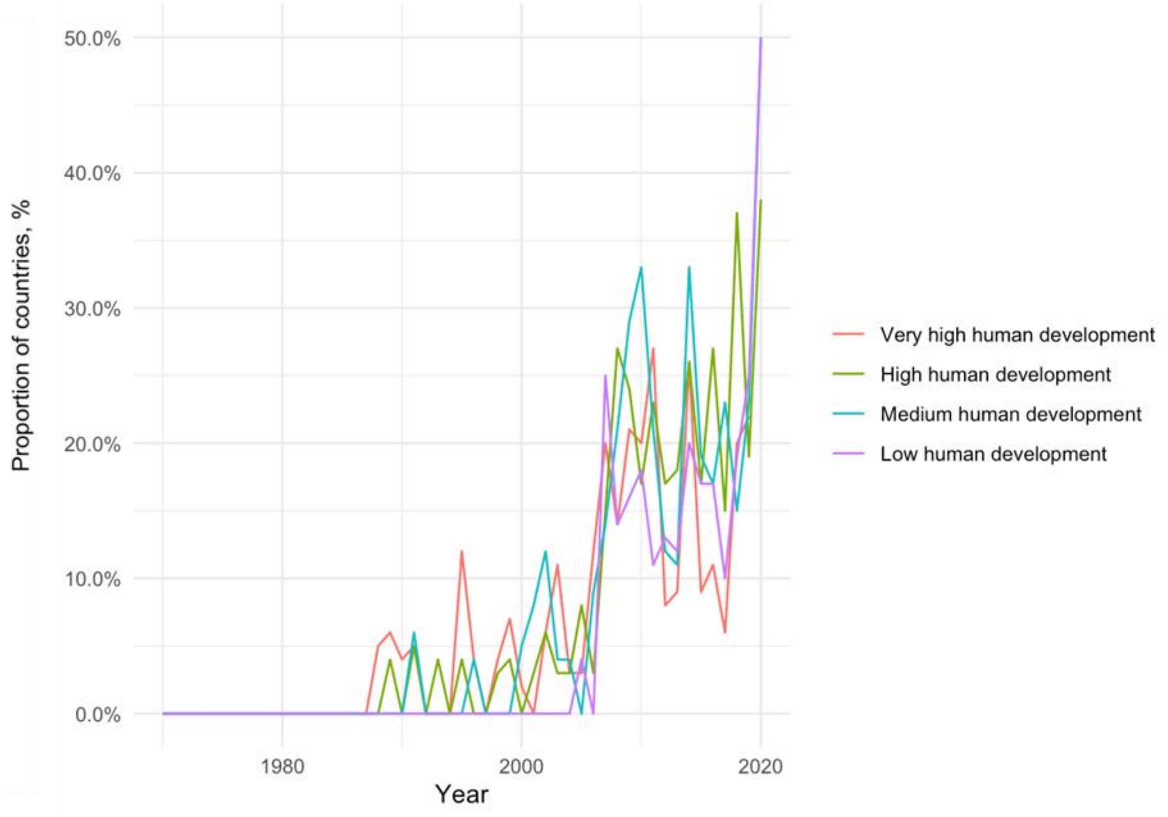

Figure 125. Proportion of countries referring to intersection of health and climate change by HDI categories, 1970-2020.

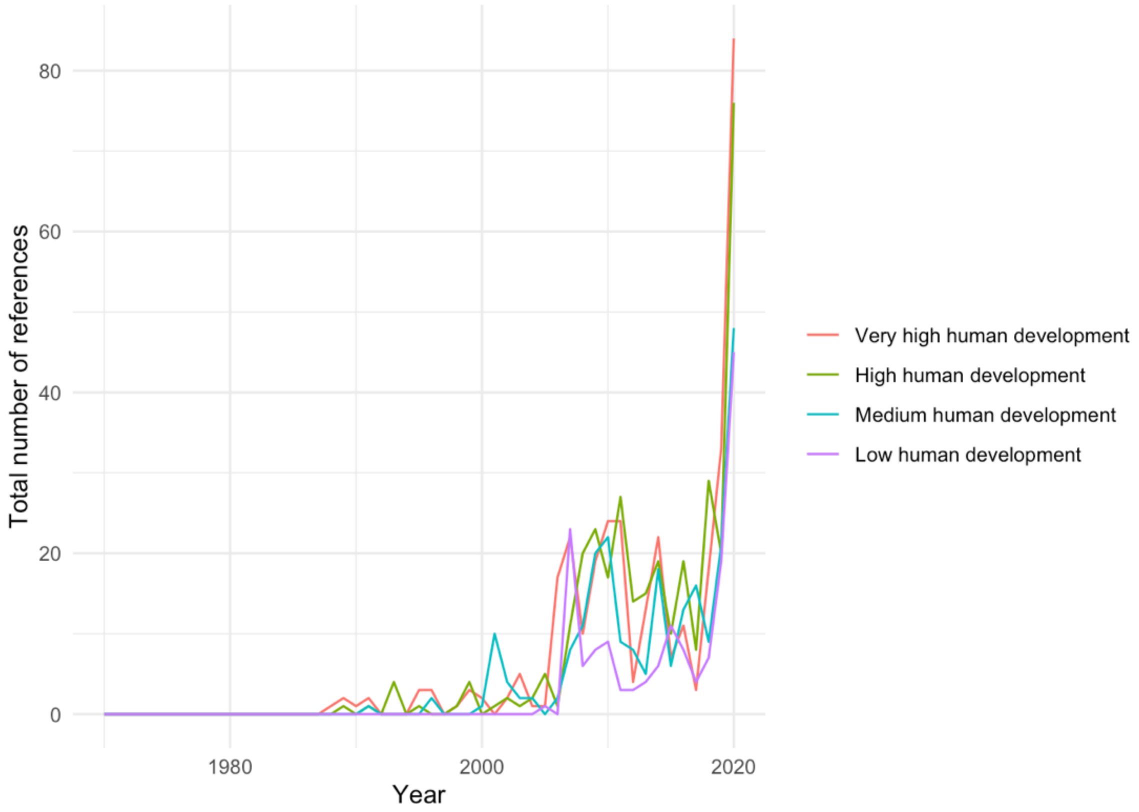

Figure 126. Total number of references to intersection by HDI categories, 1970-2020.

Figure 127 below presents a world map, which shows the countries that refer to the intersection of climate change and health in their 2020 UNGD statements, and the number of individual references they make. The map shows that almost half of all countries mentioned the intersection of health and climate change in their 2020 address. The map also shows that despite the higher engagement, there is still evidence of a divide between high-income countries on the one side, and low- and middle-income countries on the other side. The latter tend to engage more with climate change and health, particularly when the SIDS are included. Due to their size, the SIDS do not show up on the map. As has been noted, the SIDS tend to be highly represented among nations engaging with the health-climate change links.

Figure 128 and Figure 129 present world maps, which show the countries that refer to public health and climate change respectively in their 2020 UNGD statements, as well as indicating the number of references made by each country. The Figures demonstrate that there is considerable engagement with the issues of climate change and health separately. As noted in the main report, in 2020 for the first time all countries mentioned health in their UNGD statements, as can be seen in Figure 128. Figure 128 and Figure 129 show that as well as a much larger share of countries around the world discussing climate change and health in their GD statements compared to those discussing the intersection, there is also much deeper engagement with these two areas individually, in that countries tend to make a number of references to climate change and health in their GD statements.

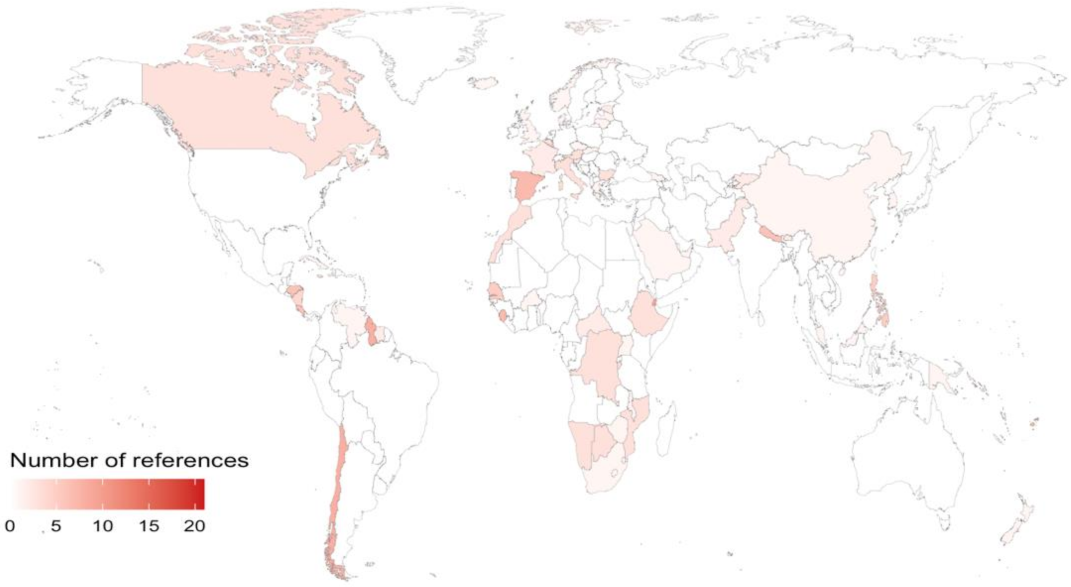

Figure 127. World map showing references to intersection of climate change and health, 2020.

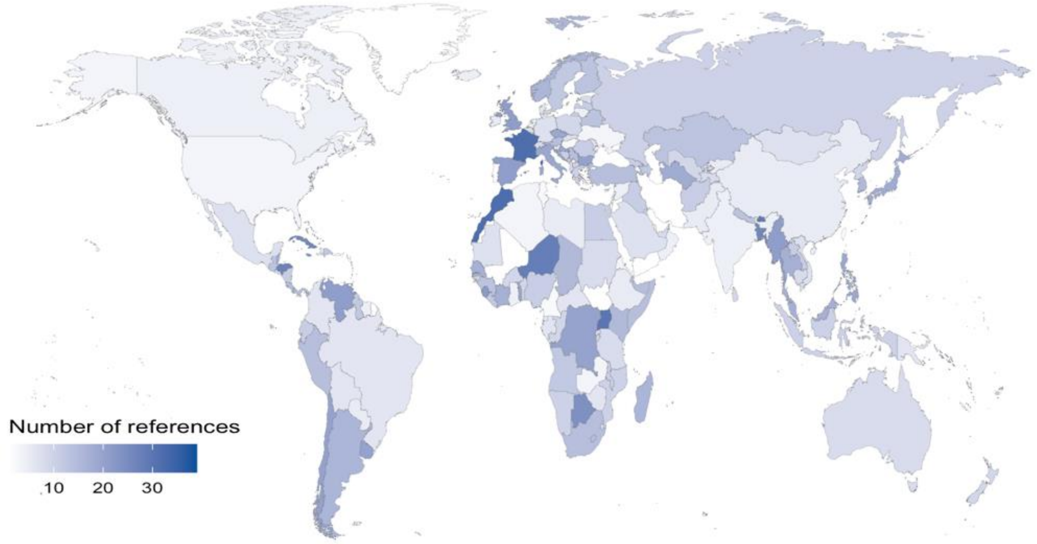

Figure 128. World map showing references to public health, 2020.

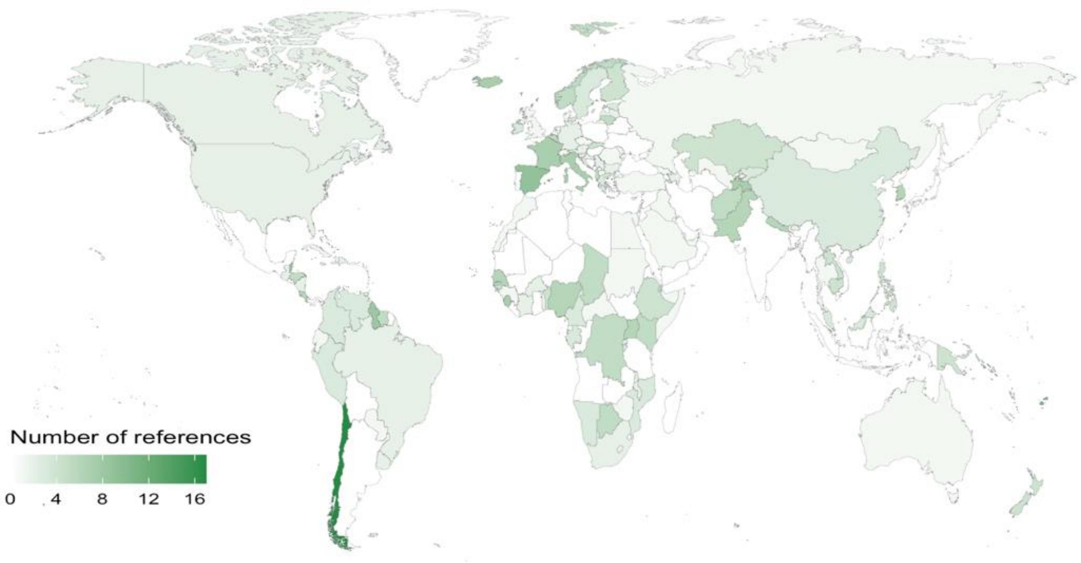

Figure 129. World map showing references to climate change, 2020.

The Figures below show engagement with climate change, health, and the intersection of climate change and health over 1970-2020 for selected countries.

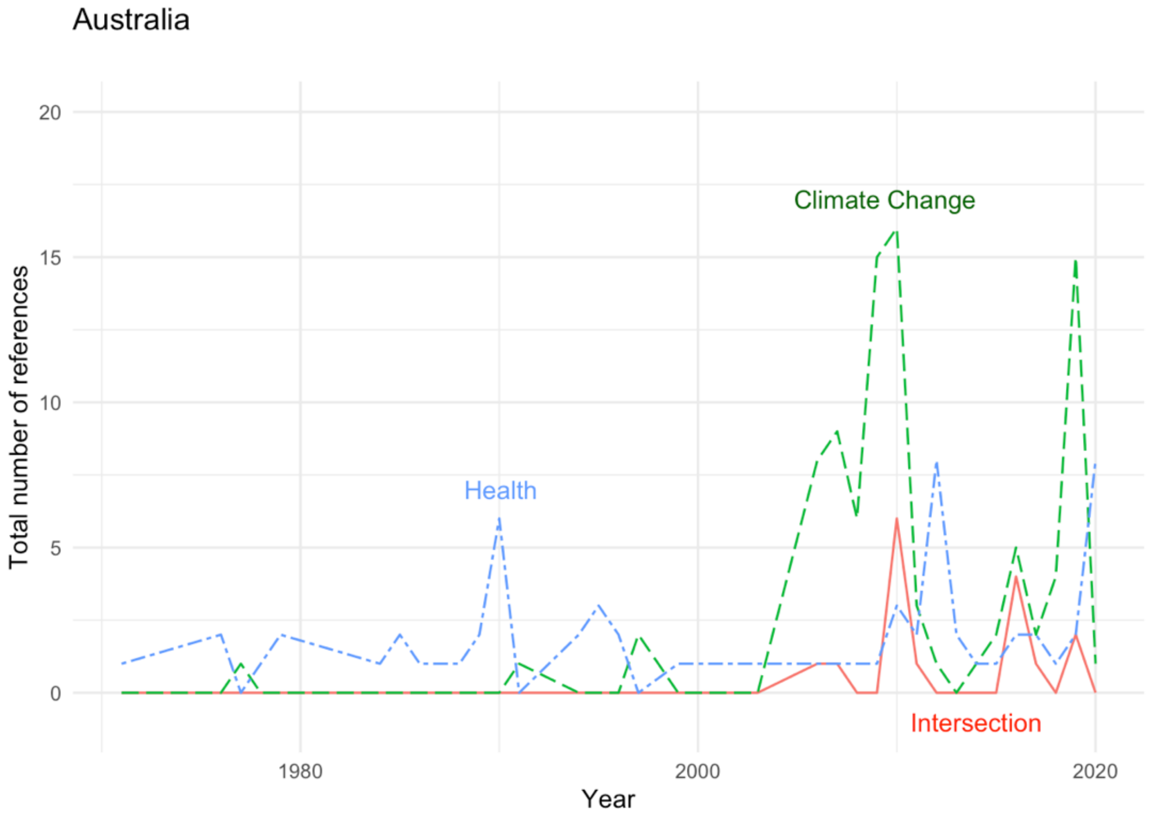

China

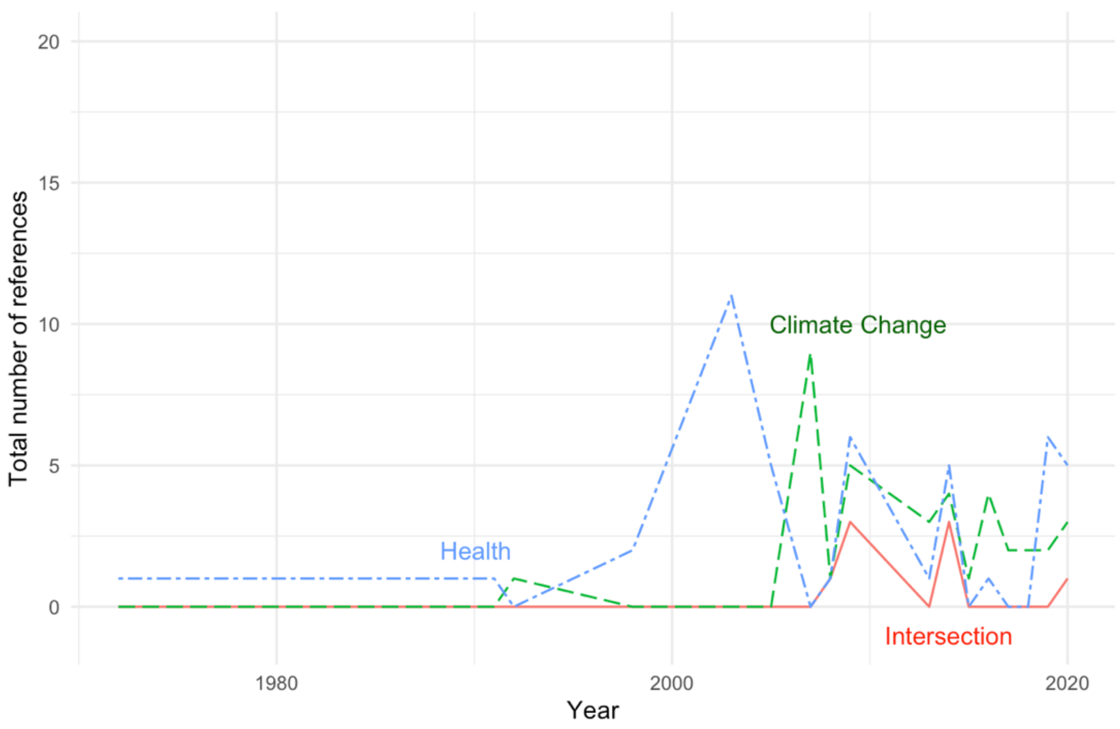

Germany

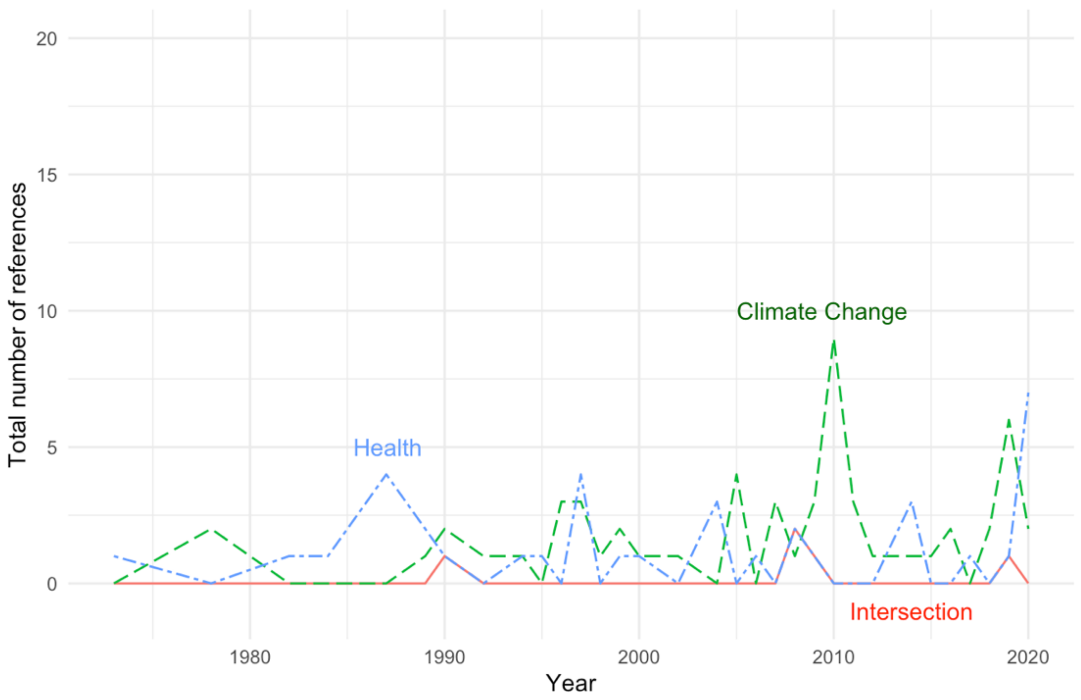

European Union

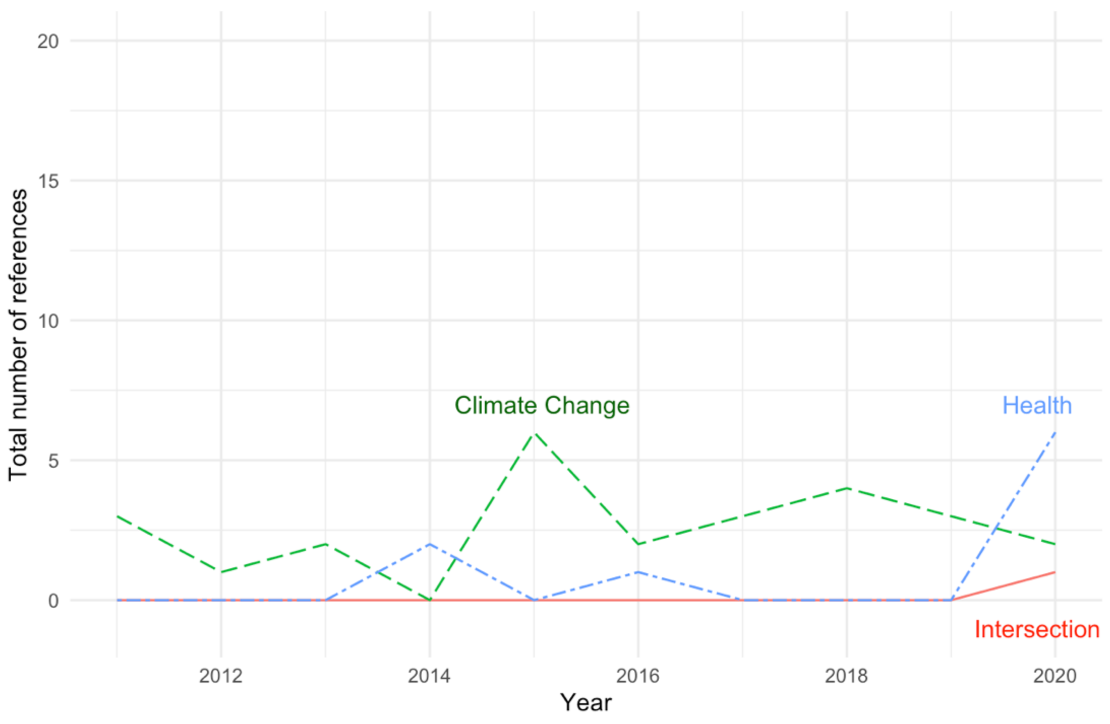

France

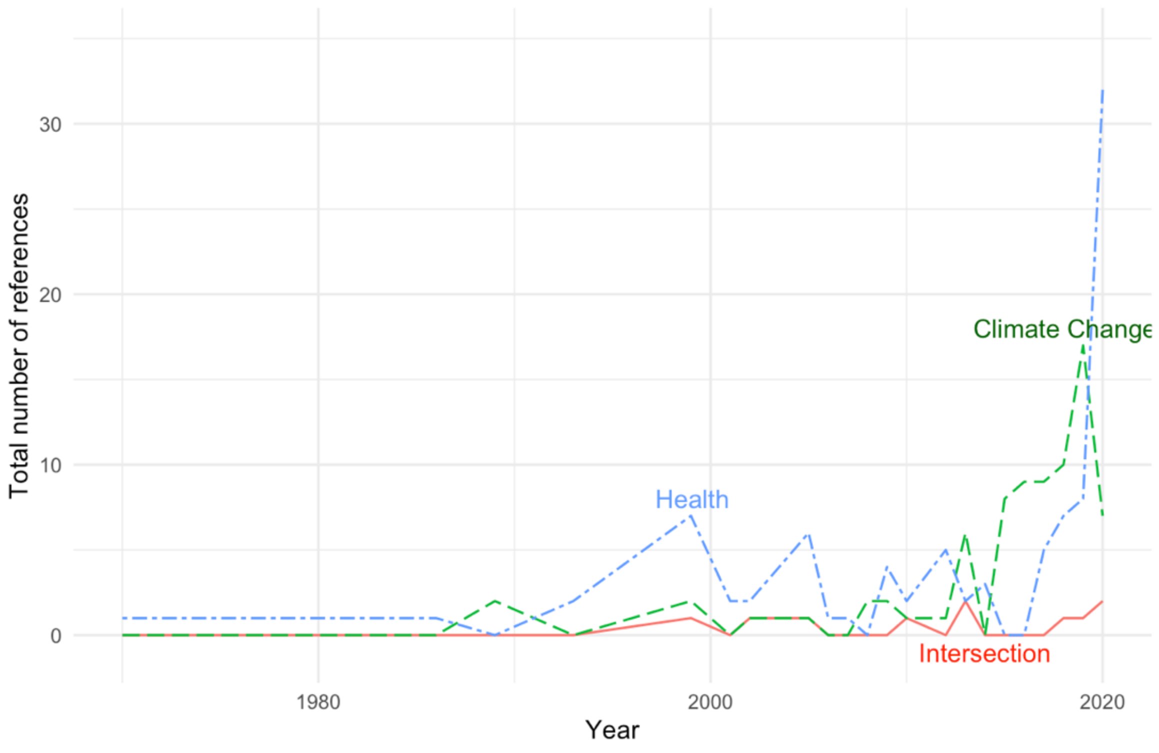

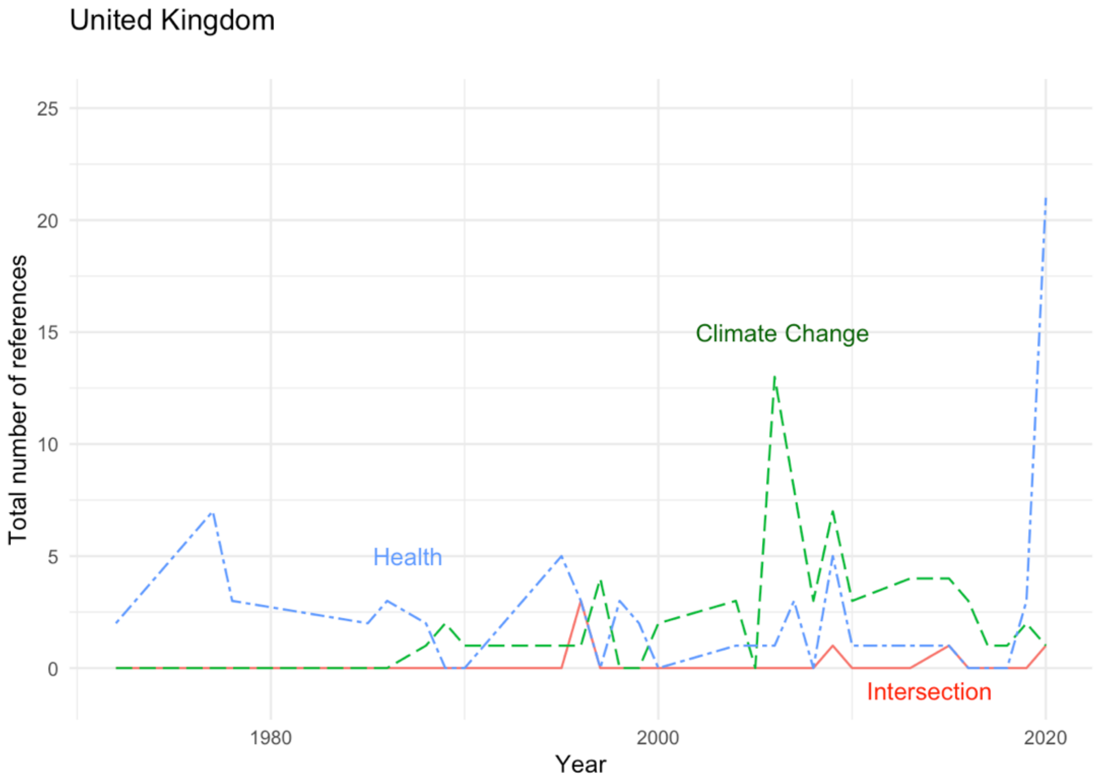
United Kingdom
Climate Change
Health
Intersection
Total number of references
Year
0
5
10
15
20
25
1980
2000
2020

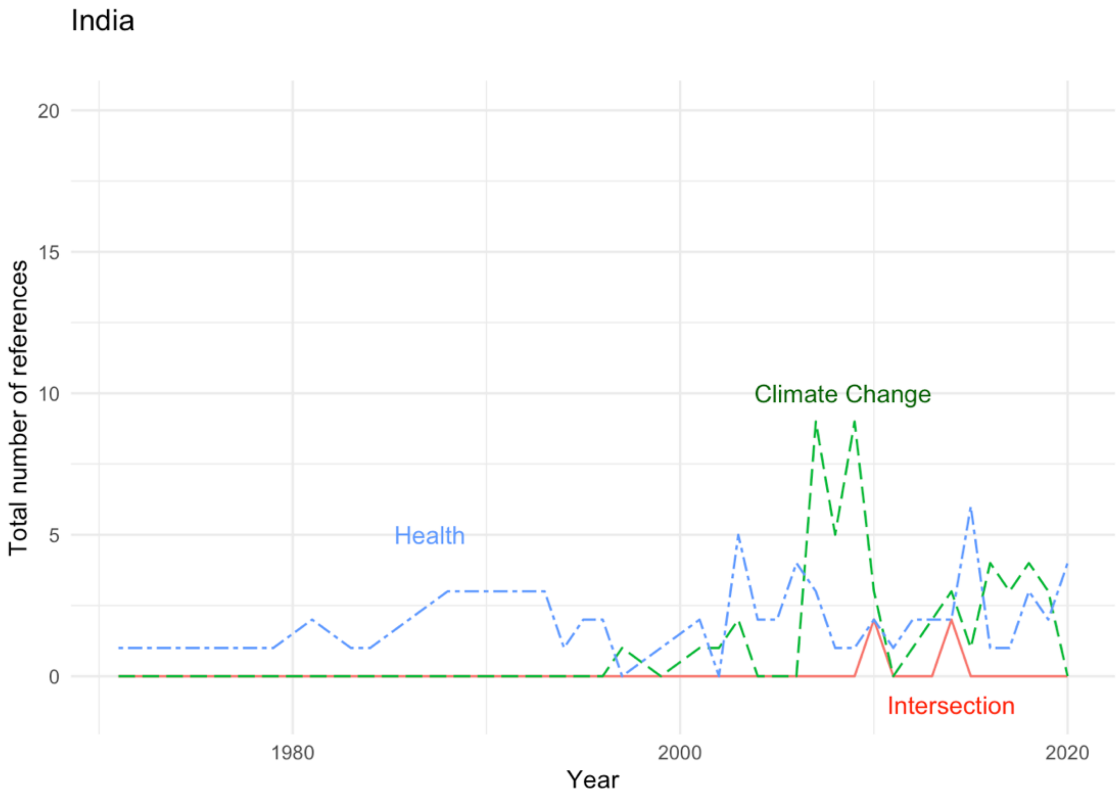
India
Climate Change
Health
Intersection
Total number of references
Year
0
5
10
15
20
1980
2000
2020

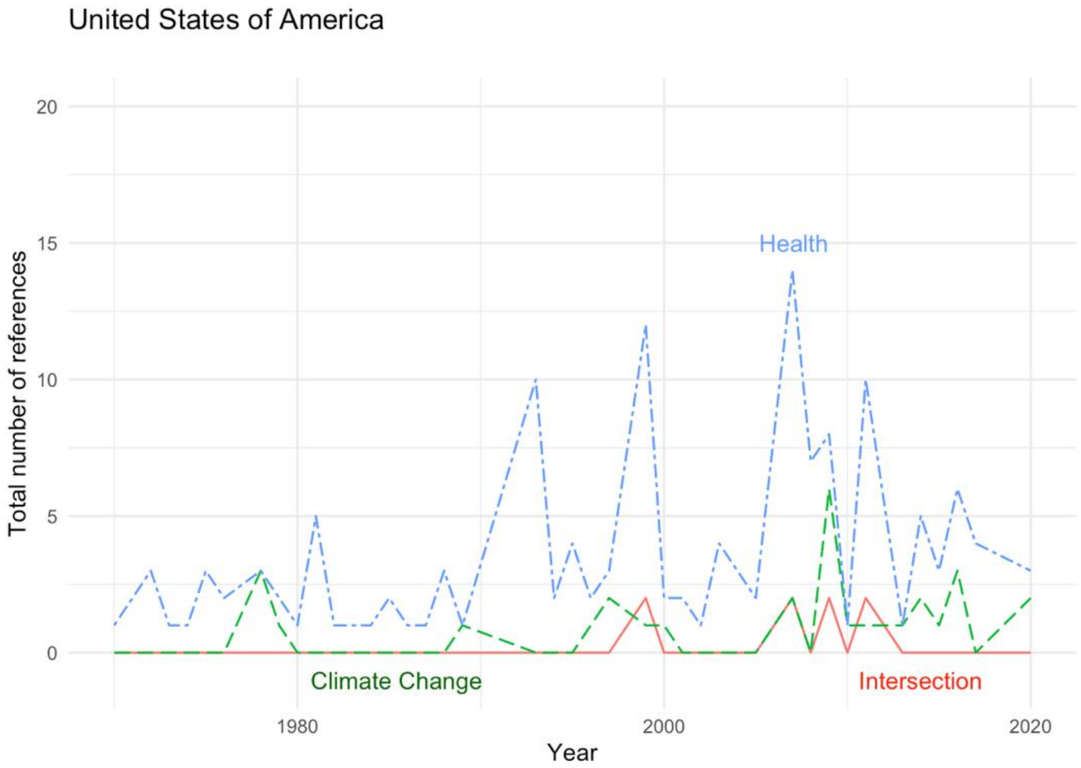
United States of America
Health
Climate Change
Intersection
Total number of references
Year
0
5
10
15
20
1980
2000
2020

Indicator 5.5: Corporate Engagement in Health and Climate Change in the Healthcare Sector

Methods

In order to produce the measure of engagement with climate change and health in companies' UN Global Compact Communication of Progress (GCCOP) reports, this indicator uses the publicly available GCCOP reports. This approach to using the GCCOP reports to produce the indicators is based on identifying references to key search terms linked to (a) health, and (b) climate change:

Health terms	Climate change terms
malaria<br>diarrhoea<br>infection<br>disease<br>diseases<br>sars<br>measles<br>pneumonia<br>epidemic<br>epidemics<br>pandemic<br>pandemics<br>epidemiology<br>healthcare<br>health<br>mortality<br>morbidity<br>nutrition<br>illness<br>illnesses<br>ncd<br>ncds<br>air pollution<br>nutrition<br>malnutrition<br>malnourishment<br>mental disorder<br>mental disorders<br>stunting	climate change<br>changing climate<br>climate emergency<br>climate action<br>climate crisis<br>climate decay<br>global warming<br>green house<br>temperature<br>extreme weather<br>global environmental change<br>climate variability<br>greenhouse<br>greenhouse-gas<br>low carbon<br>ghge<br>ghges<br>renewable energy<br>carbon emission<br>carbon emissions<br>carbon dioxide<br>carbon-dioxide<br>co2 emission<br>co2 emissions<br>climate pollutant<br>climate pollutants<br>decarbonization<br>decarbonisation<br>carbon neutral<br>carbon-neutral<br>carbon neutrality<br>climate neutrality<br>net-zero<br>net zero

These key terms from previous years have been updated to reflect the changing terminology used to discuss climate change. In order to produce an indicator of engagement with the intersection of climate change and health, the indicator focused on whether any of the climate change related terms appeared immediately before or after any public health terms in the GCCOP reports. This was based on a search of the 25 words before and after a reference to a public health related term.

Data

To produce this indicator, on the indicator draws on the publicly available UN GCCOP reports. A total of 51,344 reports were downloaded from GCCOP. The reports are available for companies based in 129 countries.

GCCOP reports are submitted in 30 different languages. For the development of this indicator, the focus included only convertible reports available in English (17,984), or around 35 per cent of the total number of UN GCCOP reports. A number of the English language files were corrupt or could not be converted into plain text format for analysis. The distribution of available English-language reports over time is presented in Table 53:

Table 53. English -language GCCOP reports by year.

Year	Number of reports
2011	1021
2012	1374
2013	1553
2014	1686
2015	1771
2016	1928
2017	1972
2018	2000
2019	2197
2020	2029

There are only single GCCOP report submissions before 2011, thus the sample of GCCOP reports is limited to the period 2011-2020. These documents were pre-processed and prepared for the application of natural language processing by converting the reports to plain text format; removing punctuation and numbers; removing stopwords; regularising (lowercasing); and stemming. All pre-processing and analysis was carried out in R using the “quanteda” package.[281]

Caveats

As noted above, only GCCOP reports that were submitted in English were included. This means a little under half of all available UN General Compact COP reports were analysed.

This analysis here is based on a narrow range of search terms, which excludes reference to many of indirect links between climate change and health. Reports may also discuss indirect connections, such as the effect of climate change on agriculture, however, these are not included here. Therefore, the results present a somewhat conservative estimate of high corporate engagement with the intersection of climate change and health. Future work in this area will consider engagement with these indirect links, as well as providing additional forms of analysis.

Future form of the indicator

In the future, the aim is to increase the number of reports analysed by translating key search terms into several other key languages, and incorporating reports submitted in languages other than English into the sample. Translation of key terms has been implemented in WG5 into Spanish, Portuguese, and German.

Additional analysis

Additional findings and breakdowns are presented in this section. Figure 130 presents the total number of references to climate change, health, and the intersection of climate change and health across for the GCCOP reports. Despite the increase in the proportion of companies engaging with the climate change-health linkages, the overall number of references remains fairly low and consistent, relative to the individual references to health and climate change.

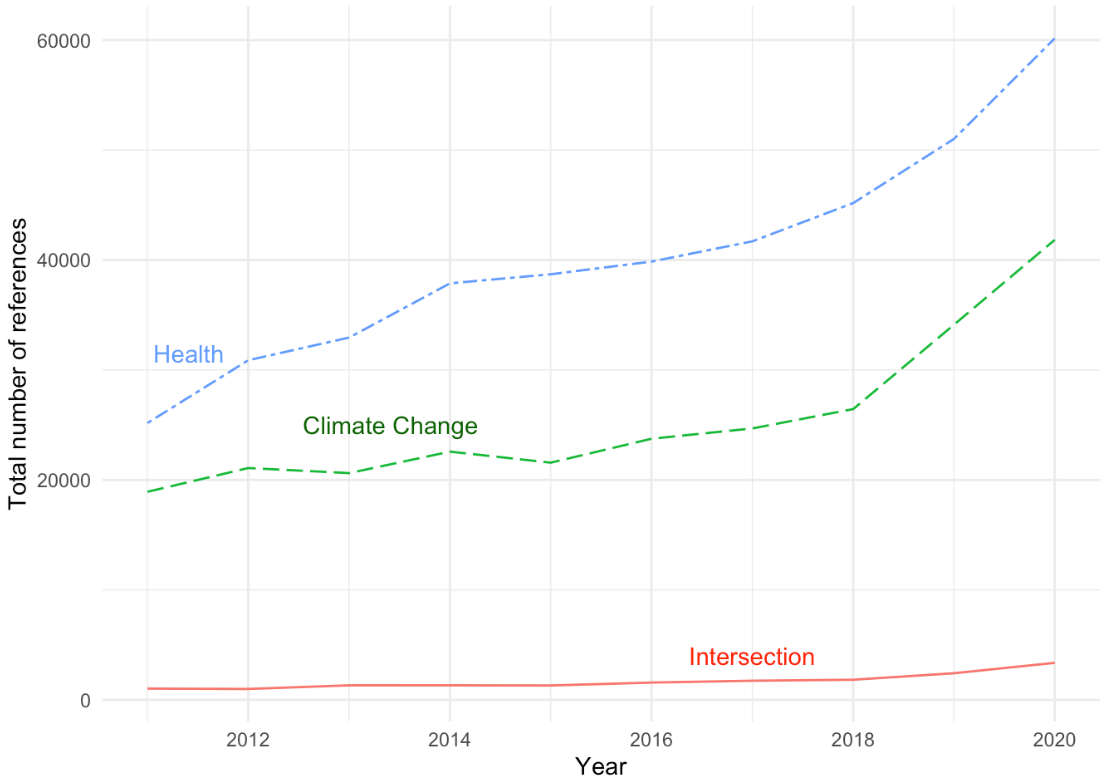

Figure 130. Total references to climate change, health, and the intersection of climate change and health, 2011-2020.

In Figure 131, below, the total references with the intersection of climate change and health are presented to better show any trends occurring in engagement. The Figure shows that since 2018 there has been a sharp rise in the number of references.

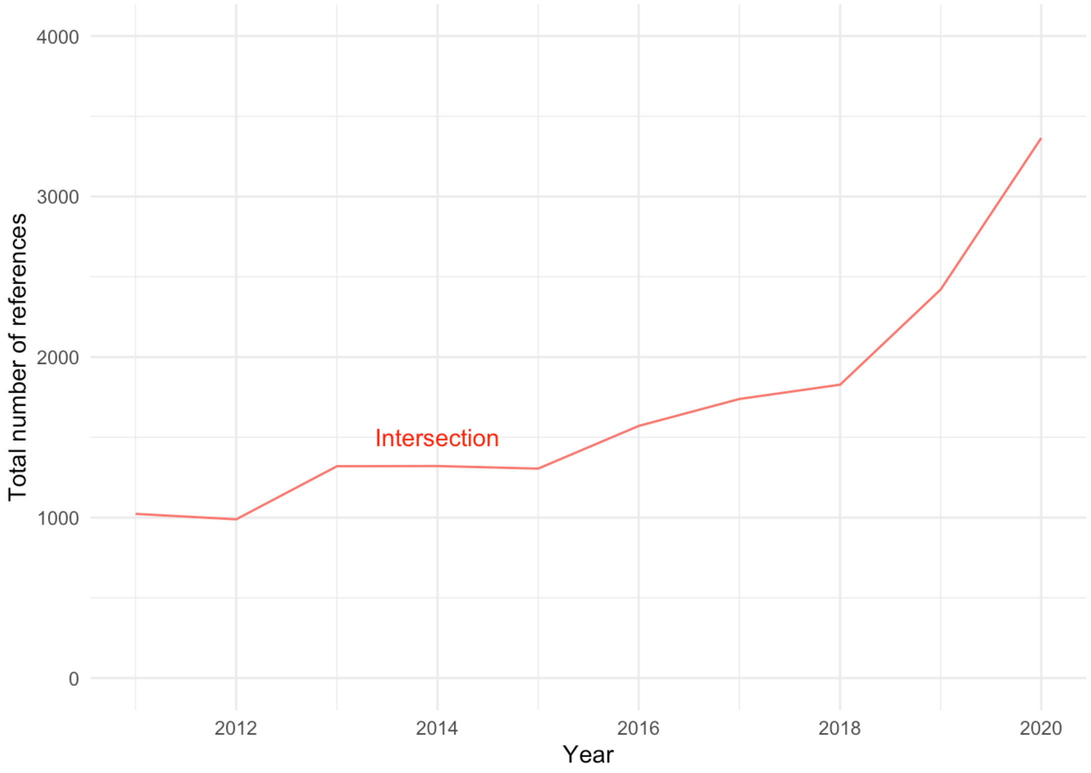

Figure 131. Total references to the intersection of climate change and health, 2011-2020.

Figure 132 shows the average number of references to climate change, health, and the intersection in GCCOP reports. The Figure again demonstrates the relatively low level of engagement with the health impacts of climate change in GCCOP reports, compared to the separate references to health and climate change.

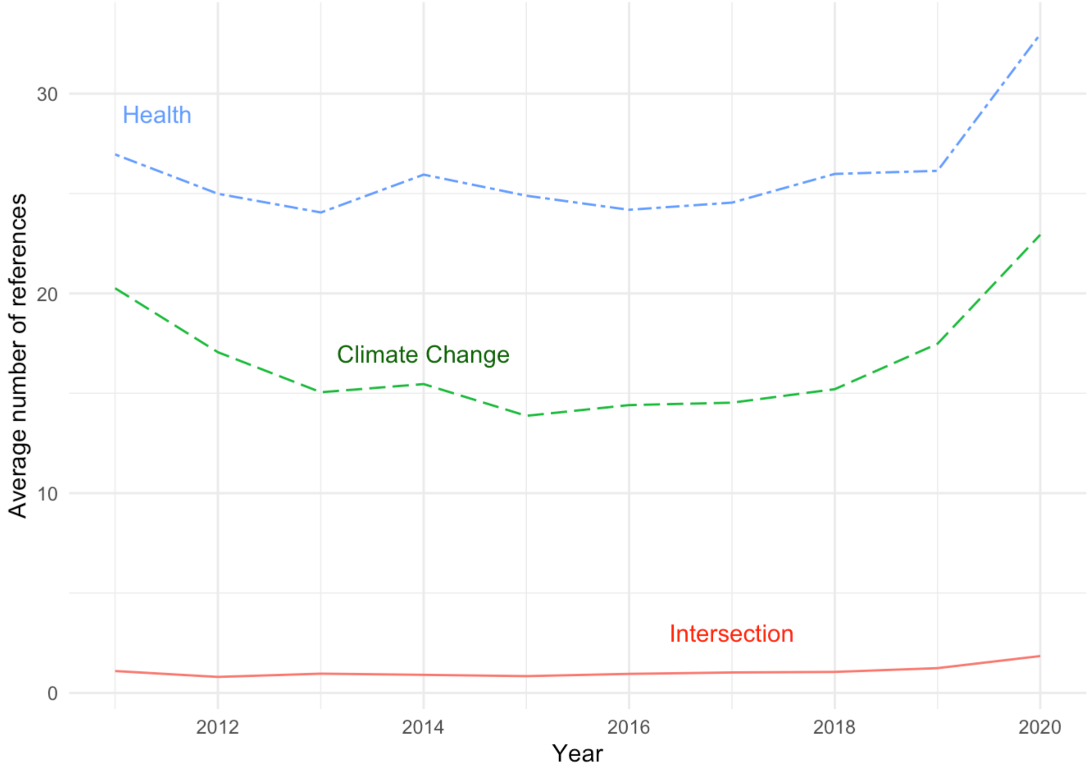

Figure 132. Average references to climate change, health, and the intersection of climate change and health in GCCOP reports, 2011-2020.

There is growing awareness of the gendered impacts of climate change and health. The indicator therefore considers the extent to which references to the health dimensions of climate change in companies' UN GCCOP reports engage with gender issues. This is achieved by further examining the references to the intersection of climate change and health. Once all references to this intersection in GCCOP reports for 2011-2020 were identified, additional search terms related to gender were used to identify which of the intersection references also engaged with gender issues. The gender-related search terms used were as follows: *women, women's, maternal, inequality, inequalities, gender, empowerment, sex, sexual, violence, violent, girls, reproduction, reproductive*. Hence, the analysis considers whether the 25 words of text identified in the primary search (for climate change and health terms) includes a reference to at least one of these gender-related keywords.

Based on the additional search of the references to the climate change-health intersection using these gender-related keywords, references to the health dimensions of climate change with a gender focus in companies' annual COP reports were identified. Figure 133 presents annual references to the gender dimensions of climate change and health in UN Global Compact COP reports between 2011 and 2020. The Figure shows a steady increase in engagement between 2014 and 2018. In 2019, there was a sharp rise, with 19% of all references to the intersection of climate change and health including a mention of one of the gender keywords. However, engagement with gender fell in 2020 to 13%.

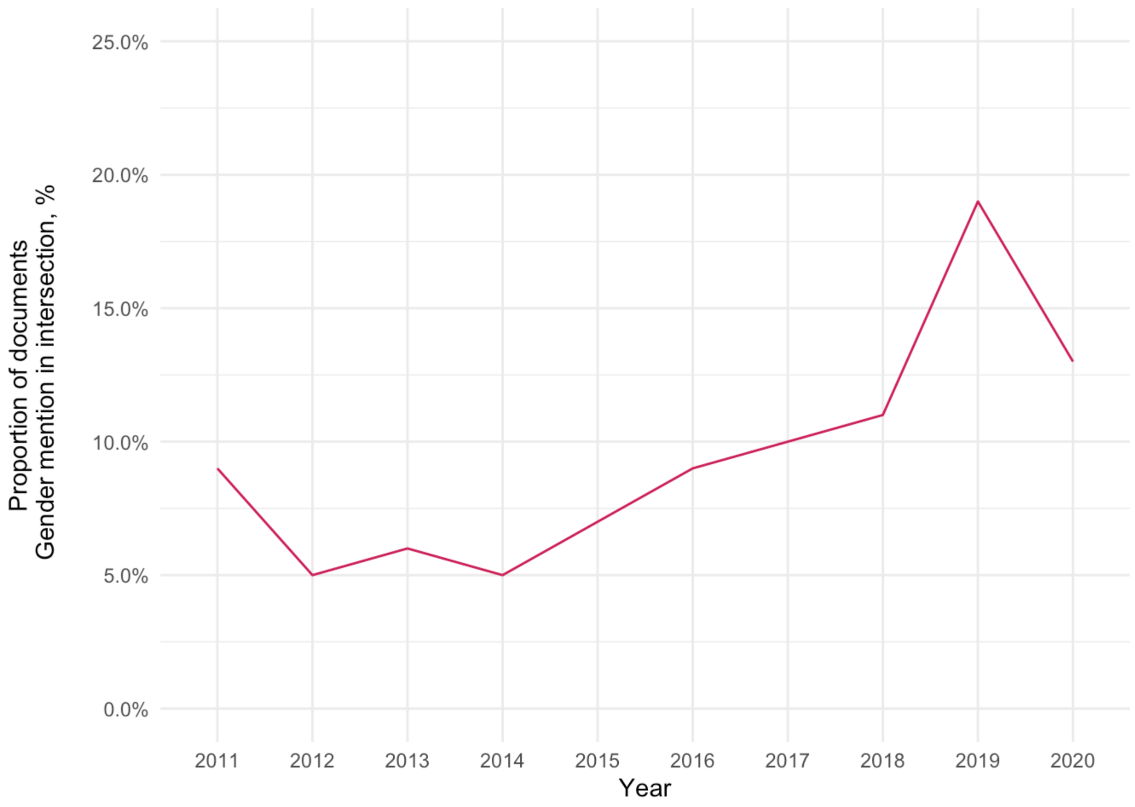

Figure 133. Proportion of references to intersection of health and climate change in COP reports that include a reference to gender, 2011-2020.

Also considered was engagement with climate change and health in the UN Global Compact COP reports by WHO region. Figure 134 shows the total number of references to the climate change-health intersection based on which of the WHO regions a company is based on, and Figure 135 shows the proportion of companies based in the different WHO regions that refer to the health impacts of climate change in their annual COP report.

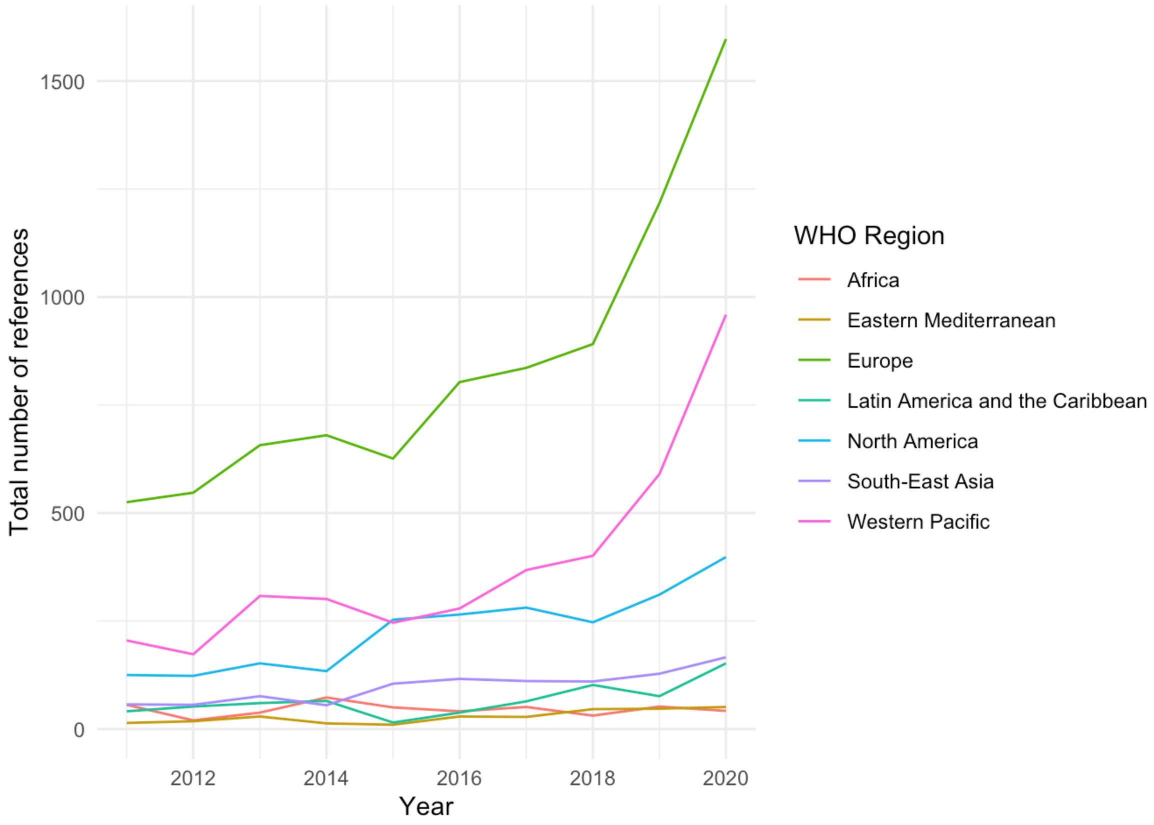

Figure 134. Total references with the intersection of climate change and health by WHO region, 2011-2020.

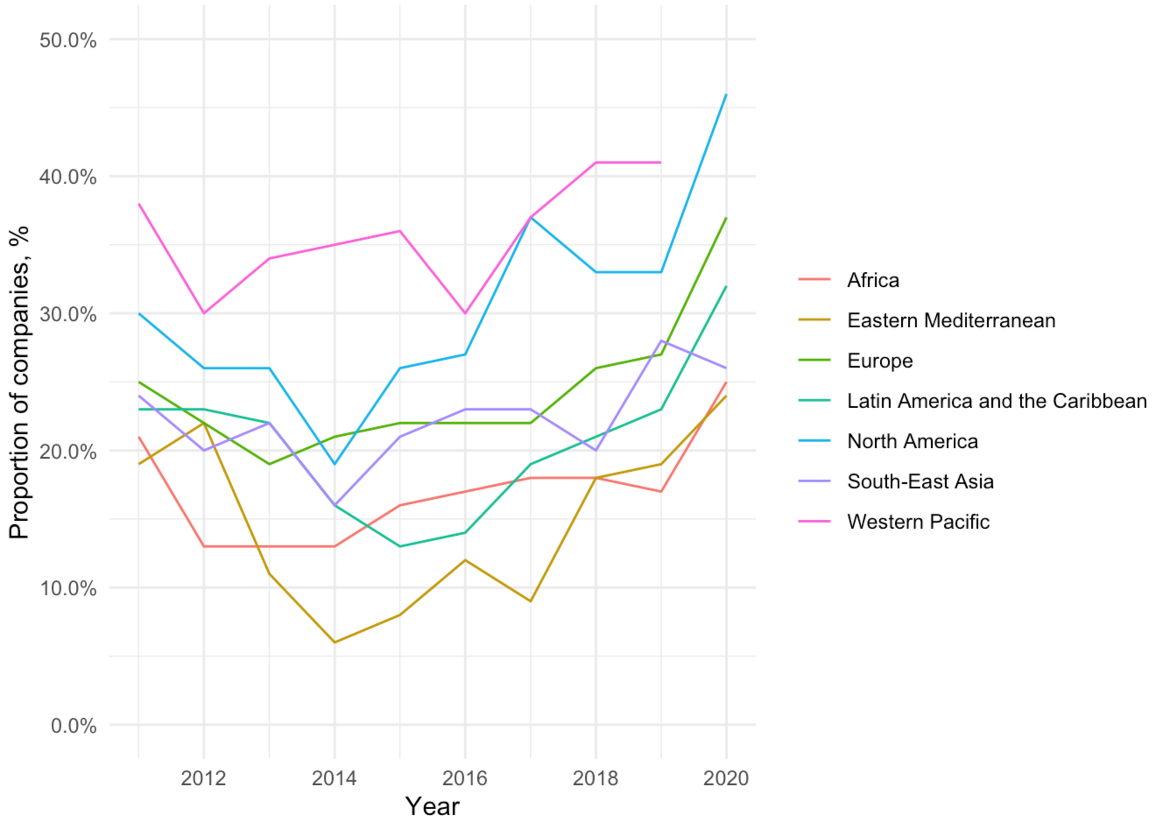

Figure 135. Proportion of companies referring to intersection of health and climate change by WHO region, 2011-2020.

Figure 134 and Figure 135 shows that the highest proportion of COP reports engaging with the climate change-health intersection in recent years has come from corporations based in Europe, North America, and Africa. The indicator finds that the lowest engagement comes from corporations based in the Eastern Mediterranean region.

Engagement across different sectors was also considered. Table 54 shows the total number of references to climate change, health, and the intersection across the different sectors in 2020. Figure 136 presents the proportion of corporations engaging with the climate change-health relations in each sector in 2020.

Table 54. Total number of references to the intersection of climate change and health by sector in 2020.

	**Health**	**Climate change**	**Intersection**
**Aerospace & Defense**	754	403	46
**Alternative Energy**	551	866	92
**Automobiles & Parts**	2032	2160	105
**Banks**	1079	1484	84
**Beverages**	927	562	37
**Chemicals**	3438	2078	191

**Construction & Materials**	3357	2699	192
**Diversified**	1789	1024	93
**Electricity**	1184	2041	77
**Electronic & Electrical Equ...**	1605	822	64
**Equity Investment Instruments**	80	99	12
**Financial Services**	3217	4243	210
**Fixed Line Telecommunications**	327	195	24
**Food & Drug Retailers**	244	195	16
**Food Producers**	2907	1282	202
**Forestry & Paper**	367	580	22
**Gas, Water & Multiutilities**	921	821	91
**General Industrials**	4532	2836	274
**General Retailers**	1470	1216	40
**Health Care Equipment & Ser...**	1751	356	45
**Household Goods & Home Cons...**	1072	1022	48
**Industrial Engineering**	1158	767	82
**Industrial Goods & Services**	12	4	0
**Industrial Metals & Mining**	1358	789	74
**Industrial Transportation**	1275	819	51

**Leisure Goods**	136	63	2
**Life Insurance**	651	330	41
**Media**	680	454	19
**Mining**	1150	594	55
**Mobile Telecommunications**	925	629	61
**Nonequity Investment Instru...**	112	37	3
**Nonlife Insurance**	413	260	23
**Oil & Gas Producers**	1603	1490	114
**Oil Equipment, Services & D...**	594	435	24
**Personal Goods**	806	504	22
**Pharmaceuticals & Biotechno...**	6336	1125	280
**Real Estate Investment & Se...**	1638	1289	103
**Real Estate Investment Trusts**	232	241	24
**Software & Computer Services**	1404	1098	106
**Support Services**	3662	2174	200
**Technology Hardware & Equip...**	1632	1131	99
**Travel & Leisure**	744	48	17

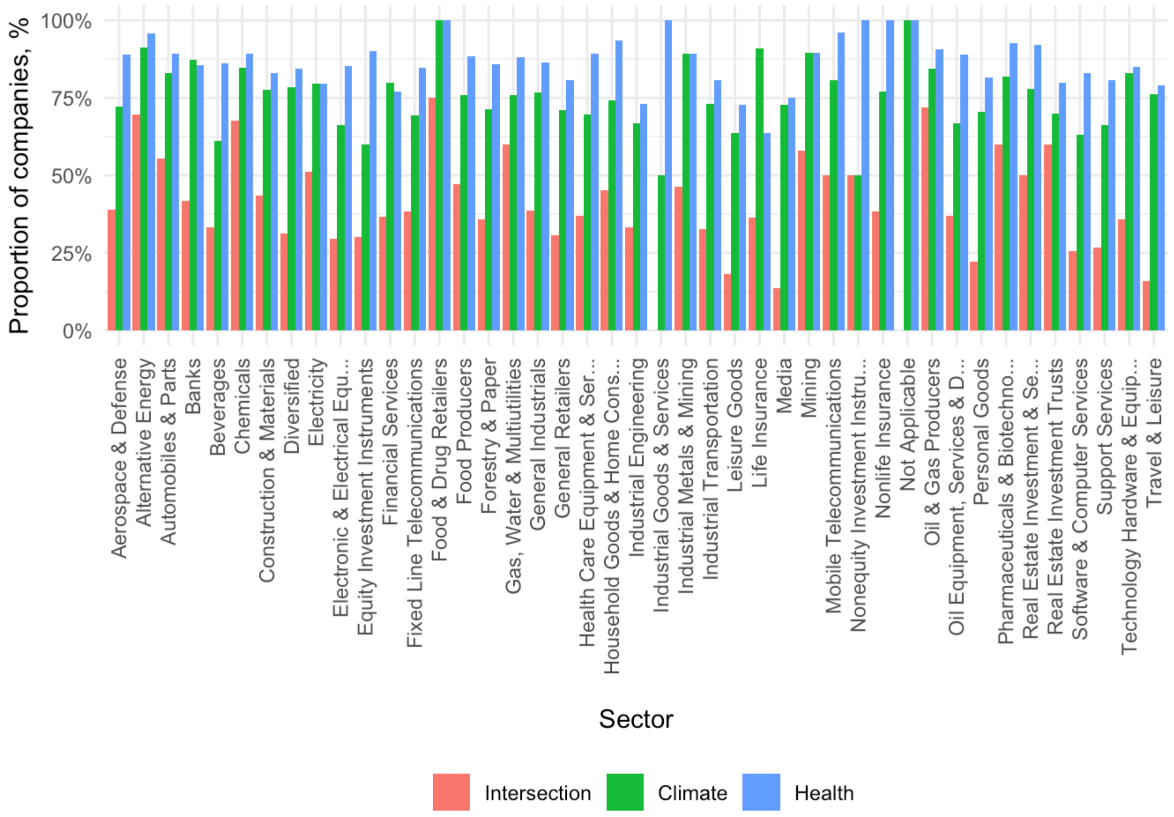

Figure 136. Proportion of corporations referring to climate change, health, and the intersection of climate change and health by sector in 2020.

As discussed in the main report, the highest level of engagement with the intersection of climate change and health in 2020 can be seen in the food and drug retailers, oil and gas producers, and alternative energy sectors. In these sectors, more than 70% of companies make reference to the health dimensions of climate change. In contrast, we surprisingly see much lower levels of engagement in the healthcare sector, where only 37% of companies refer to the intersection of health and climate change in their 2020 GCCOP report.

In addition to looking at companies by WHO region, the indicator also considers companies from different types of countries in terms of their potential importance and role in addressing issues related to climate change. This is provided in Figure 137 and Figure 138. As noted in previous years' reports, the SIDS have driven much of the engagement with the health impacts of climate change, as well as climate change more generally, in the UN General Assembly. As such, a SIDS grouping is included. Arguably the three most important countries/unions in addressing climate change are USA, China, and the EU. These are referred to as Tier 1 countries in Figure 137 and Figure 138. Finally, the indicator also considers an additional grouping of countries that are also important in terms of their CO_2 emissions, their influence in international politics, and their potential impact on addressing climate change. This grouping, which is referred to as Tier 2 countries includes: Poland, Australia, South Africa, Brazil, India, France, Germany, and Indonesia. Hence, companies are looked at based on the type of country in which they are based in Figure 137 (total references) and Figure 138 (proportion of companies). The results in Figure 137 show that the highest total references to the intersection of climate change and health tends to come from companies based in Tier 2 countries, and the lowest from those based in the SIDS. However, this is likely to reflect the vastly different numbers of companies that have signed up to the UN Global Compact from these regions. Figure 138 shows that in terms of the proportion of the companies that engage with health and climate change, the highest engagement is seen from companies based in the SIDS, followed by those based in Tier 1 countries, with companies based in Tier 2 countries having the lowest engagement.

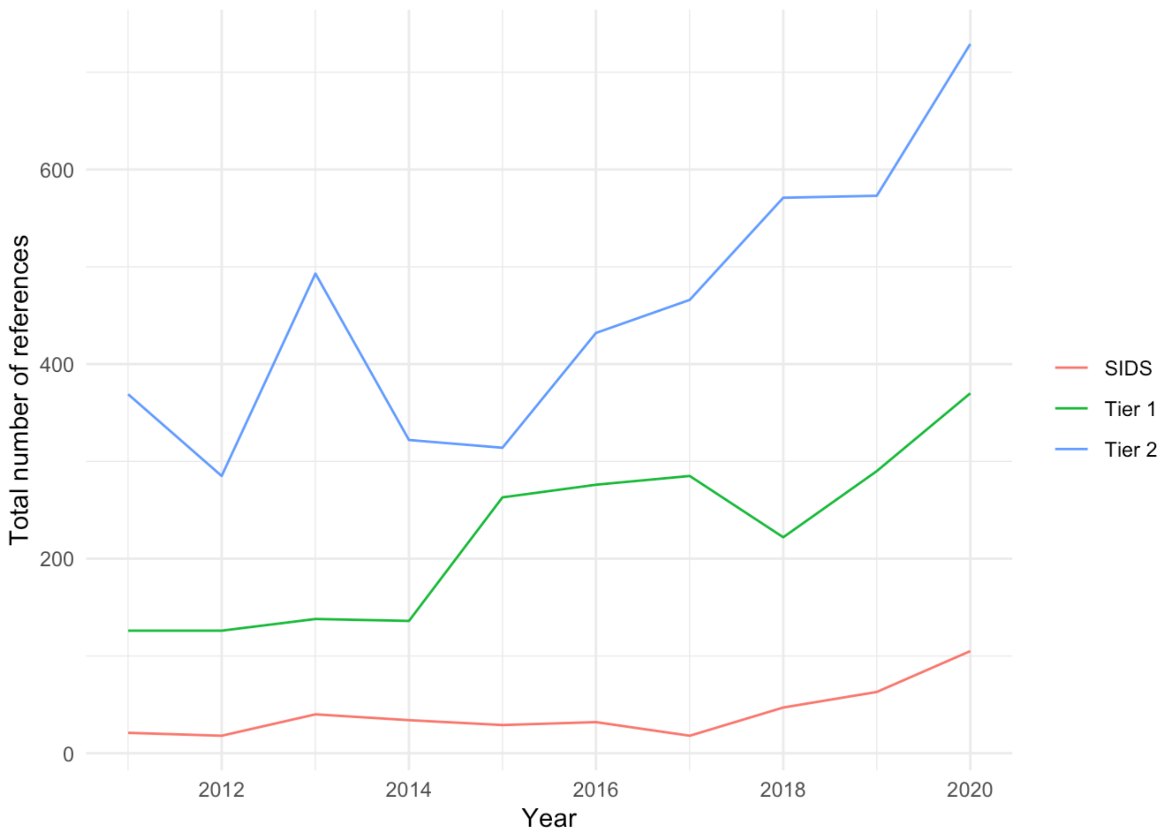

Figure 137. Total references to the climate change-health intersection by SIDS, Tier 1 countries, and Tier 2 countries, 2011-2020.

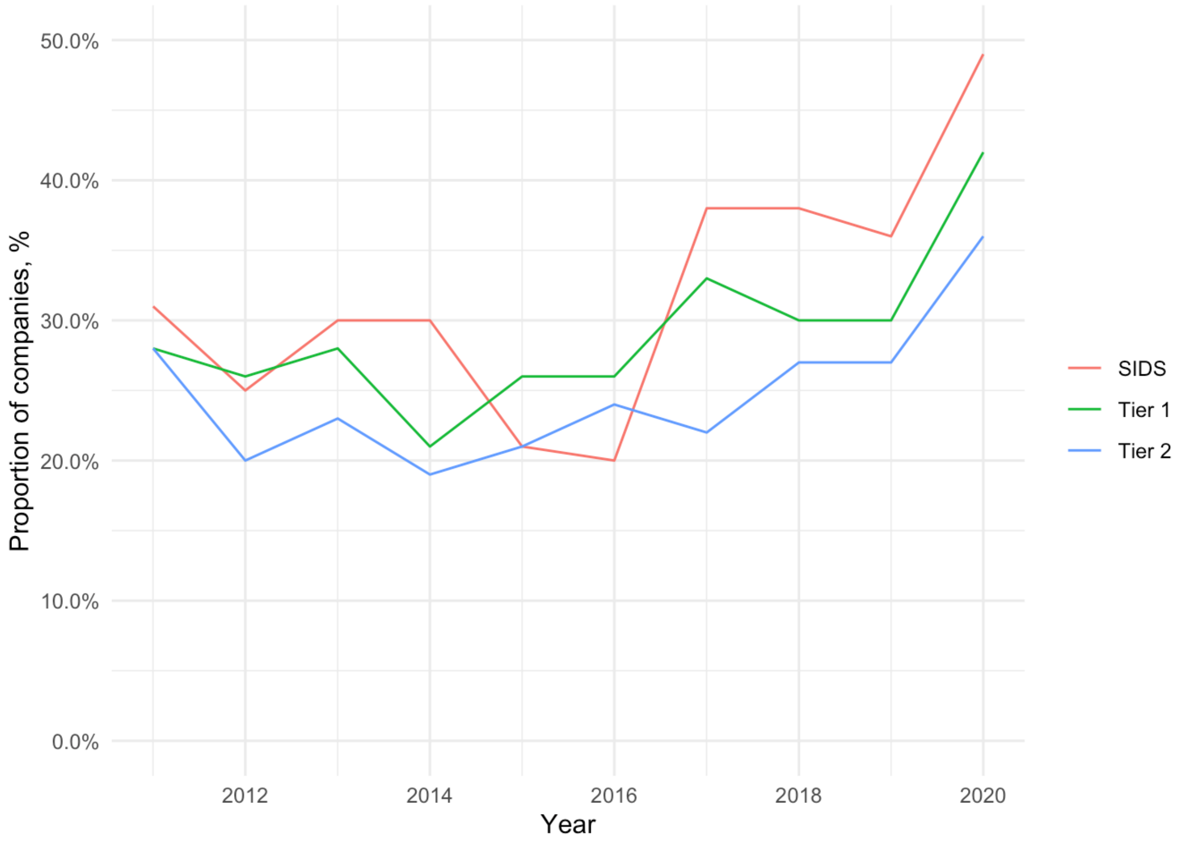

Figure 138. Proportion of corporations referring to the climate change-health intersection by SIDS, Tier 1 countries, and Tier 2 countries, 2011-2020.

The indicator also considers corporate engagement with the health dimensions of climate according to the Human Development Index (HDI) categories of the countries in which companies are based. Figure 139 shows the total references to the intersection of climate change and health in companies' 2020 COP reports based on the country HDI category and Figure 140 shows the proportion of companies engaging with climate change and health in their 2020 COP report by HDI category. Figure 139 shows significantly higher references to climate change and health made by countries based in countries that have very high human development compared to companies based in countries with other levels of human development. However, this reflects the fact that the majority of companies included in our analysis are based in countries with very high human development levels. It is worth noting that even when considering the proportion of companies that engage with climate change and health (Figure 140), it is the companies based in countries with very high human development that have highest engagement, followed by those with a high HDI. Lower engagement with climate change and health is observable by companies based in countries with low and medium human development levels.

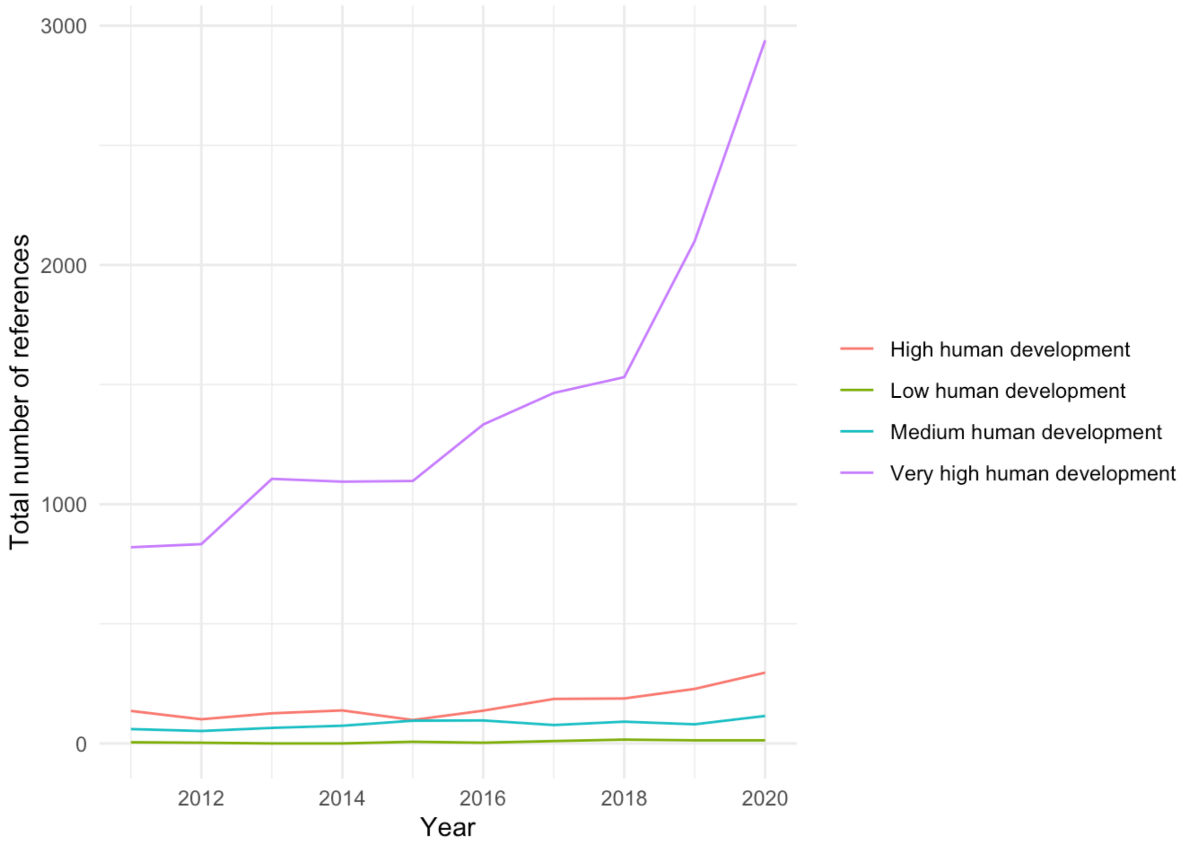

Figure 139. Total references to the climate change-health intersection by country HDI categories, 2011-2020.

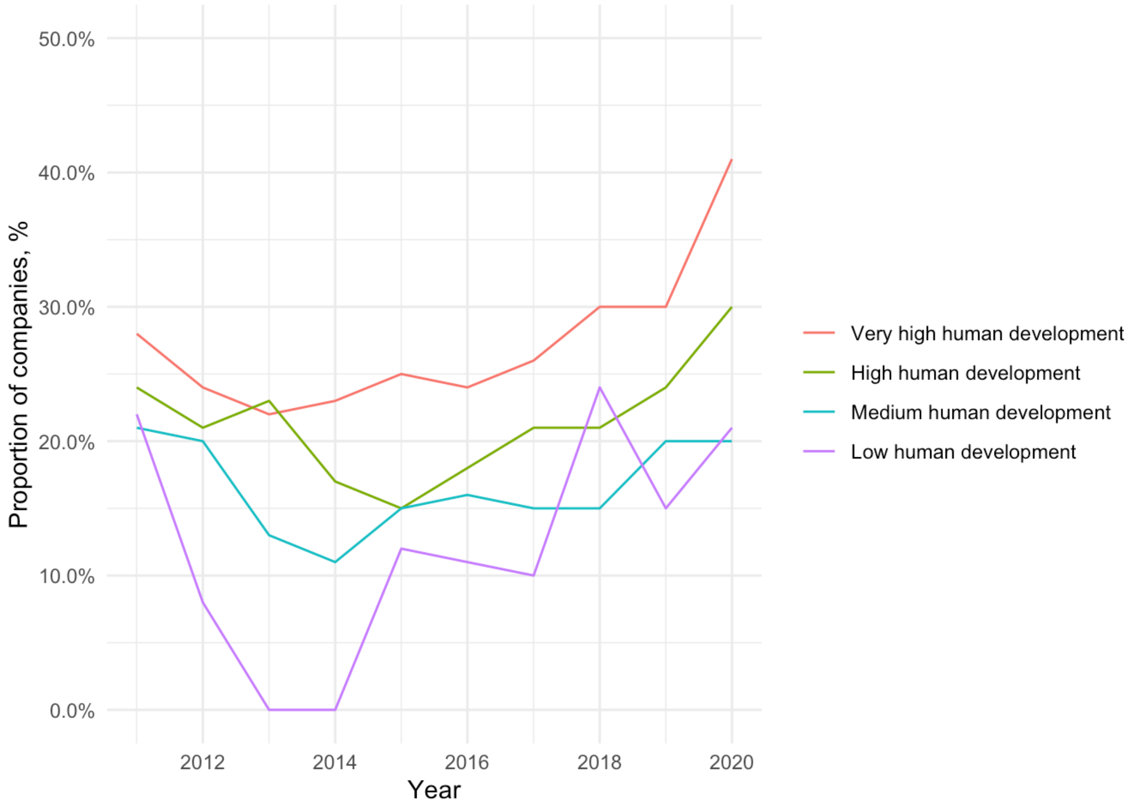

Figure 140. Proportion of corporations referring to the climate change-health intersection by country HDI categories, 2011-2020.

In the 2019 and 2020 reports, the analysis of corporate sector engagement with climate change and health focused on those companies based in the healthcare sector. In this year's report, the focus is instead on all companies that have signed up to the UN Global Compact and submitted COP reports. Additional information is provided, focusing only on companies in the healthcare sector below. As has been noted previously, engagement with the health dimensions of climate change by healthcare companies is mid-level compared to other sectors. Figure 141 shows the proportion of healthcare sector companies referring to climate change, change, health, and the intersection; Figure 142 shows the total references; and Figure 143 shows the average references per company in the healthcare sector. As would be expected the Figures show high levels of engagement with health in the COP reports of healthcare sector companies. There is also considerable engagement with climate change. However, engagement with the intersection of climate change and health remains relatively low, despite a slight rise in engagement in recent years.

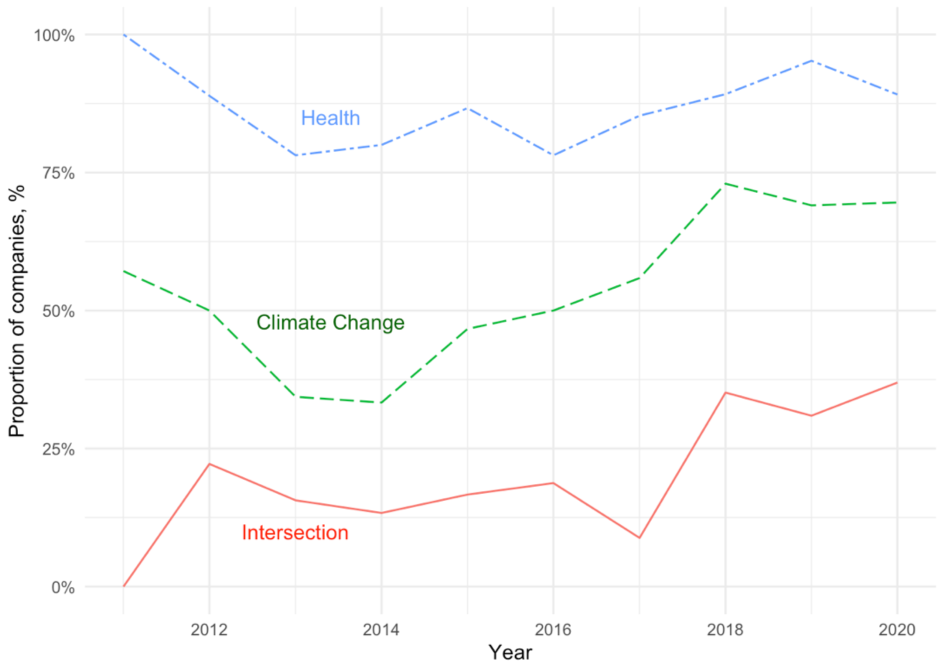

Figure 141. Proportion of healthcare sector companies referring to climate change, health, and the intersection of health and climate change in COP reports, 2011-2020

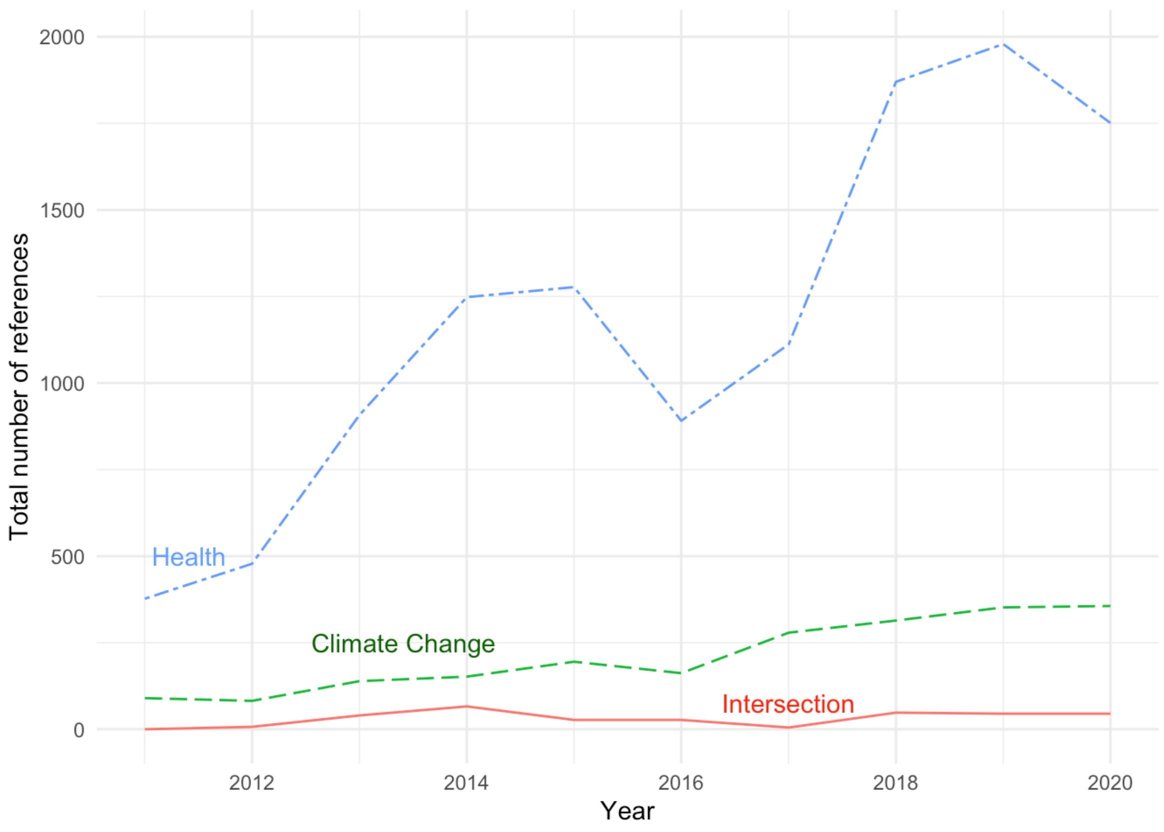

Figure 142. Total references to climate change, health, and the intersection of climate change and health in healthcare sector, 2011-2020.

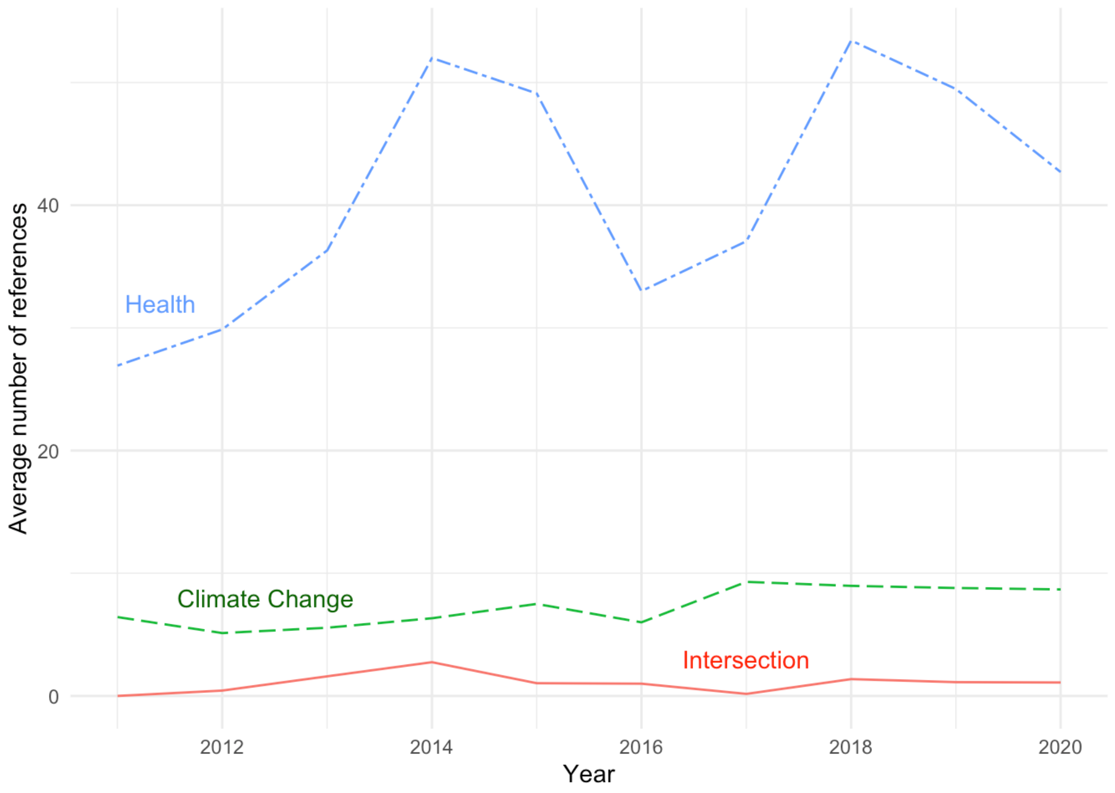

Figure 143. Average references to climate change, health, and the intersection of climate change and health in the healthcare sector COP reports, 2011-2020

References

1. Watts N, Amann M, Arnell N, et al. The 2020 report of The Lancet Countdown on health and climate change: responding to converging crises. *The Lancet* 2021; **397**(10269): 129-70.
2. Collins M, Knutti R, Arblaster J, et al. Long-term Climate Change: Projections, Commitments and Irreversibility. *Climate Change 2013: The Physical Science Basis* 2014: 1029-136.
3. Kirtman B, Power SB, Adedoyin AJ, et al. Near-term Climate Change: Projections and Predictability. *Climate Change 2013: The Physical Science Basis* 2014: 953-1028.
4. Copernicus Climate Change Service (C3S). ERA5 monthly averaged data on single levels from 1979 to present. Available at https://doi.org/10.24381/cds.f17050d7. 2021.
5. NASA Socioeconomic Data and Applications Center (SEDAC) Gridded Population of the World (GPWv4). Available at https://beta.sedac.ciesin.columbia.edu/data/collection/gpw-v4. 2021.
6. The Inter-Sectoral Impact Model Intercomparison Project (ISIMP). Input data set: Historical, gridded population. Available at https://www.isimip.org/gettingstarted/input-data-bias-correction/details/31/. 2021.
7. Copernicus Climate Change Service (C3S). Surface air temperature for August 2020. Available at https://climate.copernicus.eu/surface-air-temperature-august-2020. 2021.
8. Global Burden of Disease Collaborative Network. Global Burden of Disease Study 2019 (GBD 2019) Population Estimates 1950-2019. Seattle, United States of America: Institute for Health Metrics and Evaluation (IHME), 2020.
9. Global Burden of Disease Collaborative Network.Global Burden of Disease Study 2019 (GBD 2019) Results.Seattle, United States: Institute for Health Metrics and Evaluation (IHME), 2020.Available from http://ghdx.healthdata.org/gbd-results-tool.
10. World Bank staff estimates based on the United Nations Population Division's World Urbanization Prospects: 2018 Revision https://data.worldbank.org/indicator/SP.URB.TOTL.
11. Vos T, Lim SS, Abbafati C, et al. Global burden of 369 diseases and injuries in 204 countries and territories, 1990–2019: a systematic analysis for the Global Burden of Disease Study 2019. *The Lancet* 2020; **396**(10258): 1204-22.
12. United Nations DESA/Population Division. 2018 Revision of the World Urbanization Prospects. 2018.
13. WMO Catalogue of Major Meteorological Hazards. Available at http://puslitbang.bmkg.go.id/litbang/wmo-catalogue-of-major-meteorological-hazards/. 2021.
14. de Perez EC, van Aalst M, Bischiniotis K, et al. Global predictability of temperature extremes. *Environmental Research Letters* 2018; **13**(5).
15. Di Napoli C, Pappenberger F, Cloke HL. Verification of Heat Stress Thresholds for a Health-Based Heat-Wave Definition. *Journal of Applied Meteorology and Climatology* 2019; **58**(6): 1177-94.
16. Xu Z, Sheffield PE, Su H, Wang X, Bi Y, Tong S. The impact of heat waves on children's health: a systematic review. *Int J Biometeorol* 2014; **58**(2): 239-47.
17. Copernicus Climate Change Service (C3S). ERA5 hourly data on single levels from 1979 to present. Available at https://doi.org/10.24381/cds.adbb2d47. 2021.
18. United Nations. 2019 Revision of World Population Prospects. Available at https://population.un.org/wpp/. 2020.
19. Sports Medicine Australia. Extreme Heat Policy. 2021. https://sma.org.au/sma-site-content/uploads/2021/02/SMA-Extreme-Heat-Policy-2021-Final.pdf (accessed 18 May 2021).
20. Armstrong LE, Casa DJ, Millard-Stafford M, Moran DS, Pyne SW, Roberts WO. Exertional Heat Illness during Training and Competition. *Medicine & Science in Sports & Exercise* 2007; **39**(3): 556-72.
21. Casa DJ, DeMartini JK, Bergeron MF, et al. National Athletic Trainers' Association position statement: exertional heat illnesses. *Journal of athletic training* 2015; **50**(9): 986-1000.
22. Kawahara T. [Exercise guideline for prevention of heat injury (Japan Sports Association)]. *Nihon Rinsho* 2012; **70**(6): 1029-32.
23. Hersbach H, Bell B, Berrisford P, et al. The ERA5 global reanalysis. *Q J Roy Meteor Soc* 2020; **146**(730): 1999-2049.

24. CIESIN. GWP (Gridded Population of the World) dataset. 2017.
25. Vanos JK, Baldwin JW, Jay O, Ebi KL. Simplicity lacks robustness when projecting heat-health outcomes in a changing climate. *Nature Communications* 2020; **11**(1): 1-5.
26. Kjellstrom T, Freyberg C, Lemke B, Otto M, Briggs D. Estimating population heat exposure and impacts on working people in conjunction with climate change. *International Journal of Biometeorology* 2018; **62**(3): 291-306.
27. ILO. ILOSTAT database. Geneva, Switzerland: International Labour Organization; 2021.
28. Global Precipitation Climatology Centre (GPCC). Available at ftp://ftp-anon.dwd.de/pub/data/gpcc/first_guess/. 2021.
29. Diener E, Larsen RJ, Levine S, Emmons RA. Intensity and frequency: dimensions underlying positive and negative affect. *Journal of personality and social psychology* 1985; **48**(5): 1253.
30. Pennebaker JW, Boyd RL, Jordan K, Blackburn K. The development and psychometric properties of LIWC2015, 2015.
31. Pennebaker JW, Chung C, Ireland M, Gonzales A, Booth R. The development and psychometric properties of LIWC2007, 2007.
32. Baylis P, Obradovich N, Kryvasheyeu Y, et al. Weather impacts expressed sentiment. *PLoS One* 2018; **13**(4): e0195750.
33. Hsiang S. Climate Econometrics. *Annu Rev Resour Econ* 2016; **8**: 43-75.
34. Obradovich N, Tingley D, Rahwan I. Effects of environmental stressors on daily governance. *Proc Natl Acad Sci U S A* 2018; **115**(35): 8710-5.
35. Obradmich N, Rahwan I. Risk of a feedback loop between climatic warming and human mobility. *J R Soc Interface* 2019; **16**(158).
36. Obradovich N, Migliorini R, Mednick SC, Fowler JH. Nighttime temperature and human sleep loss in a changing climate. *Sci Adv* 2017; **3**(5): e1601555.
37. Wang J, Obradovich N, Zheng S. A 43-Million-Person Investigation into Weather and Expressed Sentiment in a Changing Climate. *One Earth* 2020; **2**(6): 568-77.
38. Honda Y, Kondo M, McGregor G, et al. Heat-related mortality risk model for climate change impact projection. *Environ Health Prev Med* 2014; **19**(1): 56-63.
39. Institute for Health Metrics and Evaluation (IHME) Global Health Data Exchange (GHDx). Global Burden of Disease. Available at http://ghdx.healthdata.org/gbd-results-tool. 2021.
40. United Nations. 2020 Revision of World Population Prospects. Available at https://population.un.org/wpp/. 2021.
41. MCC Collaborative Research Network. MCC Collaborative Research Network. 2020. https://mccstudy.lshtm.ac.uk/ (accessed March 22nd 2021).
42. NASA EarthData. Active Fire Data. Available at https://earthdata.nasa.gov/earth-observation-data/near-real-time/firms/active-fire-data. 2021.
43. Copernicus Climate Change Service (C3S). Fire danger indices historical data from the Copernicus Emergency Management Service. Available at https://doi.org/10.24381/cds.0e89c522. 2021.
44. Black C, Tesfaigzi Y, Bassein JA, Miller LA. Wildfire smoke exposure and human health: Significant gaps in research for a growing public health issue. *Environmental toxicology and pharmacology* 2017; **55**: 186-95.
45. Beguería S, Vicente-Serrano SM, Reig F, Latorre B. Standardized precipitation evapotranspiration index (SPEI) revisited: parameter fitting, evapotranspiration models, tools, datasets and drought monitoring. *International Journal of Climatology* 2014; **34**(10): 3001-23.
46. NOAA NCEP CPC GHCN_CAMS gridded dataset. Available at ftp://ftp.cpc.ncep.noaa.gov/wd51yf/GHCN_CAMS/. 2021.
47. MeteoSwiss. 2020. https://www.meteoswiss.admin.ch/home/climate/swiss-climate-in-detail/climate-indicators/drought-indices/spi-and-spei.html (accessed 23 May 2020).
48. Global SPEI database. Available at https://spei.csic.es/database.html. 2021.

49. Centre for Research on the Epidemiology of Disasters. EM-DAT The International Disaster Database. Available at https://emdat.be/. 2021.
50. United Nations Development Programme. Human Development Reports. Human Development Index (HDI). Available at http://hdr.undp.org/en/content/human-development-index-hdi. 2021.
51. Rocklöv JT, Y; . Climate change and the rising infectiousness of dengue. *Emerging Topics in Life Science* 2019; **ETLS20180123**.
52. Rocklöv J, Quam MB, Sudre B, et al. Assessing Seasonal Risks for the Introduction and Mosquito-borne Spread of Zika Virus in Europe. *EBioMedicine* 2016; **9**: 250-6.
53. Rocklöv J, Tozan Y, Ramadona A, et al. Using Big Data to Monitor the Introduction and Spread of Chikungunya, Europe, 2017. *Emerg Infect Dis* 2019; **25**(6): 1041-9.
54. Liu-Helmersson J, Brännström, Å., Sewe, M., Semenza, J., Rocklöv, J. Estimating past, present and future trends in the global distribution and abundance of the arbovirus vector Aedes aegypti under climate change scenarios. *Frontiers in Public Health* 2019.
55. DiSera L, Sjödin H, Rocklöv J, et al. The Mosquito, the Virus, the Climate: An Unforeseen Réunion in 2018. *Geohealth* 2020; **4**(8): e2020GH000253.
56. Colón-González FJ, Sewe MO, Tompkins AM, et al. (in press)Projecting the risk of mosquito-borne diseases in a warmer and more urbanised world: a multi-model multi-scenario intercomparison modelling study. *Lancet Planetary Health*.
57. Harris I, Osborn TJ, Jones P, Lister D. Version 4 of the CRU TS monthly high-resolution gridded multivariate climate dataset. *Scientific Data* 2020; **7**(1): 109.
58. Klein Goldewijk K, Beusen A, Doelman J, Stehfest E. Anthropogenic land use estimates for the Holocene – HYDE 3.2. *Earth Syst Sci Data* 2017; **9**(2): 927-53.
59. Liu-Helmersson J, Stenlund H, Wilder-Smith A, Rocklöv J. Vectorial capacity of Aedes aegypti: effects of temperature and implications for global dengue epidemic potential. *PloS one* 2014; **9**(3).
60. Liu-Helmersson J, Quam M, Wilder-Smith A, et al. Climate Change and Aedes Vectors: 21st Century Projections for Dengue Transmission in Europe. *EBioMedicine* 2016; **7**: 267-77.
61. Rocklöv JT, Y; . Climate change and the rising infectiousness of dengue. *Emerging Topics in Life Science* 2019; **ETLS20180123**(2): 133-42.
62. New M, Lister D, Hulme M, Makin I. A high-resolution data set of surface climate over global land areas. *Climate research* 2002; **21**(1): 1-25.
63. Grover-Kopec EK, Blumenthal MB, Ceccato P, Dinku T, Omumbo JA, Connor SJ. Web-based climate information resources for malaria control in Africa. *Malaria journal* 2006; **5**(1): 38.
64. Koninklijk Nederlands Meteorologisch Instituut. KNMI Climate Explorer. Available at https://climexp.knmi.nl/. 2021.
65. University of Washington Joint Institute for the Study of the Atmosphere and Ocean (JISAO). Elevation data. Available at http://research.jisao.washington.edu/data_sets/elevation/. 2020.
66. Gething PW, Van Boeckel TP, Smith DL, et al. Modelling the global constraints of temperature on transmission of Plasmodium falciparum and P. vivax. *Parasites & vectors* 2011; **4**(1): 92.
67. Snow RW, Sartorius B, Kyalo D, et al. The prevalence of Plasmodium falciparum in sub-Saharan Africa since 1900. *Nature* 2017; **550**(7677): 515-8.
68. Martinez-Urtaza J, van Aerle R, Abanto M, et al. Genomic Variation and Evolution of Vibrio parahaemolyticus ST36 over the Course of a Transcontinental Epidemic Expansion. *mBio* 2017; **8**(6).
69. Muhling BA, Gaitán CF, Stock CA, Saba VS, Tommasi D, Dixon KW. Potential Salinity and Temperature Futures for the Chesapeake Bay Using a Statistical Downscaling Spatial Disaggregation Framework. *Estuaries and Coasts* 2017; **41**(2): 349-72.
70. Parveen S, Hettiarachchi KA, Bowers JC, et al. Seasonal distribution of total and pathogenic Vibrio parahaemolyticus in Chesapeake Bay oysters and waters. *International journal of food microbiology* 2008; **128**(2): 354-61.
71. Copernicus Marine Environment Monitoring Service. Mercator Ocean Reanalysis. Available at http://marine.copernicus.eu/. 2021.

72. NOAA Earth System Research Laboratory. Optimum Interpolation 1/4 Degree Daily Sea Surface Temperature Analysis version 2. Available at https://www.ncdc.noaa.gov/oisst. 2021.
73. Watts N, Amann M, Arnell N, et al. The 2019 report of The *Lancet* Countdown on health and climate change: ensuring that the health of a child born today is not defined by a changing climate. *The Lancet* 2019; **394**(10211): 1836-78.
74. Colwell R, Huq A. Marine ecosystems and cholera. *Hydrobiologia* 2001; **460**: 141-5.
75. Jutla AS, Akanda AS, Griffiths JK, Colwell R, Islam S. Warming oceans, phytoplankton, and river discharge: implications for cholera outbreaks. *Am J Trop Med Hyg* 2011; **85**(2): 303-8.
76. Escobar LE, Craft ME. Advances and Limitations of Disease Biogeography Using Ecological Niche Modeling. *Front Microbiol* 2016; **7**.
77. Flanders Marine Institute. The intersect of the Exclusive Economic Zones and IHO sea areas, version 3. Available at http://www.marineregions.org/. 2018.
78. Escobar LE, Qiao H, Lee C, Phelps NBD. Novel Methods in Disease Biogeography: A Case Study with Heterosporosis. *Front Vet Sci* 2017; **4**: 105.
79. Cobos ME, Jiménez L, Nuñez-Penichet C, Romero-Alvarez D, Simões M. Sample data and training modules for cleaning biodiversity information. *Biodiversity Informatics* 2018; **13**: 49-50.
80. Blonder B, Morrow CB, Maitner B, et al. New approaches for delineating n-dimensional hypervolumes. *Methods in Ecology and Evolution* 2018; **9**(2): 305-19.
81. Escobar LE, Ryan SJ, Stewart-Ibarra AM, et al. A global map of suitability for coastal Vibrio cholerae under current and future climate conditions. *Acta Trop* 2015; **149**: 202-11.
82. Ryan SJ, Stewart-Ibarra AM, Ordonez-Enireb E, et al. Spatiotemporal Variation in Environmental Vibrio cholerae in an Estuary in Southern Coastal Ecuador. *Int J Environ Res Public Health* 2018; **15**(3).
83. Johnson EE, Escobar LE, Zambrana-Torrelio C. An Ecological Framework for Modeling the Geography of Disease Transmission. *Trends Ecol Evol* 2019; **34**(7): 655-68.
84. Blonder B, Lamanna C, Violle C, Enquist BJ. The n-dimensional hypervolume. *Global Ecology and Biogeography* 2014; **23**(5): 595-609.
85. Blonder B, Blonder MB, Rcpp D, Rcpp L. Package 'hypervolume'. *Global Ecology and Biogeography* 2015; **23**: 595-609.
86. Castaneda-Guzman M, G. M-S, Murray KA, Settlage R, Escobar LE. A Database of Global Coastal Conditions. Available at https://figshare.com/projects/A_Database_of_Global_Coastal_Conditions/96989. 2021.
87. NOAA SWFSC ERD. Sea surface temperature. Available at https://coastwatch.pfeg.noaa.gov/erddap/griddap/erdMH1sstdmday.html. 2021.
88. NOAA SWFSC ERD. Chlorophyll-a. Available at https://coastwatch.pfeg.noaa.gov/erddap/griddap/erdMH1chlamday.html. 2021.
89. WHO, UNICEF. 4WHO/UNICEF Joint Monitoring Programme (JMP) for Water Supply, Sanitation and Hygiene. 2021. https://unstats.un.org/sdgs/ (accessed 18 May 2021).
90. UNDP. Gross national income (GNI) per capita (constant 2017 PPP$). 2021. http://hdr.undp.org/en/indicators/195706 (accessed 18 May 2021).
91. IHME. GBD Results Tool. 2019.
92. Challinor AJ, Koehler AK, Ramirez-Villegas J, Whitfield S, Das B. Current warming will reduce yields unless maize breeding and seed systems adapt immediately. *Nature Climate Change* 2016; **6**(10): 954-+.
93. Aslam MA, Ahmed M, Stöckle CO, Higgins SS, Hayat R. Can growing degree days and photoperiod predict spring wheat phenology? *Frontiers in Environmental Science* 2017; **5**: 57.
94. Portmann FT, Siebert S, Döll P. MIRCA2000—Global monthly irrigated and rainfed crop areas around the year 2000: A new high-resolution data set for agricultural and hydrological modeling. *Global biogeochemical cycles* 2010; **24**(1).
95. Muñoz Sabater J. ERA5-Land hourly data from 1981 to present. Copernicus Climate Change Service (C3S) Climate Data Store (CDS). 2019.

96. Sacks WJ, Deryng D, Foley JA, Ramankutty N. Crop planting dates: an analysis of global patterns. *Global Ecology and Biogeography* 2010; **19**(5): 607-20.
97. Saint Ville A, Po JYT, Sen A, Bui A, Melgar-Quiñonez H. Food security and the Food Insecurity Experience Scale (FIES): ensuring progress by 2030. *Food Security* 2019; **11**(3): 483-91.
98. FAO. The State of World Fisheries and Aquaculture 2018 - Meeting the sustainable development goals. Rome: Food and Agriculture Organization of the United Nations, 2018.
99. Hoegh-Guldberg O, Cai R, Poloczanska E, Brewer P, Sundby S, Hilmi K. The Ocean In: Barros VR, Field CB, Dokken DJ, Mastrandrea MD, Mach KJ, Bilir TE, et al., editors. Climate Change 2014: Impacts, Adaptation, and Vulnerability Part B: Regional Aspects Contribution of Working Group II to the Fifth Assessment Report of the Intergovernmental Panel of Climate Change. Cambridge, United Kingdom and New York, NY. USA: Cambridge University Press; 2014.
100. NASA Earth Observations (NEO). Sea surface temperature (1 month—AQUA/MODIS). Available at https://neo.sci.gsfc.nasa.gov/view.php?datasetId=MYD28M. 2021.
101. National Oceanic and Atmospheric Administration (NOAA). Coral Reef Watch Version 3.1 Daily Global 5-km Satellite Coral Bleaching Degree Heating Week Product. 2021.
102. FAO. New Food Balance Sheets. Available at http://www.fao.org/faostat/en/#data/FBS. 2021.
103. Murray CJL, Aravkin AY, Zheng P, et al. Global burden of 87 risk factors in 204 countries and territories, 1990–2019: a systematic analysis for the Global Burden of Disease Study 2019. *The Lancet* 2020; **396**(10258): 1223-49.
104. Diaz DB. Estimating global damages from sea level rise with the Coastal Impact and Adaptation Model (CIAM). *Climatic change* 2016; **137**(1-2): 143-56.
105. Jevrejeva S, Jackson L, Grinsted A, Lincke D, Marzeion B. Flood damage costs under the sea level rise with warming of 1.5 C and 2 C. *Environmental Research Letters* 2018; **13**(7): 074014.
106. Kopp RE, DeConto RM, Bader DA, et al. Evolving Understanding of Antarctic Ice-Sheet Physics and Ambiguity in Probabilistic Sea-Level Projections. *Earth's Future* 2017; **5**(12): 1217-33.
107. Kulp SA, Strauss BH. CoastalDEM: a global coastal digital elevation model improved from SRTM using a neural network. *Remote sensing of environment* 2018; **206**: 231-9.
108. Rose AN, McKee JJ, Sims KM, Bright EA, Reith AE, Urban ML. LandScan 2019. 2019 ed. Oak Ridge, TN: Oak Ridge National Laboratory; 2020.
109. Global Administrative Areas (GADM) version 3.6. Available at http://www.gadm.org/. 2021.
110. Smits J, Permanyer I. The Subnational Human Development Database. *Sci Data* 2019; **6**: 190038.
111. Pörtner H, Roberts D, Masson-Delmotte V, et al. IPCC Special Report on the Ocean and Cryosphere in a Changing Climate. *IPCC Intergovernmental Panel on Climate Change: Geneva, Switzerland* 2019.
112. Thomas MA, Lin T. Illustrative Analysis of Probabilistic Sea Level Rise Hazard. *Journal of Climate* 2020; **33**(4): 1523-34.
113. McMichael C, Dasgupta S, Ayeb-Karlsson S, Kelman I. A review of estimating population exposure to sea-level rise and the relevance for migration. *Environmental Research Letters* 2020; **15**(12).
114. Kulp SA, Strauss BH. New elevation data triple estimates of global vulnerability to sea-level rise and coastal flooding. *Nature communications* 2019; **10**(1): 1-12.
115. Mondal P, Tatem AJ. Uncertainties in measuring populations potentially impacted by sea level rise and coastal flooding. *PLoS One* 2012; **7**(10).
116. Ayeb-Karlsson S, Kniveton D, Cannon T. Trapped in the prison of the mind: Notions of climate-induced (im)mobility decision-making and wellbeing from an urban informal settlement in Bangladesh. *Palgr Commun* 2020; **6**(1).
117. Hauer ME, Fussell E, Mueller V, et al. Sea-level rise and human migration. *Nature Reviews Earth & Environment* 2020; **1**(1): 28-39.
118. Luber G, Knowlton K, Balbus J, et al. Human health. *Climate change impacts in the United States: the third National Climate Assessment* 2014: 220-56.

119. Dannenberg AL, Frumkin H, Hess JJ, Ebi KL. Managed retreat as a strategy for climate change adaptation in small communities: public health implications. *Climatic change* 2019; **153**(1-2): 1-14.
120. McMichael C, Katonivualiku M, Powell T. Planned relocation and everyday agency in low-lying coastal villages in Fiji. *The Geographical Journal* 2019; **185**(3): 325-37.
121. Schütte S, Gemenne F, Zaman M, Flahault A, Depoux A. Connecting planetary health, climate change, and migration. *The Lancet Planetary Health* 2018; **2**(2): e58-e9.
122. International Organization for Migration. Implementation of the Workplan of the Task Force on Displacement under the Warsaw International Mechanism for Loss and Damage, United Nations Framework Convention on Climate Change (UNFCCC), Pillar I: Policy/Practice – National/Subnational Activity I.1: Mapping Human Mobility and Climate Change in Relevant National Policies and Institutional Frameworks 2018. https://environmentalmigration.iom.int/iom-pdd-task-force-displacement-stakeholder-meeting.
123. Watts N, Amann M, Arnell N, et al. The 2018 report of the Lancet Countdown on health and climate change: shaping the health of nations for centuries to come. *The Lancet* 2018; **392**(10163): 2479-514.
124. Watts N, Amann M, Ayeb-Karlsson S, et al. The Lancet Countdown on health and climate change: from 25 years of inaction to a global transformation for public health. *The Lancet* 2017.
125. Abubakar I, Aldridge RW, Devakumar D, et al. The UCL–Lancet Commission on Migration and Health: the health of a world on the move. *The Lancet* 2018; **392**(10164): 2606-54.
126. Kelman I. Difficult decisions: migration from small island developing states under climate change. *Earth's Future* 2015; **3**(4): 133-42.
127. Kelman I. Islandness within climate change narratives of small island developing states (SIDS). *Island Studies Journal* 2018; **13**(1): 149-66.
128. Kelman I. Imaginary Numbers of Climate Change Migrants? *Social Sciences* 2019; **8**(5).
129. Baldwin A. Premediation and white affect: climate change and migration in critical perspective. *Transactions of the Institute of British Geographers* 2016; **41**(1): 78-90.
130. Ayeb-Karlsson S, Smith CD, Kniveton D. A discursive review of the textual use of 'trapped'in environmental migration studies: The conceptual birth and troubled teenage years of trapped populations. *Ambio* 2018; **47**(5): 557-73.
131. WHO. WHO Health and Climate Change Global Survey. Geneva, Switzerland: World Health Organization, 2021.
132. CDP. 2020 Annual Cities Survey Data. In: CDP, editor. London, UK; 2021.
133. WMO. Country Profile Database. Geneva, Switzerland; 2021.
134. WHO. Global Health Observatory Data Repository. 2019.
135. Miettinen OS. Proportion of disease caused or prevented by a given exposure, trait or intervention. *American journal of epidemiology* 1974; **99**(5): 325-32.
136. Ciancio B, Di Renzi M, Binkin N, et al. Fattori di rischio per la mortalita durante un'ondata di calore a Bari, estate 2005. *Bollettino Epidemiologico Nazionale* 2007; **63**: 113-25.
137. Kaiser R, Rubin CH, Henderson AK, et al. Heat-related death and mental illness during the 1999 Cincinnati heat wave. *The American journal of forensic medicine and pathology* 2001; **22**(3): 303-7.
138. Lorente C, Serazin C, Salines G, et al. Etude des facteurs de décès des personnes âgées résidant en établissement durant la vague de chaleur d'août 2003: Institut de Veille Sanitaire (InVS), 2005.
139. Marmor M. Heat wave mortality in nursing homes. *Environmental Research* 1978; **17**(1): 102-15.
140. Naughton MP, Henderson A, Mirabelli MC, et al. Heat-related mortality during a 1999 heat wave in Chicago. *American journal of preventive medicine* 2002; **22**(4): 221-7.
141. Semenza JC, Rubin CH, Falter KH, et al. Heat-related deaths during the July 1995 heat wave in Chicago. *New England journal of medicine* 1996; **335**(2): 84-90.
142. Theocharis G, Tansarli G, Mavros M, Spiropoulos T, Barbas S, Falagas M. Association between use of air-conditioning or fan and survival of elderly febrile patients: a prospective study. *European journal of clinical microbiology & infectious diseases* 2013; **32**(9): 1143-7.

143. Vandentorren S, Bretin P, Zeghnoun A, et al. August 2003 heat wave in France: risk factors for death of elderly people living at home. *The European Journal of Public Health* 2006; **16**(6): 583-91.
144. Zhang Y, Nitschke M, Krackowizer A, et al. Risk factors for deaths during the 2009 heat wave in Adelaide, Australia: a matched case-control study. *Int J Biometeorol* 2017; **61**(1): 35-47.
145. Strain T, Brage S, Sharp SJ, et al. Use of the prevented fraction for the population to determine deaths averted by existing prevalence of physical activity: a descriptive study. *The Lancet Global Health* 2020; **8**(7): e920-e30.
146. Bishop YM, Fienberg SE, Holland PW. Discrete multivariate analysis: theory and practice: Springer Science & Business Media; 2007.
147. European Commission. Global Human Settlement. 2021.
148. Center For International Earth Science Information Network CIESIN-Columbia University. Gridded Population of the World, Version 4 (GPWv4): Population Density, Revision 11. 2017.
149. Kriegler FJ, Malila WA, Nalepka RF, Richardson W. Preprocessing Transformations and Their Effects on Multispectral Recognition. Proceedings of the Sixth International Symposium on Remote Sensing of Environment; 1969 1969; 1969. p. 97.
150. U. S. Geological Survey. U.S. Geological Survey Landsat Data. 2021.
151. Rhew IC, Vander Stoep A, Kearney A, Smith NL, Dunbar MD. Validation of the Normalized Difference Vegetation Index as a Measure of Neighborhood Greenness. *Ann Epidemiol* 2011; **21**(12): 946-52.
152. James P, Banay RF, Hart JE, Laden F. A Review of the Health Benefits of Greenness. *Curr Epidemiol Rep* 2015; **2**(2): 131-42.
153. Fong KC, Hart JE, James P. A Review of Epidemiologic Studies on Greenness and Health: Updated Literature Through 2017. *Current Environmental Health Reports* 2018; **5**(1): 77-87.
154. kMatrix. Adaptation and Resilience to Climate Change Dataset. 2020.
155. Jaikumar R. Postindustrial Manufacturing. Harvard Business Review. 1986 1986.
156. Georgeson L, Maslin M, Poessinouw M. The global green economy: a review of concepts, definitions, measurement methodologies and their interactions: Global green economy: definitions & measurement. *Geo: Geography and Environment* 2017; **4**(1): e00036.
157. International Monetary Fund. World Economic Outlook Database. 2018.
158. Climate Funds Update. Climate Funds Update Data Dashboard. 2021. https://climatefundsupdate.org/data-dashboard/ (accessed 3 May 2021).
159. Le Quéré C, Peters GP, Friedlingstein P, et al. Fossil CO2 emissions in the post-COVID-19 era. *Nature Climate Change* 2021; **11**(3): 197-9.
160. IEA. CO2 Emissions from Fuel Combustion (2020 edition). UK Data Service; 2021.
161. IEA. World Energy Outlook 2020. Paris: IEA, 2021.
162. IEA. IEA Statistical Report. Paris: IEA, 2020.
163. IEA. Global Energy Review 2021 (forthcoming). Paris: IEA, 2021.
164. IEA. CO2 Emissions From Fuel Combustion: Database Documentation (2020 Edition). 2020. http://wds.iea.org/wds/pdf/WorldCO2_Documentation.pdf (accessed 19 May 2021).
165. IEA. World Extended Energy Balances (2020 edition). UK Data Service; 2021.
166. IEA. World Energy Balances: Data Documentation (2020 edition). 2020. http://wds.iea.org/wds/pdf/WORLDBAL_Documentation.pdf). (accessed 19 May 2021).
167. IEA. Methodology. Defining energy access. 2020. https://www.iea.org/articles/defining-energy-access-2020-methodology (accessed 14 April 2021 2021).
168. WHO. Indicator 7.1.2: Proportion of population with primary reliance on clean fuels and technology. 19 July 2016 2016. https://unstats.un.org/sdgs/metadata/files/Metadata-07-01-02.pdf (accessed 14 April 2021).
169. WHO. Household Energy Database. Geneva: World Health Organization; 2021.
170. Stoner O, Lewis J, Martínez I, Gumy S, Economou T, Adair-Rohani H. What fuels the fire? Cooking fuel estimates at global, regional and country level for 1990-2030: In Review, 2021.

171. Stoner O, Shaddick G, Economou T, et al. Global household energy model: a multivariate hierarchical approach to estimating trends in the use of polluting and clean fuels for cooking. *Journal of the Royal Statistical Society: Series C (Applied Statistics)* 2020; **69**(4): 815-39.
172. United Nations Population Division. Urban population (% of total population). World Urbanization Prospects: 2018 Revision. 2019 ed. New York: World Bank; 2018.
173. IEA. World Extended Energy Balances (2019 edition). UK Data Service; 2020.
174. WHO. Household Energy database. 2020.
175. Shupler M, Godwin W, Frostad J, Gustafson P, Arku R, M B. Global estimation of exposure to fine particulate matter (PM2.5) from household air pollution. *Environ Int* 2018; **20**: 354–63.
176. IEA. World Extended Energy Balances. UK Data Service; 2020.
177. UNDESA. World Population Prospects: The 2017 revision. New York, NY, USA: United Nations Department of Economic and Social Affairs; 2017.
178. Roth GA, Abate D, Abate KH, et al. Global, regional, and national age-sex-specific mortality for 282 causes of death in 195 countries and territories, 1980–2017: a systematic analysis for the Global Burden of Disease Study 2017. *The Lancet* 2018; **392**(10159): 1736-88.
179. Institute for Health Metrics and Evaluation. GBD Results Tool. 2021. http://ghdx.healthdata.org/gbd-results-tool (accessed 6 May 2021).
180. Amann M, Bertok I, Borken-Kleefeld J, et al. Cost-effective control of air quality and greenhouse gases in Europe: Modeling and policy applications. *Environmental Modelling & Software* 2011; **26**(12): 1489-501.
181. IEA. World Energy Outlook 2019. Paris: IEA, 2019.
182. IEA. World Energy Outlook 2020. Paris: IEA, 2020.
183. Simpson D, Benedictow A, Berge H, et al. The EMEP MSC-W chemical transport model–technical description. *Atmospheric Chemistry and Physics* 2012; **12**(16): 7825-65.
184. Kiesewetter G, Borken-Kleefeld J, Schöpp W, et al. Modelling street level PM 10 concentrations across Europe: source apportionment and possible futures. *Atmospheric Chemistry and Physics* 2015; **15**(3): 1539-53.
185. Amann M, Kiesewetter G, Schöpp W, et al. Reducing global air pollution: the scope for further policy interventions. *Phil Trans R Soc A* 2020; **378**(2183).
186. WHO. WHO Global Urban Ambient Air Pollution Database (update 2018). . Geneva: World Health Organization; 2018.
187. Global Burden of Disease Collaborative Network. Particulate Matter Risk Curves. Seattle: Institute for Health Metrics and Evaluation (IHME), 2021.
188. Forouzanfar MH, Alexander L, Anderson HR, et al. Global, regional, and national comparative risk assessment of 79 behavioural, environmental and occupational, and metabolic risks or clusters of risks in 188 countries, 1990–2013: a systematic analysis for the Global Burden of Disease Study 2013. *The Lancet* 2015; **386**(10010): 2287-323.
189. WHO European Centre for Environment and Health. Review of evidence on health aspects of air pollution - REVIHAAP Project. Copenhagen, Denmark: WHO Regional Office for Europe, 2013.
190. IEA. World Extended Energy Balances (2020 edition). Paris: IEA; 2020

191. IEA. World Extended Energy Balances (2020 edition). UK Data Service; 2020.
192. UNDESA. World Population Prospects 2019. 2019 ed: United Nations; 2019.
193. Woodcock J, Givoni M, Morgan AS. Health impact modelling of active travel visions for England and Wales using an Integrated Transport and Health Impact Modelling Tool (ITHIM). *PLoS One* 2013; **8**(1): e51462.
194. Herrero M, Havlík P, Valin H, et al. Biomass use, production, feed efficiencies, and greenhouse gas emissions from global livestock systems. *Proceedings of the National Academy of Sciences* 2013; **110**(52): 20888-93.
195. FAO. FAOSTAT. 2020. http://www.fao.org/faostat/ (accessed 15 April 2021).

196. Chang J, Ciais P, Herrero M, et al. Combining livestock production information in a process-based vegetation model to reconstruct the history of grassland management. *Biogeosciences* 2016; **13**(12): 3757-76.
197. Carlson KM, Gerber JS, Mueller ND, et al. Greenhouse gas emissions intensity of global croplands. *Nature Climate Change* 2017; **7**(1): 63-8.
198. Dalin C, Tuninetti M, Carlson K, et al. Variability, drivers and interactions of key environmental stressors from food production worldwide. EGU2019; 2019; Vienna: 21st EGU General Assembly; 2019.
199. Dalin C, Wada Y, Kastner T, Puma MJ. Groundwater depletion embedded in international food trade. *Nature* 2017; **543**: 700–4.
200. Kastner T, Kastner M, Nonhebel S. Tracing distant environmental impacts of agricultural products from a consumer perspective. *Ecological Economics* 2011; **70**(6): 1032–40.
201. Poore J, Nemecek T. Reducing food's environmental impacts through producers and consumers. *Science* 2018; (360): 987–92.
202. Wang H, Abbas KM, Abbasifard M, et al. Global age-sex-specific fertility, mortality, healthy life expectancy (HALE), and population estimates in 204 countries and territories, 1950–2019: a comprehensive demographic analysis for the Global Burden of Disease Study 2019. *The Lancet* 2020; **396**(10258): 1160-203.
203. Gustavsson J, Cederberg C, Sonesson U, Van Otterdijk R, Meybeck A. Global food losses and food waste: extent, causes and prevention, 2011.
204. Food and Agriculture Organization of the United Nations. Food balance sheets: a handbook. Rome: FAO, 2001.
205. Miller V, Singh GM, Onopa J, et al. Global Dietary Database 2017: data availability and gaps on 54 major foods, beverages and nutrients among 5.6 million children and adults from 1220 surveys worldwide. *BMJ Global Health* 2021; **6**(2): e003585.
206. Gobbo DLC, Khatibzadeh S, Imamura F, et al. Assessing global dietary habits: a comparison of national estimates from the FAO and the Global Dietary Database. *The American Journal of Clinical Nutrition* 2015; **101**(5): 1038-46.
207. Micha R, Khatibzadeh S, Shi P, et al. Global, regional and national consumption of major food groups in 1990 and 2010: a systematic analysis including 266 country-specific nutrition surveys worldwide. *BMJ Open* 2015; **5**(9): e008705.
208. Freedman LS, Commins JM, Moler JE, et al. Pooled results from 5 validation studies of dietary self-report instruments using recovery biomarkers for energy and protein intake. *American journal of epidemiology* 2014; **180**(2): 172-88.
209. Rennie KL, Coward A, Jebb SA. Estimating under-reporting of energy intake in dietary surveys using an individualised method. *British Journal of Nutrition* 2007; **97**(6): 1169-76.
210. N. C. D. Risk Factor Collaboration. Trends in adult body-mass index in 200 countries from 1975 to 2014: a pooled analysis of 1698 population-based measurement studies with 19·2 million participants. *The Lancet* 2016; **387**(10026): 1377-96.
211. Murray CJL, Ezzati M, Lopez AD, Rodgers A, Vander Hoorn S. Comparative quantification of health risks: conceptual framework and methodological issues. *Population Health Metrics* 2003; **1**(1): 1.
212. Lim SS, Vos T, Flaxman AD, et al. A comparative risk assessment of burden of disease and injury attributable to 67 risk factors and risk factor clusters in 21 regions, 1990–2010: a systematic analysis for the Global Burden of Disease Study 2010. *The Lancet* 2012; **380**(9859): 2224-60.
213. Forouzanfar MH, Alexander L, Anderson HR, et al. Global, regional, and national comparative risk assessment of 79 behavioural, environmental and occupational, and metabolic risks or clusters of risks in 188 countries, 1990–2013: a systematic analysis for the Global Burden of Disease Study 2013. *The Lancet* 2015; **386**(10010): 2287-323.
214. Murray CJL, Ezzati M, Flaxman AD, et al. GBD 2010: design, definitions, and metrics. *Lancet* 2012; **380**(9859): 2063-6.

215. Afshin A, Micha R, Khatibzadeh S, Mozaffarian D. Consumption of nuts and legumes and risk of incident ischemic heart disease, stroke, and diabetes: a systematic review and meta-analysis. *The American Journal of Clinical Nutrition* 2014; **100**(1): 278-88.
216. Aune D, Keum N, Giovannucci E, et al. Nut consumption and risk of cardiovascular disease, total cancer, all-cause and cause-specific mortality: a systematic review and dose-response meta-analysis of prospective studies. *BMC medicine* 2016; **14**(1): 207.
217. Aune D, Giovannucci E, Boffetta P, et al. Fruit and vegetable intake and the risk of cardiovascular disease, total cancer and all-cause mortality—a systematic review and dose-response meta-analysis of prospective studies. *International Journal of Epidemiology* 2017; **46**(3): 1029-56.
218. Bechthold A, Boeing H, Schwedhelm C, et al. Food groups and risk of coronary heart disease, stroke and heart failure: A systematic review and dose-response meta-analysis of prospective studies. *Critical Reviews in Food Science and Nutrition* 2019; **59**(7): 1071-90.
219. Schwingshackl L, Hoffmann G, Lampousi AM, et al. Food groups and risk of type 2 diabetes mellitus: a systematic review and meta-analysis of prospective studies. *European Journal of Epidemiology* 2017; **32**(5): 363-75.
220. Schwingshackl L, Schwedhelm C, Hoffmann G, et al. Food groups and risk of colorectal cancer. *International Journal of Cancer* 2018; **142**(9): 1748-58.
221. Global Bmi Mortality Collaboration DE, Di Angelantonio E, Bhupathiraju S, et al. Body-mass index and all-cause mortality: individual-participant-data meta-analysis of 239 prospective studies in four continents. *Lancet (London, England)* 2016; **388**(10046): 776-86.
222. Singh GM, Danaei G, Farzadfar F, et al. The Age-Specific Quantitative Effects of Metabolic Risk Factors on Cardiovascular Diseases and Diabetes: A Pooled Analysis. *PLOS ONE* 2013; **8**(7): e65174.
223. Micha R, Shulkin ML, Peñalvo JL, et al. Etiologic effects and optimal intakes of foods and nutrients for risk of cardiovascular diseases and diabetes: Systematic reviews and meta-analyses from the Nutrition and Chronic Diseases Expert Group (NutriCoDE). *PLOS ONE* 2017; **12**(4): e0175149.
224. GBD Diet Collaborators A, Sur PJ, Fay KA, et al. Health effects of dietary risks in 195 countries, 1990-2017: a systematic analysis for the Global Burden of Disease Study 2017. *Lancet (London, England)* 2019; **0**(0).
225. Schwingshackl L, Knüppel S, Schwedhelm C, et al. Perspective: NutriGrade: A Scoring System to Assess and Judge the Meta-Evidence of Randomized Controlled Trials and Cohort Studies in Nutrition Research. *Advances in Nutrition: An International Review Journal* 2016; **7**(6): 994-1004.
226. World Cancer Research Fund/American Institute for Cancer R. Diet, Nutrition, Physical Activity and Cancer: A Global Perspective. Continuous Update Project Expert Report. 2018.
227. Aune D, Lau R, Chan DSM, et al. Dairy products and colorectal cancer risk: a systematic review and meta-analysis of cohort studies. *Annals of Oncology: Official Journal of the European Society for Medical Oncology* 2012; **23**(1): 37-45.
228. Aune D, Norat T, Romundstad P, Vatten LJ. Dairy products and the risk of type 2 diabetes: a systematic review and dose-response meta-analysis of cohort studies. *The American Journal of Clinical Nutrition* 2013; **98**(4): 1066-83.
229. Mohan D, Mente A, Dehghan M, et al. Associations of Fish Consumption With Risk of Cardiovascular Disease and Mortality Among Individuals With or Without Vascular Disease From 58 Countries. *JAMA Internal Medicine* 2021.
230. Food and Agriculture Organization of the United Nations. Food balance sheets. 2020.
231. Satija A, Yu E, Willett WC, Hu FB. Understanding Nutritional Epidemiology and Its Role in Policy. *Advances in Nutrition* 2015; **6**(1): 5-18.
232. Zheng J, Huang T, Yu Y, Hu X, Yang B, Li D. Fish consumption and CHD mortality: an updated meta-analysis of seventeen cohort studies. *Public Health Nutrition* 2012; **15**(4): 725-37.
233. Aune D, Keum N, Giovannucci E, et al. Whole grain consumption and risk of cardiovascular disease, cancer, and all cause and cause specific mortality: systematic review and dose-response meta-analysis of prospective studies. *BMJ (Clinical research ed)* 2016; **353**: i2716.

234. Prospective Studies Collaboration, Whitlock G, Lewington S, et al. Body-mass index and cause-specific mortality in 900 000 adults: collaborative analyses of 57 prospective studies. *Lancet* 2009; **373**(9669): 1083-96.
235. Schwingshackl L, Hoffmann G, … KIAjo, Undefined. Food groups and intermediate disease markers: a systematic review and network meta-analysis of randomized trials. *American Journal of Clinical Nutrition* 2018; **108**(3): 576-86.
236. Xun P, Qin B, Song Y, et al. Fish consumption and risk of stroke and its subtypes: accumulative evidence from a meta-analysis of prospective cohort studies. *European Journal of Clinical Nutrition* 2012; **66**(11): 1199-207.
237. Zhao LG, Sun JW, Yang Y, Ma X, Wang YY, Xiang YB. Fish consumption and all-cause mortality: a meta-analysis of cohort studies. *European Journal of Clinical Nutrition* 2016; **70**(2): 155-61.
238. Jayedi A, Shab-Bidar S, Eimeri S, Djafarian K. Fish consumption and risk of all-cause and cardiovascular mortality: A dose-response meta-analysis of prospective observational studies. *Public Health Nutrition* 2018; **21**(7): 1297-306.
239. Guasch-Ferré M, Satija A, Blondin SA, et al. Meta-Analysis of Randomized Controlled Trials of Red Meat Consumption in Comparison With Various Comparison Diets on Cardiovascular Risk Factors. *Circulation* 2019; **139**(15): 1828-45.
240. Schwingshackl L, Knüppel S, Michels N, et al. Intake of 12 food groups and disability-adjusted life years from coronary heart disease, stroke, type 2 diabetes, and colorectal cancer in 16 European countries. *European Journal of Epidemiology* 2019; **34**(8): 765-75.
241. Miller RE, Blair PD. Input-output analysis: foundations and extensions. Cambridge, UK: Cambridge University Press; 2009.
242. Dietzenbacher E, Los B, Stehrer R, Timmer M, De Vries G. The construction of world input–output tables in the WIOD project. *Economic Systems Research* 2013; **25**(1): 71-98.
243. Stadler K, Wood R, Bulavskaya T, et al. EXIOBASE 3: Developing a time series of detailed environmentally extended multi-regional input-output tables. *Journal of Industrial Ecology* 2018; **22**(3): 502-15.
244. WBG. Consumer price index (2010 = 100). 2020. https://data.worldbank.org/indicator/FP.CPI.TOTL?end=2017&locations=US&start=2000.
245. WHO. Global Health Expenditure Database: Indicators and data. Geneva, Switzerland: World Health Organization; 2019.
246. Pichler P-P, Jaccard IS, Weisz U, Weisz H. International comparison of health care carbon footprints. *Environmental Research Letters* 2019; **14**(6): 064004.
247. Health Care Without Harm. Health Care's Climate Footprint: Health Care Without Harm, 2019.
248. Gütschow J, Jeffery L, Gieseke R, Günther A. The PRIMAP-hist national historical emissions time series (1850-2017). v2.1. 2019. https://doi.org/10.5880/pik.2019.018.
249. WHO. Current health expenditure by financing schemes, in Global Health Expenditure Database. In: Organization WH, editor.; 2020.
250. UNSD. Basic Data Selection. United Nations Statistics Division; 2019.
251. Swiss Re. Sigma explorer. Zurich, Switzerland: Swiss Re; 2021.
252. International Monetary Fund. World Economic Outlook Update April 2021. Washington, DC, USA: International Monetary Fund, 2021.
253. Aldy JE, Viscusi WK. Age Differences in the Value of Statistical Life: Revealed Preference Evidence. *Review of Environmental Economics and Policy* 2007; **1**(2): 241-60
254. Robinson LA, Sullivan R, Shogren JF. Do the Benefits of COVID-19 Policies Exceed the Costs? Exploring Uncertainties in the Age–VSL Relationship. *Risk Analysis* 2020.
255. World Bank Group. Population, total,. Washington, DC, USA; 2019.
256. OECD. Gross domestic product (GDP) : GDP per head, US $, constant prices, constant PPPs, reference year 2015, millions. 2021.
257. WHO. WHO methods and data sources for global burden of disease estimates 2000-2015 2017.

258. OECD. Mortality Risk Valuation in Environment, Health and Transport Policies. OECD publishing; 2012. p. 3.
259. ILO. ILOSTAT Statistics on wages. 2021. https://ilostat.ilo.org/topics/wages/ (accessed 1st April 2021).
260. ILO. ILOSTAT: Concepts and Definitions. 2021. https://ilostat.ilo.org/resources/concepts-and-definitions/ (accessed 1st April 2021).
261. IMF. International Finance Statistics. 2021. https://data.imf.org/?sk=4c514d48-b6ba-49ed-8ab9-52b0c1a0179b (accessed 21st March 2021).
262. IMF. World Economic Outlook Database. 2021. https://www.imf.org/en/Publications/WEO/weo-database/2020/October (accessed 25th March 2021).
263. Muriithi MK, Mutegi RG, Mwabu G. Counting unpaid work in Kenya: Gender and age profiles of hours worked and imputed wage incomes. *The Journal of the Economics of Ageing* 2020; **17**: 100120.
264. Reddy AA, Mittal S, Roy NS, Kanjilal-Bhaduri S. Time Allocation between Paid and Unpaid Work among Men and Women: An Empirical Study of Indian Villages. *Sustainability* 2021; **13**(5): 17.
265. Sarker MR. Labor market and unpaid works implications of COVID-19 for Bangladeshi women. *Gend Work Organ*: 8.
266. IEA. World Energy Investment. Paris, France: International Energy Agency, 2021.
267. Buchko M. Global Coal Mining. 202.
268. Kanda S. Global Oil & Gas Exploration & Production. 2021.
269. 350.org. Divestment Commitments. 2020. https://gofossilfree.org/divestment/commitments/ (accessed 30 March 2021).
270. IEA. Energy subsidies - tracking the impact of fossil fuel subsidies. 2021. https://www.iea.org/topics/energy-subsidies (accessed 16th February 2021).
271. IEA. Energy subsidies. Methodology and assumptions - the price-gap approach. 2021. https://www.iea.org/topics/energy-subsidies#methodology-and-assumptions (accessed 10th April 2021).
272. OECD. OECD Inventory of support measures for fossil fuels. 2021. https://stats.oecd.org/Index.aspx?DataSetCode=FFS_AUS (accessed 6th April 2021).
273. OECD. OECD Companion to the Inventory of Support Measures for Fossil Fuels 2018. Paris, France: OECD Publishing; 2018.
274. World Bank. World Bank Carbon Pricing Dashboard. 2021. https://carbonpricingdashboard.worldbank.org/ (accessed 3rd April 2021).
275. IEA. International Energy Agency Carbon Dioxide (CO2) Emissions from Fuel Combustion, 1751-2019. [data collection]. 13th Edition. UK Data Service. SN: 5181. 2020. http://doi.org/10.5257/iea/co2/2020.
276. European Commission. Auctioning. 2020. https://ec.europa.eu/clima/policies/ets/auctioning_en (accessed 25 April 2020).
277. WHO. World Health Organization Global Health Expenditure Database. 2021. https://apps.who.int/nha/database/Select/Indicators/en (accessed 7th April 2021).
278. Mi Z, Zheng J, Meng J, et al. Economic development and converging household carbon footprints in China. *Nature Sustainability* 2020; **3**(7): 529-37.
279. Friedlingstein P, O'Sullivan M, Jones MW, et al. Global Carbon Budget 2020. *Earth System Science Data* 2020; **12**(4): 3269-340.
280. Crippa M, Oreggioni G, Guizzardi D, et al. Fossil CO2 and GHG emissions of all world countries. *Luxemburg: Publication Office of the European Union* 2019.
281. Benoit K, Watanebe K, Wang H, et al. quanteda: An R package for the quantitative analysis of textual data. *Journal of Open Source Software* 2018; **3**: 774.
282. Wickham H, Francois R, Henry L, Müller K. dplyr: A grammar of data manipulation. *R package version 04, 3* 2015.

283. HTCorrespondent. Gates steps down from board of Microsoft. Hindustan Times. 2020 15 March.
284. Boot M. The coronavirus response shows the costs of a 'chaos president'. The Washington Post. 2020 27 February.
285. Burns A, Martin J. Virus Takes Center Stage as Pence and Harris Skirmish in Debate. The New York Times. 2020 October 7.
286. Roberts ME, Stewart BM, Tingley D. stm: An R Package for Structural Topic Models. *2019* 2019; **91**(2): 40.
287. Tharoor I. The current health crisis should be a call to action for the climate crisis to come. The Washington Post. 2020 25 April.
288. Doyne S. What Are You Doing to Combat Pandemic Fatigue? The New York Times. 2020 October 27.
289. Lu MC. What the world can do to halt future pandemics. The Washington Post. 2020 21 April
290. HTCorrespondent. Need multi-sectoral coordination to tackle health impacts of climate change. Hindustan Times. 2020 February 25.
291. Chaturvedi A. How to deal with dengue during Covid-19? States come up with new protocols. Hindustan Times. 2020 19 July.
292. Brulliard K. 'Broken' balance with nature helped trigger pandemic. The Washington Post. 2020 11 September.
293. HTCorrespondent. Experts flag biodiversity loss link. Hindustan Times. 2020 15 March.
294. Nandi J. India lost 118 billion work hours due to heat in '19: Study. Hindustan Times. 2020 3 December.
295. Guterres A. A Time to Save the Sick and Rescue the Planet. The New York Times. 2020 April 28.
296. Pierre-Louis K. Heat Waves in the Age of Climate Change: Longer, More Frequent and More Dangerous. The New York Times. 2020 June 18.
297. Cimons M. Health risks for some drugs rise as temperatures climb. The Washington Post 2020 30 June.
298. Schwartz J. A season of climate-fueled disasters. The New York Times 2020 September 16.
299. Sengupta S. Hotter Planet Already Poses Fatal Risks, Health Experts Warn. The New York Times. 2020 2 December.
300. Freedman A, Samenow J, Dennis B. Global warming skeptic to run federal climate program The Washington Post. 2020 12 November.
301. Shuttleworth KA, J. As bush fires devastate Australia, a coal-friendly leader faces an early test. The Washington Post. 2020 9 January.
302. Fears DS, F. Kaplan, S. Eilperin, J. Heat is turbocharging fires, drought and tropical storms this summer. The Washington Post. 2020 23 August.
303. Frost N. Your Thursday briefing. The New York Times. 2020 2 December.
304. Dey S. 31,000 heat-related deaths of 65+ in India in 2018: Report. The Times of India. 2020 3 December.
305. Giles J. Internet encyclopaedias go head to head. *Nature* 2005; **438**(7070): 900-1.
306. Alexa. The top 500 sites on the Web. 2018. https://www.alexa.com/topsites.
307. Smith DA. Situating Wikipedia as a health information resource in various contexts: A scoping review. *PLOS ONE* 2020; **15**(2): e0228786.
308. Baturo A, Dasandi N, Mikhaylov SJ. Understanding state preferences with text as data: Introducing the UN General Debate corpus. *Research & Politics* 2017; **4**(2): 2053168017712821.
